# Internationalization tactics and enterprise value in the context of the "Belt and Road" initiative—Analysis from the perspective of host country institutional environment and enterprise digital transformation

**DOI:** 10.1371/journal.pone.0287324

**Published:** 2023-08-18

**Authors:** Dongchao Zhang, Xiyue Zheng, Fusheng Wang

**Affiliations:** 1 School of Economics, Liaoning University, Shenyang, Liaoning, China; 2 School of Management, Harbin Institute of Technology, Harbin, Heilongjiang, China; Nanjing Audit University, CHINA

## Abstract

This paper analyzes the impact of internationalization tactics on the value of 1107 multinational enterprises investing in the "Belt and Road" countries (regions) based on the two dimensions: entry mode and the degree of internationalization (including the breadth and depth of internationalization). It is found that the choice of sole proprietorship in the "Belt and Road" countries has a positive effect on enterprise value than joint venture. The increase of internationalization breadth and depth can promote enterprise value. Further research shows that the institutional environment of the host country and the digital transformation of enterprises significantly affect the choice of internationalization tactics and enterprise value. Specifically, the better the institutional environment of the host country and the more adequate the digital transformation of enterprises, the more inclined enterprises are to choose the sole proprietorship in the "Belt and Road" countries (regions) to obtain higher enterprise value. A good institutional environment of the host country and the digital transformation of enterprises can positively regulate the relationship between the degree of internationalization and enterprise value. these findings have important for Chinese enterprises to implement internationalization tactics rationally and scientifically, and to continuously improve enterprise value.

## Introduction

In recent years, with the increasing growth of Chinese companies and the saturation of the domestic market, visionary entrepreneurs have begun to focus on the global market [1, 2]. The world economy has shrunk by 3.3% since 2020 due to the severe impact of COVID-19. Despite the repercussion of the pandemic, Chinese companies still achieved positive growth in OFDI and ranked first in the world for the first time. Internationalization tactics have become a regular option for domestic companies. In 2013, the "Belt and Road" initiative provided a new opportunity for Chinese enterprises to optimize their international resource allocation. The energy-based countries along the route are highly complementary to China’s manufacturing-based economic structure, and their large population and market size provide feasible business value realization. However, as the complexity and changes of the world economic environment grow, and under the dual suppression of local wars and international trade wars, the overseas operations of Chinese enterprises along the "Belt and Road" are facing severe challenges, and many enterprises are "not going global", "afraid to go global" or even "unwilling to go global" due to the lack of clear internationalization tactics guidance. Although this "self-protective" business mode reduces the external risks faced by enterprises to a certain extent, it deprives enterprises of a large amount of overseas strategic resources and is not conducive to the realization of enterprise value. Based on this, this paper argues that it is important to investigate the value effects of Chinese enterprises’ internationalization tactics and to discuss how multinational enterprises can formulate scientific and reasonable internationalization tactics to achieve enterprise value-added, which is important to promote high-quality development of enterprises and better implement the "Belt and Road" initiative.

According to institutional theory, institutions are the key elements affecting the realization of enterprise value. As an external condition for the growth of multinational enterprises, the degree of perfection of the institutional environment in the host country is closely related to enterprise value [3]. A mature and stable institutional environment in the host country means open government and transparent information, which can reduce the risks of overseas business [4]. As is known to all, the emerging economies along the Belt and Road are not perfect in terms of the political system and resource endowment, and the fragile institutional environment will increase the risk and uncertainty of overseas investment. In this context, enterprises should reduce their investment in countries (regions) along the Belt and Road, but in fact, the scope of Chinese enterprises’ investment in these regions is still expanding, and the reasons behind this deserve our deep consideration. In addition, Chinese companies are experiencing novel technological changes. Intensive, high-throughput technology activities have turned into a core way for companies to add value [5]. Through digital transformation (e.g., artificial intelligence, data analytics, brain-based computing, and data visualization applications), companies can highly link various traditional nodes (e.g., linking consumer market demand, domestic and international innovation resources, etc.) [6], which maximally helps multinational companies reduce transaction costs in overseas markets and improve production efficiency. However, digital technology application is inherently challenging, and the high costs and uncertainty risks associated with digital transformation in enterprises can sap the value efficiency that digital transformation brings. Therefore, with the interplay of the positive and negative effects of digital transformation, the questions we should pay attention to include: what are the economic consequences of digital transformation for enterprises? Does it affect the relationship between internationalization tactics and enterprise value for multinational companies? The exploration of these questions has gradually become an important issue that requires urgent attention from scholars.

To solve the above problems, this paper analyzes the correlation between international tactics choice and the value of enterprises based on OFDI theory, institutional theory and digital technology application of enterprises. Using empirical data of multinational enterprises investing in the "Belt and Road" countries (regions) from 2010 to 2020. We analyze the correlation between the choice of internationalization tactics and the value of enterprises. We also take into account the internal and external environmental factors faced by enterprises in the process of value realization and analyze the moderating role of the institutional environment of the host country and the digital transformation of enterprises. Compared with the existing studies, the possible marginal utility of this paper mainly includes. First, providing empirical evidence and literature supplement for the study of the value-added effect of MNEs’ internationalization tactics. MNEs’ internationalization tactics mainly include two dimensions: entry mode and the degree of internationalization. Although there is a wealth of research on the degree of internationalization and enterprise value, the findings are widely divergent (positive, negative, and nonlinear). In addition, previous studies have been limited to analyzing the impact of MNEs’ internal and external environments (e.g., environmental uncertainty, firm resources, and transaction costs) on their entry mode choice, but few studies have examined the relationship between entry mode and enterprise value. Based on the above two shortcomings, this paper incorporates the rational choice of entry mode and the degree of internationalization together into the research framework of internationalization tactics, and attempts to comprehensively analyze the impact of MNEs’ internationalization tactics choice on value, enrich the research on the value-added effect of MNEs’ internationalization tactics. Second, the paper provides a sound theoretical interpretation for portraying the situational dependence of corporate internationalization tactics affecting enterprise value. In practice, a large number of multinational enterprises have relied on digital transformation to drive their international expansion and thus achieve global access to valuable resources. However, there is a gap in academic research on the role of digital transformation in the relationship between internationalization tactics and enterprise value. In addition, few previous studies have analyzed the important role of the host country’s institutional environment in the relationship between entry mode and enterprise value. This paper empirically examines the moderating role of the host country’s institutional environment (external factors) and the firm’s digital transformation (internal factors) in the relationship between internationalization tactics and enterprise value. The research framework of the relationship between the two is enriched. Third, This study deepens the contextualization of the "Belt and Road" investment. Previous studies were mainly based on Chinese A-share listed companies, ignoring the special characteristics of the "Belt and Road" investments. This paper focuses on analyzing the specific regional context of Chinese companies’ investment in the "Belt and Road", and discusses the antecedents and consequences of companies’ internationalization tactics in the "Belt and Road", so that the research results and recommendations can be more relevant and practical.

## 1 Theoretical analysis and research hypothesis

### 1.1 The degree of internationalization and enterprise value

Internationalization tactics embody the process of integrating high-quality elements and resources on a global scale beyond geographical and spatial boundaries [7], which mainly includes the degree of internationalization and the rational choice of overseas entry modes. The degree of internationalization represents the extent to which a firm operates internationally by investing in overseas assets or controlling activities abroad [8, 9]. In OFDI theory, Hymer’s Monopolistic Advantage Theory argues that MNEs invest in overseas markets based on their specific advantages to obtain monopoly profits [10]. Caves attribute this monopoly to the knowledge, capital, technology and scale advantages of MNEs and argue that it is based on these advantages that MNEs occupy the high end of the industrial value chain in imperfectly competitive markets [11]. The international production trade-off theory, which emphasizes factor endowments and locational advantages, argues that multinational enterprises achieve overseas market spillovers of core technologies and resources within the enterprise by taking advantage of the host market [12]. Based on the above investment theories, domestic and foreign scholars have conducted extensive studies to examine the regular connection between the degree of internationalization and enterprise value. Some scholars argue that enterprise value increases with increasing internationalization [13]. However, some scholars hold the opposite view that internationalization does not promote enterprise value or there is not a simple linear relationship between internationalization and enterprise value [14, 15]. It can be seen that extensive studies have not formed consistent conclusions and the regular connection between the degree of internationalization and enterprise value still needs further confirmation.

In the 1990s, the rapid development of developing countries and other emerging economies led to active overseas investment, and internationalization was considered a strategic choice for enterprises to continuously gain competitive advantages. The "Belt and Road" initiative has provided a new opportunity for enterprises to optimize the allocation of overseas resources. On the one hand, most of the countries along the "Belt and Road" are emerging economies with relatively low technology levels, so the "reverse technology spillover" effect obtained by enterprises through internationalization tactics is low, but the dense population and huge consumer market in the countries (regions) along the Belt and Road can bring enterprises greater profit growth space. However, the densely populated countries (regions) along the route have huge consumer markets that can bring enterprises greater profit growth, and the much-needed energy resources and cheap labor also reduce operating costs to a certain extent. By establishing subsidiaries in Belt and Road countries (regions), enterprises can obtain more resources such as innovative technologies, scarce materials and cheap labor. On the other hand, China, as the world’s largest emerging economy, has a fast economic development rate, and Chinese enterprises have advantages in terms of technology, organization, management and economies of scale, etc. The internal advantages such as target market selection and internationalization methods accumulated in the past internationalization process can also help to overcome the risks caused by external market failure.

To sum up, academic research on internationalization-enterprise value has not reached a unanimous agreement. However, this paper argues that investment in countries (regions) along the "Belt and Road" can help enterprises to obtain heterogeneous resources and utilize global resource endowments to create alliances and expand resource integration. In the complex international environment, it can also help enterprises to improve their learning dynamics, enhance the knowledge structure of their employees, and gain economies of scale quickly. Based on this, related hypotheses are proposed:

**H1:** The higher the internationalization of enterprises in "Belt and Road" countries (regions), the greater the enhancement of enterprise value.

Corporate internationalization cannot be achieved overnight, but a dynamic process of continuous deepening based on continuous learning and experience accumulation, which mainly includes internationalization breadth and internationalization depth [16]. On the one hand, internationalization breadth refers to the breadth of enterprises’ participation in overseas operations. Usually, the geographical diversity of investment host countries is considered to represent the breadth of information resources sought by enterprises in the world. As is well known, the population of countries (regions) along the "Belt and Road" accounts for about 2/3 of the total global population, and the huge overseas market can enable enterprises to quickly establish their economies of scale and scope, and the wide geographical distribution can also enable enterprises to diversify their investments into different "baskets" and reduce the risk of their overseas operations. In addition, with the continuous expansion of overseas markets, the diverse and extensive geographical distribution can help enterprises to upgrade their products while acquiring value resources, so that they can better cater to the consumer preferences of the host countries, thus promoting the enhancement of enterprise value. On the other hand, the depth of internationalization represents how effectively an enterprise is integrated into the host country. The stronger integration into the host country, the more comprehensive the enterprise’s knowledge of the host market, consumers and competitors, and the stronger the sustained learning benefits it can obtain [17]. In other words, the cultural and market differences between enterprises and host country markets and consumers are gradually reduced as the enterprise becomes more deeply integrated in the overseas host country [18]. Effective host country integration can help enterprises better grasp and integrate into different local cultures in the process of production, operation and organizational management, and the increased familiarity with the local area will also help enterprises grasp more information about host country consumers, suppliers and competitors, improve the chances of in-depth cooperation with local institutions in the host country, and provide expansion potential for enhancing corporate value. Based on this, two complementary hypotheses H1a and H1b are proposed.

**H1a:** The breadth of internationalization of Chinese enterprises in "Belt and Road" countries is positively related to enterprise value.
**H1b:** The depth of internationalization of Chinese enterprises in "Belt and Road" countries is positively correlated with enterprise value.

### 1.2 Entry mode and enterprise value

The "frontier issue" of MNEs’ internationalization tactics is the proper choice of the entry mode into foreign markets [19]. The choice is important for enterprises to overcome the multiple disadvantages of the host country’s institutional environment and to obtain sustained external compliance, internal rationalization and value enhancement [20]. According to institutional isomorphism theory, when different organizational units are faced with the same internal and external environments, there is a force that drives the organizational unit to be similar to other organizational units [21]. In the process of overseas investment, the institutional forces of the host country will have an impact on the behavior of the overseas subsidiary. To survive and gain external legitimacy, the overseas subsidiary will tend to choose to align with the institutional rules of the host country, i.e., "institutional isomorphism" to the host country [22]. On the contrary, when there is a strong interdependence between the parent company and the overseas investment subsidiary, the subsidiary’s behavior will tend to mimic the established behavior and pattern under the domination of the parent company, and it will seek internal legitimacy by entering through a sole proprietorship, i.e., "institutional isomorphism" to the parent company [23].

Entry modes to the market can have an impact on companies’ innovation and business performance [24]. On the one hand, MNEs face the (interactive) problem of integrating the target firm after the completion of the investment transaction. Typically, joint ventures are considered as contractual alliances with small investment sizes, and the parent company keeps most of its assets to ensure its operations while only contributing some of them to set up subsidiaries abroad. As a result, the joint venture is relatively less resilient to external risks, and if the host country takes strong defensive measures, the firm is likely to choose to terminate its overseas operations due to a lack of capital. Compared with joint ventures, the sole proprietorship implies that the enterprise devotes more resources to overseas markets and has a relatively high proportion of resource integration, which can guarantee that the overseas subsidiaries have sufficient capital sources to cope with the higher information search and processing costs in the host country, and are often more beneficial in promoting enterprise value enhancement. Some scholars believe that if enterprises want to obtain more innovative resources and technologies in overseas markets, enterprises with full ownership are more advantageous than those with partial ownership [25].

On the other hand, the differences in the institutional environment between China and the countries along the "Belt and Road" will make the overseas subsidiaries rely more on the technology and management experience of the parent company to mitigate the impact of the diverse cultures on the internal management model and the external market trading activities. In contrast to sole proprietorships, joint venture partners deliberately protect their core technologies and even adopt "secrecy" measures to prevent plagiarism by their partners, which to a certain extent inhibits the enhancement of corporate value. A sole proprietorship entry mode does not involve such an antagonistic relationship, and the knowledge and business philosophy can be accurately transferred within the organization. Therefore, compared with the joint venture entry mode, the enterprise’s choice of the sole proprietorship entry mode has a more significant effect on enterprise value. Based on this, the related hypothesis is proposed:

**H2:** The enhancement of enterprise value is more significant when enterprises choose the sole proprietorship entry mode than the joint venture entry mode in "Belt and Road" countries.

### 1.3 Moderating role of the institutional environment

Institutional theory suggests that no business can operate in a ’vacuum’ and that the institutional environment (both formal and informal) is part of the external environment to which a business must adapt [26]. Institutional environment refers to a set of basic rules used to establish the national economic cycle of production, exchange, consumption and distribution. It covers political, social, legal and cultural institutional arrangements with obvious state-imposed characteristics and geographical features. The process by which a country’s institutional environment is formed and how well it is developed can be influenced by various factors such as political stability, discourse accountability, government efficiency, quality regulation, legal rules and corruption control (World Bank, 2010). Therefore, when exploring the correlation between an enterprise’s internationalization tactics and enterprise value, the moderating role of the host country’s institutional environment must also be examined.

From the perspective of the internationalization of the enterprise, the premise that MNEs can obtain enterprise value effects through diverse geographical distribution is that the host country system nurtures resources that are accessible to enterprises and a favorable market environment. As enterprises expand their business scope in Belt and Road countries (regions), they need to deal with different types of institutional environments. A sound institutional environment in the host country can reduce the risk of overseas operations and provide a basic guarantee for the survival of the enterprise. The "Belt and Road" countries are mainly developing countries, with obvious differences in political stability among them. If companies want to access heterogeneous resources through geographically dispersed investments, they need a stable political environment and the rule of law in the host country. Investing in a better environment can save companies from spending a lot of time dealing with multicultural conflicts and reduce uncertainty risks and transaction costs for companies. In other words, the better the host country’s institutional environment, the more heterogeneous resources the geographic diversification of the investment can help firms to access, and the more significant the value-enhancing effect on the firm. Conversely, when the institutional environment is poor, e.g. Nepal, Afghanistan, etc., where long-term social unrest will inhibit the survival and development space of enterprises. The diversified geographical distribution of enterprises’ investments will distract enterprises from devoting more energy to obtaining the legitimacy of the host country’s system rather than to obtaining high profits, which is detrimental to the realization of enterprise value.

A well-developed institutional environment can help enterprises quickly gain "legitimacy" in the host country so that they will not be less integrated in the host country for fear of political instability or inadequate market regulation [27]. The better the institutional environment, the more convenient services (e.g. intermediary services, complaint service networks and other external support) enterprises can enjoy, and the stronger the willingness of enterprises to invest in [28]. Some scholars conducted an empirical analysis of Chinese enterprises’ overseas investment performance and found that the high-quality institutional environment of countries along the Belt and Road has a significant impact on the performance of investing enterprises, which can accelerate the pace and improve the performance of Chinese enterprises [29, 30]. Conversely, when the institutional environment in the host country is poor and unpredictable, the business environment is not good, and there is prolonged political instability and lack of resources, the enterprises will face higher external risks. This is detrimental to the enhancement of corporate value. Based on this, the relevant hypothesis is proposed:

**H3a:** A well-developed institutional environment in the "Belt and Road" countries will have a positive moderating effect on the relationship between the breadth of internationalization and the enterprise value.
**H3b:** A well-developed institutional environment in the "Belt and Road" countries will have a positive moderating effect on the relationship between the depth of internationalization and enterprise value.

From the perspective of the entry mode, as an economic organization integrated into an institutional environment, MNEs should choose their international market entry mode according to the institutional environment of different countries to enhance their enterprise value more effectively [31]. When an organization initially invests abroad, its organizational form and business model are established under the premise of parent company regulation. The sole proprietorship mode represents a higher degree of investment in resources. The company can have complete control and profitability over the operations of its overseas subsidiaries. The parent company can make full use of international experience to make a long-term layout of its overseas subsidiary operations without having to consider the restrictive conditions of its partner companies. To this end, the company increases its efficiency in expanding into the host market or developing new technologies and enjoys exclusive access to expansion opportunities and potential benefits [32]. However, the sole proprietorship mode can also increase the external risks of the business. When the institutional environment is hostile, sole proprietorship is left alone to face restrictions, discrimination or unfair treatment due to the ever-changing quality of government regulation and government stakeholders [33]. To reduce the cost of uncertainty and opportunistic (free-rider) risk of a firm’s overseas investment, sole proprietorship requires more transaction and time costs in the overseas investment process, which adversely affects enterprise value. Conversely, when the institutional environment in the host country is better, the external risks faced by the enterprise are lower. Good quality regulation, corruption control or government efficiency in the host country will reduce the cost of the enterprise’s exposure to risk alone, and the sole proprietor can then devote more energy to product development, market research and sales channels, which enhance the value of the enterprise.

Under the joint venture mode, MNEs need to share the control and benefits of their subsidiaries with their partners. When the institutional environment is hostile, the formation of a joint venture forms a community of interest with the host country, using the "legitimacy spillover" effect of the joint venture to quickly establish ties with powerful stakeholders such as the host government [34] ensuring that the enterprise can realize the complementary advantages of both sides while reducing the investment risks faced in the host country. This ensures that the company can achieve complementary advantages while reducing the investment risks faced in the host country and promoting a steady increase in enterprise value. In addition, close ties between partners can also help MNEs to quickly gather information about potential changes in the host country’s policies [35] and thus gain quick access to inexpensive local supplies of materials, cheap land rents and sound infrastructure services [36] and have a positive impact on the enhancement of corporate value. Conversely, when the institutional environment of the host country is favorable, the external risks faced by the enterprise are less. The joint venture needs the support of its partners in formulating its overseas market strategy, which does not fit perfectly with the long-term goals of the enterprise, and the benefits obtained need to be shared with the partners, thus weakening the positive impact on enterprise value to a certain extent. Based on this, the following hypotheses are proposed:

**H4a:** The better the institutional environment of the countries along the Belt and Road, the greater the effect of the sole proprietorship mode on enterprise value.
**H4b:** The better the institutional environment of the countries along the Belt and Road, the smaller the effect of joint venture mode on enterprise value.

### 1.4 Moderating role of digital transformation

Digital transformation represents the highest level of digital application and refers to the process of deep integration of digital technology with traditional business modes by relying on blockchain, big data and other methods to process business operations, production links, business modes and strategic thinking in the operation process of enterprises. Theoretically, technological innovation is an important factor in the production function of enterprises, and as a non-physical factor input, the level of technological innovation affects the output level and value of enterprises. The digital confidence technology represents a new round of technological change, and is a key object to be examined when studying the factors affecting enterprise value.

From the perspective of the internationalisation of enterprises, the extensive use of digital technology by MNEs not only reduces the barriers to trade in multiple countries, but also promotes the deep integration between enterprises and host countries. On the one hand, the geographic diversity of host countries will make it more difficult and costly for enterprises to understand market information. If enterprises rely on traditional technology alone, they will not be able to collect, store and process the massive amount of market information from multiple host countries, and the reduced sensitivity to market demand will weaken the positive impact of the breadth of internationalization on the enterprise value. However, through digital transformation, companies can complete the mapping and reconstructing of the physical world in virtual space, helping them to gain more information about the host country market, consumer preferences and competitors, and efficiently achieve relevance discovery and value extraction. Companies can use digital technology to better achieve a zero-distance interaction with stakeholders in the host country, quickly capture host country market demand, develop and design products that exceed customer expectations and thus increase enterprise value [37].

On the other hand, digital transformation can contribute to the impact of the depth of internationalization on enterprise value. The "outsider disadvantage" of operating deeply in a host country can cost companies a lot of time to gain "legitimacy" in the host country, which reduces the efficiency of resource allocation and information gathering in the host country. Companies that have undergone digital transformation are able to access and utilize the hidden resources in the host country through mobile connectivity, machine learning and data mining, enabling them to better anchor their operations in the host countryassert that reliance on the openness and real-time transparency of digital platforms can facilitate efficient data collaboration and resource layout [38] and stimulate the interweaving and convergence of data value chain nodes across R&D, production and suppliers, thereby breaking down the barriers of information asymmetry with the host country. In addition, the extensibility and openness of digital technology will also help enterprises exchange information with relevant enterprises and governments in the host country, enabling them to search for information resources that meet their stated innovation objectives in the shortest possible time and at the lowest possible cost. Based on this, the relevant hypothesis is proposed:

**H5a:** Digital transformation will have a positive moderating effect on the relationship between the breadth of an enterprise’s internationalization and its enterprise value.
**H5b:** Digital transformation will have a positive moderating effect on the relationship between the depth of internationalization and enterprise value.

From the perspective of entry mode, the relationship between entry mode and enterprise value is influenced by the digital transformation of the company. In general, when the digital transformation of a company is relatively high, a sole proprietorship will capture a higher enterprise value than a joint venture. First, a company that chooses to operate as a sole proprietor as opposed to a joint venture has to take on the risks associated with uncertainty (e.g. unfamiliarity with the host government, consumers, culture and institutions) for its managers. A sole proprietor with a high degree of digital transformation can use digital technology to access more explicit and implicit resources in the host country (e.g. information on local regulations and policies, consumer habits and preferences, etc.), helping the company to better develop partnerships with local governments, suppliers and distributors, reducing the "outsider disadvantage" faced by the sole proprietor in the host country. In this way, they can create a comparative advantage in terms of status and structural equivalence in the process of cooperation with host country companies. Second, compared to joint venture, sole proprietors have greater autonomy in the process of overseas production and operation and can effectively control their comparative advantage within the organization. Not only can they independently choose to enter the host market with higher added value, they can also choose to use digital technology applications to integrate stakeholders into the whole process of product design and development [39], enabling them to independently choose to follow the overall strategic layout of the company by its stated objectives, thus using digital customer orientation to shape high growth potential or create strategic value [40]. Conversely, if a company is less digital and slower to capture information about the host market, it will face more pressure to imitate the host country or industry, and will then prefer to adopt a joint venture entry mode to reduce risk and capture value efficiency. However, as joint ventures may be in a relatively weak position and it will increase the risks and costs of enterprises in the cooperative operation, thus the effect on the enhancement of enterprise value will be relatively slow. Based on this, the relevant hypothesis is proposed:

**H6:** Compared to the entry mode of joint ventures, enterprises with a higher degree of digital transformation can have a greater effect on the enhancement of enterprise value by choosing the sole proprietorship mode.

**Fig 1:**
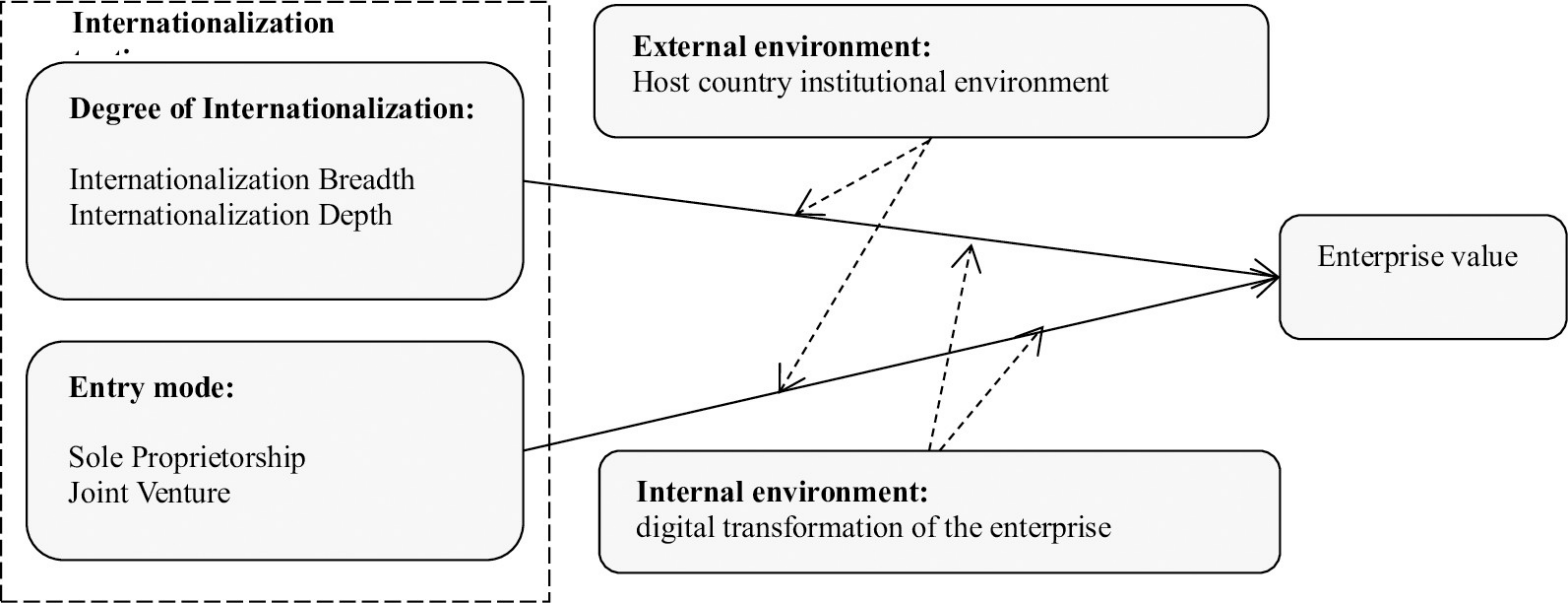
Logical framework.

Based on the above theoretical analysis and research hypothesis, this paper constructs the theoretical framework as shown in Fig 1.

## 2 Study design

### 2.1 Data sources

This paper uses the 2010–2020 data of MNEs listed on A-shares in China as the sample to conduct an econometric analysis of the relationship between internationalization tactics, host country institutional environment, digital transformation and enterprise value. Specifically, we use data from the CSMAR database on OFDI enterprises listed in China and the Ministry of Commerce’s Foreign Investment Enterprises List database to initially identify the "list of multinational enterprises engaged in OFDI and listed in China". Firstly, based on the perspective of "Belt and Road" countries, enterprises investing in non- "Belt and Road" countries (regions) were excluded; secondly, listed companies that were ST or * ST during the data extraction period and those registered in "tax havens" were excluded, and a sample database consisting of 1107 companies with 4035 observations were finally obtained.

### 2.2 Variable settings

#### 2.2.1 Explained variable: Enterprise value (ROA)

Most existing studies use Return on Assets (ROA) and Tobin’Q to measure the market value of a company. However, Tobin’Q is the market value of the company/replacement cost. Considering the imperfection of the Chinese bond market and the volatility of the stock market, we finally choose ROA to measure the enterprise value.

#### 2.2.2 Explanatory variables: Internationalization tactics (IT)

In this paper, we measure the internationalization tactics of companies in terms of two dimensions: the degree of internationalization and the entry mode.

1. Degree of Internationalization (DOI). Drawing on existing research, we divide the degree of internationalization into breadth of internationalization and depth of internationalization and reconstruct the measurement indicators [23, 41]. defined as follows.
DOI=Breadth*50%+Depth*50% (1)
  In the above equation, Breadth represents the internationalization breadth of a company, reflecting the geographical diversity of its overseas subsidiaries. This paper uses the number of enterprises investing in host countries as an indicator of geographical diversity; Depth represents the internationalization depth, reflecting the degree of integration of enterprises’ overseas branches in host countries, and this paper uses the average number of enterprises setting up subsidiaries in host countries as an indicator of enterprises’ internationalization depth.
2. Entry mode (Mode). This paper uses binary categorical variables to classify the entry mode of enterprises. The sole proprietorship entry mode takes the value of 1 and the joint venture is 0.

#### 2.2.3 Moderating variables

(1) Host country institutional environment (WGI). The data on the host country’s institutional environment comes from the World Governance Indicators published by the World Bank and contains six items, namely: Voice Accountability (Voice A), Regime Stability (Reg Q), Government Efficiency (Pol-SNV), Regulatory Quality (Gov E), Rule of Law (Rule Law) and Control of Corruption (ConCor) [42]. The specific measures are as follows:

$$WGI=\sum_{avr}\left(Voice\;A,Reg\;Q,Pol-SNV,Gov\;E,Rule\;Law\&ConCOr\right)$$ (2)

(2) Digital transformation (Digital). To measure the degree of digital transformation of enterprises using the frequency of occurrence of their keyword segments. Specifically: firstly, we crawled the annual reports of high-tech enterprises that implemented overseas investment from 2010–2020 in Juchao.com through Python software; secondly, we used Python’s Jieba Chinese word-sorting function to filter out words related to the digital development of enterprises (Table 1) and conducted word frequency statistics; finally, we used the word frequency data obtained by summing up to measure the digital transformation of enterprises index [43].

**Table 1:** Summary of digital transformation keywords.

Items	
Artificial Intelligence Technology	Machine learning, artificial intelligence, face recognition, business intelligence, identity verification, deep learning, biometrics, image understanding, semantic search, speech recognition, intelligent robotics, intelligent data analysis, autonomous driving, natural language processing
Blockchain Technology	Bitcoin, distributed computing, consensus mechanisms, federated chains, decentralization, digital currencies, smart contracts
Cloud Computing Technology	EB-class storage, multiparty secure computing, brain computing, stream computing, green computing, in-memory computing, cognitive computing, converged architectures, graph computing, Internet of Things, information physical systems, billion dollar concurrency, cloud computing
Big Data Technology	Big Digital, Mixed Reality, Data Visualization, Data Mining, Text Mining, Virtual Reality, Heterogeneous Data, Augmented Reality, Credit
Applications of Digital Technology	B2B, B2C, C2B, C2C, Fintech, NFC payment, O2O, third party payment, e-commerce industrial internet, internet finance, internet healthcare, fintech, open banking, quantitative finance, digital finance, digital marketing, netlink, unmanned retail, mobile internet, mobile internet, mobile payment, smart agriculture, smart wear, smart grid, smart environmental protection Smart Home, Smart Transportation, Smart Customer Service, Smart Energy, Smart Investment, Smart Culture and Tourism, Smart Healthcare, Smart Marketing

#### 2.2.4 Control variables

In conjunction with the nature of the research question, this paper also examines several control variables that affect the realization of enterprise value. The first is the age of the business (Age). In a volatile market and macro environment, the long-term survival of a company is a sign of resilience and adaptability. In this paper, we use the survival period from the year of registration of the company to the year of observation as the age of the company [44]. Second, capital structure. Capital structure can reflect the composition of the enterprise’s capital sources and is an important indicator affecting the realisation of enterprise value. In this paper, the enterprise’s gearing ratio (DEBT) is selected to measure it. Third, the operational capability of the enterprise. Operating capability is the basis for the achievement of enterprise value objectives, and good operating capability can improve the market share of enterprise products and promote the achievement of enterprise value. This paper draws on the existing literature and uses total asset turnover (TAT) as a measure of the operating capability of an enterprise [45]. Companies need to overcome the ’unfamiliarity’ of outsiders to the institutional environment. In this paper, the following formula is used to measure cultural distance (CD) [46]:

$${CD}_{j}=\sum_{i=1}^{6}[\frac{{\left(I_{ij}-I_{ih}\right)}^{2}}{V_{i}}]/6$$ (3)

Where CD_j_ denotes the cultural distance between the j-th country and China; I_ij_ is the i-th cultural dimension index of the j-th country, and I_ih_ represents the cultural dimension index of China; Vi is the variance of the i-th dimension index.

### 2.3 Econometric model

First, to test the impact of internationalization tactics on enterprise value, the basic model (4) is constructed in this paper. If the coefficient of the degree of internationalization (DOI) is significantly positive, it indicates that the degree of internationalization has a catalytic effect on enterprise value, which supports the research hypothesis H1. If the coefficient of the entry mode (Mode) is positive, it indicates that the entry of sole proprietorship mode into "Belt and Road" countries has a more significant effect on enterprise value than the entry of joint venture mode, which supports the research hypothesis H2. In addition, since the degree of internationalization (DOI) has two sub-variables Breadth and Depth, we introduce two variables Breadth and Depth in the model (4) to replace DOI in the model respectively. based on hypotheses H1a, H1b. We expect that the model coefficients of Breadth and Depth have significantly positive coefficients. The specific settings are as follows:

$${ROA}_{it}=\beta_{0}+\beta_{1}{DOI}_{it-1}+\beta_{2}{Mode}_{it-1}+\beta_{3}{Control}_{it-1}+\mu_{i}+\nu_{t}+\varepsilon_{it}^{1}$$ (4)

Second, we add the interaction terms of the degree of internationalization (DOI) and host country institutional environment (WGI) and entry mode (Mode) and host country institutional environment (WGI) to the basic model (4) to construct model (5), to determine whether there is an interaction between host country institutional environment and internationalization tactics in influencing firm value. Since the degree of internationalization (DOI) has two subdivision variables of internationalization breadth (Breadth) and internationalization depth (Depth), we introduce two interaction terms Breadth×WGI and Depth×WGI to replace DOI×WGI in the model (5) respectively. based on hypotheses H3a, H3b and H4a, H4b. We expect the model coefficients of the interaction terms for both the host country’s institutional environment and internationalization tactics to be significantly positive. This is set as follows:

$${ROA}_{it}=\beta_{0}+\beta_{1}{DOI}_{it-1}+\beta_{2}{Mode}_{it-1}+\beta_{3}{WGI}_{it-1}+\beta_{4}{{DOI}_{it-1}*WGI}_{it-1}+\beta_{5}{{Mode}_{it-1}*WGI}_{it-1}+\mu_{i}+\nu_{t}+\varepsilon_{it}^{2}$$ (5)

Finally, we build a model (6) by adding an interaction term between the degree of internationalization (DOI) and the digital transformation (Digital), the mode of entry (Mode) and the digital transformation (Digital) to the basic model (4). Model (6) is used to determine the impact of digital transformation and internationalization strategy on the value of enterprises. Since the degree of internationalization (DOI) includes the breadth of internationalization (Breadth) and depth of internationalization (Depth), we introduce Breadth×Digital and Depth×Digital to replace DOI×WGI in the model (6) to test hypotheses H5a, H5b, and H6, respectively. we expect that the coefficients of the interaction term between digital transformation and the internationalization strategy of firms in the model The coefficients of the interaction terms are significantly positive. The specific settings are as follows:

$${ROA}_{it}=\beta_{0}+\beta_{1}{DOI}_{it-1}+\beta_{2}{Mode}_{it-1}+\beta_{3}{Digital}_{it-1}+\beta_{4}{{DOI}_{it-1}*Digital}_{it-1}+\beta_{5}{{Mode}_{it-1}*Digital}_{it-1}+\mu_{i}+\nu_{t}+\varepsilon_{it}^{3}$$ (6)

In this paper, the models (4)-(6) are estimated using Stata 15.1 software. Considering the existence of both cross-sectional and time series in the panel data, we adopt the GLS model to analyze and test the data. The GLS model can solve the autocorrelation and heteroskedasticity problems well and ensures the quality of the analysis.

## 3 Empirical results and analysis

Based on the above model, this paper selects 1107 enterprises that first invested in "Belt and Road" countries from 2010 to 2020 as the object of analysis. These enterprises invested in 46 countries (regions), accounting for 71% of the total number of " Belt and Road" countries (regions). Enterprises’ first investment in Belt and Road countries was mainly concentrated from 2016 to 2020, accounting for 67.07% of the total sample. Investment regions are mainly concentrated in countries and regions with a stable institutional environment, such as India, Russia, Japan and South Korea, while Central Asia and West Asia have fewer investors due to political instability, which to a certain extent indicates that the institutional environment of the host country affects the internationalization of enterprises.

Table 2 shows the descriptive statistics of the sample. Considering the special characteristics of state-owned enterprises (SOEs) that may respond to national policy requirements to invest in the Belt and Road, this paper divides the sample into SOE and private enterprise groups. By comparing the core variables between groups, we find that: firstly, the mean value of ROA for SOEs is 3.82, which is lower than that of 5.30 for private enterprises, indicating that private enterprises can obtain higher enterprise value by investing in Belt and Road countries (regions) compared to SOEs. This may be because SOEs have obvious "non-market motives" when investing overseas, and their internationalization tends to prioritize national macro policy objectives rather than the pursuit of "profit maximization", while private enterprises are relatively flexible in their investment approach and more efficient in their use of resources, making it easier to promote enterprise value. Secondly, in terms of entry mode, the average value of Mode for SOEs is 0.64, while that for private enterprises is 0.69. This indicates that 64% of SOEs choose the sole proprietorship mode, while 69% of private enterprises do. This shows that the entry mode of Chinese enterprises investing in "Belt and Road" countries (regions) is dominated by sole proprietorship entry mode. Thirdly, from the internationalization index, the mean value of the DOI of SOEs is 4.57, which is greater than that of private enterprises at 3.85, indicating that SOEs are more inclined to adopt the "going global" strategy due to the influence of national policies.

**Table 2:** Results of descriptive statistics and group tests for variables.

Variables	Owner = 1 (SOEs)				Owner = 0 (private enterprise)			
	Mean Value	SD	Maximum value	Minimum value	Mean Value	SD	Maximum value	Minimum value
ROA	3.82	5.16	-44.84	22.83	5.30	7.18	-77.47	59.81
Mode	0.64	0.48	0.00	1.00	0.69	0.46	0.00	1.00
DOI	4.57	3.01	1.00	18.77	3.85	3.38	1.00	22.53
WGI	1.66	1.33	0.10	7.69	0.17	0.11	0.01	2.08
Digital	16.49	29.99	0.00	267.00	15.75	30.39	0.00	338.00
Age	20.47	6.60	3.00	50.00	19.23	5.21	6.00	40.00
DEBT	2.79	6.59	0.12	152.21	2.95	54.45	0.05	1767.97
TAT	0.75	0.46	0 .02	4.52	0.75	0.45	0 .01	4.56
CD	2.72	1.21	0 .82	5.83	2.56	1.11	0 .84	5.83
N	1929				2106			

Table 3 provides the correlation tests and multiple co-integration analysis of the variables. The results show that the correlation coefficients between the main variables are all below 0.32, and all have varying degrees of correlation with firm value (ROA). For example, the degree of firm internationalization (DOI) may have a linear positive relationship with firm value (ROA), but still needs regression analyses.

**Table 3:** Correlation tests and multiple co-integration analysis of variables.

Variables	ROA	Mode	DOI	Digital	WGI	Age	DEBT	TAT	VIF
ROA									1.07
Jvs	0.04***								1.04
DOI	0.11***	0.11***							1.25
Digital	0.11***	0.10***	0.32***						1.14
WGI	0.03*	-0.01	0.27***	-0.02					1.11
Age	-0.13***	0.03**	0.10***	0.02	0.13***				1.03
DEBT	-0.09***	-0.01	0.00	-0.00	-0.01	0.05***			1.00
TAT	0.24***	0.02	0.02	0.04***	0.01	-0.05***	-0.01		1.01
CD	0.03*	-0.13***	0.11***	0.04**	0.04**	0.02	-0.01	-0.01	1.03

**Notes:** *p<0.1,**p<0.05,***p<0.01; N = 4035; Source: Calculations based on Stata 15.1 software. Same as below.

### 3.1 Internationalization tactics and enterprise value

In this paper, a fixed effects model is selected for the empirical analysis through the Hausman test. Table 4 presents the regression results of internationalization tactics and enterprise value. Model 1 is the base model with only four control variables: firm age (Age), capital structure (DEBT), operational capability (TAT) and cultural distance (CD) on firm value (ROA). Model 2 adds the degree of internationalization (DOI) to test whether hypothesis H1 is valid. Models 3 and 4 report the regression results of Breadth and Depth of internationalization on enterprise value to test the validity of hypotheses H1a and H1b respectively. In addition, to test the validity of hypothesis H2, we include Mode in model 5 to test the impact of different entry modes on enterprise value.

**Table 4:** Results of regression analysis.

Variables	Model 1	Model 2	Model 3	Model 4	Model 5
Age	-0.13*** (0.03)	-0.13*** (0.03)	-0.13*** (0.03)	-0.13*** (0.03)	-0.12*** (0.03)
DEBT	-0.01*** (0.00)	-0.01*** (0.00)	-0.01*** (0.00)	-0.01*** (0.00)	-0.01*** (0.00)
TAT	5.59*** (0.33)	5.44*** (0.33)	5.45*** (0.33)	5.58*** (0.33)	5.60*** (0.33)
CD	0.29** (0.13)	0.23* (0.13)	0.24* (0.13)	0.28** (0.13)	0.32** (0.13)
DOI		0.18*** (0.04)			
Breadth			0.09*** (0.02)		
Depth				0.24* (0.14)	
Mode					0.78** (0.34)
Year	Yes	Yes	Yes	Yes	Yes
Industry	Yes	Yes	Yes	Yes	Yes
cons	2.61 (2.15)	2.42 (2.11)	2.52 (2.11)	2.33 (2.15)	1.96 (2.16)
Wald chi^2^	596.9***	622.1***	620.1***	600.6***	602.8***
R-square	0.1616	0.1738	0.1727	0.1642	0.1643

**Notes:** Standard errors in parentheses

*p<0.1

**p<0.05

***p<0.01

In model 1, firm age (Age), gearing (DEBT) and enterprise value are significantly negatively related, i.e., high gearing is associated with low enterprise value. Longer established companies are worth less than younger companies, which indicates that older companies are not an advantage for overseas investment. Companies with the same degree of internationalization, established for a long time, will be bottlenecked in the development of overseas investment due to the lack of innovation power and transformation difficulties. The operating capacity (TAT) of companies is significantly and positively correlated with enterprise value, indicating that companies with higher operating capacity have a higher value. In addition, at the country level, cultural distance (CD) between home and host countries is significantly and positively correlated with enterprise value, as a diverse and different culture can give firms access to more heterogeneous resources, thus positively influencing enterprise value.

Model 2 adds DOI. The regression results show that the coefficient of DOI is significantly positive at the 1% level (β = 0.18, p<0.01), indicating that the degree of internationalization positively affects the realization of enterprise value and hypothesis H1 is tested. Further, the coefficient of the breadth of internationalization (Breadth) in model 3 is significantly positive at the 1% level (β = 0.09, p<0.01), and the coefficient of the depth of internationalization (Depth) in model 4 is significantly positive at the 10% level (β = 0.24, p<0.10). This indicates that when multinational enterprises invest in "Belt and Road" countries (regions), both the increase in internationalization breadth and depth can have a positive effect on enterprise value. Model 5 examines the impact of entry mode on enterprise value, and the results show that the coefficient of Mode is significantly positive at the 5% level (β = 0.78, p<0.05), and since the entry value of sole proprietorship mode in this paper is 1, the positive coefficient of entry mode indicates that the entry of multinational enterprises’ sole proprietorship mode into "Belt and Road" countries (regions) is more significant than that of joint venture mode, which verifies the validity of hypothesis H2 of this paper.

### 3.2 Moderating effects test

Table 5 reports the moderating role of the host country’s institutional environment and digital transformation between internationalization tactics and enterprise value during overseas investment by multinational firms. Model 1 is the base model, with only four control variables added to the regression of firm value. Models 2 and 3 include interaction terms between the breadth and depth of internationalization and the host country’s institutional environment to examine the moderating effect of the host country’s institutional environment on the relationship between the degree of internationalization and enterprise value. The regression results show that the coefficient of Breadth×WGI is significantly positive at the 10% level (β = 0.05, p<0.10), which indicates that the better the institutional environment of the host country, the greater the effect of the breadth of internationalization on the enhancement of firm value, validating hypothesis H3a. In model 3, we include the interaction term between the depth of firm internationalization and the host country institutional environment (Depth × WGI), and the regression coefficient is significantly positive at the 5% level (β = 0.98, p<0.05), indicating that a well-developed host country institutional environment can positively influence the relationship between internationalization depth and enterprise value. That is, the higher the level of institutional environment in the host country, the more significant the contribution of internationalization depth to enterprise value, verifying hypothesis H3b. In model 5, we include a cross-sectional term between the entry mode and host country institutional environment to test the moderating effect of the host country institutional environment on entry mode and enterprise value, and the results show that the coefficient of Mode×WGI is significantly positive at the 5% level (β = 0.43, p<0.05). As the sole proprietorship model is set to 1 in this paper, a positive regression coefficient indicates that a sound host country institutional environment positively moderates the relationship between sole proprietorship entry mode and firm value, and hypotheses H4a and H4b are tested.

**Table 5:** Regression analysis results.

Variables	Model 1	Model 2	Model 3	Model 4	Model 5	Model 6	Model 7
Age	-0.13***	-0.13***	-0.13***	-0.13***	-0.12***	-0.12***	-0.12***
	(0.03)	(0.03)	(0.03)	(0.03)	(0.03)	(0.03)	(0.03)
DEBT	-0.01***	-0.01***	-0.01***	-0.01***	-0.01***	-0.01***	-0.01***
	(0.00)	(0.00)	(0.00)	(0.00)	(0.00)	(0.00)	(0.00)
TAT	5.59***	5.44***	5.57***	5.59***	5.46***	5.59***	5.57***
	(0.33)	(0.33)	(0.33)	(0.33)	(0.32)	(0.33)	(0.33)
CD	0.29**	0.23*	0.28**	0.31**	0.22*	0.27**	0.31**
	(0.13)	(0.13)	(0.13)	(0.13)	(0.13)	(0.13)	(0.13)
WGI		2.30	-16.86*	2.05			
		(1.93)	(9.25)	(1.79)			
Digital					-0.01**	-0.04**	-0.01
					(0.00)	(0.02)	(0.01)
Mode				0.70			0.56
				(0.51)			(0.36)
Depth			-0.16*			-0.06	
			(0.10)			(0.07)	
Breadth		0.10**			-0.01		
		(0.04)			(0.03)		
Breadth×WGI		0.05*					
		(0.21)					
Depth×WGI			0.98**				
			(0.46)				
Breadth×Digital					0.01***		
					(0.00)		
Depth×Digital						0.01**	
						(0.00)	
Mode×WGI				0.43**			
				(2.15)			
Mode×Digital							0.01*
							(0.01)
Year	Yes	Yes	Yes	Yes	Yes	Yes	Yes
Industry	Yes	Yes	Yes	Yes	Yes	Yes	Yes
cons	2.610	2.24	5.37*	1.75	2.93	3.70	2.17
	(2.15)	(2.13)	(2.78)	(2.18)	(2.08)	(2.43)	(2.15)
Wald chi^2^	596.9***	622.6***	606.2***	606.1***	648.6***	609.1***	610.9***
R-square	0.1616	0.1744	0.1668	0.1666	0.1851	0.1679	0.1682

**Notes:** Standard errors in parentheses

*p<0.1

**p<0.05

***p<0.01

Models 5–7 demonstrate the moderating effect of corporate digital transformation on the relationship between internationalization strategy and firm value. In particular, model 5 tests the moderating effect of corporate digital transformation in the relationship between internationalization breadth and enterprise value, and the regression results show that the Breadth×Digital coefficient is significantly positive at the 1% level (β = 0.01, p<0.01), indicating that the more adequate corporate digital transformation is, the greater the effect of internationalization breadth on enterprise value, validating hypothesis H5a. In model 6, we include the cross-sectional variable of the depth of internationalization and enterprise digital transformation, and the regression results show that the coefficient of Depth×Digital is significantly positive at the 5% level (β = 0.01, p<0.05), indicating that enterprise digital transformation can positively influence the relationship between depth of internationalization and enterprise value. That is, the higher the degree of digital transformation of a company, the more significant the contribution of the depth of internationalization to enterprise value, thus, hypothesis H5b was verified. In model 7, we include a cross term between firm entry mode and digital transformation to test the moderating effect of firm digital transformation on the relationship between entry mode and enterprise value, and the results show that the coefficient of Mode×Digital is significantly positive at the 10% level (β = 0.01, p<0.10), and since the sole proprietorship mode is set to 1 in this paper, a positive regression coefficient indicates that digital transformation positively moderates the relationship between sole proprietorship entry mode and enterprise value, and hypothesis H6 is also verified.

### 3.3 Robustness tests

To test the robustness of the empirical results, we use a substitution of core variables to examine the impact of the degree of internationalization (a proxy indicator) on enterprise value. The total number of overseas investment subsidiaries of a firm per year is used as a proxy for the internationalization breadth of a company [37]. The logarithm of the average size of a firm’s investment per host country is used to measure the internationalization depth of MNEs. In addition, the Economic Freedom Index (EFI), a global index of economic freedom published by the Heritage Foundation, is selected as a proxy for the institutional environment of the host country. The test results show that the effect of the degree of internationalization (including the breadth and depth of internationalization) on enterprise value after replacing the core variables is still significantly positive, and the effect of the replacement variable of the host country’s institutional environment on enterprise value is also significantly positive. Moreover, the host country’s institutional environment can positively influence the relationship between enterprise internationalization tactics and enterprise value. In summary, the regression results are believed to be robust.

Second, we also examined the impact of different research designs on the empirical results. Based on the specificity of the "Belt and Road" research context, we grouped the sample into state-owned enterprises and private enterprises, taking into account the differences in the strategic objectives of "going global" under the influence of national policies. The sample was divided into state-owned enterprises and private enterprises to test whether the impact of going abroad on enterprise value differs between different ownership groups. The results of the group test show that the hypothesis of this paper still holds, and there is no substantial change in the positive and negative signs of the regression coefficients or the significance of the model.

## 4 Conclusions

Based on OFDI theory, institutional theory and digital technology applications, this paper explores the relationship between internationalization tactics, host country institutional environment, digital transformation and enterprise value using macro data on multinational enterprises’ investments in Belt and Road countries and micro data on enterprises’ overseas investments (2010–2020). The following conclusions are drawn: First, two important dimensions of firms’ internationalization tactics: entry mode and degree of internationalization both significantly affect the realization of enterprise value. The degree of internationalization (including the breadth and depth of internationalization) of overseas investment by MNEs positively affects the realization of enterprise value. Second, The institutional environment of the host country has a moderating effect on the relationship between internationalization tactics and enterprise value. In particular, the better the institutional environment of the host country, the more enterprises tend to choose the sole proprietorship mode to enhance their enterprise value, and the better the institutional environment of the Belt and Road countries, the more it positively moderates the relationship between the degree of internationalization (including the breadth and depth of internationalization) and the enterprise value. Third, digital transformation has a moderating effect on the relationship between an enterprise’s internationalization tactics and its enterprise value. This is reflected in the fact that the higher the degree of digital transformation, the more companies tend to choose the sole proprietorship mode to enhance enterprise value, and the digital transformation of companies has a positive moderating effect on the relationship between the degree of internationalization (including the breadth and depth of internationalization) and enterprise value.

The findings of this paper have implications for the value enhancement of multinational enterprises from emerging economies investing in Belt and Road countries (regions):

From the perspective of enterprises: Firstly, enterprises should be fully aware of the impact of investing in Belt and Road countries (regions) on enterprise value. MNEs often prefer to invest in developed countries in their internationalization tactics. In recent years, due to the prevalence of international trade protectionism, the value benefits gained by enterprises through overseas investments in developed countries have tended to diminish at the margin. Enterprises should be fully aware that the natural resources and cheap labor markets in "Belt and Road" countries (regions) can bring new opportunities for enterprise development. Second, when investing in "Belt and Road" countries (regions), enterprises should pay attention to the important role of the institutional environment of the host country. A sound institutional environment can positively promote the realization of enterprise value, which requires enterprises to invest in countries with a relatively sound institutional environment to avoid operational risks caused by institutional factors such as political instability or legal loopholes. Thirdly, enterprises should use digital transformation as an important means to achieve value addition. In the face of an increasingly competitive international environment, MNEs that have been or are in the process of digital transformation, can use digital technology to develop communication channels with stakeholders such as customers, suppliers and partners in the host country, and enhance information exchange and knowledge sharing. For companies that have not yet implemented digitalization, digital transformation can be used to improve their ability to cope with overseas operational risks. For example, integrate themselves into the digital business ecosystem through the purchase of digital services, and digital acquisitions. Fourth, when enterprises invest in Belt and Road countries (regions), they should choose their entry mode by taking into account the host country’s institutional environment and the degree of digital transformation. When the institutional environment is poor, the joint venture model should be chosen to gain the "legitimacy" of the host country. When the institutional environment is perfect, the sole proprietorship model should be chosen to obtain greater value enhancement. In addition, if the digital transformation of an enterprise is high, the enterprise may choose to enter the host country as a sole proprietor to obtain higher enterprise value. Fifth, enterprises should comprehensively understand the value effect of the degree of internationalization and make reasonable multinational business decisions fully taking into account their actual situation and investment motives. Based on ensuring the geographical diversity of enterprises’ overseas investment, the degree of embeddedness of enterprises in host countries should be further enhanced. Realize the double superposition of the positive effects of internationalization breadth and internationalization depth, and more effectively improve the value of enterprises’ overseas investment.

From the government’s perspective: Firstly, given the large differences in the institutional environment of the Belt and Road countries, the government should update the political and regulatory information of the Belt and Road countries (regions) through regular training or information disclosure. Remind multinational enterprises of the economic obstacles and political risks they may encounter so that they can make timely adjustments to their internationalization strategy decisions by the institutional environment of the host country. Secondly, to motivate enterprises to invest in the Belt and Road countries (regions), the government and relevant departments should also provide more policies and funding favors. Thirdly, the government should give full play to the role of economic, cultural and geographical ties between enterprises and countries, actively build communication platforms for enterprises, and strengthen investment protection mechanisms such as signing or upgrading bilateral trade agreements with countries along the Belt and Road.

## Supporting information

S1 File ROA, Mode, DOI, Digital, WGI, Age, DEBT, data of 1107 multinational enterprises investing in the "Belt and Road" countries. Data from the CSMAR database on OFDI enterprises listed in China and the Ministry of Commerce’s Foreign Investment Enterprises List database to initially identify the "list of multinational enterprises engaged in OFDI and listed in China". These data were used to generate Tables 2–5.
(PDF)
