# Supplementary material for: Internationalization tactics and enterprise value in the context of the "Belt and Road" initiative—Analysis from the perspective of host country institutional environment and enterprise digital transformation
Source: PLoS One. 2023 Aug 18;18(8):e0287324. doi: 10.1371/journal.pone.0287324 (PMC10438032; doi:10.1371/journal.pone.0287324)
Supplement: S1 File — Data from the CSMAR database on OFDI enterprises listed in China and the Ministry of Commerce’s Foreign Investment Enterprises List database to initially identify the "list of multinational enterprises engaged in OFDI and listed in China". These data were used to generate Tables 2–5. (PDF) [file pone.0287324.s001.pdf]

icode	year	DI	ind	JV	ROA	DIV	INT	WGI
9	2017	7	S90	0	1.33	6	19.07	0.45
9	2018	7	S90	0	2.34	6	18.96	0.45
9	2019	7	S90	0	2.37	6	19.21	0.47
10	2017	21	E50	1	4.48	10	17.07	0.2
10	2019	36	E50	1	2.05	13	11.49	0.16
11	2017	21	E50	1	4.48	10	17.07	0.2
11	2019	36	E50	1	2.05	13	11.49	0.16
12	2017	21	E50	1	4.48	10	17.07	0.2
12	2019	36	E50	1	2.05	13	11.49	0.16
13	2013	1	C36	1	5.61	4	16.89	0.1
14	2016	11	F51	1	11.67	7	17.01	0.21
14	2017	5	F51	1	6.92	7	17.37	0.34
14	2018	4	F51	1	8.26	6	17.72	0.55
14	2019	9	F51	1	6.41	9	17.42	0.47
16	2014	1	E48	0	4.49	10	17.23	0.14
16	2015	1	E48	0	5.23	11	15.84	0.14
16	2016	1	E48	0	6.38	11	16.18	0.14
16	2017	1	E48	0	7.29	11	16.15	0.15
16	2018	1	E48	0	6.43	13	16.31	0.18
16	2019	4	E48	0	5.22	13	16.49	0.18
18	2012	1	E48	1	4.64	8	16.63	0.15
18	2013	1	E48	1	4.21	9	16.91	0.14
18	2014	1	E48	1	4.49	10	17.23	0.14
18	2015	1	E48	1	5.23	11	15.84	0.14
18	2016	1	E48	1	6.38	11	16.18	0.14
18	2017	1	E48	1	7.29	11	16.15	0.15
18	2018	1	E48	1	6.43	13	16.31	0.18
18	2019	4	E48	1	5.22	13	16.49	0.18
19	2012	1	E48	1	4.64	8	16.63	0.15
19	2013	1	E48	1	4.21	9	16.91	0.14
19	2014	1	E48	1	4.49	10	17.23	0.14
19	2015	1	E48	1	5.23	11	15.84	0.14
19	2016	1	E48	1	6.38	11	16.18	0.14
19	2017	1	E48	1	7.29	11	16.15	0.15
19	2018	1	E48	1	6.43	13	16.31	0.18
19	2019	4	E48	1	5.22	13	16.49	0.18
20	2012	1	E48	1	4.64	8	16.63	0.15
20	2013	1	E48	1	4.21	9	16.91	0.14
20	2014	1	E48	1	4.49	10	17.23	0.14
20	2015	1	E48	1	5.23	11	15.84	0.14
20	2016	1	E48	1	6.38	11	16.18	0.14
20	2017	1	E48	1	7.29	11	16.15	0.15
20	2018	1	E48	1	6.43	13	16.31	0.18
20	2019	4	E48	1	5.22	13	16.49	0.18
21	2010	2	C39	0	5.89	3	19	0.1
21	2011	7	C39	0	2.59	4	19.09	0.1
21	2013	2	C39	0	1.33	6	18.89	0.13
21	2014	2	C39	0	1.14	8	18.33	0.19
21	2016	2	C39	0	1.72	8	18.39	0.16
21	2017	11	C39	0	4.05	10	18.23	0.17

22	2013	22	C39	1	0.08	6	18.84	0.13
22	2014	9	C39	1	1.14	8	18.33	0.16
22	2015	1	C39	1	1.26	6	18.49	0.15
22	2016	2	C39	1	1.72	8	18.39	0.15
22	2017	11	C39	1	4.05	10	18.23	0.14
22	2018	22	C39	1	3.52	10	18.42	0.2
22	2019	9	C39	1	2.6	10	18.33	0.13
23	2017	11	C39	1	4.05	10	18.23	0.15
23	2018	22	C39	1	3.52	10	18.42	0.18
23	2019	9	C39	1	2.6	10	18.33	0.14
35	2017	9	N77	1	3.18	4	14.03	0.15
35	2018	8	N77	1	2.62	5	17.76	0.2
35	2019	6	N77	1	2.74	26	17.76	0.51
39	2010	1	C33	0	6.23	15	18.45	0.33
39	2011	2	C33	0	6.18	16	18.62	0.32
39	2012	3	C33	0	3.03	15	18.66	0.36
39	2013	4	C33	0	3.89	17	18.66	0.38
39	2014	5	C33	0	3.78	9	17.68	0.33
39	2015	6	C33	0	2.34	10	18.45	0.35
39	2016	7	C33	0	0.64	17	17.66	0.47
39	2017	8	C33	0	2.48	16	17.83	0.53
39	2018	9	C33	0	2.81	13	17.43	0.36
39	2019	10	C33	0	1.52	13	19.84	0.36
41	2015	11	C33	1	2.34	10	18.45	0.35
41	2016	12	C33	1	0.64	17	17.66	0.47
41	2017	13	C33	1	2.48	16	17.83	0.53
45	2010	1	C39	0	3.31	2	16.98	0.15
45	2011	2	C39	0	2.82	2	16.98	0.15
45	2012	3	C39	0	-4.39	2	17	0.15
45	2013	4	C39	0	2	2	17.01	0.15
45	2014	5	C39	0	-4	2	16.16	0.15
45	2015	6	C39	0	0.29	2	16.25	0.15
45	2016	7	C39	0	-2.46	2	16.34	0.15
45	2017	8	C39	0	1.77	2	16.14	0.15
45	2018	9	C39	0	-1.41	2	16.62	0.15
45	2019	10	C39	0	-0.4	2	18.15	0.15
46	2015	1	J69	0	2.5	3	19.75	1.73
46	2016	2	J69	0	2.14	6	20.36	1.15
46	2017	3	J69	0	1.74	5	22.84	2.08
46	2018	4	J69	0	0.52	7	21.09	1.63
46	2019	5	J69	0	0.73	8	21.14	1.5
47	2019	6	J69	1	0.73	8	21.14	1.5
49	2013	6	C38	0	10.51	2	15.45	0.15
49	2014	5	C38	0	7.78	3	15.77	0.13
49	2015	6	C38	0	7.63	3	16.4	0.13
50	2010	1	C39	1	1.95	4	17.61	0.1
50	2011	2	C39	1	3.21	4	17.43	0.1
50	2012	3	C39	1	1.74	4	17.44	0.1
50	2013	4	C39	1	3.62	4	17.54	0.1
50	2014	5	C39	1	5.8	5	17.92	0.12
50	2015	6	C39	1	2.98	5	17.91	0.1

50	2016	7	C39	1	2.63	5	18.01	0.1
50	2017	8	C39	1	3.15	5	17.95	0.1
50	2018	9	C39	1	2.19	5	15.68	0.1
50	2019	10	C39	1	1.32	6	15.51	0.1
51	2014	11	C39	1	5.8	5	17.92	0.12
51	2015	12	C39	1	2.98	5	17.91	0.1
51	2016	13	C39	1	2.63	5	18.01	0.1
51	2017	14	C39	1	3.15	5	17.95	0.1
51	2018	15	C39	1	2.19	5	15.68	0.1
51	2019	16	C39	1	1.32	6	15.51	0.1
52	2019	17	C39	0	1.32	6	15.51	0.1
55	2019	1	C33	1	3.15	4	16.48	0.1
60	2019	1	C32	1	4.38	5	18.62	0.16
63	2010	16	C39	0	4.56	4	19.25	0.13
63	2011	30	C39	0	2.37	4	18.67	0.1
63	2012	35	C39	0	-2.45	4	18.67	0.1
63	2013	29	C39	0	1.38	9	18.67	0.11
63	2014	27	C39	0	2.64	10	19.65	0.11
63	2015	51	C39	0	3.29	5	18.54	0.12
63	2016	60	C39	0	-1.07	6	18.52	0.12
63	2017	38	C39	0	3.77	8	17.02	0.13
63	2018	50	C39	0	-5.09	8	19.75	0.13
63	2019	43	C39	0	4.27	18	20.68	0.12
64	2010	16	C39	1	4.56	4	19.25	0.13
64	2013	29	C39	1	1.38	9	17.79	0.11
64	2014	27	C39	1	2.64	10	17.79	0.11
64	2019	43	C39	1	4.27	18	19.68	0.12
65	2010	1	E48	0	2.32	1	18.59	0.1
65	2011	1	E48	0	3.98	1	18.19	0.1
65	2012	1	E48	0	5.19	1	19.25	0.1
65	2013	1	E48	0	5.46	4	17.21	0.13
65	2017	1	E48	0	4.6	9	16.25	0.11
66	2018	1	E48	1	5.39	10	16.1	0.11
66	2019	1	E48	1	5.65	10	16.23	0.12
67	2013	1	E48	0	5.46	4	17.21	0.13
67	2014	1	E48	0	5.04	3	17.13	0.13
67	2015	1	E48	0	4	4	14.91	0.13
67	2016	1	E48	0	5.63	7	16.33	0.11
67	2017	1	E48	0	4.6	9	16.25	0.11
67	2018	1	E48	0	5.39	10	16.1	0.11
67	2019	1	E48	0	5.65	10	16.23	0.12
68	2013	1	E48	1	5.46	4	17.21	0.13
68	2014	1	E48	1	5.04	3	17.13	0.13
68	2015	1	E48	1	4	4	14.91	0.13
68	2016	1	E48	1	5.63	7	16.33	0.11
68	2017	1	E48	1	4.6	9	16.25	0.11
68	2018	1	E48	1	5.39	10	16.1	0.11
68	2019	1	E48	1	5.65	10	16.23	0.12
69	2016	1	E48	0	5.63	7	16.33	0.11
69	2017	1	E48	0	4.6	9	16.25	0.11
69	2018	1	E48	0	5.39	10	16.1	0.11

69	2019	1	E48	0	5.65	10	16.23	0.12
70	2017	1	E48	1	4.6	9	16.25	0.11
70	2018	1	E48	1	5.39	10	16.1	0.11
70	2019	1	E48	1	5.65	10	16.23	0.12
71	2018	1	E48	0	5.39	10	16.1	0.11
71	2019	1	E48	0	5.65	10	16.23	0.12
100	2010	1	C39	1	1.13	30	17.53	0.3
100	2011	2	C39	1	2.62	29	17.53	0.34
100	2012	3	C39	1	1.66	29	16.03	0.35
100	2013	4	C39	1	4.95	28	16.02	0.43
100	2014	5	C39	1	3.16	31	15.36	0.43
100	2015	6	C39	1	1.65	13	15.06	0.26
100	2016	7	C39	1	2.31	13	15.89	0.22
100	2017	8	C39	1	2.3	14	15.89	0.3
100	2018	9	C39	1	2.05	14	15.89	0.33
157	2011	1	C35	1	12.15	5	19.95	0.2
157	2012	2	C35	1	9.38	5	19.01	0.18
157	2013	3	C35	1	4.43	8	18.56	0.19
157	2014	4	C35	1	0.69	9	17.05	0.14
157	2015	5	C35	1	0.1	9	16.96	0.2
157	2016	6	C35	1	-0.99	9	18.02	0.18
157	2017	7	C35	1	1.45	9	18.02	0.2
157	2018	8	C35	1	2.22	7	18.02	0.16
158	2013	9	C35	0	4.43	8	17.56	0.19
158	2014	10	C35	0	0.69	9	17.95	0.14
158	2015	11	C35	0	0.1	9	17.95	0.2
158	2016	12	C35	0	-0.99	9	17.95	0.18
158	2017	13	C35	0	1.45	9	18.66	0.2
158	2019	14	C35	0	4.61	10	18.66	0.14
159	2012	1	F51	1	3.29	3	18.6	0.1
159	2013	2	F51	1	1.18	4	18.6	0.1
159	2014	3	F51	0	4.46	4	18.96	0.1
159	2015	4	F51	0	2.03	3	18.38	0.1
159	2016	5	F51	0	1.18	3	18.36	0.1
159	2017	6	F51	0	-2.31	3	18.51	0.1
159	2018	7	F51	0	1.73	3	19.58	0.1
159	2019	8	F51	0	0.94	2	19.88	0.1
160	2014	9	F51	1	4.46	4	17.96	0.1
161	2015	10	F51	1	2.03	3	18.33	0.1
161	2016	11	F51	1	1.18	3	18.36	0.1
161	2017	12	F51	1	-2.31	3	18.51	0.1
161	2018	13	F51	1	1.73	3	18.58	0.1
333	2013	8	C38	0	8.99	3	20.19	0.37
333	2014	32	C38	0	10.72	3	20.67	0.21
334	2013	8	C38	0	8.99	3	20.63	0.21
334	2014	32	C38	0	10.72	3	20.67	0.21
335	2013	8	C38	0	8.99	3	21.63	0.21
335	2014	32	C38	0	10.72	3	20.74	0.21
336	2016	9	C38	0	10.59	1	22.52	0.22
336	2017	39	C38	0	8.89	4	21.69	0.22
336	2018	42	C38	0	8.46	3	20.72	0.21

336	2019	42	C38	0	8.94	3	20.75	0.21
338	2012	1	C36	1	5.06	11	19.93	0.14
338	2013	1	C36	1	5.27	12	20.29	0.17
339	2012	1	C36	1	5.06	11	19.93	0.14
339	2013	1	C36	1	5.27	12	20.29	0.17
339	2014	2	C36	1	5.82	5	19.5	0.1
339	2015	2	C36	1	1.84	5	19.48	0.1
339	2016	2	C36	1	2.58	6	19.37	0.1
339	2017	4	C36	1	5.19	6	19.9	0.12
339	2018	2	C36	1	5.89	8	20.14	0.11
339	2019	3	C36	1	5.39	6	20.48	0.15
340	2013	1	C36	1	5.27	12	20.29	0.17
415	2015	1	L71	0	1.79	5	19.29	0.12
415	2016	2	L71	0	1.63	6	19.98	0.15
425	2012	1	C35	1	6.16	3	18.07	0.17
425	2013	1	C35	1	3.28	5	17.56	0.14
425	2014	1	C35	1	0.83	6	17.85	0.13
425	2015	1	C35	1	-0.14	6	18.74	0.12
425	2016	2	C35	1	0.5	7	18.61	0.11
425	2017	10	C35	1	2.19	7	18.74	0.11
425	2018	30	C35	1	3.7	10	19.27	0.11
425	2019	40	C35	1	5.26	12	19.13	0.12
426	2013	1	C35	1	3.28	5	17.56	0.14
426	2014	1	C35	1	0.83	6	17.85	0.13
426	2015	1	C35	1	-0.14	6	18.74	0.12
426	2016	2	C35	1	0.5	7	18.61	0.11
426	2017	10	C35	1	2.19	7	18.74	0.11
426	2018	30	C35	1	3.7	10	19.27	0.11
426	2019	40	C35	1	5.26	12	19.13	0.12
427	2018	30	C35	1	3.7	10	19.27	0.11
427	2019	40	C35	1	5.26	12	19.13	0.12
428	2018	30	C35	1	3.7	10	19.27	0.11
428	2019	40	C35	1	5.26	12	19.13	0.12
429	2018	30	C35	1	3.7	10	19.27	0.11
429	2019	40	C35	1	5.26	12	19.13	0.12
488	2012	4	C22	1	0.1	3	18.59	0.1
488	2013	5	C22	1	1.45	4	17.18	0.1
488	2014	6	C22	1	0.87	4	16.02	0.1
488	2015	7	C22	1	3.12	5	16.46	0.1
488	2016	8	C22	1	2.52	5	16.41	0.1
488	2017	9	C22	1	4	5	18.18	0.1
488	2018	10	C22	1	2.43	5	18.39	0.12
488	2019	11	C22	1	1.73	5	20.4	0.12
489	2015	7	C22	1	3.12	5	16.46	0.1
489	2016	8	C22	1	2.52	5	16.41	0.1
489	2017	9	C22	1	4	5	18.18	0.1
489	2018	10	C22	1	2.43	5	18.39	0.12
489	2019	11	C22	1	1.73	5	20.4	0.12
521	2011	2	C38	0	1.21	1	17.42	0.2
521	2012	3	C38	0	2.08	1	17.38	0.2
521	2013	11	C38	0	3.37	1	17.2	0.2

521	2014	6	C38	0	3.2	1	16.47	0.2
521	2015	9	C38	0	0.23	1	18.12	0.2
521	2016	14	C38	0	2.04	2	17.52	0.15
521	2017	16	C38	0	0.25	2	17.63	0.15
521	2018	12	C38	0	0.22	1	17.94	0.2
521	2019	10	C38	0	0.29	2	17.61	0.15
523	2016	14	C38	1	2.04	2	17.52	0.15
523	2017	16	C38	1	0.25	2	17.63	0.15
523	2019	10	C38	1	0.29	2	17.61	0.15
528	2010	1	C35	1	11	7	16.92	0.11
528	2011	2	C35	1	6.41	10	17.1	0.11
528	2012	3	C35	1	1.23	11	17.52	0.11
528	2013	4	C35	1	1.48	10	17.63	0.11
528	2014	5	C35	1	0.9	10	17.5	0.13
528	2015	6	C35	1	0.1	11	17.3	0.12
528	2016	7	C35	1	0.23	11	17.5	0.12
528	2017	8	C35	1	1.53	13	17.33	0.12
528	2018	9	C35	1	3.51	13	17.5	0.12
528	2019	10	C35	1	3.83	14	17.56	0.13
529	2012	11	C35	0	1.23	11	17.52	0.11
529	2013	12	C35	0	1.48	10	17.63	0.11
529	2014	13	C35	0	0.9	10	17.5	0.13
529	2015	14	C35	0	0.1	11	17.3	0.12
529	2016	15	C35	0	0.23	11	17.5	0.12
529	2017	16	C35	0	1.53	13	17.33	0.12
529	2018	17	C35	0	3.51	13	17.5	0.12
529	2019	18	C35	0	3.83	14	17.56	0.13
530	2017	19	C35	1	1.53	13	17.33	0.12
530	2018	20	C35	1	3.51	13	17.5	0.12
530	2019	21	C35	1	3.83	14	17.56	0.13
531	2019	22	C35	1	3.83	14	17.56	0.13
547	2018	10	C39	1	5.99	1	20.5	0.1
547	2019	19	C39	1	7.16	3	19.69	0.13
551	2019	1	S90	1	4.36	9	15.49	0.12
552	2019	1	S90	0	4.36	9	15.49	0.12
553	2017	1	C26	1	7.26	9	16.25	0.13
553	2018	1	C26	1	5.83	11	16.1	0.15
553	2019	1	C26	1	0.63	9	16.51	0.17
554	2017	1	C26	1	7.26	9	16.25	0.13
554	2018	1	C26	1	5.83	11	16.1	0.15
554	2019	1	C26	1	0.63	9	16.51	0.17
555	2013	28	I65	0	8.01	3	20.61	0.17
555	2019	108	I65	1	3.56	3	19.88	0.13
586	2010	2	C39	0	11.49	2	16.99	0.1
586	2011	1	C39	0	4.31	2	17.1	0.1
586	2012	1	C39	0	1.66	2	17.22	0.1
586	2013	1	C39	0	3.37	2	17.22	0.1
586	2014	4	C39	0	1.74	1	17.05	0.1
586	2015	3	C39	0	3.33	1	16.99	0.1
586	2016	4	C39	0	0.7	1	17.04	0.1
586	2017	6	C39	0	0.17	1	17.09	0.1

586	2018	13	C39	0	-0.32	1	17.05	0.1
598	2017	1	D46	0	5.14	1	12.62	0.1
599	2016	8	C29	0	1.18	2	14.23	0.2
599	2017	6	C29	0	1.3	2	16.92	0.2
599	2018	5	C29	0	0.33	2	16.92	0.2
599	2019	2	C29	0	-3.31	2	16.92	0.2
607	2011	1	R85	0	2.48	4	15	0.13
607	2012	2	R85	0	2.64	4	15.11	0.13
607	2013	3	R85	0	1.78	4	14.82	0.13
608	2011	4	R85	0	2.48	4	15	0.13
608	2012	5	R85	0	2.64	4	15.11	0.13
608	2013	6	R85	0	1.78	4	14.82	0.13
609	2011	7	R85	0	2.48	4	15	0.13
609	2012	8	R85	0	2.64	4	15.11	0.13
609	2013	9	R85	0	1.78	4	14.82	0.13
610	2012	10	R85	0	2.64	4	15.11	0.13
610	2013	11	R85	0	1.78	4	14.82	0.13
618	2018	1	C29	1	0.09	2	16.59	0.15
618	2019	2	C29	1	0.28	2	17.41	0.15
619	2018	3	C29	0	0.09	2	16.59	0.15
619	2019	4	C29	0	0.28	2	17.41	0.15
620	2014	1	K70	0	1.88	3	19.43	0.1
620	2015	2	K70	0	0.98	3	19.43	0.17
620	2017	3	K70	0	1.8	5	19.43	0.12
625	2011	1	C36	1	2.76	4	21.34	0.1
625	2012	1	C36	1	3.42	4	21.36	0.1
625	2013	1	C36	1	6.97	4	21.86	0.1
625	2014	1	C36	1	12.22	6	21.47	0.1
625	2015	4	C36	1	12.47	6	21.63	0.1
625	2016	1	C36	1	10.49	6	21.62	0.1
625	2017	10	C36	1	6.78	6	21.58	0.1
625	2018	8	C36	1	0.72	6	21.52	0.1
625	2019	7	C36	1	-2.77	7	21.18	0.1
626	2014	1	C36	1	12.22	6	21.47	0.1
626	2015	4	C36	1	12.47	6	21.63	0.1
626	2016	1	C36	1	10.49	6	21.62	0.1
626	2017	10	C36	1	6.78	6	21.58	0.1
626	2018	8	C36	1	0.72	6	21.52	0.1
626	2019	7	C36	1	-2.77	7	21.18	0.1
627	2019	7	C36	1	-2.77	7	21.18	0.1
651	2010	1	C38	0	7.35	3	15.8	0.13
651	2011	2	C38	0	7.02	4	15.26	0.13
651	2012	3	C38	0	7.72	4	15.17	0.13
651	2013	4	C38	0	9.07	4	15.17	0.13
651	2014	29	C38	0	9.83	4	15.26	0.13
651	2016	11	C38	0	7.94	4	15.8	0.15
651	2017	10	C38	0	9.02	4	15.87	0.15
651	2018	30	C38	0	11.33	4	16.13	0.15
651	2019	40	C38	0	11.32	4	16.13	0.15
659	2010	1	C29	0	9.3	3	21.58	0.13
659	2011	2	C29	0	1.23	3	21.38	0.13

659	2012	3	C29	0	-3.42	3	21.58	0.13
659	2013	4	C29	0	-22.61	3	21.68	0.13
659	2014	5	C29	0	0.91	3	21.38	0.13
659	2015	6	C29	0	-2.08	3	21.97	0.13
659	2016	7	C29	0	-20.31	3	21.47	0.13
660	2010	8	C29	1	3.7	3	21.58	0.13
660	2011	9	C29	1	1.23	3	21.38	0.13
660	2012	10	C29	1	-3.42	3	21.58	0.13
660	2013	11	C29	1	-22.61	3	21.68	0.13
660	2014	12	C29	1	0.91	3	21.38	0.13
660	2015	13	C29	1	-2.08	3	21.97	0.13
660	2016	14	C29	1	-20.31	3	21.47	0.13
666	2013	1	J67	0	10.35	4	17.91	0.18
666	2014		J67	0	9.97	6	17.85	0.17
666	2015	6	J67	0	8.04	7	19.47	0.29
666	2016	2	J67	0	6.95	7	19.9	0.37
666	2019	3	J67	1	4.31	3	20.88	0.1
672	2015	3	C30	1	1.41	1	17.32	0.1
672	2016	4	C30	1	2.65	1	19.06	0.1
672	2017	5	C30	1	13.01	1	19.06	0.4
673	2017	6	C30	0	13.01	1	17.32	0.4
673	2018	7	C30	0	22.89	1	19.06	0.1
673	2019	8	C30	0	28.14	1	19.06	0.4
676	2016	68	I64	1	9.3	5	12.57	0.2
676	2017	70	I64	1	7.82	5	16.74	0.26
676	2018	58	I64	1	9.57	5	13.57	0.24
676	2019	87	I64	1	7.08	5	17.68	0.48
678	2013	1	C34	0	0.24	4	12.27	0.15
680	2011	1	C35	1	4.5	7	18.25	0.1
680	2012	1	C35	1	0.17	9	18.07	0.11
680	2013	1	C35	1	-3.01	9	18.18	0.11
680	2014	1	C35	1	-0.23	9	17.89	0.11
680	2015	1	C35	1	-9.4	9	17.91	0.11
680	2016	1	C35	1	0.19	10	17.97	0.11
680	2017	1	C35	1	0.66	10	18.42	0.12
680	2018	2	C35	1	0.9	10	18.45	0.12
680	2019	3	C35	1	0.68	10	18.52	0.12
682	2011	1	I65	1	2.18	1	17.55	0.1
682	2012	3	I65	1	2.75	1	17.51	0.1
682	2013	6	I65	1	3.54	1	17.16	0.1
682	2014	6	I65	1	3.76	1	15.17	0.1
682	2015	11	I65	1	3.56	1	15.34	0.1
682	2016	14	I65	1	4.44	1	15.44	0.1
682	2017	15	I65	1	4.54	1	16.4	0.1
682	2018	10	I65	1	4.49	1	16.61	0.1
682	2019	35	I65	1	4.95	1	17.69	0.1
703	2014	1	C28	1	-1.57	4	20.29	0.13
703	2015	1	C28	1	0.62	4	20.96	0.13
710	2018	8	M74	1	13.04	3	17.07	0.2
710	2019	5	M74	1	14.76	3	17.92	0.2
718	2015	1	K70	1	3.82	2	19.24	0.1

718	2016	2	K70	1	4.61	2	19.24	0.2
718	2017	3	K70	1	5.23	3	19.24	0.1
719	2016	4	K70	0	4.61	2	19.24	0.2
719	2018	5	K70	0	5.07	3	19.24	0.1
719	2019	6	K70	0	6.56	3	19.24	0.1
724	2019	1	C39	0	-0.15	4	20.34	0.1
725	2017	2	C39	1	3.41	2	21.97	0.1
725	2018	3	C39	1	1.03	3	20.5	0.1
725	2019	4	C39	0	-0.15	4	20.34	0.1
726	2017	4	C17	1	9.02	5	16.79	0.12
726	2018	7	C17	1	8.29	5	16.79	0.12
726	2019	2	C17	1	8.82	5	16.79	0.14
758	2010	1	B09	1	3.31	6	18.42	0.17
758	2011	2	B09	1	4.59	6	19.14	0.15
758	2012	3	B09	1	1.63	5	19.13	0.18
758	2013	4	B09	1	1.07	8	18.68	0.14
758	2014	5	B09	1	1.77	10	17.79	0.15
758	2015	6	B09	1	1.46	13	17.53	0.14
758	2016	7	B09	1	1.47	13	17.73	0.14
758	2017	8	B09	1	1.6	6	18.36	0.22
758	2018	9	B09	1	1.04	15	17.52	0.14
758	2019	10	B09	1	-4.6	15	18.15	0.17
759	2010	11	B09	0	3.31	6	18.42	0.17
759	2011	12	B09	0	4.59	6	19.14	0.15
759	2012	13	B09	0	1.63	5	19.13	0.18
759	2013	14	B09	0	1.07	8	18.68	0.14
759	2014	15	B09	0	1.77	10	17.79	0.15
759	2015	16	B09	0	1.46	13	17.53	0.14
759	2016	17	B09	0	1.47	13	17.73	0.14
759	2017	18	B09	0	1.6	6	18.36	0.22
759	2018	19	B09	0	1.04	15	17.52	0.14
759	2019	20	B09	0	-4.6	15	18.15	0.17
760	2013	21	B09	1	1.07	8	18.68	0.14
760	2014	22	B09	1	1.77	10	17.79	0.15
760	2015	23	B09	1	1.46	13	17.53	0.14
760	2016	24	B09	1	1.47	13	17.73	0.14
760	2017	25	B09	1	1.6	6	18.36	0.22
760	2018	26	B09	1	1.04	15	17.52	0.14
760	2019	27	B09	1	-4.6	15	18.15	0.17
761	2013	28	B09	1	1.07	8	18.68	0.14
761	2014	29	B09	1	1.77	10	17.79	0.15
761	2015	30	B09	1	1.46	13	17.53	0.14
761	2016	31	B09	1	1.47	13	17.73	0.14
761	2017	32	B09	1	1.6	6	18.36	0.22
761	2018	33	B09	1	1.04	15	17.52	0.14
761	2019	34	B09	1	-4.6	15	18.15	0.17
762	2014	35	B09	1	1.77	10	17.79	0.15
762	2015	36	B09	1	1.46	13	17.53	0.14
762	2016	37	B09	1	1.47	13	17.73	0.14
762	2017	38	B09	1	1.6	6	18.36	0.22
762	2018	39	B09	1	1.04	15	17.52	0.14

762	2019	40	B09	1	-4.6	15	18.15	0.17
763	2014	41	B09	1	1.77	10	17.79	0.15
763	2015	42	B09	1	1.46	13	17.53	0.14
763	2016	43	B09	1	1.47	13	17.73	0.14
763	2017	44	B09	1	1.6	6	18.36	0.22
763	2018	45	B09	1	1.04	15	17.52	0.14
763	2019	46	B09	1	-4.6	15	18.15	0.17
764	2018	47	B09	1	1.04	15	17.52	0.14
764	2019	48	B09	1	-4.6	15	18.15	0.17
778	2015	1	C33	0	0.9	3	21.31	0.17
778	2017	1	C33	0	2.17	4	20.78	0.15
778	2018	1	C33	0	4.03	4	20.63	0.18
786	2019	1	C30	1	2.32	2	18.33	0.1
796	2019	1	L72	1	2.41	5	18.91	0.16
797	2018	80	C39	1	3.97	9	14.17	0.12
797	2019	63	C39	1	6.53	8	16.49	0.13
810	2016	30	C39	1	8.9	5	14.17	0.1
810	2017	51	C39	1	1.35	7	14.01	0.11
810	2018	80	C39	1	3.97	9	14.17	0.12
810	2019	63	C39	1	6.53	8	13.17	0.13
811	2011	1	C34	1	7.86	1	19.75	0.1
811	2012	2	C34	1	6.3	1	19.87	0.1
811	2013	3	C34	1	10.62	1	19.98	0.1
811	2014	4	C34	1	8.32	1	20	0.1
811	2015	5	C34	1	8.94	10	17.29	0.14
811	2016	6	C34	1	6.61	12	17.47	0.13
811	2017	7	C34	1	6.11	12	16.88	0.13
811	2018	8	C34	1	5.2	12	16.93	0.13
811	2019	9	C34	1	6.95	12	17	0.13
812	2015	10	C34	1	8.94	10	17.29	0.14
812	2016	11	C34	1	6.61	12	17.47	0.13
812	2017	12	C34	1	6.11	12	16.88	0.13
812	2018	13	C34	1	5.2	12	16.93	0.13
812	2019	14	C34	1	6.95	12	17	0.13
816	2011	1	C34	1	1.82	2	16.34	0.1
816	2012	2	C34	1	1.91	2	16.12	0.1
816	2013	3	C34	1	1.51	2	16.12	0.1
816	2014	4	C34	1	0.68	2	16.34	0.1
816	2015	5	C34	1	0.06	2	17.18	0.1
816	2016	6	C34	1	-2.51	2	17.18	0.1
816	2017	7	C34	1	-4.75	2	17.83	0.1
816	2018	8	C34	1	0.04	2	18.83	0.1
821	2012	1	C35	1	-5.44	2	17.1	0.1
821	2013	1	C35	1	0.69	2	17.3	0.1
821	2014	1	C35	1	1.15	2	17.3	0.1
821	2015	1	C35	1	2.78	2	18.17	0.1
821	2016	20	C35	1	3.42	2	18.39	0.1
821	2017	33	C35	1	5.35	3	18.28	0.1
821	2018	32	C35	1	3.77	3	18.09	0.1
821	2019	10	C35	1	-10.32	3	18.39	0.1
822	2017	33	C35	1	5.35	3	18.08	0.1

822	2018	32	C35	1	3.77	3	18.08	0.1
825	2017	2	C31	1	5.94	4	19.67	0.1
825	2018	3	C31	1	6.71	4	19.75	0.1
825	2019	4	C31	1	2.92	4	19.82	0.1
826	2018	1	N77	1	1.88	3	19.49	0.1
826	2019	2	N77	1	1.01	3	19.72	0.13
876	2011	1	C13	1	21.28	7	20.75	0.2
876	2012	2	C13	1	9.46	8	20.73	0.2
876	2013	3	C13	1	9.19	5	20.75	0.22
878	2011	4	C13	0	21.28	7	20.75	0.2
878	2012	5	C13	0	9.46	8	20.83	0.2
887	2016	1	C29	1	9.61	9	15.85	0.37
887	2017	2	C29	1	8.48	17	15.85	0.28
887	2018	3	C29	1	7.24	19	15.85	0.27
887	2019	4	C29	1	3.46	20	15.46	0.27
890	2015	5	C29	0	11.63	6	16.07	0.32
890	2016	6	C29	0	9.61	9	16.07	0.37
890	2017	7	C29	0	8.48	17	16.07	0.28
890	2018	8	C29	0	7.24	19	16.07	0.27
890	2019	9	C29	0	3.46	20	16.46	0.27
910	2014	1	C20	0	3.23	5	16.44	0.16
910	2015	7	C20	0	5.33	5	16.44	0.16
910	2016	3	C20	0	8.61	5	16.44	0.14
910	2017	3	C20	0	12.02	5	16.44	0.14
910	2018	4	C20	0	11.52	6	16.44	0.13
910	2019	2	C20	0	9.87	5	16.44	0.14
913	2012	1	C37	0	-0.13	2	19.04	0.1
919	2011	1	C27	0	7.39	1	18.1	0.1
919	2012	1	C27	0	5.82	1	18.1	0.1
919	2013	1	C27	0	5.85	1	18.2	0.1
919	2014	1	C27	0	7.08	1	17.41	0.1
919	2015	1	C27	0	7.01	1	17.63	0.1
919	2016	1	C27	0	5.77	1	17.71	0.1
919	2017	2	C27	0	4.48	1	17.82	0.1
919	2018	3	C27	0	7.46	1	18.13	0.1
919	2019	4	C27	0	4.57	1	19.38	0.1
921	2011	1	C38	1	2.88	4	18.84	0.13
921	2012	2	C38	1	8.7	3	19.34	0.13
921	2013	3	C38	1	11.99	4	19.33	0.13
921	2014	4	C38	1	5.64	4	19.53	0.13
921	2015	5	C38	1	3.95	3	19.9	0.13
921	2016	6	C38	1	6.85	3	20.11	0.13
927	2017	1	E48	1	3.14	6	17.56	0.1
927	2018	1	E48	1	2.93	6	17.76	0.12
927	2019	1	E48	1	3.17	7	17.67	0.11
928	2016	1	E48	1	3.82	5	17.39	0.1
928	2017	1	E48	1	3.14	6	17.56	0.1
928	2018	1	E48	1	2.93	6	17.76	0.12
928	2019	1	E48	1	3.17	7	17.67	0.11
929	2016	1	E48	1	3.82	5	17.39	0.1
929	2017	1	E48	1	3.14	6	17.56	0.1

929	2018	1	E48	1	2.93	6	17.76	0.12
929	2019	1	E48	1	3.17	7	17.67	0.11
930	2012	1	C26	0	3.02	4	16.1	0.15
930	2013	2	C26	0	0.75	4	16.1	0.15
930	2018	3	C26	0	3.94	6	18.32	0.52
930	2019	4	C26	0	2.99	5	18.37	0.6
931	2012	5	C26	1	3.02	4	16.1	0.15
931	2013	6	C26	1	0.75	4	16.1	0.15
931	2018	7	C26	1	3.94	6	18.32	0.52
931	2019	8	C26	1	2.99	5	18.37	0.6
938	2019	141	C39	1	5.95	8	16.57	0.16
939	2019	141	C39	1	5.95	8	16.57	0.16
940	2019	141	C39	1	5.95	8	16.57	0.16
941	2016	73	C39	1	5.5	3	17.91	0.2
941	2017	79	C39	1	6.6	3	17.74	0.27
941	2018	85	C39	1	6.44	3	17.46	0.27
941	2019	141	C39	1	5.95	8	16.57	0.16
942	2019	141	C39	1	5.95	8	16.57	0.16
943	2016	73	C39	1	5.5	3	17.91	0.2
943	2017	79	C39	1	6.6	3	17.74	0.27
943	2018	85	C39	1	6.44	3	17.46	0.27
943	2019	141	C39	1	5.95	8	16.57	0.16
963	2018	1	F52	1	13.61	11	15.91	0.24
963	2019	2	F52	0	14.38	12	15.91	0.23
964	2019	1	F52	1	14.38	12	16.74	0.23
977	2018	75	C39	1	3	3	18.47	0.2
977	2019	80	C39	1	3.47	5	17.96	0.16
998	2011	2	A01	1	8.4	2	18.47	0.15
998	2012	1	A01	1	8.59	2	18.22	0.15
998	2013	2	A01	1	8.17	2	18.81	0.15
998	2017	3	A01	1	8.53	6	20	0.13
999	2017	4	A01	1	8.53	6	20	0.13
999	2018	2	A01	1	6.37	5	20.22	0.2
1000	2017	5	A01	0	8.53	6	20	0.13
1000	2018	1	A01	0	6.37	5	20.22	0.2
1000	2019	1	A01	0	-1.17	2	21.05	0.2
1696	2010	2	C37	1	12.32	1	16.42	0.1
1696	2011	3	C37	1	9.95	1	16.35	0.1
1696	2012	4	C37	1	8.1	1	16.32	0.1
1696	2013	5	C37	1	6.73	1	16.32	0.1
1696	2014	6	C37	1	7.97	1	16.35	0.1
1696	2015	7	C37	1	7.21	1	16.42	0.1
1696	2016	8	C37	1	5.56	2	16.42	0.1
1696	2017	9	C37	1	5.09	2	18.11	0.1
1696	2018	10	C37	1	5.47	3	18.12	0.17
1696	2019	11	C37	1	5.56	3	18.12	0.17
1872	2018	1	G55	1	2.43	9	22.44	0.2
1872	2019	2	G55	1	5.74	7	22.84	0.19
1979	2018	3	K70	0	5.15	5	21.97	1.86
1979	2019	4	K70	1	3.62	5	22.39	1.64
2002	2017	32	C26	1	7.48	2	18.79	0.1

2002	2018	26	C26	1	4.3	2	18.79	0.1
2002	2019	29	C26	1	4.07	2	18.79	0.1
2003	2016	1	C18	0	12.99	2	16.21	0.15
2003	2017	1	C18	0	14.33	2	16.32	0.15
2003	2018	2	C18	0	10.54	2	16.26	0.15
2003	2019	2	C18	1	8.52	4	16.34	0.13
2006	2013	1	C35	0	8.72	7	17.58	0.19
2006	2014	2	C35	0	10.36	7	17.58	0.19
2006	2015	1	C35	0	10.1	7	17.58	0.2
2006	2016	1	C35	0	8.4	8	17.58	0.29
2006	2017	3	C35	0	13.98	9	17.58	0.23
2006	2018	4	C35	0	10.44	10	17.58	0.26
2007	2012	1	C35	1	10.09	6	17.72	0.18
2007	2013	1	C35	1	8.72	7	17.58	0.19
2007	2014	2	C35	1	10.36	7	17.71	0.19
2007	2015	1	C35	1	10.1	7	17.71	0.2
2007	2016	1	C35	1	8.4	8	17.71	0.29
2008	2017	3	C35	1	13.98	9	18.25	0.23
2008	2018	4	C35	1	10.44	10	18.25	0.26
2009	2019	1	C34	1	0.99	3	18.53	0.1
2011	2015	1	C34	1	0.65	3	16.8	0.2
2011	2016	2	C34	1	0.73	4	16.8	0.15
2011	2017	1	C34	1	0.64	4	16.01	0.18
2011	2018	1	C34	1	-19.11	5	15.74	0.16
2011	2019	1	C34	1	0.24	5	15.74	0.16
2013	2018	1	C37	1	3.71	1	18.27	0.1
2013	2019	1	C37	1	3.84	1	19.29	0.1
2015	2019	1	D44	1	6.77	6	19.18	0.22
2017	2011	2	C39	0	2.99	4	8.21	0.13
2017	2013	3	C39	0	2.73	4	15.83	0.1
2017	2014	4	C39	0	3.92	4	15.7	0.1
2017	2015	5	C39	0	4.49	4	15.57	0.1
2017	2016	6	C39	0	5.56	4	15.46	0.1
2017	2017	7	C39	0	2.38	4	16.6	0.1
2017	2018	8	C39	0	2.43	4	16.56	0.1
2017	2019	9	C39	0	2.12	4	16.36	0.1
2018	2011	2	C39	1	2.99	4	8.21	0.13
2018	2013	3	C39	1	2.73	4	15.83	0.1
2018	2014	4	C39	1	3.92	4	15.7	0.1
2018	2015	5	C39	1	4.49	4	15.57	0.1
2018	2016	6	C39	1	5.56	4	15.46	0.1
2018	2017	7	C39	1	2.38	4	16.6	0.1
2018	2018	8	C39	1	2.43	4	16.56	0.1
2018	2019	9	C39	1	2.12	4	16.36	0.1
2019	2018	1	C27	1	7.48	7	15.65	0.2
2019	2019	1	C27	1	8.38	9	15.65	0.19
2020	2017	2	C27	0	6.52	4	18.4	0.1
2020	2018	3	C27	0	7.51	4	18.4	0.13
2021	2011	1	C39	0	2.99	4	8.21	0.13
2021	2013	2	C39	0	2.73	4	15.83	0.1
2021	2014	3	C39	0	3.92	4	15.7	0.1

2021	2015	4 C39	0	4.49	4	15.57	0.1
2021	2016	5 C39	0	5.56	4	15.46	0.1
2021	2017	6 C39	0	2.38	4	16.6	0.1
2021	2018	7 C39	0	2.43	4	16.56	0.1
2021	2019	8 C39	0	2.12	4	16.36	0.1
2022	2019	1 C27	0	8.38	9	18.18	0.19
2023	2019	1 C27	0	9.83	3	17.73	0.17
2024	2011	33 F52	0	9.42	1	20.13	0.2
2024	2012	32 F52	0	3.69	2	19.48	0.1
2024	2013	30 F52	0	0.13	4	20.08	0.15
2024	2014	14 F52	0	1	3	20.13	0.17
2024	2015	21 F52	0	0.89	3	20.13	0.17
2024	2016	49 F52	0	0.44	3	20.13	0.17
2024	2017	53 F52	0	2.75	3	19.58	0.13
2024	2018	114 F52	0	7.09	4	19.48	0.15
2024	2019	40 F52	0	4.27	4	19.48	0.25
2026	2019	1 C33	1	-4.06	1	17.33	0.1
2027	2017	2 L72	1	43.15	3	19.31	0.7
2027	2018	3 L72	1	33.5	4	19.31	0.68
2027	2019	4 L72	1	9.84	5	19.31	0.56
2028	2012	13 C38	1	7.1	2	17.04	0.1
2028	2013	8 C38	1	8.7	2	17.09	0.1
2028	2014	4 C38	1	9.66	4	17.09	0.1
2028	2015	3 C38	1	7.37	4	17.54	0.1
2028	2016	3 C38	1	6.35	4	18.02	0.1
2028	2017	2 C38	1	4.19	7	17.72	0.1
2028	2018	2 C38	1	4.09	11	17.37	0.1
2028	2019	2 C38	1	7.01	16	18.02	0.1
2031	2011	1 C35	1	6.93	3	14.72	0.1
2031	2012	1 C35	1	4.39	5	13.72	0.1
2031	2013	1 C35	1	6.09	5	13.69	0.12
2031	2014	1 C35	1	4.52	6	13.69	0.12
2031	2015	3 C35	1	3.12	6	14.42	0.13
2031	2016	1 C35	1	0.96	6	14.82	0.13
2031	2017	3 C35	1	2.19	7	14.52	0.14
2031	2018	2 C35	1	2	7	14.32	0.16
2031	2019	1 C35	1	1.76	7	14.82	0.17
2032	2012	2 C33	1	11.01	1	17.54	0.1
2032	2013	3 C33	1	11.98	1	17.54	0.1
2032	2014	4 C33	1	12.37	1	17.7	0.1
2032	2015	5 C33	1	14.06	1	17.75	0.1
2032	2016	6 C33	1	14.87	1	17.75	0.1
2032	2017	7 C33	1	15.36	2	17.88	0.15
2032	2018	8 C33	1	16.85	2	18.23	0.15
2032	2019	9 C33	1	17.04	3	18.23	0.13
2036	2016	12 C39	1	6.8	2	16.77	0.1
2036	2017	10 C39	1	6.75	2	16.77	0.1
2036	2018	12 C39	1	3.79	3	16.77	0.1
2036	2019	18 C39	1	3.18	4	16.77	0.1
2042	2015	1 C17	1	3.72	3	10.41	0.13
2042	2016	3 C17	1	5.03	3	10.41	0.13

2042	2017	1	C17	1	5.2	3	13.08	0.17
2042	2018	1	C17	1	4.37	3	13.68	0.17
2042	2019	3	C17	1	2.23	3	13.68	0.17
2043	2019	1	C20	1	11.62	2	17.48	0.2
2045	2019	1	C39	1	7.73	6	17.95	0.12
2046	2019	1	C39	0	7.73	6	17.95	0.12
2047	2014	24	E50	0	7.08	3	17.43	0.1
2047	2015	34	E50	0	6.38	4	17.95	0.23
2047	2016	22	E50	0	5.37	3	18.21	0.13
2047	2017	21	E50	0	4.48	10	17.07	0.2
2047	2019	36	E50	0	2.05	13	11.49	0.16
2048	2019	1	C36	1	7.62	2	19.99	0.1
2049	2010	1	C36	1	10.97	6	16.13	0.1
2049	2011	4	C36	1	9.36	4	16.27	0.1
2049	2012	4	C36	1	5.82	7	16.45	0.1
2049	2017	2	C36	1	12.06	5	16.3	0.1
2050	2010	1	C36	0	10.97	6	16.13	0.1
2050	2011	4	C36	0	9.36	4	16.27	0.1
2050	2012	4	C36	0	5.82	7	16.45	0.1
2050	2013	2	C36	0	5.61	4	16.89	0.1
2051	2012	1	E48	1	4.64	8	16.63	0.15
2051	2013	1	E48	1	4.21	9	16.91	0.14
2051	2016	1	E48	1	6.38	11	16.18	0.14
2051	2017	1	E48	1	7.29	11	16.15	0.15
2051	2018	1	E48	1	6.43	13	16.31	0.18
2051	2019	4	E48	1	5.22	13	16.49	0.18
2052	2014	1	E48	0	4.49	10	17.23	0.14
2052	2015	1	E48	0	5.23	11	15.84	0.14
2052	2016	1	E48	0	6.38	11	16.18	0.14
2052	2017	1	E48	0	7.29	11	16.15	0.15
2052	2018	1	E48	0	6.43	13	16.31	0.18
2052	2019	4	E48	0	5.22	13	16.49	0.18
2053	2012	1	E48	1	4.64	8	16.63	0.15
2053	2013	1	E48	1	4.21	9	16.91	0.14
2053	2014	1	E48	1	4.49	10	17.23	0.14
2053	2015	1	E48	1	5.23	11	15.84	0.14
2053	2016	1	E48	1	6.38	11	16.18	0.14
2053	2017	1	E48	1	7.29	11	16.15	0.15
2053	2018	1	E48	1	6.43	13	16.31	0.18
2053	2019	4	E48	1	5.22	13	16.49	0.18
2054	2017	1	C26	1	1.36	2	18.96	0.1
2054	2018	1	C26	1	2.13	2	18.87	0.1
2055	2014	2	C39	1	2.78	5	15.67	0.1
2055	2015	41	C39	1	1.38	9	15.67	0.11
2055	2016	24	C39	1	-0.22	9	15.47	0.12
2055	2017	8	C39	1	1.78	6	15.31	0.17
2055	2018	9	C39	1	2.49	6	15.15	0.17
2055	2019	8	C39	1	-6.08	5	15.29	0.16
2056	2012	2	C38	1	-5.21	4	16.44	0.1
2056	2013	4	C38	1	6.44	4	16.64	0.1
2056	2014	1	C38	1	8.31	4	17.64	0.1

2056	2015	2	C38	1	6.51	4	17.64	0.1
2056	2016	2	C38	1	8.04	4	17.64	0.1
2056	2017	2	C38	1	9.34	4	16.48	0.1
2056	2018	12	C38	1	10.33	4	16.48	0.1
2056	2019	10	C38	1	9.07	4	16.48	0.1
2061	2019	8	E48	1	2.41	2	19.31	0.1
2062	2011	1	E48	0	1.46	1	18.43	0.1
2062	2012	1	E48	0	1.01	1	18.53	0.1
2062	2013	1	E48	0	1.4	1	18.63	0.1
2062	2014	1	E48	0	1.51	1	18.67	0.1
2062	2015	1	E48	0	1.48	1	19.6	0.1
2062	2016	1	E48	0	1.63	1	18.42	0.1
2062	2017	1	E48	0	2.07	1	19.3	0.1
2062	2018	1	E48	0	1.44	1	20.36	0.1
2062	2019	1	E48	0	2.56	1	20.58	0.2
2064	2015	1	C28	1	5.18	1	19.81	0.1
2064	2016	1	C28	1	-5.64	1	19.84	0.1
2064	2017	1	C28	1	7.04	1	19.83	0.1
2064	2018	1	C28	1	7.74	1	19.83	0.1
2064	2019	1	C28	1	16.07	4	19.98	0.13
2067	2014	2	C22	1	0.06	2	18.13	0.1
2067	2015	3	C22	1	0.04	2	18.13	0.1
2067	2016	4	C22	1	5.53	2	18.13	0.1
2067	2017	5	C22	1	11.55	2	17.84	0.1
2067	2018	6	C22	1	5.64	2	17.75	0.1
2067	2019	7	C22	1	3.38	3	17.75	0.13
2069	2012	2	A04	1	2.22	6	16.87	0.1
2069	2013	4	A04	1	1.9	6	16.86	0.1
2069	2014	4	A04	1	-23.45	7	16.76	0.11
2069	2015	1	A04	1	-5.24	7	16.77	0.13
2069	2016	1	A04	1	-1.28	7	17.06	0.11
2069	2017	7	A04	1	-10.99	6	17.06	0.13
2069	2018	5	A04	1	0.91	6	16.53	0.15
2069	2019	12	A04	1	-11.73	6	16.53	0.12
2070	2014	4	A04	0	-23.45	7	16.76	0.11
2070	2015	1	A04	0	-5.24	7	16.76	0.13
2070	2016	1	A04	0	-1.28	7	16.76	0.11
2070	2017	7	A04	0	-10.99	6	16.06	0.13
2070	2018	5	A04	0	0.91	6	15.53	0.15
2070	2019	12	A04	0	-11.73	6	15.53	0.12
2073	2010	1	C35	1	10.88	1	16.22	0.1
2073	2011	1	C35	1	9.74	3	15.86	0.13
2073	2012	1	C35	1	3.6	3	15.37	0.13
2073	2013	1	C35	1	1.91	3	15.37	0.17
2073	2014	1	C35	1	2.6	3	15.37	0.17
2073	2015	1	C35	1	2.47	3	15.37	0.17
2073	2016	1	C35	1	-9.65	3	15.37	0.17
2073	2018	1	C35	1	-3.95	3	15.37	0.13
2074	2017	2	C38	1	6.15	2	18.85	0.1
2074	2018	1	C38	1	3.09	4	18.63	0.1
2074	2019	1	C38	1	0.21	4	18.63	0.1

2078	2010	1	C22	1	7.21	2	19.39	0.15
2078	2011	1	C22	1	4.2	3	18.4	0.1
2078	2012	1	C22	1	1.44	3	18.3	0.1
2078	2013	1	C22	1	2.05	2	18.3	0.1
2078	2014	2	C22	1	3.4	2	18.4	0.1
2078	2015	1	C22	1	4.19	2	19.39	0.1
2078	2016	1	C22	1	5.8	2	19.39	0.1
2078	2017	3	C22	1	9.73	4	19.39	0.1
2078	2018	2	C22	1	8.07	3	19.64	0.13
2078	2019	2	C22	1	7.11	3	19.76	0.13
2080	2018	1	C30	0	4.27	2	18.26	0.1
2080	2019	1	C30	0	5.36	1	19.59	0.1
2081	2016	338	E50	1	6.57	8	11.41	0.11
2081	2017	29	E50	1	7.01	9	14.11	0.16
2081	2018	24	E50	1	7.06	11	14.78	0.15
2081	2019	73	E50	1	6.21	10	14.64	0.18
2084	2017	29	E50	0	7.01	9	14.11	0.16
2084	2018	24	E50	0	7.06	11	14.78	0.15
2084	2019	73	E50	0	6.21	10	14.84	0.18
2085	2013	1	C36	0	10.67	3	17.16	0.1
2085	2014	1	C36	0	11.42	3	17.16	0.1
2085	2015	4	C36	0	13.12	7	18.39	0.21
2086	2015	4	C36	1	13.12	7	16.39	0.21
2090	2011	21	C38	1	4.41	2	19.82	0.15
2090	2012	23	C38	1	4.19	2	18.54	0.25
2090	2013	27	C38	1	4.53	1	19.45	0.4
2090	2014	25	C38	1	7.24	1	19.65	0.6
2090	2015	66	C38	1	6.48	1	19.9	0.5
2090	2016	42	C38	1	4.43	1	20.9	0.5
2090	2017	47	C38	1	3.72	1	20.9	0.5
2090	2018	52	C38	1	2.3	2	20.9	0.3
2090	2019	39	C38	1	2.47	2	20.8	0.1
2091	2018	4	F51	1	8.26	6	17.72	0.55
2091	2019	9	F51	1	6.41	9	17.42	0.47
2092	2018	4	F51	1	8.26	6	17.72	0.55
2092	2019	9	F51	1	6.41	9	17.42	0.47
2093	2015	1	F51	1	7.64	2	18.02	0.1
2093	2016	11	F51	1	11.67	7	17.01	0.21
2093	2017	5	F51	1	6.92	7	17.37	0.34
2093	2019	9	F51	1	6.41	9	17.42	0.47
2094	2010	1	C26	1	4.26	2	18.68	0.1
2094	2011	2	C26	1	5.35	2	17.18	0.1
2094	2012	3	C26	1	4.94	2	17.13	0.1
2094	2013	4	C26	1	4.88	2	17.13	0.1
2094	2014	5	C26	1	4.21	2	17.7	0.1
2094	2015	6	C26	1	7.19	2	18.68	0.1
2094	2016	7	C26	1	8.3	2	18.68	0.1
2094	2017	8	C26	1	11.79	2	18.98	0.15
2094	2018	9	C26	1	2.93	2	19.1	0.2
2094	2019	10	C26	1	0.85	2	19.1	0.25
2095	2010	1	I64	0	10.54	2	14.79	0.1

2095	2011	2	I64	0	6.33	2	14.79	0.1
2095	2012	3	I64	0	6.81	2	14.86	0.1
2095	2013	4	I64	0	5.85	2	15.01	0.1
2095	2014	5	I64	0	5.72	2	15.55	0.1
2096	2019	10	F51	1	6.41	9	17.42	0.47
2097	2014	1	C35	1	0.07	5	15.36	0.12
2097	2015	1	C35	1	-0.55	9	15.32	0.11
2097	2016	2	C35	1	1.14	10	15.18	0.11
2097	2017	1	C35	1	1.86	9	15.96	0.11
2097	2018	1	C35	1	3.47	9	15.77	0.11
2097	2019	3	C35	1	3.45	13	15.29	0.16
2098	2016	11	F51	0	11.67	7	17.01	0.21
2098	2017	5	F51	0	6.92	7	17.37	0.34
2099	2012	1	C27	0	1.21	2	14.61	0.1
2099	2013	1	C27	0	-4.93	2	14.4	0.1
2099	2014	1	C27	0	1.42	2	14.4	0.1
2099	2015	1	C27	0	11.32	2	14.4	0.1
2100	2016	11	F51	1	11.67	7	17.01	0.21
2100	2017	5	F51	1	6.92	7	17.37	0.34
2101	2015	1	C35	1	-0.55	9	15.32	0.11
2101	2016	2	C35	1	1.14	10	15.18	0.11
2101	2017	1	C35	1	1.86	9	15.96	0.11
2101	2018	1	C35	1	3.47	9	15.77	0.11
2101	2019	3	C35	1	3.45	13	15.29	0.16
2102	2015	1	C35	1	-0.55	9	15.32	0.11
2102	2016	2	C35	1	1.14	10	15.18	0.11
2102	2017	1	C35	1	1.86	9	15.96	0.11
2103	2016	27	L72	0	4.93	4	18.91	0.15
2103	2017	20	L72	0	4.19	4	18.65	0.18
2103	2018	20	L72	0	-28.76	4	18.03	0.18
2103	2019	22	L72	1	0.61	4	16.03	0.18
2104	2012	13	C39	1	13.17	2	16.17	0.1
2104	2013	24	C39	1	17.41	2	16.07	0.1
2104	2015	26	C39	1	21.36	2	17.11	0.1
2104	2016	53	C39	1	7.73	2	18.42	0.1
2104	2017	67	C39	1	7.78	2	18.33	0.1
2104	2018	93	C39	1	6.65	4	17.07	0.1
2104	2019	59	C39	1	3.78	5	18.42	0.12
2105	2018	1	C37	1	-0.5	3	15.69	0.13
2105	2019	1	C37	1	1.45	3	15.69	0.17
2106	2018	1	C35	1	3.47	9	15.77	0.11
2106	2019	3	C35	1	3.45	13	15.29	0.16
2107	2019	3	C35	1	3.45	13	15.29	0.16
2108	2019	3	C35	1	3.45	13	15.29	0.16
2111	2011	1	C35	0	5.46	3	16.61	0.1
2111	2012	1	C35	0	5.21	3	16.05	0.1
2111	2013	1	C35	0	4.76	3	16.05	0.1
2111	2017	3	C35	0	2.72	3	15.56	0.1
2111	2018	5	C35	0	5.18	3	15.56	0.1
2112	2019	3	C37	0	1.45	3	16.13	0.17
2120	2011	1	G60	1	2.8	3	16.97	0.13

2120	2012	1	G60	1	9.06	3	16.97	0.13
2123	2012	3	I65	0	2.83	2	17.04	0.1
2124	2013	1	A03	0	8.29	3	16.91	0.2
2124	2014	4	A03	0	1.46	4	16.41	0.18
2124	2015	1	A03	0	5.59	4	17.18	0.15
2124	2016	7	A03	0	16.72	5	17.18	0.18
2124	2017	3	A03	0	7.48	5	18.21	0.14
2124	2018	6	A03	0	-10.78	5	18.77	0.14
2124	2019	6	A03	0	1.45	4	18.77	0.15
2126	2015	2	C36	0	5.72	3	17.04	0.2
2126	2017	4	C36	0	5.98	5	17.82	0.24
2126	2018	3	C36	0	5.48	6	17.03	0.22
2126	2019	4	C36	0	4.29	9	17.52	0.19
2127	2018	3	C36	1	5.48	6	17.03	0.22
2127	2019	4	C36	1	4.29	9	17.52	0.19
2130	2019	1	C39	1	3.51	3	16.68	0.13
2131	2012	91	I64	0	1.88	4	17.28	0.1
2131	2013	88	I64	0	2.08	4	17.28	0.1
2131	2019	88	I64	0	2.25	8	18.04	0.11
2132	2016	92	I64	0	5.91	1	17.87	0.1
2132	2018	88	I64	0	-13.46	5	17.94	0.16
2133	2017	91	I64	1	3.55	4	18.6	0.1
2133	2018	88	I64	1	-13.46	5	18.36	0.16
2133	2019	88	I64	1	2.25	8	18.41	0.11
2139	2016	82	C38	1	7.07	2	14.44	0.1
2139	2017	91	C38	1	7.59	2	14.38	0.1
2139	2018	88	C38	1	6.62	2	14.33	0.1
2139	2019	88	C38	1	7.51	4	14.44	0.1
2141	2019	1	C38	0	0.35	2	20.01	0.15
2151	2016	22	C39	1	1.27	5	16.85	0.18
2151	2018	23	C39	1	1.99	7	17.18	0.39
2152	2011	4	C34	1	16.5	3	17.84	0.13
2152	2012	22	C34	1	15.72	4	17.53	0.13
2152	2014	25	C34	1	13.97	5	16.39	0.12
2152	2015	24	C34	1	12.79	5	16.39	0.12
2152	2016	24	C34	1	9.1	5	16.83	0.14
2152	2017	72	C34	1	7.84	6	18.56	0.13
2152	2018	159	C34	1	6	8	18.18	0.18
2152	2019	171	C34	1	6.39	8	18.2	0.14
2153	2016	1	I65	1	7.16	11	17.74	0.24
2153	2017	2	I65	1	6.62	13	18.12	0.27
2153	2018	3	I65	1	6.38	20	18.88	0.2
2153	2019	4	I65	1	4.4	24	19.43	0.18
2154	2013	22	C34	1	15.67	5	16.38	0.12
2154	2014	25	C34	1	13.97	5	16.39	0.12
2154	2015	24	C34	1	12.79	5	16.39	0.12
2154	2016	24	C34	1	9.1	5	16.83	0.14
2154	2017	72	C34	1	7.84	6	18.56	0.13
2154	2018	159	C34	1	6	8	18.18	0.18
2155	2018	159	C34	1	6	8	18.18	0.18
2155	2019	171	C34	1	6.39	8	18.2	0.14

2157	2016	24	I65	1	7.16	11	17.74	0.24
2157	2017	72	I65	1	6.62	13	18.12	0.27
2157	2018	159	I65	1	6.38	20	19.88	0.2
2158	2013	3	C34	1	13.91	2	14.31	0.1
2158	2014	3	C34	1	15.21	2	14.61	0.1
2158	2015	3	C34	1	8.85	3	15.11	0.1
2158	2016	3	C34	1	6.83	3	15.11	0.1
2158	2017	3	C34	1	8.07	3	16.13	0.17
2158	2018	3	C34	1	6.37	4	16.98	0.15
2158	2019	3	C34	1	7.25	5	16.96	0.14
2159	2019	1	I65	1	4.4	24	16.43	0.18
2161	2016	85	C39	1	26.48	5	16.19	0.14
2161	2017	100	C39	1	17.74	5	16.13	0.14
2161	2018	77	C39	1	19.85	6	16.82	0.13
2161	2019	71	C39	1	11.04	6	16.84	0.13
2162	2019	2	C34	0	7.25	5	15.96	0.14
2163	2018	3	C34	1	6.37	4	15.98	0.15
2168	2019	1	I64	1	5.38	2	18.26	0.1
2174	2016	4	I64	1	15.65	11	17.15	0.16
2174	2017	3	I64	1	11.71	12	17.42	0.17
2174	2018	3	I64	1	14.48	11	17.49	0.15
2174	2019	3	I64	1	3.15	13	17.85	0.15
2180	2019	47	C39	1	2.34	8	12.89	0.34
2182	2018	1	C32	1	7.97	2	18.51	0.1
2183	2011	1	L72	1	0.89	7	10.79	0.4
2183	2013	2	L72	0	1.66	7	17.76	0.53
2183	2014	3	L72	0	1.78	6	18.14	0.62
2183	2015	4	L72	0	1.72	7	18.79	0.56
2183	2016	5	L72	0	1.42	6	19.07	0.65
2183	2017	6	L72	0	1.3	7	19.22	0.56
2183	2018	7	L72	0	0.35	7	19.34	0.54
2183	2019	8	L72	0	0.05	6	19.8	0.57
2185	2019	1	C39	1	2.06	5	15.82	0.22
2195	2012	10	I65	1	14.93	1	15.89	0.1
2195	2013	14	I65	1	6	1	16.45	0.1
2195	2014	60	I65	1	4.75	2	16.84	0.1
2195	2015	83	I65	1	9.17	2	16.84	0.1
2196	2012	1	C35	1	2.99	1	11.29	0.1
2196	2013	1	C35	1	0.69	1	11.29	0.1
2196	2014	1	C35	1	0.94	1	12.8	0.1
2196	2016	8	C35	1	4.2	1	14.93	0.1
2196	2017	7	C35	1	4.26	1	15.15	0.1
2196	2018	2	C35	1	-14.43	1	16.62	0.1
2196	2019	11	C35	1	0.58	2	16.62	0.1
2203	2017	2	C32	1	5.14	7	19.18	0.17
2203	2018	2	C32	1	5.37	6	19.38	0.22
2203	2019	1	C32	1	4.95	13	19.63	0.16
2204	2013	1	C35	1	1.71	2	17.56	0.1
2204	2014	2	C35	1	0.03	2	16.23	0.1
2204	2015	3	C35	1	0.03	3	15.69	0.1
2204	2016	4	C35	1	0.05	4	15.43	0.1

2204	2017	5	C35	1	0.12	5	15.01	0.1
2204	2018	6	C35	1	0.02	5	14.76	0.1
2204	2019	7	C35	1	0.22	6	15.97	0.1
2206	2010	1	C32	1	4.8	3	19.94	0.13
2206	2011	1	C32	1	3.76	3	19.84	0.13
2206	2012	1	C32	1	3.49	4	19.65	0.13
2206	2013	1	C32	1	4.19	5	19.65	0.14
2206	2014	1	C32	1	5.82	5	19.84	0.14
2206	2015	1	C32	1	5.49	5	19.94	0.14
2206	2016	2	C32	1	5.14	6	19.94	0.15
2207	2015	1	B11	1	-18.3	2	18.66	0.1
2207	2016	2	B11	1	-9.64	2	18.65	0.1
2207	2017	2	B11	1	1.19	2	19.37	0.2
2208	2019	1	C35	1	0.22	6	15.97	0.1
2209	2019	1	C35	1	-2.54	3	13.82	0.1
2211	2017	10	C26	1	8.33	2	19.3	0.1
2211	2018	13	C26	1	4.4	2	19.72	0.1
2211	2019	13	C26	1	4.4	1	20.41	0.1
2215	2013	3	C26	0	7.75	1	20.33	0.1
2215	2014	2	C26	0	7.6	1	20.33	0.1
2215	2015	4	C26	0	8.32	1	20.33	0.1
2215	2016	11	C26	0	-8.88	1	20.33	0.1
2215	2017	10	C26	0	8.33	2	20.33	0.1
2215	2018	13	C26	0	6.81	2	20.33	0.1
2217	2017	15	C39	1	6.1	5	18.09	0.14
2217	2018	8	C39	1	5.18	5	18.04	0.14
2217	2019	15	C39	1	3.29	5	15.41	0.14
2224	2017	1	C29	1	8.55	1	18.24	0.1
2224	2018	1	C29	1	3.42	1	18.57	0.1
2224	2019	1	C29	1	4.75	1	18.99	0.1
2225	2010	1	C30	1	8.21	2	11.92	0.1
2225	2011	1	C30	1	4.95	2	11.13	0.1
2225	2012	1	C30	1	3.42	2	14.96	0.1
2225	2013	1	C30	1	3.74	2	14.89	0.1
2225	2014	1	C30	1	3.8	2	14.05	0.1
2225	2015	1	C30	1	1.77	3	15.62	0.1
2225	2016	1	C30	1	-3.59	3	16.33	0.1
2225	2017	1	C30	1	0.66	3	17.34	0.1
2225	2018	1	C30	1	4.76	1	16.39	0.1
2226	2015	1	C30	1	1.77	3	14.62	0.1
2226	2016	1	C30	1	-3.59	3	15.33	0.1
2226	2017	1	C30	1	0.66	3	15.33	0.1
2228	2018	7	C22	1	4.24	5	16.12	0.12
2228	2019	7	C22	1	4.29	5	16.24	0.12
2229	2018	7	C22	1	4.24	5	16.12	0.12
2229	2019	7	C22	1	4.29	5	16.24	0.12
2230	2016	160	I65	1	5.28	2	18.79	0.1
2230	2017	238	I65	1	4.03	3	18.48	0.1
2230	2018	267	I65	1	4.32	3	18.65	0.1
2230	2019	239	I65	1	5.33	4	18.47	0.13
2235	2010	1	I64	1	0.81	2	14.49	0.1

2235	2011	2	I64	1	0.88	2	14.49	0.1
2235	2012	3	I64	1	1.36	2	14.49	0.1
2236	2018	117	C39	1	10.88	42	15.3	0.11
2236	2019	134	C39	1	11.31	44	15.23	0.1
2237	2016	66	C39	1	13.47	24	14.25	0.1
2237	2017	112	C39	1	12.95	35	14.25	0.11
2237	2018	117	C39	1	10.88	42	14.25	0.11
2237	2019	134	C39	1	11.31	44	14.23	0.1
2238	2016	66	C39	1	13.47	24	14.25	0.1
2238	2017	112	C39	1	12.95	35	14.38	0.11
2238	2018	117	C39	1	10.88	42	15.3	0.11
2238	2019	134	C39	1	11.31	44	16.23	0.1
2239	2017	15	C36	1	4.31	13	16.54	0.15
2239	2018	8	C36	1	0.41	13	16.54	0.15
2239	2019	3	C36	1	1.23	14	17.06	0.14
2240	2015	36	C39	1	14.17	13	13.22	0.1
2240	2016	66	C39	1	13.47	24	14.25	0.1
2240	2017	112	C39	1	12.95	35	14.25	0.11
2240	2018	117	C39	1	10.88	42	14.25	0.11
2240	2019	134	C39	1	11.31	44	16.23	0.1
2241	2012	1	C39	1	12.34	6	15.16	0.1
2241	2013	3	C39	1	12.05	7	15.16	0.11
2241	2014	9	C39	1	11.1	8	15.16	0.14
2241	2015	29	C39	1	6.7	9	15.16	0.16
2241	2016	44	C39	1	7.63	8	15.16	0.18
2241	2017	59	C39	1	8.52	8	15.16	0.18
2241	2018	46	C39	1	3	8	15.16	0.15
2241	2019	55	C39	1	3.97	7	15.16	0.17
2242	2017	112	C39	1	12.95	35	14.38	0.11
2242	2018	117	C39	1	10.88	42	15.3	0.11
2242	2019	134	C39	1	11.31	44	16.23	0.1
2243	2017	112	C39	1	12.95	35	14.38	0.11
2243	2018	117	C39	1	10.88	42	14.38	0.11
2243	2019	134	C39	1	11.31	44	14.38	0.1
2244	2017	112	C39	1	12.95	35	14.28	0.11
2244	2018	117	C39	1	10.88	42	15.3	0.11
2244	2019	134	C39	1	11.31	44	15.3	0.1
2245	2018	117	C39	1	10.88	42	15.3	0.11
2245	2019	134	C39	1	11.31	44	16.23	0.1
2246	2016	66	C39	1	13.47	24	14.25	0.1
2246	2017	112	C39	1	12.95	35	14.18	0.11
2246	2018	117	C39	1	10.88	42	15.2	0.11
2246	2019	134	C39	1	11.31	44	16.22	0.1
2247	2018	117	C39	1	10.88	42	15.3	0.11
2247	2019	134	C39	1	11.31	44	16.23	0.1
2248	2018	117	C39	1	10.88	42	15.3	0.11
2248	2019	134	C39	1	11.31	44	16.23	0.1
2249	2015	2	C38	1	5.35	8	16.74	0.14
2249	2016	2	C38	1	4.96	8	17.33	0.15
2249	2017	1	C38	1	2.84	7	17.33	0.16
2249	2018	4	C38	1	-15.16	9	17.33	0.16

2249	2019	5	C38	1	0.22	9	17.49	0.19
2250	2017	112	C39	1	12.95	35	14.38	0.11
2250	2018	117	C39	1	10.88	42	15.3	0.11
2250	2019	134	C39	1	11.31	44	16.23	0.1
2251	2018	117	C39	1	10.88	42	16.3	0.11
2251	2019	134	C39	1	11.31	44	16.23	0.1
2252	2016	66	C39	1	13.47	24	14.25	0.1
2252	2017	112	C39	1	12.95	35	14.38	0.11
2252	2018	117	C39	1	10.88	42	15.3	0.11
2252	2019	134	C39	1	11.31	44	16.37	0.1
2253	2017	112	C39	1	12.95	35	15.3	0.11
2253	2018	117	C39	1	10.88	42	15.29	0.11
2253	2019	134	C39	1	11.31	44	16.23	0.1
2254	2017	112	C39	1	12.95	35	16.3	0.11
2254	2018	117	C39	1	10.88	42	15.3	0.11
2254	2019	134	C39	1	11.31	44	15.39	0.1
2255	2015	15	C36	1	6.02	10	16.35	0.16
2255	2016	8	C36	1	5.99	10	16.57	0.16
2255	2017	3	C36	1	4.31	13	16.59	0.15
2255	2018	15	C36	1	0.41	13	16.58	0.15
2255	2019	8	C36	1	1.23	14	17.06	0.14
2256	2015	3	C36	0	6.02	10	16.35	0.16
2256	2016	15	C36	0	5.99	10	16.57	0.16
2256	2017	8	C36	0	4.31	13	16.54	0.15
2256	2018	3	C36	0	0.41	13	16.31	0.15
2256	2019	3	C36	0	1.23	14	16.57	0.14
2257	2013	3	C39	1	12.05	7	15.82	0.11
2257	2014	9	C39	1	11.1	8	16.54	0.14
2257	2015	29	C39	1	6.7	9	16.54	0.16
2257	2016	44	C39	1	7.63	8	17.12	0.18
2257	2017	59	C39	1	8.52	8	17.26	0.18
2257	2018	46	C39	1	3	8	17.47	0.15
2257	2019	55	C39	1	3.97	7	17.47	0.17
2258	2018	1	C26	1	11.75	2	8.47	0.15
2259	2015	2	C38	1	5.35	8	16.74	0.14
2259	2016	2	C38	1	4.96	8	16.74	0.15
2259	2017	1	C38	1	2.84	7	17.26	0.16
2259	2018	4	C38	1	-15.16	9	17.93	0.16
2259	2019	5	C38	1	0.22	9	17.93	0.19
2260	2015	2	C38	1	5.35	8	16.74	0.14
2260	2016	2	C38	1	4.96	8	17.33	0.15
2260	2017	1	C38	1	2.84	7	17.26	0.16
2260	2018	4	C38	1	-15.16	9	16.73	0.16
2260	2019	5	C38	1	0.22	9	17.33	0.19
2261	2013	36	I65	1	4.99	2	16.68	0.1
2261	2014	33	I65	1	6.35	2	16.68	0.1
2261	2017	37	I65	1	1.54	4	16.68	0.13
2262	2018	4	C38	1	-15.16	9	16.93	0.16
2262	2019	5	C38	1	0.22	9	16.93	0.19
2263	2017	37	I65	1	1.54	4	17.33	0.13
2263	2018	43	I65	1	-36.28	3	17.21	0.13

2263	2019	58	I65	1	0.69	3	17.21	0.13
2264	2012	1	C39	1	12.34	6	15.16	0.1
2264	2013	3	C39	1	12.05	7	15.82	0.11
2264	2014	9	C39	1	11.1	8	16.54	0.14
2264	2015	29	C39	1	6.7	9	16.54	0.16
2264	2016	44	C39	1	7.63	8	16.54	0.18
2264	2017	59	C39	1	8.52	8	15.17	0.18
2264	2018	46	C39	1	3	8	15.16	0.15
2264	2019	55	C39	1	3.97	7	15.16	0.17
2266	2016	4	N77	1	2.08	5	19.51	0.14
2266	2017	3	N77	1	1.73	5	20.15	0.16
2266	2018	1	N77	1	1.65	4	19.71	0.15
2266	2019	1	N77	1	2.26	4	19.91	0.15
2273	2014	10	C39	0	10.27	1	18.59	0.1
2273	2015	11	C39	0	6.24	1	18.59	0.1
2273	2016	11	C39	0	7.96	2	18.59	0.1
2273	2017	13	C39	0	8.47	3	18.06	0.17
2273	2018	6	C39	0	8.85	3	18	0.13
2273	2019	9	C39	0	8.27	3	18	0.13
2274	2017	13	C39	1	8.47	3	18.56	0.17
2274	2018	6	C39	1	8.85	3	18.9	0.13
2274	2019	9	C39	1	8.27	3	19.01	0.13
2276	2019	1	C38	1	3.12	4	15.55	0.1
2277	2019	1	C38	1	3.12	4	15.55	0.1
2282	2015	7	C34	1	0.64	3	16.71	0.17
2282	2016	5	C34	1	1.21	3	16.71	0.13
2282	2018	4	C34	1	3.45	4	16.71	0.18
2282	2019	2	C34	1	2.68	4	16.71	0.23
2283	2014	1	C34	1	2.92	3	16.77	0.13
2283	2018	4	C34	1	3.45	4	16.77	0.18
2283	2019	2	C34	1	2.68	4	16.86	0.23
2284	2016	19	C36	0	3.31	1	18.63	0.1
2284	2017	17	C36	0	1.58	1	18.63	0.1
2284	2018	21	C36	0	0.21	1	18.63	0.1
2284	2019	42	C36	0	-1.63	1	19.61	0.1
2292	2017	7	C24	1	0.82	7	17.99	0.26
2292	2018	9	C24	1	-22.48	7	18.04	0.24
2292	2019	9	C24	1	1.61	7	18.04	0.23
2293	2019	10	C30	1	3.47	3	18.55	0.1
2294	2019	10	C30	1	3.47	3	18.55	0.1
2302	2017	10	C30	1	0.88	1	19.44	0.1
2302	2018	10	C30	1	1.78	2	18.75	0.1
2303	2019	18	C22	1	7.78	4	15.89	0.13
2309	2012	1	C38	1	3.08	9	16.83	0.43
2309	2013	1	C38	1	1.88	10	16.8	0.5
2309	2014	1	C38	1	1.54	12	16.8	0.35
2309	2015	10	C38	1	2.74	13	16.8	0.35
2309	2016	1	C38	1	0.4	14	16.81	0.46
2309	2017	2	C38	1	1.35	14	17	0.47
2309	2018	1	C38	1	-0.72	16	16.82	0.4
2309	2019	1	C38	1	0.51	17	17.38	0.57

2310	2013	1	C38	0	1.88	10	16.9	0.5
2310	2014	1	C38	0	1.54	12	16.93	0.35
2310	2015	10	C38	0	2.74	13	16.93	0.35
2310	2016	1	C38	0	0.4	14	16.93	0.46
2310	2017	2	C38	0	1.35	14	16.5	0.47
2310	2018	1	C38	0	-0.72	16	16.45	0.4
2310	2019	1	C38	0	0.51	17	16.45	0.57
2311	2019	2	C13	1	9.88	10	15.65	0.41
2312	2015	10	C38	1	2.74	13	16.72	0.35
2312	2016	1	C38	1	0.4	14	16.74	0.46
2312	2017	2	C38	1	1.35	14	17	0.47
2312	2018	1	C38	1	-0.72	16	16.85	0.4
2312	2019	1	C38	1	0.51	17	17.38	0.57
2313	2016	1	C38	1	0.4	14	16.44	0.46
2313	2017	2	C38	1	1.35	14	16.4	0.47
2313	2018	1	C38	1	-0.72	16	16.42	0.4
2313	2019	1	C38	1	0.51	17	16.72	0.57
2314	2019	1	C38	1	0.51	17	17.38	0.57
2315	2019	1	I64	1	5.47	7	15.7	0.19
2316	2015	1	I65	0	0.95	2	18.21	0.1
2317	2014	2	C13	1	7.31	7	12.2	0.21
2317	2015	3	C13	1	9.99	7	15.01	0.29
2317	2016	4	C13	1	9.45	7	15.84	0.34
2317	2017	5	C13	1	10.46	9	15.1	0.34
2317	2018	6	C13	1	9.72	9	14.97	0.4
2317	2019	7	C13	1	9.88	10	15.84	0.41
2318	2017	5	C13	1	10.46	9	16.1	0.34
2318	2018	6	C13	1	9.72	9	15.07	0.4
2318	2019	7	C13	1	9.88	10	15.95	0.41
2319	2012	1	C13	1	8.77	4	13.8	0.25
2319	2013	2	C13	1	5	5	13.8	0.24
2319	2014	2	C13	1	7.31	7	13.9	0.21
2319	2015	3	C13	1	9.99	7	14.51	0.29
2319	2016	4	C13	1	9.45	7	14.51	0.34
2319	2017	5	C13	1	10.46	9	15.1	0.34
2319	2018	6	C13	1	9.72	9	15.27	0.4
2319	2019	7	C13	1	9.88	10	15.27	0.41
2325	2017	2	E50	0	1.26	2	17.07	0.1
2325	2018	1	E50	0	-3.63	3	17.07	0.1
2325	2019	1	E50	0	0.68	3	17.07	0.1
2334	2017	11	C38	1	7.32	4	19.44	0.19
2335	2017	11	C38	1	7.32	4	19.44	0.19
2340	2019	1	C42	0	2.89	5	18.96	0.16
2341	2015	4	C26	1	-3.43	1	17.83	0.18
2341	2016	5	C26	1	-0.58	2	17.83	0.19
2341	2017	6	C26	1	1.14	2	17.74	0.19
2341	2018	7	C26	1	3.44	3	17.1	0.2
2341	2019	8	C26	1	-0.01	6	17.1	0.21
2342	2018	7	C26	1	3.44	3	19.86	0.2
2342	2019	8	C26	1	-0.01	6	19.86	0.21
2343	2019	8	C26	1	-0.01	6	20.73	0.21

2344	2017	1	L72	1	2.38	1	19.7	0.1
2345	2019	1	C41	0	1.5	2	19.55	0.1
2352	2016	52	G60	0	18.48	3	21.36	0.13
2352	2019	56	G60	0	6.85	3	20.42	0.1
2360	2013	1	C26	0	13.49	1	17.51	0.18
2360	2014	2	C26	0	10.54	1	17.33	0.17
2360	2016	3	C26	0	6.79	1	17.4	0.17
2365	2019	1	C27	1	5.57	1	19.28	0.19
2372	2019	1	C29	1	20.87	3	18.01	0.1
2373	2019	1	I65	1	7.19	6	19.4	0.19
2376	2011	2	C39	1	11.4	1	18.27	0.18
2376	2012	3	C39	1	12.84	1	18.27	0.18
2376	2013	4	C39	1	11.58	1	18.27	0.19
2376	2014	5	C39	1	13.09	1	18.27	0.18
2378	2017	4	C32	0	0.91	3	17.3	0.13
2378	2018	2	C32	0	1.26	3	17.3	0.13
2378	2019	3	C32	0	-7.65	3	17.3	0.13
2382	2018	4	C35	1	4.89	9	21.32	0.21
2383	2017	100	C39	0	3.69	3	20.09	0.2
2383	2018	98	C39	0	2.12	5	20.6	0.21
2383	2019	68	C39	0	-11.68	6	21.61	0.22
2384	2016	8	C39	1	1.35	5	19.65	0.2
2384	2017	3	C39	1	2.85	5	19.65	0.2
2385	2018	4	C35	1	4.89	9	21.32	0.21
2386	2018	5	C35	1	4.89	9	21.32	0.21
2387	2018	98	C39	1	2.12	5	20.6	0.21
2387	2019	68	C39	1	-11.68	6	21.61	0.22
2392	2015	1	C30	1	3.42	2	14.69	0.1
2392	2016	1	C30	1	5.03	2	17.56	0.1
2392	2017	1	C30	1	5.34	2	17.54	0.1
2392	2019	1	C30	1	4.56	2	17.52	0.1
2394	2012	1	C17	1	7.42	4	14.04	0.1
2394	2013	1	C17	1	7.43	4	14.04	0.13
2399	2018	2	C27	1	4.41	12	22.15	0.22
2402	2019	1	C39	1	7.85	3	18.81	0.19
2404	2019	2	C18	1	5.61	3	17.62	0.27
2405	2019	3	C18	1	5.61	3	17.62	0.27
2406	2018	1	I65	1	3.97	3	20.98	0.21
2406	2019	2	I65	1	3.35	3	20.98	0.21
2409	2018	2	C26	1	4.33	3	21.21	0.21
2410	2019	66	I65	1	4.32	6	19.27	0.19
2411	2019	66	I65	1	4.32	6	19.27	0.19
2415	2019	106	C39	1	17.96	35	25.35	0.25
2416	2019	106	C39	1	17.96	35	25.35	0.25
2417	2018	93	C39	1	19.78	27	22.91	0.23
2417	2019	106	C39	1	17.96	35	25.35	0.25
2418	2016	37	C39	1	20.71	17	17.37	0.17
2418	2017	78	C39	1	20.19	23	20.69	0.21
2418	2018	93	C39	1	19.78	27	22.91	0.23
2418	2019	106	C39	1	17.96	35	25.35	0.25
2419	2016	37	C39	0	20.71	17	17.37	0.17

2419	2017	78	C39	0	20.19	23	20.69	0.21
2419	2018	93	C39	0	19.78	27	22.91	0.23
2419	2019	106	C39	0	17.96	35	25.35	0.25
2420	2016	37	C39	1	20.71	17	17.37	0.17
2420	2017	78	C39	1	20.19	23	20.69	0.21
2420	2018	93	C39	1	19.78	27	22.91	0.23
2420	2019	106	C39	1	17.96	35	25.35	0.25
2421	2016	37	C39	1	20.71	17	17.37	0.17
2421	2017	78	C39	1	20.19	23	20.69	0.21
2421	2018	93	C39	1	19.78	27	22.91	0.23
2421	2019	106	C39	1	17.96	35	25.35	0.25
2422	2013	1	C27	0	6.64	1	17.23	0.17
2422	2014	1	C27	0	5.03	2	17.67	0.18
2422	2015	1	C27	0	2.48	2	16.72	0.18
2422	2016	1	C27	0	2.71	2	16.28	0.17
2422	2017	1	C27	0	3.16	1	17.11	0.17
2422	2018	1	C27	0	4.42	2	21.56	0.22
2422	2019	1	C27	0	3.36	2	21.74	0.22
2423	2017	78	C39	1	20.19	23	20.69	0.21
2423	2018	93	C39	1	19.78	27	22.91	0.23
2423	2019	106	C39	1	17.96	35	25.35	0.25
2424	2019	106	C39	1	17.96	35	25.35	0.25
2425	2019	106	C39	1	17.96	35	25.35	0.25
2426	2019	106	C39	1	17.96	35	25.35	0.25
2427	2016	37	C39	1	20.71	17	17.37	0.17
2427	2017	78	C39	1	20.19	23	20.69	0.21
2427	2018	93	C39	1	19.78	27	22.91	0.23
2427	2019	106	C39	1	17.96	35	25.35	0.25
2428	2017	78	C39	1	20.19	23	20.69	0.21
2428	2018	93	C39	1	19.78	27	22.91	0.23
2428	2019	106	C39	1	17.96	35	25.35	0.25
2429	2017	78	C39	1	20.19	23	20.69	0.21
2429	2018	93	C39	1	19.78	27	22.91	0.23
2429	2019	106	C39	1	17.96	35	25.35	0.25
2430	2012	2	C35	0	6.61	2	18.64	0.19
2430	2013	2	C35	0	2.93	2	18.64	0.19
2431	2018	93	C39	1	19.78	27	22.91	0.23
2431	2019	106	C39	1	17.96	35	25.35	0.25
2432	2016	37	C39	0	20.71	17	17.37	0.17
2432	2017	78	C39	0	20.19	23	20.69	0.21
2432	2018	93	C39	0	19.78	27	22.91	0.23
2432	2019	106	C39	0	17.96	35	25.35	0.25
2433	2019	106	C39	1	17.96	35	25.35	0.25
2436	2016	5	C39	0	5.56	3	18.89	0.19
2436	2017	1	C39	0	4.41	3	18.89	0.19
2436	2018	6	C39	0	5.26	3	18.89	0.19
2439	2015	1	I65	0	9.76	1	18.16	0.1
2439	2016	2	I65	0	8.59	2	18.16	0.1
2439	2017	3	I65	0	11.76	3	17.48	0.1
2439	2018	4	I65	0	12.21	4	17.36	0.1
2444	2017	26	C33	1	7.41	3	19.47	0.23

2444	2018	11 C33	1	8.55	12	18.1	0.19
2444	2019	3 C33	1	8.85	12	18.1	0.19
2445	2018	11 C33	1	8.55	12	18.1	0.19
2445	2019	3 C33	1	8.85	12	18.1	0.19
2448	2014	5 C36	1	7.51	2	19.89	0.19
2448	2015	5 C36	1	7.59	2	19.26	0.19
2448	2016	5 C36	1	8.01	3	20.35	0.19
2448	2017	5 C36	1	8.46	3	20.35	0.2
2448	2018	5 C36	1	7.03	3	20.35	0.2
2448	2019	5 C36	1	2.03	3	20.88	0.2
2454	2016	3 C36	1	5.59	1	16.64	0.17
2454	2017	3 C36	1	6.96	1	18.87	0.17
2454	2018	3 C36	1	3.26	2	17.09	0.17
2454	2019	3 C36	1	3.2	2	17.55	0.18
2456	2015	23 C39	1	3.17	3	17.37	0.17
2456	2016	35 C39	1	3.63	4	17.37	0.19
2456	2017	45 C39	1	3.03	4	17.37	0.18
2456	2018	38 C39	1	-1.54	4	17.37	0.19
2457	2015	45 C39	1	3.17	3	17.37	0.17
2457	2016	35 C39	1	3.63	4	17.37	0.19
2457	2017	45 C39	1	3.03	4	17.33	0.18
2457	2018	38 C39	1	-1.54	4	17.24	0.19
2475	2015	2 C39	0	10.64	5	17.34	0.17
2475	2016	2 C39	0	7.26	6	17.34	0.18
2475	2017	2 C39	0	7.31	7	16.88	0.18
2475	2018	4 C39	0	8.89	7	17.31	0.2
2475	2019	7 C39	0	11.48	9	17.31	0.2
2476	2015	2 C39	1	10.64	5	17.34	0.17
2476	2016	2 C39	1	7.26	6	17.34	0.18
2476	2017	2 C39	1	7.31	7	17.34	0.18
2476	2018	4 C39	1	8.89	7	17.34	0.2
2476	2019	7 C39	1	11.48	9	17.34	0.2
2477	2016	2 C39	1	7.26	6	17.65	0.18
2477	2017	2 C39	1	7.31	7	17.88	0.18
2477	2018	4 C39	1	8.89	7	19.61	0.2
2477	2019	7 C39	1	11.48	9	19.61	0.2
2478	2019	7 C39	1	11.48	9	19.53	0.2
2479	2019	7 C39	1	11.48	9	19.53	0.2
2480	2015	1 C34	1	-3.57	2	14.91	0.1
2480	2017	2 C34	1	0.36	2	16.61	0.1
2480	2018	3 C34	1	0.44	2	16.94	0.1
2483	2018	1 C34	0	2.04	7	17.86	0.17
2483	2019	1 C34	0	2.4	7	17.95	0.17
2484	2019	1 C34	1	2.4	7	17.95	0.17
2486	2012	1 C18	1	3.38	2	15.5	0.1
2486	2013	1 C18	1	8.41	2	15.51	0.1
2486	2014	2 C18	1	1.63	3	18.07	0.13
2486	2015	1 C18	1	-6.78	3	18.07	0.13
2486	2016	1 C18	1	0.13	3	18.07	0.1
2486	2017	1 C18	1	1.15	3	18.18	0.1
2486	2018	2 C18	1	1.37	3	15.5	0.1

2486	2019	2	C18	1	-1.51	3	15.5	0.1
2487	2014	2	C18	0	1.63	3	18.07	0.13
2487	2015	1	C18	0	-6.78	3	18.11	0.13
2487	2016	1	C18	0	0.13	3	18.14	0.1
2488	2014	4	C36	1	2.05	2	19.44	0.19
2488	2015	11	C36	1	1.2	2	19.37	0.19
2488	2016	9	C36	1	-3.52	2	19.48	0.2
2488	2017	12	C36	1	0.95	1	19.86	0.19
2488	2018	9	C36	1	2.24	2	20.61	0.21
2489	2014	2	C18	0	1.63	3	18.07	0.13
2489	2015	1	C18	0	-6.78	3	18.11	0.13
2489	2017	1	C18	0	1.15	3	18.18	0.1
2489	2018	2	C18	0	1.37	3	18.18	0.1
2489	2019	2	C18	0	-1.51	3	18.1	0.1
2494	2013	1	C19	1	6.47	3	14.84	0.2
2494	2014	1	C19	1	5.4	3	14.9	0.23
2494	2015	11	C19	1	0.6	3	14.9	0.23
2494	2016	1	C19	1	0.42	3	14.9	0.23
2494	2017	1	C19	1	-3.04	3	14.9	0.23
2494	2018	1	C19	1	0.5	3	14.86	0.23
2494	2019	1	C19	1	0.48	3	14.54	0.27
2506	2016	1	C39	1	-0.19	4	21.14	0.19
2506	2017	7	C39	1	0.18	5	21.01	0.21
2506	2018	4	C39	1	0.29	6	20.17	0.21
2506	2019	7	C39	1	0.4	7	21.14	0.22
2507	2016	1	C39	1	-0.19	4	22.14	0.19
2507	2017	7	C39	1	0.18	5	22.04	0.21
2507	2018	4	C39	1	0.29	6	22.01	0.21
2507	2019	7	C39	1	0.4	7	22.01	0.22
2508	2018	4	C39	1	0.29	6	21.17	0.21
2508	2019	7	C39	1	0.4	7	21.17	0.22
2517	2015	25	I64	1	59.81	3	17.09	0.13
2518	2018	1	C38	1	6.29	4	16.19	0.16
2518	2019	2	C38	1	8.47	5	17.03	0.17
2519	2015	25	I64	1	59.81	3	17.09	0.13
2519	2016	25	I64	1	24.97	2	17.09	0.15
2519	2017	35	I64	1	33.17	2	17.09	0.15
2519	2018	18	I64	1	5.57	2	17.09	0.2
2519	2019	16	I64	1	-36.94	2	17.09	0.2
2520	2019	1	C34	1	3.06	9	15.12	0.22
2521	2019	1	C38	1	8.47	5	17.03	0.17
2527	2019	1	C38	1	0.93	3	19.61	0.2
2528	2016	98	C39	1	-12	5	21.07	0.21
2528	2017	160	C39	1	2.99	6	21.57	0.22
2529	2017	160	C39	1	2.99	6	21.57	0.22
2530	2017	10	I64	0	5.31	1	18.42	0.1
2532	2016	1	C32	1	5.47	3	15.31	0.13
2532	2017	2	C32	1	6.23	3	15.35	0.13
2532	2018	3	C32	1	6.65	3	15.41	0.13
2532	2019	4	C32	1	6.8	3	15.45	0.13
2533	2017	5	C38	0	4.03	1	18.31	0.18

2533	2018	6	C38	0	3.63	1	18.15	0.18
2539	2014	38	C26	1	3.05	1	10.04	0.1
2539	2016	33	C26	1	1.73	5	10.04	0.17
2539	2017	15	C26	1	1.12	4	10.04	0.14
2540	2016	33	C26	1	1.73	3	15.86	0.16
2540	2017	15	C26	1	1.12	5	16.56	0.17
2540	2018	18	C26	1	1.71	4	16.36	0.14
2540	2019	9	C26	1	2.18	4	17.36	0.14
2541	2017	15	C26	1	1.12	5	17.04	0.17
2541	2018	18	C26	1	1.71	4	17.61	0.14
2541	2019	9	C26	1	2.18	4	17.61	0.14
2543	2019	1	C38	1	8.85	2	19.93	0.2
2544	2016	30	I65	1	2.88	2	16.08	0.16
2544	2017	33	I65	1	4.19	3	18.84	0.19
2544	2018	20	I65	1	0.38	3	19.36	0.19
2544	2019	21	I65	1	0.69	3	19.42	0.19
2545	2016	1	C33	1	1.84	2	17.27	0.15
2545	2017	1	C33	1	2.3	2	17.27	0.15
2545	2018	5	C33	1	3.97	2	17.7	0.15
2545	2019	5	C33	1	2.6	2	17.86	0.15
2546	2016	30	I65	0	2.88	2	16.08	0.16
2546	2017	33	I65	0	4.19	3	18.84	0.19
2546	2018	20	I65	0	0.38	3	19.36	0.19
2546	2019	21	I65	0	0.69	3	19.42	0.19
2547	2013	4	C33	1	1.93	5	11.29	0.1
2547	2017	7	C33	1	-5.28	8	18.3	0.16
2548	2017	33	I65	0	4.19	3	18.84	0.19
2548	2018	20	I65	0	0.38	3	19.36	0.19
2548	2019	21	I65	0	0.69	3	19.42	0.19
2549	2018	7	C33	1	0.93	8	17.67	0.15
2549	2019	5	C33	1	0.38	9	17.7	0.17
2550	2017	18	C33	1	-5.28	8	18.3	0.16
2550	2018	7	C33	1	0.93	8	18.97	0.15
2550	2019	5	C33	1	0.38	9	19.3	0.17
2551	2019	5	C33	1	0.38	9	17.7	0.17
2554	2012	1	B11	1	6.62	2	15.58	0.1
2554	2016	3	B11	1	3.41	9	18.45	0.13
2554	2017	3	B11	1	2.08	10	18.2	0.14
2554	2018	4	B11	1	-11.58	9	16.57	0.17
2554	2019	1	B11	1	2.36	8	16.73	0.14
2555	2014	1	I64	0	2.78	4	15.26	0.18
2555	2015	3	I64	0	22.93	6	15.26	0.18
2555	2016	2	I64	0	22.1	6	15.26	0.2
2555	2017	3	I64	0	23.63	5	15.26	0.22
2555	2018	3	I64	0	13.12	5	15.26	0.18
2555	2019	3	I64	0	26.07	7	15.26	0.26
2556	2017	3	B11	1	2.08	10	18.2	0.14
2556	2018	4	B11	1	-11.58	9	16.57	0.17
2556	2019	1	B11	1	2.36	8	16.73	0.14
2557	2014	9	C13	0	7.17	2	17.91	0.1
2557	2017	4	C13	1	7.23	2	17.3	0.1

2557	2018	7	C13	1	9.01	2	17.18	0.1
2557	2019	6	C13	1	11.45	2	18.17	0.1
2558	2016	38	I64	1	24.25	4	17.52	0.15
2558	2017	102	I64	1	12.74	4	16.39	0.15
2558	2018	62	I64	1	9.87	3	16.02	0.3
2558	2019	5	I64	1	8.28	5	16.02	0.22
2559	2019	1	C34	1	3.47	2	18.85	0.15
2560	2014	1	I64	0	2.78	4	15.26	0.18
2560	2015	3	I64	0	22.93	6	15.26	0.18
2560	2016	2	I64	0	22.1	6	15.26	0.2
2561	2015	3	I64	1	22.93	6	17.16	0.18
2561	2016	2	I64	1	22.1	6	17.16	0.2
2561	2017	3	I64	1	23.63	5	17.14	0.22
2561	2018	3	I64	1	13.12	5	17.05	0.18
2561	2019	3	I64	1	26.07	7	17.05	0.26
2562	2019	1	C34	0	3.47	2	18.85	0.15
2563	2018	2	E48	0	0.77	5	17.42	0.1
2564	2017	2	E48	1	1.62	4	17.03	0.1
2564	2018	2	E48	1	0.77	5	17.42	0.1
2564	2019	2	E48	0	0.48	4	18.04	0.1
2565	2019	1	C22	1	-5.8	3	18.29	0.17
2571	2018	6	C30	0	-7.04	1	19.29	0.1
2571	2019	5	C30	0	0.71	1	18.41	0.1
2573	2015	1	N77	1	9.12	1	17.75	0.1
2573	2016	2	N77	1	8.48	1	18.45	0.1
2573	2017	3	N77	1	5.64	1	18.42	0.1
2573	2018	4	N77	1	4.84	1	20.23	0.1
2573	2019	5	N77	1	3.65	1	20.19	0.1
2577	2015	5	C39	1	-14.43	4	17.47	0.17
2577	2016	7	C39	1	0.08	4	17.52	0.17
2590	2019	1	C36	1	3.62	1	18.77	0.19
2594	2011	2	C36	1	2.69	11	17.77	0.16
2594	2017	3	C36	1	3.04	6	20.05	0.13
2594	2018	8	C36	1	1.91	6	20.02	0.13
2595	2015	5	C35	0	19.99	1	17.11	0.17
2595	2016	6	C35	0	19.23	2	18.1	0.18
2595	2017	7	C35	0	15.85	4	18.07	0.19
2595	2018	8	C35	0	14.53	3	19.38	0.19
2595	2019	9	C35	0	13.96	4	19.52	0.2
2596	2011	2	C36	0	2.69	11	19.77	0.16
2596	2012	1	C36	0	0.32	6	18.88	0.15
2596	2013	2	C36	0	1.07	6	18.02	0.15
2596	2014	2	C36	0	0.87	6	18.02	0.15
2596	2015	5	C36	0	3	6	19.57	0.15
2596	2016	5	C36	0	4.21	3	20.43	0.17
2596	2017	3	C36	0	3.04	6	19.65	0.13
2596	2018	8	C36	0	1.91	6	20.2	0.13
2596	2019	5	C36	0	1.09	3	21.03	0.17
2597	2011	2	C36	0	2.69	11	17.77	0.16
2598	2011	2	C36	0	2.69	11	20.77	0.16
2598	2012	1	C36	0	0.32	6	18.88	0.15

2598	2013	2	C36	0	1.07	6	18.88	0.15
2598	2014	2	C36	0	0.87	6	19.28	0.15
2598	2015	5	C36	0	3	6	19.57	0.15
2599	2016	5	C35	1	19.23	2	18.1	0.18
2599	2017	6	C35	1	15.85	4	17.49	0.19
2600	2017	7	C35	1	15.85	4	19.49	0.19
2600	2018	8	C35	1	14.53	3	19.38	0.19
2600	2019	9	C35	1	13.96	4	20.52	0.2
2601	2019	10	C35	1	13.96	4	19.52	0.2
2603	2016	3	C27	0	8.96	3	20.96	0.21
2603	2017	2	C27	0	7.59	3	20.96	0.18
2610	2018	4	C38	1	0.89	3	21.45	0.21
2610	2019	4	C38	1	-12.99	3	21.45	0.21
2611	2018	4	C38	1	-12.99	3	21.45	0.21
2612	2014	8	C18	0	4.46	2	18.85	0.15
2612	2015	8	C18	0	2.62	2	18.85	0.25
2612	2016	4	C18	0	5.1	2	18.85	0.4
2612	2017	2	C18	0	3.4	2	18.85	0.4
2612	2018	6	C18	0	2.87	2	18.85	0.45
2612	2019	5	C18	0	0.65	2	18.85	0.6
2613	2015	8	C18	1	2.62	2	19.78	0.25
2613	2016	4	C18	1	5.1	2	19.78	0.4
2613	2017	2	C18	1	3.4	2	19.78	0.4
2613	2018	6	C18	1	2.87	2	19.78	0.45
2613	2019	5	C18	1	0.65	2	19.78	0.6
2614	2012	2	C38	0	1.01	2	17.03	0.17
2614	2015	2	C38	0	5.02	4	17.11	0.16
2614	2016	4	C38	0	6.38	4	17.11	0.17
2614	2017	8	C38	0	7.69	10	17.04	0.2
2614	2018	4	C38	0	8.19	6	17.03	0.18
2614	2019	6	C38	0	4.73	10	17.03	0.21
2615	2015	2	C38	1	5.02	4	16.11	0.16
2615	2016	4	C38	1	6.38	4	16.11	0.17
2615	2017	8	C38	1	7.69	10	20.01	0.2
2615	2018	4	C38	1	8.19	6	20.14	0.18
2615	2019	6	C38	1	4.73	10	20.14	0.21
2616	2017	8	C38	1	7.69	10	16.11	0.2
2616	2018	4	C38	1	8.19	6	16.11	0.18
2616	2019	6	C38	1	4.73	10	20.13	0.21
2617	2019	6	C38	1	4.73	10	20.63	0.21
2626	2016	1	C14	0	10.22	4	15.75	0.3
2626	2017	14	C14	0	14.47	4	16.59	0.33
2626	2018	11	C14	0	17.35	4	16.48	0.43
2626	2019	19	C14	0	9.78	4	16.48	0.5
2630	2013	1	C34	1	2.58	1	18.13	0.1
2630	2014	1	C34	1	2.41	1	18.13	0.1
2630	2015	1	C34	1	2.5	2	19.94	0.15
2630	2016	3	C34	1	2.07	2	20.13	0.15
2630	2017	2	C34	1	1.56	2	20.06	0.15
2630	2018	3	C34	1	-1	2	19.91	0.15
2630	2019	2	C34	1	0.24	2	19.94	0.15

2635	2017	5 C39	1	6.49	1	14.51	0.15
2635	2018	7 C39	1	6.22	2	19.42	0.19
2635	2019	5 C39	1	-8.27	2	19.47	0.19
2637	2016	1 C26	0	5.5	1	19.52	0.2
2637	2017	2 C26	0	2.81	1	20.15	0.19
2637	2018	3 C26	0	3.12	1	18.31	0.19
2637	2019	4 I65	0	5.69	1	18.8	0.19
2639	2016	1 C34	0	1.02	3	16.09	0.13
2639	2017	2 C34	0	-1.87	3	16.72	0.13
2639	2018	2 C34	0	0.39	3	16.55	0.13
2639	2019	4 C34	0	1.21	4	16.8	0.13
2649	2013	17 I65	1	8.09	4	16.19	0.16
2649	2014	17 I65	1	8.68	5	16.19	0.17
2649	2015	25 I65	1	8.09	7	17.4	0.17
2649	2016	28 I65	1	4.59	7	18	0.18
2649	2017	38 I65	1	7.91	7	17.51	0.17
2649	2018	49 I65	1	7.24	7	17.36	0.18
2650	2013	17 I65	1	8.09	4	17.19	0.16
2650	2014	17 I65	1	8.68	5	17.6	0.17
2650	2015	25 I65	1	8.09	7	17.6	0.17
2650	2016	28 I65	1	4.59	7	17.6	0.18
2650	2017	38 I65	1	7.91	7	17.12	0.17
2650	2018	49 I65	1	7.24	7	17.06	0.18
2659	2017	38 P82	0	0.56	3	11.11	0.1
2661	2018	1 C14	1	5.32	2	16.75	0.1
2661	2019	2 C14	1	5.12	2	16.79	0.2
2675	2016	1 C27	1	5.88	2	17.2	0.17
2675	2017	2 C27	1	5.55	2	20.52	0.21
2678	2019	1 C24	1	4.23	4	16.92	0.15
2682	2018	2 G54	1	2.93	3	16.58	0.1
2682	2019	3 G54	1	1.79	3	16.69	0.1
2683	2019	1 B11	1	5.2	3	17.09	0.1
2684	2019	1 B11	1	5.2	3	17.09	0.1
2689	2012	1 C34	1	5.83	5	10.56	0.14
2689	2018	6 C34	1	0.34	10	10.61	0.14
2689	2019	10 C34	1	-4.84	10	11.38	0.12
2690	2018	6 C34	1	0.34	10	9.61	0.14
2690	2019	10 C34	1	-4.84	10	9.68	0.12
2691	2018	6 C34	1	0.34	10	9.62	0.14
2691	2019	10 C34	1	-4.84	10	9.98	0.12
2699	2017	11 C34	1	4.41	4	17.8	0.18
2705	2019	1 C38	0	9.01	1	16.95	0.17
2714	2018	2 A03	1	1.96	3	17.61	0.1
2714	2019	3 A03	1	15.32	4	17.61	0.18
2715	2019	3 A03	1	15.32	4	17.46	0.18
2717	2017	2 N77	1	6.37	2	19.58	0.15
2717	2018	1 N77	1	5.85	3	19.58	0.13
2717	2019	1 N77	1	1.96	2	19.58	0.15
2725	2017	1 C36	1	1.48	2	18.01	0.18
2725	2018	2 C36	1	4.02	2	18	0.18
2726	2017	1 C36	1	1.48	2	18.01	0.18

2726	2018	2	C36	1	4.02	2	18.01	0.18
2733	2015	2	C38	1	6.44	3	18.29	0.18
2733	2016	3	C38	1	4.44	5	18.29	0.2
2733	2017	3	C38	1	0.99	5	19.66	0.2
2733	2018	3	C38	1	2.27	5	19.73	0.2
2733	2019	2	C38	1	3.83	5	19.73	0.2
2738	2015	1	C32	1	5.71	8	13.68	0.13
2738	2016	2	C32	1	5.99	8	12.07	0.15
2738	2019	2	C32	1	3.77	9	13.99	0.19
2739	2019	1	R86	1	-19.04	7	16.44	0.96
2745	2017	9	C39	1	4.12	2	20.83	0.21
2745	2018	7	C39	1	2.86	3	20.77	0.22
2745	2019	26	C39	1	1.5	3	21.79	0.22
2747	2015	10	C40	1	8.69	2	14.7	0.15
2747	2016	11	C40	1	5.81	2	14.85	0.14
2747	2017	30	C40	1	3.98	8	17.73	0.17
2747	2018	25	C40	1	3.33	8	17.78	0.17
2748	2015	10	C40	1	8.69	2	14.7	0.15
2748	2016	11	C40	1	5.81	2	15.35	0.14
2748	2017	30	C40	1	3.98	8	17.33	0.17
2748	2018	25	C40	1	3.33	8	14.6	0.17
2748	2019	29	C40	1	2.38	7	15.35	0.17
2749	2017	30	C40	1	3.98	8	14.69	0.17
2749	2018	25	C40	1	3.33	8	13.48	0.17
2749	2019	29	C40	1	2.38	7	17.23	0.17
2752	2019	1	C33	1	1.09	2	17.64	0.15
2761	2019	1	E48	1	2.25	4	18.26	0.13
2773	2018	2	C27	1	14.21	2	19.6	0.2
2773	2019	3	C27	1	13.03	2	19.67	0.2
2781	2017	53	E50	1	3.52	2	18.01	0.1
2781	2018	40	E50	1	3.44	2	18.13	0.1
2781	2019	30	E50	1	1.55	2	18.48	0.1
2782	2019	1	C39	1	1.95	2	19.09	0.19
2786	2019	6	C35	0	-6.51	5	14.26	0.12
2791	2016	9	C33	1	10.02	5	15.37	0.1
2791	2017	9	C33	1	5.91	6	16.06	0.1
2791	2018	12	C33	1	4.52	6	16.48	0.1
2791	2019	9	C33	1	9.79	6	16.94	0.1
2792	2019	7	C39	1	1.02	5	19.63	0.2
2793	2016	9	C33	0	10.02	5	15.37	0.1
2793	2017	9	C33	0	5.91	6	15.06	0.1
2793	2018	12	C33	0	4.52	6	14.48	0.1
2793	2019	9	C33	0	9.79	6	15.37	0.1
2794	2016	9	C33	1	10.02	5	15.37	0.1
2794	2017	9	C33	1	5.91	6	15.46	0.1
2794	2018	12	C33	1	4.52	6	16.48	0.1
2794	2019	9	C33	1	9.79	6	16.48	0.1
2795	2017	9	C33	1	5.91	6	15.06	0.1
2795	2018	12	C33	1	4.52	6	14.48	0.1
2795	2019	9	C33	1	9.79	6	16.94	0.1
2796	2019	7	C39	1	1.02	5	19.63	0.2

2811	2018	1	E50	1	4.26	5	11.28	0.16
2811	2019	2	E50	1	4.27	5	11.28	0.18
2823	2018	2	C38	1	4.72	2	19.09	0.19
2828	2016	2	B11	1	6	1	8.41	0.1
2828	2018	2	B11	1	2.76	3	8.41	0.1
2828	2019	1	B11	1	1.98	3	6.19	0.1
2831	2017	10	C22	1	11.33	4	16.48	0.15
2832	2017	10	C22	1	11.33	4	16.48	0.15
2848	2016	8	C39	1	5.43	2	16	0.16
2848	2017	73	C39	1	1.21	2	16.9	0.17
2848	2018	43	C39	1	-5.97	3	17.91	0.18
2848	2019	39	C39	1	0.91	2	16.91	0.17
2849	2016	8	C39	0	5.43	2	16	0.16
2849	2017	73	C39	0	1.21	2	16.9	0.17
2849	2018	43	C39	0	-5.97	3	17.91	0.18
2849	2019	39	C39	0	0.91	2	16.91	0.17
2851	2019	1	C38	1	10.24	5	18.74	0.19
2852	2019	1	C38	1	10.24	5	18.74	0.19
2855	2017	6	C39	1	2.73	3	14.51	0.15
2855	2018	6	C39	1	1.14	3	14.51	0.14
2855	2019	9	C39	1	1.44	2	14.51	0.14
2856	2017	6	C39	1	2.73	3	14.51	0.15
2856	2018	6	C39	1	1.14	3	14.55	0.14
2856	2019	9	C39	1	1.44	2	14.55	0.14
2857	2017	6	C39	1	2.73	3	15.51	0.15
2857	2018	6	C39	1	1.14	3	15.45	0.14
2889	2019	9	L72	1	1.58	2	16.54	0.25
2891	2019	1	C13	0	5.75	2	17.34	0.25
2901	2018	2	C35	0	24.76	2	15.14	0.15
2901	2019	3	C35	0	24.52	1	16.09	0.14
2920	2017	2	C36	1	11.51	2	17.13	0.17
2920	2019	3	C36	1	4.86	3	17.31	0.17
2925	2019	82	C39	0	5.72	4	17.23	0.17
2941	2018	1	E48	1	3.82	2	12.04	0.1
2941	2019	2	E48	1	1.74	2	10.52	0.1
2942	2018	3	E48	0	3.82	2	12.04	0.1
2942	2019	4	E48	0	1.74	2	10.52	0.1
300001	2016	3	C38	1	2.14	11	16.71	0.13
300001	2017	4	C38	1	1.87	13	17.06	0.12
300001	2018	5	C38	1	1.46	13	18.23	0.14
300001	2019	6	C38	1	1.73	13	18.37	0.14
300003	2014	65	C38	1	12.46	7	17.91	0.14
300003	2015	86	C38	1	5.13	9	17.91	0.17
300003	2016	177	C38	1	7.25	7	17.91	0.19
300003	2017	327	C38	1	1.39	7	17.91	0.2
300006	2013	54	C38	0	13.4	3	17.74	0.13
300006	2014	65	C38	0	13.4	7	17.74	0.14
300006	2015	86	C38	0	5.13	9	19.09	0.17
300006	2016	177	C38	0	7.25	7	19.09	0.19
300006	2017	327	C38	0	1.39	7	19.09	0.2
300012	2017	1	M74	1	4.15	6	15.33	0.13

300012	2018	2	M74	1	7.46	5	16.76	0.1
300013	2019	16	G59	1	-25.19	3	17.06	0.1
300015	2019	1	Q83	1	13.3	8	11.7	0.4
300017	2017	94	I65	1	8.63	12	16.12	0.16
300017	2018	72	I65	1	7.18	12	16.12	0.17
300017	2019	62	I65	1	0.33	12	16.23	0.18
300026	2017	2	C27	1	6.08	5	14.39	0.1
300026	2018	5	C27	1	2.9	5	15.02	0.12
300026	2019	7	C27	1	5.13	7	15.2	0.1
300027	2014	17	R86	1	12.15	15	17.77	0.19
300027	2015	9	R86	1	8.79	15	18.91	0.2
300027	2016	11	R86	1	5.27	15	19.3	0.26
300027	2017	12	R86	1	4.93	16	19.22	0.33
300027	2018	15	R86	1	-5.11	17	18.52	0.33
300031	2019	9	I64	1	7.89	5	14.48	0.24
300036	2010	27	I65	0	7.55	2	15.62	0.1
300036	2011	26	I65	0	6.92	2	15.62	0.1
300036	2012	12	I65	0	0.68	2	15.02	0.1
300036	2013	20	I65	0	7	2	15.59	0.1
300036	2014	18	I65	0	5.45	2	15.62	0.1
300036	2015	16	I65	0	6.13	2	15.62	0.1
300036	2016	6	I65	0	7.78	2	15.62	0.1
300036	2017	13	I65	0	7.84	2	15.04	0.1
300036	2018	27	I65	0	5.73	2	15.02	0.1
300036	2019	52	I65	0	7.27	2	15.02	0.1
300037	2018	3	C26	0	8.12	2		0.01
300041	2019	3	C26	1	6.21	1	18.47	0.18
300043	2019	1	I64	1	4.45	4	18.56	0.1
300052	2014	2	I64	1	0.44	5	15.48	0.12
300052	2015	3	I64	1	4.49	5	15.48	0.12
300052	2016	4	I64	1	-5.56	5	15.48	0.12
300052	2017	5	I64	1	4.38	5	15.48	0.12
300058	2017	90	L72	1	1.49	4	18.99	0.15
300058	2018	56	L72	1	2.45	4	18.93	0.15
300068	2017	13	C38	1	4.17	5	20.55	0.21
300068	2018	11	C38	1	1.03	5	20	0.21
300068	2019	8	C38	1	2	4	20.99	0.2
300072	2018	1	C26	0	2.31	2	18.49	0.18
300088	2016	4	C39	1	5.87	1	20.54	0.21
300088	2017	2	C39	1	7.44	1	20.73	0.21
300088	2018	18	C39	1	8.63	1	20.46	0.2
300088	2019	26	C39	1	9.37	1	20.59	0.21
300109	2015	1	C26	0	6.66	2	17.22	0.17
300109	2016	1	C26	0	6.3	3	17.22	0.2
300115	2015	7	C39	1	10.1	2	20.29	0.2
300115	2016	5	C39	1	10.55	2	20.29	0.21
300115	2017	1	C39	1	6.45	2	20.29	0.2
300115	2018	14	C39	1	0.09	2	20.29	0.2
300115	2019	26	C39	1	1.22	2	20.29	0.2
300118	2017	8	C38	1	5.2	5	19.61	0.2
300118	2018	18	C38	1	1.29	5	18.83	0.2

300118	2019	11	C38	1	4.41	5	18.97	0.2
300124	2015	23	C38	1	15.71	2	14.91	0.15
300124	2016	14	C38	1	14.08	2	19.42	0.19
300124	2017	27	C38	1	12.82	2	19.12	0.19
300124	2018	34	C38	1	12.48	4	14.81	0.2
300124	2019	27	C38	1	8.01	5	19.42	0.21
300128	2017	1	C39	1	2.11	4	17.67	0.13
300136	2014	1	C39	1	6.84	3	17.32	0.17
300136	2015	4	C39	1	14.79	3	17.32	0.17
300136	2017	12	C39	1	20.96	4	17.32	0.19
300136	2018	2	C39	1	15.78	4	17.32	0.19
300136	2019	3	C39	1	13.18	5	17.32	0.21
300138	2018	17	C13	1	4.57	3	12.58	0.17
300138	2019	16	C13	1	4.83	4	16.89	0.2
300144	2019	1	R87	1	12.49	3	20.87	0.17
300150	2019	1	I65	1	4.48	1	18.47	0.18
300151	2016	4	C35	1	3.29	1	19.97	0.2
300157	2017	5	B11	0	-6.96	7	18.42	0.24
300157	2018	1	B11	0	0.73	7	18.25	0.26
300157	2019	7	B11	0	-23.8	7	17.76	0.21
300169	2017	4	C29	1	-1.83	3	16.73	0.23
300169	2018	6	C29	1	0.76	3	16.2	0.23
300169	2019	5	C29	1	-14.84	3	16.2	0.23
300170	2013	10	I65	1	10.55	1	13.82	0.14
300170	2017	7	I65	1	10.54	2	12.85	0.14
300170	2018	17	I65	1	11.15	2	12.49	0.14
300170	2019	16	I65	1	2.16	4	12.49	0.14
300171	2013	1	C35	1	8	2	16.81	0.17
300171	2014	2	C35	1	8.84	2	16.81	0.17
300171	2015	2	C35	1	9.6	2	18.73	0.19
300171	2016	1	C35	1	5.89	2	18.9	0.19
300171	2017	9	C35	1	3.18	2	18.83	0.19
300171	2018	6	C35	1	1.93	3	18.72	0.19
300171	2019	1	C35	1	3.03	3	18.9	0.18
300177	2016	6	C39	1	0.94	2	18.49	0.18
300177	2017	2	C39	1	3.83	5	19.13	0.19
300177	2018	2	C39	1	5.46	6	19.54	0.2
300177	2019	3	C39	1	-5.39	6	19.54	0.19
300190	2016	4	N77	1	2.59	2	17.19	0.2
300190	2018	1	N77	1	3.7	3	17.34	0.13
300190	2019	4	N77	1	4.2	3	18.85	0.13
300195	2012	4	C35	0	10.39	2	19.32	0.15
300195	2013	3	C35	0	9.74	2	19.72	0.15
300195	2015	1	C35	0	5.4	3	19.92	0.2
300195	2016	2	C35	0	3.65	4	19.92	0.2
300195	2017	1	C35	0	3.02	4	19.18	0.2
300195	2018	1	C35	0	1.48	4	19.32	0.2
300195	2019	2	C35	0	-12.49	4	19.32	0.21
300196	2016	6	C35	1	3.65	4	19.95	0.2
300196	2017	4	C35	1	3.02	4	19.78	0.2
300196	2018	14	C35	1	1.48	4	19.93	0.2

300196	2019	13	C35	1	-12.49	4	20.67	0.21
300206	2016	21	C35	1	1.33	4	16.46	0.16
300206	2017	16	C35	1	2.62	6	16.32	0.17
300206	2018	27	C35	1	6.01	4	16.3	0.16
300206	2019	1	C35	1	8.67	5	16.46	0.15
300207	2016	2	C38	1	6.59	2	17.87	0.18
300207	2017	1	C38	1	5.28	2	17.8	0.18
300207	2018	2	C38	1	4.45	2	17.49	0.18
300207	2019	2	C38	1	3.55	2	17.87	0.2
300208	2018	10	C38	0	4.15	4	16.8	0.17
300209	2015	1	I65	0	-0.09	1	13.42	0.13
300210	2018	5	C38	1	4.15	4	16.8	0.17
300222	2016	3	C38	1	8.01	1	17.39	0.17
300222	2018	5	C38	1	5.87	4	16.44	0.18
300224	2017	6	C30	1	2.7	2	14.85	0.1
300227	2016	8	C39	0	2.12	1	14.44	0.14
300227	2017	8	C39	0	6.7	1	14.44	0.16
300230	2015	2	C29	0	8.13	8	15.04	0.16
300230	2016	2	C29	0	5.73	12	14.35	0.23
300230	2017	1	C29	0	7.5	12	14.85	0.25
300230	2018	1	C29	0	9.24	12	15.04	0.28
300230	2019	1	C29	0	6.06	11	16.2	0.3
300232	2019	2	C39	0	7.72	3	19.31	0.19
300233	2016	2	C29	1	5.73	12	14.35	0.23
300233	2017	2	C29	1	7.5	12	14.85	0.25
300233	2018	3	C29	1	9.24	12	15.03	0.28
300233	2019	1	C29	1	6.06	11	14.2	0.3
300240	2019	5	G59	1	2.02	3	17.45	0.27
300244	2018	11	Q83	0	6.52	2	19.93	0.1
300252	2016	11	C38	1	6.79	4	19.47	0.18
300252	2017	8	C38	1	3.26	4	19.15	0.19
300252	2018	5	C38	1	2.83	5	19.02	0.2
300252	2019	4	C38	1	1.75	4	19.47	0.2
300253	2016	4	C38	0	6.79	4	17.74	0.18
300253	2017	4	C38	0	3.26	4	19.15	0.19
300253	2018	4	C38	0	2.83	5	19.82	0.2
300253	2019	5	C38	0	1.75	4	20.31	0.2
300257	2019	4	C34	1	1.94	8	16.12	0.18
300260	2015	1	C34	1	-0.22	4	12.86	0.13
300262	2017	1	N77	1	3.25	7	17.59	0.1
300262	2018	2	N77	1	2.34	7	17.59	0.1
300262	2019	3	N77	1	1.4	8	17.59	0.1
300265	2016	2	C38	1	7.36	1	18.43	0.17
300265	2017	5	C38	1	2.2	1	18.29	0.19
300265	2018	3	C38	1	1.88	1	18.15	0.19
300265	2019	3	C38	1	1.41	1	18.43	0.19
300267	2016	3	C27	1	15.32	1	19.3	0.19
300267	2017	3	C27	1	9.24	1	19.3	0.18
300274	2018	1	C38	1	4.71	15	18.63	0.19
300274	2019	4	C38	1	4.41	17	18.63	0.19
300275	2015	7	C38	1	7.31	6	17.61	0.18

300275	2016	1	C38	1	5.92	8	17.72	0.18
300275	2017	5	C38	1	7.27	9	18	0.18
300278	2016	1	C38	1	5.92	8	17.72	0.18
300278	2017	3	C38	1	7.27	9	18	0.18
300278	2018	4	C38	1	4.71	15	18.63	0.19
300278	2019	2	C38	1	4.41	17	18.95	0.19
300279	2014	1	C38	1	2.9	1	16.44	0.16
300285	2016	1	M74	1	5.74	13	14.75	0.24
300285	2017	1	M74	1	5.1	15	14.57	0.21
300285	2018	3	M74	1	5.56	14	15.4	0.17
300285	2019	3	M74	1	5.75	14	15.4	0.16
300292	2018	2	I65	1	-38.81	1	18.19	0.18
300296	2015	1	C39	0	9.18	7	14.05	0.14
300296	2016	2	C39	0	9.87	7	14.05	0.18
300296	2017	2	C39	0	11.6	8	14.05	0.18
300296	2018	4	C39	0	9.28	8	14.05	0.19
300296	2019	13	C39	0	4.72	8	19.07	0.19
300297	2015	15	C39	1	9.18	7	14.05	0.14
300297	2016	6	C39	1	9.87	7	18.26	0.18
300297	2017	5	C39	1	11.6	8	18.41	0.18
300297	2018	6	C39	1	9.28	8	19.04	0.19
300297	2019	6	C39	1	4.72	8	19.07	0.19
300298	2015	8	C35	1	11.24	1	19.51	0.2
300298	2016	4	C35	1	8.16	2	20.03	0.2
300298	2017	9	C35	1	16.45	2	19.99	0.2
300298	2019	1	C35	1	7.98	2	20.03	0.2
300309	2014	1	J67	0	6.63	1	16.29	0.1
300309	2016	6	J67	1	-15.14	2	17.2	0.15
300309	2017	2	J67	1	4.63	4	17.2	0.15
300315	2013	1	I64	1	10.81	2	15.97	0.25
300315	2014	1	I64	1	10.08	3	15.97	0.23
300315	2015	1	I64	1	7.77	3	15.97	0.27
300315	2016	1	I64	1	6.1	4	15.97	0.28
300315	2017	3	I64	1	2.88	4	15.97	0.33
300315	2018	4	I64	1	-39.33	4	15.97	0.3
300315	2019	3	I64	1	6.08	4	15.97	0.3
300316	2017	4	C35	1	7.32	1	19.39	0.12
300316	2018	2	C35	1	9.2	1	19.49	0.19
300316	2019	2	C35	1	8.79	1	19.49	0.2
300318	2016	2	I64	0	6.1	4	19.51	0.28
300318	2017	1	I64	0	2.88	4	19.51	0.33
300318	2018	5	I64	0	-39.33	4	19.51	0.3
300318	2019	9	I64	0	6.08	4	19.51	0.3
300322	2015	4	C39	1	-10.08	2	17.76	0.18
300322	2016	5	C39	1	1.2	2	17.76	0.17
300322	2018	1	C39	1	3.7	2	17.76	0.15
300322	2019	1	C39	1	5.31	3	17.76	0.14
300323	2018	2	C39	0	2.14	2	14.39	0.14
300339	2016	8	I65	0	6.08	2	17.75	0.18
300339	2017	6	I65	0	4.7	2	16.48	0.16
300339	2018	3	I65	0	5.22	2	16.27	0.18

300339	2019	1	I65	0	-32.81	2	16.27	0.18
300347	2015	1	M73	1	11.72	9	14.41	0.14
300347	2016	2	M73	1	7.84	10	14.41	0.13
300347	2017	1	M73	1	11.13	11	14.41	0.14
300347	2018	2	M73	1	12.89	13	14.41	0.17
300347	2019	3	M73	1	16.51	13	14.41	0.25
300349	2018	7	M73	0	12.89	13	15.89	0.17
300349	2019	1	M73	0	16.51	13	15.95	0.25
300364	2018	4	R85	1	-54.19	4	17.99	0.18
300364	2019	9	R85	1	-27.27	3	17.99	0.23
300366	2018	7	I65	1	-11.55	1	16.58	0.17
300366	2019	7	I65	1	2.74	1	16.58	0.17
300369	2014	8	I65	1	14.76	4	16.09	0.16
300369	2015	10	I65	1	11.53	4	16.09	0.16
300369	2019	6	I65	1	5.72	3	16.02	0.17
300373	2017	10	C39	1	9.26	2	16.79	0.17
300373	2019	9	C39	1	6.31	2	16.79	0.17
300378	2015	4	I65	1	0.8	2	14.21	0.14
300378	2016	5	I65	1	2.55	2	14.21	0.14
300378	2017	3	I65	1	3.44	2	15.54	0.16
300378	2018	3	I65	1	3.65	2	17.94	0.18
300382	2018	3	C35	0	8.65	4	15.98	0.16
300382	2019	5	C35	0	5.07	4	15.98	0.17
300390	2017	5	C39	1	3.38	1	14.14	0.14
300390	2018	1	C39	1	4.92	1	14.11	0.18
300403	2019	2	C38	1	9.61	1	17.36	0.17
300415	2016	1	C35	1	6.99	2	19.2	0.19
300415	2017	2	C35	1	13.49	3	19.86	0.19
300415	2018	1	C35	1	7.2	4	19.25	0.19
300415	2019	4	C35	1	7.13	4	19.9	0.19
300418	2015	2	I64	1	15.13	8	18.1	0.18
300418	2016	4	I64	1	10.7	8	18.1	0.18
300418	2017	2	I64	1	15.45	8	18.09	0.19
300418	2018	2	I64	1	14.34	8	17.46	0.21
300418	2019	7	I64	1	14.53	8	17.46	0.24
300422	2017	4	N77	1	4.66	3	17.84	0.13
300422	2018	2	N77	1	4.43	3	18.49	0.13
300422	2019	5	N77	1	3.54	4	18.07	0.1
300423	2019	2	N77	1	3.54	4	18.07	0.1
300424	2018	5	C37	0	2.93	3	18.6	0.19
300424	2019	3	C37	0	2.85	10	18.6	0.19
300425	2019	4	C37	1	2.85	10	18.51	0.19
300428	2018	2	C36	1	10.84	3	18.44	0.18
300428	2019	1	C36	1	6.53	4	18.59	0.19
300429	2018	2	C26	1	8.65	1	17.8	0.18
300433	2017	4	C39	1	6.8	2	17.39	0.17
300433	2018	2	C39	1	1.52	2	17.65	0.17
300433	2019	1	C39	1	5.39	2	17.65	0.18
300438	2015	4	C38	0	7.87	1	14.32	0.14
300438	2016	3	C38	0	8.39	1	14.32	0.14
300438	2017	8	C38	0	8.99	1	13.66	0.18

300438	2018	3	C38	0	6.26	1	12.93	0.18
300438	2019	10	C38	0	3.47	1	12.93	0.18
300439	2016	7	C27	0	9.86	3	19.02	0.19
300439	2017	11	C27	0	8.01	3	18.82	0.19
300439	2018	3	C27	0	6.34	3	19.33	0.19
300439	2019	2	C27	0	-13.83	2	19.33	0.2
300454	2019	2	I65	1	12.75	4	17.2	0.17
300459	2018	1	I64	1	7.67	10	18.1	0.2
300459	2019	2	I64	1	-27.1	3	18.58	0.1
300464	2018	1	F52	1	0.18	5	15.07	0.14
300464	2019	2	F52	1	4.73	5	15.07	0.26
300468	2019	2	I65	1	7.92	1	17.69	0.18
300496	2015	3	I65	1	14.39	2	18.23	0.18
300496	2016	3	I65	1	7.74	3	18.23	0.18
300496	2017	3	I65	1	3.34	4	17.69	0.18
300496	2018	5	I65	1	6.34	6	17.61	0.18
300496	2019	4	I65	1	8.73	8	17.61	0.18
300507	2017	8	C36	0	10.6	2	17.85	0.18
300507	2018	30	C36	0	8.57	2	17.87	0.18
300507	2019	31	C36	0	7.59	2	18.9	0.18
300512	2018	6	C34	1	9.04	2	16.45	0.1
300512	2019	6	C34	1	5.41	2	16.47	0.1
300520	2016	3	I65	1	7.23	1	15.25	0.15
300520	2017	5	I65	1	1.66	1	15.19	0.15
300520	2018	6	I65	1	3.02	1	16.14	0.16
300520	2019	6	I65	1	5.2	1	16.14	0.16
300545	2018	3	C35	1	7.74	1	16.33	0.16
300545	2019	1	C35	1	6.17	1	16.33	0.16
300553	2019	1	C40	1	4.79	1	14.85	0.15
300567	2018	1	C40	0	15.56	2	17.99	0.18
300567	2019	1	C40	0	7.57	3	17.99	0.19
300568	2018	1	C26	1	6.82	3	15.87	0.16
300568	2019	1	C26	1	2.92	3	15.87	0.16
300577	2016	1	C17	1	13.23	2	14.82	0.1
300577	2017	2	C17	1	15.75	2	14.43	0.1
300577	2018	6	C17	1	15.37	2	14.09	0.1
300577	2019	4	C17	1	14.02	5	14.82	0.26
300596	2018	1	C26	1	11.18	3	13.56	0.14
300598	2019	2	I65	1	23.16	1	19.13	0.19
300604	2018	2	C35	1	5.99	1	17.74	0.18
300607	2019	4	C34	1	8.59	2	15.39	0.1
300624	2019	4	I65	1	9.98	2	19.26	0.19
300627	2017	6	C39	1	15.65	3	17.32	0.17
300627	2018	12	C39	1	8.5	3	17.74	0.18
300627	2019	9	C39	1	9.53	2	16.84	0.17
300647	2018	8	C39	1	2.35	1	18.55	0.18
300647	2019	7	C39	1	1.42	1	18.45	0.18
300662	2017	7	L72	1	11.8	5	16.85	0.14
300662	2018	3	L72	1	11.91	6	16.39	0.18
300662	2019	2	L72	1	11.68	8	16.91	0.19
300663	2018	2	I65	0	3.31	1	16.86	0.17

300663	2019	5	I65	0	3.19	1	16.86	0.17
300666	2018	7	C39	1	5.01	1	16.16	0.16
300666	2019	1	C39	1	4.38	1	16.16	0.16
300672	2019	3	C39	1	3.85	2	18.77	0.19
300673	2018	1	C13	1	13.06	3	15.32	0.2
300673	2019	3	C13	1	4.12	3	15.29	0.37
300674	2018	5	C13	0	13.06	3	15.32	0.2
300674	2019	15	C13	0	4.12	3	15.32	0.37
300676	2017	12	M74	1	9.07	6	14.45	0.2
300676	2018	1	M74	1	7.87	6	14.34	0.2
300676	2019	9	M74	1	4.99	10	14.34	0.16
300677	2019	2	M74	0	4.99	10	16.38	0.16
300703	2019	3	C24	1	11.32	4	15.81	0.15
300712	2019	3	E48	1	3.92	3	16.16	0.2
300716	2019	6	C29	1	1.25	2	15.94	0.15
300729	2017	9	C21	1	7.18	5	14.35	0.12
300729	2018	1	C21	1	5.09	5	14.52	0.14
300729	2019	12	C21	1	4.71	5	14.52	0.14
300738	2019	1	I65	1	7.71	1	17.85	0.18
300743	2018	4	C39	1	7.94	8	13.23	0.13
300743	2019	8	C39	1	5.29	8	13.28	0.13
300748	2018	1	C30	1	8.35	4	14.75	0.1
300748	2019	1	C30	1	6.4	4	14.75	0.1
300750	2018	1	C38	1	6.05	8	18.61	0.2
300750	2019	1	C38	1	5.72	8	21.16	0.21
300752	2019	17	C39	1	5	1	17.22	0.17
300760	2019	16	C35	1	19.83	15	15.42	0.15
300788	2019	8	R85	0	10.8	1	18.42	0.1
600005	2011	3	C31	1	1.1	2	21.35	0.1
600005	2012	1	C31	1	0.04	2	21.6	0.1
600005	2013	2	C31	1	0.46	2	21.69	0.1
600005	2014	1	C31	1	1.36	8	20.31	0.16
600005	2015	6	C31	1	-7.88	8	20.37	0.15
600006	2014	1	C31	1	1.36	8	20.31	0.16
600006	2015	17	C31	1	-7.88	8	20.37	0.15
600007	2014	10	C31	1	1.36	8	20.31	0.16
600007	2015	14	C31	1	-7.88	8	20.37	0.15
600011	2018	7	D44	0	0.62	3	22.59	0.5
600011	2019	10	D44	0	0.58	3	22.65	0.5
600018	2015	3	G55	1	8.16	6	21.93	0.23
600018	2016	10	G55	1	7.51	7	21.9	0.47
600018	2017	4	G55	1	9.96	8	22.17	0.41
600018	2018	1	G55	1	8.03	8	22.33	0.41
600018	2019	4	G55	1	6.93	8	22.47	0.43
600019	2010	1	C31	1	6.4	7	20.27	0.11
600019	2011	4	C31	1	3.46	9	20.03	0.11
600019	2012	5	C31	1	3.46	11	19.83	0.11
600019	2013	11	C31	1	2.74	10	21.04	0.11
600019	2014	3	C31	1	2.68	8	20.25	0.11
600019	2015	3	C31	1	0.31	7	20.39	0.11
600019	2016	1	C31	1	3.67	8	20.31	0.11

600019	2017	19	C31	1	6.6	8	21.49	0.13
600019	2018	11	C31	1	6.79	8	21.59	0.13
600019	2019	5	C31	1	3.99	7	21.74	0.13
600020	2015	10	G55	1	8.16	6	21.93	0.23
600020	2016	14	G55	1	7.51	7	21.9	0.47
600020	2017	12	G55	1	9.96	8	22.17	0.41
600020	2018	2	G55	1	8.03	8	22.33	0.41
600020	2019	2	G55	1	6.93	8	22.47	0.43
600021	2011	18	C31	0	3.46	9	20.03	0.11
600021	2012	12	C31	0	4.68	11	20.54	0.11
600021	2013	19	C31	0	2.74	10	21.04	0.11
600021	2014	10	C31	0	2.68	8	20.25	0.11
600021	2016	13	C31	0	3.67	8	20.31	0.11
600021	2017	1	C31	0	6.6	8	21.49	0.13
600021	2018	2	C31	0	6.79	8	21.59	0.13
600022	2012	2	C31	0	3.46	11	19.83	0.11
600022	2013	3	C31	0	2.74	10	21.04	0.11
600022	2014	1	C31	0	2.68	8	20.25	0.11
600022	2015	2	C31	0	0.31	7	20.39	0.11
600022	2016	17	C31	0	3.67	8	20.31	0.11
600022	2017	22	C31	0	6.6	8	21.49	0.13
600022	2018	17	C31	0	6.79	8	21.59	0.13
600022	2019	18	C31	0	3.99	7	21.74	0.13
600023	2012	15	C31	0	3.46	11	19.83	0.11
600023	2013	1	C31	0	2.74	10	21.04	0.11
600023	2014	1	C31	0	2.68	8	20.25	0.11
600023	2015	1	C31	0	0.31	7	20.39	0.11
600023	2016	5	C31	0	3.67	8	20.31	0.11
600023	2017	3	C31	0	6.6	8	21.49	0.13
600023	2018	1	C31	0	6.79	8	21.59	0.13
600023	2019	2	C31	0	3.99	7	21.74	0.13
600024	2013	2	D44	1	5.22	4	21.19	0.1
600024	2014	1	D44	1	4.74	7	20.53	0.16
600024	2015	3	D44	1	4.49	7	20.73	0.24
600024	2016	5	D44	1	3.08	9	20.61	0.22
600024	2017	5	D44	1	2.09	9	20.74	0.28
600024	2018	1	D44	1	3.8	8	21.11	0.3
600024	2019	9	D44	1	1.92	7	21.31	0.31
600025	2017	1	D44	1	1.44	4	20.26	0.1
600025	2018	2	D44	1	3.6	4	15.75	0.1
600025	2019	2	D44	1	3.54	4	20.4	0.1
600026	2014	3	D44	0	4.74	7	20.53	0.16
600026	2015	1	D44	0	4.49	7	20.73	0.24
600026	2016	2	D44	0	3.08	9	20.61	0.22
600026	2017	2	D44	0	2.09	9	20.74	0.28
600026	2018	1	D44	0	3.8	8	21.11	0.3
600026	2019	19	D44	0	1.92	7	21.31	0.31
600027	2018	10	D44	1	3.6	4	15.75	0.1
600027	2019	6	D44	1	3.54	4	20.4	0.1
600028	2017	2	B07	0	4.54	6	23.81	0.1
600028	2018	13	B07	0	5.04	6	23.91	0.1

600029	2017	6	D44	1	1.44	4	20.26	0.1
600030	2014	8	J67	0	3.16	7	20.15	0.59
600030	2015	11	J67	0	3.72	7	20.28	0.6
600030	2016	4	J67	0	1.81	8	20.02	0.58
600030	2017	1	J67	0	1.96	7	20.93	0.59
600030	2018	9	J67	0	1.54	9	20.73	0.47
600030	2019	7	J67	0	1.75	9	20.72	0.48
600031	2010	5	C35	1	26.13	11	16.71	0.1
600031	2011	4	C35	1	22.65	10	16.71	0.11
600031	2012	4	C35	1	10.38	11	16.16	0.15
600031	2013	2	C35	1	4.82	11	15.35	0.15
600031	2014	2	C35	1	1.19	12	15.35	0.13
600031	2015	2	C35	1	0.22	11	16.51	0.14
600031	2016	2	C35	1	0.27	9	16.75	0.11
600031	2017	3	C35	1	3.72	9	18.66	0.11
600031	2018	4	C35	1	9.55	9	19.5	0.11
600031	2019	10	C35	1	13.99	5	16.75	0.12
600032	2016	12	J67	0	1.81	8	20.02	0.58
600032	2017	9	J67	0	1.96	7	20.93	0.59
600032	2018	10	J67	0	1.54	9	20.73	0.47
600032	2019	5	J67	0	1.75	9	20.72	0.48
600033	2019	22	J67	0	1.75	9	20.72	0.48
600035	2016	13	D44	1	3.08	9	20.61	0.22
600035	2017	7	D44	1	2.09	9	20.74	0.28
600035	2018	4	D44	1	3.8	8	21.11	0.3
600035	2019	16	D44	1	1.92	7	21.31	0.31
600039	2019	21	E48	1	1.85	4	19.77	0.13
600050	2010	21	E48	0	0.85	5	16.07	0.14
600050	2011	28	E48	0	0.93	5	16.07	0.14
600050	2012	23	E48	0	1.44	5	16.12	0.14
600050	2013	24	E48	0	1.96	6	16	0.13
600050	2014	24	E48	0	2.22	9	19.64	0.14
600050	2015	7	E48	0	1.8	9	22.02	0.14
600050	2016	7	E48	0	0.08	11	21.83	0.13
600050	2017	2	E48	0	0.28	14	21.66	0.12
600050	2018	4	E48	0	1.67	15	21.7	0.13
600050	2019	5	E48	0	2.04	15	21.73	0.13
600051	2010	8	F51	0	2.97	2	17.9	0.1
600051	2011	7	F51	0	4.01	2	17.88	0.1
600051	2012	4	F51	0	0.3	2	17.61	0.1
600051	2013	6	F51	0	0.63	2	17.44	0.1
600052	2013	4	E48	0	1.96	6	16	0.13
600052	2014	4	E48	0	2.22	9	19.64	0.14
600052	2015	9	E48	0	1.8	9	22.02	0.14
600052	2016	16	E48	0	0.08	11	21.83	0.13
600052	2017	17	E48	0	0.28	14	21.66	0.12
600052	2018	14	E48	0	1.67	15	21.7	0.13
600052	2019	10	E48	0	2.04	15	21.73	0.13
600053	2016	8	E48	1	0.08	11	21.83	0.13
600053	2017	8	E48	1	0.28	14	21.66	0.12
600053	2018	6	E48	1	1.67	15	21.7	0.13

600053	2019	2	E48	1	2.04	15	21.73	0.13
600054	2017	61	E48	1	0.28	14	21.66	0.12
600054	2018	49	E48	1	1.67	15	21.7	0.13
600054	2019	38	E48	1	2.04	15	21.73	0.13
600055	2017	2	E48	1	0.28	14	21.66	0.12
600055	2018	1	E48	1	1.67	15	21.7	0.13
600055	2019	4	E48	1	2.04	15	21.73	0.13
600056	2018	16	E48	1	1.67	15	21.7	0.13
600056	2019	11	E48	1	2.04	15	21.73	0.13
600057	2019	18	L72	0	2.42	5	19.56	0.42
600058	2018	11	E48	1	1.67	15	21.7	0.13
600058	2019	13	E48	1	2.04	15	21.73	0.13
600061	2018	13	C39	1	2.02	6	18.12	0.17
600061	2019	12	C39	1	2.75	8	18.12	0.15
600063	2019	16	C39	0	2.75	8	17.91	0.15
600066	2016	27	C36	1	12.57	5	17.98	0.1
600066	2017	20	C36	1	8.88	7	18.37	0.1
600066	2018	1	C36	1	6.38	9	18.13	0.1
600066	2019	8	C36	1	5.34	9	18.23	0.1
600068	2016	9	E48	0	3.21	2	21.72	0.1
600068	2017	10	E48	0	3.46	3	21.56	0.1
600068	2018	6	E48	0	2.94	2	22.68	0.1
600071	2016	15	C40	1	-12.23	1	16.28	0.1
600071	2017	5	C40	1	3.52	1	16.14	0.1
600073	2010	7	C14	0	-0.41	1	19.81	0.1
600073	2011	5	C14	0	4.64	2	18.98	0.1
600073	2012	10	C14	0	2.72	2	18.68	0.1
600073	2013	25	C14	0	3.05	2	18.67	0.1
600073	2014	19	C14	0	1.22	2	18.74	0.1
600073	2015	4	C14	0	3.23	2	18.78	0.1
600073	2016	20	C14	0	4.76	2	19.02	0.1
600079	2016	18	C27	1	4.85	8	18.94	0.19
600079	2017	2	C27	1	7.5	18	18.99	0.18
600079	2018	4	C27	1	-5.54	19	18.78	0.18
600079	2019	3	C27	1	3.84	18	18.85	0.18
600081	2015	13	C36	1	6.57	1	19.34	0.1
600081	2016	14	C36	1	5.17	1	19.37	0.1
600081	2017	5	C36	1	5.45	1	19.53	0.1
600081	2018	1	C36	1	5.09	1	19.64	0.1
600081	2019	3	C36	1	4.82	1	19.71	0.1
600085	2010	3	C27	0	9.05	7	15.96	0.1
600085	2011	2	C27	0	10.21	6	15.38	0.1
600085	2012	4	C27	0	10.34	6	15.63	0.1
600085	2013	1	C27	0	9.89	5	15.59	0.1
600085	2014	1	C27	0	10.09	6	15.36	0.12
600085	2015	12	C27	0	10.74	6	15.4	0.15
600085	2016	2	C27	0	9.95	6	15.25	0.15
600085	2017	3	C27	0	9.74	6	15.25	0.13
600085	2018	1	C27	0	9.3	6	14.9	0.13
600086	2010	5	C27	0	9.05	7	15.96	0.1
600086	2011	5	C27	0	10.21	6	15.38	0.1

600086	2012	3	C27	0	10.34	6	15.63	0.1
600086	2013	1	C27	0	9.89	5	15.59	0.1
600086	2014	4	C27	0	10.09	6	15.36	0.12
600086	2015	5	C27	0	10.74	6	15.4	0.15
600086	2016	10	C27	0	9.95	6	15.25	0.15
600086	2017	8	C27	0	9.74	6	15.25	0.13
600086	2018	5	C27	0	9.3	6	14.9	0.13
600086	2019	1	C27	0	7.54	5	15.02	0.16
600087	2019	12	C27	0	7.54	5	15.02	0.16
600088	2010	13	C27	0	9.05	7	15.96	0.1
600088	2011	7	C27	0	10.21	6	15.38	0.1
600088	2012	4	C27	0	10.34	6	15.63	0.1
600088	2013	2	C27	0	9.89	5	15.59	0.1
600088	2014	1	C27	0	10.09	6	15.36	0.12
600088	2015	1	C27	0	10.74	6	15.4	0.15
600088	2016	15	C27	0	9.95	6	15.25	0.15
600088	2017	14	C27	0	9.74	6	15.25	0.13
600088	2018	17	C27	0	9.3	6	14.9	0.13
600088	2019	22	C27	0	7.54	5	15.02	0.16
600089	2012	26	C38	0	2.47	1	20.92	0.1
600089	2013	15	C38	0	2.97	2	20.36	0.1
600089	2014	14	C38	0	3.29	2	20.13	0.1
600089	2015	9	C38	0	3.13	2	20.17	0.1
600089	2016	5	C38	0	3.45	2	20.23	0.1
600089	2017	6	C38	0	3.33	2	20.32	0.1
600089	2018	6	C38	0	2.88	3	19.99	0.1
600089	2019	10	C38	0	2.45	5	20.08	0.1
600090	2010	3	C27	0	9.05	7	15.96	0.1
600090	2011	34	C27	0	10.21	6	15.38	0.1
600090	2012	34	C27	0	10.34	6	15.63	0.1
600090	2013	36	C27	0	9.89	5	15.59	0.1
600090	2014	32	C27	0	10.09	6	15.36	0.12
600090	2015	6	C27	0	10.74	6	15.4	0.15
600090	2016	5	C27	0	9.95	6	15.25	0.15
600090	2017	1	C27	0	9.74	6	15.25	0.13
600090	2018	3	C27	0	9.3	6	14.9	0.13
600092	2019	4	C38	1	2.45	5	20.08	0.1
600096	2013	4	C26	1	1.05	3	19.18	0.1
600096	2015	1	C26	1	0.28	5	19.56	0.1
600096	2016	2	C26	1	-5.25	5	19.22	0.12
600096	2017	13	C26	1	0.4	5	19.46	0.12
600096	2018	3	C26	1	0.38	5	19.77	0.12
600096	2019	3	C26	1	0.28	5	19.91	0.12
600097	2015	2	C26	1	0.28	5	19.56	0.1
600097	2016	5	C26	1	-5.25	5	19.22	0.12
600097	2017	1	C26	1	0.4	5	19.46	0.12
600097	2018	5	C26	1	0.38	5	19.77	0.12
600097	2019	3	C26	1	0.28	5	19.91	0.12
600100	2010	3	C39	0	2.59	1	21.33	0.1
600100	2011	5	C39	0	3.16	1	22.44	0.1
600100	2012	6	C39	0	2.64	1	22.56	0.1

600100	2013	1	C39	0	2.83	3	21.51	0.1
600100	2014	2	C39	0	2.63	4	21.48	0.1
600100	2015	2	C39	0	4.06	5	21.36	0.1
600100	2016	18	C39	0	8.21	5	21.61	0.12
600100	2017	10	C39	0	0.87	5	21.97	0.12
600100	2018	7	C39	0	-5.5	5	21.92	0.12
600100	2019	21	C39	0	0.86	5	21.94	0.12
600101	2010	15	C39	1	2.59	1	21.33	0.1
600101	2011	12	C39	1	3.16	1	22.44	0.1
600101	2012	3	C39	1	2.64	1	22.56	0.1
600101	2013	21	C39	1	2.83	3	21.51	0.1
600101	2014	15	C39	1	2.63	4	21.48	0.1
600102	2010	17	C39	0	2.59	1	21.33	0.1
600102	2011	5	C39	0	3.16	1	22.44	0.1
600102	2012	2	C39	0	2.64	1	22.56	0.1
600102	2013	1	C39	0	2.83	3	21.51	0.1
600102	2014	3	C39	0	2.63	4	21.48	0.1
600102	2015	1	C39	0	4.06	5	21.36	0.1
600102	2016	1	C39	0	8.21	5	21.61	0.12
600102	2017	1	C39	0	0.87	5	21.97	0.12
600102	2018	1	C39	0	-5.5	5	21.92	0.12
600102	2019	5	C39	0	0.86	5	21.94	0.12
600103	2017	4	C39	1	0.87	5	21.97	0.12
600103	2018	4	C39	1	-5.5	5	21.92	0.12
600103	2019	15	C39	1	0.86	5	21.94	0.12
600104	2013	9	C36	1	10.3	4	23.37	0.18
600104	2014	7	C36	1	9.7	4	23.49	0.13
600104	2015	8	C36	1	8.65	5	23.19	0.12
600104	2016	7	C36	1	7.98	7	22.92	0.13
600104	2017	2	C36	1	7.17	8	22.86	0.13
600104	2018	16	C36	1	6.43	8	22.91	0.14
600104	2019	9	C36	1	4.32	7	22.95	0.11
600105	2015	19	C38	0	6.63	1	20.58	0.1
600105	2016	5	C38	0	7.86	2	20.07	0.1
600105	2017	4	C38	0	7.6	2	20.07	0.1
600105	2018	4	C38	0	4.81	2	20.16	0.1
600106	2017	8	C39	1	0.87	5	21.97	0.12
600106	2018	9	C39	1	-5.5	5	21.92	0.12
600106	2019	12	C39	1	0.86	5	21.94	0.12
600107	2014	8	C39	1	2.63	4	21.48	0.1
600107	2015	7	C39	1	4.06	5	21.36	0.1
600107	2016	5	C39	1	8.21	5	21.61	0.12
600107	2017	6	C39	1	0.87	5	21.97	0.12
600107	2018	13	C39	1	-5.5	5	21.92	0.12
600107	2019	11	C39	1	0.86	5	21.94	0.12
600108	2015	4	C36	0	8.65	5	23.19	0.12
600108	2016	1	C36	0	7.98	7	22.92	0.13
600108	2017	4	C36	0	7.17	8	22.86	0.13
600108	2018	3	C36	0	6.43	8	22.91	0.14
600108	2019	2	C36	0	4.32	7	22.95	0.11
600109	2016	9	C36	0	7.98	7	22.92	0.13

600110	2017	4	C36	1	7.17	8	22.86	0.13
600110	2018	2	C36	1	6.43	8	22.91	0.14
600110	2019	3	C36	1	4.32	7	22.95	0.11
600118	2016	4	C39	0	4.5	1	16.67	0.1
600118	2017	4	C39	0	4.79	1	17.46	0.1
600118	2018	7	C39	0	4.65	1	17.79	0.1
600118	2019	6	C39	0	3.47	1	17.97	0.1
600120	2012	9	J69	0	6.13	2	19.59	0.2
600120	2013	3	J69	0	6.61	3	19.38	0.2
600120	2014	6	J69	0	5.96	4	18.02	0.23
600120	2015	1	J69	0	5.5	5	17.68	0.2
600121	2013	6	J69	0	6.61	3	19.38	0.2
600121	2014	14	J69	0	5.96	4	18.02	0.23
600121	2015	7	J69	0	5.5	5	17.68	0.2
600121	2016	14	J69	0	6.13	2	19.74	0.1
600121	2017	1	J69	0	4.88	2	20.77	0.15
600121	2018	5	J69	0	4.16	2	20.67	0.15
600121	2019	7	J69	0	4.82	2	20.88	0.15
600122	2014	2	J69	1	5.96	4	18.02	0.23
600122	2015	2	J69	1	5.5	5	17.68	0.2
600123	2015	2	J69	1	5.5	5	17.68	0.2
600127	2010	2	C13	0	0.43	1	18.02	0.1
600127	2011	2	C13	0	-4.93	1	17.32	0.1
600127	2012	2	C13	0	0.51	1	17.34	0.1
600127	2013	4	C13	0	0.91	1	17.44	0.1
600127	2014	1	C13	0	0.93	1	16.93	0.1
600127	2015	3	C13	0	-11.98	1	16.94	0.1
600127	2016	1	C13	0	0.56	1	17.11	0.1
600127	2017	2	C13	0	0.54	1	17.13	0.1
600127	2018	4	C13	0	-2.57	1	17.15	0.2
600128	2016	12	F51	1	0.88	3	18.98	0.1
600128	2017	13	F51	1	0.59	6	18.02	0.15
600128	2018	5	F51	1	-1.96	8	17.72	0.14
600128	2019	5	F51	1	0.27	7	17.84	0.14
600129	2017	1	F51	1	0.59	6	18.02	0.15
600129	2018	3	F51	1	-1.96	8	17.72	0.14
600129	2019	21	F51	1	0.27	7	17.84	0.14
600130	2017	27	F51	1	0.59	6	18.02	0.15
600130	2018	4	F51	1	-1.96	8	17.72	0.14
600130	2019	15	F51	1	0.27	7	17.84	0.14
600138	2015	13	L72	0	4.95	2	20.36	0.1
600138	2016	7	L72	0	7.19	2	20.54	0.1
600143	2013	4	C29	0	5.78	2	16.89	0.1
600143	2014	2	C29	0	3.57	2	16.69	0.1
600143	2015	2	C29	0	4.87	5	17	0.16
600143	2016	16	C29	0	4.21	7	18.26	0.1
600143	2017	2	C29	0	2.69	8	18.36	0.1
600143	2018	1	C29	0	2.89	8	19.37	0.1
600143	2019	4	C29	0	4.9	8	19.11	0.1
600144	2016	3	C29	1	4.21	7	18.26	0.1
600144	2017	4	C29	1	2.69	8	18.36	0.1

600144	2018	14	C29	1	2.89	8	19.37	0.1
600144	2019	8	C29	1	4.9	8	20.11	0.1
600151	2016	32	C39	1	1.73	5	16.33	0.16
600151	2017	13	C39	1	-2.59	5	16.63	0.16
600151	2018	9	C39	1	0.26	12	17.22	0.13
600152	2010	39	C39	0	2.3	1	19.6	0.1
600152	2011	42	C39	0	6.44	1	19.55	0.1
600152	2012	42	C39	0	-4.41	1	19.61	0.1
600152	2013	11	C39	0	0.49	1	19.57	0.1
600152	2014	135	C39	0	-12.59	2	18.78	0.1
600153	2015	115	F51	1	3.69	6	19.29	0.48
600153	2016	9	F51	1	3.5	7	19.69	0.56
600153	2017	1	F51	1	3.2	8	19.68	0.53
600154	2018	19	C39	1	0.26	12	17.22	0.13
600154	2019	17	C39	1	-6.76	12	17.43	0.13
600155	2018	21	C39	1	0.26	12	17.22	0.13
600155	2019	49	C39	1	-6.76	12	17.43	0.13
600156	2018	46	C39	1	0.26	12	17.22	0.13
600156	2019	28	C39	1	-6.76	12	17.43	0.13
600157	2018	152	C39	1	0.26	12	17.22	0.13
600157	2019	4	C39	1	-6.76	12	17.43	0.13
600158	2019	2	C39	1	-6.76	12	17.43	0.13
600159	2019	4	C39	1	-6.76	12	17.43	0.13
600166	2010	8	C36	1	7.78	3	19	0.1
600166	2011	10	C36	1	4.37	4	19.27	0.13
600166	2012	6	C36	1	4.58	7	19.05	0.13
600166	2013	7	C36	1	2.28	7	19.27	0.11
600166	2014	11	C36	1	1.37	7	19.51	0.11
600166	2015	10	C36	1	0.92	10	19.26	0.14
600166	2016	10	C36	1	1.06	10	19.32	0.14
600166	2017	1	C36	1	0.05	10	19.52	0.14
600166	2018	4	C36	1	-6	13	19.64	0.19
600166	2019	1	C36	1	0.26	11	20.15	0.17
600167	2010	9	C36	1	7.78	3	19	0.1
600167	2011	29	C36	1	4.37	4	19.27	0.13
600167	2012	48	C36	1	4.58	7	19.05	0.13
600167	2013	100	C36	1	2.28	7	19.27	0.11
600167	2014	98	C36	1	1.37	7	19.51	0.11
600167	2015	68	C36	1	0.92	10	19.26	0.14
600167	2016	7	C36	1	1.06	10	19.32	0.14
600167	2017	1	C36	1	0.05	10	19.52	0.14
600167	2018	8	C36	1	-6	13	19.64	0.19
600167	2019	3	C36	1	0.26	11	20.15	0.17
600168	2015	3	C36	0	0.92	10	19.26	0.14
600168	2016	3	C36	0	1.06	10	19.32	0.14
600168	2017	2	C36	0	0.05	10	19.52	0.14
600168	2018	40	C36	0	-6	13	19.64	0.19
600168	2019	36	C36	0	0.26	11	20.15	0.17
600169	2011	87	C35	1	2.32	2	13.24	0.1
600169	2012	101	C35	1	-1.69	2	13	0.1
600169	2015	39	C35	1	0.08	3	14.26	0.1

600169	2016	24	C35	1	-6.68	3	14.71	0.1
600169	2017	55	C35	1	0.17	3	14.71	0.1
600169	2018	103	C35	1	0.13	3	14.84	0.1
600169	2019	178	C35	1	-2.49	3	17.44	0.1
600170	2014	194	E48	1	1.74	6	18.53	0.2
600170	2018	188	E48	1	1.67	10	19.36	0.18
600171	2011	1	C36	1	4.37	4	19.27	0.13
600171	2012	30	C36	1	4.58	7	19.05	0.13
600171	2013	37	C36	1	2.28	7	19.27	0.11
600171	2014	39	C36	1	1.37	7	19.51	0.11
600171	2015	99	C36	1	0.92	10	19.26	0.14
600171	2016	66	C36	1	1.06	10	19.32	0.14
600171	2017	15	C36	1	0.05	10	19.52	0.14
600171	2018	21	C36	1	-6	13	19.64	0.19
600171	2019	37	C36	1	0.26	11	20.15	0.17
600172	2012	78	C36	1	4.58	7	19.05	0.13
600172	2013	93	C36	1	2.28	7	19.27	0.11
600172	2014	106	C36	1	1.37	7	19.51	0.11
600172	2015	1	C36	1	0.92	10	19.26	0.14
600172	2016	1	C36	1	1.06	10	19.32	0.14
600172	2017	5	C36	1	0.05	10	19.52	0.14
600172	2018	2	C36	1	-6	13	19.64	0.19
600172	2019	3	C36	1	0.26	11	20.15	0.17
600173	2018	2	C36	1	-6	13	19.64	0.19
600173	2019	4	C36	1	0.26	11	20.15	0.17
600174	2016	19	E48	1	1.36	6	18.94	0.12
600174	2017	16	E48	1	1.51	6	18.79	0.12
600174	2018	16	E48	1	1.67	10	19.36	0.18
600174	2019	16	E48	1	1.82	5	20.63	0.14
600175	2011	35	C30	0	2.88	13	15.85	0.12
600175	2012	29	C30	0	1.65	14	15.7	0.12
600175	2014	53	C30	0	2.46	15	15.38	0.12
600175	2015	3	C30	0	4.55	14	15.43	0.12
600175	2016	5	C30	0	6.37	14	18.08	0.13
600175	2017	1	C30	0	8.86	13	18.33	0.12
600175	2018	6	C30	0	8.65	13	18.35	0.12
600175	2019	8	C30	0	6.61	13	18.37	0.12
600176	2012	1	C30	1	1.65	14	15.7	0.12
600176	2014	2	C30	1	2.46	15	15.38	0.12
600176	2015	1	C30	1	4.55	14	15.43	0.12
600176	2016	6	C30	1	6.37	14	18.08	0.13
600176	2017	8	C30	1	8.86	13	18.33	0.12
600176	2018	3	C30	1	8.65	13	18.35	0.12
600176	2019	23	C30	1	6.61	13	18.37	0.12
600177	2010	35	C18	1	5.06	5	19.24	0.34
600177	2011	45	C18	1	5.7	6	19.67	0.28
600178	2011	38	C30	0	2.88	13	15.85	0.12
600178	2012	4	C30	0	1.65	14	15.7	0.12
600178	2014	4	C30	0	2.46	15	15.38	0.12
600178	2015	5	C30	0	4.55	14	15.43	0.12
600178	2016	1	C30	0	6.37	14	18.08	0.13

600178	2017	1	C30	0	8.86	13	18.33	0.12
600178	2018	2	C30	0	8.65	13	18.35	0.12
600178	2019	2	C30	0	6.61	13	18.37	0.12
600179	2010	4	C18	0	5.06	5	19.24	0.34
600179	2011	7	C18	0	5.7	6	19.17	0.28
600180	2013	3	F51	1	10.89	3	18.67	0.17
600180	2014	2	F51	1	6.86	3	19.59	0.17
600180	2015	4	F51	1	4.38	3	19.62	0.17
600180	2016	11	F51	1	3.79	3	19.51	0.17
600180	2017	9	F51	1	3.81	4	19.29	0.15
600180	2018	12	F51	1	2.28	4	19.92	0.15
600180	2019	9	F51	1	1.91	4	21.19	0.15
600181	2011	9	C30	1	2.88	13	15.85	0.12
600181	2012	1	C30	1	1.65	14	15.7	0.12
600181	2014	1	C30	1	2.46	15	15.38	0.12
600181	2015	7	C30	1	4.55	14	15.43	0.12
600181	2016	4	C30	1	6.37	14	18.08	0.13
600181	2017	1	C30	1	8.86	13	18.33	0.12
600181	2018	13	C30	1	8.65	13	18.35	0.12
600181	2019	13	C30	1	6.61	13	18.37	0.12
600182	2018	19	E48	1	1.67	10	19.36	0.18
600196	2012	5	C27	0	7.7	4	21.49	0.13
600196	2013	3	C27	0	8.73	3	21.92	0.17
600196	2014	6	C27	0	7.31	5	21.59	0.14
600196	2015	17	C27	0	7.81	5	21.75	0.22
600196	2017	12	C27	0	6.78	10	21.34	0.18
600196	2018	8	C27	0	4.56	10	21.49	0.19
600197	2016	5	C27	0	7.86	4	22.12	0.2
600197	2019	16	C27	0	5.1	9	22.57	0.19
600198	2017	15	C27	1	6.78	10	21.34	0.18
600198	2018	1	C27	1	4.56	10	21.09	0.19
600198	2019	12	C27	1	5.1	9	21.57	0.19
600202	2019	26	C38	0	2.74	1	17.25	0.1
600219	2015	98	C32	1	2.02	4	17.99	0.15
600219	2016	160	C32	1	3.47	4	18.07	0.15
600219	2017	84	C32	1	3.88	5	17.88	0.14
600219	2018	120	C32	1	3.1	4	18.26	0.15
600219	2019	4	C32	1	3.24	4	18.39	0.15
600234	2017	33	G60	1	11.43	22	16.12	0.23
600234	2018	15	G60	1	11.33	25	16.12	0.24
600234	2019	18	G60	1	7.98	22	16.12	0.25
600237	2018	9	G60	0	11.33	25	15.34	0.24
600237	2019	12	G60	0	7.98	22	15.47	0.25
600250	2010	15	F51	1	-1.63	1	19.77	0.1
600250	2011	8	F51	1	-3.65	1	19.91	0.1
600250	2012	9	F51	1	-0.24	2	19.12	0.1
600250	2013	52	F51	1	4.38	2	19.87	0.1
600250	2014	43	F51	1	0.53	2	19.83	0.1
600250	2015	52	F51	1	-2.53	2	19.82	0.1
600250	2016	37	F51	1	0.88	2	19.87	0.1
600250	2017	23	F51	1	5.23	2	20.02	0.1

600250	2018	30	F51	1	10.32	2	19.99	0.15
600250	2019	33	F51	1	5.17	2	19.9	0.25
600256	2010	20	B07	0	6.27	3	18.63	0.1
600256	2011	21	B07	0	6.1	3	15.05	0.1
600256	2013	1	B07	0	2.89	3	18.51	0.17
600256	2014	5	B07	0	5.06	3	18.99	0.17
600256	2015	8	B07	0	0.59	4	19.09	0.15
600256	2016	5	B07	0	0.34	4	19.17	0.18
600256	2017	7	B07	0	1.11	4	19.18	0.15
600256	2018	12	B07	0	3.49	4	18.49	0.15
600256	2019	6	B07	0	2.98	5	18.69	0.14
600261	2010	4	C38	1	7.42	5	17	0.12
600261	2011	6	C38	1	7.56	4	17.16	0.13
600261	2012	2	C38	1	5.83	5	16.9	0.12
600261	2013	6	C38	1	5.47	5	16.79	0.12
600262	2010	11	C38	0	7.42	5	17	0.12
600262	2012	21	C38	0	5.83	5	16.9	0.12
600262	2013	23	C38	0	5.47	5	16.79	0.12
600263	2014	1	F51	1	2.88	9	15.87	0.17
600276	2018	2	C27	1	20.1	6	12.02	0.13
600276	2019	2	C27	1	21.34	6	16.12	0.18
600277	2016	2	C26	1	1.78	2	21.59	0.1
600277	2017	1	C26	1	2.86	2	21.65	0.1
600278	2011	1	F51	0	5.88	8	17.45	0.16
600278	2012	1	F51	0	3.64	8	17.66	0.18
600278	2013	1	F51	0	2.83	8	17.62	0.18
600278	2014	2	F51	0	2.88	9	15.87	0.17
600282	2012	1	C31	1	-1.64	3	19.27	0.1
600282	2013	1	C31	1	-1.74	5	18.77	0.1
600282	2014	1	C31	1	0.77	6	17.85	0.12
600282	2015	13	C31	1	-6.41	7	17.63	0.11
600282	2016	11	C31	1	1	7	17.34	0.17
600282	2017	11	C31	1	9.44	7	17.53	0.16
600282	2018	1	C31	1	12.01	6	19.63	0.18
600282	2019	3	C31	1	7.93	6	19.65	0.2
600284	2014	3	C31	0	0.77	6	17.85	0.12
600284	2016	2	C31	0	1	7	17.34	0.17
600284	2018	3	C31	0	12.01	6	17.63	0.18
600284	2019	4	C31	0	7.93	6	19.65	0.2
600295	2014	4	C31	1	2.09	1	22.44	0.1
600295	2015	4	C31	1	1.1	1	22.5	0.1
600295	2016	4	C31	1	1.29	1	22.5	0.1
600295	2017	3	C31	1	2.26	2	21.82	0.1
600295	2018	4	C31	1	3.31	2	21.86	0.1
600295	2019	2	C31	1	3.15	2	21.91	0.1
600298	2010	13	C14	1	12.73	2	16.77	0.1
600298	2011	8	C14	1	9.46	2	16.81	0.1
600298	2012	10	C14	1	5.91	2	16.77	0.1
600298	2013	2	C14	1	3.09	2	16.77	0.1
600298	2014	4	C14	1	2.8	2	16.26	0.1
600298	2015	8	C14	1	5.11	3	15.86	0.1

600298	2016	4	C14	1	8.9	3	15.99	0.1
600298	2017	6	C14	1	12.06	3	16.15	0.1
600298	2018	15	C14	1	10.58	3	16.12	0.1
600298	2019	13	C14	1	9.95	3	15.88	0.1
600299	2015	5	C14	1	5.11	3	15.86	0.1
600299	2016	7	C14	1	8.9	3	15.99	0.1
600299	2017	5	C14	1	12.06	3	16.15	0.1
600299	2018	119	C14	1	10.58	3	16.12	0.1
600299	2019	17	C14	1	9.95	3	15.88	0.1
600303	2016	17	C36	1	0.71	1	18.09	0.1
600303	2017	25	C36	1	3.71	2	16.95	0.1
600303	2018	28	C36	1	-1.86	1	15.9	0.1
600304	2019	38	C26	1	12.19	13	17.83	0.25
600305	2012	49	C26	1	15.11	5	16.02	0.1
600305	2013	2	C26	1	14.03	5	16.93	0.1
600305	2014	9	C26	1	8.85	7	16.36	0.14
600305	2015	6	C26	1	5.1	8	16.96	0.15
600305	2016	4	C26	1	9.23	7	17.76	0.16
600305	2017	5	C26	1	22.83	7	18.13	0.16
600305	2018	8	C26	1	17.98	7	18.34	0.17
600305	2019	6	C26	1	12.19	13	17.83	0.25
600306	2019	9	C26	1	12.19	13	17.83	0.25
600307	2010	7	C26	1	15.23	4	15.42	0.13
600307	2011	3	C26	1	15.78	4	15.65	0.13
600307	2012	5	C26	1	15.11	5	16.02	0.1
600307	2013	3	C26	1	14.03	5	16.93	0.1
600307	2014	1	C26	1	8.85	7	16.36	0.14
600307	2015	1	C26	1	5.1	8	16.96	0.15
600307	2016	1	C26	1	9.23	7	17.76	0.16
600307	2017	2	C26	1	22.83	7	18.13	0.16
600307	2018	3	C26	1	17.98	7	18.34	0.17
600307	2019	6	C26	1	12.19	13	17.83	0.25
600309	2019	11	C26	1	12.19	13	17.83	0.25
600310	2015	20	C26	1	5.1	8	16.96	0.15
600310	2016	5	C26	1	9.23	7	17.76	0.16
600310	2017	2	C26	1	22.83	7	18.13	0.16
600310	2018	3	C26	1	17.98	7	18.34	0.17
600310	2019	3	C26	1	12.19	13	17.83	0.25
600312	2016	3	C38	1	8.53	1	18.76	0.1
600312	2017	2	C38	1	3.5	1	18.85	0.1
600312	2018	4	C38	1	1.24	1	18.93	0.1
600312	2019	6	C38	1	1.02	1	19.01	0.1
600315	2017	9	C26	1	4.52	10	16.85	0.3
600315	2018	7	C26	1	5.47	10	17.13	0.32
600315	2019	26	C26	1	5.23	11	17.39	0.3
600320	2015	1	C35	0	0.34	15	18.48	0.17
600320	2016	10	C35	0	0.51	17	18.68	0.17
600320	2017	11	C35	0	0.51	20	18.57	0.19
600320	2018	30	C35	0	0.57	21	18.7	0.18
600320	2019	25	C35	0	0.69	21	18.73	0.18
600321	2014	29	C35	0	0.28	13	18.08	0.12

600321	2015	2	C35	0	0.34	15	18.48	0.17
600321	2016	5	C35	0	0.51	17	18.68	0.17
600321	2017	1	C35	0	0.51	20	18.57	0.19
600321	2018	8	C35	0	0.57	21	18.7	0.18
600321	2019	6	C35	0	0.69	21	18.73	0.18
600322	2013	1	C35	0	0.28	5	18.15	0.1
600322	2014	2	C35	0	0.28	13	18.08	0.12
600322	2015	6	C35	0	0.34	15	18.48	0.17
600322	2016	6	C35	0	0.51	17	18.68	0.17
600322	2017	7	C35	0	0.51	20	18.57	0.19
600322	2018	4	C35	0	0.57	21	18.7	0.18
600322	2019	2	C35	0	0.69	21	18.73	0.18
600323	2013	2	C35	0	0.28	5	18.15	0.1
600323	2014	5	C35	0	0.28	13	18.08	0.12
600323	2015	8	C35	0	0.34	15	18.48	0.17
600323	2016	73	C35	0	0.51	17	18.68	0.17
600323	2017	43	C35	0	0.51	20	18.57	0.19
600323	2018	39	C35	0	0.57	21	18.7	0.18
600323	2019	14	C35	0	0.69	21	18.73	0.18
600324	2014	12	C35	1	0.28	13	18.08	0.12
600324	2015	11	C35	1	0.34	15	18.48	0.17
600324	2016	6	C35	1	0.51	17	18.68	0.17
600324	2017	6	C35	1	0.51	20	18.57	0.19
600324	2018	9	C35	1	0.57	21	18.7	0.18
600324	2019	1	C35	1	0.69	21	18.73	0.18
600335	2019	3	F51	1	1.74	7	17.11	0.16
600336	2019	5	C38	1	3.4	3	18.31	0.13
600337	2010	2	F52	1	3.83	2	16.32	0.1
600337	2015	6	F52	1	6.82	2	15.37	0.15
600337	2016	9	F52	1	6.64	2	15.25	0.15
600337	2019	2	F52	1	5.42	5	17.12	0.16
600338	2015	3	B09	1	16.36	1	14.03	0.1
600338	2016	6	B09	1	41.04	1	13.97	0.2
600338	2017	8	B09	1	42.96	1	14	0.2
600338	2018	7	B09	1	22.44	2	13.24	0.15
600338	2019	3	B09	1	13.38	2	13.25	0.15
600339	2019	1	F51	1	1.74	7	17.11	0.16
600341	2019	3	F52	0	5.42	5	17.12	0.16
600343	2016	10	K70	1	2.95	9	18.69	0.41
600343	2017	19	K70	1	2.81	15	19.41	0.43
600343	2018	33	K70	1	3	16	19.41	0.46
600343	2019	45	K70	1	3.39	17	19.41	0.49
600346	2014	15	C28	1	-6.44	3	13.96	0.2
600349	2012	56	C26	0	4.71	9	17.64	0.12
600349	2013	79	C26	0	8.1	9	17.21	0.12
600351	2014	82	C26	0	13.37	5	17.81	0.12
600351	2015	21	C26	0	11.59	5	17.06	0.16
600351	2016	12	C26	0	7.46	5	17.36	0.16
600351	2017	27	C26	0	6.33	5	18.97	0.18
600351	2018	57	C26	0	8.55	5	17.09	0.18
600351	2019	2	C26	0	10.22	6	17.98	0.15

600352	2010	1	C26	0	7.48	2	19.29	0.1
600352	2011	3	C26	0	5.35	2	19.3	0.1
600352	2012	22	C26	0	4.71	9	17.64	0.12
600352	2013	88	C26	0	8.1	9	18.21	0.12
600362	2010	147	C32	0	10.69	3	19.59	0.13
600362	2011	10	C32	0	10.75	3	20.07	0.13
600362	2012	12	C32	0	7.24	3	20.34	0.17
600362	2013	2	C32	0	4.36	4	20.32	0.15
600362	2014	6	C32	0	3.1	5	20.31	0.14
600362	2016	9	C32	0	1.06	6	20.03	0.18
600362	2017	23	C32	0	1.85	6	20.09	0.18
600362	2018	13	C32	0	2.45	7	20.08	0.19
600363	2010	12	C39	0	3.12	1	19.55	0.1
600363	2011	6	C39	0	5.57	1	19.72	0.1
600363	2012	4	C39	0	5.21	1	19.95	0.1
600363	2013	2	C39	0	5.92	1	20.4	0.1
600363	2014	2	C39	0	4.84	1	20.54	0.1
600364	2014	1	C32	1	3.1	5	20.31	0.14
600364	2015	13	C32	1	0.74	4	20.4	0.15
600364	2016	11	C32	1	1.06	6	20.03	0.18
600364	2017	8	C32	1	1.85	6	20.09	0.18
600364	2018	1	C32	1	2.45	7	20.08	0.19
600366	2010	6	C30	1	8.74	3	17.67	0.17
600366	2011	50	C30	1	23.08	3	18.46	0.13
600366	2012	47	C30	1	12.92	4	18.29	0.13
600388	2011	75	C35	1	4.34	2	13.82	0.1
600388	2012	74	C35	1	4.42	2	13.76	0.1
600388	2013	25	C35	1	5.8	2	14.44	0.1
600388	2014	14	C35	1	4.68	2	14.59	0.1
600388	2015	20	C35	1	4.5	2	15.69	0.1
600388	2016	23	C35	1	4.77	2	14.73	0.1
600388	2018	80	C35	1	4.82	2	15.4	0.1
600388	2019	127	C35	1	4.26	3	17.43	0.13
600389	2019	127	C35	0	4.26	3	17.43	0.13
600392	2016	166	C32	1	-1.3	2	18.23	0.15
600392	2017	237	C32	1	5.8	3	17.6	0.17
600392	2018	33	C32	1	3.18	4	17.29	0.15
600392	2019	42	C32	1	1.23	4	17.66	0.15
600398	2018	46	C18	1	12.64	7	18.52	0.16
600398	2019	49	C18	1	10.83	8	11.65	0.15
600405	2018	39	C39	0	-10.15	8	13.69	0.15
600405	2019	4	C39	0	0.41	8	13.68	0.15
600406	2017	2	I65	0	11.66	8	13.35	0.13
600406	2018	18	I65	0	9.05	8	14.6	0.14
600406	2019	26	I65	0	8.49	9	15.73	0.12
600407	2017	166	I65	1	11.66	8	13.35	0.13
600407	2018	194	I65	1	9.05	8	14.6	0.14
600407	2019	1	I65	1	8.49	9	15.73	0.12
600410	2010	2	I65	0	7.3	4	13.9	0.1
600410	2011	1	I65	0	6.43	4	13.93	0.1
600410	2012	1	I65	0	3.9	4	15.66	0.1

600410	2013	2	I65	0	0.99	4	15.15	0.1
600410	2014	1	I65	0	1.71	8	15.89	0.13
600410	2015	1	I65	0	1.59	8	17.16	0.11
600410	2016	17	I65	0	0.47	8	17.73	0.11
600410	2019	35	I65	0	1.81	7	19.21	0.14
600418	2012	13	C36	1	2.94	4	18.57	0.1
600418	2013	28	C36	1	4.31	4	18.88	0.1
600418	2014	21	C36	1	2.13	6	18.9	0.1
600418	2015	12	C36	1	2.6	6	19.09	0.1
600418	2016	12	C36	1	2.76	6	19.27	0.1
600418	2017	9	C36	1	0.51	6	19.42	0.1
600418	2018	20	C36	1	-3.09	6	19.98	0.1
600418	2019	16	C36	1	0.38	5	20.33	0.1
600419	2010	17	C36	1	8.43	2	16.79	0.1
600419	2011	1	C36	1	4.13	3	16.49	0.1
600419	2012	6	C36	1	2.94	4	18.57	0.1
600419	2013	5	C36	1	4.31	4	18.88	0.1
600419	2014	7	C36	1	2.13	6	18.9	0.1
600419	2015	5	C36	1	2.6	6	19.09	0.1
600419	2016	1	C36	1	2.76	6	19.27	0.1
600419	2017	14	C36	1	0.51	6	19.42	0.1
600419	2018	26	C36	1	-3.09	6	19.98	0.1
600419	2019	1	C36	1	0.38	5	20.33	0.1
600420	2011	7	C36	0	4.13	3	16.49	0.1
600420	2012	3	C36	0	2.94	4	18.57	0.1
600420	2013	8	C36	0	4.31	4	18.88	0.1
600420	2014	18	C36	0	2.13	6	18.9	0.1
600420	2015	11	C36	0	2.6	6	19.09	0.1
600420	2016	8	C36	0	2.76	6	19.27	0.1
600420	2017	6	C36	0	0.51	6	19.42	0.1
600420	2018	5	C36	0	-3.09	6	19.98	0.1
600420	2019	5	C36	0	0.38	5	20.33	0.1
600422	2010	23	C27	0	7.42	1	14.92	0.1
600422	2011	14	C27	0	9.55	1	14.92	0.1
600422	2012	27	C27	0	10.64	1	14.92	0.1
600422	2016	34	C27	0	10.82	7	15.49	0.11
600422	2017	27	C27	0	7.91	7	15.17	0.11
600422	2018	2	C27	0	5.71	7	16.02	0.11
600422	2019	4	C27	0	5.17	7	16.35	0.13
600428	2018	1	G55	0	0.4	7	18.53	0.11
600429	2018	1	G55	0	0.4	7	18.53	0.11
600430	2018	2	G55	0	0.4	7	18.53	0.11
600433	2019	2	C22	1	4.22	1	16.63	0.1
600438	2010	5	C13	1	3.09	1	16.28	0.2
600438	2011	1	C13	1	1.84	1	16.43	0.2
600438	2012	4	C13	1	1.98	1	16.87	0.3
600438	2013	12	C13	1	5.96	3	15.96	0.13
600438	2014	12	C13	1	5.86	3	15.56	0.17
600438	2015	2	C13	1	5.57	3	16.68	0.17
600438	2016	3	C13	1	7.45	2	17.83	0.1
600438	2017	2	C13	1	8.7	1	18.82	0.1

600439	2011	2	C19	1	6.96	9	16.18	0.12
600439	2012	6	C19	1	4.01	9	16.18	0.12
600439	2013	9	C19	1	4.16	9	16.18	0.11
600460	2013	9	C39	0	3.16	5	17.1	0.1
600460	2014	7	C39	0	4.1	4	17.07	0.1
600460	2015	24	C39	0	0.99	4	16.82	0.1
600460	2016	15	C39	0	1.94	4	16.88	0.1
600478	2011	10	C38	1	0.12	4	16.17	0.1
600478	2012	6	C38	1	-2.77	4	16.48	0.1
600478	2013	5	C38	1	1.41	4	16.41	0.1
600478	2014	9	C38	1	-1.97	4	17.56	0.1
600478	2015	9	C38	1	1.78	3	17.69	0.1
600478	2016	7	C38	1	-5.58	1	18.52	0.1
600478	2017	13	C38	1	0.68	3	18.45	0.13
600478	2018	9	C38	1	0.08	3	18.16	0.13
600478	2019	27	C38	1	-6.42	3	18.76	0.13
600480	2016	6	C36	1	4.04	2	19.1	0.1
600480	2017	3	C36	1	5.25	2	19.14	0.1
600480	2018	1	C36	1	4.41	2	19.04	0.1
600480	2019	1	C36	1	1.14	2	19.08	0.1
600487	2016	1	C38	0	8.65	10	18.3	0.1
600487	2017	10	C38	0	9.32	12	18.36	0.1
600487	2018	107	C38	0	8.2	15	18.29	0.11
600487	2019	235	C38	0	3.45	14	18.33	0.16
600488	2017	249	C38	1	9.32	12	18.36	0.1
600488	2018	6	C38	1	8.2	15	18.36	0.11
600488	2019	21	C38	1	3.45	14	18.63	0.16
600491	2010	43	E48	1	2.21	5	16.18	0.12
600491	2011	47	E48	1	2.87	5	16.34	0.12
600491	2012	65	E48	1	2.97	5	15.14	0.12
600491	2013	72	E48	1	1.37	5	16.62	0.12
600491	2015	2	E48	1	0.88	5	14.55	0.12
600491	2016	2	E48	1	1.35	5	18.33	0.12
600491	2017	2	E48	1	1.89	5	18.68	0.12
600491	2018	10	E48	1	2.12	6	18.56	0.12
600491	2019	50	E48	1	1.86	6	18.59	0.12
600496	2012	64	C33	1	3.34	5	16.14	0.22
600496	2013	58	C33	1	3.01	5	16.13	0.22
600496	2014	1	C33	1	2.92	7	17.47	0.19
600496	2015	2	C33	1	1.89	9	17.23	0.17
600496	2016	2	C33	1	1.04	8	17.42	0.18
600496	2017	2	C33	1	0.56	10	18.09	0.15
600497	2015	3	C33	0	1.89	9	17.23	0.17
600497	2017	16	C33	0	0.56	10	18.09	0.15
600497	2018	15	C33	0	1.46	9	18.25	0.16
600498	2010	61	C39	1	6.08	2	18.6	0.1
600498	2011	6	C39	1	5.64	2	18.74	0.1
600498	2012	2	C39	1	4.98	6	17.6	0.1
600498	2013	1	C39	1	4.82	9	17.44	0.1
600498	2014	2	C39	1	4.23	11	17.15	0.1
600498	2015	2	C39	1	4.1	12	17.22	0.1

600499	2016	3	C35	0	3.33	4	15.65	0.13
600499	2017	14	C35	0	4.79	7	18.77	0.11
600499	2018	8	C35	0	-4.21	8	18.77	0.15
600499	2019	10	C35	0	1.83	12	18.39	0.13
600500	2012	7	C26	1	2.6	14	19.85	0.17
600500	2013	2	C26	1	2.54	11	20.16	0.15
600500	2014	6	C26	1	2.99	11	20.07	0.15
600500	2015	7	C26	1	2.92	12	19.46	0.14
600500	2016	6	C26	1	0.98	12	19.41	0.15
600500	2017	9	C26	1	2.58	9	18.57	0.18
600500	2018	9	C26	1	3.97	6	17.82	0.2
600500	2019	12	C26	1	3.1	4	18.73	0.28
600501	2018	14	C33	1	1.46	9	18.25	0.16
600501	2019	8	C33	1	2.95	11	19.11	0.13
600502	2017	12	E48	1	1.93	6	17.88	0.1
600502	2018	51	E48	1	1.18	6	18.03	0.1
600502	2019	76	E48	1	0.91	6	18.16	0.1
600503	2015	96	C39	1	4.1	12	17.22	0.1
600504	2012	6	C39	1	4.98	6	17.6	0.1
600504	2013	3	C39	1	4.82	9	17.44	0.1
600504	2014	4	C39	1	4.23	11	17.15	0.1
600504	2015	3	C39	1	4.1	12	17.22	0.1
600505	2012	1	C39	1	4.98	6	17.6	0.1
600505	2013	11	C39	1	4.82	9	17.44	0.1
600505	2014	11	C39	1	4.23	11	17.15	0.1
600505	2015	7	C39	1	4.1	12	17.22	0.1
600505	2016	79	C39	1	3.66	2	19.26	0.1
600505	2017	132	C39	1	3.38	4	18.72	0.1
600505	2018	1	C39	1	3.11	3	19.4	0.1
600505	2019	2	C39	1	3.47	4	20.13	0.1
600506	2012	16	C39	1	4.98	6	17.6	0.1
600506	2013	1	C39	1	4.82	9	17.44	0.1
600506	2014	2	C39	1	4.23	11	17.15	0.1
600506	2015	24	C39	1	4.1	12	17.22	0.1
600517	2010	28	C26	1	4.09	7	19.08	0.17
600517	2011	13	C26	1	4.18	9	19.18	0.19
600517	2012	6	C26	1	2.6	14	19.85	0.17
600517	2013	22	C26	1	2.54	11	20.16	0.15
600517	2014	15	C26	1	2.99	11	20.07	0.15
600517	2015	1	C26	1	2.92	12	19.46	0.14
600517	2016	20	C26	1	0.98	12	19.41	0.15
600517	2017	55	C26	1	2.58	9	18.57	0.18
600517	2018	68	C26	1	3.97	6	17.82	0.2
600518	2010	5	C26	0	4.09	7	19.08	0.17
600518	2011	4	C26	0	4.18	9	19.18	0.19
600518	2012	7	C26	0	2.6	14	19.85	0.17
600518	2013	3	C26	0	2.54	11	20.16	0.15
600518	2014	23	C26	0	2.99	11	20.07	0.15
600518	2015	2	C26	0	2.92	12	19.46	0.14
600518	2016	1	C26	0	0.98	12	19.41	0.15
600518	2017	28	C26	0	2.58	9	18.57	0.18

600518	2018	33	C26	0	3.97	6	17.82	0.2
600520	2017	22	C35	1	4.79	7	18.77	0.11
600520	2018	14	C35	1	-4.21	8	18.8	0.15
600520	2019	32	C35	1	1.83	12	19.39	0.13
600521	2017	21	C27	1	8.32	4	18.24	0.1
600521	2018	1	C27	1	1.45	5	18.12	0.1
600521	2019	4	C27	1	5.75	5	18.05	0.1
600522	2016	11	C38	1	8.83	6	16.27	0.1
600522	2017	6	C38	1	7.61	10	16.09	0.1
600522	2018	6	C38	1	7.24	11	17	0.1
600522	2019	6	C38	1	5.45	12	17.01	0.11
600523	2017	2	C27	0	8.32	4	18.24	0.1
600523	2018	4	C27	0	1.45	5	18.24	0.1
600523	2019	1	C27	0	5.75	5	18.24	0.1
600525	2015	2	C39	0	6.56	4	17.71	0.23
600525	2016	6	C39	0	5.65	6	18.38	0.22
600525	2017	8	C39	0	6.72	8	18.13	0.18
600525	2018	7	C39	0	-0.07	8	18.39	0.19
600525	2019	3	C39	0	-6.58	8	18.96	0.19
600526	2010	1	C35	1	0.58	1	16.08	0.1
600526	2011	18	C35	1	0.74	1	17.46	0.1
600526	2012	19	C35	1	0.87	1	17.48	0.1
600526	2013	36	C35	1	1.44	1	17.5	0.1
600526	2014	5	C35	1	1.4	1	17.44	0.1
600526	2015	12	C35	1	1.53	1	19.48	0.1
600526	2016	11	C35	1	0.66	2	18.76	0.1
600526	2017	10	C35	1	-2.43	2	19.23	0.1
600526	2018	1	C35	1	-5.2	2	18.51	0.1
600526	2019	3	C35	1	1.19	2	16.97	0.1
600530	2016	7	C39	1	5.65	6	18.38	0.22
600530	2017	4	C39	1	6.72	8	18.13	0.18
600530	2018	31	C39	1	-0.07	8	17.39	0.19
600530	2019	37	C39	1	-6.58	8	17.39	0.19
600536	2014	43	I65	0	1.08	2	17.45	0.1
600536	2015	18	I65	0	2.06	2	17.68	0.1
600536	2016	35	I65	0	2.06	2	17.85	0.1
600536	2017	74	I65	0	1.09	2	18.92	0.1
600536	2018	61	I65	0	2.21	2	19.09	0.1
600536	2019	81	I65	0	2.28	2	19.2	0.1
600545	2017	1	C35	1	6.1	15	10.06	0.15
600548	2019	1	C32	1	2.44	5	19.28	0.1
600549	2018	2	C32	1	3.88	4	19.32	0.1
600549	2019	15	C32	1	2.44	5	19.28	0.1
600550	2010	13	C38	0	3.97	1	21.73	0.1
600550	2011	21	C38	0	0.39	1	21.63	0.1
600550	2012	31	C38	0	-10.36	2	20.75	0.1
600550	2013	26	C38	0	-44.84	2	20.55	0.1
600550	2014	4	C38	0	0.82	2	20.38	0.1
600550	2015	3	C38	0	1.11	2	17.78	0.1
600550	2016	63	C38	0	1.24	2	17.86	0.1
600550	2017	41	C38	0	0.76	2	17.96	0.1

600550	2018	26	C38	0	-10.26	2	17.66	0.1
600551	2012	1	C38	0	-10.36	2	20.75	0.1
600551	2013	29	C38	0	-44.84	2	20.55	0.1
600551	2014	34	C38	0	0.82	2	20.38	0.1
600551	2015	36	C38	0	1.11	2	17.78	0.1
600551	2016	32	C38	0	1.24	2	17.86	0.1
600551	2017	1	C38	0	0.76	2	17.96	0.1
600551	2018	2	C38	0	-10.26	2	17.66	0.1
600551	2019	19	C38	0	0.25	1	17.24	0.1
600558	2015	11	C33	0	2.82	2	17.35	0.15
600558	2016	19	C33	0	2.05	2	17.36	0.15
600558	2017	17	C33	0	2.1	2	17.34	0.15
600558	2018	2	C33	0	2.56	2	17.3	0.15
600558	2019	3	C33	0	2.89	2	17.42	0.15
600567	2013	2	C22	1	1.6	7	13.91	0.1
600567	2014	6	C22	1	0.6	7	13.46	0.1
600567	2015	5	C22	1	1.12	6	13.62	0.1
600567	2016	1	C22	1	1.87	6	16.6	0.12
600567	2017	3	C22	1	8.6	8	18.34	0.19
600567	2018	10	C22	1	10.94	15	18.06	0.21
600567	2019	6	C22	1	3.45	15	18.06	0.22
600570	2010	3	I65	0	15.1	3	18.68	0.1
600570	2011	7	I65	0	15.66	3	18.89	0.1
600570	2012	19	I65	0	11.81	3	18.96	0.17
600570	2013	19	I65	0	16.25	3	18.99	0.17
600570	2014	41	I65	0	13.22	3	18.49	0.17
600570	2015	102	I65	0	12.81	3	19.15	0.1
600570	2016	92	I65	0	-1.23	3	19.16	0.13
600570	2017	2	I65	0	8.33	2	19.84	0.25
600570	2018	3	I65	0	11.24	4	19.32	0.2
600570	2019	48	I65	0	19.42	2	19.88	0.15
600571	2010	84	I65	1	5.57	1	18.53	0.1
600571	2011	65	I65	1	6.33	1	18.74	0.1
600580	2013	86	C38	1	5.95	5	17.21	0.16
600580	2014	129	C38	1	5.1	6	17.13	0.15
600580	2015	117	C38	1	3.64	6	17.15	0.22
600580	2016	130	C38	1	1.91	8	16.91	0.19
600580	2017	9	C38	1	4.09	16	16.37	0.17
600580	2018	12	C38	1	3.91	10	17.03	0.2
600580	2019	7	C38	1	5.28	10	18.54	0.21
600583	2010	64	B11	1	0.54	5	16.38	0.12
600583	2011	75	B11	1	1.01	5	15.42	0.12
600583	2012	1	B11	1	4.38	5	16.46	0.12
600583	2013	2	B11	1	11.36	5	16.46	0.12
600583	2016	2	B11	1	4.29	8	19.36	0.11
600583	2017	1	B11	1	1.68	7	19.53	0.14
600583	2018	1	B11	1	0.27	8	19.44	0.13
600583	2019	61	B11	1	0.09	8	19.34	0.13
600584	2015	48	C39	1	-0.87	9	17.21	0.14
600584	2016	102	C39	1	-1.14	8	17.39	0.15
600584	2017	61	C39	1	0.24	9	17	0.16

600584	2018	9	C39	1	-2.85	8	16.99	0.16
600584	2019	6	C39	1	0.28	7	18.75	0.17
600585	2016	16	C30	0	8.31	7	19.98	0.23
600585	2017	28	C30	0	14.18	7	19.8	0.26
600585	2018	32	C30	0	22.55	8	19.8	0.24
600585	2019	9	C30	0	20.93	8	19.98	0.26
600587	2017	31	C35	0	1.25	1	19.64	0.2
600587	2018	76	C35	0	0.99	2	18.94	0.1
600587	2019	95	C35	0	7.17	3	20.03	0.1
600588	2016	2	B11	1	4.29	8	19.36	0.11
600588	2017	4	B11	1	1.68	7	19.53	0.14
600588	2018	4	B11	1	0.27	8	19.44	0.13
600588	2019	1	B11	1	0.09	8	19.34	0.13
600591	2015	3	C30	1	7.33	5	20.2	0.24
600591	2016	27	C30	1	8.31	7	19.98	0.23
600591	2017	32	C30	1	14.18	7	19.8	0.26
600591	2018	22	C30	1	22.55	8	19.8	0.24
600591	2019	44	C30	1	20.93	8	19.98	0.26
600592	2016	2	C30	1	8.31	7	19.98	0.23
600592	2017	8	C30	1	14.18	7	19.8	0.26
600592	2018	6	C30	1	22.55	8	19.8	0.24
600592	2019	6	C30	1	20.93	8	19.98	0.26
600593	2014	4	C30	0	11.86	3	20.6	0.3
600593	2015	14	C30	0	7.33	5	20.2	0.24
600593	2016	30	C30	0	8.31	7	19.98	0.23
600593	2017	31	C30	0	14.18	7	19.8	0.26
600593	2018	37	C30	0	22.55	8	19.8	0.24
600593	2019	40	C30	0	20.93	8	19.98	0.26
600594	2018	31	C30	1	22.55	8	19.8	0.24
600594	2019	1	C30	1	20.93	8	19.98	0.26
600595	2011	19	C30	1	16.38	1	19.8	0.2
600595	2012	105	C30	1	7.53	1	21.47	0.2
600595	2013	17	C30	1	10.86	2	20.89	0.15
600595	2014	18	C30	1	11.86	3	20.6	0.3
600595	2015	2	C30	1	7.33	5	20.2	0.24
600595	2016	1	C30	1	8.31	7	19.98	0.23
600595	2017	11	C30	1	14.18	7	19.8	0.26
600595	2018	3	C30	1	22.55	8	19.8	0.24
600595	2019	1	C30	1	20.93	8	19.98	0.26
600597	2019	1	C14	1	3.84	3	17.04	0.13
600606	2016	1	K70	0	1.41	3	21.71	0.13
600606	2017	13	K70	0	1.72	6	21.71	0.15
600606	2018	5	K70	0	1.7	4	22.26	0.15
600606	2019	5	K70	0	1.92	5	22.21	0.16
600609	2012	5	C36	1	1.41	2	17.96	0.1
600609	2013	3	C36	1	1.22	2	18.31	0.1
600609	2014	15	C36	1	-0.13	2	17.93	0.1
600609	2015	3	C36	1	2.04	2	18.53	0.1
600609	2016	2	C36	1	0.01	2	18.75	0.1
600609	2017	1	C36	1	4.15	2	18.59	0.1
600619	2013	4	C34	0	1.81	2	18.28	0.1

600619	2014	3	C34	0	1.64	2	18.34	0.1
600619	2015	2	C34	0	1.06	2	17.97	0.1
600619	2017	1	C34	0	2.68	2	17.48	0.1
600619	2018	1	C34	0	3	3	17.08	0.1
600619	2019	6	C34	0	2.57	4	16.8	0.1
600620	2013	1	C34	0	1.81	2	18.28	0.1
600620	2014	1	C34	0	1.64	2	18.34	0.1
600620	2015	1	C34	0	1.06	2	17.97	0.1
600620	2017	2	C34	0	2.68	2	17.48	0.1
600620	2018	5	C34	0	3	3	17.08	0.1
600620	2019	8	C34	0	2.57	4	16.8	0.1
600623	2010	7	C26	0	2.52	5	16.71	0.12
600623	2011	30	C26	0	1.16	5	16.46	0.12
600623	2012	28	C26	0	3.39	5	16.66	0.14
600623	2013	3	C26	0	2.76	5	16.6	0.12
600623	2014	6	C26	0	3.49	5	15.06	0.12
600623	2015	19	C26	0	2.24	3	20.21	0.13
600623	2016	22	C26	0	1.44	5	19.75	0.12
600623	2017	20	C26	0	1.21	3	20.37	0.13
600623	2018	30	C26	0	4.07	5	20.19	0.18
600623	2019	8	C26	0	1.34	6	20.14	0.17
600624	2010	6	C26	0	2.52	5	16.71	0.12
600624	2011	6	C26	0	1.16	5	16.46	0.12
600624	2012	8	C26	0	3.39	5	16.66	0.14
600624	2013	8	C26	0	2.76	5	16.6	0.12
600624	2014	23	C26	0	3.49	5	15.06	0.12
600624	2016	2	C26	0	1.44	5	19.75	0.12
600625	2010	2	C26	0	2.52	5	16.71	0.12
600625	2011	1	C26	0	1.16	5	16.46	0.12
600625	2012	1	C26	0	3.39	5	16.66	0.14
600625	2013	7	C26	0	2.76	5	16.6	0.12
600625	2014	11	C26	0	3.49	5	15.06	0.12
600625	2016	3	C26	0	1.44	5	19.75	0.12
600625	2019	2	C26	0	1.34	6	20.14	0.17
600626	2017	79	C17	1	2.76	12	17.69	0.3
600626	2018	108	C17	1	1.19	12	17.78	0.33
600626	2019	2	C17	1	-0.65	12	17.84	0.31
600627	2017	1	C17	1	2.76	12	17.69	0.3
600627	2018	2	C17	1	1.19	12	17.78	0.33
600627	2019	5	C17	1	-0.65	12	17.84	0.31
600628	2017	2	C17	1	2.76	12	17.69	0.3
600628	2018	120	C17	1	1.19	12	17.78	0.33
600628	2019	111	C17	1	-0.65	12	17.84	0.31
600629	2015	17	M74	1	5.57	4	13.09	0.15
600629	2016	17	M74	1	5.76	4	14.86	0.15
600629	2017	9	M74	1	4.81	4	17.51	0.15
600629	2018	32	M74	1	3.86	8	17.18	0.13
600630	2017	3	C17	1	2.76	12	17.69	0.3
600630	2018	20	C17	1	1.19	12	17.78	0.33
600630	2019	12	C17	1	-0.65	12	17.84	0.31
600631	2018	11	M74	1	3.86	8	17.18	0.13

600631	2019	6	M74	1	3.41	8	17.59	0.13
600632	2018	7	M74	1	3.86	8	17.18	0.13
600632	2019	4	M74	1	3.41	8	17.59	0.13
600633	2019	2	M74	1	3.41	8	17.59	0.13
600635	2017	1	D45	1	2.92	3	21.34	0.3
600635	2018	2	D45	1	2.03	3	21.59	0.4
600635	2019	30	D45	1	2.87	3	21.69	0.33
600641	2011	18	K70	0	1.72	3	17.32	0.17
600641	2012	50	K70	0	1.58	3	17.32	0.17
600641	2013	66	K70	0	2.57	3	17.32	0.17
600645	2010	66	M73	0	3.75	1	14.61	0.1
600645	2011	4	M73	0	5.82	1	14.29	0.1
600645	2012	4	M73	0	2.57	1	16.35	0.1
600645	2013	6	M73	0	1.24	1	16.2	0.1
600648	2018	9	F51	1	2.88	2	19.89	0.15
600648	2019	9	F51	1	2.8	2	20.24	0.15
600655	2010	3	F52	0	7.53	4	20.43	0.18
600655	2011	1	F52	0	8.04	4	20.46	0.2
600655	2012	5	F52	0	8.53	4	20.6	0.18
600655	2013	1	F52	0	8.28	4	20.59	0.18
600655	2014	1	F52	0	7.46	4	20.71	0.18
600655	2015	4	F52	1	5.25	4	20.74	0.2
600655	2016	1	F52	1	2.21	4	20.68	0.23
600655	2017	87	F52	1	2.78	3	21.06	0.3
600655	2018	105	F52	1	6.26	4	21.32	0.3
600655	2019	114	F52	1	4.23	10	20.4	0.21
600660	2011	149	C30	1	13.28	6	16.41	0.2
600660	2012	100	C30	1	12.07	6	16.67	0.17
600660	2013	94	C30	1	13.88	6	16.89	0.17
600660	2015	108	C30	1	12.51	6	15.86	0.23
600660	2016	105	C30	1	11.49	6	16.64	0.23
600660	2017	1	C30	1	10.23	6	16.58	0.23
600660	2018	1	C30	1	12.41	6	17.35	0.28
600660	2019	25	C30	1	7.91	8	17.03	0.24
600667	2017	1	E48	1	3.42	3	17.69	0.1
600667	2018	6	E48	1	3.89	3	18.06	0.1
600667	2019	6	E48	1	3.81	3	18.32	0.1
600673	2018	6	S90	0	9.04	2	17.8	0.1
600673	2019	6	S90	0	8.69	1	18.55	0.1
600679	2010	10	C37	0	0.65	1	16.98	0.1
600679	2011	26	C37	0	-0.24	1	18.61	0.1
600679	2012	39	C37	0	-0.13	1	18.63	0.1
600679	2013	22	C37	0	-0.62	1	18.71	0.1
600679	2014	35	C37	0	3.27	1	19.04	0.1
600679	2015	2	C37	0	0.28	1	18.97	0.1
600679	2016	8	C37	0	3.46	1	18.95	0.1
600679	2017	14	C37	0	5.42	1	19.02	0.1
600679	2018	13	C37	0	0.71	2	17.25	0.1
600679	2019	4	C37	0	2.25	1	17.61	0.1
600682	2017	14	F52	1	3.93	5	18.27	0.4
600682	2018	19	F52	1	-3.44	6	18.09	0.4

600682	2019	26	F52	1	8.25	6	18.09	0.25
600686	2014	18	C36	1	2.82	3	17.13	0.13
600686	2015	1	C36	1	4.32	3	17.72	0.13
600686	2016	1	C36	1	-7.49	3	17.73	0.13
600686	2017	2	C36	1	3.6	3	17.71	0.13
600686	2018	1	C36	1	0.81	3	17.66	0.13
600686	2019	1	C36	1	0.91	4	17.39	0.13
600690	2017	54	C38	0	6.4	11	20.89	0.12
600690	2018	47	C38	0	6.14	11	20.96	0.15
600690	2019	6	C38	0	6.97	4	22.36	0.13
600699	2015	5	C36	1	4.72	6	15.47	0.23
600699	2016	24	C36	1	2.78	18	15.69	0.31
600699	2017	1	C36	1	2.05	19	15.97	0.31
600699	2018	32	C36	1	4.49	30	14.74	0.35
600699	2019	7	C36	1	2.5	31	17.15	0.37
600703	2017	26	C39	1	12.96	3	17.47	0.27
600703	2018	47	C39	1	10.1	3	17.54	0.3
600703	2019	18	C39	1	4.29	4	17.23	0.23
600710	2019	27	F51	1	3.49	18	18.23	0.32
600711	2016	32	F51	1	5.28	10	17.94	0.3
600711	2017	9	F51	1	2.8	11	18.03	0.35
600711	2018	11	F51	1	2.95	13	18.58	0.32
600711	2019	2	F51	1	3.49	18	18.23	0.32
600712	2016	3	F51	1	5.28	10	17.94	0.3
600712	2017	77	F51	1	2.8	11	18.03	0.35
600712	2018	95	F51	1	2.95	13	18.58	0.32
600712	2019	82	F51	1	3.49	18	18.23	0.32
600713	2011	1	F51	1	5.55	2	19.58	0.1
600713	2012	1	F51	1	0.31	3	19.18	0.1
600713	2013	5	F51	1	-7.35	4	18.83	0.1
600713	2014	12	F51	1	-6.67	4	18.5	0.1
600713	2015	78	F51	1	-23.5	4	17.63	0.1
600713	2019	61	F51	1	3.49	18	18.23	0.32
600714	2019	2	F51	1	3.49	18	18.23	0.32
600719	2010	2	I65	1	7.76	8	18.16	0.11
600719	2011	21	I65	1	5.73	10	18.07	0.12
600719	2012	21	I65	1	5.31	11	17.38	0.13
600719	2013	28	I65	1	4.3	12	16.51	0.13
600719	2014	23	I65	1	2.57	12	16.51	0.13
600719	2015	58	I65	1	2.82	12	16.51	0.13
600719	2016	66	I65	1	14.29	8	16.79	0.11
600719	2017	3	I65	1	7.22	8	18.07	0.11
600719	2018	6	I65	1	-0.78	9	17.27	0.11
600719	2019	7	I65	1	-1.19	9	18.67	0.11
600721	2011	4	I65	0	5.73	10	18.07	0.12
600721	2012	4	I65	0	5.31	11	18.07	0.13
600721	2013	4	I65	0	4.3	12	18.07	0.13
600721	2014	12	I65	0	2.57	12	18.07	0.13
600721	2015	15	I65	0	2.82	12	18.07	0.13
600735	2013	12	C19	1	7.02	4	16.1	0.25
600735	2014	19	C19	1	8.43	4	16.02	0.25

600735	2015	4	C19	1	6.44	4	16.01	0.25
600735	2016	2	C19	1	8.73	5	15.75	0.22
600735	2017	9	C19	1	10.48	5	15.77	0.2
600735	2019	44	C19	1	9.91	6	16.54	0.2
600736	2018	5	C19	1	10.11	5	16.79	0.2
600741	2013	5	C36	1	12.38	1	22.76	0.1
600741	2014	1	C36	1	10.54	1	22.85	0.1
600741	2015	2	C36	1	9.77	1	22.91	0.1
600741	2016	3	C36	1	9.22	2	22.44	0.1
600741	2017	2	C36	1	7.91	2	22.55	0.1
600741	2018	3	C36	1	8.13	3	22.25	0.1
600741	2019	1	C36	1	6.24	2	22.67	0.1
600745	2018	16	C39	1	0.52	3	19.79	0.1
600745	2019	3	C39	1	3.36	14	14.92	0.16
600751	2017	4	F51	0	0.99	8	19.54	0.35
600751	2018	2	F51	0	0.17	9	19.39	0.31
600751	2019	10	F51	0	0.68	8	19.48	0.39
600759	2017	4	B07	1	-0.57	5	15.97	0.22
600761	2018	1	C35	0	9.8	2	18.45	0.1
600761	2019	2	C35	0	10.11	2	18.56	0.1
600796	2010	9	C26	0	5.32	1	18.98	0.1
600796	2011	11	C26	0	0.68	1	19.37	0.1
600796	2012	3	C26	0	1.64	1	19.25	0.1
600796	2013	1	C26	0	2.91	1	19.17	0.1
600796	2014	1	C26	0	2.29	1	18.08	0.1
600796	2015	2	C26	0	2.95	1	17.97	0.1
600796	2016	4	C26	0	3.83	1	17.84	0.1
600796	2017	2	C26	0	4.56	1	18.28	0.1
600796	2018	3	C26	0	-3.92	1	15.58	0.1
600796	2019	3	C26	0	2.92	1	15.41	0.1
600800	2017	11	C26	0	-10.84	1	18.85	0.1
600800	2018	8	C26	0	12.82	1	18.66	0.1
600801	2019	7	C30	1	20.11	10	17.54	0.19
600802	2015	14	C30	1	0.88	3	18.49	0.2
600802	2016	16	C30	1	2.35	3	18.49	0.2
600802	2017	1	C30	1	7.64	4	18.49	0.15
600802	2018	6	C30	1	17.92	5	18.49	0.2
600802	2019	6	C30	1	20.11	10	18.49	0.19
600804	2014	10	I64	1	3.86	2	19.17	0.1
600804	2015	6	I64	1	4.23	2	19.3	0.1
600804	2016	2	I64	1	3.88	3	18.9	0.1
600804	2017	4	I64	1	3.38	3	18.78	0.1
600804	2018	2	I64	1	1.63	4	19.35	0.45
600804	2019	7	I64	1	-29.86	6	19.08	0.33
600805	2012	4	C30	0	3.02	2	18.95	0.15
600805	2013	1	C30	0	5.68	2	18.95	0.15
600805	2015	1	C30	0	0.88	3	19.49	0.2
600805	2016	3	C30	0	2.35	3	19.49	0.2
600805	2017	5	C30	0	7.64	4	18.97	0.15
600805	2018	20	C30	0	17.92	5	18.45	0.2
600805	2019	19	C30	0	20.11	10	18.54	0.19

600819	2010	23	C30	0	3.82	2	11.42	0.15
600819	2011	31	C30	0	1.33	2	18.24	0.15
600819	2012	24	C30	0	0.6	2	18.1	0.1
600823	2017	13	K70	0	4.32	2	20.75	0.3
600823	2018	46	K70	0	4.72	2	19.86	0.3
600823	2019	57	K70	0	3.26	2	20.02	0.3
600835	2010	1	C34	0	7.68	2	20.31	0.1
600835	2012	1	C34	1	6.02	8	19.4	0.14
600835	2013	2	C34	1	6.95	8	19.46	0.15
600835	2014	12	C34	1	6.33	7	19.6	0.16
600835	2015	10	C34	1	9.39	8	19.44	0.15
600837	2015	11	J67	1	3.63	13	19.79	0.44
600837	2016	15	J67	1	1.57	15	20.18	0.46
600837	2017	21	J67	1	1.8	14	20.39	0.41
600837	2018	5	J67	1	1.04	14	19.75	0.42
600837	2019	5	J67	1	1.74	12	19.84	0.28
600838	2016	19	J67	1	1.57	15	20.18	0.46
600838	2017	12	J67	1	1.8	14	20.39	0.41
600838	2018	1	J67	1	1.04	14	19.75	0.42
600838	2019	4	J67	1	1.74	12	19.84	0.28
600839	2011	4	C39	0	0.67	8	18.15	0.1
600839	2012	3	C39	0	0.51	8	18.37	0.1
600839	2013	8	C39	0	1.34	7	18.65	0.1
600839	2015	7	C39	0	-2.98	7	18.89	0.1
600839	2016	12	C39	0	2.01	7	19.64	0.1
600839	2017	9	C39	0	1.06	15	19.06	0.15
600839	2018	29	C39	0	0.97	15	18.98	0.15
600839	2019	24	C39	0	0.46	17	18.9	0.14
600840	2010	1	C39	1	1.18	7	18.24	0.1
600840	2011	6	C39	1	0.67	8	18.15	0.1
600840	2012	8	C39	1	0.51	8	18.37	0.1
600840	2013	10	C39	1	1.34	7	18.65	0.1
600840	2014	5	C39	1	0.45	6	18.88	0.1
600840	2015	6	C39	1	-2.98	7	18.89	0.1
600840	2016	4	C39	1	2.01	7	19.64	0.1
600840	2017	4	C39	1	1.06	15	19.06	0.15
600840	2018	2	C39	1	0.97	15	18.98	0.15
600840	2019	1	C39	1	0.46	17	18.9	0.14
600841	2017	1	C39	1	1.06	15	19.06	0.15
600841	2018	8	C39	1	0.97	15	18.98	0.15
600841	2019	2	C39	1	0.46	17	18.9	0.14
600842	2010	2	C39	1	1.18	7	18.24	0.1
600842	2011	5	C39	1	0.67	8	18.15	0.1
600842	2012	2	C39	1	0.51	8	18.37	0.1
600842	2013	13	C39	1	1.34	7	18.65	0.1
600842	2014	40	C39	1	0.45	6	18.88	0.1
600842	2015	9	C39	1	-2.98	7	18.89	0.1
600842	2016	7	C39	1	2.01	7	19.64	0.1
600842	2017	5	C39	1	1.06	15	19.06	0.15
600842	2018	11	C39	1	0.97	15	18.98	0.15
600842	2019	12	C39	1	0.46	17	18.9	0.14

600843	2017	34	C35	1	5.9	2	18.74	0.15
600843	2018	2	C35	1	4.04	2	18.64	0.15
600843	2019	2	C35	1	2.3	12	15.78	0.13
600844	2017	3	C39	1	1.06	15	19.06	0.15
600844	2018	2	C39	1	0.97	15	18.98	0.15
600844	2019	3	C39	1	0.46	17	18.9	0.14
600845	2010	14	I65	1	10.15	1	17.09	0.1
600845	2011	5	I65	1	9.32	1	16.47	0.1
600845	2012	4	I65	1	8.73	1	16.78	0.1
600845	2013	1	I65	1	8.09	1	17.13	0.1
600845	2014	7	I65	1	7.03	1	16.59	0.1
600845	2015	13	I65	1	5.74	1	17.94	0.1
600845	2016	15	I65	1	5.52	1	17.91	0.1
600845	2017	15	I65	1	5.96	1	17.93	0.1
600845	2018	210	I65	1	7.89	1	18.11	0.1
600845	2019	153	I65	1	9.38	1	19.11	0.1
600846	2010	6	C39	1	1.18	7	18.24	0.1
600846	2011	7	C39	1	0.67	8	18.15	0.1
600846	2012	3	C39	1	0.51	8	18.37	0.1
600846	2013	6	C39	1	1.34	7	18.65	0.1
600846	2014	1	C39	1	0.45	6	18.88	0.1
600846	2015	6	C39	1	-2.98	7	18.89	0.1
600846	2016	33	C39	1	2.01	7	19.64	0.1
600846	2017	27	C39	1	1.06	15	19.06	0.15
600846	2018	25	C39	1	0.97	15	18.98	0.15
600846	2019	1	C39	1	0.46	17	18.9	0.14
600847	2019	1	C39	1	0.46	17	18.9	0.14
600852	2018	1	E48	1	1.05	5	16.12	0.1
600852	2019	1	E48	1	1.29	5	17.53	0.12
600853	2011	6	E48	0	0.63	1	18.82	0.1
600853	2012	7	E48	0	0.3	1	18.82	0.1
600853	2013	4	E48	0	0.28	2	18.13	0.1
600853	2018	4	E48	1	1.05	5	16.12	0.1
600853	2019	9	E48	1	1.29	5	17.53	0.12
600864	2010	14	J67	1	7.24	1	20.6	0.3
600864	2011	8	J67	1	7.32	1	17.26	0.4
600864	2012	4	J67	1	6.03	1	17.73	0.4
600864	2013	2	J67	1	6.51	1	17.06	0.4
600864	2014	18	J67	1	4.82	1	16.93	0.3
600875	2010	18	C34	1	3.45	1	19.6	0.1
600875	2011	12	C34	1	3.79	1	19.94	0.1
600875	2012	8	C34	1	2.8	1	20.2	0.1
600875	2013	19	C34	1	3.07	1	20.5	0.1
600875	2014	24	C34	1	1.62	1	20.7	0.1
600875	2015	30	C34	1	0.54	3	19.69	0.1
600875	2016	42	C34	1	-2.06	3	19.99	0.1
600875	2017	2	C34	1	0.83	3	19.96	0.1
600875	2018	4	C34	1	1.36	4	19.75	0.1
600875	2019	2	C34	1	1.53	4	19.88	0.1
600876	2015	3	C34	1	0.54	3	19.69	0.1
600876	2016	2	C34	1	-2.06	3	19.99	0.1

600876	2017	1	C34	1	0.83	3	19.96	0.1
600876	2018	11	C34	1	1.36	4	19.75	0.1
600876	2019	3	C34	1	1.53	4	19.88	0.1
600877	2018	12	C34	1	1.36	4	19.75	0.1
600877	2019	4	C34	1	1.53	4	19.88	0.1
600880	2014	3	R85	1	6.95	1	18.22	0.1
600880	2015	3	R85	1	1.37	1	18.39	0.1
600886	2019	12	D44	1	3.92	3	21.9	0.43
600887	2015	4	C14	1	11.76	5	17.01	0.12
600887	2016	2	C14	1	14.37	6	19.42	0.12
600887	2017	2	C14	1	13.56	6	19.5	0.12
600887	2018	10	C14	1	13.32	6	19.58	0.12
600887	2019	11	C14	1	12.86	10	19.09	0.19
600888	2017	53	C39	1	1.21	1	20.41	0.1
600888	2018	5	C39	1	1.8	1	20.7	0.1
600888	2019	4	C39	1	1.28	1	20.81	0.1
600962	2018	6	C15	1	0.54	3	13.74	0.17
600962	2019	6	C15	1	0.72	3	13.74	0.17
600970	2012	7	E48	1	3.79	1	18.51	0.1
600970	2013	8	E48	1	0.32	4	16.57	0.1
600970	2014	15	E48	1	0.23	4	15.46	0.1
600981	2014	10	F51	0	0.44	2	16.2	0.1
600981	2017	9	F51	1	2.82	2	18.59	0.3
600981	2018	17	F51	1	4.59	4	19.58	0.28
600981	2019	1	F51	1	1.91	4	19.59	0.23
600999	2017	3	J67	1	2.2	6	20.95	0.23
600999	2018	1	J67	1	1.51	6	21.05	0.22
600999	2019	1	J67	1	2.13	6	21.11	0.22
601008	2010	2	G55	0	4.01	1	20.3	0.1
601008	2011	2	G55	0	3.98	1	20.35	0.1
601008	2012	1	G55	0	3.63	1	20.56	0.1
601008	2013	5	G55	0	2.94	1	20.22	0.1
601008	2014	6	G55	0	1.6	1	20.24	0.1
601008	2015	12	G55	0	0.78	1	20.25	0.1
601008	2016	9	G55	0	0.06	1	20.3	0.1
601008	2017	1	G55	0	0.07	1	20.59	0.1
601008	2018	4	G55	0	0.23	1	20.33	0.1
601008	2019	8	G55	0	0.36	1	20.34	0.1
601012	2016	11	C38	1	10.56	5	17.66	0.1
601012	2017	12	C38	1	13.64	8	17.98	0.15
601012	2018	20	C38	1	7.08	8	18.33	0.11
601012	2019	56	C38	1	11.23	8	18.72	0.11
601014	2016	35	C38	0	10.56	5	17.66	0.1
601014	2017	21	C38	0	13.64	8	17.98	0.15
601014	2018	17	C38	0	7.08	8	17.33	0.11
601014	2019	18	C38	0	11.23	8	17.66	0.11
601021	2014	27	G56	0	9.35	3	17.42	0.1
601021	2015	2	G56	0	9.73	3	17.27	0.1
601021	2016	10	G56	0	5.33	3	14.89	0.1
601021	2017	6	G56	0	6.27	3	15.75	0.1
601021	2018	22	G56	0	6.37	3	16.17	0.1

601028	2015	16	F51	0	3.54	2	15.31	0.15
601028	2016	51	F51	0	-20.27	2	15.08	0.15
601038	2017	33	C35	1	0.28	3	17.67	0.1
601038	2018	37	C35	1	-10.02	3	17.59	0.13
601038	2019	37	C35	1	1.02	3	17.5	0.1
601058	2012	30	C29	1	2.66	5	18.38	0.12
601058	2013	22	C29	1	3.01	6	18.36	0.12
601058	2018	20	C29	1	4.33	8	17.18	0.14
601058	2019	14	C29	1	7.18	9	17.68	0.14
601059	2012	14	C29	0	2.66	5	18.38	0.12
601059	2013	23	C29	0	3.01	6	18.38	0.12
601068	2018	37	E48	1	1.08	4	18.6	0.1
601068	2019	4	E48	1	0.42	4	19.08	0.1
601069	2018	36	E48	1	1.08	4	18.6	0.1
601069	2019	48	E48	1	0.42	4	19.08	0.1
601088	2011	1	B06	0	13.98	2	21.37	0.15
601088	2012	7	B06	0	13.1	3	21.34	0.13
601088	2013	2	B06	0	11.59	3	21.39	0.13
601088	2014	1	B06	0	8.92	3	21.22	0.13
601088	2015	5	B06	0	4.28	3	21.24	0.13
601088	2016	3	B06	0	5.25	3	21.25	0.13
601088	2017	3	B06	0	9.49	3	21.87	0.2
601088	2018	1	B06	0	9.36	3	21.93	0.17
601088	2019	23	B06	0	9	3	23.33	0.13
601099	2013	27	J67	0	1.64	1	17.82	0.1
601099	2014	1	J67	0	6.08	1	17.17	0.1
601099	2015	4	J67	0	4.7	2	16.46	0.2
601099	2016	6	J67	0	1.79	2	17.35	0.2
601099	2017	3	J67	0	0.29	3	18.71	0.17
601099	2019	1	J67	0	1.3	1	19.92	0.1
601117	2010	11	E48	0	5.3	2	19.66	0.1
601117	2011	15	E48	0	6.3	2	19.73	0.1
601117	2012	20	E48	0	6.18	2	19.92	0.1
601117	2013	1	E48	0	5.32	2	19.92	0.1
601117	2014	1	E48	0	4.26	2	18.22	0.1
601117	2015	3	E48	0	3.63	2	18.23	0.1
601117	2016	3	E48	0	2.14	3	17.96	0.1
601117	2017	3	E48	0	1.86	2	19.05	0.15
601117	2018	3	E48	0	2.25	4	19.31	0.15
601117	2019	4	E48	0	3.09	4	19.8	0.15
601118	2010	9	A01	0	6.06	2	15.92	0.1
601118	2011	9	A01	0	6.45	2	17.9	0.1
601118	2012	6	A01	0	2.74	3	17.86	0.1
601118	2013	3	A01	0	1.4	3	18.03	0.1
601118	2014	9	A01	0	0.24	2	18.05	0.1
601118	2015	17	A01	1	-7.81	4	17.81	0.13
601118	2016	32	A01	1	0.48	4	19.1	0.13
601118	2017	1	A01	1	-2.04	4	19.1	0.13
601118	2018	1	A01	1	1.57	4	19.08	0.15
601118	2019	1	A01	1	0.73	10	18.29	0.18
601119	2019	6	E48	1	3.09	4	19.8	0.15

601121	2019	20	A01	1	0.73	10	18.29	0.18
601122	2019	1	A01	1	0.73	10	18.29	0.18
601123	2019	5	A01	1	0.73	10	18.29	0.18
601126	2015	26	C38	1	6.36	1	18.92	0.19
601126	2016	2	C38	1	5.11	1	19.29	0.19
601126	2017	1	C38	1	4.17	1	19.15	0.19
601126	2018	59	C38	1	3.61	2	18.66	0.19
601126	2019	3	C38	1	3.13	2	18.31	0.18
601127	2016	104	C36	1	3.94	3	18.96	0.19
601127	2017	2	C36	1	5.06	3	19	0.19
601127	2018	47	C36	1	2.02	4	19.02	0.19
601127	2019	9	C36	1	0.3	4	18.92	0.19
601179	2010	124	C38	0	2.72	1	19.49	0.19
601179	2011	52	C38	0	-2.14	1	19.39	0.19
601179	2012	1	C38	0	0.31	1	19.75	0.2
601179	2013	8	C38	0	0.97	2	19.83	0.2
601179	2014	32	C38	0	2.09	2	19.76	0.2
601179	2015	13	C38	0	2.84	2	19.76	0.2
601179	2016	9	C38	0	3.37	2	18.54	0.19
601179	2017	39	C38	0	2.55	2	18.49	0.18
601180	2013	42	C38	0	0.97	2	19.83	0.2
601180	2014	42	C38	0	2.09	2	19.76	0.2
601180	2015	0	C38	0	2.84	2	19.76	0.2
601180	2016	0	C38	1	3.37	2	18.54	0.19
601180	2017	0	C38	1	2.55	2	18.49	0.18
601211	2019	0	J67	1	1.82	6	19.83	0.37
601222	2019	0	C40	0	4.03	4	18.36	0.18
601231	2019	0	C39	1	5.99	6	20	0.2
601233	2013	11	C28	1	0.69	1	16.08	0.16
601233	2014	135	C28	1	0.89	1	16.08	0.16
601233	2015	115	C28	1	0.83	1	16.08	0.16
601238	2017	0	C36	1	10.73	2	23.97	0.24
601238	2019	0	C36	1	4.98	2	24.19	0.24
601288	2015	9	J66	1	1.07	5	17.82	0.12
601288	2017	0	J66	1	0.95	5	17.63	0.12
601288	2018	0	J66	1	0.93	5	20.5	0.12
601288	2019	0	J66	1	0.9	5	21.01	0.12
601339	2012	0	C17	1	3.29	3	19.24	0.2
601339	2013	0	C17	1	5.78	3	19.24	0.2
601339	2016	0	C17	1	5.34	3	19.4	0.23
601339	2017	0	C17	1	4.2	3	20.24	0.23
601339	2018	0	C17	1	3.48	3	20.27	0.23
601339	2019	1	C17	1	2.14	3	20.33	0.23
601369	2015	0	C38	0	2.33	1	19.12	0.19
601369	2016	0	C38	0	1.53	2	19.21	0.19
601369	2017	0	C38	0	1.62	2	19.2	0.19
601369	2018	0	C38	0	2.2	2	19.14	0.19
601369	2019	19	C38	0	3.46	2	19.1	0.19
601370	2016	17	C38	1	1.53	2	19.21	0.19
601370	2017	21	C38	1	1.62	2	19.2	0.19
601370	2018	49	C38	1	2.2	2	19.14	0.19

601370	2019	46	C38	1	3.46	2	19.1	0.19
601390	2012	28	E48	1	1.58	4	21.4	0.1
601398	2010	152	J66	1	1.32	15	21.71	0.13
601398	2011	4	J66	1	1.44	15	21.5	0.13
601398	2012	2	J66	1	1.45	14	21.59	0.11
601398	2013	4	J66	1	1.44	17	21.24	0.11
601398	2014	8	J66	1	1.4	16	21.32	0.11
601398	2015	10	J66	1	1.3	17	21.08	0.12
601398	2016	6	J66	1	1.2	18	21.24	0.12
601398	2017	7	J66	1	1.14	18	21.31	0.12
601398	2018	11	J66	1	1.11	18	21.2	0.12
601398	2019	10	J66	1	1.08	19	21.26	0.12
601399	2010	10	J66	1	1.32	15	21.71	0.13
601399	2011	0	J66	1	1.44	15	21.5	0.13
601399	2012	0	J66	1	1.45	14	21.59	0.11
601399	2013	0	J66	1	1.44	17	21.24	0.11
601399	2014	1	J66	1	1.4	16	21.32	0.11
601399	2015	4	J66	1	1.3	17	21.08	0.12
601399	2016	1	J66	1	1.2	18	21.24	0.12
601399	2017	9	J66	1	1.14	18	21.31	0.12
601399	2018	29	J66	1	1.11	18	21.2	0.12
601399	2019	48	J66	1	1.08	19	21.26	0.12
601400	2010	100	J66	1	1.32	15	21.71	0.13
601400	2011	98	J66	1	1.44	15	21.5	0.13
601400	2012	68	J66	1	1.45	14	21.59	0.11
601400	2013	7	J66	1	1.44	17	21.24	0.11
601400	2014	1	J66	1	1.4	16	21.32	0.11
601400	2015	8	J66	1	1.3	17	21.08	0.12
601400	2016	3	J66	1	1.2	18	21.24	0.12
601400	2017	3	J66	1	1.14	18	21.31	0.12
601400	2018	3	J66	1	1.11	18	21.2	0.12
601400	2019	0	J66	1	1.08	19	21.26	0.12
601401	2015	0	J66	0	1.3	17	21.08	0.12
601401	2016	0	J66	0	1.2	18	21.24	0.12
601401	2017	0	J66	0	1.14	18	21.31	0.12
601401	2018	2	J66	0	1.11	18	21.2	0.12
601401	2019	0	J66	0	1.08	19	21.26	0.12
601402	2010	0	J66	1	1.32	15	21.71	0.13
601402	2011	40	J66	1	1.44	15	21.5	0.13
601402	2012	36	J66	1	1.45	14	21.59	0.11
601402	2013	87	J66	1	1.44	17	21.24	0.11
601402	2014	101	J66	1	1.4	16	21.32	0.11
601402	2015	39	J66	1	1.3	17	21.08	0.12
601402	2016	24	J66	1	1.2	18	21.24	0.12
601402	2017	55	J66	1	1.14	18	21.31	0.12
601402	2018	103	J66	1	1.11	18	21.2	0.12
601402	2019	178	J66	1	1.08	19	21.26	0.12
601500	2018	194	C29	1	3.51	3	13.8	0.1
601500	2019	188	C29	1	1.83	3	18.71	0.1
601519	2014	1	J69	1	7.2	7	17.18	0.17
601519	2015	0	J69	1	-14.49	6	14.29	0.17

601519	2016	0	J69	1	-77.47	6	13.79	0.18
601519	2017	0	J69	1	20.98	6	17.95	0.18
601567	2016	0	C40	1	8.31	2	19.94	0.2
601567	2017	0	C40	1	7.33	2	20.13	0.2
601567	2018	0	C40	1	3.77	2	21.08	0.21
601567	2019	0	C40	1	7.5	2	21.25	0.21
601598	2018	30	G58	1	5.17	7	20.72	0.13
601598	2019	37	G58	1	4.74	9	20.76	0.12
601599	2018	39	G58	1	5.17	7	20.72	0.13
601599	2019	99	G58	1	4.74	9	20.76	0.12
601600	2019	66	G58	1	4.74	9	20.76	0.12
601608	2015	15	C35	1	0.31	5	18.11	0.18
601608	2016	21	C35	1	-7.72	6	18.64	0.19
601608	2017	37	C35	1	0.33	6	18.64	0.19
601608	2018	78	C35	1	0.77	7	18.78	0.19
601608	2019	93	C35	1	0.68	7	19.48	0.19
601609	2015	106	C35	1	0.31	5	18.11	0.18
601609	2016	0	C35	1	-7.72	6	18.64	0.19
601609	2017	0	C35	1	0.33	6	18.64	0.19
601609	2018	0	C35	1	0.77	7	18.78	0.19
601609	2019	0	C35	1	0.68	7	19.48	0.19
601610	2016	0	C35	0	-7.72	6	18.64	0.19
601610	2017	1	C35	0	0.33	6	18.64	0.19
601610	2018	1	C35	0	0.77	7	18.78	0.19
601610	2019	5	C35	0	0.68	7	19.48	0.19
601611	2019	0	E48	1	1.3	9	18.48	0.12
601612	2018	0	C35	0	0.77	7	18.78	0.19
601612	2019	0	C35	0	0.68	7	19.48	0.19
601613	2019	0	E48	1	1.3	9	18.48	0.12
601614	2019	0	E48	0	1.3	9	18.48	0.12
601615	2018	0	C34	0	2.04	8	17.74	0.14
601615	2019	2	C34	0	2.32	6	18.07	0.12
601616	2019	3	E48	1	1.3	9	18.48	0.12
601617	2019	2	E48	0	1.3	9	18.48	0.12
601618	2017	4	E48	1	1.69	7	20.97	0.11
601618	2018	19	E48	1	1.77	5	21.74	0.12
601618	2019	16	E48	1	1.69	5	22.2	0.12
601620	2017	16	E48	0	1.69	7	20.97	0.11
601633	2011	16	C36	1	12.35	3	16.97	0.13
601633	2012	35	C36	1	15.12	3	16.45	0.1
601633	2013	29	C36	1	17.3	3	16.69	0.1
601633	2014	53	C36	1	14.11	4	16.62	0.2
601633	2015	3	C36	1	12.1	5	15.1	0.18
601633	2019	5	C36	1	4.03	10	19.56	0.17
601668	2018	1	E48	1	3.24	6	23.12	0.33
601668	2019	6	E48	1	3.24	7	23.09	0.41
601717	2011	8	C36	1	12.54	1	18.12	0.18
601717	2012	0	C36	1	13.84	1	19.88	0.2
601717	2013	0	C36	1	6.53	2	19.82	0.2
601717	2014	0	C36	1	1.56	2	19.83	0.2
601717	2015	1	C36	1	0.09	4	19.86	0.2

601717	2016	0	C36	1	0.31	4	19.88	0.2
601717	2017	0	C36	1	2.2	4	20	0.2
601717	2018	0	C36	1	4.01	16	19.89	0.2
601717	2019	0	C36	1	3.97	16	19.69	0.2
601727	2010	0	C34	0	4.15	4	20.51	0.25
601727	2011	0	C34	0	4.39	6	20.14	0.17
601727	2012	0	C34	0	3.96	12	19.55	0.18
601727	2013	0	C34	0	3.57	5	20.48	0.14
601729	2011	0	C34	1	4.39	6	20.14	0.17
601729	2012	0	C34	1	3.96	12	19.55	0.18
601729	2017	0	C34	1	2.67	4	21.71	0.15
601729	2018	2	C34	1	2.62	4	21.94	0.13
601730	2011	0	C34	1	4.39	6	20.14	0.17
601730	2012	1	C34	1	3.96	12	19.55	0.18
601766	2013	6	C37	0	4.48	3	20.72	0.1
601766	2014	8	C37	0	4.88	3	20.59	0.13
601800	2011	3	E48	0	3.52	4	21.18	0.1
601800	2012	0	E48	0	2.88	6	20.82	0.1
601800	2013	0	E48	0	2.49	5	21.38	0.1
601800	2014	0	E48	0	2.4	5	21.42	0.1
601800	2015	0	E48	0	2.32	5	21.67	0.14
601800	2016	23	E48	0	2.25	4	22.29	0.13
601800	2017	35	E48	0	2.58	6	22.36	0.12
601800	2018	45	E48	0	2.24	10	22.15	0.13
601800	2019	38	E48	0	2.08	9	22.48	0.14
601801	2011	4	E48	1	3.52	4	21.18	0.1
601801	2012	4	E48	1	2.88	6	20.82	0.1
601801	2013	5	E48	1	2.49	5	21.38	0.1
601801	2014	1	E48	1	2.4	5	21.42	0.1
601801	2015	1	E48	1	2.32	5	21.67	0.14
601801	2017	0	E48	1	2.58	6	22.36	0.12
601801	2018	2	E48	1	2.24	10	22.15	0.13
601801	2019	2	E48	1	2.08	9	22.48	0.14
601808	2010	4	B11	1	6.63	8	17.91	0.19
601808	2011	7	B11	1	6.28	8	17.95	0.2
601808	2012	0	B11	1	6.54	8	18.07	0.21
601808	2013	3	B11	1	8.74	9	18.18	0.14
601808	2014	2	B11	1	9.05	10	18.13	0.14
601808	2015	4	B11	1	1.23	10	18.04	0.14
601808	2016	11	B11	1	-13.17	10	17.91	0.13
601808	2017	9	B11	1	0.09	13	17.62	0.17
601808	2018	12	B11	1	0.12	12	17.85	0.18
601808	2019	9	B11	1	3.35	12	18.11	0.15
601809	2010	0	B11	0	6.63	8	17.91	0.19
601809	2011	0	B11	0	6.28	8	17.95	0.2
601809	2012	9	B11	0	6.54	8	18.07	0.21
601869	2018	1	C39	0	13.49	4	21.21	0.21
601869	2019	0	C39	0	5.88	7	21.13	0.21
601870	2018	1	C39	1	13.49	4	21.21	0.21
601870	2019	7	C39	1	5.88	7	22.13	0.21
601877	2011	4	C38	1	12.6	4	19.87	0.2

601877	2012	1	C38	1	15.35	4	19.84	0.2
601877	2013	0	C38	1	16.18	4	20.11	0.2
601877	2016	0	C38	0	11.03	2	20.22	0.2
601877	2019	0	C38	0	7.72	1	21.78	0.22
601886	2012	13	E50	1	4.71	15	14.89	0.1
601886	2013	13	E50	1	2.05	18	17.38	0.1
601886	2014	19	E50	1	2.17	18	12.09	0.22
601886	2015	5	E50	1	1.63	12	12.49	0.27
601888	2013	0	L72	1	13.56	14	16.49	0.14
601888	2014	0	L72	1	12.08	14	16.46	0.14
601888	2015	3	L72	1	11.31	15	16.55	0.14
601888	2016	6	L72	1	12.33	16	16.55	0.14
601889	2016	17	E50	1	1.97	8	15.14	0.45
601889	2017	12	E50	1	2.39	8	16.11	0.49
601889	2019	8	E50	1	1.93	7	16.1	0.66
601892	2013	5	L72	0	13.56	14	16.49	0.14
601892	2014	16	L72	0	12.08	14	16.46	0.14
601892	2015	15	L72	0	11.31	15	16.55	0.14
601892	2016	0	L72	0	12.33	16	16.55	0.14
601892	2017	1	L72	0	15.36	4	18.14	0.18
601893	2010	12	L72	1	9.26	10	16.64	0.14
601893	2012	26	L72	1	14.2	12	16.37	0.15
601893	2013	98	L72	1	13.56	14	16.49	0.14
601893	2014	160	L72	1	12.08	14	16.46	0.14
601893	2015	84	L72	1	11.31	15	16.55	0.14
601893	2016	120	L72	1	12.33	16	16.55	0.14
601894	2015	0	L72	1	11.31	15	16.55	0.14
601894	2016	4	L72	1	12.33	16	16.55	0.14
601899	2010	0	B09	1	16.92	5	20.1	0.2
601899	2011	33	B09	1	15.23	6	20.1	0.2
601899	2016	15	B09	1	1.95	5	21.18	0.18
601899	2017	18	B09	1	3.64	9	20.44	0.13
601899	2018	9	B09	1	4.63	13	20.11	0.13
601899	2019	12	B09	1	4.28	5	21.05	0.22
601900	2011	15	B09	0	15.23	6	20.1	0.2
601900	2018	8	B09	0	4.63	13	20.11	0.13
601901	2010	9	B09	1	16.92	5	20.1	0.2
601901	2011	52	B09	1	15.23	6	20.1	0.2
601901	2018	43	B09	1	4.63	13	20.11	0.13
601901	2019	52	B09	1	4.28	5	21.05	0.22
601902	2010	37	B09	0	16.92	5	20.1	0.2
601902	2011	23	B09	0	15.23	6	20.1	0.2
601902	2017	30	B09	0	3.64	9	20.44	0.13
601902	2018	33	B09	0	4.63	13	20.11	0.13
601919	2010	20	G55	0	5.52	16	20.63	0.22
601919	2011	21	G55	0	-5.73	16	20.68	0.23
601919	2012	0	G55	0	-5.04	16	20.74	0.23
601919	2013	0	G55	0	1.76	15	20.49	0.21
601919	2014	0	G55	0	1	23	20.01	0.19
601919	2015	1	G55	0	1.21	20	20.46	0.21
601919	2016	0	G55	0	-6.8	20	20.74	0.19

601919	2017	0	G55	0	3.82	21	20.93	0.18
601919	2018	0	G55	0	1.68	19	21.14	0.18
601920	2014	5	G55	0	1	23	20.01	0.19
601921	2010	8	G55	1	5.52	16	20.63	0.22
601921	2011	5	G55	1	-5.73	16	20.68	0.23
601921	2012	7	G55	1	-5.04	16	20.74	0.23
601921	2013	12	G55	1	1.76	15	20.49	0.21
601921	2014	6	G55	1	1	23	20.01	0.19
601921	2015	4	G55	1	1.21	20	20.46	0.21
601921	2016	0	G55	1	-6.8	20	20.74	0.19
601921	2017	0	G55	1	3.82	21	20.93	0.18
601921	2018	0	G55	1	1.68	19	21.14	0.18
601921	2019	6	G55	1	4.22	14	21.51	0.17
601922	2014	2	G55	0	1	23	20.01	0.19
601923	2010	6	G55	1	5.52	16	20.63	0.22
601923	2011	11	G55	1	-5.73	16	20.68	0.23
601923	2012	21	G55	1	-5.04	16	20.74	0.23
601923	2013	23	G55	1	1.76	15	20.49	0.21
601923	2014	1	G55	1	1	23	20.01	0.19
601923	2015	2	G55	1	1.21	20	20.46	0.21
601923	2016	2	G55	1	-6.8	20	20.74	0.19
601923	2017	2	G55	1	3.82	21	20.93	0.18
601923	2018	1	G55	1	1.68	19	21.14	0.18
601923	2019	1	G55	1	4.22	14	21.51	0.17
601924	2013	1	G55	0	1.76	15	20.49	0.21
601924	2014	1	G55	0	1	23	20.01	0.19
601924	2015	2	G55	0	1.21	20	20.46	0.21
601924	2016	1	G55	0	-6.8	20	20.74	0.19
601924	2017	1	G55	0	3.82	21	20.93	0.18
601925	2014	1	G55	0	1	23	20.01	0.19
601925	2017	0	G55	0	3.82	21	20.93	0.18
601925	2018	13	G55	0	1.68	19	21.14	0.18
601926	2014	11	G55	0	1	23	20.01	0.19
601926	2015	11	G55	0	1.21	20	20.46	0.21
601926	2016	0	G55	0	-6.8	20	20.74	0.19
601926	2017	0	G55	0	3.82	21	20.93	0.18
601926	2018	0	G55	0	1.68	19	21.14	0.18
601927	2013	0	G55	0	1.76	15	20.49	0.21
601927	2014	0	G55	0	1	23	20.01	0.19
601927	2015	0	G55	0	1.21	20	20.46	0.21
601927	2016	0	G55	0	-6.8	20	20.74	0.19
601927	2017	0	G55	0	3.82	21	20.93	0.18
601927	2018	1	G55	0	1.68	19	21.14	0.18
601928	2013	0	R85	1	6.77	3	17.93	0.13
601928	2014	0	R85	1	7.86	10	16.7	0.16
601928	2015	0	R85	1	6.64	10	17.1	0.17
601928	2016	0	R85	1	6.5	10	18.15	0.18
601928	2017	3	R85	1	6.07	10	18.27	0.21
601928	2018	3	R85	1	6.56	9	18.54	0.21
601928	2019	2	R85	1	6.2	8	18.69	0.16
601933	2016	3	F52	1	4.88	2	20.71	0.1

601933	2017	4	F52	1	5.41	2	21.33	0.2
601933	2018	4	F52	1	2.75	2	21.77	0.2
601933	2019	4	F52	1	3.16	2	21.8	0.2
601939	2013	0	J66	1	1.47	7	19.74	0.17
601939	2014	4	J66	1	1.42	9	19.65	0.17
601939	2015	3	J66	1	1.3	8	20.25	0.14
601939	2016	4	J66	1	1.18	8	20.63	0.15
601939	2017	2	J66	1	1.13	9	20.48	0.14
601939	2018	13	J66	1	1.13	9	20.61	0.14
601939	2019	0	J66	1	1.11	9	20.96	0.14
601940	2017	0	J66	0	1.13	9	20.48	0.14
601940	2018	0	J66	0	1.13	9	20.61	0.14
601940	2019	0	J66	0	1.11	9	20.96	0.14
601968	2017	0	C33	1	0.29	3	12.1	0.13
601968	2018	8	C33	1	0.78	3	12.41	0.13
601968	2019	10	C33	1	2.51	3	12.23	0.13
601989	2018	2	C37	1	-0.09	3	21.68	0.1
601989	2019	4	C37	1	0.25	3	21.78	0.1
601991	2010	8	D44	0	1.9	2	22.27	0.1
601991	2011	4	D44	0	1.31	2	22.46	0.1
601991	2012	6	D44	0	2.4	2	22.84	0.1
601991	2013	15	D44	0	2.04	2	22.78	0.1
601991	2014	13	D44	0	0.65	3	22.18	0.1
601991	2015	5	D44	0	1.08	3	22.21	0.1
601991	2016	7	D44	0	0.75	3	22.32	0.1
601991	2017	5	D44	0	1.04	3	22.34	0.1
601991	2018	0	D44	0	1.07	2	22.83	0.25
601991	2019	0	D44	0	1.04	2	22.87	0.15
601998	2018	0	J66	0	0.77	2	21.39	0.25
601998	2019	0	J66	0	0.76	2	21.33	0.2
603000	2012	119	I64	1	13.15	7	14.12	0.1
603000	2013	0	I64	1	11.11	8	15.04	0.1
603000	2014	0	I64	1	14.14	10	14.75	0.11
603000	2015	0	I64	1	11.02	10	16.24	0.11
603000	2016	0	I64	1	5.57	10	16.82	0.11
603000	2017	0	I64	1	2.93	10	17.63	0.12
603000	2018	0	I64	1	6.74	13	17.47	0.12
603000	2019	0	I64	1	9.86	12	17.53	0.13
603001	2012	17	I64	1	13.15	7	14.12	0.1
603001	2013	17	I64	1	11.11	8	15.04	0.1
603001	2014	25	I64	1	14.14	10	14.75	0.11
603001	2015	28	I64	1	11.02	10	16.24	0.11
603001	2016	38	I64	1	5.57	10	16.82	0.11
603001	2017	49	I64	1	2.93	10	17.63	0.12
603001	2018	2	I64	1	6.74	13	17.47	0.12
603001	2019	9	I64	1	9.86	12	17.53	0.13
603002	2012	6	I64	1	13.15	7	14.12	0.1
603002	2013	4	I64	1	11.11	8	15.04	0.1
603002	2014	5	I64	1	14.14	10	14.75	0.11
603002	2015	8	I64	1	11.02	10	16.24	0.11
603002	2016	6	I64	1	5.57	10	16.82	0.11

603002	2017	9	I64	1	2.93	10	17.63	0.12
603002	2018	7	I64	1	6.74	13	17.47	0.12
603002	2019	3	I64	1	9.86	12	17.53	0.13
603008	2018	5	C21	1	-7.2	2	15.59	0.15
603013	2018	3	C36	1	6.15	8	18.7	0.19
603013	2019	1	C36	1	6.45	8	18.67	0.19
603014	2018	1	C36	1	6.15	8	18.7	0.19
603014	2019	1	C36	1	6.45	8	18.67	0.19
603015	2015	0	C38	1	7	1	15.17	0.15
603015	2016	0	C38	1	3.64	2	15.1	0.15
603015	2017	0	C38	1	4.82	2	15.54	0.16
603015	2019	0	C38	1	1.97	2	16.71	0.17
603016	2018	0	C36	1	6.15	8	18.7	0.19
603016	2019	0	C36	1	6.45	8	18.67	0.19
603030	2019	0	E50	1	3.1	5	15.43	0.12
603031	2019	0	C41	0	6.89	2	14.47	0.1
603077	2017	0	C26	0	3.88	1	19.13	0.19
603096	2017	0	R85	1	18.38	4	16.51	0.1
603096	2018	0	R85	1	13.06	4	16.64	0.1
603096	2019	0	R85	1	11.97	4	16.93	0.13
603118	2019	0	C39	1	3.69	3	17.31	0.17
603126	2019	0	M74	1	3.94	2	15.27	0.15
603167	2014	0	G55	0	6.2	2	16.8	0.15
603167	2015	2	G55	0	4.73	2	16.78	0.2
603167	2016	0	G55	0	6.03	2	16.8	0.15
603167	2017	3	G55	0	9.31	2	16.83	0.2
603167	2018	6	G55	0	9.05	2	16.92	0.25
603167	2019	11	G55	0	8.43	2	16.97	0.25
603177	2018	20	N77	1	1.02	1	16.8	0.1
603177	2019	0	N77	1	0.29	2	16.04	0.1
603178	2019	0	N77	0	0.29	2	16.04	0.1
603180	2019	0	C21	0	10.69	5	13.54	0.1
603197	2017	0	C36	1	11.14	3	16.68	0.17
603197	2018	5	C36	1	7.33	4	16.36	0.16
603197	2019	2	C36	1	4.67	4	16.69	0.17
603259	2018	3	M73	1	13.24	8	18.22	0.48
603259	2019	3	M73	1	7.36	8	18.41	0.43
603269	2018	3	C34	1	2.52	4	16.12	0.13
603269	2019	2	C34	1	3.43	5	15.97	0.12
603277	2019	4	C34	1	12.11	9	14.14	0.14
603298	2017	6	C34	0	10.75	3	16.77	0.1
603298	2018	9	C34	0	11.35	3	17.84	0.1
603313	2017	7	C21	0	7.14	6	15.34	0.1
603313	2018	26	C21	0	6.92	5	16.12	0.1
603313	2019	1	C21	0	9.81	6	15.92	0.1
603314	2019	10	C21	1	9.81	6	15.92	0.1
603335	2017	11	C36	0	1.91	3	16.33	0.16
603355	2016	30	C38	1	11.36	1	16.78	0.17
603355	2017	25	C38	1	7.12	1	16.68	0.17
603355	2018	29	C38	1	8.77	1	16.36	0.16
603363	2019	0	C13	1	2.44	2	17.05	0.1

603366	2012	0 C38	1	12.97	1	13.82	0.14
603366	2013	0 C38	1	6.73	1	19.24	0.19
603366	2014	0 C38	1	4.73	2	15.31	0.15
603366	2015	2 C38	1	5.79	3	15.29	0.15
603367	2019	5 C27	1	9.55	2	17.57	0.18
603379	2019	0 C26	1	14.52	1	18.73	0.19
603444	2019	1 I64	1	25.46	2	18.9	0.2
603486	2018	0 C38	1	14.04	7	18.72	0.19
603486	2019	0 C38	1	2.84	7	18.98	0.19
603501	2019	0 C39	1	6.39	4	17.01	0.17
603515	2018	8 C39	1	13.19	7	18.71	0.19
603517	2019	6 C13	1	17.04	5	19.23	0.12
603533	2017	1 I64	1	10.65	2	16.67	0.1
603533	2018	2 I64	1	9.25	2	17.93	0.1
603533	2019	6 I64	1	8.38	2	18.52	0.1
603538	2017	6 C27	1	4.05	1	19.29	0.19
603538	2018	7 C27	1	5.55	1	18.55	0.19
603556	2016	0 C40	1	13.62	11	16.7	0.17
603556	2017	0 C40	1	9.59	11	16.69	0.17
603556	2018	0 C40	1	5.13	11	17.22	0.17
603556	2019	0 C40	1	7.6	13	17.2	0.17
603558	2016	0 C40	0	13.62	11	16.7	0.17
603558	2017	0 C40	0	9.59	11	17.27	0.17
603558	2018	0 C40	0	5.13	11	17.22	0.17
603558	2019	0 C40	0	7.6	13	17.3	0.17
603588	2019	0 N77	1	4.85	2	19.68	0.1
603605	2018	4 C26	1	11.1	1	15.79	0.16
603605	2019	2 C26	1	12.55	1	16.51	0.17
603609	2014	2 C13	0	7.15	2	18.77	0.1
603609	2015	0 C13	0	8.34	2	18.93	0.1
603609	2016	0 C13	0	9.97	2	19.32	0.1
603609	2017	5 C13	0	8.96	2	20.05	0.1
603609	2018	8 C13	0	9.36	5	19.42	0.1
603609	2019	73 C13	0	18.62	4	20.06	0.13
603611	2014	43 C34	1	9.06	4	16.34	0.1
603611	2017	39 C34	1	6.11	6	15.74	0.12
603611	2018	14 C34	1	6.14	5	17.24	0.1
603611	2019	12 C34	1	5.76	5	17.31	0.1
603628	2016	11 C41	1	5.64	7	14.16	0.11
603628	2017	6 C41	1	2.96	8	13.47	0.16
603628	2018	6 C41	1	1.17	8	13.76	0.2
603628	2019	9 C41	1	-2.03	8	14.41	0.25
603630	2016	1 C41	0	5.64	7	14.16	0.11
603630	2017	3 C41	0	2.96	8	14.47	0.16
603630	2018	5 C41	0	1.17	8	14.38	0.2
603630	2019	2 C41	0	-2.03	8	15.41	0.25
603636	2019	6 I65	0	6.62	1	19.48	0.19
603639	2019	9 C26	1	9.39	1	15.25	0.15
603661	2019	2 C21	1	7.46	5	17.83	0.16
603662	2019	3 C21	0	7.46	5	17.83	0.16
603680	2017	6 C37	0	11.16	5	19.94	0.2

603680	2018	8	C37	0	6.64	6	20.95	0.21
603680	2019	0	C37	0	4.61	6	21.03	0.21
603690	2017	0	C35	1	6.05	1	18.46	0.18
603690	2018	7	C35	1	2.57	2	18.47	0.18
603691	2017	3	C35	0	6.05	1	18.46	0.18
603691	2018	1	C35	0	2.57	2	18.4	0.18
603699	2013	3	C34	0	16.77	9	12.88	0.13
603699	2014	10	C34	0	16.33	9	12.78	0.13
603699	2015	19	C34	0	8.56	9	12.91	0.13
603699	2016	33	C34	0	5.29	9	12.46	0.17
603699	2017	45	C34	0	4.9	10	12.18	0.13
603699	2019	0	C34	1	9.23	9	14.42	0.13
603728	2017	15	C38	1	9.82	4	13.98	0.14
603730	2017	56	C36	1	18.26	4	14.93	0.15
603730	2018	79	C36	1	12.46	7	15.11	0.15
603730	2019	82	C36	1	12.04	7	14.87	0.15
603731	2019	21	C36	1	12.04	7	14.87	0.15
603738	2019	12	C39	1	0.88	2	15.18	0.15
603739	2019	27	C14	1	8.03	2	12.45	0.1
603766	2016	57	C37	0	10.4	1	16.87	0.17
603766	2017	2	C37	0	9.98	2	17.14	0.17
603766	2018	1	C37	0	8.55	2	17.14	0.17
603766	2019	3	C37	0	4.8	3	17.32	0.17
603767	2019	22	C37	1	4.8	3	17.32	0.17
603776	2018	88	M74	1	4.7	3	14.76	0.1
603776	2019	147	M74	1	15.34	4	14.6	0.15
603777	2018	10	M74	0	4.7	3	14.76	0.1
603777	2019	12	M74	0	15.34	4	15.6	0.15
603799	2017	2	C32	1	13.85	8	17.29	0.18
603799	2018	0	C32	1	8.55	9	17.92	0.14
603799	2019	0	C32	0	0.51	8	18.93	0.21
603806	2016	0	C29	1	16.41	2	15.5	0.1
603806	2017	6	C29	1	10.45	2	15.65	0.1
603806	2018	9	C29	1	12.34	2	15.76	0.1
603816	2019	23	C21	1	10.72	6	17.5	0.25
603825	2017	13	I64	1	2.86	4	16.72	0.2
603825	2018	12	I64	1	2.12	5	16.74	0.18
603825	2019	6	I64	1	3.17	8	16.2	0.15
603871	2019	4	G58	1	15.25	3	15.42	0.1
603876	2018	2	C32	0	3.42	3	17.96	0.17
603899	2019	2	C24	1	16.25	2	16.69	0.1
603926	2018	1	C36	0	5.42	3	15.75	0.16
603926	2019	13	C36	0	6.53	3	16.44	0.16
603986	2016	11	C39	1	13.6	3	16.99	0.17
603986	2017	8	C39	1	18.73	3	15.17	0.15
603986	2018	0	C39	1	14.87	3	16.17	0.16
603993	2019	1	B09	0	1.62	14	18.12	0.24
603997	2016	0	C36	1	14.37	2	14.19	0.14
603997	2017	6	C36	1	14.48	4	15.6	0.16
603997	2019	50	C36	1	3.6	19	15.38	0.15
688003	2019	47	C36	0	3.6	19	15.38	0.15

688005	2019	75	C39	1	1.71	1	17.22	0.17
688036	2019	74	C39	0	12.79	4	18.75	0.19
688166	2019	25	C27	0	9.67	2	15.66	0.16
688233	2019	14	C30	1	20.64	2	14.88	0.1
688366	2019	20	C27	0	7.11	5	15.49	0.15
3.00E+06	2018	3	C39	0	5.46	6	19.54	0.2
3.00E+06	2019	8	C39	0	-5.39	6	20.27	0.19

breadth	depth	owner	age	back	debt	ep	ip	cd
27	4.5	0	28	1	3.43	2.07	7.96	2.33
27	4.5	0	29	1	3.68	1.17	7.88	2.33
28	4.67	0	30	1	3.52	1.19	8.83	2.33
20	2	1	25	1	1.21	0.99	8	2.86
21	1.62	1	27	1	1.49	1.75	8	2.86
20	2	1	25	1	1.21	0.64	8.08	0.84
21	1.62	1	27	1	1.49	0.44	8.5	0.84
20	2	1	25	1	1.21	2.1	7	3.4
21	1.62	1	27	1	1.49	0.21	9	4.17
4	1	0	20	0	1.09	3.29	11.46	2.8
15	2.14	1	19	0	2.41	1.16	7.58	2.86
24	3.43	1	20	0	1.37	0.99	8	2.86
33	5.5	1	21	1	1.47	1	8	2.86
42	4.67	1	22	1	1.46	1.75	8	2.86
14	1.4	1	14	1	2.32	1.33	7.5	1.9
15	1.36	1	15	1	2.25	1.35	8.08	1.9
15	1.36	1	16	1	1.56	1.29	8.67	1.9
16	1.45	1	17	1	1.19	1.04	8.38	1.9
24	1.85	1	18	1	0.97	0.85	8.04	1.9
23	1.77	1	19	1	1.02	1.22	8.67	1.9
12	1.5	1	12	1	2.94	6.22	6.67	4.43
13	1.44	1	13	1	2.41	6.31	6.75	4.43
14	1.4	1	14	1	2.32	6.33	6.08	4.43
15	1.36	1	15	1	2.25	5.22	7.71	4.43
15	1.36	1	16	1	1.56	6.21	6.42	4.43
16	1.45	1	17	1	1.19	4.66	7.33	4.43
24	1.85	1	18	1	0.97	3.84	7.75	4.43
23	1.77	1	19	1	1.02	2.64	8.5	4.43
12	1.5	1	12	1	2.94	2.34	4	2.86
13	1.44	1	13	1	2.41	1.91	6	2.86
14	1.4	1	14	1	2.32	1.74	5.71	2.86
15	1.36	1	15	1	2.25	1.37	6.58	2.86
15	1.36	1	16	1	1.56	1.16	7.58	2.86
16	1.45	1	17	1	1.19	0.99	8	2.86
24	1.85	1	18	1	0.97	1	8	2.86
23	1.77	1	19	1	1.02	1.75	8	2.86
12	1.5	1	12	1	2.94	0.84	8	5.07
13	1.44	1	13	1	2.41	0.6	8	5.07
14	1.4	1	14	1	2.32	0.3	8	5.07
15	1.36	1	15	1	2.25	0.21	8	5.07
15	1.36	1	16	1	1.56	0.6	8	5.07
16	1.45	1	17	1	1.19	1.22	8	5.07
24	1.85	1	18	1	0.97	1.25	8	5.07
23	1.77	1	19	1	1.02	1.13	8.08	5.07
3	1	1	26	1	0.52	2.16	10	2.26
4	1	1	27	1	1.48	2.18	10	2.26
8	1.33	1	29	1	1.81	1.98	10	2.26
15	1.88	1	30	1	1.89	1.98	10	2.26
13	1.63	1	32	1	1.22	0.43	10	2.26
17	1.7	1	33	1	1.82	0.31	10	2.26

8	1.33	1	29	1	1.51	3.41	8.5	2.33
13	1.63	1	30	1	1.89	3.49	7.88	2.33
9	1.5	1	31	1	1.75	2.97	7.5	2.33
12	1.5	1	32	1	1.22	2.32	7.71	2.33
14	1.4	1	33	1	1.82	2.07	7.96	2.33
20	2	1	34	1	1.46	1.17	7.88	2.33
13	1.3	1	35	1	1.64	1.19	8.83	2.33
15	1.5	1	33	1	1.82	0.74	11.04	2.8
18	1.8	1	34	1	1.46	0.83	11	2.8
14	1.4	1	35	1	1.64	0.71	11	2.8
6	1.5	0	34	1	1.68	0.64	8.08	0.84
10	2	0	35	1	1.7	0.61	8.5	0.84
133	5.12	0	36	1	3.29	0.44	8.5	0.84
49	3.27	0	31	1	2.15	2.91	7.5	2.33
51	3.19	0	32	1	2.29	3.15	8	2.33
54	3.6	0	33	1	2.09	3.51	8.5	2.33
64	3.76	0	34	1	2.33	3.41	8.5	2.33
30	3.33	0	35	1	2.71	3.49	7.88	2.33
35	3.5	0	36	1	2.5	2.97	7.5	2.33
80	4.71	0	37	1	2.92	2.32	7.71	2.33
85	5.31	0	38	1	2.69	2.07	7.96	2.33
47	3.62	0	39	1	2.85	1.17	7.88	2.33
47	3.62	0	40	1	2.98	1.19	8.83	2.33
35	3.5	0	36	1	2.5	2.35	9.5	4.65
80	4.71	0	37	1	2.92	2.36	9.08	4.65
85	5.31	0	38	1	2.69	2.36	9.42	4.65
3	1.5	1	29	0	0.23	2.33	10	3.46
3	1.5	1	30	0	0.32	2.66	10	3.46
3	1.5	1	31	0	0.44	2.59	8.88	3.46
3	1.5	1	32	0	0.24	2.03	8.79	3.46
3	1.5	1	33	0	0.29	1.17	9	3.46
3	1.5	1	34	0	0.37	0.29	9	3.46
3	1.5	1	35	0	0.29	0.37	8.5	3.46
3	1.5	1	36	1	0.28	0.42	8	3.46
3	1.5	1	37	1	0.49	0.47	7.63	3.46
3	1.5	1	38	1	0.25	0.44	7.5	3.46
52	17.33	0	27	1	10.03	0.59	7.42	1.45
69	11.5	0	28	1	7.8	0.56	7.71	1.45
104	20.8	0	29	1	7.99	0.44	9	1.45
114	16.29	0	30	1	9.13	0.23	8.29	1.45
120	15	0	31	1	6.74	0.1	8.88	1.45
120	15	0	31	1	6.74	1.04	10.46	4.44
3	1.5	1	29	1	5.09	0.49	7	0.84
4	1.33	1	30	1	5.07	0.49	7.17	0.84
4	1.33	1	31	1	2.37	0.57	8	0.84
4	1	1	28	1	4.51	2.16	10	2.26
4	1	1	29	1	3.89	2.18	10	2.26
4	1	1	30	1	3.46	2.22	10	2.26
4	1	1	31	1	3.16	1.98	10	2.26
6	1.2	1	32	1	0.88	1.98	10	2.26
5	1	1	33	1	0.63	1.59	10	2.26

5	1	1	34	1	0.58	0.43	10	2.26
5	1	1	35	1	1.05	0.31	10	2.26
5	1	1	36	1	1.31	0.22	10	2.26
6	1	1	37	1	1.45	0.43	10	2.26
6	1.2	1	32	1	0.88	3.23	11.5	2.8
5	1	1	33	1	0.63	1.51	11.5	2.8
5	1	1	34	1	0.58	0.79	11.5	2.8
5	1	1	35	1	1.05	0.74	11.04	2.8
5	1	1	36	1	1.31	0.83	11	2.8
6	1	1	37	1	1.45	0.71	11	2.8
6	1	1	37	1	1.45	1.22	8.67	1.9
4	1	0	26	0	1.18	0.44	8.5	0.84
8	1.6	1	36	1	0.82	1.75	8	2.86
5	1.25	0	14	1	2.56	3.79	7.58	1.79
4	1	0	15	1	3.26	6.74	7.5	1.79
4	1	0	16	1	3.94	2.91	7.5	1.79
10	1.11	0	17	1	3.39	2.43	7.5	1.79
11	1.1	0	18	1	3.21	2.33	7.63	1.79
6	1.2	0	19	1	2.61	2.33	6.54	1.79
7	1.17	0	20	1	3.82	2.55	6.5	1.79
10	1.25	0	21	1	3.12	1.99	7.33	1.79
10	1.25	0	22	1	4.21	1.92	8	1.79
21	1.17	0	23	1	3.58	1.59	8.46	1.79
5	1.25	0	14	1	2.56	2.03	8.5	1.9
10	1.11	0	17	1	3.39	1.96	7.5	1.9
11	1.1	0	18	1	3.21	1.33	7.5	1.9
21	1.17	0	23	1	3.58	1.22	8.67	1.9
1	1	1	25	1	2.87	3	4.88	4.65
1	1	1	26	1	3.1	3.04	4.5	4.65
1	1	1	27	1	3.69	5.4	4.5	4.65
5	1.25	1	28	1	3.77	4.39	4.5	4.65
10	1.11	1	32	1	2.01	7	7.79	4.65
11	1.1	1	33	1	1.64	5.8	7.5	4.65
12	1.2	1	34	1	1.86	6.11	7	4.65
5	1.25	1	28	1	3.77	0.33	7	1.42
4	1.33	1	29	1	3.89	0.37	7.17	1.42
5	1.25	1	30	1	2.29	0.43	8	1.42
8	1.14	1	31	1	2.66	0.44	8	1.42
10	1.11	1	32	1	2.01	0.49	8	1.42
11	1.1	1	33	1	1.64	0.61	8.08	1.42
12	1.2	1	34	1	1.86	1.06	8.5	1.42
5	1.25	1	28	1	3.77	1.91	6	2.86
4	1.33	1	29	1	3.89	1.74	5.71	2.86
5	1.25	1	30	1	2.29	1.37	6.58	2.86
8	1.14	1	31	1	2.66	1.16	7.58	2.86
10	1.11	1	32	1	2.01	0.99	8	2.86
11	1.1	1	33	1	1.64	1	8	2.86
12	1.2	1	34	1	1.86	1.75	8	2.86
8	1.14	1	31	1	2.66	0.99	8	5.83
10	1.11	1	32	1	2.01	0.76	8	5.83
11	1.1	1	33	1	1.64	0.59	8	5.83

12	1.2	1	34	1	1.86	1.81	7.13	5.83
10	1.11	1	32	1	2.01	0.52	7.13	2.52
11	1.1	1	33	1	1.64	0.73	7.04	2.52
12	1.2	1	34	1	1.86	0.78	7.17	2.52
11	1.1	1	33	1	1.64	1.73	8	4.13
12	1.2	1	34	1	1.86	1.29	9.38	4.13
91	3.03	0	29	1	3.44	6.07	9.5	2.65
98	3.38	0	30	1	4.84	5.98	9.5	2.65
102	3.52	0	31	1	5.07	5.6	8.92	2.65
120	4.29	0	32	1	3.63	4.94	8.5	2.65
134	4.32	0	33	1	3.06	4.69	7.67	2.65
34	2.62	0	34	1	4.45	2.56	7.5	2.65
29	2.23	0	35	1	3.57	2.39	8	2.65
42	3	0	36	1	4.33	2	8	2.65
46	3.29	0	37	1	3.35	1.61	8	2.65
10	2	0	13	1	1.01	5.98	9.5	2.65
9	1.8	0	14	1	1.17	5.6	8.92	2.65
15	1.88	0	15	1	1.14	4.94	8.5	2.65
13	1.44	0	16	1	1.29	4.69	7.67	2.65
18	2	0	17	1	1.33	2.56	7.5	2.65
16	1.78	0	18	1	1.39	2.39	8	2.65
18	2	0	19	1	1.2	2	8	2.65
11	1.57	0	20	1	1.43	1.61	8	2.65
15	1.88	0	15	1	1.14	3.29	11.46	2.8
13	1.44	0	16	1	1.29	3.23	11.5	2.8
18	2	0	17	1	1.33	1.51	11.5	2.8
16	1.78	0	18	1	1.39	0.79	11.5	2.8
18	2	0	19	1	1.2	0.74	11.04	2.8
14	1.4	0	21	1	1.35	0.71	11	2.8
3	1	0	14	0	0.44	2.91	7.5	1.79
4	1	0	15	0	0.44	2.43	7.5	1.79
4	1	0	16	0	0.36	2.33	7.63	1.79
3	1	0	17	0	0.35	2.33	6.54	1.79
3	1	0	18	0	0.42	2.55	6.5	1.79
3	1	0	19	0	0.45	1.99	7.33	1.79
3	1	0	20	0	0.28	1.92	8	1.79
2	1	0	21	0	0.29	1.59	8.46	1.79
4	1	0	16	0	0.36	4.23	9	3.8
3	1	0	17	0	0.35	0.21	8	5.07
3	1	0	18	0	0.42	0.6	8	5.07
3	1	0	19	0	0.45	1.22	8	5.07
3	1	0	20	0	0.28	1.25	8	5.07
11	3.67	0	14	1	1.76	0.61	7.5	1.45
7	2.07	0	15	1	1.89	0.66	8.5	1.45
11	2.06	0	14	1	1.76	2.15	6	5.48
7	2.07	0	15	1	1.89	1.42	6.04	5.48
11	2.06	0	14	1	1.76	3.41	8.5	2.33
7	2.07	0	15	1	1.89	3.49	7.88	2.33
9	2.15	0	17	1	1.66	0.79	11.5	2.8
11	2.17	0	18	1	2.24	0.74	11.04	2.8
8	2.07	0	19	1	2.06	0.83	11	2.8

9	2.07	0	20	1	1.91	0.71	11	2.8
15	1.36	1	11	1	1.4	5.6	8.92	2.65
20	1.67	1	12	1	1.59	4.94	8.5	2.65
15	1.36	1	11	1	1.4	2.06	7.71	1.9
20	1.67	1	12	1	1.59	1.96	7.5	1.9
5	1	1	13	1	2.45	1.33	7.5	1.9
5	1	1	14	1	2.31	1.35	8.08	1.9
6	1	1	15	1	3.76	1.29	8.67	1.9
7	1.17	1	16	1	3.78	1.04	8.38	1.9
9	1.13	1	17	1	3.64	0.85	8.04	1.9
9	1.5	1	18	1	3.69	1.22	8.67	1.9
20	1.67	1	12	1	1.59	3.41	8.5	2.33
6	1.2	0	23	1	3.64	1.35	8.08	1.9
9	1.5	0	24	1	5.74	1.29	8.67	1.9
5	1.67	1	20	1	1.6	2.06	7.71	1.9
7	1.4	1	21	1	1.52	1.96	7.5	1.9
8	1.33	1	22	1	1.41	1.33	7.5	1.9
7	1.17	1	23	1	1.07	1.35	8.08	1.9
8	1.14	1	24	1	1.15	1.29	8.67	1.9
8	1.14	1	25	1	1.07	1.04	8.38	1.9
11	1.1	1	26	0	1.03	0.85	8.04	1.9
14	1.17	1	27	1	1.32	1.22	8.67	1.9
7	1.4	1	21	1	1.52	4.94	8.5	2.65
8	1.33	1	22	1	1.41	4.69	7.67	2.65
7	1.17	1	23	1	1.07	2.56	7.5	2.65
8	1.14	1	24	1	1.15	2.39	8	2.65
8	1.14	1	25	1	1.07	2	8	2.65
11	1.1	1	26	0	1.03	1.61	8	2.65
14	1.17	1	27	1	1.32	1.58	8.38	2.65
11	1.1	1	26	0	1.03	1.92	8	1.79
14	1.17	1	27	1	1.32	1.59	8.46	1.79
11	1.1	1	26	0	1.03	0.23	8.29	1.45
14	1.17	1	27	1	1.32	0.1	8.88	1.45
11	1.1	1	26	0	1.03	2.27	6.38	3.31
14	1.17	1	27	1	1.32	2.41	7.5	3.31
3	1	1	20	1	2.43	3.26	11	2.8
4	1	1	21	1	2.34	3.29	11.46	2.8
4	1	1	22	1	3.05	3.23	11.5	2.8
5	1	1	23	1	3.6	1.51	11.5	2.8
5	1	1	24	1	2.69	0.79	11.5	2.8
5	1	1	25	1	2.71	0.74	11.04	2.8
6	1.2	1	26	1	3.17	0.83	11	2.8
6	1.2	1	27	1	2.85	0.71	11	2.8
5	1	1	23	1	3.6	1.59	10	2.26
5	1	1	24	1	2.69	0.43	10	2.26
5	1	1	25	1	2.71	0.31	10	2.26
6	1.2	1	26	1	3.17	0.22	10	2.26
6	1.2	1	27	1	2.85	0.43	10	2.26
2	2	1	16	1	1.7	1.73	7.17	5.83
2	2	1	17	1	1.66	1.2	6.71	5.83
2	2	1	18	1	1.67	1.07	6.83	5.83

2	2	1	19	1	1.63	1.14	7	5.83
2	2	1	20	1	1.67	1.25	7.5	5.83
3	1.5	1	21	1	1.44	0.99	8	5.83
3	1.5	1	22	1	2.14	0.76	8	5.83
2	2	1	23	1	2.08	0.59	8	5.83
3	1.5	1	24	1	1.82	1.81	7.13	5.83
3	1.5	1	21	1	1.44	0.56	7.71	1.45
3	1.5	1	22	1	2.14	0.44	9	1.45
3	1.5	1	24	1	1.82	0.1	8.88	1.45
8	1.14	1	18	1	1.2	2.03	8.5	1.9
11	1.1	1	19	1	1.44	2.12	8.5	1.9
12	1.09	1	20	1	1.43	2.06	7.71	1.9
11	1.1	1	21	1	1.37	1.96	7.5	1.9
13	1.3	1	22	1	1.28	1.33	7.5	1.9
13	1.18	1	23	1	1.29	1.35	8.08	1.9
13	1.18	1	24	1	1.33	1.29	8.67	1.9
15	1.15	1	25	1	1.38	1.04	8.38	1.9
15	1.15	1	26	1	1.67	0.85	8.04	1.9
18	1.29	1	27	1	1.84	1.22	8.67	1.9
12	1.09	1	20	1	1.43	1.72	9.42	4.65
11	1.1	1	21	1	1.37	3.89	9	4.65
13	1.3	1	22	1	1.28	3.57	9.5	4.65
13	1.18	1	23	1	1.29	2.35	9.5	4.65
13	1.18	1	24	1	1.33	2.36	9.08	4.65
15	1.15	1	25	1	1.38	2.36	9.42	4.65
15	1.15	1	26	1	1.67	2.94	9.5	4.65
18	1.29	1	27	1	1.84	0.9	10.38	4.65
15	1.15	1	25	1	1.38	2	8	2.65
15	1.15	1	26	1	1.67	1.61	8	2.65
18	1.29	1	27	1	1.84	1.58	8.38	2.65
18	1.29	1	27	1	1.84	0.1	8.88	1.45
1	1	1	26	0	0.31	0.66	10	5.44
4	1.33	1	27	0	0.34	0.59	10.46	5.44
11	1.22	1	27	0	1.25	1.22	8.67	1.9
11	1.22	1	27	0	1.25	0.43	10	2.26
12	1.33	1	20	1	1.11	1.04	8.38	1.9
16	1.45	1	21	1	0.92	0.85	8.04	1.9
15	1.67	1	22	1	1.02	1.22	8.67	1.9
12	1.33	1	20	1	1.11	0.84	10	5.44
16	1.45	1	21	1	0.92	0.66	10	5.44
15	1.67	1	22	1	1.02	0.59	10.46	5.44
5	1.67	0	18	1	2.16	3.29	11.46	2.8
4	1.33	0	24	1	1.04	0.1	8.88	1.45
2	1	0	17	0	2.09	2.91	7.5	2.33
2	1	0	18	0	1.69	3.15	8	2.33
2	1	0	19	0	1.57	3.51	8.5	2.33
2	1	0	20	0	1.28	3.41	8.5	2.33
1	1	0	21	0	1.42	3.49	7.88	2.33
1	1	0	22	1	1.19	2.97	7.5	2.33
1	1	0	23	1	1.23	2.32	7.71	2.33
1	1	0	24	1	1.26	2.07	7.96	2.33

1	1	0	25	1	1.44	1.17	7.88	2.33
1	1	1	22	1	0.89	0.76	8	5.83
4	2	1	21	0	1.72	2.3	7	3.95
4	2	1	22	0	2.02	2.81	7	3.95
4	2	1	23	0	1.39	2.04	7	3.95
4	2	1	24	0	1.84	1.02	8.67	3.95
5	1.25	1	19	1	3.64	0.65	7.54	1.45
5	1.25	1	20	0	4.05	0.64	7.33	1.45
5	1.25	1	21	0	3.71	0.61	7.5	1.45
5	1.25	1	19	1	3.64	2.12	8.5	1.9
5	1.25	1	20	0	4.05	2.06	7.71	1.9
5	1.25	1	21	0	3.71	1.96	7.5	1.9
5	1.25	1	19	1	3.64	3.15	8	2.33
5	1.25	1	20	0	4.05	3.51	8.5	2.33
5	1.25	1	21	0	3.71	3.41	8.5	2.33
5	1.25	1	20	0	4.05	0.84	8	5.07
5	1.25	1	21	0	3.71	0.6	8	5.07
3	1.5	1	23	0	0.47	1.17	7.88	2.33
3	1.5	1	24	0	0.55	1.19	8.83	2.33
3	1.5	1	23	0	0.47	1	8	2.86
3	1.5	1	24	0	0.55	1.75	8	2.86
3	1	0	22	0	5.96	1.98	10	2.26
5	1.67	0	23	0	4.6	1.59	10	2.26
6	1.2	0	25	1	6.7	0.31	10	2.26
4	1	1	16	1	1.48	3.25	11.5	2.8
4	1	1	17	1	1.98	3.26	11	2.8
4	1	1	18	1	1.85	3.29	11.46	2.8
6	1	1	19	1	1.73	3.23	11.5	2.8
6	1	1	20	1	1.61	1.51	11.5	2.8
6	1	1	21	1	1.45	0.79	11.5	2.8
6	1	1	22	1	1.23	0.74	11.04	2.8
6	1	1	23	1	1.02	0.83	11	2.8
7	1	1	24	1	1.22	0.71	11	2.8
6	1	1	19	1	1.73	4.69	7.67	2.65
6	1	1	20	1	1.61	2.56	7.5	2.65
6	1	1	21	1	1.45	2.39	8	2.65
6	1	1	22	1	1.23	2	8	2.65
6	1	1	23	1	1.02	1.61	8	2.65
7	1	1	24	1	1.22	1.58	8.38	2.65
7	1	1	24	1	1.22	1.81	7.13	5.83
4	1.33	0	22	1	3.88	0.85	8	0.84
5	1.25	0	23	1	3.8	0.69	7.17	0.84
5	1.25	0	24	1	2.99	0.71	7	0.84
5	1.25	0	25	1	2.84	0.49	7	0.84
5	1.25	0	26	1	2.52	0.49	7.17	0.84
6	1.5	0	28	0	2.38	0.54	8	0.84
6	1.5	0	29	0	2.37	0.64	8.08	0.84
6	1.5	0	30	0	2.26	0.61	8.5	0.84
6	1.5	0	31	0	1.74	0.44	8.5	0.84
4	1.33	0	26	1	1.55	4.12	6.67	4.43
4	1.33	0	27	1	1.3	6.01	6.5	4.43

4	1.33	0	28	1	1.55	6.22	6.67	4.43
4	1.33	0	29	1	3.18	6.31	6.75	4.43
4	1.33	0	30	1	2.31	6.33	6.08	4.43
4	1.33	0	31	1	2.03	5.22	7.71	4.43
4	1.33	0	32	0	4.46	6.21	6.42	4.43
4	1.33	0	26	1	1.28	2.91	7.5	2.33
4	1.33	0	27	1	1.3	3.15	8	2.33
4	1.33	0	28	1	1.55	3.51	8.5	2.33
4	1.33	0	29	1	3.18	3.41	8.5	2.33
4	1.33	0	30	1	2.31	3.49	7.88	2.33
4	1.33	0	31	1	2.03	2.97	7.5	2.33
4	1.33	0	32	0	4.46	2.32	7.71	2.33
7	1.75	1	18	0	1.69	3.87	7.13	3.95
10	1.67	1	19	1	1.66	3.71	7.63	3.95
20	2.86	1	20	1	2.36	2.05	7.04	3.95
26	3.71	1	21	1	2.89	2.3	7	3.95
3	1	1	24	1	1.55	0.71	11	2.8
1	1	0	19	0	1.62	4.2	9.42	3.8
1	1	0	20	0	2.87	3.59	9.5	3.8
4	4	0	21	0	1.62	3.54	9.5	3.8
4	4	0	21	0	1.62	3.54	9.5	3.8
1	1	0	22	0	0.99	2.79	9.42	3.8
4	4	0	23	1	0.76	0.38	9.58	3.8
10	2	0	21	1	0.41	1.08	7.67	3.2
13	2.6	0	22	1	0.39	1.7	8	3.2
12	2.4	0	23	1	0.35	1.38	7.71	3.2
24	4.8	0	24	1	0.39	1.31	8.25	3.2
6	1.5	0	21	1	0.99	3.89	9	4.65
7	1	1	19	0	2.07	5.98	9.5	2.65
10	1.11	1	20	0	2	5.6	8.92	2.65
10	1.11	1	21	1	1.84	4.94	8.5	2.65
10	1.11	1	22	1	1.65	4.69	7.67	2.65
10	1.11	1	23	1	1.86	2.56	7.5	2.65
11	1.1	1	24	0	1.78	2.39	8	2.65
12	1.2	1	25	1	1.8	2	8	2.65
12	1.2	1	26	1	1.69	1.61	8	2.65
12	1.2	1	27	1	1.53	1.58	8.38	2.65
1	1	1	18	1	0.32	2.12	8.5	1.9
1	1	1	19	1	0.6	2.06	7.71	1.9
1	1	1	20	1	0.72	1.96	7.5	1.9
1	1	1	21	1	0.92	1.33	7.5	1.9
1	1	1	22	1	0.91	1.35	8.08	1.9
1	1	1	23	0	0.88	1.29	8.67	1.9
1	1	1	24	0	0.94	1.04	8.38	1.9
1	1	1	25	1	0.62	0.85	8.04	1.9
1	1	1	26	1	0.83	1.22	8.67	1.9
5	1.25	0	19	1	3.92	3.23	11.5	2.8
5	1.25	0	20	1	2.7	1.51	11.5	2.8
6	2	0	22	0	0.12	1.17	7.88	2.33
6	2	0	23	0	0.31	1.19	8.83	2.33
2	1	0	23	1	1.67	1.59	10	2.26

4	2	0	24	1	1.72	0.43	10	2.26
3	1	0	25	1	1.31	0.31	10	2.26
4	2	0	24	1	1.72	0.43	10	2.26
3	1	0	26	1	1.16	0.22	10	2.26
3	1	0	27	1	1.15	0.43	10	2.26
4	1	1	27	1	2.1	0.59	10.46	5.44
2	1	1	25	1	1.79	0.31	10	2.26
3	1	1	26	1	2.14	0.22	10	2.26
4	1	1	27	1	2.1	0.43	10	2.26
6	1.2	0	30	1	0.33	0.99	8	2.86
6	1.2	0	31	1	0.39	1	8	2.86
7	1.4	0	32	1	0.47	1.75	8	2.86
10	1.67	1	28	1	3.1	0.6	8	1.42
9	1.5	1	29	1	3.52	0.47	7.17	1.42
9	1.8	1	30	1	4.44	0.37	7	1.42
11	1.38	1	31	1	2.5	0.33	7	1.42
15	1.5	1	32	1	2.77	0.37	7.17	1.42
18	1.38	1	33	1	3.48	0.43	8	1.42
18	1.38	1	34	1	3.39	0.44	8	1.42
13	2.17	1	35	1	3.09	0.49	8	1.42
21	1.4	1	36	1	3.06	0.61	8.08	1.42
25	1.67	1	37	1	3.45	1.06	8.5	1.42
10	1.67	1	28	1	3.1	4.12	6.67	4.43
9	1.5	1	29	1	3.52	6.01	6.5	4.43
9	1.8	1	30	1	4.44	6.22	6.67	4.43
11	1.38	1	31	1	2.5	6.31	6.75	4.43
15	1.5	1	32	1	2.77	6.33	6.08	4.43
18	1.38	1	33	1	3.48	5.22	7.71	4.43
18	1.38	1	34	1	3.39	6.21	6.42	4.43
13	2.17	1	35	1	3.09	4.66	7.33	4.43
21	1.4	1	36	1	3.06	3.84	7.75	4.43
25	1.67	1	37	1	3.45	2.64	8.5	4.43
11	1.38	1	31	1	2.5	1.96	7.5	1.9
15	1.5	1	32	1	2.77	1.33	7.5	1.9
18	1.38	1	33	1	3.48	1.35	8.08	1.9
18	1.38	1	34	1	3.39	1.29	8.67	1.9
13	2.17	1	35	1	3.09	1.04	8.38	1.9
21	1.4	1	36	1	3.06	0.85	8.04	1.9
25	1.67	1	37	1	3.45	1.22	8.67	1.9
11	1.38	1	31	1	2.5	4.62	9	3.8
15	1.5	1	32	1	2.77	4.23	9	3.8
18	1.38	1	33	1	3.48	4.2	9.42	3.8
18	1.38	1	34	1	3.39	3.59	9.5	3.8
13	2.17	1	35	1	3.09	3.54	9.5	3.8
21	1.4	1	36	1	3.06	2.79	9.42	3.8
25	1.67	1	37	1	3.45	0.38	9.58	3.8
15	1.5	1	32	1	2.77	2.33	7.63	1.79
18	1.38	1	33	1	3.48	2.33	6.54	1.79
18	1.38	1	34	1	3.39	2.55	6.5	1.79
13	2.17	1	35	1	3.09	1.99	7.33	1.79
21	1.4	1	36	1	3.06	1.92	8	1.79

25	1.67	1	37	1	3.45	1.59	8.46	1.79
15	1.5	1	32	1	2.77	4.69	7.67	2.65
18	1.38	1	33	1	3.48	2.56	7.5	2.65
18	1.38	1	34	1	3.39	2.39	8	2.65
13	2.17	1	35	1	3.09	2	8	2.65
21	1.4	1	36	1	3.06	1.61	8	2.65
25	1.67	1	37	1	3.45	1.58	8.38	2.65
21	1.4	1	36	1	3.06	0.23	8.29	1.45
25	1.67	1	37	1	3.45	0.1	8.88	1.45
5	1.67	1	19	1	1.92	0.59	7.42	1.45
6	1.5	1	21	1	1.45	0.44	9	1.45
7	1.75	1	22	0	1.47	0.23	8.29	1.45
2	1	1	23	1	0.5	1.13	8.08	5.07
8	1.6	0	24	1	1.64	0.71	11	2.8
11	1.22	0	17	1	1.72	0.22	10	2.26
10	1.25	0	18	1	1.76	0.43	10	2.26
5	1	0	15	1	1.45	1.29	8.67	1.9
8	1.14	0	16	1	1.79	1.04	8.38	1.9
11	1.22	0	17	1	1.72	0.85	8.04	1.9
10	1.25	0	18	1	1.76	1.22	8.67	1.9
1	1	1	23	0	1.11	0.69	7.17	0.84
1	1	1	24	0	0.78	0.71	7	0.84
1	1	1	25	0	0.68	0.49	7	0.84
1	1	1	26	0	0.75	0.49	7.17	0.84
14	1.4	1	27	0	1.18	0.57	8	0.84
15	1.25	1	28	0	1.27	0.54	8	0.84
16	1.33	1	29	0	0.99	0.64	8.08	0.84
16	1.33	1	30	0	1.09	0.61	8.5	0.84
16	1.33	1	31	0	0.91	0.44	8.5	0.84
14	1.4	1	27	0	1.18	1.35	8.08	1.9
15	1.25	1	28	0	1.27	1.29	8.67	1.9
16	1.33	1	29	0	0.99	1.04	8.38	1.9
16	1.33	1	30	0	1.09	0.85	8.04	1.9
16	1.33	1	31	0	0.91	1.22	8.67	1.9
2	1	0	21	0	1.06	0.69	7.17	0.84
2	1	0	22	0	1.98	0.71	7	0.84
2	1	0	23	0	1.96	0.49	7	0.84
2	1	0	24	0	1.03	0.49	7.17	0.84
2	1	0	25	0	0.85	0.57	8	0.84
2	1	0	26	0	0.82	0.54	8	0.84
2	1	0	27	0	0.89	0.64	8.08	0.84
2	1	0	28	0	0.7	0.61	8.5	0.84
2	1	0	20	1	0.37	2.06	7.71	1.9
2	1	0	21	1	0.68	1.96	7.5	1.9
2	1	0	22	1	0.71	1.33	7.5	1.9
2	1	0	23	1	0.6	1.35	8.08	1.9
2	1	0	24	1	0.62	1.29	8.67	1.9
3	1	0	25	1	0.59	1.04	8.38	1.9
3	1	0	26	1	0.8	0.85	8.04	1.9
3	1	0	27	1	1.16	1.22	8.67	1.9
3	1	0	25	1	0.59	2	8	2.65

3	1	0	26	1	0.8	1.61	8	2.65
4	1	1	20	0	1.75	2	8	2.65
4	1	1	21	0	1.35	1.61	8	2.65
4	1	1	22	0	1.17	1.58	8.38	2.65
3	1	0	26	1	1.64	0.83	11	2.8
4	1.33	0	27	1	1.84	0.71	11	2.8
14	2	0	14	0	0.97	0.65	7.54	1.45
16	2	0	15	0	0.91	0.64	7.33	1.45
11	2.2	0	16	0	0.98	0.61	7.5	1.45
14	2	0	14	0	0.97	3.83	9	4.17
16	2	0	15	0	0.91	3.34	9	4.17
33	3.67	0	19	1	0.81	17.01	10.5	2.35
47	2.76	0	20	1	1	15.04	10.5	2.35
52	2.74	0	21	1	0.92	0.57	10.5	2.35
53	2.65	0	22	1	1.03	0.75	10.25	2.35
19	3.17	0	18	1	1	1.86	9	3.8
33	3.67	0	19	1	0.81	1.37	9	3.8
47	2.76	0	20	1	1	1.38	9	3.8
52	2.74	0	21	1	0.92	1.44	9	3.8
53	2.65	0	22	1	1.03	1.01	10.04	3.8
8	1.6	0	16	0	1.96	1.98	10	2.26
8	1.6	0	17	1	1.77	1.59	10	2.26
7	1.4	0	18	1	1	0.43	10	2.26
7	1.4	0	19	1	0.76	0.31	10	2.26
8	1.33	0	20	1	0.56	0.22	10	2.26
7	1.4	0	21	1	0.54	0.43	10	2.26
2	1	0	14	1	0.71	0.64	7.33	1.45
1	1	1	14	0	0.31	0.47	7.17	1.42
1	1	1	15	0	0.35	0.37	7	1.42
1	1	1	16	0	0.33	0.33	7	1.42
1	1	1	17	1	0.38	0.37	7.17	1.42
1	1	1	18	1	0.38	0.43	8	1.42
1	1	1	19	1	0.32	0.44	8	1.42
1	1	1	20	1	0.31	0.49	8	1.42
1	1	1	21	0	0.23	0.61	8.08	1.42
1	1	1	22	0	0.25	1.06	8.5	1.42
5	1.25	1	15	1	8.04	3.25	11.5	2.8
4	1.33	1	16	1	4.85	3.26	11	2.8
5	1.25	1	17	1	3.19	3.29	11.46	2.8
5	1.25	1	18	1	2.7	3.23	11.5	2.8
4	1.33	1	19	1	2.43	1.51	11.5	2.8
4	1.33	1	20	1	2.82	0.79	11.5	2.8
6	1	1	19	1	1.93	1.04	8.38	1.9
7	1.17	1	20	1	2.42	0.85	8.04	1.9
8	1.14	1	21	1	2.54	1.22	8.67	1.9
5	1	1	18	1	2.24	2.39	8	2.65
6	1	1	19	1	1.93	2	8	2.65
7	1.17	1	20	1	2.42	1.61	8	2.65
8	1.14	1	21	1	2.54	1.58	8.38	2.65
5	1	1	18	1	2.24	2.87	6.5	3.31
6	1	1	19	1	1.93	1.92	6.33	3.31

7	1.17	1	20	1	2.42	2.27	6.38	3.31
8	1.14	1	21	1	2.54	2.41	7.5	3.31
6	1.5	1	15	0	1.33	3.26	11	2.8
6	1.5	1	16	0	1.2	3.29	11.46	2.8
31	5.17	1	21	0	1.15	0.83	11	2.8
30	6	1	22	0	0.71	0.71	11	2.8
6	1.5	1	15	0	1.33	3.51	8.5	2.33
6	1.5	1	16	0	1.2	3.41	8.5	2.33
31	5.17	1	21	0	1.15	1.17	7.88	2.33
30	6	1	22	0	0.71	1.19	8.83	2.33
13	1.63	1	21	0	0.79	1.81	7.13	5.83
13	1.63	1	21	0	0.79	1.58	8.38	2.65
13	1.63	1	21	0	0.79	1.59	8.46	1.79
6	2	1	18	1	0.42	0.79	11.5	2.8
8	2.67	1	19	0	0.54	0.74	11.04	2.8
8	2.67	1	20	0	0.68	0.83	11	2.8
13	1.63	1	21	0	0.79	0.71	11	2.8
13	1.63	1	21	0	0.79	1.19	8.83	2.33
6	2	1	18	1	0.42	1.29	8.67	1.9
8	2.67	1	19	1	0.54	1.04	8.38	1.9
8	2.67	1	20	0	0.68	0.85	8.04	1.9
13	1.63	1	21	0	0.79	1.22	8.67	1.9
26	2.36	0	26	0	0.88	0.22	10	2.26
28	2.33	0	27	1	0.7	0.43	10	2.26
28	2.33	0	27	1	0.7	1.22	8.67	1.9
6	2	1	21	0	1.81	1.25	10.29	4.44
8	1.6	1	22	0	1.92	1.04	10.46	4.44
3	1.5	1	13	1	1.48	0.65	7.54	1.45
3	1.5	1	14	1	1.6	0.64	7.33	1.45
3	1.5	1	15	1	1.33	0.61	7.5	1.45
8	1.33	1	19	1	1.1	0.44	9	1.45
8	1.33	1	19	1	1.1	0.76	8	5.83
10	2	1	20	1	1.15	0.59	8	5.83
8	1.33	1	19	1	1.1	1.04	8.38	1.9
10	2	1	20	1	1.15	0.85	8.04	1.9
4	2	1	21	1	1.41	0.44	8.5	0.84
1	1	0	22	0	0.61	0.85	8	0.84
1	1	0	23	0	0.95	0.69	7.17	0.84
1	1	0	24	0	0.55	0.71	7	0.84
1	1	0	25	0	0.54	0.49	7	0.84
1	1	0	26	0	0.64	0.49	7.17	0.84
1	1	0	27	0	0.66	0.57	8	0.84
2	1	0	28	1	0.64	0.54	8	0.84
2	1	0	29	1	0.73	0.64	8.08	0.84
5	1.67	0	30	1	0.9	0.61	8.5	0.84
5	1.67	0	31	1	0.86	0.44	8.5	0.84
18	2	1	29	1	1.55	0.79	7	3.4
13	1.86	1	30	1	1.72	1.07	7.04	3.4
93	18.6	1	29	1	4.14	2.85	7.5	3.1
82	16.4	1	30	1	4.12	2.86	7.88	3.1
2	1	0	23	0	1.47	0.2	9	4.17

2	1	0	24	0	1.31	0.23	9	4.17
2	1	0	25	0	1.3	0.21	9	4.17
3	1.5	0	29	0	0.19	0.4	6.83	2.52
3	1.5	0	30	0	0.23	0.52	7.13	2.52
3	1.5	0	31	0	0.3	0.73	7.04	2.52
5	1.25	0	32	1	0.39	0.78	7.17	2.52
13	1.86	0	15	1	0.77	1.73	10	5.44
13	1.86	0	16	1	0.7	1.71	10	5.44
14	2	0	17	1	0.56	1.34	10	5.44
23	2.88	0	18	1	0.91	1.04	10	5.44
21	2.33	0	19	1	0.98	0.84	10	5.44
26	2.6	0	20	1	1.24	0.66	10	5.44
11	1.83	0	14	1	0.84	3.26	11	2.8
13	1.86	0	15	1	0.77	3.29	11.46	2.8
13	1.86	0	16	1	0.7	3.23	11.5	2.8
14	2	0	17	1	0.56	1.51	11.5	2.8
23	2.88	0	18	1	0.91	0.79	11.5	2.8
21	2.33	0	19	1	0.98	0.31	10	2.26
26	2.6	0	20	1	1.24	0.22	10	2.26
3	1	0	23	1	1.87	0.71	11	2.8
6	2	0	15	0	1.79	1.51	11.5	2.8
6	1.5	0	16	0	1.63	0.79	11.5	2.8
7	1.75	0	17	0	2.14	0.74	11.04	2.8
8	1.6	0	18	0	3.69	0.83	11	2.8
8	1.6	0	19	0	2.72	0.71	11	2.8
1	1	1	19	0	1.62	1.17	7.88	2.33
1	1	1	20	0	1.62	1.19	8.83	2.33
13	2.17	0	28	1	3.39	2.41	7.5	3.31
5	1.25	1	14	1	0.84	5.98	9.5	2.65
4	1	1	16	1	0.99	4.94	8.5	2.65
4	1	1	17	1	1.08	4.69	7.67	2.65
4	1	1	18	1	0.88	2.56	7.5	2.65
4	1	1	19	1	0.74	2.39	8	2.65
4	1	1	20	1	0.64	2	8	2.65
4	1	1	21	1	0.69	1.61	8	2.65
4	1	1	22	1	0.5	1.58	8.38	2.65
5	1.25	1	14	1	0.84	0.74	6.71	2.52
4	1	1	16	1	0.99	0.56	6	2.52
4	1	1	17	1	1.08	0.56	6.04	2.52
4	1	1	18	1	0.88	0.39	6.58	2.52
4	1	1	19	1	0.74	0.4	6.83	2.52
4	1	1	20	1	0.64	0.52	7.13	2.52
4	1	1	21	1	0.69	0.73	7.04	2.52
4	1	1	22	1	0.5	0.78	7.17	2.52
14	2	0	19	1	0.44	0.22	10	2.26
17	1.89	0	20	1	0.43	0.43	10	2.26
4	1	0	19	0	0.24	0.84	10	5.44
5	1.25	0	20	0	0.4	0.66	10	5.44
5	1.25	1	14	1	0.84	2.12	8.5	1.9
4	1	1	16	1	0.99	1.96	7.5	1.9
4	1	1	17	1	1.08	1.33	7.5	1.9

4	1	1	18	1	0.88	1.35	8.08	1.9
4	1	1	19	1	0.74	1.29	8.67	1.9
4	1	1	20	1	0.64	1.04	8.38	1.9
4	1	1	21	1	0.69	0.85	8.04	1.9
4	1	1	22	1	0.5	1.22	8.67	1.9
17	1.89	0	20	1	0.43	0.9	10.38	4.65
5	1.67	0	21	0	0.52	0.43	10	2.26
2	2	0	16	1	1.65	3.25	11.5	2.8
2	1	0	17	1	1.65	3.26	11	2.8
6	1.5	0	18	1	1.89	3.29	11.46	2.8
5	1.67	0	19	0	1.8	3.23	11.5	2.8
5	1.67	0	20	0	1.84	1.51	11.5	2.8
5	1.67	0	21	1	1.02	0.79	11.5	2.8
4	1.33	0	22	1	0.93	0.74	11.04	2.8
6	1.5	0	23	1	1.37	0.83	11	2.8
10	2.5	0	24	1	1.7	0.71	11	2.8
1	1	0	22	0	0.24	0.44	8.5	0.84
21	7	0	21	1	0.48	0.31	10	2.26
27	6.75	0	22	1	0.33	0.22	10	2.26
28	5.6	0	23	1	0.34	0.43	10	2.26
2	1	0	20	0	0.46	2.06	7.71	1.9
2	1	0	21	0	0.48	1.96	7.5	1.9
4	1	0	22	0	0.47	1.33	7.5	1.9
4	1	0	23	0	0.53	1.35	8.08	1.9
4	1	0	24	0	0.5	1.29	8.67	1.9
7	1	0	25	0	0.52	1.04	8.38	1.9
11	1	0	26	0	0.63	0.85	8.04	1.9
16	1	0	27	0	0.72	1.22	8.67	1.9
3	1	0	11	0	0.99	2.12	8.5	1.9
5	1	0	12	1	0.96	2.06	7.71	1.9
6	1.2	0	13	1	0.59	1.96	7.5	1.9
7	1.17	0	14	1	0.38	1.33	7.5	1.9
8	1.33	0	15	0	0.43	1.35	8.08	1.9
8	1.33	0	16	0	0.57	1.29	8.67	1.9
10	1.43	0	17	0	1.21	1.04	8.38	1.9
11	1.57	0	18	0	1.18	0.85	8.04	1.9
12	1.71	0	19	1	0.7	1.22	8.67	1.9
1	1	0	15	1	0.52	0.71	7	0.84
1	1	0	16	1	0.6	0.49	7	0.84
1	1	0	17	1	0.62	0.49	7.17	0.84
1	1	0	18	0	0.54	0.57	8	0.84
1	1	0	19	0	0.73	0.54	8	0.84
3	1.5	0	20	0	0.76	0.64	8.08	0.84
3	1.5	0	21	0	0.8	0.61	8.5	0.84
4	1.33	0	22	1	0.73	0.44	8.5	0.84
2	1	0	19	1	1.3	0.43	10	2.26
2	1	0	20	1	1.51	0.31	10	2.26
3	1	0	21	1	2.23	0.22	10	2.26
4	1	0	22	1	2.79	0.43	10	2.26
4	1.33	0	16	1	1.55	0.57	8	0.84
4	1.33	0	17	1	1.7	0.54	8	0.84

5	1.67	0	18	1	1.28	0.64	8.08	0.84
5	1.67	0	19	1	1.61	0.61	8.5	0.84
5	1.67	0	20	1	1.85	0.44	8.5	0.84
4	2	0	19	0	1	0.21	9	4.17
7	1.17	0	25	0	1.2	0.44	8.5	0.84
7	1.17	0	25	0	1.2	1.18	10.5	5.28
3	1	1	22	1	1.33	0.66	8.5	1.45
9	2.25	1	23	1	1.77	0.59	7.42	1.45
4	1.33	1	24	1	2.14	0.56	7.71	1.45
20	2	1	25	1	1.21	0.44	9	1.45
21	1.62	1	27	1	1.49	0.1	8.88	1.45
2	1	0	32	0	0.72	0.71	11	2.8
6	1	0	17	0	0.7	2.16	10	2.26
4	1	0	18	0	0.67	2.18	10	2.26
7	1	0	19	0	0.69	2.22	10	2.26
5	1	0	24	0	0.56	0.31	10	2.26
6	1	0	17	0	0.7	1.93	10	5.44
4	1	0	18	0	0.67	1.94	10	5.44
7	1	0	19	0	0.69	1.97	10	5.44
4	1	0	20	0	1.09	1.73	10	5.44
12	1.5	1	12	1	2.94	3.99	6.75	3.1
13	1.44	1	13	1	2.41	4.07	6.5	3.1
15	1.36	1	16	1	1.56	3.31	6.79	3.1
16	1.45	1	17	1	1.19	2.62	6.83	3.1
24	1.85	1	18	1	0.97	2.85	7.5	3.1
23	1.77	1	19	1	1.02	2.86	7.88	3.1
14	1.4	1	14	1	2.32	2.96	6.5	3.1
15	1.36	1	15	1	2.25	3.99	6.5	3.1
15	1.36	1	16	1	1.56	3.31	6.79	3.1
16	1.45	1	17	1	1.19	2.62	6.83	3.1
24	1.85	1	18	1	0.97	2.85	7.5	3.1
23	1.77	1	19	1	1.02	2.86	7.88	3.1
12	1.5	1	12	1	2.94	0.37	7	1.42
13	1.44	1	13	1	2.41	0.33	7	1.42
14	1.4	1	14	1	2.32	0.37	7.17	1.42
15	1.36	1	15	1	2.25	0.43	8	1.42
15	1.36	1	16	1	1.56	0.44	8	1.42
16	1.45	1	17	1	1.19	0.49	8	1.42
24	1.85	1	18	1	0.97	0.61	8.08	1.42
23	1.77	1	19	1	1.02	1.06	8.5	1.42
2	1	0	16	1	0.57	0.74	11.04	2.8
2	1	0	17	1	0.5	0.83	11	2.8
5	1	0	23	1	1.43	3.57	9.5	4.65
10	1.11	0	24	1	2.15	2.35	9.5	4.65
11	1.22	0	25	1	3.05	2.36	9.08	4.65
10	1.67	0	26	1	2.83	0.44	9	1.45
10	1.67	0	27	1	2.62	0.23	8.29	1.45
8	1.6	0	28	1	4.05	0.1	8.88	1.45
4	1	0	14	1	0.58	3.26	11	2.8
4	1	0	15	1	0.48	3.29	11.46	2.8
4	1	0	16	1	0.45	3.23	11.5	2.8

4	1	0	17	1	0.45	1.51	11.5	2.8
4	1	0	18	1	0.47	0.79	11.5	2.8
4	1	0	19	0	0.43	0.74	11.04	2.8
4	1	0	20	0	0.43	0.83	11	2.8
4	1	0	21	0	0.59	0.71	11	2.8
2	1	1	22	1	3.27	0.21	9	4.17
1	1	0	18	0	3.82	6.01	6.5	4.43
1	1	0	19	0	4.62	6.22	6.67	4.43
1	1	0	20	0	4.86	6.31	6.75	4.43
1	1	0	21	0	5.01	6.33	6.08	4.43
1	1	0	22	0	5.09	5.22	7.71	4.43
1	1	0	23	0	4.17	6.21	6.42	4.43
1	1	0	24	0	3.64	4.66	7.33	4.43
1	1	0	25	0	3.83	3.84	7.75	4.43
2	2	0	26	1	3.85	2.64	8.5	4.43
1	1	0	17	1	0.61	2.44	6	3.31
1	1	0	18	1	0.79	2.87	6.5	3.31
1	1	0	19	1	0.64	1.92	6.33	3.31
1	1	0	20	1	0.56	2.27	6.38	3.31
5	1.25	0	21	1	1.19	2.41	7.5	3.31
2	1	0	19	0	0.96	3.23	11.5	2.8
2	1	0	20	0	0.92	1.51	11.5	2.8
2	1	0	21	0	0.71	0.79	11.5	2.8
2	1	0	22	0	0.6	0.74	11.04	2.8
2	1	0	23	0	0.48	0.83	11	2.8
4	1.33	0	24	0	0.26	0.71	11	2.8
6	1	0	21	1	0.93	2.22	10	2.26
6	1	0	22	1	1.19	1.98	10	2.26
8	1.14	0	23	1	3.29	1.98	10	2.26
9	1.29	0	24	1	4.04	1.59	10	2.26
8	1.14	0	25	1	4.26	0.43	10	2.26
8	1.33	0	26	1	10.14	0.31	10	2.26
9	1.5	0	27	0	8.08	0.22	10	2.26
7	1.17	0	28	1	1767.97	0.43	10	2.26
8	1.14	0	23	1	3.29	3.23	11.5	2.8
9	1.29	0	24	1	4.04	1.51	11.5	2.8
8	1.14	0	25	1	4.26	0.79	11.5	2.8
8	1.33	0	26	1	10.14	0.74	11.04	2.8
9	1.5	0	27	0	8.08	0.83	11	2.8
7	1.17	0	28	1	1767.97	0.71	11	2.8
1	1	0	11	0	0.71	5.63	10.33	3.8
4	1.33	0	12	1	0.84	5.66	9.83	3.8
4	1.33	0	13	1	1.03	5.41	9	3.8
5	1.67	0	14	1	1.05	4.11	9	3.8
5	1.67	0	15	1	1.26	3.75	9	3.8
5	1.67	0	16	1	0.84	1.86	9	3.8
5	1.67	0	17	1	0.74	1.37	9	3.8
4	1.33	0	19	1	1	1.44	9	3.8
2	1	0	23	1	1.07	0.74	11.04	2.8
4	1	0	24	1	1.41	0.83	11	2.8
4	1	0	25	1	1.81	0.71	11	2.8

3	1.5	0	11	0	2.08	0.6	8	1.42
3	1	0	12	0	2.75	0.47	7.17	1.42
3	1	0	13	0	2.71	0.37	7	1.42
2	1	0	14	0	2.04	0.33	7	1.42
2	1	0	15	0	1.93	0.37	7.17	1.42
2	1	0	16	1	1.76	0.43	8	1.42
2	1	0	17	1	1.49	0.44	8	1.42
4	1	0	18	1	1.48	0.49	8	1.42
4	1.33	0	19	1	1.34	0.61	8.08	1.42
4	1.33	0	20	1	1.21	1.06	8.5	1.42
2	1	1	18	0	1.22	0.61	8.5	0.84
1	1	1	19	0	1.41	0.44	8.5	0.84
9	1.13	0	24	0	1.67	0.14	9	4.17
14	1.56	0	25	0	1.43	0.2	9	4.17
16	1.45	0	26	1	1.49	0.23	9	4.17
18	1.8	0	27	1	1.59	0.21	9	4.17
14	1.56	0	25	0	1.43	2.1	7	3.4
16	1.45	0	26	1	1.49	0.79	7	3.4
18	1.8	0	27	1	1.59	1.07	7.04	3.4
3	1	0	13	1	1.28	1.96	7.5	1.9
3	1	0	14	1	1.36	1.33	7.5	1.9
15	2.14	0	15	1	1.01	1.35	8.08	1.9
15	2.14	0	15	1	1.01	1.51	11.5	2.8
3	1.5	0	17	1	0.87	4.97	9.42	2.35
5	2.5	0	18	0	1.08	8.14	8.08	2.35
4	4	0	19	0	1.47	13.69	9	2.35
6	6	0	20	0	1.18	16.53	8.5	2.35
5	5	0	21	0	1.33	17.03	9.75	2.35
5	5	0	22	1	2.07	17.01	10.5	2.35
5	5	0	23	1	2.29	15.04	10.5	2.35
6	3	0	24	1	2.05	0.57	10.5	2.35
2	1	0	25	1	1.8	0.75	10.25	2.35
33	5.5	1	21	1	1.47	1.13	8	5.48
42	4.67	1	22	1	1.46	0.94	8.08	5.48
33	5.5	1	21	1	1.47	2.94	9.5	4.65
42	4.67	1	22	1	1.46	0.9	10.38	4.65
2	1	1	18	0	1.08	1.59	10	2.26
15	2.14	1	19	0	2.41	0.43	10	2.26
24	3.43	1	20	0	1.37	0.31	10	2.26
42	4.67	1	22	1	1.46	0.43	10	2.26
2	1	0	14	1	0.9	0.85	8	0.84
2	1	0	15	1	1	0.69	7.17	0.84
2	1	0	16	1	1.03	0.71	7	0.84
2	1	0	17	1	0.84	0.49	7	0.84
2	1	0	18	1	0.94	0.49	7.17	0.84
2	1	0	19	1	1.46	0.57	8	0.84
2	1	0	20	1	0.55	0.54	8	0.84
3	1.5	0	21	1	0.72	0.64	8.08	0.84
4	2	0	22	1	0.87	0.61	8.5	0.84
5	2.5	0	23	1	0.74	0.44	8.5	0.84
2	1	0	11	1	0.17	2.16	10	2.26

2	1	0	12	1	0.16	2.18	10	2.26
2	1	0	13	1	0.19	2.22	10	2.26
2	1	0	14	1	0.2	1.98	10	2.26
2	1	0	15	1	0.19	1.98	10	2.26
42	4.67	1	22	1	1.46	0.21	9	4.17
6	1.2	1	16	1	1.65	0.49	7.17	0.84
10	1.11	1	17	1	1.64	0.57	8	0.84
11	1.1	1	18	1	2.84	0.54	8	0.84
10	1.11	1	19	1	1.76	0.64	8.08	0.84
10	1.11	1	20	1	2.02	0.61	8.5	0.84
21	1.62	1	21	1	2.14	0.44	8.5	0.84
15	2.14	1	19	0	2.41	1.16	7.58	2.86
24	3.43	1	20	0	1.37	0.99	8	2.86
2	1	0	15	1	1.66	3.55	8.96	5.28
2	1	0	16	0	2.37	2.81	8	5.28
2	1	0	17	1	0.31	2.81	8	5.28
2	1	0	18	1	0.35	2.25	8	5.28
15	2.14	1	19	1	2.41	0.79	11.5	2.8
24	3.43	1	20	1	1.37	0.74	11.04	2.8
10	1.11	1	17	1	1.64	0.59	7.42	1.45
11	1.1	1	18	1	2.84	0.56	7.71	1.45
10	1.11	1	19	1	1.76	0.44	9	1.45
10	1.11	1	20	1	2.02	0.23	8.29	1.45
21	1.62	1	21	1	2.14	0.1	8.88	1.45
10	1.11	1	17	1	1.64	2.56	7.5	2.65
11	1.1	1	18	1	2.84	2.39	8	2.65
10	1.11	1	19	1	1.76	2	8	2.65
6	1.5	0	21	0	0.53	0.79	11.5	2.8
7	1.75	0	22	0	0.49	0.74	11.04	2.8
7	1.75	0	23	1	1.03	0.83	11	2.8
7	1.75	0	24	1	0.86	0.44	8.5	0.84
2	1	0	17	0	0.33	2.06	7.71	1.9
2	1	0	18	0	0.27	1.96	7.5	1.9
2	1	0	20	0	0.23	1.35	8.08	1.9
2	1	0	21	1	0.23	1.29	8.67	1.9
2	1	0	22	1	0.17	1.04	8.38	1.9
4	1	0	23	1	0.14	0.85	8.04	1.9
6	1.2	0	24	1	0.17	1.22	8.67	1.9
4	1.33	0	28	1	2.14	0.61	8.5	0.84
5	1.67	0	29	1	1.91	0.44	8.5	0.84
10	1.11	1	20	1	2.02	0.22	10	2.26
21	1.62	1	21	1	2.14	0.43	10	2.26
21	1.62	1	21	1	2.14	1.19	8.83	2.33
21	1.62	1	21	1	2.14	1.06	8.5	1.42
3	1	0	16	0	0.55	5.98	9.5	2.65
3	1	0	17	0	0.66	5.6	8.92	2.65
3	1	0	18	0	0.79	4.94	8.5	2.65
3	1	0	22	1	0.7	2	8	2.65
3	1	0	23	1	0.64	1.61	8	2.65
5	1.67	0	29	1	1.91	0.44	8.5	0.84
4	1.33	0	16	1	1.6	3.83	9	4.17

4	1.33	0	17	1	0.74	3.34	9	4.17
2	1	0	15	1	0.88	3.51	8.5	2.33
6	2	0	18	1	2.36	0.49	7	0.84
7	1.75	0	19	1	2.28	0.49	7.17	0.84
6	1.5	0	20	1	0.77	0.57	8	0.84
9	1.8	0	21	1	0.76	0.54	8	0.84
7	1.4	0	22	1	0.46	0.64	8.08	0.84
7	1.4	0	23	1	1.61	0.61	8.5	0.84
6	1.5	0	24	1	1.61	0.44	8.5	0.84
6	2	0	17	1	0.84	1.35	8.08	1.9
12	2.4	0	19	1	0.89	1.04	8.38	1.9
13	2.17	0	20	1	1.04	0.85	8.04	1.9
17	1.89	0	21	1	1.16	1.22	8.67	1.9
13	2.17	0	20	1	1.04	0.85	8.04	1.9
17	1.89	0	21	1	1.16	1.22	8.67	1.9
4	1.33	0	22	0	1.16	0.44	8.5	0.84
4	1	0	12	1	1.04	3.12	7.5	4.44
4	1	0	13	1	0.85	3.3	7.5	4.44
9	1.13	0	19	0	0.64	1.04	10.46	4.44
1	1	0	16	1	0.51	2.32	7.71	2.33
8	1.6	0	18	1	1.05	1.17	7.88	2.33
4	1	0	17	1	0.75	0.44	9	1.45
8	1.6	0	18	1	1.05	0.23	8.29	1.45
9	1.13	0	19	0	0.64	0.1	8.88	1.45
2	1	0	21	0	0.51	1.29	8.67	1.9
2	1	0	22	1	0.59	1.04	8.38	1.9
2	1	0	23	1	0.79	0.85	8.04	1.9
4	1	0	24	1	0.99	1.22	8.67	1.9
3	1.5	0	18	1	0.24	0.71	11	2.8
9	1.8	0	17	1	0.27	2.02	9.92	4.44
27	3.86	0	19	1	0.57	1.25	10.29	4.44
4	1.33	1	13	1	0.38	5.74	7.5	3.31
5	1.25	1	14	1	0.35	5.62	7.58	3.31
6	1.2	1	16	1	0.56	5.55	6.04	3.31
6	1.2	1	17	1	0.65	2.44	6	3.31
7	1.4	1	18	0	0.36	2.87	6.5	3.31
8	1.33	1	19	0	0.36	1.92	6.33	3.31
14	1.75	1	20	0	0.45	2.27	6.38	3.31
11	1.38	1	21	0	0.47	2.41	7.5	3.31
26	2.36	0	19	1	0.19	2.36	9.08	4.65
35	2.69	0	20	1	0.26	2.36	9.42	4.65
40	2	0	21	1	0.16	2.94	9.5	4.65
43	1.79	0	22	1	0.18	0.9	10.38	4.65
6	1.2	1	15	1	0.41	7.63	9	2.95
6	1.2	1	16	1	0.56	7.69	9	2.95
6	1.2	1	17	1	0.65	1.59	9	2.95
7	1.4	1	18	0	0.36	1.38	9	2.95
8	1.33	1	19	0	0.36	0.83	9	2.95
14	1.75	1	20	0	0.45	0.81	9	2.95
14	1.75	1	20	0	0.45	1.61	8	2.65
11	1.38	1	21	0	0.47	1.58	8.38	2.65

26	2.36	0	19	1	0.19	2.26	8.46	5.28
35	2.69	0	20	1	0.26	2.48	9.29	5.28
40	2	0	21	1	0.16	1.98	10.5	5.28
2	1	0	16	1	0.32	0.49	7	0.84
2	1	0	17	1	0.31	0.49	7.17	0.84
3	1	0	18	1	0.24	0.57	8	0.84
3	1	0	19	1	0.24	0.54	8	0.84
5	1.67	0	20	1	0.75	0.64	8.08	0.84
6	1.5	0	21	1	0.75	0.61	8.5	0.84
7	1.4	0	22	1	0.71	0.44	8.5	0.84
43	1.79	0	22	1	0.18	0.71	11	2.8
7	1.4	0	18	1	0.46	0.43	10	2.26
7	1.4	0	19	1	0.3	0.31	10	2.26
8	1.33	0	20	1	0.39	0.22	10	2.26
8	1.33	0	21	1	0.51	0.43	10	2.26
7	1.4	0	22	1	0.71	0.1	8.88	1.45
6	1.5	0	21	1	0.75	0.22	10	2.26
2	1	1	21	1	0.75	0.71	11	2.8
18	1.64	0	22	0	0.73	0.43	10	2.26
20	1.67	0	23	1	0.63	0.31	10	2.26
17	1.55	0	24	1	0.63	0.22	10	2.26
19	1.46	0	25	1	0.8	0.43	10	2.26
27	3.38	0	29	1	4.89	0.71	11	2.8
2	1	0	26	0	1.48	0.85	8.04	1.9
28	4	1	15	1	8.55	2.12	8.5	1.9
37	5.29	1	17	1	4.32	3.41	8.5	2.33
37	6.17	1	18	1	5.67	3.49	7.88	2.33
39	5.57	1	19	1	5.19	2.97	7.5	2.33
39	6.5	1	20	1	6.3	2.32	7.71	2.33
39	5.57	1	21	1	6.48	2.07	7.96	2.33
38	5.43	1	22	1	5.88	1.17	7.88	2.33
34	5.67	1	23	1	5.64	1.19	8.83	2.33
11	2.2	0	17	1	0.79	0.1	8.88	1.45
1	1	0	24	1	0.06	3.26	11	2.8
1	1	0	25	1	0.05	3.29	11.46	2.8
2	1	0	26	1	0.05	3.23	11.5	2.8
2	1	0	27	1	0.08	1.51	11.5	2.8
1	1	0	12	0	1.25	0.71	7	0.84
1	1	0	13	0	0.46	0.49	7	0.84
1	1	0	14	0	0.56	0.49	7.17	0.84
1	1	0	16	0	0.24	0.54	8	0.84
1	1	0	17	1	0.33	0.64	8.08	0.84
1	1	0	18	0	0.46	0.61	8.5	0.84
2	1	0	19	1	0.36	0.44	8.5	0.84
12	1.71	0	17	0	1.88	2.07	7.96	2.33
13	2.17	0	18	0	1.35	1.17	7.88	2.33
21	1.62	0	19	0	1.57	1.19	8.83	2.33
2	1	1	21	1	1.89	1.96	7.5	1.9
2	1	1	22	1	1.87	1.33	7.5	1.9
3	1	1	23	1	1.62	1.35	8.08	1.9
4	1	1	24	1	1.5	1.29	8.67	1.9

5	1	1	25	1	1.32	1.04	8.38	1.9
5	1	1	26	1	1.36	0.85	8.04	1.9
6	1	1	27	0	1.53	1.22	8.67	1.9
4	1.33	0	10	1	2.5	0.85	8	0.84
4	1.33	0	11	1	1.75	0.69	7.17	0.84
5	1.25	0	12	1	1.39	0.71	7	0.84
7	1.4	0	13	1	1.54	0.49	7	0.84
7	1.4	0	14	1	1.53	0.49	7.17	0.84
7	1.4	0	15	1	1.33	0.57	8	0.84
9	1.5	0	16	1	1.88	0.54	8	0.84
2	1	0	15	1	1.27	2.33	6.54	1.79
2	1	0	16	1	1.67	2.55	6.5	1.79
4	2	0	17	1	1.13	1.99	7.33	1.79
6	1	1	27	0	1.53	0.44	8.5	0.84
3	1	0	22	0	1.38	1.22	8.67	1.9
2	1	0	19	1	1.18	0.2	9	4.17
2	1	0	20	1	1.32	0.23	9	4.17
1	1	0	21	0	1.32	0.21	9	4.17
1	1	0	15	0	0.73	7.3	6.29	3.71
1	1	0	16	0	0.62	7.51	6	3.71
1	1	0	17	0	0.75	5.92	7.13	3.71
1	1	0	18	1	0.87	5.68	7.96	3.71
2	1	0	19	1	1.18	5.06	8	3.71
2	1	0	20	1	1.31	5.47	7.63	3.71
7	1.4	1	15	0	1.11	1.04	8.38	1.9
7	1.4	1	16	0	1.45	0.85	8.04	1.9
7	1.4	1	17	0	1.72	1.22	8.67	1.9
1	1	0	16	0	0.15	0.49	8	1.42
1	1	0	17	0	0.36	0.61	8.08	1.42
1	1	0	18	0	0.22	1.06	8.5	1.42
2	1	0	9	1	0.82	8.31	5.88	3.71
2	1	0	10	1	1.25	8.45	5.67	3.71
2	1	0	11	1	1.3	8.02	6.5	3.71
2	1	0	12	1	1	7.3	6.29	3.71
2	1	0	13	1	1.03	7.51	6	3.71
3	1	0	14	1	0.98	5.92	7.13	3.71
3	1	0	15	1	0.96	5.68	7.96	3.71
3	1	0	16	1	0.98	5.06	8	3.71
1	1	0	17	1	0.94	5.47	7.63	3.71
3	1	0	14	1	0.98	2.56	7.5	2.65
3	1	0	15	1	0.96	2.39	8	2.65
3	1	0	16	1	0.98	2	8	2.65
6	1.2	0	26	1	1.46	0.23	8.29	1.45
6	1.2	0	27	1	1.06	0.1	8.88	1.45
6	1.2	0	26	1	1.46	1.17	7.88	2.33
6	1.2	0	27	1	1.06	1.19	8.83	2.33
2	1	1	18	1	0.45	0.79	11.5	2.8
3	1	1	19	1	0.7	0.74	11.04	2.8
3	1	1	20	1	0.89	0.83	11	2.8
5	1.25	1	21	1	0.73	0.71	11	2.8
2	1	0	13	1	0.78	3.31	11.5	2.8

2	1	0	14	1	0.7	3.25	11.5	2.8
2	1	0	15	1	0.47	3.26	11	2.8
47	1.12	0	18	0	1.07	0.59	8	5.83
46	1.05	0	19	0	0.87	1.81	7.13	5.83
24	1	0	16	1	0.84	17.01	10.5	2.35
37	1.06	0	17	1	1.03	15.04	10.5	2.35
47	1.12	0	18	0	1.07	0.57	10.5	2.35
46	1.05	0	19	0	0.87	0.75	10.25	2.35
24	1	0	16	1	0.84	2.36	9.08	4.65
37	1.06	0	17	1	1.03	2.36	9.42	4.65
47	1.12	0	18	0	1.07	2.94	9.5	4.65
46	1.05	0	19	0	0.87	0.9	10.38	4.65
19	1.46	0	16	1	0.67	1.38	9	3.8
19	1.46	0	17	1	0.61	1.44	9	3.8
19	1.36	0	18	1	0.55	1.01	10.04	3.8
13	1	0	15	1	0.76	2.56	7.5	2.65
24	1	0	16	1	0.84	2.39	8	2.65
37	1.06	0	17	1	1.03	2	8	2.65
47	1.12	0	18	0	1.07	1.61	8	2.65
46	1.05	0	19	0	0.87	1.58	8.38	2.65
6	1	0	12	1	0.8	2.22	10	2.26
8	1.14	0	13	1	0.98	1.98	10	2.26
11	1.38	0	14	1	1.11	1.98	10	2.26
14	1.56	0	15	1	1.04	1.59	10	2.26
14	1.75	0	16	1	1.11	0.43	10	2.26
14	1.75	0	17	1	0.78	0.31	10	2.26
12	1.5	0	18	1	0.96	0.22	10	2.26
12	1.71	0	19	1	1.15	0.43	10	2.26
37	1.06	0	17	1	1.03	1.99	7.33	1.79
47	1.12	0	18	0	1.07	1.92	8	1.79
46	1.05	0	19	0	0.87	1.59	8.46	1.79
37	1.06	0	17	1	1.03	0.31	10	2.26
47	1.12	0	18	0	1.07	0.22	10	2.26
46	1.05	0	19	0	0.87	0.43	10	2.26
37	1.06	0	17	1	1.03	2.48	9.29	5.28
47	1.12	0	18	0	1.07	1.98	10.5	5.28
46	1.05	0	19	0	0.87	1.18	10.5	5.28
47	1.12	0	18	0	1.07	1.38	7.71	3.2
46	1.05	0	19	0	0.87	1.31	8.25	3.2
24	1	0	16	1	0.84	1.99	6.75	3.23
37	1.06	0	17	1	1.03	1.99	6.5	3.23
47	1.12	0	18	0	1.07	2.06	7.13	3.23
46	1.05	0	19	0	0.87	2.21	8	3.23
47	1.12	0	18	0	1.07	0.79	7	3.4
46	1.05	0	19	0	0.87	1.07	7.04	3.4
47	1.12	0	18	0	1.07	1.17	7.88	2.33
46	1.05	0	19	0	0.87	1.19	8.83	2.33
11	1.38	0	16	0	1.05	2.56	7.5	2.65
12	1.5	0	17	0	0.66	2.39	8	2.65
11	1.57	0	18	0	0.81	2	8	2.65
14	1.56	0	19	0	1.25	1.61	8	2.65

17	1.89	0	20	0	0.92	1.58	8.38	2.65
37	1.06	0	17	1	1.03	1.92	6.33	3.31
47	1.12	0	18	0	1.07	2.27	6.38	3.31
46	1.05	0	19	0	0.87	2.41	7.5	3.31
47	1.12	0	18	0	1.07	1.25	8	5.07
46	1.05	0	19	0	0.87	1.13	8.08	5.07
24	1	0	16	1	0.84	2.02	9.92	4.44
37	1.06	0	17	1	1.03	2.2	9.67	4.44
47	1.12	0	18	0	1.07	1.25	10.29	4.44
46	1.05	0	19	0	0.87	1.04	10.46	4.44
37	1.06	0	17	1	1.03	1.04	8.38	1.9
47	1.12	0	18	0	1.07	0.85	8.04	1.9
46	1.05	0	19	0	0.87	1.22	8.67	1.9
37	1.06	0	17	1	1.03	0.44	9	1.45
47	1.12	0	18	0	1.07	0.23	8.29	1.45
46	1.05	0	19	0	0.87	0.1	8.88	1.45
16	1.6	0	14	1	0.75	2.97	7.5	2.33
16	1.6	0	15	1	0.7	2.32	7.71	2.33
19	1.46	0	16	1	0.67	2.07	7.96	2.33
19	1.46	0	17	1	0.61	1.17	7.88	2.33
19	1.36	0	18	1	0.55	1.19	8.83	2.33
16	1.6	0	14	1	0.75	1.35	8.08	1.9
16	1.6	0	15	1	0.7	1.29	8.67	1.9
19	1.46	0	16	1	0.67	1.04	8.38	1.9
19	1.46	0	17	1	0.61	0.85	8.04	1.9
19	1.36	0	18	1	0.55	1.22	8.67	1.9
8	1.14	0	13	1	0.98	0.49	7	0.84
11	1.38	0	14	1	1.11	0.49	7.17	0.84
14	1.56	0	15	1	1.04	0.57	8	0.84
14	1.75	0	16	1	1.11	0.54	8	0.84
14	1.75	0	17	1	0.78	0.64	8.08	0.84
12	1.5	0	18	1	0.96	0.61	8.5	0.84
12	1.71	0	19	1	1.15	0.44	8.5	0.84
3	1.5	0	19	0	0.91	0.23	9	4.17
11	1.38	0	16	0	1.05	1.51	11.5	2.8
12	1.5	0	17	0	0.66	0.79	11.5	2.8
11	1.57	0	18	0	0.81	0.74	11.04	2.8
14	1.56	0	19	0	1.25	0.83	11	2.8
17	1.89	0	20	0	0.92	0.71	11	2.8
11	1.38	0	16	0	1.05	1.35	8.08	1.9
12	1.5	0	17	0	0.66	1.29	8.67	1.9
11	1.57	0	18	0	0.81	1.04	8.38	1.9
14	1.56	0	19	0	1.25	0.85	8.04	1.9
17	1.89	0	20	0	0.92	1.22	8.67	1.9
2	1	0	18	1	0.16	1.98	10	2.26
2	1	0	19	1	0.24	1.98	10	2.26
5	1.25	0	22	1	0.13	0.31	10	2.26
14	1.56	0	19	0	1.25	0.61	8.5	0.84
17	1.89	0	20	0	0.92	0.44	8.5	0.84
5	1.25	0	22	1	0.13	0.74	11.04	2.8
4	1.33	0	23	1	0.27	0.83	11	2.8

4	1.33	0	24	1	0.22	0.71	11	2.8
6	1	0	12	1	0.8	3.26	11	2.8
8	1.14	0	13	1	0.98	3.29	11.46	2.8
11	1.38	0	14	1	1.11	3.23	11.5	2.8
14	1.56	0	15	1	1.04	1.51	11.5	2.8
14	1.75	0	16	1	1.11	0.79	11.5	2.8
14	1.75	0	17	1	0.78	0.74	11.04	2.8
12	1.5	0	18	1	0.96	0.83	11	2.8
12	1.71	0	19	1	1.15	0.71	11	2.8
7	1.4	0	13	1	1.1	0.56	7.71	1.45
8	1.6	0	14	1	1.21	0.44	9	1.45
6	1.5	0	15	1	1.22	0.23	8.29	1.45
6	1.5	0	16	0	0.91	0.1	8.88	1.45
1	1	0	13	1	0.26	3.23	11.5	2.8
1	1	0	14	1	0.14	1.51	11.5	2.8
2	1	0	15	1	0.16	0.79	11.5	2.8
5	1.67	0	16	1	0.46	0.74	11.04	2.8
4	1.33	0	17	1	0.43	0.83	11	2.8
4	1.33	0	18	1	0.34	0.71	11	2.8
5	1.67	0	16	1	0.46	0.74	11.04	2.8
4	1.33	0	17	1	0.43	0.83	11	2.8
4	1.33	0	18	1	0.34	0.71	11	2.8
4	1	1	24	0	0.78	0.44	8.5	0.84
4	1	1	24	0	0.78	0.21	9	4.17
5	1.67	0	18	1	0.33	1.59	10	2.26
4	1.33	0	19	1	0.29	0.43	10	2.26
7	1.75	0	21	1	0.24	0.22	10	2.26
9	2.25	0	22	1	0.23	0.43	10	2.26
4	1.33	0	17	1	0.27	3.49	7.88	2.33
7	1.75	0	21	1	0.24	1.17	7.88	2.33
9	2.25	0	22	1	0.23	1.19	8.83	2.33
1	1	0	17	1	0.83	2.3	7	3.95
1	1	0	18	1	1.17	2.81	7	3.95
1	1	0	19	1	1.09	2.04	7	3.95
1	1	0	20	1	1.21	1.02	8.67	3.95
18	2.57	0	18	1	0.72	0.31	10	2.26
17	2.43	0	19	1	0.69	0.22	10	2.26
16	2.29	0	20	1	0.53	0.43	10	2.26
3	1	1	19	0	1.85	0.1	8.88	1.45
3	1	1	19	0	1.85	0.21	9	4.17
1	1	1	17	0	1.83	0.44	9	1.45
2	1	1	18	1	1.97	0.23	8.29	1.45
5	1.25	0	20	1	0.39	1.19	8.83	2.33
39	4.33	0	25	1	3.61	5.87	7.88	3.2
50	5	0	26	1	4.47	5.36	7.5	3.2
42	3.5	0	27	1	2.84	3.94	7.5	3.2
46	3.54	0	28	1	3.44	2.91	7.5	3.2
64	4.57	0	29	1	3.36	1.08	7.67	3.2
66	4.71	0	30	1	2.51	1.7	8	3.2
64	4	0	31	1	1.93	1.38	7.71	3.2
97	5.71	0	32	1	1.47	1.31	8.25	3.2

50	5	0	26	1	4.47	3.29	11.46	2.8
42	3.5	0	27	1	2.84	3.23	11.5	2.8
46	3.54	0	28	1	3.44	1.51	11.5	2.8
64	4.57	0	29	1	3.36	0.79	11.5	2.8
66	4.71	0	30	1	2.51	0.74	11.04	2.8
64	4	0	31	1	1.93	0.83	11	2.8
97	5.71	0	32	1	1.47	0.71	11	2.8
41	4.1	0	16	1	1	0.94	8.08	5.48
46	3.54	0	28	1	3.44	2.97	7.5	2.33
64	4.57	0	29	1	3.36	2.32	7.71	2.33
66	4.71	0	30	1	2.51	2.07	7.96	2.33
64	4	0	31	1	1.93	1.17	7.88	2.33
97	5.71	0	32	1	1.47	1.19	8.83	2.33
64	4.57	0	29	1	3.36	2.87	6.5	3.31
66	4.71	0	30	1	2.51	1.92	6.33	3.31
64	4	0	31	1	1.93	2.27	6.38	3.31
97	5.71	0	32	1	1.47	2.41	7.5	3.31
97	5.71	0	32	1	1.47	5.79	8.08	3.71
13	1.86	0	24	1	0.45	0.44	8.5	0.84
2	1	0	17	1	1.28	0.51	9	4.17
15	2.14	0	11	1	0.75	1.33	7.5	1.9
20	2.86	0	12	1	0.62	1.35	8.08	1.9
24	3.43	0	13	1	0.79	1.29	8.67	1.9
31	3.44	0	14	1	0.98	1.04	8.38	1.9
36	4	0	15	1	1.18	0.85	8.04	1.9
41	4.1	0	16	1	1	1.22	8.67	1.9
31	3.44	0	14	1	0.98	0.44	9	1.45
36	4	0	15	1	1.18	0.23	8.29	1.45
41	4.1	0	16	1	1	0.1	8.88	1.45
10	2.5	0	9	1	1.06	0.71	7	0.84
12	2.4	0	10	1	0.84	0.49	7	0.84
15	2.14	0	11	1	0.75	0.49	7.17	0.84
20	2.86	0	12	1	0.62	0.57	8	0.84
24	3.43	0	13	1	0.79	0.54	8	0.84
31	3.44	0	14	1	0.98	0.64	8.08	0.84
36	4	0	15	1	1.18	0.61	8.5	0.84
41	4.1	0	16	1	1	0.44	8.5	0.84
2	1	0	33	1	2.05	0.2	9	4.17
3	1	0	34	1	2.55	0.23	9	4.17
3	1	0	35	1	2.56	0.21	9	4.17
4	1.94	0	16	1	0.73	1.04	8.38	1.9
4	1.94	0	16	1	0.73	2.07	7.96	2.33
8	1.6	0	19	1	1.5	0.1	8.88	1.45
1	1.78	0	14	1	1.48	1.51	11.5	2.8
2	1.88	0	15	1	0.94	0.79	11.5	2.8
2	1.88	0	16	1	1	0.74	11.04	2.8
3	1.99	0	17	1	0.93	0.83	11	2.8
8	2.07	0	18	1	0.95	0.71	11	2.8
3	1.99	0	17	1	0.93	0.22	10	2.26
8	2.07	0	18	1	0.95	0.43	10	2.26
8	2.07	0	18	1	0.95	1.22	8.67	1.9

1	1	1	19	1	0.44	0.74	11.04	2.8
2	1	0	24	1	0.55	0.71	11	2.8
4	1.33	0	14	1	1.15	2.17	9.5	2.48
3	1	0	17	1	1.18	0.1	8.88	1.45
1	1.75	0	13	0	0.44	6.31	6.75	4.43
1	1.73	0	14	0	0.37	6.33	6.08	4.43
1	1.74	0	16	0	0.3	6.21	6.42	4.43
1	1.93	0	19	1	0.16	0.21	9	4.17
3	1	0	21	1	0.24	1.19	8.83	2.33
6	1.94	0	18	1	0.77	2.21	8	3.23
2	1.83	0	10	1	0.19	2.69	6	5.13
2	1.84	0	11	1	0.19	2.64	6	5.13
2	1.86	0	12	1	0.45	2.16	6	5.13
2	1.83	0	13	0	0.41	2.04	6	5.13
4	1.33	0	18	1	0.83	0.31	10	2.26
4	1.33	0	19	1	1.01	0.22	10	2.26
4	1.33	0	20	1	1.19	0.43	10	2.26
11.7	2.13	0	17	1	0.63	0.22	10	2.26
3	2.01	1	20	1	1.23	2.07	7.96	2.33
6	2.06	1	21	1	1.48	1.17	7.88	2.33
7.2	2.16	1	22	0	1.97	1.19	8.83	2.33
8	1.97	0	19	1	4.54	0.43	10	2.26
7	2	0	20	1	1.84	0.31	10	2.26
11.7	2.13	0	17	1	0.63	0.83	11	2.8
11.7	2.13	0	17	1	0.63	0.23	8.29	1.45
6	2.06	1	21	1	1.48	0.83	11	2.8
7.2	2.16	1	22	0	1.97	0.71	11	2.8
2	1	1	16	1	1.44	0.57	8	0.84
2	1	1	17	1	1.73	0.54	8	0.84
2	1	1	18	1	1.88	0.64	8.08	0.84
2	1	1	20	1	1.44	0.44	8.5	0.84
4	1	0	11	1	0.64	3.34	9	4.17
5	1.25	0	12	1	0.6	2.82	9	4.17
24	2.21	0	21	1	1.22	1.42	9.5	4.65
3.9	1.88	0	20	1	1.13	0.44	8.5	0.84
8	2.67	0	21	0	0.67	1.75	8	2.86
8	2.67	0	21	0	0.67	0.21	9	4.17
3.9	2.1	0	17	1	0.25	0.83	11	2.8
3	2.12	0	18	1	0.14	0.71	11	2.8
5.1	2.12	0	22	1	0.12	0.22	10	2.26
6	1.93	0	22	1	0.86	0.9	10.38	4.65
6	1.93	0	22	1	0.86	0.1	8.88	1.45
38.5	2.53	1	19	1	0.67	2.09	7.63	2.53
38.5	2.53	1	19	1	0.67	0.94	8.08	5.48
29.7	2.29	1	18	1	0.68	0.59	8	5.83
38.5	2.53	1	19	1	0.67	1.81	7.13	5.83
20.4	1.74	1	16	1	0.69	1.37	9.08	4.65
27.6	2.07	1	17	1	0.69	1.35	9.42	4.65
29.7	2.29	1	18	1	0.68	1.42	9.5	4.65
38.5	2.53	1	19	1	0.67	0.9	10.38	4.65
20.4	1.74	1	16	1	0.69	2.39	8	2.65

27.6	2.07	1	17	1	0.69	2	8	2.65
29.7	2.29	1	18	1	0.68	1.61	8	2.65
38.5	2.53	1	19	1	0.67	1.58	8.38	2.65
20.4	1.74	1	16	1	0.69	2.55	6.5	1.79
27.6	2.07	1	17	1	0.69	1.99	7.33	1.79
29.7	2.29	1	18	1	0.68	1.92	8	1.79
38.5	2.53	1	19	1	0.67	1.59	8.46	1.79
20.4	1.74	1	16	1	0.69	0.43	10	2.26
27.6	2.07	1	17	1	0.69	0.31	10	2.26
29.7	2.29	1	18	1	0.68	0.22	10	2.26
38.5	2.53	1	19	1	0.67	0.43	10	2.26
1	1.72	0	12	1	0.81	2.43	7.5	1.79
2	1.77	0	13	1	0.95	2.33	7.63	1.79
2	1.77	0	14	1	1.03	2.33	6.54	1.79
2	1.73	0	15	1	1.05	2.55	6.5	1.79
1	1.75	0	16	1	1.36	1.99	7.33	1.79
3	2.16	0	17	1	1.28	1.92	8	1.79
4	2.17	0	18	1	1.33	1.59	8.46	1.79
27.6	2.07	1	17	1	0.69	2.48	9.29	5.28
29.7	2.29	1	18	1	0.68	1.98	10.5	5.28
38.5	2.53	1	19	1	0.67	1.18	10.5	5.28
38.5	2.53	1	19	1	0.67	1.31	8.25	3.2
38.5	2.53	1	19	1	0.67	0.71	11	2.8
38.5	2.53	1	19	1	0.67	1.19	8.83	2.33
20.4	1.74	1	16	1	0.69	2.87	6.5	3.31
27.6	2.07	1	17	1	0.69	1.92	6.33	3.31
29.7	2.29	1	18	1	0.68	2.27	6.38	3.31
38.5	2.53	1	19	1	0.67	2.41	7.5	3.31
27.6	2.07	1	17	1	0.69	1.22	8	5.07
29.7	2.29	1	18	1	0.68	1.25	8	5.07
38.5	2.53	1	19	1	0.67	1.13	8.08	5.07
27.6	2.07	1	17	1	0.69	2.2	9.67	4.44
29.7	2.29	1	18	1	0.68	1.25	10.29	4.44
38.5	2.53	1	19	1	0.67	1.04	10.46	4.44
2	1.86	1	11	0	1.51	1.87	9.42	4.65
2	1.86	1	12	0	1.93	1.45	9	4.65
29.7	2.29	1	18	1	0.68	0.66	10	5.44
38.5	2.53	1	19	1	0.67	0.59	10.46	5.44
20.4	1.74	1	16	1	0.69	1.29	8.67	1.9
27.6	2.07	1	17	1	0.69	1.04	8.38	1.9
29.7	2.29	1	18	1	0.68	0.85	8.04	1.9
38.5	2.53	1	19	1	0.67	1.22	8.67	1.9
38.5	2.53	1	19	1	0.67	0.1	8.88	1.45
3.9	1.89	0	18	0	0.77	1.04	10	5.44
3.9	1.9	0	19	1	0.82	0.84	10	5.44
3.9	1.87	0	20	1	0.82	0.66	10	5.44
1	1	0	20	1	0.61	1.51	11.5	2.8
2	1	0	21	1	0.45	0.79	11.5	2.8
3	1	0	22	1	0.35	0.74	11.04	2.8
4	1	0	23	1	0.36	0.83	11	2.8
7	2.33	0	17	0	0.31	0.74	11.04	2.8

23	1.92	0	18	0	0.37	0.83	11	2.8
23	1.92	0	19	0	0.47	0.71	11	2.8
23	1.92	0	18	0	0.37	0.61	8.5	0.84
23	1.92	0	19	0	0.47	0.44	8.5	0.84
2	1.92	0	19	0	0.3	4.69	7.67	2.65
2	1.93	0	20	0	0.27	2.56	7.5	2.65
3	1.93	0	21	0	0.24	2.39	8	2.65
6	1.95	0	22	0	0.58	2	8	2.65
6	1.97	0	23	1	0.52	1.61	8	2.65
6	1.99	0	24	1	0.57	1.58	8.38	2.65
1	1.66	0	15	1	0.68	0.56	7.71	1.45
1	1.69	0	16	1	0.89	0.44	9	1.45
2	1.71	0	17	1	0.7	0.23	8.29	1.45
2	1.75	0	18	1	0.61	0.1	8.88	1.45
3	1.74	0	15	1	1.66	1.59	10	2.26
4	1.9	0	16	1	1.91	0.43	10	2.26
5.2	1.83	0	17	1	2.38	0.31	10	2.26
5.2	1.88	0	18	1	3.41	0.22	10	2.26
3	1.74	0	15	1	1.66	1.51	11.5	2.8
4	1.9	0	16	1	1.91	0.79	11.5	2.8
5.2	1.83	0	17	1	2.38	0.74	11.04	2.8
5.2	1.88	0	18	1	3.41	0.83	11	2.8
5	1.73	0	12	1	1.03	1.51	11.5	2.8
6	1.76	0	13	1	0.78	0.79	11.5	2.8
7	1.79	0	14	1	1	0.74	11.04	2.8
7.7	1.96	0	15	1	1.28	0.83	11	2.8
12.6	1.95	0	16	1	1.36	0.71	11	2.8
5	1.73	0	12	1	1.03	1.59	10	2.26
6	1.76	0	13	1	0.78	0.43	10	2.26
7	1.79	0	14	1	1	0.31	10	2.26
7.7	1.96	0	15	1	1.28	0.22	10	2.26
12.6	1.95	0	16	1	1.36	0.43	10	2.26
6	1.76	0	13	1	0.78	0.54	8	0.84
7	1.79	0	14	1	1	0.64	8.08	0.84
7.7	1.96	0	15	1	1.28	0.61	8.5	0.84
12.6	1.95	0	16	1	1.36	0.44	8.5	0.84
12.6	1.95	0	16	1	1.36	1.22	8.67	1.9
12.6	1.95	0	16	1	1.36	1.31	8.25	3.2
2	1	1	15	0	1.16	3.99	6.5	3.1
2	1	1	17	0	1.35	2.62	6.83	3.1
2	1	1	18	0	1.96	2.85	7.5	3.1
12	1.71	0	16	1	0.63	0.85	8.04	1.9
12	1.71	0	17	1	0.89	1.22	8.67	1.9
12	1.71	0	17	1	0.89	1.58	8.38	2.65
2	1	0	12	1	0.16	3.26	11	2.8
2	1	0	13	1	0.19	3.29	11.46	2.8
4	1.33	0	14	1	0.59	3.23	11.5	2.8
4	1.33	0	15	1	0.74	1.51	11.5	2.8
3	1	0	16	1	0.75	0.79	11.5	2.8
3	1	0	17	1	0.55	0.74	11.04	2.8
3	1	0	18	1	0.17	0.83	11	2.8

3	1	0	19	1	0.19	0.71	11	2.8
4	1.33	0	14	1	0.59	1.14	7	5.83
4	1.33	0	15	1	0.74	1.25	7.5	5.83
3	1	0	16	1	0.75	0.99	8	5.83
2	1.94	0	19	1	1.21	3.49	7.88	2.33
2	1.94	0	20	1	1.33	2.97	7.5	2.33
2	1.95	0	21	1	1.99	2.32	7.71	2.33
1	1.86	0	22	1	0.74	2.07	7.96	2.33
2	2.06	0	23	1	0.82	1.17	7.88	2.33
4	1.33	0	14	1	0.59	1.14	7	5.83
4	1.33	0	15	1	0.74	1.25	7.5	5.83
3	1	0	17	1	0.55	0.76	8	5.83
3	1	0	18	1	0.17	0.59	8	5.83
3	1	0	19	1	0.19	1.81	7.13	5.83
6	2	0	14	1	0.37	7.5	9	3.97
7	2.33	0	15	1	0.52	7.56	10	3.97
7	2.33	0	16	1	0.63	3.64	10	3.97
7	2.33	0	17	1	0.26	1.72	10	3.97
7	2.33	0	18	1	0.39	1	10	3.97
7	2.33	0	19	0	0.33	1.04	9.29	3.97
8	2.67	0	20	0	0.34	1.05	10.13	3.97
8	1.87	0	14	1	3.87	1.29	8.67	1.9
9	2.11	0	15	1	3.86	1.04	8.38	1.9
10.2	2.12	0	16	1	3.42	0.85	8.04	1.9
21.7	2.18	0	17	1	2.64	1.22	8.67	1.9
8	1.87	0	14	1	3.87	0.79	11.5	2.8
9	2.11	0	15	1	3.86	0.74	11.04	2.8
10.2	2.12	0	16	1	3.42	0.83	11	2.8
21.7	2.18	0	17	1	2.64	0.71	11	2.8
10.2	2.12	0	16	1	3.42	0.22	10	2.26
21.7	2.18	0	17	1	2.64	0.43	10	2.26
4	1.33	0	16	1	0.76	1.51	11.5	2.8
4	1.62	0	26	1	0.46	0.85	8.04	1.9
5	1.7	0	27	1	0.55	1.22	8.67	1.9
4	1.33	0	16	1	0.76	1.59	10	2.26
3	1.5	0	17	1	0.2	0.43	10	2.26
3	1.5	0	18	1	0.21	0.31	10	2.26
4	2	0	19	1	0.2	0.22	10	2.26
4	2	0	20	1	0.33	0.43	10	2.26
20	2.22	0	20	0	1.04	1.58	8.38	2.65
5	1.7	0	27	1	0.55	0.44	8.5	0.84
3	1.96	0	25	1	1.17	0.71	11	2.8
6	2.11	1	17	1	0.41	1.29	8.67	1.9
9	2.16	1	18	1	0.51	1.04	8.38	1.9
9	2.16	1	18	1	0.51	2.07	7.96	2.33
1	1	0	30	1	0.19	0.74	11.04	2.8
4	1.33	0	20	0	0.71	2.36	9.08	4.65
4	1.33	0	21	0	0.37	2.36	9.42	4.65
4	1.33	0	22	0	0.4	2.94	9.5	4.65
4	1.33	0	23	0	0.38	0.9	10.38	4.65
1	1.83	0	14	1	0.43	0.44	9	1.45

1	1.84	0	15	1	0.69	0.23	8.29	1.45
1	1	0	20	1	1.79	1.98	10	2.26
5	1.66	0	22	0	1.93	0.43	10	2.26
4	1.41	0	23	0	2	0.31	10	2.26
3	1.59	0	22	0	1.93	2.32	7.71	2.33
5	1.66	0	23	0	2	2.07	7.96	2.33
4	1.41	0	24	0	2.32	1.17	7.88	2.33
4	1.45	0	25	1	2	1.19	8.83	2.33
5	1.66	0	23	0	2	0.64	8.08	0.84
4	1.41	0	24	0	2.32	0.61	8.5	0.84
4	1.45	0	25	1	2	0.44	8.5	0.84
2	1.99	0	17	1	0.82	1.58	8.38	2.65
2	1.61	1	23	1	1.87	0.56	7.71	1.45
3	1.88	1	24	1	1.96	0.44	9	1.45
3	1.94	1	25	1	2.42	0.23	8.29	1.45
3	1.94	1	26	0	2.65	0.1	8.88	1.45
3	1.5	0	21	0	0.57	0.44	8	1.42
3	1.5	0	22	1	0.56	0.49	8	1.42
3	1.5	0	23	0	0.52	0.61	8.08	1.42
3	1.5	0	24	1	0.53	1.06	8.5	1.42
2	1.61	1	23	1	1.87	0.14	9	4.17
3	1.88	1	24	1	1.96	0.2	9	4.17
3	1.94	1	25	1	2.42	0.23	9	4.17
3	1.94	1	26	0	2.65	0.21	9	4.17
5	1	0	13	0	1.28	1.96	7.5	1.9
13	1.63	0	17	0	2.04	1.04	8.38	1.9
3	1.88	1	24	1	1.96	0.99	8	2.86
3	1.94	1	25	1	2.42	1	8	2.86
3	1.94	1	26	0	2.65	1.75	8	2.86
12	1.5	0	18	0	2.14	0.85	8.04	1.9
15	1.67	0	19	0	1.88	1.22	8.67	1.9
13	1.63	0	17	0	2.04	2.36	9.42	4.65
12	1.5	0	18	0	2.14	2.94	9.5	4.65
15	1.67	0	19	0	1.88	0.9	10.38	4.65
15	1.67	0	19	0	1.88	0.43	10	2.26
2	1	1	15	1	0.28	6.22	6.67	4.43
12	1.33	1	19	1	1.11	6.21	6.42	4.43
14	1.4	1	20	1	1.17	4.66	7.33	4.43
15	1.67	1	21	1	1.28	3.84	7.75	4.43
11	1.38	1	22	1	1.32	2.64	8.5	4.43
7	1.75	0	20	1	0.22	1.98	10	2.26
11	1.83	0	21	1	0.26	1.59	10	2.26
12	2	0	22	1	0.37	0.43	10	2.26
11	2.2	0	23	1	0.27	0.31	10	2.26
9	1.8	0	24	1	0.38	0.22	10	2.26
18	2.57	0	25	1	0.41	0.43	10	2.26
14	1.4	1	20	1	1.17	1.99	7.33	1.79
15	1.67	1	21	1	1.28	1.92	8	1.79
11	1.38	1	22	1	1.32	1.59	8.46	1.79
2	1	0	14	1	0.54	0.66	8.5	1.45
2	1	0	17	1	0.5	2.07	7.96	2.33

2	1	0	18	1	0.53	1.17	7.88	2.33
2	1	0	19	1	0.48	1.19	8.83	2.33
6	1.5	0	20	1	0.14	0.79	11.5	2.8
6	1.5	0	21	1	0.44	0.74	11.04	2.8
9	3	0	22	1	0.18	0.83	11	2.8
11	2.2	0	23	1	0.23	0.71	11	2.8
3	1.5	0	20	1	0.71	0.43	10	2.26
7	1.75	0	20	1	0.22	1.98	10	2.26
11	1.83	0	21	1	0.26	1.59	10	2.26
12	2	0	22	1	0.37	0.43	10	2.26
11	1.83	0	21	1	0.26	1.51	11.5	2.8
12	2	0	22	1	0.37	0.79	11.5	2.8
11	2.2	0	23	1	0.27	0.74	11.04	2.8
9	1.8	0	24	1	0.38	0.83	11	2.8
18	2.57	0	25	1	0.41	0.71	11	2.8
3	1.5	0	20	1	0.71	0.43	10	2.26
5	1	1	18	1	5.66	1.17	7.88	2.33
4	1	1	17	1	7.16	0.44	9	1.45
5	1	1	18	1	5.66	0.23	8.29	1.45
4	1	1	19	1	7.45	1.19	8.83	2.33
5	1.67	0	16	1	0.47	0.21	9	4.17
1	1	0	17	0	0.25	0.59	8	5.83
1	1	0	18	0	0.33	1.81	7.13	5.83
1	1	1	15	0	1.22	2.35	9.5	4.65
1	1	1	16	0	1.99	2.36	9.08	4.65
1	1	1	17	0	1.89	2.36	9.42	4.65
1	1	1	18	1	1.27	2.94	9.5	4.65
1	1	1	19	1	0.99	0.9	10.38	4.65
5	1.75	0	14	1	0.39	1.35	8.08	1.9
5	1.74	0	15	1	0.13	1.29	8.67	1.9
1	1.88	0	21	1	0.85	1.04	10.46	4.44
18	1.64	0	17	1	1.97	3.25	11.5	2.8
8	1.33	0	23	1	2.15	0.74	11.04	2.8
8	1.33	0	24	1	2.43	0.83	11	2.8
1	1.71	0	21	1	0.17	2.09	8.92	4.44
2	1.81	0	22	1	0.11	2.02	9.92	4.44
4	1.95	0	23	1	0.18	2.2	9.67	4.44
3	1.94	0	24	1	0.28	1.25	10.29	4.44
4	1.95	0	25	1	0.37	1.04	10.46	4.44
18	1.64	0	17	1	1.97	2.12	8.5	1.9
9	1.5	0	18	1	2.1	2.06	7.71	1.9
9	1.5	0	19	1	2.37	1.96	7.5	1.9
9	1.5	0	20	1	2.57	1.33	7.5	1.9
9	1.5	0	21	1	2.46	1.35	8.08	1.9
5	1.67	0	22	1	1.75	1.29	8.67	1.9
8	1.33	0	23	1	2.15	1.04	8.38	1.9
8	1.33	0	24	1	2.43	0.85	8.04	1.9
5	1.67	0	25	1	2.34	1.22	8.67	1.9
18	1.64	0	17	1	1.97	6.39	8.13	3.2
18	1.64	0	17	1	1.97	3.63	9.42	4.44
9	1.5	0	18	1	2.1	3.12	7.5	4.44

9	1.5	0	19	1	2.37	3.3	7.5	4.44
9	1.5	0	20	1	2.57	3.8	8.13	4.44
9	1.5	0	21	1	2.46	2.09	8.92	4.44
2	1.81	0	22	1	0.1	1.29	8.67	1.9
4	1.95	0	23	1	0.15	1.04	8.38	1.9
4	1.95	0	23	1	0.15	2.07	7.96	2.33
3	1.94	0	24	1	0.22	1.17	7.88	2.33
4	1.95	0	25	1	0.27	1.19	8.83	2.33
4	1.95	0	25	1	0.27	0.1	8.88	1.45
3	2.1	0	16	1	0.17	1.04	10	5.44
3	1.82	0	17	1	0.11	0.84	10	5.44
5.1	2.15	0	13	1	1.38	0.22	10	2.26
3.9	2.12	0	14	1	1.83	0.43	10	2.26
5.1	2.15	0	13	1	1.83	0.83	11	2.8
3	1.5	0	9	1	0.28	1.98	10	2.26
5	2.5	0	10	1	0.2	1.59	10	2.26
8	4	0	11	1	0.48	0.43	10	2.26
8	4	0	12	1	1.18	0.31	10	2.26
9	4.5	0	13	1	1.08	0.22	10	2.26
12	6	0	14	1	0.54	0.43	10	2.26
5	2.5	0	10	1	0.2	1.59	10	2.26
8	4	0	11	1	0.48	0.43	10	2.26
8	4	0	12	1	1.18	0.31	10	2.26
9	4.5	0	13	1	1.08	0.22	10	2.26
12	6	0	14	1	0.54	0.43	10	2.26
2	1.7	0	17	1	0.34	3.26	11	2.8
7	1.61	0	20	1	0.69	1.51	11.5	2.8
8	1.73	0	21	1	0.73	0.79	11.5	2.8
21	2	0	22	1	0.66	0.74	11.04	2.8
12	1.81	0	23	1	0.82	0.83	11	2.8
17	2.06	0	24	1	0.81	0.71	11	2.8
7	1.61	0	20	1	0.69	0.57	8	0.84
8	1.73	0	21	1	0.73	0.54	8	0.84
21	2	0	22	1	0.66	0.64	8.08	0.84
12	1.81	0	23	1	0.82	0.61	8.5	0.84
17	2.06	0	24	1	0.81	0.44	8.5	0.84
21	2	0	22	1	0.66	2	8	2.65
12	1.81	0	23	1	0.82	1.61	8	2.65
17	2.06	0	24	1	0.81	1.58	8.38	2.65
17	2.06	0	24	1	0.81	0.59	10.46	5.44
12	3	0	20	1	0.43	0.79	11.5	2.8
13	3.25	0	21	1	0.34	0.74	11.04	2.8
17	4.25	0	22	1	0.52	0.83	11	2.8
20	5	0	23	1	0.6	0.71	11	2.8
1	1	0	10	1	1.96	1.96	7.5	1.9
1	1	0	11	1	1.61	1.33	7.5	1.9
3	1.5	0	12	1	2.14	1.35	8.08	1.9
3	1.5	0	13	1	2.43	1.29	8.67	1.9
3	1.5	0	14	1	3.35	1.04	8.38	1.9
3	1.5	0	15	0	3.62	0.85	8.04	1.9
3	1.5	0	16	0	3.01	1.22	8.67	1.9

1	1.45	0	19	0	0.29	2.07	7.96	2.33
2	1.94	0	20	1	0.27	1.17	7.88	2.33
2	1.95	0	21	1	0.22	1.19	8.83	2.33
1	1.95	0	17	0	1.11	0.56	7.71	1.45
1	1.92	0	18	0	1.69	0.44	9	1.45
1	1.93	0	19	1	1.83	0.23	8.29	1.45
1	1.92	0	20	1	1.29	0.1	8.88	1.45
4	1.33	0	17	1	0.44	0.56	7.71	1.45
4	1.33	0	18	1	0.55	0.44	9	1.45
4	1.33	0	19	1	0.72	0.23	8.29	1.45
5	1.25	0	20	1	0.73	0.1	8.88	1.45
4	1.62	0	19	1	0.29	1.96	7.5	1.9
6	1.66	0	20	1	0.35	1.33	7.5	1.9
9.1	1.74	0	21	1	0.38	1.35	8.08	1.9
11.2	1.8	0	22	1	0.43	1.29	8.67	1.9
11.9	1.73	0	23	1	0.36	1.04	8.38	1.9
9.1	1.78	0	24	1	0.37	0.85	8.04	1.9
4	1.62	0	19	1	0.29	3.29	11.46	2.8
6	1.66	0	20	1	0.35	3.23	11.5	2.8
9.1	1.74	0	21	1	0.38	1.51	11.5	2.8
11.2	1.8	0	22	1	0.43	0.79	11.5	2.8
11.9	1.73	0	23	1	0.36	0.74	11.04	2.8
9.1	1.78	0	24	1	0.37	0.83	11	2.8
3	1	1	19	1	0.42	1.38	9	3.8
2	1	0	22	0	0.77	1.92	8	1.79
4	2	0	23	1	0.83	1.59	8.46	1.79
2	1.72	0	19	1	0.43	2.32	7.71	2.33
2	2.05	0	20	1	0.68	2.07	7.96	2.33
6	1.5	1	33	0	0.31	0.9	10.38	4.65
3	1	1	16	0	2.28	0.59	8	5.83
3	1	1	17	0	2.25	1.81	7.13	5.83
3	1	1	32	0	1.22	1.81	7.13	5.83
3	1	1	32	0	1.22	2.21	8	3.23
7	1.4	0	12	1	0.97	6.22	6.67	4.43
14	1.4	0	18	1	0.74	3.84	7.75	4.43
12	1.2	0	19	1	0.63	2.64	8.5	4.43
14	1.4	0	18	1	0.74	1.92	8	1.79
12	1.2	0	19	1	0.63	1.59	8.46	1.79
14	1.4	0	18	1	0.74	0.85	8.04	1.9
12	1.2	0	19	1	0.63	1.22	8.67	1.9
7	1.75	0	16	0	0.31	0.74	11.04	2.8
1	1.69	0	25	0	0.89	0.1	8.88	1.45
3	1	0	19	1	1.31	1.2	8	5.5
7	1.75	0	20	1	0.92	1.2	8	5.5
7	1.75	0	20	1	0.92	2.64	8.5	4.43
3	1.5	0	20	1	1.99	0.31	10	2.26
4	1.33	0	21	1	2.61	0.22	10	2.26
3	1.5	0	22	1	2.94	0.43	10	2.26
2	1.8	0	20	1	0.23	0.74	11.04	2.8
2	1.8	0	21	1	0.26	0.83	11	2.8
2	1.8	0	20	1	0.23	2	8	2.65

2	1.8	0	21	1	0.26	1.61	8	2.65
3	1.83	0	22	1	0.84	0.57	8	0.84
5	1.95	0	23	1	0.44	0.54	8	0.84
7	1.97	0	24	1	0.61	0.64	8.08	0.84
7	1.97	0	25	1	0.73	0.61	8.5	0.84
9	1.97	0	26	1	0.82	0.44	8.5	0.84
10	1.25	0	17	0	0.39	0.59	7.42	1.45
12	1.5	0	18	0	0.46	0.56	7.71	1.45
17	1.89	0	21	0	0.51	0.1	8.88	1.45
67	9.57	0	15	1	0.89	0.71	11	2.8
2	2.08	0	21	1	2.21	1.04	8.38	1.9
3	2.19	0	22	1	2.34	0.85	8.04	1.9
3	2.18	0	23	1	2.36	1.22	8.67	1.9
2	1.47	0	14	0	0.49	2.44	6	3.31
2	1.43	0	15	0	0.28	2.87	6.5	3.31
12	1.73	0	16	1	1.04	1.92	6.33	3.31
10.4	1.75	0	17	1	1.18	2.27	6.38	3.31
2	1.47	0	14	0	0.49	1.35	8.08	1.9
2	1.43	0	15	0	0.28	1.29	8.67	1.9
12	1.73	0	16	1	1.04	1.04	8.38	1.9
10.4	1.75	0	17	1	1.18	0.85	8.04	1.9
14.7	1.69	0	18	1	1.28	1.22	8.67	1.9
12	1.73	0	16	1	1.04	1.7	8	3.2
10.4	1.75	0	17	1	1.18	1.38	7.71	3.2
14.7	1.69	0	18	1	1.28	1.31	8.25	3.2
3	1.5	0	28	0	1.54	0.21	9	4.17
5	1.25	1	14	0	16.12	0.71	11	2.8
2	1.96	0	23	1	0.27	0.66	10	5.44
2	1.97	0	24	1	0.26	0.59	10.46	5.44
2	1	1	23	1	1.42	0.44	9	1.45
2	1	1	24	1	1.55	0.23	8.29	1.45
2	1	1	25	1	1.38	0.1	8.88	1.45
2	1.91	0	16	1	0.47	0.44	8.5	0.84
6	1.2	1	20	1	3.68	1.22	8.67	1.9
5	1	0	14	1	0.23	0.56	7.71	1.45
6	1	0	15	1	0.29	0.44	9	1.45
6	1	0	16	1	0.43	0.23	8.29	1.45
6	1	0	17	1	0.58	0.1	8.88	1.45
6	1.96	0	24	1	0.57	1.05	9.29	3.97
5	1	0	14	1	0.23	1.29	8.67	1.9
6	1	0	15	1	0.29	1.04	8.38	1.9
6	1	0	16	1	0.43	0.85	8.04	1.9
6	1	0	17	1	0.58	1.22	8.67	1.9
5	1	0	14	1	0.23	0.54	8	0.84
6	1	0	15	1	0.29	0.64	8.08	0.84
6	1	0	16	1	0.43	0.61	8.5	0.84
6	1	0	17	1	0.58	0.44	8.5	0.84
6	1	0	15	1	0.29	2.07	7.96	2.33
6	1	0	16	1	0.43	1.17	7.88	2.33
6	1	0	17	1	0.58	1.19	8.83	2.33
6	1.96	0	24	1	0.57	1.22	8.67	1.9

8	1.6	0	25	1	0.98	0.61	8.5	0.84
9	1.8	0	26	1	1.1	0.44	8.5	0.84
8	1.91	0	10	1	1.16	0.83	11	2.8
1	1	0	8	0	0.37	6.96	7.5	4.65
3	1	0	10	1	0.93	5.8	7.5	4.65
3	1	0	11	1	1.55	6.11	7	4.65
6	1.5	0	16	1	0.79	0.64	8.08	0.84
6	1.5	0	16	1	0.79	1.04	8.38	1.9
2	1.6	1	16	0	0.87	1.29	8.67	1.9
2	1.69	1	17	1	1.05	1.04	8.38	1.9
3	1.79	1	18	1	0.93	0.85	8.04	1.9
2	1.69	1	19	1	0.58	1.22	8.67	1.9
2	1.6	1	16	0	0.87	1.82	7.5	5.31
2	1.69	1	17	1	1.05	2.57	7.5	5.31
3	1.79	1	18	1	0.93	2.72	7.5	5.31
2	1.69	1	19	1	0.58	2.83	7.5	5.31
5	1.87	0	17	0	1.04	1.22	8.67	1.9
5	1.87	0	17	0	1.04	1.19	8.83	2.33
3	1.45	0	11	1	0.65	0.31	10	2.26
3	1.45	0	12	1	1.15	0.22	10	2.26
2	1.36	0	13	1	1.13	0.43	10	2.26
3	1.45	0	11	1	0.65	0.64	8.08	0.84
3	1.45	0	12	1	1.15	0.61	8.5	0.84
2	1.36	0	13	1	1.13	0.44	8.5	0.84
3	1.45	0	11	1	0.65	0.74	11.04	2.8
3	1.45	0	12	1	1.15	0.83	11	2.8
5	2.5	0	19	1	6.59	0.43	10	2.26
5	2.5	0	18	1	0.89	0.21	9	4.17
2	1.51	0	15	1	0.14	1.61	8	2.65
1	1.41	0	16	1	0.28	1.58	8.38	2.65
2	1.71	1	32	1	0.67	0.74	11.04	2.8
5.1	1.73	1	34	1	0.51	0.71	11	2.8
5.2	1.72	0	9	0	0.21	1.04	10.46	4.44
2	1	1	20	0	3.38	3.84	7.75	4.43
2	1	1	21	0	3.49	2.64	8.5	4.43
2	1	1	20	0	3.38	0.59	7	2.96
2	1	1	21	0	3.49	0.55	7.13	2.96
14	1.27	0	13	1	3.24	2.55	6.5	1.79
16	1.23	0	14	1	3.02	1.99	7.33	1.79
18	1.38	0	15	1	3.15	1.92	8	1.79
18	1.38	0	16	1	3.37	1.59	8.46	1.79
10	1.43	0	14	1	0.13	1.98	10	2.26
15	1.67	0	15	1	0.19	1.59	10	2.27
13	1.86	0	16	1	0.4	0.43	10	2.28
14	2	0	17	1	0.35	0.31	10	2.27
4	1.33	0	13	1	0.15	0.49	7	0.84
10	1.43	0	14	1	0.15	0.49	7.17	0.84
15	1.67	0	15	1	0.19	0.57	8	0.85
13	1.86	0	16	1	0.4	0.54	8	0.86
14	2	0	17	1	0.35	0.64	8.08	0.84
8	1.33	0	15	1	0.39	0.64	8.08	0.84

5	1	0	16	1	0.47	0.61	8.5	0.84
3	1	0	23	1	1.36	0.44	8.5	0.84
32	4	0	17	1	0.74	1.75	8	2.86
19	1.58	0	18	1	0.29	2	8	2.65
20	1.67	0	19	1	0.38	1.61	8	2.65
21	1.75	0	20	1	0.2	1.58	8.38	2.65
5	1	1	18	1	0.14	1.04	8.38	1.9
6	1.2	1	19	1	0.14	0.85	8.04	1.9
7	1	1	20	1	0.2	1.22	8.67	1.9
29	1.93	0	11	1	0.82	1.33	7.5	1.9
30	2	0	12	1	0.72	1.35	8.08	1.9
39	2.6	0	13	1	1.11	1.29	8.67	1.9
53	3.31	0	14	1	0.99	1.04	8.38	1.9
56	3.29	0	15	1	1.04	0.85	8.04	1.9
12	2.4	0	20	0	0.52	0.71	11	2.8
2	1	0	14	1	0.22	3.31	11.5	2.8
2	1	0	15	1	0.29	3.25	11.5	2.8
2	1	0	16	1	0.3	3.26	11	2.8
2	1	0	17	1	0.28	3.29	11.46	2.8
2	1	0	18	1	0.32	3.23	11.5	2.8
2	1	0	19	1	0.48	1.51	11.5	2.8
2	1	0	20	1	0.35	0.79	11.5	2.8
2	1	0	21	1	0.45	0.74	11.04	2.8
2	1	0	22	1	0.46	0.83	11	2.8
2	1	0	23	0	0.44	0.71	11	2.8
2		0	17	1	0.56	1.42	9.5	4.65
1	1.85	0	22	1	0.6	0.44	8.5	0.84
4	1	0	20	1	0.95	0.71	11	2.8
6	1.2	0	12	1	0.58	1.98	10	2.26
6	1.2	0	13	1	0.22	1.59	10	2.26
6	1.2	0	14	1	0.14	0.43	10	2.26
6	1.2	0	15	1	0.47	0.31	10	2.26
6	1.5	0	16	1	1.68	1.04	8.38	1.9
6	1.5	0	17	1	1.66	0.85	8.04	1.9
6	2.06	0	21	1	0.78	1.04	8.38	1.9
7	2.1	0	22	1	1.06	0.85	8.04	1.9
6	2	0	23	1	1.06	1.22	8.67	1.9
2	1.85	1	22	1	1.56	0.83	11	2.8
1	2.05	1	11	1	0.92	0.79	11.5	2.8
1	2.07	1	12	1	0.71	0.74	11.04	2.8
1	2.05	1	13	1	0.91	0.83	11	2.8
1	2.06	1	14	1	0.42	0.71	11	2.8
2	1.72	0	13	1	0.06	1.51	11.5	2.8
3.9	1.96	0	14	1	0.07	0.79	11.5	2.8
2	2.03	0	15	1	0.52	1.59	10	2.26
2	2.06	0	16	1	1	0.43	10	2.26
2	2.03	0	17	1	1.22	0.31	10	2.26
2	2.02	0	18	1	1.2	0.22	10	2.26
2	2.02	0	19	1	1.21	0.43	10	2.26
5	1.96	0	16	0	1.2	0.74	11.04	2.8
5	1.98	0	17	0	1.4	1.92	8	1.79

5	1.97	0	18	1	1.97	1.59	8.46	1.79
2	1.49	0	13	1	0.41	1.35	8.08	1.9
2	1.91	0	14	1	0.63	1.29	8.67	1.9
2	1.94	0	15	1	0.61	1.04	8.38	1.9
4	2.02	0	16	1	0.61	0.85	8.04	1.9
6	2.05	0	17	1	0.69	1.22	8.67	1.9
5	1.25	1	14	1	0.87	0.31	10	2.26
3	1.73	0	9	1	0.56	1.98	10	2.26
3	1.73	0	10	1	0.4	1.59	10	2.26
4	1.85	0	12	0	0.93	0.31	10	2.26
4	1.85	0	13	0	0.95	0.22	10	2.26
5	2.07	0	14	1	0.77	0.43	10	2.26
5	1.67	0	19	0	0.93	0.85	8.04	1.9
8	2	0	20	0	1.58	1.22	8.67	1.9
5	1.67	0	26	0	0.12	1.19	8.83	2.33
1	1.85	0	21	0	0.16	1.22	8.67	1.9
1	2	0	16	1	0.21	0.54	8	0.84
17	2.43	0	13	1	0.85	7	7.79	4.65
18	2.57	0	14	1	0.59	5.8	7.5	4.65
15	2.14	0	15	1	0.89	6.11	7	4.65
7	2.33	0	20	1	0.75	0.74	11.04	2.8
7	2.33	0	21	1	0.73	0.83	11	2.8
7	2.33	0	22	1	1.01	0.71	11	2.8
1	1.38	0	12	1	0.09	3.29	11.46	2.8
2	1.38	0	16	1	0.28	0.74	11.04	2.8
2	1.39	0	17	1	0.3	0.83	11	2.8
4	1.37	0	18	1	0.37	0.71	11	2.8
2	1.68	0	21	1	0.58	1.96	7.5	1.9
2	1.75	0	22	1	0.57	1.33	7.5	1.9
2	1.87	0	23	1	0.39	1.35	8.08	1.9
2	1.87	0	24	1	0.41	1.29	8.67	1.9
2	1.86	0	25	1	0.43	1.04	8.38	1.9
3	1.86	0	26	1	0.52	0.85	8.04	1.9
3	1.84	0	27	1	0.62	1.22	8.67	1.9
2	1.85	0	11	0	0.24	2.26	8.46	5.28
6	1.91	0	12	0	0.34	2.48	9.29	5.28
6	1.95	0	13	1	0.37	1.98	10.5	5.28
6	1.93	0	14	1	0.64	1.18	10.5	5.28
4	2	0	14	1	0.59	2.32	7.71	2.33
4	1.33	0	16	1	0.96	1.17	7.88	2.33
4	1.33	0	17	1	1.05	1.19	8.83	2.33
2	1.52	0	18	1	0.1	3.26	11	2.8
2	1.5	0	19	1	0.12	3.29	11.46	2.8
3	1.99	0	21	1	0.51	1.51	11.5	2.8
5	2	0	22	1	0.42	0.79	11.5	2.8
4	1.98	0	23	0	0.35	0.74	11.04	2.8
4	1.99	0	24	1	0.58	0.83	11	2.8
4	2.07	0	25	1	0.84	0.71	11	2.8
5	2	0	22	1	0.42	1.37	9	3.8
4	1.98	0	23	0	0.35	1.38	9	3.7
4	1.99	0	24	1	0.58	1.44	9	3.9

4	2.07	0	25	1	0.84	1.01	10.04	4.01
4	1.65	0	22	0	0.2	1.29	8.67	1.9
6	1.65	0	23	0	0.14	1.04	8.38	1.9
4	1.63	0	24	0	0.15	0.85	8.04	1.9
5	1.51	0	25	0	0.15	1.22	8.67	1.9
2	1.79	0	20	1	2.59	1.29	8.67	1.9
2	1.79	0	21	1	3.43	1.04	8.38	1.9
2	1.78	0	22	1	2.48	0.85	8.04	1.9
2	1.95	0	23	1	3.05	1.22	8.67	1.9
4	1.68	1	21	1	1.41	0.23	8.29	1.45
1	1.34	0	16	1	0.09	1.51	11.5	2.8
4	1.68	1	21	1	1.41	0.85	8.04	1.9
1	1.74	0	15	0	0.33	0.79	11.5	2.8
4	1.84	0	17	0	0.74	0.83	11	2.8
2	1	0	18	0	0.28	0.74	11.04	2.8
1	1.44	0	12	1	0.76	0.43	10	2.26
1	1.56	0	13	1	0.5	0.31	10	2.26
13	1.63	0	14	1	0.59	1.39	9.5	4.65
28	2.33	0	15	1	0.58	1.37	9.08	4.65
30	2.5	0	16	1	0.52	1.35	9.42	4.65
34	2.83	0	17	1	0.45	1.42	9.5	4.65
33	3	0	18	1	0.41	0.9	10.38	4.65
3	1.93	0	16	1	1.49	0.71	11	2.8
28	2.33	0	15	1	0.58	2.32	8.86	2.33
30	2.5	0	16	1	0.52	2.07	8.86	2.32
34	2.83	0	17	1	0.45	1.17	7.88	2.31
33	3	0	18	1	0.41	1.19	7.71	2.3
8	2.67	0	27	0	1.04	0.44	8.5	0.84
2	1	0	18	1	1.62	0.22	10	2.26
4	1.77	0	15	1	0.83	1.29	8.67	1.9
4	1.91	0	16	1	1.45	1.04	8.38	1.9
6.5	1.98	0	17	1	1.08	0.85	8.04	1.9
6	2.03	0	18	1	1.17	1.22	8.67	1.9
4	1.77	0	15	1	0.83	2.32	7.71	2.33
4	1.91	0	16	1	1.45	2.07	7.96	2.53
6.5	1.98	0	17	1	1.08	1.17	7.88	2.5
6	2.03	0	18	1	1.17	1.19	8.83	2.51
14	1.75	0	18	1	1.27	1.04	10.46	4.44
5	1.25	0	16	1	0.55	2.17	9.5	2.48
7	1	0	19	1	1.07	2.57	7.5	5.31
7	1	0	20	0	1.32	2.72	7.5	5.31
8	1	0	21	0	1.5	2.83	7.5	5.31
1	1.74	0	15	1	1.17	1.29	8.67	1.9
1	1.87	0	16	1	1.17	1.04	8.38	1.9
1	1.89	0	17	1	1.26	0.85	8.04	1.9
1	1.85	0	18	1	1.3	1.22	8.67	1.9
1	1.93	0	14	1	0.09	0.14	9	4.17
1	1.77	0	15	0	0.06	0.2	9	4.17
16.5	1.86	0	12	1	1.39	0.22	10	2.26
18.7	1.9	0	13	1	1.64	0.43	10	2.26
6	1.76	0	9	1	1.4	1.51	11.5	2.8

8	1.77	0	10	1	0.96	0.79	11.4	2.6
9	1.8	0	11	1	1.33	0.74	11.42	2.79
8	1.77	0	10	1	0.96	1.29	8.04	1.9
9	1.8	0	11	1	1.33	1.04	8.67	2.9
16.5	1.86	0	12	1	1.39	0.85	8.67	2.9
18.7	1.9	0	13	1	1.64	1.22	8.77	2.94
1	1.64	0	17	1	0.82	3.49	7.88	2.33
31	2.38	0	15	1	1.9	1.08	8.71	3.2
31	2.07	0	16	1	1.87	1.7	8.25	3.19
24	1.71	0	17	1	1.82	1.38	8.25	3.18
23	1.64	0	18	1	1.7	1.31	8	3.17
1	1.82	0	20	0	0.62	0.85	8.04	1.9
11.2	1.4	0	21	0	1.21	1.86	9	3.8
11.2	1.83	0	22	1	0.84	1.37	9	3.8
14.4	1.84	0	23	1	1.25	1.38	9	3.8
14.4	1.9	0	24	1	0.88	1.44	9	3.8
12.8	1.91	0	25	1	0.83	1.01	10.04	3.8
11.2	1.4	0	21	0	1.21	1.51	11.5	2.8
11.2	1.83	0	22	1	0.84	0.79	11.4	2.6
14.4	1.84	0	23	1	1.25	0.74	11.74	2.9
14.4	1.9	0	24	1	0.88	0.83	11.7	2.7
12.8	1.91	0	25	1	0.83	0.71	11.7	2.7
1	1.95	0	14	1	0.08	1.35	8.08	1.9
2	2	0	15	1	0.15	1.29	8.67	1.9
2	2	0	16	1	0.2	1.04	8.38	1.9
5	1.99	0	18	1	0.24	1.22	8.67	1.9
1	1	0	9	1	0.07	4.72	5.5	4.65
3	1.5	0	11	1	1.22	0.99	8	5.83
6	1.5	0	12	1	2.18	0.76	8	5.83
5	2.5	0	10	1	0.26	1.98	10	2.26
7	2.33	0	11	1	0.25	1.98	10	2.26
8	2.67	0	12	1	0.23	1.59	10	2.26
11	2.75	0	13	1	0.23	0.43	10	2.26
13	3.25	0	14	1	0.15	0.31	10	2.26
12	3	0	15	1	0.14	0.22	10	2.26
12	3	0	16	0	0.08	0.43	10	2.26
1	1.21	0	12	1	0.65	0.74	11.04	2.8
1	1.94	0	13	1	0.52	0.83	11	2.8
1	2	0	14	1	0.69	0.71	11	2.8
11	2.75	0	13	1	0.23	0.43	10	2.26
13	3.25	0	14	1	0.15	0.31	10	2.16
12	3	0	15	1	0.14	0.22	10	2.14
12	3	0	16	0	0.08	0.43	10	2.06
2	1.78	0	12	1	1.48	1.59	10	2.26
2	1.74	0	13	1	2.91	0.43	10	2.26
3	1.53	0	15	1	2.04	0.22	10	2.26
3.9	1.43	0	16	1	1.68	0.43	10	2.26
2	1.44	1	14	1	1.18	0.22	10	2.26
2	1.78	0	11	0	0.44	0.79	11.5	2.8
2	1.65	0	12	0	0.46	0.74	11.04	2.8
2	1.77	0	13	1	0.37	0.83	11	2.8

3	1.83	0	14	1	0.64	0.71	11	2.8
13	1.44	0	12	1	0.54	1.59	10	2.26
13	1.3	0	13	1	0.34	0.43	10	2.26
15	1.36	0	14	1	0.31	0.31	10	2.26
22	1.69	0	15	1	0.47	0.22	10	2.26
32	2.46	0	16	1	0.48	0.43	10	2.26
22	1.69	0	15	1	0.47	1.38	7.71	3.2
32	2.46	0	16	1	0.48	1.31	8.25	3.21
7	1.75	0	19	1	0.19	0.83	11	2.8
7	2.33	0	20	1	0.27	0.71	11	2.8
1	1.66	0	23	0	0.5	0.23	9	4.17
1	1.71	0	24	0	0.58	0.21	9	4.17
4	1.61	0	15	1	0.26	3.23	11.5	2.8
4	1.6	0	16	1	0.26	1.51	11.5	2.8
3	1.72	0	20	1	0.21	0.71	11	2.8
2	1.68	0	12	1	0.45	0.31	10	2.26
2	1.69	0	14	1	0.35	0.43	10	2.26
2	1.42	0	15	1	0.37	0.57	8	0.84
3	1.44	0	16	1	0.52	0.54	8	0.84
2	1.55	0	17	1	0.61	0.64	8.08	0.84
2	1.79	0	18	0	0.83	0.61	8.5	0.84
6	1.6	0	15	1	0.58	0.22	10	2.26
6	1.67	0	16	1	0.97	0.43	10	2.26
1	1.41	0	21	0	0.22	1.04	8.38	1.9
1	1.81	0	22	0	0.29	0.85	8.04	1.9
1	1.74	0	18	0	0.2	1.19	8.83	2.33
3	1.9	0	13	0	1.13	1.29	8.67	1.9
3.9	1.92	0	14	0	1.19	1.04	8.38	1.9
5.2	1.92	0	15	0	1.3	0.85	8.04	1.9
7.2	1.93	0	16	0	1.22	1.22	8.67	1.9
14	1.75	0	8	1	0.45	1.51	11.5	2.8
14	1.75	0	9	1	0.99	0.79	11.5	2.8
15	1.88	0	10	1	0.37	0.74	11.04	2.8
17	2.13	0	11	1	0.68	0.83	11	2.8
19	2.38	0	12	1	1.19	0.71	11	2.8
4	1.33	1	19	1	2.26	0.44	9	1.45
4	1.33	1	20	1	3.14	0.23	8.29	1.45
4	1	1	21	1	4.01	0.1	8.88	1.45
4	1	1	21	1	4.01	1.22	8.67	1.9
3.9	1.86	0	14	1	1.26	1.71	9.5	2.48
15	1.85	0	15	1	1.43	1.47	9.5	2.48
15	1.85	0	15	1	1.43	0.85	9.58	2.95
3.9	1.84	0	21	1	1.26	1.17	7.88	2.33
4	1.86	0	22	1	0.78	1.19	8.83	2.33
1	1.78	0	22	1	0.15	0.83	11	2.8
2	1.74	0	12	0	1.13	0.64	8.08	0.84
2	1.73	0	13	1	1.53	0.61	8.5	0.84
2	1.76	0	14	1	1.1	0.44	8.5	0.84
1	1.43	0	15	1	0.66	1.51	11.5	2.8
1	1.43	0	16	1	1	0.79	11.5	2.8
1	1.77	0	17	1	0.82	0.74	11.04	2.8

1	1.79	0	18	1	1.2	0.83	11	2.8
1	1.76	0	19	1	1.24	0.71	11	2.8
3	1.9	0	14	1	0.59	0.79	11.5	2.8
3	1.91	0	15	1	0.85	0.74	11.04	2.8
3	1.93	0	16	1	1.24	0.83	11	2.8
2	1.95	0	17	1	1.76	0.71	11	2.8
4	1.72	0	20	1	0.57	0.1	8.88	1.45
20	2	0	12	1	0.9	0.23	8.29	1.45
3	1	0	13	1	1.18	0.1	8.88	1.45
7	1.4	0	25	1	4.67	0.83	11	2.8
13	2.6	0	26	1	0.88	0.71	11	2.8
1	1.77	0	17	1	0.09	1.19	8.83	2.33
3	1.82	0	8	1	0.23	1.51	11.5	2.8
3.9	1.85	0	9	1	0.72	0.79	11.5	2.8
6	1.77	0	10	1	1.08	0.74	11.04	2.8
9	1.76	0	11	1	0.7	0.83	11	2.8
11.2	1.75	0	12	1	0.45	0.71	11	2.8
3	1.79	0	25	0	0.18	1.35	9.42	4.65
3	1.78	0	26	0	0.2	1.42	9.5	4.65
3	1.79	0	27	1	0.2	0.9	10.38	4.65
2	1	0	20	1	0.5	0.23	8.29	1.45
2	1	0	21	1	0.37	0.1	8.88	1.45
1	1.53	0	17	0	0.96	0.79	11.5	2.8
1	1.53	0	18	0	1.24	0.74	11.04	2.8
1	1.61	0	19	0	0.63	0.83	11	2.8
1	1.63	0	20	0	0.71	0.71	11	2.8
1	1.63	0	17	1	0.96	0.83	11	2.8
1	1.63	0	18	0	1.02	0.71	11	2.8
1	1.48	0	16	0	0.12	1.22	8.67	1.9
2	1.8	0	13	0	1.22	0.22	10	2.26
3	1.93	0	14	0	1.91	0.43	10	2.26
3	1.59	0	16	1	1.33	0.83	11	2.8
3	1.63	0	17	1	1.16	0.71	11	2.8
2	1	0	8	1	0.5	1.29	8.67	1.9
2	1	0	9	1	1.01	1.04	8.38	1.9
2	1	0	10	1	1.15	0.85	8.04	1.9
13	2.6	0	11	1	1.28	1.22	8.67	1.9
3	1.36	0	16	1	0.86	0.83	11	2.8
1	1.91	0	14	1	0.32	0.71	11	2.8
1	1.77	0	11	1	0.44	0.83	11	2.8
2	1	0	13	1	0.59	0.44	8.5	0.84
2	1.93	0	17	1	0.28	0.71	11	2.8
3	1.73	0	15	0	0.47	2.2	9.67	4.44
3	1.77	0	16	1	0.59	1.25	10.29	4.44
2	1.67	0	17	1	0.58	1.04	10.46	4.44
1	1.85	0	14	1	1.2	2.85	7.5	3.1
1	1.84	0	15	1	1.06	2.86	7.88	3.1
7	1.4	0	13	1	0.44	1.04	8.38	1.9
11	1.83	0	14	1	0.75	0.85	8.04	1.9
15	1.88	0	15	1	0.81	1.22	8.67	1.9
1	1.69	0	20	1	0.93	0.22	10	2.26

1	1.68	0	21	1	0.96	0.43	10	2.26
1	1.62	0	14	1	1.35	0.83	11	2.8
1	1.62	0	15	1	1.14	0.71	11	2.8
2	1.88	0	12	1	0.67	0.71	11	2.8
6	2	0	17	1	0.21	0.61	8.5	0.84
11	3.67	0	18	1	0.32	0.44	8.5	0.84
6	2	0	17	1	0.21	0.23	9	4.17
11	3.67	0	18	1	0.32	0.21	9	4.17
12	2	0	8	1	0.21	0.74	11.04	2.8
12	2	0	9	1	0.24	0.83	11	2.8
16	1.6	0	10	1	0.35	0.71	11	2.8
16	1.6	0	10	1	0.35	1.19	8.83	2.33
6	1.5	0	19	0	0.57	0.44	8.5	0.84
6	2	0	26	0	1.38	0.44	8.5	0.84
3	1.5	0	18	0	1.16	0.44	8.5	0.84
6	1.2	0	16	1	0.6	0.64	8.08	0.84
7	1.4	0	17	1	0.58	0.61	8.5	0.84
7	1.4	0	18	1	0.89	0.44	8.5	0.84
1	1.78	0	16	1	1.37	0.1	8.88	1.45
8.8	1.32	0	17	0	0.25	0.85	8.04	1.9
8.8	1.33	0	18	0	0.33	1.22	8.67	1.9
4	1	0	11	1	0.87	0.83	11	2.8
4	1	0	12	1	1.12	0.71	11	2.8
16	2	0	8	1	1.17	0.83	11	2.8
11.2	2.12	0	9	1	1.55	0.71	11	2.8
1	1.72	0	13	1	1.34	1.22	8.67	1.9
15	1.54	0	21	1	0.38	1.22	8.67	1.9
1	1	1	27	1	0.59	0.71	11	2.8
2	1	1	15	1	1.64	0.65	7.54	1.45
2	1	1	16	1	1.74	0.64	7.33	1.45
2	1	1	17	1	1.6	0.61	7.5	1.45
13	1.63	1	18	1	1.64	3.23	11.5	2.8
12	1.5	1	19	1	2.33	1.51	11.5	2.8
13	1.63	1	18	1	1.64	1.33	7.5	1.9
12	1.5	1	19	1	2.33	1.35	8.08	2.9
13	1.63	1	18	1	1.64	1.98	10	2.26
12	1.5	1	19	1	2.33	1.59	10	3.26
15	5	1	25	1	3.62	0.59	8	5.83
15	5	1	26	1	3.01	1.81	7.13	5.83
14	2.33	1	28	1	0.53	1.34	10	5.44
33	4.71	1	29	1	0.8	1.04	10	5.44
33	4.13	1	30	1	0.92	0.84	10	5.44
33	4.13	1	31	1	0.82	0.66	10	5.44
34	4.25	1	32	1	0.63	0.59	10.46	5.44
8	1.14	1	11	1	1	3.31	11.5	2.8
10	1.11	1	12	1	1.1	3.25	11.5	2.8
12	1.09	1	13	1	1.1	3.26	11	2.8
11	1.1	1	14	1	0.96	3.29	11.46	2.8
9	1.13	1	15	1	0.91	3.23	11.5	2.8
8	1.14	1	16	1	0.99	1.51	11.5	2.8
9	1.13	1	17	1	1.13	0.79	11.5	2.8

10	1.25	1	18	1	1.07	0.74	11.04	2.8
10	1.25	1	19	1	0.83	0.83	11	2.8
9	1.29	1	20	1	0.83	0.71	11	2.8
14	2.33	1	28	1	0.53	1.51	11.5	2.8
33	4.71	1	29	1	0.8	0.79	11.5	2.9
33	4.13	1	30	1	0.92	0.74	11.04	3
33	4.13	1	31	1	0.82	0.83	11	2.9
34	4.25	1	32	1	0.63	0.71	11	2.8
10	1.11	1	12	1	1.1	0.69	7.17	0.84
12	1.09	1	13	1	0.87	0.71	7	0.82
11	1.1	1	14	1	0.96	0.49	7	0.83
9	1.13	1	15	1	0.91	0.49	7.17	0.83
9	1.13	1	17	1	1.13	0.54	8	0.85
10	1.25	1	18	1	1.07	0.64	8.08	0.84
10	1.25	1	19	1	0.83	0.61	8.5	0.83
12	1.09	1	13	1	1.1	3.51	8.5	2.33
11	1.1	1	14	1	0.96	3.41	8.5	2.13
9	1.13	1	15	1	0.91	3.49	7.88	2.12
8	1.14	1	16	1	0.99	2.97	7.5	2.14
9	1.13	1	17	1	1.13	2.32	7.71	2.35
10	1.25	1	18	1	1.07	2.07	7.96	2.34
10	1.25	1	19	1	0.83	1.17	7.88	2.14
9	1.29	1	20	1	0.83	1.19	8.83	2.14
12	1.09	1	13	1	1.1	2.22	10	2.26
11	1.1	1	14	1	0.96	1.98	10	2.26
9	1.13	1	15	1	0.91	1.98	10	2.26
8	1.14	1	16	1	0.99	1.59	10	2.26
9	1.13	1	17	1	1.13	0.43	10	2.26
10	1.25	1	18	1	1.07	0.31	10	2.26
10	1.25	1	19	1	0.83	0.22	10	2.26
9	1.29	1	20	1	0.83	0.43	10	2.26
4	1	1	16	1	2.98	3.29	11.46	2.8
11	1.57	1	17	1	3.38	3.23	11.5	2.8
17	2.43	1	18	1	3.53	1.51	11.5	2.8
20	2.22	1	19	1	3.86	0.79	11.5	2.8
25	2.78	1	20	1	4.84	0.74	11.04	2.8
24	3	1	21	1	4.55	0.83	11	2.8
22	3.14	1	22	1	4.25	0.71	11	2.8
4	1	1	17	1	3.22	0.99	8	2.86
4	1	1	18	0	2.77	1	8	2.86
4	1	1	19	0	2.01	1.75	8	2.86
11	1.57	1	17	1	3.38	5.55	6.04	3.31
17	2.43	1	18	1	3.53	2.44	6	3.31
20	2.22	1	19	1	3.86	2.87	6.5	3.31
25	2.78	1	20	1	4.84	1.92	6.33	3.31
24	3	1	21	1	4.55	2.27	6.38	3.31
22	3.14	1	22	1	4.25	2.41	7.5	3.31
4	1	1	18	0	2.77	0.23	9	4.17
4	1	1	19	0	2.01	0.21	9	4.17
6	1	1	18	1	1.02	2	8	2.65
6	1	1	19	1	1.02	1.61	8	2.65

4	1	1	17	1	3.22	0.2	9	4.17
41	5.86	1	20	1	3.82	2.58	8	3.4
42	6	1	21	1	3.41	2.33	7.92	3.4
46	5.75	1	22	1	3.17	2.31	7.17	3.4
41	5.86	1	23	1	3.15	2.1	7	3.4
42	4.67	1	24	1	3.24	0.79	7	3.4
43	4.78	1	25	1	3.87	1.07	7.04	3.4
11	1	0	17	1	1.71	2.03	8.5	1.9
11	1.1	0	18	1	1.55	2.12	8.5	1.9
16	1.45	0	19	1	1.73	2.06	7.71	1.9
16	1.45	0	20	1	1.63	1.96	7.5	1.9
16	1.33	0	21	1	1.61	1.33	7.5	1.9
15	1.36	0	22	1	1.66	1.35	8.08	1.9
10	1.11	0	23	1	1.68	1.29	8.67	1.9
10	1.11	0	24	1	1.25	1.04	8.38	1.9
10	1.11	0	25	1	1.31	0.85	8.04	1.9
6	1.2	0	26	1	1.01	1.22	8.67	1.9
46	5.75	1	22	1	3.17	0.43	10	2.26
41	5.86	1	23	1	3.15	0.31	10	2.26
42	4.67	1	24	1	3.24	0.22	10	2.26
43	4.78	1	25	1	3.87	0.43	10	2.26
43	4.78	1	25	1	3.87	1.81	7.13	5.83
20	2.22	1	19	1	3.86	2.41	8	5.5
25	2.78	1	20	1	4.84	2.63	8	5.5
24	3	1	21	1	4.55	1.2	8	5.5
22	3.14	1	22	1	4.25	1.2	8	5.5
5	1.25	1	21	1	5.15	0.78	7.17	2.52
7	1.4	1	10	1	3.32	3.31	11.5	2.8
7	1.4	1	11	1	3.52	3.25	11.5	2.8
7	1.4	1	12	1	4.23	3.26	11	2.8
8	1.33	1	13	1	4.14	3.29	11.46	2.8
13	1.44	1	14	1	4.1	3.23	11.5	2.8
13	1.44	1	15	1	4.85	1.51	11.5	2.8
14	1.27	1	16	1	4.97	0.79	11.5	2.8
17	1.21	1	17	1	1.97	0.74	11.04	2.8
19	1.27	1	18	1	1.6	0.83	11	2.8
19	1.27	1	19	1	1.68	0.71	11	2.8
2	1	0	17	1	2.07	5.31	7.5	3.31
2	1	0	18	0	1.98	5.74	7.5	3.31
2	1	0	19	0	2.39	5.62	7.58	3.31
2	1	0	20	0	3.29	5.88	7.25	3.31
8	1.33	1	13	1	4.14	1.91	6	2.86
13	1.44	1	14	1	4.1	1.74	5.71	2.86
13	1.44	1	15	1	4.85	1.37	6.58	2.86
14	1.27	1	16	1	4.97	1.16	7.58	2.86
17	1.21	1	17	1	1.97	0.99	8	2.86
19	1.27	1	18	1	1.6	1	8	2.86
19	1.27	1	19	1	1.68	1.75	8	2.86
14	1.27	1	16	1	4.97	2.39	8	2.65
17	1.21	1	17	1	1.97	2	8	2.65
19	1.27	1	18	1	1.6	1.61	8	2.65

19	1.27	1	19	1	1.68	1.58	8.38	2.65
17	1.21	1	17	1	1.97	2.07	7.96	2.33
19	1.27	1	18	1	1.6	1.17	7.88	2.33
19	1.27	1	19	1	1.68	1.19	8.83	2.33
17	1.21	1	17	1	1.97	0.31	10	2.26
19	1.27	1	18	1	1.6	0.22	10	2.26
19	1.27	1	19	1	1.68	0.43	10	2.26
19	1.27	1	18	1	1.6	0.23	9	4.17
19	1.27	1	19	1	1.68	0.21	9	4.17
21	4.2	1	23	0	3.23	0.1	8.88	1.45
19	1.27	1	18	1	1.6	0.61	8.5	0.84
19	1.27	1	19	1	1.68	0.44	8.5	0.84
10	1.67	0	22	1	1	0.83	11	2.8
12	1.5	0	23	1	0.89	0.71	11	2.8
12	1.5	0	23	1	0.89	0.94	8.08	5.48
5	1	0	20	1	1.58	2.39	8	2.65
7	1	0	21	1	1.33	2	8	2.65
9	1	0	22	0	1.2	1.61	8	2.65
9	1	0	23	0	1.08	1.58	8.38	2.65
2	1	1	20	0	2.66	0.99	8	5.83
3	1	1	21	0	3.31	0.76	8	5.83
2	1	1	22	0	3.81	0.59	8	5.83
1	1	1	20	0	1.2	0.79	11.5	2.8
1	1	1	21	0	1.06	0.74	11.04	2.8
1	1	1	14	1	1.73	4.37	11.5	5.28
2	1	1	15	1	1.26	4.06	11.38	5.28
2	1	1	16	1	1.7	3.55	8.96	5.28
2	1	1	17	1	1.67	2.81	8	5.28
2	1	1	18	1	1.1	2.81	8	5.28
2	1	1	19	1	1.38	2.25	8	5.28
2	1	1	20	0	2.01	2.26	8.46	5.28
15	1.88	0	24	1	1.44	0.6	8	5.07
32	1.78	0	25	1	1.39	1.22	8	5.07
35	1.84	0	26	1	1.95	1.25	8	5.07
33	1.83	0	27	1	2.07	1.13	8.08	5.07
1	1	1	18	0	2.57	1.51	11.5	2.8
1	1	1	19	0	2.67	0.79	11.5	2.8
1	1	1	20	0	2.9	0.74	11.04	2.8
1	1	1	21	0	3.08	0.83	11	2.8
1	1	1	22	0	3	0.71	11	2.8
7	1	1	14	0	0.39	2.16	10	2.26
6	1	1	15	0	0.72	2.18	10	2.26
6	1	1	16	0	1.02	2.22	10	2.26
5	1	1	17	1	0.81	1.98	10	2.26
7	1.17	1	18	1	0.78	1.98	10	2.26
9	1.5	1	19	1	0.49	1.59	10	2.26
9	1.5	1	20	0	0.64	0.43	10	2.26
8	1.33	1	21	0	0.66	0.31	10	2.26
8	1.33	1	22	1	0.64	0.22	10	2.26
7	1	1	14	0	0.39	4.66	9	4.17
6	1	1	15	0	0.72	3.83	9	4.17

6	1	1	16	0	1.02	3.34	9	4.17
5	1	1	17	1	0.81	2.82	9	4.17
7	1.17	1	18	1	0.78	2.86	9	4.17
9	1.5	1	19	1	0.49	0.51	9	4.17
9	1.5	1	20	0	0.64	0.14	9	4.17
8	1.33	1	21	0	0.66	0.2	9	4.17
8	1.33	1	22	1	0.64	0.23	9	4.17
8	1.6	1	23	1	0.68	0.21	9	4.17
8	1.6	1	23	1	0.68	0.71	11	2.8
7	1	1	14	0	0.39	2.91	7.5	2.33
6	1	1	15	0	0.72	3.15	8	2.33
6	1	1	16	0	1.02	3.51	8.5	2.33
5	1	1	17	1	0.81	3.41	8.5	2.33
7	1.17	1	18	1	0.78	3.49	7.88	2.33
9	1.5	1	19	1	0.49	2.97	7.5	2.33
9	1.5	1	20	0	0.64	2.32	7.71	2.33
8	1.33	1	21	0	0.66	2.07	7.96	2.33
8	1.33	1	22	1	0.64	1.17	7.88	2.33
8	1.6	1	23	1	0.68	1.19	8.83	2.33
1	1	0	20	1	1.99	1.52	7.5	2.96
2	1	0	21	1	2.38	1.48	7.5	2.96
2	1	0	22	1	1.96	1.37	7.79	2.96
2	1	0	23	1	2.18	0.6	8	2.96
2	1	0	24	1	2.05	0.57	8	2.96
2	1	0	25	1	1.69	0.61	7.67	2.96
3	1	0	26	0	1.64	0.59	7	2.96
5	1	0	27	0	1.7	0.55	7.13	2.96
7	1	1	14	0	0.39	0.63	8.88	1.45
6	1	1	15	0	0.72	0.65	7.54	1.45
6	1	1	16	0	1.02	0.64	7.33	1.45
5	1	1	17	1	0.81	0.61	7.5	1.45
7	1.17	1	18	1	0.78	0.66	8.5	1.45
9	1.5	1	19	1	0.49	0.59	7.42	1.45
9	1.5	1	20	0	0.64	0.56	7.71	1.45
8	1.33	1	21	0	0.66	0.44	9	1.45
8	1.33	1	22	1	0.64	0.23	8.29	1.45
5	1	0	27	0	1.7	0.78	7.17	2.52
3	1	1	17	1	6.82	0.49	7	0.84
5	1	1	19	1	10.66	0.57	8	0.84
6	1.2	1	20	1	16.13	0.54	8	0.84
6	1.2	1	21	1	16.08	0.64	8.08	0.84
6	1.2	1	22	1	14.06	0.61	8.5	0.84
6	1.2	1	23	1	12.21	0.44	8.5	0.84
5	1	1	19	1	10.66	1.37	6.58	2.86
6	1.2	1	20	1	16.13	1.16	7.58	2.86
6	1.2	1	21	1	16.08	0.99	8	2.86
6	1.2	1	22	1	14.06	1	8	2.86
6	1.2	1	23	1	12.21	1.75	8	2.86
1	1	1	14	1	1.79	1.67	11.5	4.65
1	1	1	15	1	2.38	1.59	11.13	4.65
1	1	1	16	1	2.47	1.87	9.42	4.65

3	1	1	17	1	2.7	1.45	9	4.65
4	1	1	18	1	2.98	1.35	9.5	4.65
5	1	1	19	1	1.94	1.39	9.5	4.65
6	1.2	1	20	1	1.52	1.37	9.08	4.65
6	1.2	1	21	1	1.83	1.35	9.42	4.65
6	1.2	1	22	0	2.73	1.42	9.5	4.65
6	1.2	1	23	0	2.53	0.9	10.38	4.65
1	1	1	14	1	1.79	3.31	11.5	2.8
1	1	1	15	1	2.38	3.25	11.5	2.8
1	1	1	16	1	2.47	3.26	11	2.8
3	1	1	17	1	2.7	3.29	11.46	2.8
4	1	1	18	1	2.98	3.23	11.5	2.8
1	1	1	14	1	1.79	5.31	7.5	3.31
1	1	1	15	1	2.38	5.74	7.5	3.31
1	1	1	16	1	2.47	5.62	7.58	3.31
3	1	1	17	1	2.7	5.88	7.25	3.31
4	1	1	18	1	2.98	5.55	6.04	3.31
5	1	1	19	1	1.94	2.44	6	3.31
6	1.2	1	20	1	1.52	2.87	6.5	3.31
6	1.2	1	21	1	1.83	1.92	6.33	3.31
6	1.2	1	22	0	2.73	2.27	6.38	3.31
6	1.2	1	23	0	2.53	2.41	7.5	3.31
6	1.2	1	21	1	1.83	0.84	10	5.44
6	1.2	1	22	0	2.73	0.66	10	5.44
6	1.2	1	23	0	2.53	0.59	10.46	5.44
7	1.75	1	30	1	1.54	3.41	8.5	2.33
5	1.25	1	31	1	1.46	3.49	7.88	2.33
6	1.2	1	32	1	1.72	2.97	7.5	2.33
9	1.29	1	33	1	1.85	2.32	7.71	2.33
10	1.25	1	34	1	2	2.07	7.96	2.33
11	1.38	1	35	1	2.13	1.17	7.88	2.33
8	1.14	1	36	0	2.2	1.19	8.83	2.33
1	1	0	22	0	0.51	0.43	8	1.42
2	1	0	23	0	0.51	0.44	8	1.42
2	1	0	24	0	0.65	0.49	8	1.42
2	1	0	25	0	0.83	0.61	8.08	1.42
6	1.2	1	21	1	1.83	1.04	8.38	1.9
6	1.2	1	22	0	2.73	0.85	8.04	1.9
6	1.2	1	23	0	2.53	1.22	8.67	1.9
4	1	1	18	1	2.98	0.49	7.17	0.84
5	1	1	19	1	1.94	0.57	8	0.84
6	1.2	1	20	1	1.52	0.54	8	0.84
6	1.2	1	21	1	1.83	0.64	8.08	0.84
6	1.2	1	22	0	2.73	0.61	8.5	0.84
6	1.2	1	23	0	2.53	0.44	8.5	0.84
6	1.2	1	32	1	1.72	0.59	7.42	1.45
9	1.29	1	33	1	1.85	0.56	7.71	1.45
10	1.25	1	34	1	2	0.44	9	1.45
11	1.38	1	35	1	2.13	0.23	8.29	1.45
8	1.14	1	36	0	2.2	0.1	8.88	1.45
9	1.29	1	33	1	1.85	0.43	10	2.26

10	1.25	1	34	1	2	1.04	8.38	1.9
11	1.38	1	35	1	2.13	0.85	8.04	1.9
8	1.14	1	36	0	2.2	1.22	8.67	1.9
1	1	1	20	1	1.05	0.44	8	1.42
1	1	1	21	1	0.66	0.49	8	1.42
1	1	1	22	0	0.69	0.61	8.08	1.42
1	1	1	23	0	0.75	1.06	8.5	1.42
4	2	1	19	1	1.49	3.34	9	4.17
6	2	1	20	1	1.52	2.82	9	4.17
9	2.25	1	21	1	1.53	2.86	9	4.17
10	2	1	22	1	1.08	0.51	9	4.17
6	2	1	20	1	1.52	5.36	7.5	3.2
9	2.25	1	21	1	1.53	3.94	7.5	3.2
10	2	1	22	1	1.08	2.91	7.5	3.2
2	1	1	23	1	0.72	1.08	7.67	3.2
3	1.5	1	24	1	0.84	1.7	8	3.2
3	1.5	1	25	0	0.93	1.38	7.71	3.2
3	1.5	1	26	0	0.86	1.31	8.25	3.2
9	2.25	1	21	1	1.53	1.74	5.71	2.86
10	2	1	22	1	1.08	1.37	6.58	2.86
10	2	1	22	1	1.08	0.57	8	0.84
1	1	1	13	0	1.63	2.91	7.5	2.33
1	1	1	14	0	1.84	3.15	8	2.33
1	1	1	15	0	1.73	3.51	8.5	2.33
1	1	1	16	0	0.57	3.41	8.5	2.33
1	1	1	17	1	0.5	3.49	7.88	2.33
1	1	1	18	1	1.03	2.97	7.5	2.33
1	1	1	19	1	1.38	2.32	7.71	2.33
1	1	1	20	1	1.65	2.07	7.96	2.33
2	2	1	21	0	2.02	1.17	7.88	2.33
3	1	1	23	1	0.93	1.16	7.58	2.86
9	1.5	1	24	1	1.19	0.99	8	2.86
11	1.38	1	25	1	1.2	1	8	2.86
10	1.43	1	26	1	1.06	1.75	8	2.86
9	1.5	1	24	1	1.19	1.35	9.42	4.65
11	1.38	1	25	1	1.2	1.42	9.5	4.65
10	1.43	1	26	1	1.06	0.9	10.38	4.65
9	1.5	1	24	1	1.19	2.48	9.29	5.28
11	1.38	1	25	1	1.2	1.98	10.5	5.28
10	1.43	1	26	1	1.06	1.18	10.5	5.28
2	1	1	19	1	0.76	1.51	11.5	2.8
2	1	1	20	1	0.83	0.79	11.5	2.8
2	1	0	21	1	0.67	1.96	7.5	1.9
2	1	0	22	1	0.7	1.33	7.5	1.9
8	1.6	0	23	1	0.76	1.35	8.08	1.9
7	1	0	24	1	1.09	1.29	8.67	1.9
8	1	0	25	1	1.08	1.04	8.38	1.9
8	1	0	26	1	1.18	0.85	8.04	1.9
8	1	0	27	1	1.75	1.22	8.67	1.9
7	1	0	24	1	1.09	2.32	7.71	2.33
8	1	0	25	1	1.08	2.07	7.96	2.33

8	1	0	26	1	1.18	1.17	7.88	2.33
8	1	0	27	1	1.75	1.19	8.83	2.33
8	1.6	1	19	1	1.24	2.87	6.5	3.31
8	1.6	1	20	1	1.26	1.92	6.33	3.31
15	1.25	1	21	1	0.8	2.27	6.38	3.31
1	1	0	18	0	3.05	3.31	11.5	2.8
1	1	0	19	0	2.27	3.25	11.5	2.8
1	1	0	20	0	2.36	3.26	11	2.8
1	1	0	21	0	1.82	3.29	11.46	2.8
2	1	0	22	0	1.96	3.23	11.5	2.8
29	4.83	1	18	1	3.81	1.59	10	2.26
39	5.57	1	19	1	4.36	0.43	10	2.26
42	5.25	1	20	1	5.57	0.31	10	2.26
15	1.25	1	21	1	0.8	1.17	7.88	2.33
15	1.25	1	22	1	0.98	1.19	8.83	2.33
15	1.25	1	21	1	0.8	0.85	8.04	1.9
15	1.25	1	22	1	0.98	1.22	8.67	1.9
15	1.25	1	21	1	0.8	1.61	8	2.65
15	1.25	1	22	1	0.98	1.58	8.38	2.65
15	1.25	1	21	1	0.8	1.42	9.5	4.65
15	1.25	1	22	1	0.98	0.9	10.38	4.65
15	1.25	1	22	1	0.98	2.41	7.5	3.31
15	1.25	1	22	1	0.98	0.43	10	2.26
3	1	1	15	1	2.06	6.07	9.5	2.65
5	1.25	1	16	1	2.15	5.98	9.5	2.65
9	1.29	1	17	1	1.22	5.6	8.92	2.65
8	1.14	1	18	1	1.15	4.94	8.5	2.65
8	1.14	1	19	1	1.25	4.69	7.67	2.65
14	1.4	1	20	1	1.29	2.56	7.5	2.65
14	1.4	1	21	1	1.84	2.39	8	2.65
14	1.4	1	22	1	2.29	2	8	2.65
25	1.92	1	23	1	2.88	1.61	8	2.65
19	1.73	1	24	1	2.49	1.58	8.38	2.65
3	1	1	15	1	2.06	3.31	11.5	2.8
5	1.25	1	16	1	2.15	3.25	11.5	2.8
9	1.29	1	17	1	1.22	3.26	11	2.8
8	1.14	1	18	1	1.15	3.29	11.46	2.8
8	1.14	1	19	1	1.25	3.23	11.5	2.8
14	1.4	1	20	1	1.29	1.51	11.5	2.8
14	1.4	1	21	1	1.84	0.79	11.5	2.8
14	1.4	1	22	1	2.29	0.74	11.04	2.8
25	1.92	1	23	0	2.88	0.83	11	2.8
19	1.73	1	24	1	2.49	0.71	11	2.8
14	1.4	1	20	1	1.29	2.97	7.5	2.33
14	1.4	1	21	1	1.84	2.32	7.71	2.33
14	1.4	1	22	1	2.29	2.07	7.96	2.33
25	1.92	1	23	0	2.88	1.17	7.88	2.33
19	1.73	1	24	1	2.49	1.19	8.83	2.33
2	1	1	14	0	2.28	2.12	8.5	1.9
2	1	1	15	0	2.83	2.06	7.71	1.9
3	1	1	18	1	4.22	1.35	8.08	1.9

3	1	1	19	1	6.12	1.29	8.67	1.9
3	1	1	20	1	6.35	1.04	8.38	1.9
3	1	1	21	0	6.57	0.85	8.04	1.9
3	1	1	22	0	9.07	1.22	8.67	1.9
12	2	1	17	0	5.45	4.69	7.67	2.65
18	1.8	1	21	0	5.81	1.61	8	2.65
5	1.25	1	16	1	2.15	2.12	8.5	1.9
9	1.29	1	17	1	1.22	2.06	7.71	1.9
8	1.14	1	18	1	1.15	1.96	7.5	1.9
8	1.14	1	19	1	1.25	1.33	7.5	1.9
14	1.4	1	20	1	1.29	1.35	8.08	1.9
14	1.4	1	21	1	1.84	1.29	8.67	1.9
14	1.4	1	22	1	2.29	1.04	8.38	1.9
25	1.92	1	23	0	2.88	0.85	8.04	1.9
19	1.73	1	24	1	2.49	1.22	8.67	1.9
9	1.29	1	17	1	1.22	0.64	7.33	1.45
8	1.14	1	18	1	1.15	0.61	7.5	1.45
8	1.14	1	19	1	1.25	0.66	8.5	1.45
14	1.4	1	20	1	1.29	0.59	7.42	1.45
14	1.4	1	21	1	1.84	0.56	7.71	1.45
14	1.4	1	22	1	2.29	0.44	9	1.45
25	1.92	1	23	0	2.88	0.23	8.29	1.45
19	1.73	1	24	1	2.49	0.1	8.88	1.45
25	1.92	1	23	0	2.88	0.61	8.5	0.84
19	1.73	1	24	1	2.49	0.44	8.5	0.84
7	1.17	1	19	0	6.44	0.14	9	4.17
7	1.17	1	20	0	6.12	0.2	9	4.17
18	1.8	1	21	0	5.81	0.23	9	4.17
7	1.4	1	22	0	6.69	0.21	9	4.17
16	1.23	1	13	1	3.61	2.18	10	2.26
17	1.21	1	14	1	4.04	2.22	10	2.26
18	1.2	1	16	1	3.79	1.98	10	2.26
17	1.21	1	17	1	1.47	1.59	10	2.26
18	1.29	1	18	1	1.17	0.43	10	2.26
15	1.15	1	19	1	0.98	0.31	10	2.26
16	1.23	1	20	0	1.11	0.22	10	2.26
16	1.23	1	21	0	1.12	0.43	10	2.26
17	1.21	1	14	1	4.04	2.31	6	5.48
18	1.2	1	16	1	3.79	1.42	6.04	5.48
17	1.21	1	17	1	1.47	1.36	6.88	5.48
18	1.29	1	18	1	1.17	1.14	7	5.48
15	1.15	1	19	1	0.98	1.05	7.79	5.48
16	1.23	1	20	0	1.11	1.13	8	5.48
16	1.23	1	21	0	1.12	0.94	8.08	5.48
17	3.4	0	18	0	2.58	1.6	8.5	3.4
17	2.83	0	19	0	2.87	1.8	8.33	3.4
16	1.23	1	13	1	3.61	2.12	8.5	1.9
17	1.21	1	14	1	4.04	2.06	7.71	1.9
18	1.2	1	16	1	3.79	1.33	7.5	1.9
17	1.21	1	17	1	1.47	1.35	8.08	1.9
18	1.29	1	18	1	1.17	1.29	8.67	1.9

15	1.15	1	19	1	0.98	1.04	8.38	1.9
16	1.23	1	20	0	1.11	0.85	8.04	1.9
16	1.23	1	21	0	1.12	1.22	8.67	1.9
17	3.4	0	18	0	2.58	0.85	8	0.84
17	2.83	0	19	0	2.87	0.69	7.17	0.84
5	1.67	0	16	1	2.8	0.61	7.5	1.45
5	1.67	0	17	1	2.7	0.66	8.5	1.45
5	1.67	0	18	1	1.78	0.59	7.42	1.45
5	1.67	0	19	0	2.54	0.56	7.71	1.45
6	1.5	0	20	0	3.04	0.44	9	1.45
6	1.5	0	21	1	2.61	0.23	8.29	1.45
6	1.5	0	22	1	2.66	0.1	8.88	1.45
16	1.23	1	13	1	3.61	3.25	11.5	2.8
17	1.21	1	14	1	4.04	3.26	11	2.8
18	1.2	1	16	1	3.79	3.23	11.5	2.8
17	1.21	1	17	1	1.47	1.51	11.5	2.8
18	1.29	1	18	1	1.17	0.79	11.5	2.8
15	1.15	1	19	1	0.98	0.74	11.04	2.8
16	1.23	1	20	0	1.11	0.83	11	2.8
16	1.23	1	21	0	1.12	0.71	11	2.8
18	1.8	1	21	0	5.81	3.84	7.75	4.43
5	1.25	0	18	1	0.75	2.22	10	2.26
5	1.67	0	19	1	0.77	1.98	10	2.26
7	1.4	0	20	1	0.97	1.98	10	2.26
11	2.2	0	21	1	0.96	1.59	10	2.26
18	1.8	0	23	1	1.27	0.31	10	2.26
19	1.9	0	24	1	1.32	0.22	10	2.26
8	2	0	22	1	0.83	1.04	10	5.44
17	1.89	0	25	1	1.16	0.59	10.46	5.44
18	1.8	0	23	1	1.27	1.04	8.38	1.9
19	1.9	0	24	1	1.32	0.85	8.04	1.9
17	1.89	0	25	1	1.16	1.22	8.67	1.9
1	1	1	27	0	2.01	1.22	8.67	1.9
6	1.5	0	23	0	0.39	0.59	7.42	1.45
6	1.5	0	24	0	0.29	0.56	7.71	1.45
7	1.4	0	25	1	0.35	0.44	9	1.45
6	1.5	0	26	1	0.32	0.23	8.29	1.45
6	1.5	0	27	0	0.34	0.1	8.88	1.45
50	2.27	0	26	1	0.52	0.31	10	2.26
60	2.4	0	27	1	0.71	0.22	10	2.26
56	2.55	0	28	1	0.69	0.43	10	2.26
60	2.4	0	27	1	0.71	0.73	7.04	2.52
56	2.55	0	28	1	0.69	0.78	7.17	2.52
1	1	1	19	1	12.76	4.12	6.67	4.43
1	1	1	20	1	17.99	6.01	6.5	4.43
2	1	1	21	1	9.25	6.22	6.67	4.43
2	1	1	22	1	6.08	6.31	6.75	4.43
2	1	1	23	1	4.43	6.33	6.08	4.43
2	1	1	24	0	4.59	5.22	7.71	4.43
2	1	1	25	0	4.28	6.21	6.42	4.43
2	1	1	26	0	3.05	4.66	7.33	4.43

3	1.5	1	27	0	1.79	3.84	7.75	4.43
5	2.5	1	28	0	1.15	2.64	8.5	4.43
3	1	0	12	0	1.97	3.79	7.58	1.79
3	1	0	13	0	1.57	3.55	7.5	1.79
5	1.67	0	15	0	2.02	2.43	7.5	1.79
5	1.67	0	16	0	2.32	2.33	7.63	1.79
6	1.5	0	17	0	2.59	2.33	6.54	1.79
7	1.75	0	18	1	2.72	2.55	6.5	1.79
6	1.5	0	19	1	2.62	1.99	7.33	1.79
6	1.5	0	20	1	2.05	1.92	8	1.79
7	1.4	0	21	1	2.01	1.59	8.46	1.79
6	1.2	0	14	0	1.32	0.85	8	0.84
5	1.25	0	15	0	1.37	0.69	7.17	0.84
6	1.2	0	16	0	0.7	0.71	7	0.84
6	1.2	0	17	0	0.69	0.49	7	0.84
6	1.2	0	14	0	1.32	2.03	8.5	1.9
6	1.2	0	16	0	0.7	2.06	7.71	1.9
6	1.2	0	17	0	0.69	1.96	7.5	1.9
15	1.67	1	17	1	0.98	2.86	9	4.17
8	1.33	0	22	1	0.13	0.83	11	2.8
11	1.83	0	23	1	0.11	0.71	11	2.8
2	1	0	18	1	1.34	2.32	7.71	2.33
2	1	0	19	1	0.94	2.07	7.96	2.33
13	1.63	1	14	1	1.09	4.06	11.38	5.28
14	1.75	1	15	1	0.93	3.55	8.96	5.28
14	1.75	1	16	1	1.01	2.81	8	5.28
15	1.67	1	17	1	0.98	2.81	8	5.28
3	1	0	14	1	2.89	2.06	7.71	1.9
5	1	0	15	1	3.44	1.96	7.5	1.9
7	1.17	0	16	1	3.62	1.33	7.5	1.9
8	1.14	0	17	0	4.76	1.35	8.08	1.9
12	1.71	0	18	1	4.07	1.29	8.67	1.9
11	1.57	0	19	1	1.92	1.04	8.38	1.9
11	1.83	0	20	0	1.39	0.85	8.04	1.9
12	2	0	21	0	1.29	1.22	8.67	1.9
7	1.17	0	16	1	3.62	0.66	8.5	1.45
12	1.71	0	18	1	4.07	0.56	7.71	1.45
11	1.83	0	20	0	1.39	0.23	8.29	1.45
12	2	0	21	0	1.29	0.1	8.88	1.45
1	1	0	20	1	4.43	3.23	11.5	2.8
1	1	0	21	1	4.39	1.51	11.5	2.8
1	1	0	22	1	4.3	0.79	11.5	2.8
2	1	0	23	1	4.11	0.74	11.04	2.8
2	1	0	24	0	3.55	0.83	11	2.8
2	1	0	25	0	2.48	0.71	11	2.8
2	1	1	13	1	0.96	1.26	6.5	5.48
2	1	1	14	1	0.57	2.09	6.08	5.48
2	1	1	15	1	0.97	2.31	6	5.48
2	1	1	16	1	1.25	2.15	6	5.48
2	1	1	17	1	1.15	1.42	6.04	5.48
3	1	1	18	1	0.96	1.36	6.88	5.48

3	1	1	19	1	1.01	1.14	7	5.48
3	1	1	20	1	1.02	1.05	7.79	5.48
3	1	1	21	0	1.03	1.13	8	5.48
3	1	1	22	1	0.92	0.94	8.08	5.48
3	1	1	18	1	0.96	2.56	7.5	2.65
3	1	1	19	1	1.01	2.39	8	2.65
3	1	1	20	1	1.02	2	8	2.65
3	1	1	21	0	1.03	1.61	8	2.65
3	1	1	22	1	0.92	1.58	8.38	2.65
1	1	0	22	1	2.27	0.43	10	2.26
2	1	0	23	1	1.45	0.31	10	2.26
1	1	0	24	1	1.17	0.22	10	2.26
33	2.54	1	22	1	1.25	1.58	8.38	2.65
5	1	1	15	1	1.53	2.06	7.71	1.9
5	1	1	16	1	2	1.96	7.5	1.9
10	1.43	1	17	1	2.67	1.33	7.5	1.9
12	1.5	1	18	1	2.85	1.35	8.08	1.9
11	1.57	1	19	1	2.19	1.29	8.67	1.9
11	1.57	1	20	1	1.29	1.04	8.38	1.9
12	1.71	1	21	1	1.12	0.85	8.04	1.9
33	2.54	1	22	1	1.25	1.22	8.67	1.9
33	2.54	1	22	1	1.25	0.43	10	2.26
5	1.25	1	13	1	0.94	3.31	11.5	2.8
5	1.25	1	14	1	1.27	3.25	11.5	2.8
5	1	1	15	1	1.53	3.26	11	2.8
5	1	1	16	1	2	3.29	11.46	2.8
10	1.43	1	17	1	2.67	3.23	11.5	2.8
12	1.5	1	18	1	2.85	1.51	11.5	2.8
11	1.57	1	19	1	2.19	0.79	11.5	2.8
11	1.57	1	20	1	1.29	0.74	11.04	2.8
12	1.71	1	21	1	1.12	0.83	11	2.8
33	2.54	1	22	1	1.25	0.71	11	2.8
33	2.54	1	22	1	1.25	1.18	10.5	5.28
12	1.5	1	18	1	2.85	2.09	8.92	4.44
11	1.57	1	19	1	2.19	2.02	9.92	4.44
11	1.57	1	20	1	1.29	2.2	9.67	4.44
12	1.71	1	21	1	1.12	1.25	10.29	4.44
33	2.54	1	22	1	1.25	1.04	10.46	4.44
1	1	1	18	1	0.93	1.29	8.67	1.9
1	1	1	19	1	1.14	1.04	8.38	1.9
1	1	1	20	1	1.49	0.85	8.04	1.9
1	1	1	21	1	1.48	1.22	8.67	1.9
30	3	0	23	1	0.79	0.74	11.04	2.8
32	3.2	0	24	1	0.75	0.83	11	2.8
33	3	0	25	1	0.77	0.71	11	2.8
26	1.73	1	24	0	2.91	2.56	7.5	2.65
29	1.71	1	25	0	2.92	2.39	8	2.65
37	1.85	1	26	0	3.38	2	8	2.65
38	1.81	1	27	1	3.49	1.61	8	2.65
38	1.81	1	28	1	3.59	1.58	8.38	2.65
16	1.23	1	23	0	2.79	1.98	10	2.26

26	1.73	1	24	0	2.91	1.59	10	2.26
29	1.71	1	25	0	2.92	0.43	10	2.26
37	1.85	1	26	0	3.38	0.31	10	2.26
38	1.81	1	27	1	3.49	0.22	10	2.26
38	1.81	1	28	1	3.59	0.43	10	2.26
5	1	1	22	0	2.37	2.51	7.67	3.4
16	1.23	1	23	0	2.79	2.58	8	3.4
26	1.73	1	24	0	2.91	2.33	7.92	3.4
29	1.71	1	25	0	2.92	2.31	7.17	3.4
37	1.85	1	26	0	3.38	2.1	7	3.4
38	1.81	1	27	1	3.49	0.79	7	3.4
38	1.81	1	28	1	3.59	1.07	7.04	3.4
5	1	1	22	0	2.37	5.88	7.25	3.31
16	1.23	1	23	0	2.79	5.55	6.04	3.31
26	1.73	1	24	0	2.91	2.44	6	3.31
29	1.71	1	25	0	2.92	2.87	6.5	3.31
37	1.85	1	26	0	3.38	1.92	6.33	3.31
38	1.81	1	27	1	3.49	2.27	6.38	3.31
38	1.81	1	28	1	3.59	2.41	7.5	3.31
16	1.23	1	23	0	2.79	1.33	7.5	1.9
26	1.73	1	24	0	2.91	1.35	8.08	1.9
29	1.71	1	25	0	2.92	1.29	8.67	1.9
37	1.85	1	26	0	3.38	1.04	8.38	1.9
38	1.81	1	27	1	3.49	0.85	8.04	1.9
38	1.81	1	28	1	3.59	1.22	8.67	1.9
11	1.57	1	21	0	2.73	1.19	8.83	2.33
4	1.33	1	22	0	2.23	0.1	8.88	1.45
2	1	0	16	0	0.59	6.07	9.5	2.65
3	1.5	0	21	0	0.58	2.56	7.5	2.65
3	1.5	0	22	0	0.6	2.39	8	2.65
8	1.6	0	25	0	0.75	1.58	8.38	2.65
1	1	0	18	0	0.81	0.6	8	2.96
2	2	0	19	1	0.5	0.57	8	2.96
2	2	0	20	1	0.9	0.61	7.67	2.96
3	1.5	0	21	1	1.21	0.59	7	2.96
3	1.5	0	22	1	0.53	0.55	7.13	2.96
11	1.57	1	21	0	2.73	1.22	8.67	1.9
8	1.6	0	25	0	0.75	0.44	8.5	0.84
37	4.11	0	24	1	8.35	1.16	7.58	2.86
65	4.33	0	25	1	8.22	0.99	8	2.86
74	4.63	0	26	1	8.11	1	8	2.86
83	4.88	0	27	1	7.68	1.75	8	2.86
6	2	0	16	1	3.4	2.81	8	5.28
11	1.22	0	15	1	1.15	5.62	7.58	3.31
11	1.22	0	16	1	0.99	5.88	7.25	3.31
6	1.2	0	17	1	0.97	0.66	8.5	1.45
8	1.6	0	18	1	0.84	0.59	7.42	1.45
8	1.6	0	19	1	1.44	0.56	7.71	1.45
9	1.8	0	20	1	1.62	0.44	9	1.45
9	1.8	0	21	1	1.52	0.23	8.29	1.45
9	1.5	0	22	0	1.01	0.1	8.88	1.45

2	1	0	13	0	1.12	2.03	8.5	1.9
2	1	0	14	1	1.49	2.12	8.5	1.9
11	1.22	0	15	1	1.15	2.06	7.71	1.9
11	1.22	0	16	1	0.99	1.96	7.5	1.9
4	1.33	1	14	1	0.6	3.31	11.5	2.8
4	1.33	1	15	1	0.72	3.25	11.5	2.8
5	1.67	1	16	1	0.8	3.26	11	2.8
6	1.5	1	17	1	0.97	3.29	11.46	2.8
7	1.4	1	18	1	1.06	3.23	11.5	2.8
11	1.83	1	20	1	0.83	0.79	11.5	2.8
11	1.83	1	21	1	1	0.74	11.04	2.8
13	1.86	1	22	1	1.02	0.83	11	2.8
1	1	0	12	0	0.83	3.97	9.38	4.44
1	1	0	13	0	0.72	3.63	9.42	4.44
1	1	0	14	0	0.51	3.12	7.5	4.44
1	1	0	15	0	0.56	3.3	7.5	4.44
1	1	0	16	1	0.69	3.8	8.13	4.44
7	1.4	1	18	1	1.06	5.55	6.04	3.31
6	1.5	1	19	0	0.91	2.44	6	3.31
11	1.83	1	20	1	0.83	2.87	6.5	3.31
11	1.83	1	21	1	1	1.92	6.33	3.31
13	1.86	1	22	1	1.02	2.27	6.38	3.31
5	1.67	0	17	0	0.62	3.31	11.5	2.8
4	1.33	0	18	0	0.65	3.25	11.5	2.8
5	1.25	0	19	0	0.37	3.26	11	2.8
2	1	0	14	1	1.59	2.12	8.5	1.9
2	1	0	15	1	1.9	2.06	7.71	1.9
2	1	0	16	1	2.04	1.96	7.5	1.9
2	1	0	17	1	2.61	1.33	7.5	1.9
2	1	0	18	0	2.76	1.35	8.08	1.9
2	1	0	19	0	2.64	1.29	8.67	1.9
2	1	0	21	0	2.73	0.85	8.04	1.9
4	1.33	0	22	0	2.77	1.22	8.67	1.9
4	1.33	0	22	0	2.77	0.1	8.88	1.45
3	1.5	1	19	1	0.94	0.54	8	0.84
5	1.67	1	20	1	0.63	0.64	8.08	0.84
6	1.5	1	21	1	0.66	0.61	8.5	0.84
6	1.5	1	22	1	0.75	0.44	8.5	0.84
11	1.57	0	22	0	1.27	0.83	11	2.8
12	1.5	0	23	0	1.11	0.71	11	2.8
12	1.5	0	24	0	1.4	0.85	8.04	1.9
12	1.5	0	25	0	1.45	1.22	8.67	1.9
10	1.25	1	17	1	1.28	0.44	9	1.45
11	1.38	1	18	1	0.83	0.23	8.29	1.45
11	1.22	1	19	1	0.81	0.1	8.88	1.45
10	1.25	1	17	1	1.28	1.04	8.38	1.9
11	1.38	1	18	1	0.83	0.85	8.04	1.9
11	1.22	1	19	1	0.81	1.22	8.67	1.9
4	1	0	13	1	0.99	2.91	7.5	2.33
4	1	0	14	1	0.77	3.15	8	2.33
4	1	0	15	1	0.85	3.51	8.5	2.33

4	1	0	16	1	1.38	3.41	8.5	2.33
10	1.25	0	17	1	1.71	3.49	7.88	2.33
9	1.13	0	18	1	1.67	2.97	7.5	2.33
9	1.13	0	19	1	1.01	2.32	7.71	2.33
10	1.43	0	22	1	0.93	1.19	8.83	2.33
4	1	1	14	1	2.24	5.6	8.92	2.65
4	1	1	15	1	2.44	4.94	8.5	2.65
6	1	1	16	1	2.79	4.69	7.67	2.65
6	1	1	17	1	3.42	2.56	7.5	2.65
6	1	1	18	0	2.45	2.39	8	2.65
6	1	1	19	0	2.11	2	8	2.65
6	1	1	20	1	2.63	1.61	8	2.65
5	1	1	21	1	2.33	1.58	8.38	2.65
2	1	1	12	0	1.87	3.31	11.5	2.8
3	1	1	13	1	1.55	3.25	11.5	2.8
4	1	1	14	1	2.24	3.26	11	2.8
4	1	1	15	1	2.44	3.29	11.46	2.8
6	1	1	16	1	2.79	3.23	11.5	2.8
6	1	1	17	1	3.42	1.51	11.5	2.8
6	1	1	18	0	2.45	0.79	11.5	2.8
6	1	1	19	0	2.11	0.74	11.04	2.8
6	1	1	20	1	2.63	0.83	11	2.8
5	1	1	21	1	2.33	0.71	11	2.8
3	1	1	13	1	1.55	0.69	7.17	0.84
4	1	1	14	1	2.24	0.71	7	0.84
4	1	1	15	1	2.44	0.49	7	0.84
6	1	1	16	1	2.79	0.49	7.17	0.84
6	1	1	17	1	3.42	0.57	8	0.84
6	1	1	18	0	2.45	0.54	8	0.84
6	1	1	19	0	2.11	0.64	8.08	0.84
6	1	1	20	1	2.63	0.61	8.5	0.84
5	1	1	21	1	2.33	0.44	8.5	0.84
1	1	0	16	1	0.81	1.61	2.5	2.86
1	1	0	17	1	0.87	2.58	2.58	2.86
1	1	0	18	1	1.17	2.34	4	2.86
8	1.14	0	22	1	0.49	0.6	8	5.07
8	1.14	0	23	1	0.5	1.22	8	5.07
8	1.14	0	24	0	0.7	1.25	8	5.07
9	1.29	0	25	0	0.77	1.13	8.08	5.07
8	1.14	1	20	1	1.26	1.13	8	5.48
8	1.14	1	20	1	1.26	0.59	8	5.83
8	1.14	1	20	1	1.26	1.42	9.5	4.65
1	1	1	27	0	0.48	1.58	8.38	2.65
2	2	0	16	1	1.68	0.85	8	0.84
2	2	0	17	1	1.77	0.69	7.17	0.84
3	3	0	18	1	2.06	0.71	7	0.84
4	1.33	0	19	1	1.35	0.49	7	0.84
5	1.67	0	20	1	1.62	0.49	7.17	0.84
5	1.67	0	21	0	1.4	0.57	8	0.84
2	1	0	22	1	0.82	0.54	8	0.84
1	1	0	23	1	0.89	0.64	8.08	0.84

11	1.22	0	13	1	0.72	3.83	9	4.17
11	1.22	0	14	1	0.95	3.34	9	4.17
10	1.11	0	15	1	0.8	2.82	9	4.17
5	1	0	17	0	0.82	3.29	11.46	2.8
4	1	0	18	0	0.67	3.23	11.5	2.8
4	1	0	19	0	0.8	1.51	11.5	2.8
4	1	0	20	1	0.8	0.79	11.5	2.8
4	1	0	14	1	1.21	3.25	11.5	2.8
4	1	0	15	1	1.27	3.26	11	2.8
4	1	0	16	1	0.74	3.29	11.46	2.8
4	1	0	17	1	1.62	3.23	11.5	2.8
3	1	0	18	1	1.13	1.51	11.5	2.8
1	1	0	19	1	2.3	0.79	11.5	2.8
4	1.33	0	20	1	1.59	0.74	11.04	2.8
4	1.33	0	21	1	1.38	0.83	11	2.8
4	1.33	0	22	1	1.35	0.71	11	2.8
2	1	1	22	0	1.49	0.56	7.71	1.45
2	1	1	23	0	1.72	0.44	9	1.45
2	1	1	24	0	1.89	0.23	8.29	1.45
2	1	1	25	0	1.66	0.1	8.88	1.45
10	1	0	24	0	2.21	0.56	7.71	1.45
12	1	0	25	0	1.66	0.44	9	1.45
17	1.13	0	26	1	1.84	0.23	8.29	1.45
22	1.57	0	27	1	1.81	0.1	8.88	1.45
12	1	0	25	0	1.66	2	8	2.65
17	1.13	0	26	1	1.84	1.61	8	2.65
22	1.57	0	27	1	1.81	1.58	8.38	2.65
6	1.2	0	16	0	3.03	2.91	7.5	2.33
6	1.2	0	17	0	3.78	3.15	8	2.33
6	1.2	0	18	0	3.76	3.51	8.5	2.33
6	1.2	0	19	0	4.65	3.41	8.5	2.33
6	1.2	0	21	1	5.82	2.97	7.5	2.33
6	1.2	0	22	1	4.16	2.32	7.71	2.33
6	1.2	0	23	0	5.28	2.07	7.96	2.33
7	1.17	0	24	0	4.21	1.17	7.88	2.33
7	1.17	0	25	0	4.49	1.19	8.83	2.33
11	2.2	0	14	1	2.51	9.46	8.92	2.53
11	2.2	0	15	1	2.63	4.51	8.5	2.53
13	1.86	0	16	1	1.9	3.7	8.5	2.53
15	1.67	0	17	1	1.95	2.66	6.29	2.53
14	1.75	0	18	1	1.83	3.19	6.33	2.53
15	1.5	0	19	1	1.91	2.96	6.42	2.53
15	1.67	0	17	1	1.95	2.97	7.5	2.33
15	1.5	0	19	1	1.91	2.07	7.96	2.33
14	1.56	0	20	1	1.73	1.17	7.88	2.33
2	1	1	12	0	1.18	1.67	11.5	4.65
2	1	1	13	0	1.47	1.59	11.13	4.65
6	1	1	14	0	1.24	1.87	9.42	4.65
9	1	1	15	1	1.4	1.45	9	4.65
11	1	1	16	1	1.35	1.35	9.5	4.65
12	1	1	17	1	1.65	1.39	9.5	4.65

5	1.25	0	21	1	1.05	1.29	8.67	1.9
8	1.14	0	22	1	1.21	1.04	8.38	1.9
12	1.5	0	23	1	1.54	0.85	8.04	1.9
16	1.33	0	24	1	1.64	1.22	8.67	1.9
24	1.71	1	15	1	3.03	3.26	11	2.8
17	1.55	1	16	1	1.4	3.29	11.46	2.8
16	1.45	1	17	1	1.44	3.23	11.5	2.8
17	1.42	1	18	1	1.83	1.51	11.5	2.8
18	1.5	1	19	1	2.68	0.79	11.5	2.8
16	1.78	1	20	1	3.21	0.74	11.04	2.8
12	2	1	21	1	2.34	0.83	11	2.8
11	2.75	1	22	1	2.09	0.71	11	2.8
14	1.56	0	20	1	1.73	1.17	7.88	2.33
14	1.27	0	21	1	1.61	1.19	8.83	2.33
6	1	1	20	1	7.05	0.49	8	1.42
6	1	1	21	1	7.03	0.61	8.08	1.42
6	1	1	22	1	8.94	1.06	8.5	1.42
12	1	1	17	1	1.65	1.37	6.58	2.86
6	1	1	14	0	1.24	3.51	8.5	2.33
9	1	1	15	1	1.4	3.41	8.5	2.33
11	1	1	16	1	1.35	3.49	7.88	2.33
12	1	1	17	1	1.65	2.97	7.5	2.33
6	1	1	14	0	1.24	2.06	7.71	1.9
9	1	1	15	1	1.4	1.96	7.5	1.9
11	1	1	16	1	1.35	1.33	7.5	1.9
12	1	1	17	1	1.65	1.35	8.08	1.9
2	1	1	18	1	2.25	1.29	8.67	1.9
4	1	1	19	1	1.97	1.04	8.38	1.9
3	1	1	20	0	1.85	0.85	8.04	1.9
4	1	1	21	0	1.69	1.22	8.67	1.9
6	1	1	14	0	1.24	0.64	7.33	1.45
9	1	1	15	1	1.4	0.61	7.5	1.45
11	1	1	16	1	1.35	0.66	8.5	1.45
12	1	1	17	1	1.65	0.59	7.42	1.45
12	1.71	1	13	1	2.85	2.03	8.5	1.9
17	1.89	1	14	1	2.66	2.12	8.5	1.9
24	1.71	1	15	1	3.03	2.06	7.71	1.9
17	1.55	1	16	1	1.4	0.61	7.5	1.45
16	1.45	1	17	1	1.44	0.66	8.5	1.45
17	1.42	1	18	1	1.83	0.59	7.42	1.45
18	1.5	1	19	1	2.68	0.56	7.71	1.45
16	1.78	1	20	1	3.21	0.44	9	1.45
12	2	1	21	1	2.34	0.23	8.29	1.45
12	1.71	1	13	1	2.85	2.91	7.5	2.33
17	1.89	1	14	1	2.66	3.15	8	2.33
24	1.71	1	15	1	3.03	3.51	8.5	2.33
17	1.55	1	16	1	1.4	3.41	8.5	2.33
16	1.45	1	17	1	1.44	3.49	7.88	2.33
17	1.42	1	18	1	1.83	2.97	7.5	2.33
18	1.5	1	19	1	2.68	2.32	7.71	2.33
16	1.78	1	20	1	3.21	2.07	7.96	2.33

12	2	1	21	1	2.34	1.17	7.88	2.33
8	1.14	0	22	1	1.21	1.92	6.33	3.31
12	1.5	0	23	1	1.54	2.27	6.38	3.31
16	1.33	0	24	1	1.64	2.41	7.5	3.31
4	1	0	17	1	0.66	0.74	11.04	2.8
5	1	0	18	1	1.51	0.83	11	2.8
5	1	0	19	1	0.88	0.71	11	2.8
6	1	0	21	1	0.7	2.39	8	2.65
10	1	0	22	1	0.51	2	8	2.65
11	1	0	23	1	0.65	1.61	8	2.65
13	1.08	0	24	1	0.88	1.58	8.38	2.65
4	1	0	17	1	0.66	0.31	10	2.26
5	1	0	18	1	1.51	0.22	10	2.26
5	1	0	19	1	0.88	0.43	10	2.26
9	2.25	0	30	1	0.7	2.97	7.5	2.33
13	2.17	0	31	1	1.16	2.32	7.71	2.33
14	1.75	0	32	1	1.62	2.07	7.96	2.33
15	1.88	0	33	0	2.34	1.17	7.88	2.33
15	1.88	0	34	0	1.6	1.19	8.83	2.33
1	1	1	11	0	2.95	2.03	8.5	1.9
1	1	1	12	0	3.44	2.12	8.5	1.9
1	1	1	13	0	2.88	2.06	7.71	1.9
1	1	1	14	0	1.66	1.96	7.5	1.9
1	1	1	15	0	2.56	1.33	7.5	1.9
1	1	1	16	0	1.69	1.35	8.08	1.9
2	1	1	17	0	2.12	1.29	8.67	1.9
2	1	1	18	1	2.55	1.04	8.38	1.9
2	1	1	19	1	3.18	0.85	8.04	1.9
2	1	1	20	0	2.53	1.22	8.67	1.9
13	2.17	0	31	1	1.16	0.79	11.5	2.8
14	1.75	0	32	1	1.62	0.74	11.04	2.8
15	1.88	0	33	0	2.34	0.83	11	2.8
15	1.88	0	34	0	1.6	0.71	11	2.8
2	1	1	21	0	1.1	3.23	11.5	2.8
2	1	1	22	1	1.37	1.51	11.5	2.8
2	1	1	23	1	1.48	0.79	11.5	2.8
2	1	1	24	1	1.3	0.74	11.04	2.8
2	1	1	25	0	1.41	0.83	11	2.8
2	1	1	26	0	1.75	0.71	11	2.8
23	1.53	0	24	1	3.64	1.04	8.38	1.9
5	1	1	26	1	1.86	1.19	8.83	2.33
4	1	1	25	1	1.84	0.83	11	2.8
5	1	1	26	1	1.86	0.71	11	2.8
1	1	1	17	0	2.33	6.07	9.5	2.65
1	1	1	18	0	1.42	5.98	9.5	2.65
2	1	1	19	0	1.73	5.6	8.92	2.65
2	1	1	20	0	152.21	4.94	8.5	2.65
2	1	1	21	0	8.13	4.69	7.67	2.65
2	1	1	22	0	25.07	2.56	7.5	2.65
2	1	1	23	0	21.81	2.39	8	2.65
2	1	1	24	0	16.8	2	8	2.65

2	1	1	25	1	8	1.61	8	2.65
2	1	1	19	0	1.73	2.06	7.71	1.9
2	1	1	20	0	152.21	1.96	7.5	1.9
2	1	1	21	0	8.13	1.33	7.5	1.9
2	1	1	22	0	25.07	1.35	8.08	1.9
2	1	1	23	0	21.81	1.29	8.67	1.9
2	1	1	24	0	16.8	1.04	8.38	1.9
2	1	1	25	1	8	0.85	8.04	1.9
1	1	1	26	1	9.38	1.22	8.67	1.9
3	1.5	1	22	0	0.34	0.57	8	0.84
3	1.5	1	23	0	0.34	0.54	8	0.84
3	1.5	1	24	0	0.42	0.64	8.08	0.84
3	1.5	1	25	0	0.42	0.61	8.5	0.84
3	1.5	1	26	0	0.4	0.44	8.5	0.84
7	1	0	20	1	2.08	3.29	11.46	2.8
7	1	0	21	1	2.1	3.23	11.5	2.8
6	1	0	22	1	2	1.51	11.5	2.8
7	1.17	0	23	1	1.39	0.79	11.5	2.8
15	1.88	0	24	0	1.59	0.74	11.04	2.8
31	2.07	0	25	0	1.7	0.83	11	2.8
33	2.2	0	26	0	1.92	0.71	11	2.8
3	1	0	11	1	0.52	3.31	11.5	2.8
3	1	0	12	1	0.36	3.25	11.5	2.8
5	1.67	0	13	1	0.31	3.26	11	2.8
5	1.67	0	14	1	0.35	3.29	11.46	2.8
5	1.67	0	15	1	0.52	3.23	11.5	2.8
3	1	0	16	1	0.59	1.51	11.5	2.8
4	1.33	0	17	1	0.85	0.79	11.5	2.8
5	2.5	0	18	1	0.84	0.74	11.04	2.8
8	2	0	19	1	0.88	0.83	11	2.8
3	1.5	0	20	1	0.77	0.71	11	2.8
1	1	0	15	0	0.95	3.31	11.5	2.8
1	1	0	16	0	0.61	3.25	11.5	2.8
8	1.6	0	16	1	1.4	3.29	11.46	2.8
9	1.5	0	17	1	1.5	3.23	11.5	2.8
13	2.17	0	18	1	2.35	1.51	11.5	2.8
15	1.88	0	19	1	2.05	0.79	11.5	2.8
27	1.69	0	20	1	1.9	0.74	11.04	2.8
20	2	0	21	1	1.98	0.83	11	2.8
21	2.1	0	22	1	1.76	0.71	11	2.8
6	1.2	1	11	1	0.98	0.63	8.88	1.45
6	1.2	1	12	1	0.98	0.65	7.54	1.45
6	1.2	1	13	1	0.98	0.64	7.33	1.45
6	1.2	1	14	1	0.69	0.61	7.5	1.45
9	1.13	1	17	1	0.29	0.56	7.71	1.45
10	1.43	1	18	0	0.23	0.44	9	1.45
10	1.25	1	19	0	0.32	0.23	8.29	1.45
10	1.25	1	20	0	0.41	0.1	8.88	1.45
13	1.44	0	18	1	4.38	1.59	10	2.26
12	1.5	0	19	1	5.02	0.43	10	2.26
14	1.56	0	20	0	2.24	0.31	10	2.26

13	1.63	0	21	1	1.8	0.22	10	2.26
12	1.71	0	22	1	1.66	0.43	10	2.26
16	2.29	1	20	1	0.38	2.39	8	2.65
18	2.57	1	21	1	0.34	2	8	2.65
19	2.38	1	22	1	0.29	1.61	8	2.65
21	2.63	1	23	1	0.27	1.58	8.38	2.65
2	2	1	25	0	2.52	0.44	9	1.45
2	1	1	26	0	2.49	0.23	8.29	1.45
3	1	1	27	0	1.65	0.1	8.88	1.45
9	1.13	1	17	1	0.29	2.32	7.71	2.33
10	1.43	1	18	0	0.23	2.07	7.96	2.33
10	1.25	1	19	0	0.32	1.17	7.88	2.33
10	1.25	1	20	0	0.41	1.19	8.83	2.33
12	2.4	1	19	1	0.45	0.51	9	4.17
16	2.29	1	20	1	0.38	0.14	9	4.17
18	2.57	1	21	1	0.34	0.2	9	4.17
19	2.38	1	22	1	0.29	0.23	9	4.17
21	2.63	1	23	1	0.27	0.21	9	4.17
16	2.29	1	20	1	0.38	0.44	8	1.42
18	2.57	1	21	1	0.34	0.49	8	1.42
19	2.38	1	22	1	0.29	0.61	8.08	1.42
21	2.63	1	23	1	0.27	1.06	8.5	1.42
9	3	1	18	1	0.49	1.74	5.71	2.86
12	2.4	1	19	1	0.45	1.37	6.58	2.86
16	2.29	1	20	1	0.38	1.16	7.58	2.86
18	2.57	1	21	1	0.34	0.99	8	2.86
19	2.38	1	22	1	0.29	1	8	2.86
21	2.63	1	23	1	0.27	1.75	8	2.86
19	2.38	1	22	1	0.29	1.25	8	5.07
21	2.63	1	23	1	0.27	1.13	8.08	5.07
2	2	1	15	1	0.83	0.65	7.54	1.45
2	2	1	16	1	0.74	0.64	7.33	1.45
3	1.5	1	17	1	0.61	0.61	7.5	1.45
9	3	1	18	1	0.49	0.66	8.5	1.45
12	2.4	1	19	1	0.45	0.59	7.42	1.45
16	2.29	1	20	1	0.38	0.56	7.71	1.45
18	2.57	1	21	1	0.34	0.44	9	1.45
19	2.38	1	22	1	0.29	0.23	8.29	1.45
21	2.63	1	23	1	0.27	0.1	8.88	1.45
4	1.33	1	24	0	1.79	0.59	10.46	5.44
4	1.33	0	25	1	11.65	0.79	11.5	2.8
9	1.5	0	26	1	12.08	0.74	11.04	2.8
6	1.5	0	27	1	13.23	0.83	11	2.8
8	1.6	0	28	0	12.86	0.71	11	2.8
2	1	1	29	0	17.93	5.6	8.92	2.65
2	1	1	30	0	17.02	4.94	8.5	2.65
2	1	1	31	0	34.77	4.69	7.67	2.65
2	1	1	32	0	35.51	2.56	7.5	2.65
2	1	1	33	1	148.07	2.39	8	2.65
2	1	1	34	1	18.53	2	8	2.65
2	1	1	21	0	2.13	3.29	11.46	2.8

2	1	1	22	0	2.31	3.23	11.5	2.8
2	1	1	23	0	1.51	1.51	11.5	2.8
2	1	1	25	1	2.06	0.74	11.04	2.8
3	1	1	26	1	2.06	0.83	11	2.8
4	1	1	27	1	1.88	0.71	11	2.8
2	1	1	21	0	2.13	1.96	7.5	1.9
2	1	1	22	0	2.31	1.33	7.5	1.9
2	1	1	23	0	1.51	1.35	8.08	1.9
2	1	1	25	1	2.06	1.04	8.38	1.9
3	1	1	26	1	2.06	0.85	8.04	1.9
4	1	1	27	1	1.88	1.22	8.67	1.9
6	1.2	1	19	0	2.6	2.91	7.5	2.33
6	1.2	1	20	0	2.64	3.15	8	2.33
7	1.4	1	21	0	2.76	3.51	8.5	2.33
6	1.2	1	22	0	2.65	3.41	8.5	2.33
6	1.2	1	23	0	3.13	3.49	7.88	2.33
4	1.33	1	24	1	0.87	2.97	7.5	2.33
6	1.2	1	25	1	1.02	2.32	7.71	2.33
4	1.33	1	26	1	1.17	2.07	7.96	2.33
9	1.8	1	27	1	1.43	1.17	7.88	2.33
10	1.67	1	28	1	1.52	1.19	8.83	2.33
6	1.2	1	19	0	2.6	0.63	8.88	1.45
6	1.2	1	20	0	2.64	0.65	7.54	1.45
7	1.4	1	21	0	2.76	0.64	7.33	1.45
6	1.2	1	22	0	2.65	0.61	7.5	1.45
6	1.2	1	23	0	3.13	0.66	8.5	1.45
6	1.2	1	25	1	1.02	0.56	7.71	1.45
6	1.2	1	19	0	2.6	2.33	10	3.46
6	1.2	1	20	0	2.64	2.66	10	3.46
7	1.4	1	21	0	2.76	2.59	8.88	3.46
6	1.2	1	22	0	2.65	2.03	8.79	3.46
6	1.2	1	23	0	3.13	1.17	9	3.46
6	1.2	1	25	1	1.02	0.37	8.5	3.46
10	1.67	1	28	1	1.52	0.44	7.5	3.46
36	3	1	32	0	2.88	1.35	9.42	4.65
39	3.25	1	33	0	2.15	1.42	9.5	4.65
37	3.08	1	34	0	1.99	0.9	10.38	4.65
36	3	1	32	0	2.88	0.2	9	4.17
39	3.25	1	33	0	2.15	0.23	9	4.17
37	3.08	1	34	0	1.99	0.21	9	4.17
36	3	1	32	0	2.88	2.48	9.29	5.28
39	3.25	1	33	0	2.15	1.98	10.5	5.28
37	3.08	1	34	0	1.99	1.18	10.5	5.28
6	1.5	1	24	1	4.63	1.35	8.08	1.9
6	1.5	1	25	0	3.79	1.29	8.67	1.9
6	1.5	1	26	0	1.91	1.04	8.38	1.9
10	1.25	1	27	0	2.08	0.85	8.04	1.9
36	3	1	32	0	2.88	1.38	9	3.8
39	3.25	1	33	0	2.15	1.44	9	3.8
37	3.08	1	34	0	1.99	1.01	10.04	3.8
10	1.25	1	27	0	2.08	1.17	7.88	2.33

10	1.25	1	28	0	2.38	1.19	8.83	2.33
10	1.25	1	27	0	2.08	0.83	11	2.8
10	1.25	1	28	0	2.38	0.71	11	2.8
10	1.25	1	28	0	2.38	1.22	8.67	1.9
9	3	0	26	0	1.71	0.64	8.08	0.84
12	4	0	27	1	1.74	0.61	8.5	0.84
10	3.33	0	28	1	1.65	0.44	8.5	0.84
5	1.67	0	20	1	1.71	0.65	7.54	1.45
5	1.67	0	21	1	1.79	0.64	7.33	1.45
5	1.67	0	22	1	1.97	0.61	7.5	1.45
1	1	0	16	0	3.98	1.67	11.5	4.65
1	1	0	17	0	3.71	1.59	11.13	4.65
1	1	0	18	0	6.5	1.87	9.42	4.65
1	1	0	19	0	1.64	1.45	9	4.65
3	1.5	1	25	1	2	0.83	11	2.8
3	1.5	1	26	1	2.27	0.71	11	2.8
7	1.75	0	24	0	1.2	3.31	11.5	2.8
8	2	0	25	0	1.36	3.25	11.5	2.8
7	1.75	0	26	0	0.82	3.26	11	2.8
7	1.75	0	27	1	1	3.29	11.46	2.8
7	1.75	0	28	1	0.77	3.23	11.5	2.8
8	2	0	29	1	1.1	1.51	11.5	2.8
9	2.25	0	30	1	1.16	0.79	11.5	2.8
9	3	0	31	1	1.15	0.74	11.04	2.8
12	3	0	32	1	1.85	0.83	11	2.8
21	2.1	0	33	1	2.01	0.71	11	2.8
12	2	0	20	1	0.96	5.98	9.5	2.65
10	1.67	0	21	1	0.87	5.6	8.92	2.65
10	1.67	0	22	1	0.86	4.94	8.5	2.65
14	2.33	0	24	1	0.51	2.56	7.5	2.65
14	2.33	0	25	1	0.66	2.39	8	2.65
14	2.33	0	26	1	0.67	2	8	2.65
17	2.83	0	27	1	0.71	1.61	8	2.65
19	2.38	0	28	1	0.82	1.58	8.38	2.65
3	1	1	25	1	1.55	0.76	8	5.83
3	1	1	26	0	1.67	0.59	8	5.83
3	1	1	27	0	1.83	1.81	7.13	5.83
2	1	0	23	0	1.53	0.83	11	2.8
1	1	0	24	0	2.4	0.71	11	2.8
1	1	1	18	1	0.94	3.31	11.5	2.8
1	1	1	19	1	0.81	3.25	11.5	2.8
1	1	1	20	1	0.92	3.26	11	2.8
1	1	1	21	1	0.89	3.29	11.46	2.8
1	1	1	22	1	0.78	3.23	11.5	2.8
1	1	1	23	1	0.35	1.51	11.5	2.8
1	1	1	24	1	0.36	0.79	11.5	2.8
1	1	1	25	0	0.37	0.74	11.04	2.8
2	1	1	26	1	0.27	0.83	11	2.8
1	1	1	27	1	0.28	0.71	11	2.8
20	4	0	27	0	2.05	0.84	10	5.44
24	4	0	28	1	0.63	0.66	10	5.44

15	2.5	0	29	1	0.44	0.59	10.46	5.44
4	1.33	1	19	0	5.75	4.69	7.67	2.65
4	1.33	1	20	0	5	2.56	7.5	2.65
4	1.33	1	21	0	5.81	2.39	8	2.65
4	1.33	1	22	0	4.92	2	8	2.65
4	1.33	1	23	0	5.51	1.61	8	2.65
5	1.25	1	24	0	4.58	1.58	8.38	2.65
13	1.18	0	24	1	3.25	0.76	8	5.83
16	1.45	0	25	1	2.83	0.59	8	5.83
5	1.25	0	26	1	2.56	1.81	7.13	5.83
14	2.33	0	24	1	1.96	1.39	9.5	4.65
55	3.06	0	25	1	1.84	1.37	9.08	4.65
58	3.05	0	26	1	1.71	1.35	9.42	4.65
104	3.47	0	27	1	3.31	1.42	9.5	4.65
114	3.68	0	28	1	3.17	0.9	10.38	4.65
8	2.67	0	25	1	0.28	0.74	11.04	2.8
9	3	0	26	1	0.45	0.83	11	2.8
9	2.25	0	27	0	0.36	0.71	11	2.8
57	3.17	1	24	0	6.4	1.81	7.13	5.83
30	3	1	21	1	8.2	1.16	7.58	2.86
38	3.45	1	22	1	8.58	0.99	8	2.86
42	3.23	1	23	0	7.74	1	8	2.86
57	3.17	1	24	0	6.4	1.75	8	2.86
30	3	1	21	1	8.2	0.79	11.5	2.8
38	3.45	1	22	1	8.58	0.74	11.04	2.8
42	3.23	1	23	0	7.74	0.83	11	2.8
57	3.17	1	24	0	6.4	0.71	11	2.8
2	1	1	16	0	0.52	2.12	8.5	1.9
3	1	1	17	0	0.48	2.06	7.71	1.9
4	1	1	18	0	0.49	1.96	7.5	1.9
4	1	1	19	0	0.52	1.33	7.5	1.9
4	1	1	20	0	0.61	1.35	8.08	1.9
57	3.17	1	24	0	6.4	0.1	8.88	1.45
57	3.17	1	24	0	6.4	0.44	8.5	0.84
9	1.13	0	20	1	0.53	6.41	8.67	3.2
12	1.2	0	21	1	0.62	6.39	8.13	3.2
14	1.27	0	22	1	0.64	5.87	7.88	3.2
15	1.25	0	23	1	0.72	5.36	7.5	3.2
15	1.25	0	24	1	0.75	3.94	7.5	3.2
16	1.33	0	25	1	0.94	2.91	7.5	3.2
9	1.13	0	26	1	0.48	1.08	7.67	3.2
9	1.13	0	27	1	0.43	1.7	8	3.2
10	1.11	0	28	1	0.52	1.38	7.71	3.2
10	1.11	0	29	1	0.65	1.31	8.25	3.2
12	1.2	0	21	1	0.62	1.94	10	5.44
14	1.27	0	22	1	0.64	1.97	10	5.44
15	1.25	0	23	1	0.72	1.73	10	5.44
15	1.25	0	24	1	0.75	1.71	10	5.44
16	1.33	0	25	1	0.94	1.34	10	5.44
10	2.5	0	21	0	0.46	2.82	9	4.17
10	2.5	0	22	0	0.35	2.86	9	4.17

10	2.5	0	23	0	0.27	0.51	9	4.17
11	2.2	0	24	0	0.28	0.14	9	4.17
10	2	0	25	0	0.28	0.2	9	4.17
12	2	0	27	0	0.29	0.21	9	4.17
10	2	0	26	0	0.25	0.83	11	2.8
1	1	1	22	0	1.66	3.41	8.5	2.33
1	1	1	23	0	1.47	3.49	7.88	2.33
1	1	1	24	1	1.65	2.97	7.5	2.33
2	1	1	25	1	1.62	2.32	7.71	2.33
2	1	1	26	1	1.78	2.07	7.96	2.33
3	1	1	27	0	1.74	1.17	7.88	2.33
2	1	1	28	0	1.63	1.19	8.83	2.33
3	1	0	26	0	3.68	0.83	11	2.8
22	1.57	0	27	0	2.06	0.71	11	2.8
28	3.5	0	22	1	7.83	1.04	8.38	1.9
28	3.11	0	23	1	8.32	0.85	8.04	1.9
31	3.88	0	24	1	7.85	1.22	8.67	1.9
11	2.2	0	34	1	2.09	1.99	7.33	1.79
2	1	1	26	0	0.55	1.17	7.88	2.33
2	1	1	27	0	0.51	1.19	8.83	2.33
1	1	1	18	0	0.99	2.16	10	2.26
1	1	1	19	0	1.47	2.18	10	2.26
1	1	1	20	0	1.62	2.22	10	2.26
1	1	1	21	0	1.1	1.98	10	2.26
1	1	1	22	0	0.99	1.98	10	2.26
1	1	1	23	0	0.83	1.59	10	2.26
1	1	1	24	1	0.65	0.43	10	2.26
1	1	1	25	1	0.61	0.31	10	2.26
1	1	1	26	1	0.58	0.22	10	2.26
1	1	1	27	1	0.4	0.43	10	2.26
1	1	1	39	0	8.5	0.74	11.04	2.8
1	1	1	40	0	3.15	0.83	11	2.8
19	1.9	0	27	1	0.62	0.38	9.58	3.8
6	2	0	23	1	1.51	0.51	9	4.17
6	2	0	24	1	1.61	0.14	9	4.17
6	1.5	0	25	1	1.46	0.2	9	4.17
10	2	0	26	1	0.89	0.23	9	4.17
19	1.9	0	27	1	0.62	0.21	9	4.17
2	1	0	30	1	2.12	1.98	10	2.26
2	1	0	31	1	2.37	1.59	10	2.26
3	1	0	32	1	2.22	0.43	10	2.26
3	1	0	33	1	2.23	0.31	10	2.26
18	4.5	0	34	1	2.31	0.22	10	2.26
20	3.33	0	35	0	19.99	0.43	10	2.26
3	1.5	0	20	1	1.91	1.52	7.5	2.96
3	1.5	0	21	1	1.82	1.48	7.5	2.96
6	2	0	23	1	1.51	0.6	8	2.96
6	2	0	24	1	1.61	0.57	8	2.96
6	1.5	0	25	1	1.46	0.61	7.67	2.96
10	2	0	26	1	0.89	0.59	7	2.96
19	1.9	0	27	1	0.62	0.55	7.13	2.96

3	1.5	1	18	1	2.01	3.31	11.5	2.8
3	1.5	1	19	1	2.01	3.25	11.5	2.8
2	1	1	20	1	1.87	3.26	11	2.8
6	3	0	26	1	2.63	0.74	11.04	2.8
6	3	0	27	1	2.68	0.83	11	2.8
6	3	0	28	1	3.13	0.71	11	2.8
2	1	1	17	1	1.42	3.31	11.5	2.8
11	1.38	1	19	1	3.65	3.26	11	2.8
12	1.5	1	20	0	3.31	3.29	11.46	2.8
11	1.57	1	21	0	3.38	3.23	11.5	2.8
12	1.5	1	22	0	2.39	1.51	11.5	2.8
57	4.38	1	23	1	4.27	1.35	8.08	1.9
69	4.6	1	24	1	3.99	1.29	8.67	1.9
58	4.14	1	25	1	3.44	1.04	8.38	1.9
59	4.21	1	26	1	3.77	0.85	8.04	1.9
34	2.83	1	27	1	3.93	1.22	8.67	1.9
69	4.6	1	24	1	3.99	0.79	11.5	2.8
58	4.14	1	25	1	3.44	0.74	11.04	2.8
59	4.21	1	26	1	3.77	0.83	11	2.8
34	2.83	1	27	1	3.93	0.71	11	2.8
8	1	1	19	1	2.56	1.73	7.17	5.83
8	1	1	20	1	2.69	1.2	6.71	5.83
7	1	1	21	1	2.8	1.07	6.83	5.83
7	1	1	23	1	3.12	1.25	7.5	5.83
7	1	1	24	1	3.16	0.99	8	5.83
22	1.47	1	25	1	3.46	0.76	8	5.83
23	1.53	1	26	1	3.84	0.59	8	5.83
24	1.41	1	27	1	4.06	1.81	7.13	5.83
7	1	1	18	0	3.03	6.07	9.5	2.65
8	1	1	19	1	2.56	5.98	9.5	2.65
8	1	1	20	1	2.69	5.6	8.92	2.65
7	1	1	21	1	2.8	4.94	8.5	2.65
6	1	1	22	1	2.9	4.69	7.67	2.65
7	1	1	23	1	3.12	2.56	7.5	2.65
7	1	1	24	1	3.16	2.39	8	2.65
22	1.47	1	25	1	3.46	2	8	2.65
23	1.53	1	26	1	3.84	1.61	8	2.65
24	1.41	1	27	1	4.06	1.58	8.38	2.65
22	1.47	1	25	1	3.46	0.31	10	2.26
23	1.53	1	26	1	3.84	0.22	10	2.26
24	1.41	1	27	1	4.06	0.43	10	2.26
7	1	1	18	0	3.03	4.37	11.5	5.28
8	1	1	19	1	2.56	4.06	11.38	5.28
8	1	1	20	1	2.69	3.55	8.96	5.28
7	1	1	21	1	2.8	2.81	8	5.28
6	1	1	22	1	2.9	2.81	8	5.28
7	1	1	23	1	3.12	2.25	8	5.28
7	1	1	24	1	3.16	2.26	8.46	5.28
22	1.47	1	25	1	3.46	2.48	9.29	5.28
23	1.53	1	26	1	3.84	1.98	10.5	5.28
24	1.41	1	27	1	4.06	1.18	10.5	5.28

3	1.5	0	24	1	0.58	0.64	8.08	0.84
3	1.5	0	25	1	0.74	0.61	8.5	0.84
15	1.25	0	26	1	0.81	0.44	8.5	0.84
22	1.47	1	25	1	3.46	1.04	8.38	1.9
23	1.53	1	26	1	3.84	0.85	8.04	1.9
24	1.41	1	27	1	4.06	1.22	8.67	1.9
1	1	1	17	1	1.23	3.31	11.5	2.8
1	1	1	18	1	1.2	3.25	11.5	2.8
1	1	1	19	1	1.15	3.26	11	2.8
1	1	1	20	1	1.39	3.29	11.46	2.8
1	1	1	21	1	1	3.23	11.5	2.8
1	1	1	22	1	0.61	1.51	11.5	2.8
1	1	1	23	1	0.62	0.79	11.5	2.8
1	1	1	24	0	0.78	0.74	11.04	2.8
1	1	1	25	0	0.4	0.83	11	2.8
1	1	1	26	1	0.42	0.71	11	2.8
7	1	1	18	0	3.03	0.63	8.88	1.45
8	1	1	19	1	2.56	0.65	7.54	1.45
8	1	1	20	1	2.69	0.64	7.33	1.45
7	1	1	21	1	2.8	0.61	7.5	1.45
6	1	1	22	1	2.9	0.66	8.5	1.45
7	1	1	23	1	3.12	0.59	7.42	1.45
7	1	1	24	1	3.16	0.56	7.71	1.45
22	1.47	1	25	1	3.46	0.44	9	1.45
23	1.53	1	26	1	3.84	0.23	8.29	1.45
24	1.41	1	27	1	4.06	0.1	8.88	1.45
24	1.41	1	27	1	4.06	0.44	8.5	0.84
5	1	1	26	0	8.77	0.85	8.04	1.9
6	1.2	1	27	0	9.55	1.22	8.67	1.9
1	1	1	19	0	6.74	5.98	9.5	2.65
1	1	1	20	0	6.89	5.6	8.92	2.65
2	1	1	21	0	7.93	4.94	8.5	2.65
5	1	1	26	0	8.77	3.84	7.75	4.43
6	1.2	1	27	0	9.55	2.64	8.5	4.43
3	3	1	17	0	0.77	6.07	9.5	2.65
4	4	1	18	0	0.73	5.98	9.5	2.65
4	4	1	19	0	0.89	5.6	8.92	2.65
4	4	1	20	0	0.7	4.94	8.5	2.65
3	3	1	21	0	0.59	4.69	7.67	2.65
1	1	1	18	1	6.38	2.03	8.5	1.9
1	1	1	19	1	4.91	2.12	8.5	1.9
1	1	1	20	1	3.94	2.06	7.71	1.9
1	1	1	21	1	3.35	1.96	7.5	1.9
1	1	1	22	1	3.31	1.33	7.5	1.9
3	1	1	23	1	2.7	1.35	8.08	1.9
3	1	1	24	1	2.96	1.29	8.67	1.9
3	1	1	25	1	2.57	1.04	8.38	1.9
4	1	1	26	1	2.12	0.85	8.04	1.9
4	1	1	27	1	1.96	1.22	8.67	1.9
3	1	1	23	1	2.7	0.59	7.42	1.45
3	1	1	24	1	2.96	0.56	7.71	1.45

3	1	1	25	1	2.57	0.44	9	1.45
4	1	1	26	1	2.12	0.23	8.29	1.45
4	1	1	27	1	1.96	0.1	8.88	1.45
4	1	1	26	1	2.12	0.61	8.08	1.42
4	1	1	27	1	1.96	1.06	8.5	1.42
1	1	1	49	0	0.2	1.98	10	2.26
1	1	1	50	0	0.17	1.59	10	2.26
13	4.33	1	24	0	3.72	1.19	8.83	2.33
6	1.2	0	23	1	0.98	0.59	7.42	1.45
7	1.17	0	24	1	0.69	0.56	7.71	1.45
7	1.17	0	25	1	0.96	0.44	9	1.45
7	1.17	0	26	0	0.7	0.23	8.29	1.45
19	1.9	0	27	0	1.31	0.1	8.88	1.45
1	1	0	22	1	1.97	0.74	11.04	2.8
1	1	0	23	1	1.89	0.83	11	2.8
1	1	0	24	0	1.61	0.71	11	2.8
5	1.67	1	28	1	1.16	1.42	9.5	4.65
5	1.67	1	29	1	1.62	0.9	10.38	4.65
1	1	1	12	0	3.66	2.06	7.71	1.9
4	1	1	13	0	4.51	1.96	7.5	1.9
4	1	1	14	1	4.64	1.33	7.5	1.9
2	1	1	23	1	4.07	4.69	7.67	2.65
6	3	1	26	1	3.03	2.62	6.83	3.1
11	2.75	1	27	0	3.85	0.23	9	4.17
9	2.25	1	28	0	3.53	0.21	9	4.17
14	2.33	1	25	1	2.6	0.31	10	2.26
13	2.17	1	26	1	2.78	0.22	10	2.26
13	2.17	1	27	1	3.49	0.43	10	2.26
1	1	1	10	0	0.71	2.16	10	2.26
1	1	1	11	0	0.52	2.18	10	2.26
1	1	1	12	0	0.79	2.22	10	2.26
1	1	1	13	0	0.97	1.98	10	2.26
1	1	1	14	1	1	1.98	10	2.26
1	1	1	15	1	1.06	1.59	10	2.26
1	1	1	16	1	1.12	0.43	10	2.26
1	1	1	17	1	1.28	0.31	10	2.26
1	1	1	18	0	1.67	0.22	10	2.26
1	1	1	19	0	1.43	0.43	10	2.26
5	1	0	17	1	0.9	0.79	11.5	2.8
12	1.5	0	18	1	1.31	0.74	11.04	2.8
9	1.13	0	19	0	1.39	0.83	11	2.8
9	1.13	0	20	0	1.12	0.71	11	2.8
5	1	0	17	1	0.9	1.29	8.67	1.9
12	1.5	0	18	1	1.31	1.04	8.38	1.9
9	1.13	0	19	0	1.39	0.85	8.04	1.9
9	1.13	0	20	0	1.12	1.22	8.67	1.9
3	1	0	11	1	2.17	3.23	11.5	2.8
3	1	0	12	1	1.45	1.51	11.5	2.8
3	1	0	13	1	1.68	0.79	11.5	2.8
3	1	0	14	1	1.43	0.74	11.04	2.8
3	1	0	15	1	0.99	0.83	11	2.8

3	1.5	0	17	1	0.23	1.46	9.75	2.35
3	1.5	0	18	1	0.59	1.6	10.5	2.35
3	1	1	20	1	1.81	2.62	6.83	3.1
4	1.33	1	21	1	2.12	2.85	7.5	3.1
3	1	1	22	1	1.68	2.86	7.88	3.1
6	1.2	0	11	0	2.68	0.71	7	0.84
7	1.17	0	12	0	1.93	0.49	7	0.84
11	1.38	0	17	1	1.42	0.61	8.5	0.84
13	1.44	0	18	1	1.48	0.44	8.5	0.84
6	1.2	0	11	0	2.68	3.51	8.5	2.33
7	1.17	0	12	0	1.93	3.41	8.5	2.33
4	1	1	16	1	3.53	0.22	10	2.26
4	1	1	17	1	3.59	0.43	10	2.26
4	1	1	16	1	3.53	0.73	7.04	2.52
4	1	1	17	1	3.59	0.78	7.17	2.52
3	1.5	1	8	1	0.61	0.65	7.54	1.45
4	1.33	1	9	1	0.59	0.64	7.33	1.45
4	1.33	1	10	1	0.65	0.61	7.5	1.45
4	1.33	1	11	1	0.61	0.66	8.5	1.45
4	1.33	1	12	1	0.67	0.59	7.42	1.45
4	1.33	1	13	1	0.61	0.56	7.71	1.45
6	2	1	14	1	0.64	0.44	9	1.45
5	1.67	1	15	1	0.56	0.23	8.29	1.45
4	1.33	1	16	1	0.41	0.1	8.88	1.45
1	1	0	10	1	1.12	0.33	7	1.42
1	1	0	11	1	1.09	0.37	7.17	1.42
4	2	0	12	1	3.53	0.43	8	1.42
4	2	0	13	1	2.35	0.44	8	1.42
5	1.67	0	14	1	2.98	0.49	8	1.42
1	1	0	16	1	1.96	1.06	8.5	1.42
2	1	1	3	1	1.6	0.63	8.88	1.45
2	1	1	4	1	1.97	0.65	7.54	1.45
2	1	1	5	1	2.22	0.64	7.33	1.45
2	1	1	6	1	2.32	0.61	7.5	1.45
2	1	1	7	1	2.37	0.66	8.5	1.45
2	1	1	8	1	2.06	0.59	7.42	1.45
3	1	1	9	1	1.99	0.56	7.71	1.45
3	1.5	1	10	1	2.01	0.44	9	1.45
6	1.5	1	11	0	1.89	0.23	8.29	1.45
6	1.5	1	12	0	2.23	0.1	8.88	1.45
2	1	1	6	0	0.51	2.91	7.5	2.33
2	1	1	7	0	0.21	3.15	8	2.33
3	1	1	8	0	0.21	3.51	8.5	2.33
3	1	1	9	0	0.31	3.41	8.5	2.33
2	1	1	10	0	0.35	3.49	7.88	2.33
5	1.25	1	11	0	0.6	0.43	8	1.42
5	1.25	1	12	0	0.69	0.44	8	1.42
5	1.25	1	13	0	0.7	0.49	8	1.42
6	1.5	1	14	0	0.5	0.61	8.08	1.42
18	1.8	1	15	0	0.7	1.06	8.5	1.42
6	1.5	1	12	0	2.23	0.71	11	2.8

18	1.8	1	15	0	0.7	0.71	11	2.8
18	1.8	1	15	0	0.7	1.22	8.67	1.9
18	1.8	1	15	0	0.7	0.44	8.5	0.84
1	1.89	0	22	0	0.51	1.35	8.08	1.9
1	1.93	0	23	0	0.43	1.29	8.67	1.9
1	1.92	0	24	0	0.39	1.04	8.38	1.9
2	1.87	0	25	0	0.42	0.85	8.04	1.9
2	1.83	0	26	1	0.5	1.22	8.67	1.9
3	1.9	0	10	1	3.96	0.56	7.71	1.45
3.9	1.9	0	11	1	3.79	0.44	9	1.45
6	1.9	0	12	1	3.71	0.23	8.29	1.45
6	1.89	0	13	1	3.98	0.1	8.88	1.45
1	1.95	1	3	0	0.78	1.26	6.5	5.48
1	1.94	1	4	0	1.05	2.09	6.08	5.48
1	1.97	1	5	0	1.03	2.31	6	5.48
2	1.98	1	6	0	0.8	2.15	6	5.48
2	1.98	1	7	0	0.67	1.42	6.04	5.48
2	1.98	1	8	0	0.72	1.36	6.88	5.48
2	1.85	1	9	0	0.77	1.14	7	5.48
2	1.85	1	10	0	0.69	1.05	7.79	5.48
2	1.98	1	6	0	0.8	0.61	7.5	1.45
2	1.98	1	7	0	0.67	0.66	8.5	1.45
2	1.98	1	8	0	0.72	0.59	7.42	1.45
2	1.85	1	9	0	0.77	0.56	7.71	1.45
2	1.85	1	10	0	0.69	0.44	9	1.45
22	3.67	1	21	1	3.01	0.44	8.5	0.84
4	1.84	0	25	0	0.7	0.1	8.88	1.45
6	2	0	17	1	1.13	0.71	11	2.8
1	1.61	0	15	1	1.24	5.88	7.25	3.31
1	1.61	0	16	1	1.01	5.55	6.04	3.31
1	1.61	0	17	1	1.17	2.44	6	3.31
2	2.4	1	21	1	0.71	2	8	2.65
4	2.42	1	23	1	0.69	1.58	8.38	2.65
6	1.2	1	30	1	13.7	2.56	7.5	2.65
6	1.2	1	32	1	13.76	2	8	2.65
6	1.2	1	33	1	12.53	1.61	8	2.65
6	1.2	1	34	1	11.76	1.58	8.38	2.65
6	2	0	9	1	0.5	0.71	7	0.84
6	2	0	10	1	0.43	0.49	7	0.84
7	2.33	0	13	0	0.58	0.54	8	0.84
7	2.33	0	14	0	0.61	0.64	8.08	0.84
7	2.33	0	15	0	0.75	0.61	8.5	0.84
7	2.33	0	16	0	0.8	0.44	8.5	0.84
1	1.91	1	17	1	1.33	2.25	8	5.28
2	1.92	1	18	1	1.37	2.26	8.46	5.28
2	1.92	1	19	1	1.57	2.48	9.29	5.28
2	1.91	1	20	1	1.88	1.98	10.5	5.28
2	1.91	1	21	1	2.04	1.18	10.5	5.28
2	1.92	1	18	1	1.37	1.29	8.67	1.9
2	1.92	1	19	1	1.57	1.04	8.38	1.9
2	1.91	1	20	1	1.88	0.85	8.04	1.9

2	1.91	1	21	1	2.04	1.22	8.67	1.9
4	1	1	6	1	5.9	0.64	7.33	1.45
20	1.33	1	26	1	15.4	6.07	9.5	2.65
19	1.27	1	27	1	15.18	5.98	9.5	2.65
16	1.14	1	28	1	14.59	5.6	8.92	2.65
19	1.12	1	29	1	13.84	4.94	8.5	2.65
18	1.13	1	30	1	12.46	4.69	7.67	2.65
20	1.18	1	31	1	11.41	2.56	7.5	2.65
21	1.17	1	32	1	11.25	2.39	8	2.65
21	1.17	1	33	1	11.26	2	8	2.65
21	1.17	1	34	1	10.88	1.61	8	2.65
22	1.16	1	35	1	10.25	1.58	8.38	2.65
20	1.33	1	26	1	15.4	3.79	7.58	1.79
19	1.27	1	27	1	15.18	3.55	7.5	1.79
16	1.14	1	28	1	14.59	2.91	7.5	1.79
19	1.12	1	29	1	13.84	2.43	7.5	1.79
18	1.13	1	30	1	12.46	2.33	7.63	1.79
20	1.18	1	31	1	11.41	2.33	6.54	1.79
21	1.17	1	32	1	11.25	2.55	6.5	1.79
21	1.17	1	33	1	11.26	1.99	7.33	1.79
21	1.17	1	34	1	10.88	1.92	8	1.79
22	1.16	1	35	1	10.25	1.59	8.46	1.79
20	1.33	1	26	1	15.4	2.91	7.5	2.33
19	1.27	1	27	1	15.18	3.15	8	2.33
16	1.14	1	28	1	14.59	3.51	8.5	2.33
19	1.12	1	29	1	13.84	3.41	8.5	2.33
18	1.13	1	30	1	12.46	3.49	7.88	2.33
20	1.18	1	31	1	11.41	2.97	7.5	2.33
21	1.17	1	32	1	11.25	2.32	7.71	2.33
21	1.17	1	33	1	11.26	2.07	7.96	2.33
21	1.17	1	34	1	10.88	1.17	7.88	2.33
22	1.16	1	35	1	10.25	1.19	8.83	2.33
20	1.18	1	31	1	11.41	2.44	6	3.31
21	1.17	1	32	1	11.25	2.87	6.5	3.31
21	1.17	1	33	1	11.26	1.92	6.33	3.31
21	1.17	1	34	1	10.88	2.27	6.38	3.31
22	1.16	1	35	1	10.25	2.41	7.5	3.31
20	1.33	1	26	1	15.4	0.63	8.88	1.45
19	1.27	1	27	1	15.18	0.65	7.54	1.45
16	1.14	1	28	1	14.59	0.64	7.33	1.45
19	1.12	1	29	1	13.84	0.61	7.5	1.45
18	1.13	1	30	1	12.46	0.66	8.5	1.45
20	1.18	1	31	1	11.41	0.59	7.42	1.45
21	1.17	1	32	1	11.25	0.56	7.71	1.45
21	1.17	1	33	1	11.26	0.44	9	1.45
21	1.17	1	34	1	10.88	0.23	8.29	1.45
22	1.16	1	35	1	10.25	0.1	8.88	1.45
3	1	0	17	1	0.66	1.17	7.88	2.33
3	1	0	18	1	0.89	1.19	8.83	2.33
12	1.71	0	15	0	0.08	3.23	11.5	2.8
10	1.67	0	16	1	0.12	1.51	11.5	2.8

11	1.83	0	17	1	0.74	0.79	11.5	2.8
11	1.83	0	18	0	0.47	0.74	11.04	2.8
2	1.99	0	10	1	0.69	0.56	7.71	1.45
2	2.01	0	11	0	0.68	0.44	9	1.45
2	2.11	0	12	0	0.91	0.23	8.29	1.45
2	2.12	0	13	0	0.69	0.1	8.88	1.45
9	1.29	1	17	1	1.45	0.83	11	2.8
11	1.22	1	18	1	1.11	0.71	11	2.8
9	1.29	1	17	1	1.45	0.22	10	2.26
11	1.22	1	18	1	1.11	0.43	10	2.26
11	1.22	1	18	1	1.11	1.81	7.13	5.83
5	1.81	1	8	1	1.36	0.51	9	4.17
6	1.86	1	9	1	1.77	0.14	9	4.17
6	1.86	1	10	1	1.74	0.2	9	4.17
7	1.88	1	11	1	1.72	0.23	9	4.17
7	1.95	1	12	1	1.85	0.21	9	4.17
5	1.81	1	8	1	0.58	1.37	6.58	2.86
6	1.86	1	9	1	0.64	1.16	7.58	2.86
6	1.86	1	10	1	0.63	0.99	8	2.86
7	1.88	1	11	1	0.63	1	8	2.86
7	1.95	1	12	1	0.64	1.75	8	2.86
6	1.86	1	9	1	0.63	1.29	8.67	1.9
6	1.86	1	10	1	0.63	1.04	8.38	1.9
7	1.88	1	11	1	0.64	0.85	8.04	1.9
7	1.95	1	12	1	0.63	1.22	8.67	1.9
11	1.22	1	18	0	6.72	1.81	7.13	5.83
7	1.88	1	11	1	0.64	2.85	7.5	3.1
7	1.95	1	12	1	0.63	2.86	7.88	3.1
11	1.22	1	18	0	6.72	2.64	8.5	4.43
11	1.22	1	18	0	6.72	1.75	8	2.86
11	1.38	0	13	1	3.92	0.85	8.04	1.9
7	1.17	0	14	1	4.11	1.22	8.67	1.9
11	1.22	1	18	0	6.72	1.19	8.83	2.33
11	1.22	1	18	0	6.72	0.1	8.88	1.45
8	1.14	1	10	1	3.85	0.64	8.08	0.84
6	1.2	1	11	1	4.01	0.61	8.5	0.84
6	1.2	1	12	1	3.49	0.44	8.5	0.84
8	1.14	1	10	1	3.85	0.76	8	5.83
4	1.33	0	11	1	0.96	5.98	9.5	2.65
3	1	0	12	1	0.97	5.6	8.92	2.65
3	1	0	13	1	0.88	4.94	8.5	2.65
8	2	0	14	1	0.83	4.69	7.67	2.65
9	1.8	0	15	1	0.87	2.56	7.5	2.65
17	1.7	0	19	0	1.08	1.58	8.38	2.65
20	3.33	1	12	1	5.87	1.17	7.88	2.33
29	4.14	1	13	0	5.53	1.19	8.83	2.33
1	1.81	0	10	0	0.71	5.98	9.5	2.65
1	1.99	0	11	0	0.4	5.6	8.92	2.65
2	1.98	0	12	0	0.31	4.94	8.5	2.65
2	1.98	0	13	0	0.28	4.69	7.67	2.65
4	1.99	0	14	1	0.26	2.56	7.5	2.65

4	1.99	0	15	1	0.21	2.39	8	2.65
6	2	0	16	1	0.7	2	8	2.65
20.8	1.99	0	17	1	1.34	1.61	8	2.65
22.4	1.97	0	18	1	1.35	1.58	8.38	2.65
10	2.5	1	7	1	2.36	3.31	11.5	2.8
10	1.67	1	8	1	2.36	3.25	11.5	2.8
21	1.75	1	9	1	2.56	3.26	11	2.8
7	1.4	1	10	1	2.69	3.29	11.46	2.8
10	1.67	1	8	1	2.36	2.12	8.5	1.9
21	1.75	1	9	1	2.56	2.06	7.71	1.9
6	1.5	1	14	1	2.32	1.04	8.38	1.9
5	1.25	1	15	1	2.53	0.85	8.04	1.9
10	1.67	1	8	1	2.36	0.69	7.17	0.84
21	1.75	1	9	1	2.56	0.71	7	0.84
3	1	1	7	1	2.05	5.88	7.25	3.31
4	1.33	1	8	1	2.44	5.55	6.04	3.31
4	1	1	6	1	3.9	3.25	11.5	2.8
6	1	1	7	1	3.84	3.26	11	2.8
5	1	1	8	1	4.32	3.29	11.46	2.8
5	1	1	9	1	4.26	3.23	11.5	2.8
7	1.4	1	10	1	3.81	1.51	11.5	2.8
5	1.25	1	11	1	3.85	0.79	11.5	2.8
7	1.17	1	12	1	3.55	0.74	11.04	2.8
13	1.3	1	13	1	3.66	0.83	11	2.8
13	1.44	1	14	1	3.58	0.71	11	2.8
4	1	1	6	1	3.9	0.65	7.54	1.45
6	1	1	7	1	3.84	0.64	7.33	1.45
5	1	1	8	1	4.32	0.61	7.5	1.45
5	1	1	9	1	4.26	0.66	8.5	1.45
7	1.4	1	10	1	3.81	0.59	7.42	1.45
7	1.17	1	12	1	3.55	0.44	9	1.45
13	1.3	1	13	1	3.66	0.23	8.29	1.45
13	1.44	1	14	1	3.58	0.1	8.88	1.45
15	1.88	1	10	1	1.49	0.63	8.88	1.45
16	2	1	11	1	1.28	0.65	7.54	1.45
17	2.13	1	12	1	1.32	0.64	7.33	1.45
13	1.44	1	13	1	1.13	0.61	7.5	1.45
14	1.4	1	14	1	0.84	0.66	8.5	1.45
14	1.4	1	15	1	1	0.59	7.42	1.45
13	1.3	1	16	1	1.29	0.56	7.71	1.45
22	1.69	1	17	1	1.13	0.44	9	1.45
21	1.75	1	18	1	1.16	0.23	8.29	1.45
18	1.5	1	19	1	1.07	0.1	8.88	1.45
15	1.88	1	10	1	1.49	0.63	8.88	1.45
16	2	1	11	1	1.28	0.65	7.54	1.45
17	2.13	1	12	1	1.32	0.64	7.33	1.45
7.2	2.12	0	31	1	0.55	0.23	8.29	1.45
11.2	2.11	0	32	1	0.55	0.1	8.88	1.45
7.2	2.12	0	31	1	0.55	1.17	7.88	2.33
11.2	2.11	0	32	1	0.55	1.19	8.83	2.33
4	1.99	0	15	1	0.78	5.98	9.5	2.65

4	1.98	0	16	1	1.07	5.6	8.92	2.65
4	2.01	0	17	1	1.02	4.94	8.5	2.65
2	2.02	0	20	1	1.59	2.32	7.71	2.33
1	2.18	0	23	0	1.25	1.19	8.83	2.33
15	1	0	14	1	1.77	9.46	8.92	2.53
18	1	0	15	1	2.25	4.51	8.5	2.53
39	2.17	0	16	1	2.68	3.7	8.5	2.53
32	2.67	0	17	1	2.67	2.66	6.29	2.53
20	1.43	1	6	0	0.36	1.98	10	2.26
20	1.43	1	7	0	0.39	1.98	10	2.26
21	1.4	1	8	0	0.34	1.59	10	2.26
23	1.44	1	9	0	0.31	0.43	10	2.26
36	4.5	0	18	1	2.69	0.56	7.71	1.45
39	4.88	0	19	1	2.39	0.44	9	1.45
46	6.57	0	21	0	2.51	0.1	8.88	1.45
20	1.43	1	6	0	0.36	2.82	9	4.17
20	1.43	1	7	0	0.39	2.86	9	4.17
21	1.4	1	8	0	0.34	0.51	9	4.17
23	1.44	1	9	0	0.31	0.14	9	4.17
7	1.75	1	10	0	0.42	0.2	9	4.17
14	1.4	1	3	1	0.48	3.31	11.5	2.8
18	1.5	1	5	1	0.49	3.26	11	2.8
20	1.43	1	6	0	0.36	3.29	11.46	2.8
20	1.43	1	7	0	0.39	3.23	11.5	2.8
21	1.4	1	8	0	0.34	1.51	11.5	2.8
23	1.44	1	9	0	0.31	0.79	11.5	2.8
21	1.4	1	8	0	0.34	2.44	6	3.31
23	1.44	1	9	0	0.31	2.87	6.5	3.31
10	2	1	11	1	0.57	6.07	9.5	2.65
12	2	1	12	1	0.89	5.98	9.5	2.65
9	1.8	1	17	1	2.09	2.39	8	2.65
12	1.33	1	18	1	1.48	2	8	2.65
17	1.31	1	19	1	1.62	1.61	8	2.65
11	2.2	1	20	1	1.3	1.58	8.38	2.65
12	2	1	12	1	0.89	4.04	9.5	3.8
17	1.31	1	19	1	1.62	2.79	9.42	3.8
10	2	1	11	1	0.57	4.12	6.67	4.43
12	2	1	12	1	0.89	6.01	6.5	4.43
17	1.31	1	19	1	1.62	2.06	7.13	3.23
11	2.2	1	20	1	1.3	2.21	8	3.23
10	2	1	11	1	0.57	1.46	7.5	2.96
12	2	1	12	1	0.89	1.49	7.5	2.96
12	1.33	1	18	1	1.48	0.61	7.67	2.96
17	1.31	1	19	1	1.62	0.59	7	2.96
35	2.19	1	4	1	1.86	1.26	6.5	5.48
36	2.25	1	5	1	3.09	2.09	6.08	5.48
36	2.25	1	6	1	4.91	2.31	6	5.48
32	2.13	1	7	1	4.95	2.15	6	5.48
44	1.91	1	8	1	4.34	1.42	6.04	5.48
42	2.1	1	9	1	4.19	1.36	6.88	5.48
38	1.9	1	10	1	4.48	1.14	7	5.48

37	1.76	1	11	1	4.33	1.05	7.79	5.48
35	1.84	1	12	1	7.51	1.13	8	5.48
44	1.91	1	8	1	4.34	1.14	7	5.83
35	2.19	1	4	1	1.86	2.16	10	2.26
36	2.25	1	5	1	3.09	2.18	10	2.26
36	2.25	1	6	1	4.91	2.22	10	2.26
32	2.13	1	7	1	4.95	1.98	10	2.26
44	1.91	1	8	1	4.34	1.98	10	2.26
42	2.1	1	9	1	4.19	1.59	10	2.26
38	1.9	1	10	1	4.48	0.43	10	2.26
37	1.76	1	11	1	4.33	0.31	10	2.26
35	1.84	1	12	1	7.51	0.22	10	2.26
24	1.71	1	13	1	5.46	0.43	10	2.26
44	1.91	1	8	1	4.34	0.56	6.04	2.52
35	2.19	1	4	1	1.86	3.31	11.5	2.8
36	2.25	1	5	1	3.09	3.25	11.5	2.8
36	2.25	1	6	1	4.91	3.26	11	2.8
32	2.13	1	7	1	4.95	3.29	11.46	2.8
44	1.91	1	8	1	4.34	3.23	11.5	2.8
42	2.1	1	9	1	4.19	1.51	11.5	2.8
38	1.9	1	10	1	4.48	0.79	11.5	2.8
37	1.76	1	11	1	4.33	0.74	11.04	2.8
35	1.84	1	12	1	7.51	0.83	11	2.8
24	1.71	1	13	1	5.46	0.71	11	2.8
32	2.13	1	7	1	4.95	3.29	11.46	2.8
44	1.91	1	8	1	4.34	3.23	11.5	2.8
42	2.1	1	9	1	4.19	1.51	11.5	2.8
38	1.9	1	10	1	4.48	0.79	11.5	2.8
37	1.76	1	11	1	4.33	0.74	11.04	2.8
44	1.91	1	8	1	4.34	2.58	8	3.4
37	1.76	1	11	1	4.33	2.1	7	3.4
35	1.84	1	12	1	7.51	0.79	7	3.4
44	1.91	1	8	1	4.34	0.49	7.17	0.84
42	2.1	1	9	1	4.19	0.57	8	0.84
38	1.9	1	10	1	4.48	0.54	8	0.84
37	1.76	1	11	1	4.33	2.07	7.96	2.33
35	1.84	1	12	1	7.51	1.17	7.88	2.33
32	2.13	1	7	1	4.95	0.61	7.5	1.45
44	1.91	1	8	1	4.34	0.66	8.5	1.45
42	2.1	1	9	1	4.19	0.59	7.42	1.45
38	1.9	1	10	1	4.48	0.56	7.71	1.45
37	1.76	1	11	1	4.33	0.84	10	5.44
35	1.84	1	12	1	7.51	0.66	10	5.44
4	1.33	1	29	1	0.47	1.98	10	2.26
16	1.6	1	30	1	0.61	1.98	10	2.26
17	1.7	1	31	1	0.62	1.59	10	2.26
18	1.8	1	32	1	0.6	0.43	10	2.26
21	2.1	1	33	1	0.6	0.31	10	2.26
19	2.11	1	34	1	0.59	0.22	10	2.26
13	1.63	1	35	1	0.67	0.43	10	2.26
2	1	0	16	1	0.53	0.79	11.5	2.8

4	2	0	17	1	0.62	0.74	11.04	2.8
4	2	0	18	1	1.04	0.83	11	2.8
4	2	0	19	1	1.59	0.71	11	2.8
12	1.71	1	10	1	13.4	4.94	8.5	2.65
15	1.67	1	11	1	12.47	4.69	7.67	2.65
11	1.38	1	12	1	11.79	2.56	7.5	2.65
12	1.5	1	13	1	12.29	2.39	8	2.65
13	1.44	1	14	1	11.42	2	8	2.65
13	1.44	1	15	1	10.74	1.61	8	2.65
13	1.44	1	16	1	10.47	1.58	8.38	2.65
13	1.44	1	14	1	11.42	0.44	9	1.45
13	1.44	1	15	1	10.74	0.23	8.29	1.45
13	1.44	1	16	1	10.47	0.1	8.88	1.45
4	1.33	1	14	1	1.78	0.64	8.08	0.84
4	1.33	1	15	1	1.65	0.61	8.5	0.84
4	1.33	1	16	1	1.34	0.44	8.5	0.84
3	1	1	11	0	1.21	1.17	7.88	2.33
3	1	1	12	0	1.12	1.19	8.83	2.33
2	1	1	17	1	5.61	0.85	8	0.84
2	1	1	18	1	4.99	0.69	7.17	0.84
2	1	1	19	1	5.18	0.71	7	0.84
2	1	1	20	1	5.21	0.49	7	0.84
3	1	1	21	1	5.46	0.49	7.17	0.84
3	1	1	22	1	5.33	0.57	8	0.84
3	1	1	23	1	4.4	0.54	8	0.84
3	1	1	24	1	4.24	0.64	8.08	0.84
5	2.5	1	25	1	4.34	0.61	8.5	0.84
3	1.5	1	26	1	3.09	0.44	8.5	0.84
5	2.5	1	32	1	12.86	1.92	8	1.79
4	2	1	33	1	12.02	1.59	8.46	1.79
7	1	1	8	1	0.12	5.6	8.92	2.65
8	1	1	9	1	0.15	4.94	8.5	2.65
11	1.1	1	10	1	0.21	4.69	7.67	2.65
11	1.1	1	11	1	0.18	2.56	7.5	2.65
11	1.1	1	12	1	0.2	2.39	8	2.65
12	1.2	1	13	1	0.25	2	8	2.65
15	1.15	1	14	1	0.3	1.61	8	2.65
15	1.25	1	15	1	0.35	1.58	8.38	2.65
7	1	1	8	1	0.12	2.22	10	2.26
8	1	1	9	1	0.15	1.98	10	2.26
11	1.1	1	10	1	0.21	1.98	10	2.26
11	1.1	1	11	1	0.18	1.59	10	2.26
11	1.1	1	12	1	0.2	0.43	10	2.26
12	1.2	1	13	1	0.25	0.31	10	2.26
15	1.15	1	14	1	0.3	0.22	10	2.26
15	1.25	1	15	1	0.35	0.43	10	2.26
7	1	1	8	1	0.12	3.26	11	2.8
8	1	1	9	1	0.15	3.29	11.46	2.8
11	1.1	1	10	1	0.21	3.23	11.5	2.8
11	1.1	1	11	1	0.18	1.51	11.5	2.8
11	1.1	1	12	1	0.2	0.79	11.5	2.8

12	1.2	1	13	1	0.25	0.74	11.04	2.8
15	1.15	1	14	1	0.3	0.83	11	2.8
15	1.25	1	15	1	0.35	0.71	11	2.8
3	1.5	0	23	1	1.55	1.17	7.88	2.33
8.8	1.87	1	26	1	1	1.61	8	2.65
8.8	1.87	1	27	1	1.07	1.58	8.38	2.65
8.8	1.87	1	26	1	1	1.98	10.5	5.28
8.8	1.87	1	27	1	1.07	1.18	10.5	5.28
1	1.52	0	15	1	0.12	1.35	8.08	1.9
4	1.51	0	16	1	0.23	1.29	8.67	1.9
4	1.55	0	17	1	0.31	1.04	8.38	1.9
4	1.67	0	19	1	0.44	1.22	8.67	1.9
8.8	1.87	1	26	1	1	0.85	8.04	1.9
8.8	1.87	1	27	1	1.07	1.22	8.67	1.9
6	1.2	0	22	1	3.37	0.44	8.5	0.84
2	1	0	25	1	0.31	1.13	8.08	5.07
1	1.91	0	16	0	0.17	0.84	10	5.44
4	1	0	9	0	0.15	0.74	11.04	2.8
4	1	0	10	1	0.12	0.83	11	2.8
5	1.25	0	11	1	0.08	0.71	11	2.8
3	1.73	0	22	1	0.86	0.44	8.5	0.84
3	1.5	1	22	1	1.06	0.94	8.08	5.48
3	1.5	1	17	1	0.3	1.98	10	2.26
4	2	1	18	0	0.3	1.59	10	2.26
3	1.5	1	19	0	0.26	0.43	10	2.26
4	2	1	20	0	0.33	0.31	10	2.26
5	2.5	1	21	0	0.4	0.22	10	2.26
5	2.5	1	22	0	0.53	0.43	10	2.26
1	1	0	14	1	1.56	0.85	8.04	1.9
2	1	0	15	1	1.55	1.22	8.67	1.9
2	1	0	15	1	1.55	0.43	10	2.26
5	1	0	21	0	1.21	1.19	8.83	2.33
3	1.67	0	21	1	0.74	1.35	9.42	4.65
7.2	1.64	0	22	0	2.16	1.42	9.5	4.65
10	1.67	0	23	0	2.38	0.9	10.38	4.65
38	4.75	0	19	1	0.25	0.22	10	2.26
34	4.25	0	20	1	0.68	0.43	10	2.26
5	1.25	0	26	1	1.18	0.23	8.29	1.45
6	1.2	0	27	1	1.23	0.1	8.88	1.45
13	1.44	0	17	1	0.29	1.19	8.83	2.33
3	1	0	15	0	0.39	2.07	7.96	2.33
3	1	0	16	0	0.37	1.17	7.88	2.33
6	1	0	15	1	0.5	1.99	6.5	3.23
5	1	0	16	1	0.8	2.06	7.13	3.23
6	1	0	17	1	0.95	2.21	8	3.23
6	1	0	17	1	0.95	1.19	8.83	2.33
5.1	1.63	0	17	1	0.51	2.07	7.96	2.33
1	1.68	0	16	1	0.71	1.08	7.67	3.2
1	1.67	0	17	1	0.59	1.7	8	3.2
1	1.64	0	18	1	0.61	0.61	8.5	0.84
2	1	0	9	1	4.39	1.13	8.08	5.07

1	1.38	0	16	1	0.31	2.22	10	2.26
1	1.92	0	17	1	0.3	1.98	10	2.26
2	1.53	0	18	1	0.28	1.98	10	2.26
3	1.53	0	19	1	0.3	1.59	10	2.26
4	1.76	0	22	1	0.21	1.22	8.67	1.9
1	1.87	0	19	0	0.08	1.19	8.83	2.33
4	2	0	16	1	0.3	0.71	11	2.8
7	1.87	0	21	1	0.69	0.83	11	2.8
7.7	1.9	0	22	1	0.75	0.71	11	2.8
5.2	1.7	0	13	1	1.2	1.22	8.67	1.9
7.7	1.87	0	11	1	0.69	0.85	8.04	1.9
6	1.2	0	12	1	0.2	0.71	11	2.8
2	1	0	10	1	0.37	0.31	10	2.26
2	1	0	11	1	0.43	0.22	10	2.26
2	1	0	12	1	0.53	0.43	10	2.26
1	1.93	0	14	1	0.31	1.04	8.38	1.9
1	1.86	0	15	1	0.78	0.85	8.04	1.9
15.4	1.67	0	16	0	0.26	0.56	7.71	1.45
16.5	1.67	0	17	0	0.38	0.44	9	1.45
18.7	1.72	0	18	0	0.31	0.23	8.29	1.45
19.5	1.72	0	19	0	0.3	0.1	8.88	1.45
15.4	1.67	0	16	0	0.26	0.4	6.83	2.52
16.5	1.67	0	17	0	0.38	0.52	7.13	2.52
18.7	1.72	0	18	0	0.31	0.73	7.04	2.52
19.5	1.72	0	19	0	0.3	0.78	7.17	2.52
2	1	0	28	0	2.49	1.75	8	2.86
2	1.58	0	13	1	0.69	0.22	10	2.26
2	1.65	0	14	1	0.45	0.43	10	2.26
2	1	0	12	1	0.46	0.85	6.63	5.31
2	1	0	13	1	0.45	1	7.21	5.31
2	1	0	14	1	0.61	1.82	7.5	5.31
2	1	0	15	1	0.66	2.57	7.5	5.31
5	1	0	16	1	0.69	2.72	7.5	5.31
5	1.25	0	17	1	0.47	2.83	7.5	5.31
4	1	0	15	1	0.9	2.74	8	4.13
7	1.17	0	18	1	0.84	2	8	2.65
5	1	0	19	1	1.03	1.61	8	2.65
5	1	0	20	1	2.14	1.58	8.38	2.65
8	1.14	0	10	1	1.18	0.79	11.5	2.8
13	1.63	0	11	1	1.48	0.74	11.04	2.8
16	2	0	12	1	1.82	0.83	11	2.8
20	2.5	0	13	1	1.68	0.71	11	2.8
8	1.14	0	10	1	1.18	2.32	7.71	2.33
13	1.63	0	11	1	1.48	2.07	7.96	2.33
16	2	0	12	1	1.82	1.17	7.88	2.33
20	2.5	0	13	1	1.68	1.19	8.83	2.33
1	1.95	0	18	0	0.83	0.21	9	4.17
1	1.52	0	21	0	0.56	1.75	8	2.86
8	1.6	0	22	1	0.45	0.44	8.5	0.84
8	1.6	0	22	1	0.45	0.44	8.5	0.84
6	1.99	0	15	0	1.82	1.04	8.38	1.9

7.2	2.1	0	16	0	1	0.85	8.04	1.9
7.8	2.1	0	17	0	1.22	1.22	8.67	1.9
2	1.85	0	18	0	1.44	0.31	10	2.26
2	1.85	0	19	0	2.32	0.22	10	2.26
2	1.85	0	18	0	1.44	0.74	11.04	2.8
2	1.85	0	19	0	2.32	0.83	11	2.8
12	1.33	0	12	1	1.3	4.39	4.5	4.65
12	1.33	0	13	1	0.68	4.72	5.5	4.65
12	1.33	0	14	1	0.61	4.82	6.25	4.65
15	1.67	0	15	1	0.61	6.96	7.5	4.65
13	1.3	0	16	1	0.63	7	7.79	4.65
12	1.33	0	18	1	0.87	0.44	8.5	0.84
6	1.4	0	20	0	0.28	0.74	11.04	2.8
5.2	1.49	0	17	1	0.23	0.31	10	2.26
11.2	1.51	0	18	1	0.55	0.22	10	2.26
11.2	1.49	0	19	1	0.41	0.43	10	2.26
11.2	1.49	0	19	1	0.41	0.71	11	2.8
3	1.5	0	15	0	0.47	0.71	11	2.8
2	1	0	15	0	0.26	0.44	8.5	0.84
1	1.69	0	10	1	0.55	1.14	7	5.48
2	1.71	0	11	1	0.63	1.05	7.79	5.48
2	1.71	0	12	1	0.77	1.13	8	5.48
3	1.73	0	13	1	0.81	0.94	8.08	5.48
3	1.73	0	13	1	0.81	0.44	8.5	0.84
3	1	0	9	0	0.53	1.61	8	2.65
6	1.5	0	10	1	0.46	1.58	8.38	2.65
3	1	0	9	0	0.53	0.85	8.04	1.9
6	1.5	0	10	1	0.46	1.22	8.67	1.9
14	1.75	0	16	1	1.75	0.31	10	2.26
13	1.44	0	17	1	1.4	0.22	10	2.26
17	2.13	0	18	1	1.7	0.1	8.88	1.45
2	1	0	14	1	0.14	2.32	7.71	2.33
2	1	0	15	1	0.13	2.07	7.96	2.33
2	1	0	16	0	0.16	1.17	7.88	2.33
15	2.5	0	14	1	1.01	0.44	8.5	0.84
8	2	0	24	1	2.94	0.31	10	2.26
9	1.8	0	25	1	3.63	0.22	10	2.26
12	1.5	0	26	1	2.59	0.43	10	2.26
3	1	0	15	1	0.25	2.64	8.5	4.43
5	1.67	0	16	0	1.48	1.17	7.88	2.33
2	1	0	12	0	0.74	0.44	8.5	0.84
5.1	1.57	0	10	1	0.46	0.85	8.04	1.9
5.1	1.64	0	11	1	0.81	1.22	8.67	1.9
3	1.7	0	12	1	0.31	0.79	11.5	2.8
3	1.52	0	13	1	0.47	0.74	11.04	2.8
3	1.62	0	14	1	0.51	0.83	11	2.8
33	2.36	0	21	1	1.65	0.1	8.88	1.45
2	1.42	0	14	1	0.25	2.26	8.46	5.28
4	1.56	0	15	1	0.25	2.48	9.29	5.28
41.8	1.54	0	17	0	2.79	1.18	10.5	5.28
41.8	1.54	0	17	0	2.79	1.22	8.67	1.9

3	1.72	0	6	1	0.36	0.43	10	2.26
4	1.88	0	7	0	1.15	1.81	7.13	5.83
2	1.57	0	19	1	0.08	0.1	8.88	1.45
2	1	0	21	1	0.07	0.71	11	2.8
11	1.55	0	13	1	0.09	1.22	8.67	1.9
6	1.95	0	13	1	0.37	1.17	7.88	2.33
6	1.93	0	14	1	0.64	1.19	8.83	2.33

tat	phat	imr	doi	doi_wgi	efi	doi_1_efi	doi_1	IND
0.29	0.664017	0.428591	5.25	2.3625	24.02	10.3658	15.75	S90
0.4	0.713579	0.405731	5.25	2.3625	24.12	10.341	15.75	S90
0.39	0.959277	0.302925	5.335	2.50745	24.13	11.0944	16.335	S90
0.83	0.6017	0.458321	6	1.2	22.85	3.707	11	E50
0.68	1.04337	0.271817	7.31	1.1696	23.05	2.5992	11.31	E50
0.83	1.12867	0.242396	6	1.2	22.85	3.707	11	E50
0.68	1.49817	0.139206	7.31	1.1696	23.05	2.5992	11.31	E50
0.83	0.485197	0.516796	6	1.2	22.85	3.707	11	E50
0.68	0.59596	0.461114	7.31	1.1696	23.05	2.5992	11.31	E50
0.91	0.658216	0.431312	2.5	0.25	22.61	1.0445	2.5	C36
3.31	0.409066	0.556994	4.57	0.9597	23.38	3.36105	8.57	F51
2.09	0.511082	0.503483	5.215	1.7731	23.66	7.0329	13.715	F51
1.82	0.542724	0.487456	5.75	3.1625	23.8	13.948	19.25	F51
1.74	0.845137	0.348488	6.835	3.21245	23.88	13.9637	23.335	F51
0.55	0.409127	0.556961	5.7	0.798	23.61	2.1861	7.7	E48
0.43	0.63142	0.444006	6.18	0.8652	23.71	2.1588	8.18	E48
0.42	0.845262	0.348436	6.18	0.8652	23.66	2.1826	8.18	E48
0.58	0.94461	0.308565	6.225	0.93375	23.64	2.41125	8.725	E48
0.55	1.03349	0.275363	7.425	1.3365	23.62	3.6279	12.925	E48
0.53	1.3019	0.189199	7.385	1.3293	23.81	3.5541	12.385	E48
0.7	-0.13065	0.882879	4.75	0.7125	23.57	2.14725	6.75	E48
0.55	0.057482	0.761654	5.22	0.7308	23.53	2.0937	7.22	E48
0.55	0.157933	0.700136	5.7	0.798	23.61	2.1861	7.7	E48
0.43	0.349432	0.589545	6.18	0.8652	23.71	2.1588	8.18	E48
0.42	0.503739	0.507241	6.18	0.8652	23.66	2.1826	8.18	E48
0.58	0.566884	0.475404	6.225	0.93375	23.64	2.41125	8.725	E48
0.55	0.655618	0.432534	7.425	1.3365	23.62	3.6279	12.925	E48
0.53	0.750736	0.389056	7.385	1.3293	23.81	3.5541	12.385	E48
0.7	-0.4125	1.0777	4.75	0.7125	23.57	2.14725	6.75	E48
0.55	-0.09848	0.86162	5.22	0.7308	23.53	2.0937	7.22	E48
0.55	0.016621	0.787333	5.7	0.798	23.61	2.1861	7.7	E48
0.43	0.221231	0.662586	6.18	0.8652	23.71	2.1588	8.18	E48
0.42	0.457364	0.531313	6.18	0.8652	23.66	2.1826	8.18	E48
0.58	0.635344	0.442134	6.225	0.93375	23.64	2.41125	8.725	E48
0.55	0.780933	0.375797	7.425	1.3365	23.62	3.6279	12.925	E48
0.53	1.0327	0.275647	7.385	1.3293	23.81	3.5541	12.385	E48
0.7	-0.81247	1.37708	4.75	0.7125	23.57	2.14725	6.75	E48
0.55	-0.67101	1.26847	5.22	0.7308	23.53	2.0937	7.22	E48
0.55	-0.54296	1.17265	5.7	0.798	23.61	2.1861	7.7	E48
0.43	-0.39016	1.06173	6.18	0.8652	23.71	2.1588	8.18	E48
0.42	-0.1808	0.916439	6.18	0.8652	23.66	2.1826	8.18	E48
0.58	0.049319	0.766755	6.225	0.93375	23.64	2.41125	8.725	E48
0.55	0.197264	0.67669	7.425	1.3365	23.62	3.6279	12.925	E48
0.53	0.354325	0.58684	7.385	1.3293	23.81	3.5541	12.385	E48
3.43	0.036392	0.774862	2	0.2	22.62	1.1	2	C39
2.19	0.20215	0.673803	2.5	0.25	23.06	1.1545	2.5	C39
1.26	0.486398	0.516174	3.665	0.47645	23.34	1.74785	4.665	C39
1.17	0.611818	0.453419	4.94	0.9386	23.39	3.16635	8.44	C39
1.15	0.774985	0.378388	4.815	0.7704	23.18	2.5112	7.315	C39
1	0.917918	0.318992	5.85	0.9945	23.53	2.99455	9.35	C39

1.6	0.489256	0.514697	3.665	0.47645	23.04	1.7446	4.665	C39
1.17	0.582659	0.467622	4.815	0.7704	23.39	2.5064	7.315	C39
1.07	0.654082	0.433257	3.75	0.5625	23.39	2.06175	5.25	C39
1.15	0.761368	0.384357	4.75	0.7125	23.18	2.27925	6.75	C39
1	0.926013	0.315807	5.7	0.798	23.53	2.2561	7.7	C39
1	0.937593	0.311286	6	1.2	23.47	3.842	11	C39
0.77	1.24026	0.207131	5.65	0.7345	23.64	2.03645	7.15	C39
1	0.932803	0.313151	5.75	0.8625	23.53	2.49225	8.25	C39
1	1.08392	0.257564	5.9	1.062	23.47	3.2778	9.9	C39
0.77	1.2585	0.20171	5.7	0.798	23.64	2.2631	7.7	C39
0.22	1.12247	0.244465	2.75	0.4125	22.82	1.50225	3.75	N77
0.21	1.29162	0.192114	3.5	0.7	22.9	2.776	6	N77
0.65	1.23875	0.207585	15.56	7.9356	24.58	38.4438	69.06	N77
1.13	-0.27199	0.978718	9.135	3.01455	24.71	11.1293	26.135	C33
1.08	-0.02524	0.814023	9.595	3.0704	24.89	11.1392	27.095	C33
0.85	0.203993	0.672717	9.3	3.348	24.87	13.0788	28.8	C33
0.85	0.342951	0.593138	10.38	3.9444	25.01	15.7054	33.88	C33
0.87	0.486997	0.515864	6.165	2.03445	25.2	7.8672	16.665	C33
0.6	0.53943	0.489112	6.75	2.3625	25.39	9.35375	19.25	C33
0.44	0.570751	0.47349	10.855	5.10185	25.55	22.9501	42.355	C33
0.59	0.691083	0.41602	10.655	5.64715	25.6	27.2499	45.155	C33
0.64	0.848262	0.34719	8.31	2.9916	25.79	11.5974	25.31	C33
0.51	1.10662	0.249797	8.31	2.9916	25.87	12.0312	25.31	C33
0.6	0.017684	0.786661	6.75	2.3625	25.39	9.35375	19.25	C33
0.44	0.065361	0.756746	10.855	5.10185	25.55	22.9501	42.355	C33
0.59	0.223921	0.661011	10.655	5.64715	25.6	27.2499	45.155	C33
0.51	-0.30815	1.00385	1.75	0.2625	21.23	1.4985	2.25	C39
0.41	-0.10586	0.866479	1.75	0.2625	21.32	1.4985	2.25	C39
0.45	-0.05988	0.836393	1.75	0.2625	21.35	1.5	2.25	C39
0.47	0.028766	0.779662	1.75	0.2625	21.77	1.50075	2.25	C39
0.42	0.111309	0.7284	1.75	0.2625	21.76	1.437	2.25	C39
0.42	0.171025	0.69229	1.75	0.2625	21.81	1.44375	2.25	C39
0.34	0.295116	0.619989	1.75	0.2625	22.14	1.4505	2.25	C39
0.35	0.415673	0.553444	1.75	0.2625	22.16	1.4355	2.25	C39
0.29	0.5489	0.48436	1.75	0.2625	22.25	1.4715	2.25	C39
0.47	0.696096	0.413715	1.75	0.2625	22.23	1.58625	2.25	C39
0.12	-0.43204	1.09175	10.165	17.5855	25.5	62.0637	34.665	J69
0.15	0.123255	0.72111	8.75	10.0625	25.85	51.382	40.25	J69
0.09	-0.19047	0.922962	12.9	26.832	25.96	131.914	62.4	J69
0.06	0.163853	0.696583	11.645	18.9813	26.08	110.098	65.145	J69
0.06	0.451743	0.53427	11.5	17.25	25.9	105.855	67.5	J69
0.06	-0.11302	0.871197	11.5	17.25	25.9	105.855	67.5	J69
1.68	0.401703	0.560963	1.75	0.2625	21.94	1.38375	2.25	C38
1.59	0.594336	0.461906	2.165	0.28145	22.27	1.28505	2.665	C38
2.07	0.848068	0.34727	2.165	0.28145	21.95	1.326	2.665	C38
0.48	0.036392	0.774862	2.5	0.25	22.8	1.0805	2.5	C39
0.57	0.20215	0.673803	2.5	0.25	22.81	1.0715	2.5	C39
0.55	0.370265	0.578068	2.5	0.25	22.78	1.072	2.5	C39
0.57	0.50539	0.506395	2.5	0.25	22.8	1.077	2.5	C39
0.79	0.656131	0.432293	3.1	0.372	23.48	1.4352	3.6	C39
0.56	0.786242	0.373493	3	0.3	23.8	1.1455	3	C39

0.49	0.812968	0.362021	3	0.3	23.8	1.1505	3	C39
0.54	0.962231	0.301797	3	0.3	24.11	1.1475	3	C39
0.64	1.11503	0.246958	3	0.3	24.82	1.034	3	C39
0.48	1.30317	0.188839	3.5	0.35	24.9	1.0755	3.5	C39
0.79	0.799809	0.367643	3.1	0.372	23.48	1.4352	3.6	C39
0.56	0.773214	0.379161	3	0.3	23.8	1.1455	3	C39
0.49	0.851782	0.34573	3	0.3	23.8	1.1505	3	C39
0.54	0.964455	0.30095	3	0.3	24.11	1.1475	3	C39
0.64	1.13456	0.240444	3	0.3	24.82	1.034	3	C39
0.48	1.28383	0.194344	3.5	0.35	24.9	1.0755	3.5	C39
0.48	1.36649	0.171567	3.5	0.35	24.9	1.0755	3.5	C39
0.27	1.56405	0.124761	2.5	0.25	23.15	1.024	2.5	C33
1.14	1.07326	0.261264	3.3	0.528	23.73	2.1296	4.8	C32
0.91	0.10396	0.732901	2.625	0.34125	25.17	1.57625	3.125	C39
0.9	0.626138	0.446532	2.5	0.25	25.4	1.1335	2.5	C39
0.78	0.338272	0.595739	2.5	0.25	25.42	1.1335	2.5	C39
0.72	0.438788	0.541117	5.055	0.55605	25.35	1.57685	5.555	C39
0.78	0.603079	0.457651	5.55	0.6105	25.43	1.68575	6.05	C39
0.87	0.653904	0.433341	3.1	0.372	25.55	1.4724	3.6	C39
0.77	0.839329	0.350909	3.585	0.4302	25.67	1.5312	4.085	C39
0.77	1.01132	0.283426	4.625	0.60125	25.69	1.7563	5.625	C39
0.63	1.23178	0.209683	4.625	0.60125	25.59	1.93375	5.625	C39
0.68	1.40747	0.160987	9.585	1.1502	25.67	2.5008	11.085	C39
0.91	-0.04425	0.826268	2.625	0.34125	25.17	1.57625	3.125	C39
0.72	0.352893	0.587631	5.055	0.55605	25.35	1.52845	5.555	C39
0.78	0.442066	0.53938	5.55	0.6105	25.43	1.58345	6.05	C39
0.68	1.35383	0.174931	9.585	1.1502	25.67	2.4408	11.085	C39
0.84	-1.04071	1.55784	1	0.1	21.39	0.9795	1	E48
1.22	-0.90964	1.45325	1	0.1	21.57	0.9595	1	E48
0.6	-0.46817	1.11788	1	0.1	21.88	1.0125	1	E48
0.8	-0.44276	1.09948	2.625	0.34125	22.11	1.44365	3.125	E48
0.82	0.851718	0.345757	5.055	0.55605	23.16	1.44375	5.555	E48
0.87	0.845463	0.348353	5.55	0.6105	23.16	1.4905	6.05	E48
0.84	0.990323	0.291197	5.6	0.672	23.41	1.6938	6.6	E48
0.8	0.218661	0.664092	2.625	0.34125	22.11	1.44365	3.125	E48
0.67	0.403346	0.560076	2.165	0.28145	22.32	1.37345	2.665	E48
0.74	0.654722	0.432956	2.625	0.34125	22.61	1.29415	3.125	E48
0.93	0.831963	0.353994	4.07	0.4477	23.22	1.33815	4.57	E48
0.82	1.00126	0.287135	5.055	0.55605	23.16	1.44375	5.555	E48
0.87	1.18659	0.223638	5.55	0.6105	23.16	1.4905	6.05	E48
0.84	1.43763	0.153498	5.6	0.672	23.41	1.6938	6.6	E48
0.8	-0.09215	0.857462	2.625	0.34125	22.11	1.44365	3.125	E48
0.67	0.022952	0.783331	2.165	0.28145	22.32	1.37345	2.665	E48
0.74	0.227561	0.658884	2.625	0.34125	22.61	1.29415	3.125	E48
0.93	0.476355	0.521386	4.07	0.4477	23.22	1.33815	4.57	E48
0.82	0.660666	0.430162	5.055	0.55605	23.16	1.44375	5.555	E48
0.87	0.825246	0.35682	5.55	0.6105	23.16	1.4905	6.05	E48
0.84	1.07069	0.262163	5.6	0.672	23.41	1.6938	6.6	E48
0.93	-0.32671	1.01684	4.07	0.4477	23.22	1.33815	4.57	E48
0.82	-0.19041	0.922922	5.055	0.55605	23.16	1.44375	5.555	E48
0.87	-0.04704	0.828068	5.55	0.6105	23.16	1.4905	6.05	E48

0.84	0.168979	0.693513	5.6	0.672	23.41	1.6938	6.6	E48
0.82	0.614813	0.451974	5.055	0.55605	23.16	1.44375	5.555	E48
0.87	0.794185	0.370062	5.55	0.6105	23.16	1.4905	6.05	E48
0.84	0.96982	0.298911	5.6	0.672	23.41	1.6938	6.6	E48
0.87	0.558919	0.47936	5.55	0.6105	23.16	1.4905	6.05	E48
0.84	0.798662	0.368136	5.6	0.672	23.41	1.6938	6.6	E48
1.24	0.213542	0.667095	16.515	4.9545	24.7	16.2795	47.015	C39
0.95	0.341019	0.594212	16.19	5.5046	25.03	19.6401	50.69	C39
0.9	0.396782	0.563623	16.26	5.691	25.1	20.6552	52.76	C39
1.18	0.390838	0.566846	16.145	6.94235	25.25	29.2443	62.145	C39
1.02	0.44388	0.53842	17.66	7.5938	25.44	32.1124	69.16	C39
0.82	0.447362	0.536581	7.81	2.0306	25.71	6.3778	18.31	C39
0.72	0.664792	0.428228	7.615	1.6753	25.8	4.9379	15.615	C39
0.64	0.7316	0.397594	8.5	2.55	25.98	8.6835	22.5	C39
0.42	0.830059	0.354793	8.645	2.85285	25.83	10.2119	24.645	C39
0.69	0.437614	0.54174	3.5	0.7	24.99	2.995	6	C35
0.6	0.512369	0.502826	3.4	0.612	25.21	2.5209	5.4	C35
0.43	0.550737	0.483441	4.94	0.9386	25.22	3.1882	8.44	C35
0.28	0.635431	0.442093	5.22	0.7308	25.26	2.1035	7.22	C35
0.22	0.493314	0.512602	5.5	1.1	25.26	3.496	10	C35
0.22	0.698084	0.412803	5.39	0.9702	25.21	3.0618	8.89	C35
0.24	0.802874	0.366329	5.5	1.1	25.14	3.602	10	C35
0.33	0.945646	0.308165	4.285	0.6856	25.26	2.3216	6.285	C35
0.43	0.603235	0.457575	4.94	0.9386	25.22	3.0932	8.44	C35
0.28	0.795119	0.36966	5.22	0.7308	25.26	2.1665	7.22	C35
0.22	0.71788	0.403781	5.5	1.1	25.26	3.595	10	C35
0.22	0.809109	0.363664	5.39	0.9702	25.21	3.0555	8.89	C35
0.24	0.909121	0.322474	5.5	1.1	25.14	3.666	10	C35
0.47	1.26647	0.199372	5.7	0.798	25.25	2.2862	7.7	C35
0.44	0.320339	0.605758	2	0.2	21.82	1.08	2	F51
0.34	0.427186	0.547287	2.5	0.25	21.82	1.13	2.5	F51
0.39	0.591477	0.463302	2.5	0.25	21.81	1.148	2.5	F51
0.22	0.648632	0.435829	2	0.2	21.8	1.069	2	F51
0.17	0.834057	0.353115	2	0.2	21.86	1.068	2	F51
0.11	1.01238	0.283038	2	0.2	21.83	1.0755	2	F51
0.18	1.23284	0.209364	2	0.2	21.73	1.129	2	F51
0.15	1.4022	0.162322	1.5	0.15	21.76	1.094	1.5	F51
0.39	0.391244	0.566625	2.5	0.25	21.81	1.098	2.5	F51
0.22	-0.36882	1.04656	2	0.2	21.8	1.0665	2	F51
0.17	-0.15947	0.902099	2	0.2	21.86	1.068	2	F51
0.11	0.076986	0.749529	2	0.2	21.83	1.0755	2	F51
0.18	0.243922	0.64936	2	0.2	21.73	1.079	2	F51
1.31	0.156077	0.701251	3.335	1.23395	25.3	5.77015	7.335	C38
1.3	0.52413	0.496841	2.535	0.53235	25.51	2.90535	4.535	C38
1.31	-0.82545	1.38719	2.53	0.5313	25.3	3.32115	6.53	C38
1.3	-0.74416	1.32429	2.535	0.53235	25.51	2.90535	4.535	C38
1.31	0.440605	0.540154	2.53	0.5313	25.3	3.42615	6.53	C38
1.3	0.552999	0.482311	2.535	0.53235	25.51	2.9127	4.535	C38
1.06	0.77781	0.377156	1.575	0.3465	25.86	3.4672	5.575	C38
1.15	0.890483	0.329928	3.085	0.6787	26.24	3.5959	6.585	C38
1.02	1.06692	0.26348	2.535	0.53235	26.3	3.0156	5.035	C38

0.98	1.21618	0.214434	2.535	0.53235	26.43	3.12375	5.535	C38
0.75	0.535698	0.490991	6.18	0.8652	24.92	2.4451	8.18	C36
0.8	0.561405	0.478123	6.835	1.16195	25.09	3.42465	10.835	C36
0.75	0.208729	0.669926	6.18	0.8652	24.92	2.4451	8.18	C36
0.8	0.320888	0.60545	6.835	1.16195	25.09	3.42465	10.835	C36
0.8	0.454374	0.532885	3	0.3	25.51	1.225	3	C36
0.63	0.676667	0.42269	3	0.3	25.47	1.224	3	C36
0.67	0.890509	0.329917	3.5	0.35	25.82	1.2685	3.5	C36
0.85	0.983527	0.29374	3.585	0.4302	25.97	1.614	4.085	C36
0.8	1.09773	0.25282	4.565	0.50215	26.05	1.6027	5.065	C36
0.79	1.34082	0.178435	3.75	0.5625	26.19	2.211	5.25	C36
0.8	0.469912	0.524743	6.835	1.16195	25.09	3.42465	10.835	C36
0.1	0.6122	0.453235	3.1	0.372	25.61	1.5174	3.6	L71
0.14	0.807052	0.364542	3.75	0.5625	26.1	2.1735	5.25	L71
0.8	0.191731	0.679966	2.335	0.39695	24.54	1.96095	3.335	C35
0.57	0.341872	0.593738	3.2	0.448	24.62	1.7192	4.2	C35
0.47	0.437375	0.541866	3.665	0.47645	24.61	1.68025	4.665	C35
0.36	0.665999	0.427663	3.585	0.4302	24.48	1.5444	4.085	C35
0.39	0.886171	0.331666	4.07	0.4477	24.51	1.46355	4.57	C35
0.62	0.99185	0.290628	4.07	0.4477	24.63	1.4707	4.57	C35
0.8	1.09972	0.25214	5.55	0.6105	24.84	1.66485	6.05	C35
0.85	1.3618	0.172808	6.585	0.7902	25.07	1.9878	7.585	C35
0.57	0.582389	0.467755	3.2	0.448	24.62	1.7192	4.2	C35
0.47	0.641762	0.439083	3.665	0.47645	24.61	1.68025	4.665	C35
0.36	0.543957	0.486837	3.585	0.4302	24.48	1.5444	4.085	C35
0.39	0.742397	0.392764	4.07	0.4477	24.51	1.46355	4.57	C35
0.62	0.859847	0.342401	4.07	0.4477	24.63	1.4707	4.57	C35
0.8	0.977298	0.296083	5.55	0.6105	24.84	1.66485	6.05	C35
0.85	1.16787	0.229593	6.585	0.7902	25.07	1.9878	7.585	C35
0.8	1.25241	0.203509	5.55	0.6105	24.84	1.66485	6.05	C35
0.85	1.41544	0.158983	6.585	0.7902	25.07	1.9878	7.585	C35
0.8	1.17589	0.227032	5.55	0.6105	24.84	1.66485	6.05	C35
0.85	1.37515	0.169293	6.585	0.7902	25.07	1.9878	7.585	C35
0.8	0.714048	0.405518	5.55	0.6105	24.84	1.66485	6.05	C35
0.85	0.996787	0.288791	6.585	0.7902	25.07	1.9878	7.585	C35
0.42	0.470352	0.524513	2	0.2	24.59	1.0795	2	C22
0.43	0.682126	0.420157	2.5	0.25	24.58	1.059	2.5	C22
0.36	0.842358	0.349645	2.5	0.25	24.76	1.001	2.5	C22
0.3	0.803102	0.366232	3	0.3	25.08	1.073	3	C22
0.28	0.88167	0.333487	3	0.3	25.13	1.0705	3	C22
0.32	0.994343	0.2897	3	0.3	25.38	1.159	3	C22
0.27	1.15179	0.234792	3.1	0.372	25.38	1.4634	3.6	C22
0.3	1.30105	0.189438	3.1	0.372	25.31	1.584	3.6	C22
0.3	0.81613	0.360677	3	0.3	25.08	1.073	3	C22
0.28	0.842856	0.349438	3	0.3	25.13	1.0705	3	C22
0.32	0.992119	0.290528	3	0.3	25.38	1.159	3	C22
0.27	1.13226	0.241207	3.1	0.372	25.38	1.4634	3.6	C22
0.3	1.3204	0.184028	3.1	0.372	25.31	1.584	3.6	C22
1.22	-1.17847	1.66986	1.5	0.3	22.75	1.942	2	C38
1.19	-1.12235	1.62398	1.5	0.3	22.79	1.938	2	C38
1.27	-0.96257	1.49524	1.5	0.3	22.87	1.92	2	C38

1.22	-0.77435	1.34754	1.5	0.3	22.92	1.847	2	C38
1.14	-0.54925	1.1773	1.5	0.3	22.93	2.012	2	C38
1.15	-0.33609	1.02344	1.75	0.2625	23.25	1.539	2.25	C38
1.17	-0.19979	0.929273	1.75	0.2625	23.49	1.54725	2.25	C38
1.1	-0.08807	0.854783	1.5	0.3	23.47	1.994	2	C38
1.1	0.165929	0.695339	1.75	0.2625	23.38	1.54575	2.25	C38
1.15	0.80013	0.367506	1.75	0.2625	23.25	1.539	2.25	C38
1.17	1.07513	0.260611	1.75	0.2625	23.49	1.54725	2.25	C38
1.1	1.35018	0.175908	1.75	0.2625	23.38	1.54575	2.25	C38
1.09	-0.02362	0.812983	4.07	0.4477	23.65	1.3706	4.57	C35
0.86	0.150385	0.704677	5.55	0.6105	23.85	1.5455	6.05	C35
0.55	0.229713	0.657627	6.045	0.66495	23.84	1.6236	6.545	C35
0.56	0.360863	0.583234	5.55	0.6105	23.82	1.57465	6.05	C35
0.48	0.437375	0.541866	5.65	0.7345	23.76	1.9825	7.15	C35
0.32	0.665999	0.427663	6.09	0.7308	23.74	1.818	7.09	C35
0.34	0.879841	0.334229	6.09	0.7308	23.75	1.83	7.09	C35
0.53	0.985519	0.292993	7.075	0.849	23.8	1.9398	8.075	C35
0.75	1.09339	0.254304	7.075	0.849	23.99	1.95	8.075	C35
0.68	1.35547	0.174493	7.645	0.99385	24.12	2.3114	9.645	C35
0.55	-0.40661	1.07348	6.045	0.66495	23.84	1.6236	6.545	C35
0.56	-0.02846	0.816093	5.55	0.6105	23.82	1.57465	6.05	C35
0.48	0.133311	0.714998	5.65	0.7345	23.76	1.9825	7.15	C35
0.32	0.159298	0.699316	6.09	0.7308	23.74	1.818	7.09	C35
0.34	0.282939	0.626916	6.09	0.7308	23.75	1.83	7.09	C35
0.53	0.479482	0.51976	7.075	0.849	23.8	1.9398	8.075	C35
0.75	0.719021	0.403265	7.075	0.849	23.99	1.95	8.075	C35
0.68	0.721507	0.40214	7.645	0.99385	24.12	2.3114	9.645	C35
0.53	0.853517	0.345013	7.075	0.849	23.8	1.9398	8.075	C35
0.75	0.970967	0.298476	7.075	0.849	23.99	1.95	8.075	C35
0.68	1.16154	0.23163	7.645	0.99385	24.12	2.3114	9.645	C35
0.68	1.36882	0.170953	7.645	0.99385	24.12	2.3114	9.645	C35
0.41	0.284637	0.625948	1	0.1	22.96	1.075	1	C39
0.4	0.465638	0.526976	2.165	0.28145	23.11	1.53985	2.665	C39
0.71	1.28808	0.193126	5.11	0.6132	22.26	1.5894	6.11	S90
0.71	1.22476	0.211814	5.11	0.6132	22.26	1.5894	6.11	S90
1.11	0.997122	0.288667	5.165	0.67145	24.4	1.83625	6.665	C26
0.62	1.09234	0.254667	6.225	0.93375	24.48	2.4075	8.725	C26
0.62	1.34808	0.176473	5.335	0.90695	24.54	2.67835	8.335	C26
1.11	0.149355	0.705297	5.165	0.67145	24.4	1.83625	6.665	C26
0.62	0.278888	0.629229	6.225	0.93375	24.48	2.4075	8.725	C26
0.62	0.46622	0.526672	5.335	0.90695	24.54	2.67835	8.335	C26
2.54	0.57206	0.472843	2.335	0.39695	22.52	2.17685	3.335	I65
0.94	1.32498	0.182762	2.165	0.28145	23.1	1.5522	2.665	I65
0.73	-0.13835	0.887994	1.5	0.15	20.09	0.9495	1.5	C39
0.9	0.102072	0.73406	1.5	0.15	20.07	0.955	1.5	C39
0.84	0.356628	0.585569	1.5	0.15	20.06	0.961	1.5	C39
0.81	0.508247	0.504932	1.5	0.15	20.01	0.961	1.5	C39
0.91	0.620641	0.449169	1	0.1	20.1	0.9025	1	C39
0.81	0.685734	0.418488	1	0.1	20.09	0.8995	1	C39
0.83	0.79302	0.370564	1	0.1	20.13	0.902	1	C39
0.7	0.951334	0.305972	1	0.1	20.14	0.9045	1	C39

0.73	1.0009	0.287268	1	0.1	20.21	0.9025	1	C39
0.21	-0.18209	0.917302	1	0.1	23.65	0.681	1	D46
0.75	0.228638	0.658255	2	0.4	22.7	1.823	3	C29
0.51	0.452131	0.534066	2	0.4	22.85	2.092	3	C29
0.43	0.524808	0.496497	2	0.4	22.92	2.092	3	C29
0.43	0.730811	0.397949	2	0.4	23.01	2.092	3	C29
1.01	-0.07196	0.844249	2.625	0.34125	21.26	1.3	3.125	R85
0.94	0.0698	0.753986	2.625	0.34125	21.42	1.30715	3.125	R85
1.13	0.246238	0.648018	2.625	0.34125	21.39	1.2883	3.125	R85
1.01	0.069978	0.753875	2.625	0.34125	21.26	1.3	3.125	R85
0.94	0.149306	0.705327	2.625	0.34125	21.42	1.30715	3.125	R85
1.13	0.280456	0.628333	2.625	0.34125	21.39	1.2883	3.125	R85
1.01	0.023305	0.783108	2.625	0.34125	21.26	1.3	3.125	R85
0.94	0.27786	0.629816	2.625	0.34125	21.42	1.30715	3.125	R85
1.13	0.42948	0.546064	2.625	0.34125	21.39	1.2883	3.125	R85
0.94	-0.84563	1.40294	2.625	0.34125	21.42	1.30715	3.125	R85
1.13	-0.71051	1.29851	2.625	0.34125	21.39	1.2883	3.125	R85
0.84	0.98917	0.291628	1.75	0.2625	22.06	1.46925	2.25	C29
0.91	1.24753	0.20496	1.75	0.2625	22.12	1.53075	2.25	C29
0.84	0.833797	0.353224	1.75	0.2625	22.06	1.46925	2.25	C29
0.91	1.08557	0.256995	1.75	0.2625	22.12	1.53075	2.25	C29
0.15	0.624956	0.447098	2	0.2	23.93	1.1215	2	K70
0.15	0.698094	0.412798	2.335	0.39695	24.25	2.07655	3.335	K70
0.15	0.905734	0.323821	3.1	0.372	24.66	1.5258	3.6	K70
0.78	0.330598	0.600017	2.5	0.25	24.32	1.267	2.5	C36
0.69	0.446441	0.537067	2.5	0.25	24.55	1.268	2.5	C36
0.75	0.658216	0.431312	2.5	0.25	24.7	1.293	2.5	C36
0.83	0.818448	0.359694	3.5	0.35	24.97	1.3735	3.5	C36
0.8	0.779191	0.376554	3.5	0.35	25.22	1.3815	3.5	C36
0.76	0.85776	0.343261	3.5	0.35	25.39	1.381	3.5	C36
0.72	0.970433	0.298678	3.5	0.35	25.39	1.379	3.5	C36
0.64	1.14054	0.238473	3.5	0.35	25.26	1.376	3.5	C36
0.71	1.2898	0.192632	4	0.4	25.3	1.409	4	C36
0.83	0.65876	0.431056	3.5	0.35	24.97	1.3735	3.5	C36
0.8	0.554626	0.481499	3.5	0.35	25.22	1.3815	3.5	C36
0.76	0.746734	0.390832	3.5	0.35	25.39	1.381	3.5	C36
0.72	0.864185	0.340618	3.5	0.35	25.39	1.379	3.5	C36
0.64	0.981635	0.29445	3.5	0.35	25.26	1.376	3.5	C36
0.71	1.17854	0.226187	4	0.4	25.3	1.409	4	C36
0.71	0.201566	0.674149	4	0.4	25.3	1.409	4	C36
1.03	0.064049	0.757562	2.165	0.28145	24.91	1.287	2.665	C38
1.1	0.127695	0.718409	2.625	0.34125	25.17	1.3169	3.125	C38
1.03	0.276883	0.630375	2.625	0.34125	25.4	1.31105	3.125	C38
0.99	0.414364	0.554147	2.625	0.34125	25.62	1.31105	3.125	C38
0.96	0.594336	0.461906	2.625	0.34125	25.77	1.3169	3.125	C38
0.63	0.995274	0.289353	2.75	0.4125	25.81	1.635	3.75	C38
0.63	1.17826	0.226278	2.75	0.4125	25.93	1.64025	3.75	C38
0.75	1.37906	0.168272	2.75	0.4125	26.09	1.65975	3.75	C38
0.85	1.52244	0.133754	2.75	0.4125	26.25	1.65975	3.75	C38
0.74	-0.65836	1.25889	2.165	0.28145	26.37	1.6627	2.665	C29
0.6	-0.28884	0.990397	2.165	0.28145	22.51	1.6497	2.665	C29

0.52	-0.08412	0.852196	2.165	0.28145	22.51	1.6627	2.665	C29
0.53	0.097685	0.736754	2.165	0.28145	22.24	1.6692	2.665	C29
0.57	0.198136	0.676175	2.165	0.28145	22.04	1.6497	2.665	C29
0.54	0.389636	0.567498	2.165	0.28145	21.88	1.68805	2.665	C29
0.55	0.543942	0.486845	2.165	0.28145	21.69	1.65555	2.665	C29
0.6	-0.13741	0.887372	2.165	0.28145	22.51	1.6627	2.665	C29
0.6	0.103006	0.733486	2.165	0.28145	22.51	1.6497	2.665	C29
0.52	0.357562	0.585053	2.165	0.28145	22.51	1.6627	2.665	C29
0.53	0.509181	0.504454	2.165	0.28145	22.24	1.6692	2.665	C29
0.57	0.621575	0.44872	2.165	0.28145	22.04	1.6497	2.665	C29
0.54	0.686668	0.418056	2.165	0.28145	21.88	1.68805	2.665	C29
0.55	0.793954	0.370161	2.165	0.28145	21.69	1.65555	2.665	C29
0.61	-0.11103	0.869884	2.875	0.5175	23.66	2.2419	4.375	J67
0.5	0.088591	0.742355	3.835	0.65195	23.78	2.36725	5.835	J67
0.41	-0.07707	0.847588	4.93	1.4297	24.06	5.72315	11.43	J67
0.32	0.061246	0.759308	5.355	1.98135	24.29	8.4915	14.855	J67
0.25	1.24397	0.20602	2	0.2	24.3	1.194	2	J67
0.47	0.627916	0.44568	1	0.1	22.27	0.916	1	C30
0.54	0.727243	0.399553	1	0.1	22.48	1.003	1	C30
0.77	0.694842	0.414291	2.5	1	22.51	4.612	4	C30
0.77	0.694842	0.414291	2.5	1	22.51	4.264	4	C30
0.81	0.95199	0.30572	1	0.1	22.67	1.003	1	C30
0.86	0.657119	0.431828	2.5	1	23.02	4.612	4	C30
0.69	0.304476	0.61469	3.5	0.7	22.59	2.257	6	I64
0.94	0.535113	0.491286	3.8	0.988	22.67	3.8662	7.8	I64
1.03	0.645205	0.43745	3.7	0.888	22.79	3.0684	7.2	I64
1.27	0.701065	0.411437	4.9	2.352	22.95	10.0032	14.4	I64
0.52	-0.05179	0.831146	2.75	0.4125	21.49	1.37025	3.75	C34
1.2	0.500918	0.508689	4	0.4	23.35	1.2625	4	C35
0.77	0.556681	0.480474	5.055	0.55605	23.31	1.54385	5.555	C35
0.78	0.60138	0.458476	5.055	0.55605	23.26	1.5499	5.555	C35
0.61	0.654423	0.433097	5.055	0.55605	23.14	1.53395	5.555	C35
0.36	0.550288	0.483666	5.055	0.55605	22.96	1.53505	5.555	C35
0.47	0.742397	0.392764	5.55	0.6105	22.95	1.59335	6.05	C35
0.67	0.853517	0.345013	5.6	0.672	22.98	1.8252	6.6	C35
0.84	0.970967	0.298476	5.6	0.672	22.96	1.827	6.6	C35
0.7	1.16787	0.229593	5.6	0.672	22.91	1.8312	6.6	C35
0.61	0.11288	0.72744	1	0.1	21.39	0.9275	1	I65
0.65	0.192208	0.679684	1	0.1	21.6	0.9255	1	I65
0.65	0.323358	0.604066	1	0.1	21.71	0.908	1	I65
0.63	0.41253	0.555131	1	0.1	21.86	0.8085	1	I65
0.65	0.634824	0.442382	1	0.1	21.91	0.817	1	I65
0.7	0.848666	0.347022	1	0.1	21.95	0.822	1	I65
0.65	0.954345	0.304815	1	0.1	22.29	0.87	1	I65
0.61	1.06222	0.265132	1	0.1	22.36	0.8805	1	I65
0.59	1.33062	0.181212	1	0.1	22.56	0.9345	1	I65
1.11	0.815397	0.360988	2.625	0.34125	24.01	1.64385	3.125	C28
1.17	0.776141	0.377884	2.625	0.34125	23.95	1.6874	3.125	C28
0.72	0.885787	0.331822	2.5	0.5	21.5	2.307	4	M74
0.61	1.14415	0.237289	2.5	0.5	21.84	2.392	4	M74
0.29	0.742406	0.392759	1.5	0.15	23.93	1.062	1.5	K70

0.33	0.705828	0.40926	2	0.4	23.78	2.324	3	K70
0.26	0.918395	0.318804	2	0.2	23.7	1.112	2	K70
0.33	0.705828	0.40926	2	0.4	23.78	2.324	3	K70
0.16	1.07119	0.261985	2	0.2	23.66	1.112	2	K70
0.2	1.25934	0.201465	2	0.2	23.61	1.112	2	K70
0.36	0.484629	0.51709	2.5	0.25	26.55	1.217	2.5	C39
0.4	0.962231	0.301797	1.5	0.15	26.27	1.1985	1.5	C39
0.34	1.11503	0.246958	2	0.2	26.44	1.175	2	C39
0.36	1.30317	0.188839	2.5	0.25	26.55	1.217	2.5	C39
0.64	0.708134	0.408209	3.1	0.372	23.04	1.3674	3.6	C17
0.65	0.872714	0.337129	3.1	0.372	23.08	1.3674	3.6	C17
0.6	1.11182	0.248038	3.2	0.448	23.2	1.6653	4.2	C17
0.61	-0.10382	0.865131	3.835	0.65195	23	2.4157	5.835	B09
0.9	-0.02398	0.813211	3.75	0.5625	23.23	2.1105	5.25	B09
1.04	0.092081	0.740203	3.4	0.612	23.46	2.5317	5.4	B09
1.1	0.276091	0.630828	4.69	0.6566	23.55	2.0776	6.19	B09
1	0.454447	0.532847	5.75	0.8625	23.69	2.45925	8.25	B09
0.93	0.712152	0.40638	7.19	1.0066	23.85	2.4871	9.69	B09
0.81	0.876732	0.335492	7.19	1.0066	23.89	2.5011	9.69	B09
0.66	0.995383	0.289313	4.085	0.8987	23.83	3.4496	7.585	B09
0.62	1.23136	0.20981	8.2	1.148	23.95	2.6964	11.2	B09
0.43	1.46974	0.145795	8.335	1.41695	23.96	3.66775	13.335	B09
0.61	-0.65379	1.25544	3.835	0.65195	23	2.4157	5.835	B09
0.9	-0.27161	0.978454	3.75	0.5625	23.23	2.1105	5.25	B09
1.04	-0.08588	0.853351	3.4	0.612	23.46	2.5317	5.4	B09
1.1	0.121243	0.722335	4.69	0.6566	23.55	2.0776	6.19	B09
1	0.215363	0.666026	5.75	0.8625	23.69	2.45925	8.25	B09
0.93	0.413193	0.554775	7.19	1.0066	23.85	2.4871	9.69	B09
0.81	0.5675	0.475099	7.19	1.0066	23.89	2.5011	9.69	B09
0.66	0.586332	0.46582	4.085	0.8987	23.83	3.4496	7.585	B09
0.62	0.7447	0.391737	8.2	1.148	23.95	2.6964	11.2	B09
0.43	0.820827	0.358687	8.335	1.41695	23.96	3.66775	13.335	B09
1.1	0.383715	0.570719	4.69	0.6566	23.55	2.0776	6.19	B09
1	0.466557	0.526495	5.75	0.8625	23.69	2.45925	8.25	B09
0.93	0.695181	0.414135	7.19	1.0066	23.85	2.4871	9.69	B09
0.81	0.909023	0.322513	7.19	1.0066	23.89	2.5011	9.69	B09
0.66	0.964059	0.3011	4.085	0.8987	23.83	3.4496	7.585	B09
0.62	1.12258	0.244428	8.2	1.148	23.95	2.6964	11.2	B09
0.43	1.37199	0.17012	8.335	1.41695	23.96	3.66775	13.335	B09
1.1	0.316218	0.608072	4.69	0.6566	23.55	2.0776	6.19	B09
1	0.427338	0.547205	5.75	0.8625	23.69	2.45925	8.25	B09
0.93	0.634475	0.442548	7.19	1.0066	23.85	2.4871	9.69	B09
0.81	0.733802	0.396606	7.19	1.0066	23.89	2.5011	9.69	B09
0.66	0.84067	0.350349	4.085	0.8987	23.83	3.4496	7.585	B09
0.62	0.958549	0.303204	8.2	1.148	23.95	2.6964	11.2	B09
0.43	0.834598	0.352888	8.335	1.41695	23.96	3.66775	13.335	B09
1	0.627571	0.445846	5.75	0.8625	23.69	2.45925	8.25	B09
0.93	0.691057	0.416033	7.19	1.0066	23.85	2.4871	9.69	B09
0.81	0.876481	0.335594	7.19	1.0066	23.89	2.5011	9.69	B09
0.66	1.00416	0.286061	4.085	0.8987	23.83	3.4496	7.585	B09
0.62	1.27527	0.196813	8.2	1.148	23.95	2.6964	11.2	B09

0.43	1.42563	0.156446	8.335	1.41695	23.96	3.66775	13.335	B09
1	0.670944	0.425354	5.75	0.8625	23.69	2.45925	8.25	B09
0.93	0.57314	0.47231	7.19	1.0066	23.85	2.4871	9.69	B09
0.81	0.765249	0.382651	7.19	1.0066	23.89	2.5011	9.69	B09
0.66	0.832056	0.353954	4.085	0.8987	23.83	3.4496	7.585	B09
0.62	1.00015	0.287545	8.2	1.148	23.95	2.6964	11.2	B09
0.43	1.17807	0.226339	8.335	1.41695	23.96	3.66775	13.335	B09
0.62	1.19874	0.21983	8.2	1.148	23.95	2.6964	11.2	B09
0.43	1.38534	0.166643	8.335	1.41695	23.96	3.66775	13.335	B09
0.96	0.609297	0.454638	2.335	0.39695	24.65	2.23635	3.335	C33
0.83	1.0851	0.257158	2.75	0.4125	24.62	2.0085	3.75	C33
0.8	1.13556	0.240115	2.875	0.5175	24.67	2.4867	4.375	C33
0.67	0.436849	0.542146	1.5	0.15	23.79	1.0165	1.5	C30
0.95	1.19802	0.220053	3.3	0.528	22.6	2.1528	4.8	L72
0.97	1.10237	0.25124	5.11	0.6132	22.85	1.5102	6.11	C39
0.94	1.28418	0.194242	4.625	0.60125	23.08	1.72185	5.625	C39
1	0.884532	0.332329	3	0.3	22.61	0.9585	3	C39
1.02	0.98388	0.293608	4.07	0.4477	22.75	1.21055	4.57	C39
0.97	1.08542	0.257045	5.11	0.6132	22.85	1.5102	6.11	C39
0.94	1.3475	0.176629	4.625	0.60125	23.08	1.50605	5.625	C39
0.84	0.154656	0.702105	1	0.1	21.55	1.0375	1	C34
0.66	0.303844	0.615046	1	0.1	21.53	1.0435	1	C34
0.63	0.441325	0.539773	1	0.1	21.66	1.049	1	C34
0.61	0.621298	0.448853	1	0.1	21.79	1.05	1	C34
0.77	0.849708	0.34659	5.7	0.798	22.21	2.1903	7.7	C34
0.62	1.01591	0.281747	6.625	0.86125	22.39	2.11055	8.125	C34
0.63	1.19889	0.219783	6.665	0.86645	22.44	2.1372	8.665	C34
0.62	1.39969	0.162959	6.665	0.86645	22.52	2.14045	8.665	C34
0.56	1.54307	0.129239	6.665	0.86645	22.73	2.145	8.665	C34
0.77	0.655331	0.432669	5.7	0.798	22.21	2.1903	7.7	C34
0.62	0.875503	0.335992	6.625	0.86125	22.39	2.11055	8.125	C34
0.63	0.981182	0.294621	6.665	0.86645	22.44	2.1372	8.665	C34
0.62	1.08906	0.255794	6.665	0.86645	22.52	2.14045	8.665	C34
0.56	1.35746	0.173961	6.665	0.86645	22.73	2.145	8.665	C34
0.68	0.154656	0.702105	1.5	0.15	22.27	0.917	1.5	C34
0.58	0.303844	0.615046	1.5	0.15	22.39	0.906	1.5	C34
0.52	0.441325	0.539773	1.5	0.15	22.41	0.906	1.5	C34
0.37	0.621298	0.448853	1.5	0.15	22.69	0.917	1.5	C34
0.27	0.875029	0.336185	1.5	0.15	22.61	0.959	1.5	C34
0.28	1.0349	0.274857	1.5	0.15	22.59	0.959	1.5	C34
0.27	1.21788	0.213914	1.5	0.15	22.54	0.9915	1.5	C34
0.26	1.41869	0.158173	1.5	0.15	22.44	1.0415	1.5	C34
0.34	0.236043	0.653938	1.5	0.15	21.1	0.955	1.5	C35
0.43	0.367193	0.579754	1.5	0.15	21.36	0.965	1.5	C35
0.48	0.456366	0.531837	1.5	0.15	21.39	0.965	1.5	C35
0.43	0.67866	0.421764	1.5	0.15	21.75	1.0085	1.5	C35
0.44	0.892502	0.329115	1.5	0.15	21.81	1.0195	1.5	C35
0.51	0.99818	0.288274	2	0.2	21.83	1.064	2	C35
0.54	1.10605	0.249988	2	0.2	22.38	1.0545	2	C35
0.44	1.37446	0.169473	2	0.2	22.33	1.0695	2	C35
0.51	0.866178	0.339801	2	0.2	21.83	1.054	2	C35

0.54	0.983628	0.293702	2	0.2	22.38	1.054	2	C35
0.92	0.874148	0.336544	2.5	0.25	25.03	1.1835	2.5	C31
0.99	0.991598	0.290722	2.5	0.25	25	1.1875	2.5	C31
0.99	1.18851	0.223036	2.5	0.25	24.97	1.191	2.5	C31
0.3	1.08076	0.258656	2	0.2	24.41	1.1245	2	N77
0.24	1.21104	0.216017	2.165	0.28145	24.52	1.5418	2.665	N77
4.56	-0.01066	0.804685	4.5	0.9	23.8	3.475	8	C13
3.16	0.131091	0.716346	5	1	23.93	3.673	9	C13
2.56	0.294868	0.620129	3.6	0.792	24.11	3.4925	6.6	C13
4.56	-0.24828	0.962375	4.5	0.9	23.8	3.475	8	C13
3.16	-0.14262	0.890839	5	1	23.93	3.683	9	C13
0.86	2.63927	0.012306	6.335	2.34395	23.2	9.03725	18.335	C29
0.86	2.62753	0.012694	9.88	2.7664	23.45	8.799	24.88	C29
0.78	1.09234	0.254664	10.87	2.9349	23.5	9.15975	27.37	C29
0.68	1.25259	0.203458	11.325	3.05775	23.62	9.2421	27.825	C29
1.01	0.173992	0.690518	4.585	1.4672	22.73	5.6112	11.085	C29
0.86	0.248008	0.646993	6.335	2.34395	23.2	9.07795	18.335	C29
0.86	0.469562	0.524926	9.88	2.7664	23.45	8.8298	24.88	C29
0.78	0.646363	0.436902	10.87	2.9349	23.5	9.18945	27.37	C29
0.68	0.860474	0.342143	11.325	3.05775	23.62	9.3771	27.825	C29
0.99	0.664682	0.428279	3.3	0.528	22.83	1.9552	4.8	C20
1.03	0.782133	0.375276	3.3	0.528	22.59	1.9552	4.8	C20
1.04	0.821519	0.358394	3.2	0.448	22.5	1.6408	4.2	C20
1.13	0.970782	0.298546	3.2	0.448	22.57	1.6408	4.2	C20
1.08	1.12991	0.241985	3.665	0.47645	22.66	1.5886	4.665	C20
0.96	1.31173	0.18644	3.2	0.448	22.8	1.6408	4.2	C20
0.91	0.152552	0.703372	1.5	0.15	22.1	1.052	1.5	C37
0.79	-0.01823	0.809525	1	0.1	21.81	0.955	1	C27
0.75	0.116821	0.725032	1	0.1	21.89	0.955	1	C27
0.8	0.27551	0.63116	1	0.1	21.92	0.96	1	C27
0.8	0.460196	0.529827	1	0.1	22.01	0.9205	1	C27
0.86	0.711571	0.406644	1	0.1	22.07	0.9315	1	C27
0.92	0.876151	0.335728	1	0.1	22.07	0.9355	1	C27
0.82	1.04544	0.271077	1	0.1	22.07	0.941	1	C27
0.74	1.23078	0.209986	1	0.1	22.08	0.9565	1	C27
0.63	1.48815	0.141505	1	0.1	22.15	1.019	1	C27
2.33	0.307622	0.612913	2.625	0.34125	22.76	1.5496	3.125	C38
2.24	0.423465	0.549273	2.165	0.28145	22.94	1.5171	2.665	C38
2.29	0.63524	0.442184	2.625	0.34125	23.21	1.58145	3.125	C38
2.1	0.795471	0.369508	2.625	0.34125	23.31	1.59445	3.125	C38
1.7	0.756215	0.38663	2.165	0.28145	23.38	1.5535	2.665	C38
1.59	0.834784	0.352811	2.165	0.28145	23.67	1.56715	2.665	C38
0.58	0.976262	0.296474	3.5	0.35	23.33	1.178	3.5	E48
0.57	1.07148	0.261887	3.585	0.4302	23.47	1.4856	4.085	E48
0.79	1.34621	0.176976	4.07	0.4477	23.63	1.41185	4.57	E48
0.69	0.726809	0.399748	3	0.3	23.34	1.1195	3	E48
0.58	0.84426	0.348853	3.5	0.35	23.33	1.178	3.5	E48
0.57	0.949049	0.306851	3.585	0.4302	23.47	1.4856	4.085	E48
0.79	1.15229	0.23463	4.07	0.4477	23.63	1.41185	4.57	E48
0.69	0.454049	0.533056	3	0.3	23.34	1.1195	3	E48
0.58	0.488947	0.514856	3.5	0.35	23.33	1.178	3.5	E48

0.57	0.6858	0.418457	3.585	0.4302	23.47	1.4856	4.085	E48
0.79	0.981199	0.294614	4.07	0.4477	23.63	1.41185	4.57	E48
1.14	0.434715	0.543279	2.75	0.4125	22.65	1.6575	3.75	C26
1.07	0.646489	0.436842	2.75	0.4125	22.6	1.6575	3.75	C26
1.34	0.894589	0.328277	5.585	2.9042	23.73	12.8232	18.085	C26
1.03	0.993209	0.290122	5.5	3.3	23.57	14.511	18	C26
1.14	0.350879	0.588745	2.75	0.4125	22.65	1.6575	3.75	C26
1.07	0.502498	0.507878	2.75	0.4125	22.6	1.6575	3.75	C26
1.34	0.760923	0.384553	5.585	2.9042	23.73	12.8232	18.085	C26
1.03	0.968639	0.299359	5.5	3.3	23.57	14.511	18	C26
1.04	0.157606	0.700332	4.815	0.7704	24.73	2.3656	7.315	C39
1.04	1.13458	0.240437	4.815	0.7704	24.73	2.3656	7.315	C39
1.04	1.38215	0.167469	4.815	0.7704	24.73	2.3656	7.315	C39
1.27	0.788478	0.372525	2.5	0.5	24.34	2.391	4	C39
0.98	0.856838	0.343641	2.835	0.76545	24.47	3.4749	5.335	C39
1.05	1.02695	0.277729	2.835	0.76545	24.61	3.4371	5.335	C39
1.04	1.24584	0.205463	4.815	0.7704	24.73	2.3656	7.315	C39
1.04	1.22127	0.212876	4.815	0.7704	24.73	2.3656	7.315	C39
1.27	0.821228	0.358517	2.5	0.5	24.34	2.391	4	C39
0.98	0.882594	0.333113	2.835	0.76545	24.47	3.4749	5.335	C39
1.05	0.990468	0.291143	2.835	0.76545	24.61	3.4371	5.335	C39
1.04	1.32851	0.181793	4.815	0.7704	24.73	2.3656	7.315	C39
1.73	1.00648	0.285207	6.68	1.6032	23.68	5.0292	14.18	F52
1.73	1.20095	0.21914	7.165	1.64795	23.79	5.04965	15.165	F52
1.73	1.26427	0.200017	7.165	1.64795	23.79	5.1451	15.165	F52
2.16	0.596549	0.460827	2.5	0.5	23.97	2.447	4	C39
1.87	0.7771	0.377465	3.3	0.528	24.1	2.0768	4.8	C39
0.57	0.034937	0.775777	1.75	0.2625	21.81	1.61025	2.25	A01
0.52	0.176691	0.688908	1.75	0.2625	22	1.5915	2.25	A01
0.51	0.353129	0.587501	1.75	0.2625	22.07	1.63575	2.25	A01
0.3	1.14558	0.23682	3.665	0.47645	23.29	1.82	4.665	A01
0.3	-0.12935	0.88201	3.665	0.47645	23.29	1.82	4.665	A01
0.25	-0.03029	0.817264	3.5	0.7	23.45	3.022	6	A01
0.3	1.03099	0.276264	3.665	0.47645	23.29	1.82	4.665	A01
0.25	1.09456	0.253906	3.5	0.7	23.45	3.022	6	A01
0.2	1.54857	0.128054	2	0.4	23.46	2.505	3	A01
1.34	0.085032	0.744551	1	0.1	21.94	0.871	1	C37
1.27	0.148679	0.705705	1	0.1	22.08	0.8675	1	C37
1	0.297867	0.618429	1	0.1	22.24	0.866	1	C37
0.92	0.435347	0.542943	1	0.1	22.31	0.866	1	C37
0.85	0.61532	0.45173	1	0.1	22.48	0.8675	1	C37
0.76	0.869052	0.338624	1	0.1	22.59	0.871	1	C37
0.73	1.02892	0.277015	1.5	0.15	22.56	0.921	1.5	C37
0.78	1.2119	0.21575	1.5	0.15	22.66	1.0055	1.5	C37
0.79	1.3684	0.171065	2.335	0.39695	22.83	1.9652	3.335	C37
0.69	1.51177	0.136132	2.335	0.39695	22.85	1.9652	3.335	C37
0.08	0.486279	0.516236	5.5	1.1	25.58	4.044	10	G55
0.08	0.692901	0.415183	4.43	0.8417	25.78	3.4048	7.43	G55
0.21	-0.20981	0.936073	11.8	21.948	26.77	106.922	55.8	K70
0.17	0.131083	0.71635	10.7	17.548	27.15	85.5998	49.2	K70
0.48	0.347803	0.590447	1.5	0.15	23.39	1.0395	1.5	C26

0.42	0.51474	0.501616	1.5	0.15	23.37	1.0395	1.5	C26
0.34	0.675786	0.423099	1.5	0.15	23.55	1.0395	1.5	C26
0.89	0.434514	0.543386	1.75	0.2625	21.71	1.44075	2.25	C18
0.91	0.641297	0.439303	1.75	0.2625	21.83	1.449	2.25	C18
0.86	0.820669	0.358753	1.75	0.2625	21.88	1.4445	2.25	C18
0.81	1.0153	0.281971	2.625	0.34125	21.97	1.3871	3.125	C18
0.65	-0.4553	1.10855	4.43	0.8417	22.62	2.9051	7.43	C35
0.8	-0.29426	0.994168	4.43	0.8417	22.7	2.9051	7.43	C35
0.75	-0.18078	0.916424	4.5	0.9	22.75	3.158	8	C35
0.77	-0.1097	0.869009	5.44	1.5776	23.06	5.8841	12.94	C35
0.94	0.068118	0.755031	5.665	1.30295	23.37	4.4367	11.665	C35
0.66	0.191321	0.680209	6.3	1.638	23.66	5.6654	14.3	C35
0.68	0.397791	0.563077	3.915	0.7047	22.6	2.5848	6.415	C35
0.65	0.603235	0.457575	4.43	0.8417	22.62	2.9051	7.43	C35
0.8	0.763467	0.383434	4.43	0.8417	22.7	2.91745	7.43	C35
0.75	0.71788	0.403781	4.5	0.9	22.75	3.171	8	C35
0.77	0.739475	0.394068	5.44	1.5776	23.06	5.90295	12.94	C35
0.94	0.887906	0.330966	5.665	1.30295	23.37	4.51375	11.665	C35
0.66	1.02171	0.279629	6.3	1.638	23.66	5.7525	14.3	C35
0.53	1.29379	0.191496	2	0.2	22.49	1.0765	2	C34
0.56	0.719873	0.402879	2.5	0.5	23.06	2.28	4	C34
0.52	0.830093	0.354779	2.75	0.4125	23.19	1.71	3.75	C34
0.64	0.923774	0.316686	2.875	0.5175	23.35	2.0709	4.375	C34
0.77	1.10654	0.249823	3.3	0.528	23.05	1.8992	4.8	C34
0.95	1.25581	0.202506	3.3	0.528	22.89	1.8992	4.8	C34
0.47	1.00488	0.285796	1	0.1	24.01	0.9635	1	C37
0.44	1.26324	0.200318	1	0.1	24.07	1.0145	1	C37
0.88	0.917542	0.31914	4.085	0.8987	23.9	3.5398	7.585	D44
0.84	0.473957	0.522634	2.625	0.34125	20.89	0.85865	3.125	C39
0.85	0.59974	0.459273	2.5	0.25	21.07	0.9915	2.5	C39
0.86	0.652783	0.433869	2.5	0.25	21.18	0.985	2.5	C39
0.92	0.548648	0.484486	2.5	0.25	21.16	0.9785	2.5	C39
0.95	0.740757	0.393495	2.5	0.25	21.19	0.973	2.5	C39
0.75	0.858207	0.343076	2.5	0.25	21.16	1.03	2.5	C39
0.72	0.975658	0.296702	2.5	0.25	21.22	1.028	2.5	C39
0.63	1.17257	0.228091	2.5	0.25	21.46	1.018	2.5	C39
0.84	-0.37933	1.05402	2.625	0.34125	20.89	0.85865	3.125	C39
0.85	-0.12395	0.878432	2.5	0.25	21.07	0.9915	2.5	C39
0.86	0.043351	0.770493	2.5	0.25	21.18	0.985	2.5	C39
0.92	0.239359	0.65201	2.5	0.25	21.16	0.9785	2.5	C39
0.95	0.428308	0.546689	2.5	0.25	21.19	0.973	2.5	C39
0.75	0.635091	0.442255	2.5	0.25	21.16	1.03	2.5	C39
0.72	0.814463	0.361385	2.5	0.25	21.22	1.028	2.5	C39
0.63	0.996428	0.288925	2.5	0.25	21.46	1.018	2.5	C39
0.49	1.07563	0.260437	4.5	0.9	23	2.965	8	C27
0.49	1.27011	0.198311	5.445	1.03455	23.13	3.10175	9.445	C27
0.53	0.166354	0.695085	2.5	0.25	22.31	1.12	2.5	C27
0.59	0.289556	0.623147	2.625	0.34125	22.33	1.521	3.125	C27
0.84	0.129754	0.717157	2.625	0.34125	20.89	0.85865	3.125	C39
0.85	0.359223	0.584137	2.5	0.25	21.07	0.9915	2.5	C39
0.86	0.448396	0.536035	2.5	0.25	21.18	0.985	2.5	C39

0.92	0.670689	0.425472	2.5	0.25	21.16	0.9785	2.5	C39
0.95	0.884532	0.332329	2.5	0.25	21.19	0.973	2.5	C39
0.75	0.99021	0.291239	2.5	0.25	21.16	1.03	2.5	C39
0.72	1.09808	0.2527	2.5	0.25	21.22	1.028	2.5	C39
0.63	1.36649	0.171567	2.5	0.25	21.46	1.018	2.5	C39
0.49	0.699465	0.41217	5.445	1.03455	23.13	3.3421	9.445	C27
0.68	1.28277	0.194647	2.335	0.39695	22.45	1.93205	3.335	C27
1.8	0.241391	0.650829	1.5	0.3	24.81	2.213	2	F52
1.44	0.420538	0.550838	1.5	0.15	25.06	1.074	1.5	F52
1.33	0.600661	0.458826	2.75	0.4125	25.13	1.956	3.75	F52
1.32	0.748232	0.390167	2.335	0.39695	25.13	2.13605	3.335	F52
1.59	0.708976	0.407825	2.335	0.39695	25.2	2.13605	3.335	F52
1.31	0.787544	0.372929	2.335	0.39695	25.64	2.13605	3.335	F52
1.27	0.925538	0.315994	2.165	0.28145	25.78	1.5327	2.665	F52
1.37	1.08299	0.257887	2.75	0.4125	26.02	1.911	3.75	F52
1.23	1.16894	0.22925	3.25	0.8125	26.19	3.685	6.25	F52
0.52	1.56405	0.124761	1	0.1	21.78	0.9165	1	C33
0.84	0.534587	0.491552	5	3.5	23.47	14.1085	14	L72
0.82	0.700045	0.411904	5.375	3.655	23.67	15.7454	16.875	L72
0.63	0.964155	0.301064	5.3	2.968	23.65	13.2468	16.8	L72
0.71	0.230066	0.657421	1.5	0.15	22.19	0.952	1.5	C38
0.72	0.361216	0.58304	1.5	0.15	22.33	0.9545	1.5	C38
0.7	0.450388	0.534984	2.5	0.25	22.42	1.0545	2.5	C38
0.68	0.672682	0.424544	2.5	0.25	22.55	1.077	2.5	C38
0.69	0.886524	0.331524	2.5	0.25	22.59	1.101	2.5	C38
0.67	0.992203	0.290496	4	0.4	22.64	1.236	4	C38
0.66	1.10008	0.25202	6	0.6	22.75	1.4185	6	C38
0.77	1.36848	0.171042	8.5	0.85	22.9	1.701	8.5	C38
0.35	0.156715	0.700867	2	0.2	21.6	0.886	2	C35
0.3	0.236043	0.653938	3	0.3	21.71	0.936	3	C35
0.32	0.354533	0.586725	3.1	0.372	21.76	1.1814	3.6	C35
0.31	0.443705	0.538513	3.585	0.4302	22.11	1.2414	4.085	C35
0.23	0.659668	0.43063	3.665	0.47645	22.2	1.4573	4.665	C35
0.18	0.873511	0.336804	3.665	0.47645	22.28	1.4833	4.665	C35
0.19	0.972859	0.29776	4.215	0.5901	22.67	1.7164	5.715	C35
0.2	1.06807	0.263077	4.285	0.6856	22.68	2.0256	6.285	C35
0.22	1.33015	0.181343	4.355	0.74035	22.47	2.2797	6.855	C35
1.46	0.305837	0.613921	1	0.1	22.32	0.927	1	C33
1.56	0.443318	0.538718	1	0.1	22.47	0.927	1	C33
1.53	0.62329	0.447897	1	0.1	22.62	0.935	1	C33
1.55	0.877022	0.335374	1	0.1	22.72	0.9375	1	C33
1.56	1.03689	0.274141	1	0.1	22.78	0.9375	1	C33
1.66	1.18822	0.223126	1.75	0.2625	22.94	1.566	2.25	C33
1.79	1.38903	0.165691	1.75	0.2625	23.09	1.59225	2.25	C33
1.76	1.54506	0.128809	2.165	0.28145	23.2	1.44495	2.665	C33
0.88	0.812968	0.362021	1.5	0.15	22.08	0.9385	1.5	C39
1.15	0.962231	0.301797	1.5	0.15	22.31	0.9385	1.5	C39
0.82	1.11503	0.246958	2	0.2	22.64	0.9885	2	C39
0.77	1.30317	0.188839	2.5	0.25	22.92	1.0385	2.5	C39
0.74	0.885926	0.331765	2.165	0.28145	22.96	0.93665	2.665	C17
0.86	1.04579	0.270952	2.165	0.28145	23.12	0.93665	2.665	C17

0.93	1.20345	0.218362	2.335	0.39695	23.5	1.5368	3.335	C17
0.82	1.40426	0.161799	2.335	0.39695	23.64	1.5878	3.335	C17
0.86	1.54763	0.128255	2.335	0.39695	23.63	1.5878	3.335	C17
1.37	0.620452	0.44926	2	0.4	22.05	2.148	3	C20
0.98	1.53943	0.130026	3.585	0.4302	22.17	1.497	4.085	C39
0.98	0.589773	0.464135	3.585	0.4302	22.17	1.497	4.085	C39
1.35	0.575832	0.470982	2	0.2	22.32	1.0215	2	E50
1.12	0.543419	0.487107	3.125	0.71875	22.65	3.09925	5.625	E50
0.86	0.794858	0.369772	2.165	0.28145	22.89	1.44365	2.665	E50
0.83	1.02555	0.278235	6	1.2	22.85	3.707	11	E50
0.68	1.32592	0.182505	7.31	1.1696	23.05	2.5992	11.31	E50
1.02	1.2898	0.192632	1.5	0.15	23.56	1.0995	1.5	C36
0.9	0.042369	0.771108	3.5	0.35	22.22	1.1065	3.5	C36
0.9	0.208128	0.67028	2.5	0.25	22.3	1.0135	2.5	C36
0.78	0.376243	0.574796	4	0.4	22.32	1.1725	4	C36
0.92	0.968208	0.299522	3	0.3	23.24	1.065	3	C36
0.9	-0.86697	1.41965	3.5	0.35	22.22	1.1065	3.5	C36
0.9	-0.70238	1.29232	2.5	0.25	22.3	1.0135	2.5	C36
0.78	-0.53545	1.16711	4	0.4	22.32	1.1725	4	C36
0.91	-0.40032	1.06899	2.5	0.25	22.61	1.0445	2.5	C36
0.7	-0.01662	0.808494	4.75	0.7125	23.57	2.14725	6.75	E48
0.55	0.138171	0.712054	5.22	0.7308	23.53	2.0937	7.22	E48
0.42	0.567098	0.475298	6.18	0.8652	23.66	2.1826	8.18	E48
0.58	0.64677	0.436709	6.225	0.93375	23.64	2.41125	8.725	E48
0.55	0.883588	0.332711	7.425	1.3365	23.62	3.6279	12.925	E48
0.53	1.08521	0.257119	7.385	1.3293	23.81	3.5541	12.385	E48
0.55	0.170788	0.692432	5.7	0.798	23.61	2.1861	7.7	E48
0.43	0.455549	0.532267	6.18	0.8652	23.71	2.1588	8.18	E48
0.42	0.567098	0.475298	6.18	0.8652	23.66	2.1826	8.18	E48
0.58	0.64677	0.436709	6.225	0.93375	23.64	2.41125	8.725	E48
0.55	0.883588	0.332711	7.425	1.3365	23.62	3.6279	12.925	E48
0.53	1.08521	0.257119	7.385	1.3293	23.81	3.5541	12.385	E48
0.7	0.047311	0.768011	4.75	0.7125	23.57	2.14725	6.75	E48
0.55	0.21233	0.667807	5.22	0.7308	23.53	2.0937	7.22	E48
0.55	0.397016	0.563497	5.7	0.798	23.61	2.1861	7.7	E48
0.43	0.648391	0.435943	6.18	0.8652	23.71	2.1588	8.18	E48
0.42	0.812971	0.362019	6.18	0.8652	23.66	2.1826	8.18	E48
0.58	0.975934	0.296597	6.225	0.93375	23.64	2.41125	8.725	E48
0.55	1.14228	0.237901	7.425	1.3365	23.62	3.6279	12.925	E48
0.53	1.39964	0.162972	7.385	1.3293	23.81	3.5541	12.385	E48
0.82	0.990358	0.291184	1.5	0.15	21.83	1.048	1.5	C26
0.55	1.16047	0.231979	1.5	0.15	21.8	1.0435	1.5	C26
0.83	0.144333	0.708328	3	0.3	21.94	1.0335	3	C39
0.69	0.157658	0.7003	5.055	0.55605	22.41	1.41185	5.555	C39
0.73	0.274969	0.63147	5.11	0.6132	22.67	1.5882	6.11	C39
0.7	1.06048	0.265744	3.835	0.65195	22.98	2.15135	5.835	C39
0.75	1.12993	0.241978	3.835	0.65195	23.04	2.13775	5.835	C39
0.73	1.34186	0.178152	3.3	0.528	23.05	1.8632	4.8	C39
0.69	0.442456	0.539174	2.5	0.25	22.17	1.022	2.5	C38
0.75	0.654231	0.433187	2.5	0.25	22.2	1.032	2.5	C38
0.79	0.814463	0.361385	2.5	0.25	22.29	1.082	2.5	C38

0.79	0.775206	0.378291	2.5	0.25	22.36	1.082	2.5	C38
0.85	0.853775	0.344906	2.5	0.25	22.49	1.082	2.5	C38
0.97	0.966447	0.300191	2.5	0.25	22.6	1.024	2.5	C38
0.97	1.13655	0.239786	2.5	0.25	22.64	1.024	2.5	C38
0.85	1.28582	0.193772	2.5	0.25	22.86	1.024	2.5	C38
0.9	0.635935	0.441853	1.5	0.15	24.24	1.0655	1.5	E48
0.68	-0.30372	1.00076	1	0.1	22.95	0.9715	1	E48
0.64	-0.099	0.86196	1	0.1	23.15	0.9765	1	E48
0.63	0.082803	0.745929	1	0.1	23.25	0.9815	1	E48
0.58	0.183254	0.684999	1	0.1	23.36	0.9835	1	E48
0.56	0.374754	0.575611	1	0.1	23.42	1.03	1	E48
0.61	0.52906	0.494343	1	0.1	23.31	0.971	1	E48
0.61	0.598536	0.459859	1	0.1	23.29	1.015	1	E48
0.71	0.706261	0.409063	1	0.1	23.4	1.068	1	E48
0.78	0.738075	0.394693	1.5	0.3	23.48	2.258	2	E48
0.52	0.22711	0.659147	1	0.1	22.41	1.0405	1	C28
0.52	0.489914	0.514357	1	0.1	22.41	1.042	1	C28
0.75	0.524812	0.496495	1	0.1	22.44	1.0415	1	C28
0.77	0.734327	0.396371	1	0.1	22.5	1.0415	1	C28
1.2	1.0044	0.285972	2.625	0.34125	23.55	1.6237	3.125	C28
0.52	0.842358	0.349645	1.5	0.15	22.48	1.0065	1.5	C22
0.51	0.803102	0.366232	1.5	0.15	22.47	1.0065	1.5	C22
0.64	0.88167	0.333487	1.5	0.15	22.46	1.0065	1.5	C22
0.88	0.994343	0.2897	1.5	0.15	22.56	0.992	1.5	C22
0.93	1.16445	0.230694	1.5	0.15	22.58	0.9875	1.5	C22
0.86	1.29472	0.191231	2.165	0.28145	22.46	1.41375	2.665	C22
0.56	0.426056	0.547889	3.5	0.35	22.32	1.1435	3.5	A04
0.51	0.56118	0.478235	3.5	0.35	22.39	1.143	3.5	A04
0.52	0.718252	0.403612	4.07	0.4477	22.31	1.3618	4.57	A04
0.58	0.823042	0.35775	4.145	0.53885	22.22	1.67505	5.145	A04
0.7	0.862428	0.34134	4.07	0.4477	22.16	1.3783	4.57	A04
0.76	0.99903	0.287959	3.665	0.47645	22.1	1.6289	4.665	A04
0.74	1.13917	0.238925	3.75	0.5625	21.99	1.91475	5.25	A04
0.83	1.3463	0.176951	3.585	0.4302	21.82	1.4118	4.085	A04
0.52	0.86193	0.341544	4.07	0.4477	22.31	1.3618	4.57	A04
0.58	0.810014	0.363279	4.145	0.53885	22.22	1.6744	5.145	A04
0.7	0.901243	0.325612	4.07	0.4477	22.16	1.3618	4.57	A04
0.76	1.00125	0.287136	3.665	0.47645	22.1	1.5639	4.665	A04
0.74	1.1587	0.232549	3.75	0.5625	21.99	1.83975	5.25	A04
0.83	1.32696	0.182219	3.585	0.4302	21.82	1.3518	4.085	A04
0.43	0.058134	0.761248	1	0.1	22.13	0.861	1	C35
0.47	0.157342	0.70049	2.165	0.28145	22.41	1.2909	2.665	C35
0.27	0.210385	0.668951	2.165	0.28145	22.57	1.25905	2.665	C35
0.38	0.195293	0.677856	2.335	0.39695	22.63	1.73145	3.335	C35
0.4	0.316279	0.608038	2.335	0.39695	22.79	1.73145	3.335	C35
0.31	0.256992	0.6418	2.335	0.39695	22.81	1.73145	3.335	C35
0.23	0.36266	0.582245	2.335	0.39695	22.83	1.73145	3.335	C35
0.32	0.723034	0.401451	2.165	0.28145	22.9	1.25905	2.665	C35
0.35	0.966447	0.300191	1.5	0.15	23.56	1.0425	1.5	C38
0.27	1.13655	0.239786	2.5	0.25	23.75	1.1315	2.5	C38
0.22	1.28582	0.193772	2.5	0.25	23.95	1.1315	2.5	C38

0.77	-0.11108	0.869921	1.75	0.2625	23.17	1.67925	2.25	C22
0.65	-0.01225	0.8057	2	0.2	23.44	1.07	2	C22
0.68	0.122799	0.721388	2	0.2	23.44	1.065	2	C22
0.7	0.281488	0.627744	1.5	0.15	23.49	1.015	1.5	C22
0.65	0.466173	0.526696	1.5	0.15	23.51	1.02	1.5	C22
0.6	0.717548	0.403931	1.5	0.15	23.7	1.0695	1.5	C22
0.72	0.882129	0.333302	1.5	0.15	23.73	1.0695	1.5	C22
0.81	1.05142	0.268949	2.5	0.25	23.98	1.1695	2.5	C22
0.78	1.21777	0.213948	2.165	0.28145	24.11	1.5366	2.665	C22
0.73	1.47513	0.144528	2.165	0.28145	24.2	1.5444	2.665	C22
0.49	1.42666	0.156194	1.5	0.15	23.9	1.013	1.5	C30
0.5	1.57003	0.123506	1	0.1	24.1	1.0295	1	C30
0.75	0.129158	0.717519	4.565	0.50215	24.01	1.12255	5.065	E50
0.76	0.267978	0.635478	5.28	0.8448	24.06	2.2488	7.78	E50
0.81	0.441245	0.539815	6.225	0.93375	24.23	2.3085	8.725	E50
0.84	0.583299	0.467308	5.9	1.062	24.4	2.9376	9.9	E50
0.76	0.510519	0.503771	5.28	0.8448	24.06	2.2488	7.78	E50
0.81	0.525901	0.495943	6.225	0.93375	24.23	2.3085	8.725	E50
0.84	0.707202	0.408633	5.9	1.062	24.4	2.9556	9.9	E50
1.25	0.365201	0.580848	2	0.2	22.16	1.008	2	C36
1.19	0.454374	0.532885	2	0.2	22.35	1.008	2	C36
1.22	0.607033	0.455734	4.57	0.9597	22.9	3.50595	8.57	C36
1.22	0.709557	0.40756	4.57	0.9597	22.9	3.29595	8.57	C36
0.76	0.419717	0.551277	1.75	0.2625	20.75	1.7115	2.25	C38
0.73	0.762704	0.383769	2.25	0.5625	20.9	2.9425	3.75	C38
0.76	1.57475	0.122521	2.5	1	21.16	4.69	4	C38
0.73	1.89743	0.067898	3.5	2.1	21.22	7.695	6	C38
0.57	2.30489	0.028309	3	1.5	21.69	6.225	5	C38
0.58	2.53904	0.015975	3	1.5	22.06	6.475	5	C38
0.59	2.47033	0.018998	3	1.5	22.1	6.475	5	C38
0.42	1.05542	0.267531	2.5	0.75	22.08	4.035	4.5	C38
0.52	1.34227	0.178041	1.5	0.15	22.03	1.14	1.5	C38
1.82	-0.16883	0.908381	5.75	3.1625	23.8	13.948	19.25	F51
1.74	0.030625	0.778491	6.835	3.21245	23.88	13.9637	23.335	F51
1.82	0.420911	0.550638	5.75	3.1625	23.8	13.948	19.25	F51
1.74	0.480371	0.519298	6.835	3.21245	23.88	13.9637	23.335	F51
2.2	0.768309	0.381308	1.5	0.15	22.04	1.001	1.5	F51
3.31	0.725401	0.400383	4.57	0.9597	23.38	3.36105	8.57	F51
2.09	0.792369	0.370845	5.215	1.7731	23.66	7.0329	13.715	F51
1.74	1.05102	0.269093	6.835	3.21245	23.88	13.9637	23.335	F51
0.91	0.10695	0.731068	1.5	0.15	20.59	1.034	1.5	C26
1.16	0.170597	0.692546	1.5	0.15	20.72	0.959	1.5	C26
1.34	0.319785	0.60607	1.5	0.15	20.83	0.9565	1.5	C26
1.3	0.457265	0.531365	1.5	0.15	20.83	0.9565	1.5	C26
1.04	0.637238	0.441232	1.5	0.15	20.93	0.985	1.5	C26
0.94	0.890969	0.329732	1.5	0.15	21.37	1.034	1.5	C26
0.98	1.05084	0.269157	1.5	0.15	21.78	1.034	1.5	C26
1.24	1.20217	0.218762	1.75	0.2625	22.26	1.6485	2.25	C26
1.03	1.37132	0.170296	2	0.4	22.5	2.31	3	C26
0.95	1.48304	0.142685	2.25	0.5625	22.44	3.0125	3.75	C26
0.34	0.002519	0.796282	1.5	0.15	20.04	0.8395	1.5	I64

0.27	0.168277	0.693933	1.5	0.15	20.08	0.8395	1.5	I64
0.28	0.336392	0.596786	1.5	0.15	20.15	0.843	1.5	I64
0.34	0.471516	0.523906	1.5	0.15	20.2	0.8505	1.5	I64
0.27	0.634919	0.442337	1.5	0.15	20.19	0.8775	1.5	I64
1.74	0.397726	0.563113	6.835	3.21245	23.88	13.9637	23.335	F51
0.3	0.606644	0.455922	3.1	0.372	22.58	1.2816	3.6	C35
0.23	0.866706	0.339584	5.055	0.55605	22.57	1.3926	5.555	C35
0.24	1.02657	0.277864	5.55	0.6105	23.04	1.4399	6.05	C35
0.35	1.20956	0.216473	5.055	0.55605	23.23	1.4278	5.555	C35
0.43	1.41036	0.160258	5.055	0.55605	23.38	1.41735	5.555	C35
0.49	1.52208	0.133832	7.31	1.1696	23.48	2.9032	11.31	C35
3.31	0.409066	0.556994	4.57	0.9597	23.38	3.36105	8.57	F51
2.09	0.511082	0.503483	5.215	1.7731	23.66	7.0329	13.715	F51
0.68	-0.38834	1.06043	1.5	0.15	21.37	0.8305	1.5	C27
0.57	-0.4057	1.07283	1.5	0.15	21.47	0.82	1.5	C27
0.42	-0.2423	0.958264	1.5	0.15	22.15	0.82	1.5	C27
0.54	-0.14488	0.892346	1.5	0.15	22.3	0.82	1.5	C27
3.31	0.764215	0.383105	4.57	0.9597	23.38	3.36105	8.57	F51
2.09	0.794593	0.369886	5.215	1.7731	23.66	7.0329	13.715	F51
0.23	0.643294	0.438356	5.055	0.55605	22.57	1.3926	5.555	C35
0.24	0.831429	0.354218	5.55	0.6105	23.04	1.4399	6.05	C35
0.35	1.10643	0.24986	5.055	0.55605	23.23	1.4278	5.555	C35
0.43	1.17589	0.227032	5.055	0.55605	23.38	1.41735	5.555	C35
0.49	1.34983	0.176003	7.31	1.1696	23.48	2.9032	11.31	C35
0.23	0.550288	0.483666	5.055	0.55605	22.57	1.3926	5.555	C35
0.24	0.742397	0.392764	5.55	0.6105	23.04	1.4399	6.05	C35
0.35	0.859847	0.342401	5.055	0.55605	23.23	1.4278	5.555	C35
0.68	0.77231	0.379556	2.75	0.4125	21.69	1.86825	3.75	L72
0.9	0.865991	0.339877	2.875	0.5175	21.7	2.3085	4.375	L72
0.86	1.0361	0.274425	2.875	0.5175	21.45	2.2527	4.375	L72
1.09	1.45363	0.149624	2.875	0.5175	21.37	2.0727	4.375	L72
0.96	0.228073	0.658584	1.5	0.15	20.8	0.9085	1.5	C39
1.09	0.359223	0.584137	1.5	0.15	20.93	0.9035	1.5	C39
1.03	0.670689	0.425472	1.5	0.15	21.38	0.9555	1.5	C39
0.68	0.884532	0.332329	1.5	0.15	21.43	1.021	1.5	C39
0.64	0.99021	0.291239	1.5	0.15	21.51	1.0165	1.5	C39
0.76	1.09808	0.2527	2.5	0.25	21.53	1.0535	2.5	C39
0.66	1.35383	0.174931	3.1	0.372	21.58	1.4652	3.6	C39
0.96	1.39372	0.164486	2.165	0.28145	21.23	1.27985	2.665	C37
1.18	1.51177	0.136132	2.335	0.39695	21.22	1.75865	3.335	C37
0.43	1.11667	0.246407	5.055	0.55605	23.38	1.41735	5.555	C35
0.49	1.27316	0.197424	7.31	1.1696	23.48	2.9032	11.31	C35
0.49	1.22924	0.210452	7.31	1.1696	23.48	2.9032	11.31	C35
0.49	1.43422	0.154331	7.31	1.1696	23.48	2.9032	11.31	C35
0.45	0.500918	0.508689	2	0.2	21.19	0.9805	2	C35
0.47	0.563012	0.477325	2	0.2	21.32	0.9525	2	C35
0.45	0.607711	0.455406	2	0.2	21.46	0.9525	2	C35
0.41	0.866178	0.339801	2	0.2	22.22	0.928	2	C35
0.48	0.983628	0.293702	2	0.2	22.24	0.928	2	C35
1.18	1.51177	0.136132	2.335	0.39695	21.22	1.79605	3.335	C37
0.81	-0.28168	0.98543	2.165	0.28145	20.83	1.36305	2.665	G60

0.93	-0.17601	0.91321	2.165	0.28145	20.58	1.36305	2.665	G60
0.33	0.320762	0.605521	1.5	0.15	22.16	0.952	1.5	I65
1.37	0.425842	0.548004	2.5	0.5	21.34	2.291	4	A03
1.39	0.618475	0.45021	2.875	0.5175	21.36	2.1069	4.375	A03
1.11	0.891198	0.32964	2.75	0.4125	21.4	1.7385	3.75	A03
1.06	1.03207	0.275875	3.4	0.612	21.64	2.3562	5.4	A03
0.88	1.24038	0.207096	3.2	0.448	22.21	1.7647	4.2	A03
0.84	1.44118	0.15263	3.2	0.448	22.55	1.8039	4.2	A03
0.87	1.57823	0.1218	2.75	0.4125	22.74	1.85775	3.75	A03
0.73	0.613363	0.452674	2.5	0.5	22.09	2.304	4	C36
0.75	0.907562	0.323094	3.7	0.888	22.62	3.5784	7.2	C36
0.69	1.0281	0.277312	4.085	0.8987	22.78	3.3033	7.585	C36
0.67	1.31549	0.18539	5.445	1.03455	22.85	3.2794	9.445	C36
0.69	1.0281	0.277312	4.085	0.8987	22.78	3.3033	7.585	C36
0.67	1.31549	0.18539	5.445	1.03455	22.85	3.2794	9.445	C36
0.61	1.5331	0.131405	2.165	0.28145	22.59	1.3442	2.665	C39
0.8	-0.40605	1.07309	2.5	0.25	21.52	1.064	2.5	I64
0.72	-0.22144	0.943997	2.5	0.25	21.77	1.064	2.5	I64
1.02	0.774879	0.378434	4.565	0.50215	23.31	1.4872	5.065	I64
0.74	0.759147	0.385336	1	0.1	23.14	0.9435	1	I64
0.89	0.929041	0.314621	3.3	0.528	23.37	2.0752	4.8	I64
0.86	1.07092	0.26208	2.5	0.25	23.31	1.13	2.5	I64
0.89	1.10239	0.251232	3.3	0.528	23.37	2.1088	4.8	I64
1.02	1.33964	0.178754	4.565	0.50215	23.31	1.50755	5.065	I64
0.84	0.886524	0.331524	1.5	0.15	21.72	0.822	1.5	C38
0.9	0.992203	0.290496	1.5	0.15	21.89	0.819	1.5	C38
0.95	1.10008	0.25202	1.5	0.15	22.1	0.8165	1.5	C38
0.9	1.36848	0.171042	2.5	0.25	22.36	0.922	2.5	C38
0.53	1.25417	0.202991	1.75	0.2625	21.34	1.72575	2.25	C38
0.34	0.337065	0.596411	3.4	0.612	22.45	2.3265	5.4	C39
0.46	0.476272	0.521429	5.43	2.1177	22.6	8.6151	15.43	C39
0.67	0.077588	0.749156	2.165	0.28145	21.94	1.4196	2.665	C34
0.58	0.234649	0.65475	2.625	0.34125	22.11	1.46445	3.125	C34
0.53	0.409426	0.5568	3.1	0.372	22.62	1.3434	3.6	C34
0.55	0.202495	0.6736	3.1	0.372	22.76	1.3434	3.6	C34
0.45	0.452637	0.533799	3.2	0.448	23.2	1.6681	4.2	C34
0.35	0.493866	0.512317	3.665	0.47645	23.26	1.7264	4.665	C34
0.41	0.671728	0.424988	4.875	0.8775	23.32	2.8962	7.875	C34
0.47	0.986118	0.292769	4.69	0.6566	23.38	2.044	6.19	C34
0.47	0.163139	0.697011	6.68	1.6032	22.52	5.2488	14.18	I65
0.46	0.340691	0.594394	7.845	2.11815	22.64	7.1712	18.845	I65
0.36	0.624542	0.447297	11	2.2	23.05	5.888	21	I65
0.34	0.64602	0.437064	12.895	2.3211	23.11	5.6187	22.395	I65
0.55	0.879498	0.334369	3.1	0.372	22.34	1.3428	3.6	C34
0.53	1.04997	0.269465	3.1	0.372	22.62	1.3434	3.6	C34
0.55	0.494642	0.511918	3.1	0.372	22.76	1.3434	3.6	C34
0.45	0.62064	0.44917	3.2	0.448	23.2	1.6681	4.2	C34
0.35	0.725569	0.400307	3.665	0.47645	23.26	1.7264	4.665	C34
0.41	0.854962	0.344415	4.875	0.8775	23.32	2.8962	7.875	C34
0.41	0.934977	0.312304	4.875	0.8775	23.32	2.8962	7.875	C34
0.47	1.15721	0.233033	4.69	0.6566	23.38	2.044	6.19	C34

0.47	-0.08386	0.852027	6.68	1.6032	22.52	5.2488	14.18	I65
0.46	0.167376	0.694473	7.845	2.11815	22.64	7.1712	18.845	I65
0.36	0.434122	0.543594	11	2.2	23.05	5.988	21	I65
0.79	0.441325	0.539773	1.5	0.15	20.86	0.8155	1.5	C34
0.82	0.621298	0.448853	1.5	0.15	20.96	0.8305	1.5	C34
0.5	0.875029	0.336185	2	0.2	21.55	0.9055	2	C34
0.41	1.0349	0.274857	2	0.2	21.61	0.9055	2	C34
0.58	1.17357	0.227772	2.335	0.39695	21.86	1.79605	3.335	C34
0.54	1.38703	0.166205	2.75	0.4125	21.9	1.7235	3.75	C34
0.53	1.53674	0.130612	3.2	0.448	21.98	1.6772	4.2	C34
0.34	1.19732	0.220273	12.895	2.3211	23.11	5.3487	22.395	I65
2.07	0.787646	0.372885	3.2	0.448	22.8	1.6233	4.2	C39
1.5	0.936909	0.311552	3.2	0.448	22.9	1.6191	4.2	C39
1.64	1.09604	0.253399	3.665	0.47645	23.2	1.6133	4.665	C39
1.79	1.28418	0.194242	3.665	0.47645	23.35	1.6146	4.665	C39
0.53	1.36448	0.172098	3.2	0.448	21.98	1.6072	4.2	C34
0.54	1.09334	0.254323	2.75	0.4125	21.9	1.6485	3.75	C34
0.3	1.24995	0.20424	1.5	0.15	21.98	1.013	1.5	I64
0.66	0.741112	0.393337	6.32	1.0112	22.27	2.812	9.82	I64
0.57	0.884045	0.332526	6.835	1.16195	22.62	3.1807	10.835	I64
0.51	1.0495	0.26963	6.275	0.94125	22.72	2.58675	9.275	I64
0.39	1.23765	0.207916	7.23	1.0845	22.91	2.76375	10.23	I64
0.63	1.1319	0.241327	5.69	1.9346	24.36	6.7813	15.19	C39
1.23	1.11203	0.247967	1.5	0.15	22.23	1.0255	1.5	C32
0.54	-0.08899	0.855385	5.5	2.2	23.28	7.758	16	L72
0.93	0.18822	0.682049	6.145	3.25685	23.4	14.5114	21.145	L72
1.21	0.24364	0.649524	6.085	3.7727	23.81	17.0934	21.585	L72
1.46	0.346716	0.59105	6.285	3.5196	24.21	16.1812	22.285	L72
1.55	0.397028	0.56349	6.25	4.0625	24.47	18.8727	22.75	L72
1.53	0.612316	0.453179	6.285	3.5196	24.58	16.3016	22.285	L72
1.54	0.674539	0.423679	6.215	3.3561	24.49	15.4818	21.715	L72
1.69	0.913906	0.320577	5.835	3.32595	24.46	15.333	19.835	L72
0.57	1.30388	0.188642	3.6	0.792	23.5	2.9502	6.6	C39
0.9	0.404598	0.5594	1	0.1	20	0.8445	1	I65
0.82	0.616373	0.451222	1	0.1	20.01	0.8725	1	I65
0.27	0.776604	0.377681	1.5	0.15	22.18	0.942	1.5	I65
0.32	0.737348	0.395018	1.5	0.15	22.3	0.942	1.5	I65
0.69	0.301852	0.616173	1	0.1	20.33	0.6145	1	C35
0.62	0.439332	0.540828	1	0.1	20.62	0.6145	1	C35
0.57	0.619305	0.449811	1	0.1	20.96	0.69	1	C35
0.37	1.0329	0.275575	1	0.1	21.79	0.7965	1	C35
0.42	1.21589	0.214525	1	0.1	21.91	0.8075	1	C35
0.44	1.41669	0.15867	1	0.1	21.78	0.881	1	C35
0.39	1.56006	0.125603	1.5	0.15	21.78	0.931	1.5	C35
2.13	0.920969	0.317789	4.355	0.74035	23.45	2.6503	6.855	C32
2.36	0.938879	0.310786	4.085	0.8987	23.68	3.5618	7.585	C32
1.88	1.23522	0.208646	7.31	1.1696	23.92	3.2504	11.31	C32
0.45	0.367193	0.579754	1.5	0.15	23.66	0.978	1.5	C35
0.44	0.456366	0.531837	1.5	0.15	23.65	0.9115	1.5	C35
0.4	0.67866	0.421764	2	0.2	23.56	0.9345	2	C35
0.38	0.892502	0.329115	2.5	0.25	23.52	0.9715	2.5	C35

0.4	0.99818	0.288274	3	0.3	23.45	1.0005	3	C35
0.42	1.10605	0.249988	3	0.3	23.46	0.988	3	C35
0.44	1.37446	0.169473	3.5	0.35	23.54	1.0985	3.5	C35
1.82	0.076004	0.750137	2.165	0.28145	22.41	1.5561	2.665	C32
1.92	0.13965	0.711158	2.165	0.28145	22.66	1.5496	2.665	C32
1.55	0.288838	0.623555	2.625	0.34125	22.59	1.60225	3.125	C32
1.9	0.419989	0.551132	3.2	0.448	22.71	1.8655	4.2	C32
1.55	0.599961	0.459166	3.2	0.448	22.83	1.8788	4.2	C32
1.56	0.853693	0.34494	3.2	0.448	22.94	1.8858	4.2	C32
1.63	1.00723	0.28493	3.75	0.5625	23.27	2.1705	5.25	C32
0.27	0.714386	0.405365	1.5	0.15	20.71	1.033	1.5	B11
0.23	0.89981	0.326185	1.5	0.15	20.63	1.0325	1.5	B11
0.25	1.01483	0.282141	2	0.4	20.43	2.337	3	B11
0.44	1.56006	0.125603	3.5	0.35	23.54	1.0985	3.5	C35
0.49	1.37446	0.169473	2	0.2	21.09	0.841	2	C35
0.73	0.347803	0.590447	1.5	0.15	22.24	1.065	1.5	C26
0.71	0.51474	0.501616	1.5	0.15	22.5	1.086	1.5	C26
0.71	0.675786	0.423099	1	0.1	22.5	1.0705	1	C26
0.75	0.394213	0.565015	1	0.1	21.67	1.0665	1	C26
0.86	0.554091	0.481766	1	0.1	21.65	1.0665	1	C26
0.79	0.640298	0.439778	1	0.1	21.85	1.0665	1	C26
0.63	0.856325	0.343853	1	0.1	21.87	1.0665	1	C26
0.73	0.950575	0.306264	1.5	0.15	22.24	1.1165	1.5	C26
0.79	1.12622	0.243212	1.5	0.15	22.43	1.1165	1.5	C26
0.79	0.964889	0.300784	3.2	0.448	23.78	1.7563	4.2	C39
0.69	1.07276	0.261438	3.2	0.448	24.04	1.7528	4.2	C39
0.6	1.34117	0.178339	3.2	0.448	24.26	1.5687	4.2	C39
0.47	1.04146	0.272501	1	0.1	21.4	0.962	1	C29
0.39	1.2268	0.211194	1	0.1	21.69	0.9785	1	C29
0.34	1.48416	0.142426	1	0.1	21.75	0.9995	1	C29
0.84	-0.02493	0.813819	1.5	0.15	21.52	0.696	1.5	C30
0.78	0.134503	0.714276	1.5	0.15	21.79	0.6565	1.5	C30
0.71	0.328144	0.601388	1.5	0.15	21.86	0.848	1.5	C30
0.7	0.386243	0.569343	1.5	0.15	22.05	0.8445	1.5	C30
0.6	0.546121	0.485752	1.5	0.15	22.31	0.8025	1.5	C30
0.55	0.632328	0.443572	2	0.2	22.36	0.931	2	C30
0.47	0.848355	0.347151	2	0.2	22.27	0.9665	2	C30
0.59	0.942605	0.309341	2	0.2	22.29	1.017	2	C30
0.77	1.11825	0.245876	1	0.1	22.36	0.8695	1	C30
0.55	0.566581	0.475554	2	0.2	22.36	0.881	2	C30
0.47	0.75869	0.385538	2	0.2	22.27	0.9165	2	C30
0.59	0.87614	0.335733	2	0.2	22.29	0.9165	2	C30
2.17	1.19147	0.222103	3.1	0.372	22.67	1.3272	3.6	C22
1.63	1.39707	0.163629	3.1	0.372	22.6	1.3344	3.6	C22
2.17	1.01812	0.280937	3.1	0.372	22.67	1.3272	3.6	C22
1.63	1.27648	0.196461	3.1	0.372	22.6	1.3344	3.6	C22
0.35	0.815917	0.360768	1.5	0.15	23.07	1.0395	1.5	I65
0.45	0.928589	0.314798	2	0.2	23.31	1.074	2	I65
0.55	1.0987	0.252491	2	0.2	23.45	1.0825	2	I65
0.57	1.22897	0.210535	2.625	0.34125	23.72	1.52555	3.125	I65
0.77	0.134415	0.714329	1.5	0.15	20.58	0.8245	1.5	I64

0.74	0.290747	0.62247	1.5	0.15	20.52	0.8245	1.5	I64
0.74	0.406591	0.558326	1.5	0.15	20.4	0.8245	1.5	I64
0.98	-0.03309	0.819068	21.56	2.3716	23.99	3.4265	24.06	C39
0.93	0.195588	0.677682	22.525	2.2525	24.11	3.0615	23.525	C39
0.99	2.79027	0.008155	12.5	1.25	23.46	1.9125	12.5	C39
1.02	2.71522	0.010033	18.03	1.9833	23.78	2.81875	19.03	C39
0.98	1.1737	0.227728	21.56	2.3716	23.99	3.36875	24.06	C39
0.93	1.34028	0.178581	22.525	2.2525	24.11	3.0115	23.525	C39
0.99	0.28763	0.624243	12.5	1.25	23.46	1.9125	12.5	C39
1.02	0.477843	0.520612	18.03	1.9833	23.78	2.8259	19.03	C39
0.98	0.717381	0.404007	21.56	2.3716	23.99	3.4265	24.06	C39
0.93	0.732528	0.397177	22.525	2.2525	24.11	3.1115	23.525	C39
0.61	0.537909	0.489877	7.23	1.0845	22.88	2.6655	10.23	C36
0.48	0.70838	0.408096	7.23	1.0845	22.85	2.6655	10.23	C36
0.39	0.928821	0.314707	7.68	1.0752	22.83	2.5242	10.18	C36
1.03	0.548648	0.484486	7	0.7	23.17	1.311	7	C39
0.99	0.740757	0.393495	12.5	1.25	23.46	1.9125	12.5	C39
1.02	0.851877	0.345691	18.03	1.9833	23.78	2.81875	19.03	C39
0.98	0.969327	0.299098	21.56	2.3716	23.99	3.36875	24.06	C39
0.93	1.17257	0.228091	22.525	2.2525	24.11	3.1115	23.525	C39
0.97	0.370265	0.578068	3.5	0.35	22.96	1.058	3.5	C39
0.91	0.499059	0.509644	4.07	0.4477	23.26	1.2738	4.57	C39
0.83	0.64347	0.438272	4.69	0.6566	23.6	1.8312	6.19	C39
0.74	0.74826	0.390155	5.28	0.8448	23.68	2.3328	7.78	C39
0.91	0.762324	0.383936	4.875	0.8775	23.85	2.6244	7.875	C39
1.03	0.911588	0.321495	4.875	0.8775	24	2.6244	7.875	C39
0.84	1.08338	0.257752	4.75	0.7125	24.12	2.037	6.75	C39
1.08	1.25886	0.201605	4.355	0.74035	24.27	2.3086	6.855	C39
1.02	1.02398	0.278804	18.03	1.9833	23.78	2.8259	19.03	C39
0.98	1.24444	0.20588	21.56	2.3716	23.99	3.4265	24.06	C39
0.93	1.42013	0.157812	22.525	2.2525	24.11	3.1115	23.525	C39
1.02	0.9559	0.304218	18.03	1.9833	23.78	2.8259	19.03	C39
0.98	1.1087	0.249093	21.56	2.3716	23.99	3.3759	24.06	C39
0.93	1.30317	0.188839	22.525	2.2525	24.11	3.019	23.525	C39
1.02	0.304528	0.61466	18.03	1.9833	23.78	2.8204	19.03	C39
0.98	0.526961	0.495406	21.56	2.3716	23.99	3.4265	24.06	C39
0.93	0.602434	0.457964	22.525	2.2525	24.11	3.065	23.525	C39
0.98	0.761373	0.384355	21.56	2.3716	23.99	3.4265	24.06	C39
0.93	0.975493	0.296764	22.525	2.2525	24.11	3.1115	23.525	C39
0.99	0.410874	0.556021	12.5	1.25	23.46	1.9125	12.5	C39
1.02	0.543577	0.487028	18.03	1.9833	23.78	2.8149	19.03	C39
0.98	0.776635	0.377668	21.56	2.3716	23.99	3.421	24.06	C39
0.93	1.04884	0.269865	22.525	2.2525	24.11	3.111	23.525	C39
0.98	0.567163	0.475266	21.56	2.3716	23.99	3.4265	24.06	C39
0.93	0.773785	0.378911	22.525	2.2525	24.11	3.1115	23.525	C39
0.98	0.994566	0.289617	21.56	2.3716	23.99	3.4265	24.06	C39
0.93	1.25926	0.201489	22.525	2.2525	24.11	3.1115	23.525	C39
0.7	0.525319	0.496238	4.69	0.6566	22.75	1.9418	6.19	C38
0.61	0.711097	0.406859	4.75	0.7125	23.4	2.19975	6.75	C38
0.54	0.822218	0.358099	4.285	0.6856	23.56	2.2664	6.285	C38
0.54	0.939668	0.31048	5.28	0.8448	23.4	2.5064	7.78	C38

0.57	1.11758	0.246099	5.445	1.03455	23.35	3.27655	9.445	C38
1.02	0.496564	0.510928	18.03	1.9833	23.78	2.8259	19.03	C39
0.98	0.706078	0.409146	21.56	2.3716	23.99	3.4265	24.06	C39
0.93	1.00148	0.287053	22.525	2.2525	24.11	3.1115	23.525	C39
0.98	0.255525	0.642647	21.56	2.3716	23.99	3.4815	24.06	C39
0.93	0.418916	0.551706	22.525	2.2525	24.11	3.1115	23.525	C39
0.99	0.387708	0.568546	12.5	1.25	23.46	1.9125	12.5	C39
1.02	0.54162	0.488011	18.03	1.9833	23.78	2.8259	19.03	C39
0.98	0.653523	0.433521	21.56	2.3716	23.99	3.4265	24.06	C39
0.93	0.815083	0.361122	22.525	2.2525	24.11	3.1185	23.525	C39
1.02	0.98388	0.293608	18.03	1.9833	23.78	2.8765	19.03	C39
0.98	1.09175	0.254866	21.56	2.3716	23.99	3.42595	24.06	C39
0.93	1.36649	0.171567	22.525	2.2525	24.11	3.1115	23.525	C39
1.02	1.09846	0.25257	18.03	1.9833	23.78	2.9315	19.03	C39
0.98	1.16792	0.22958	21.56	2.3716	23.99	3.4265	24.06	C39
0.93	1.37984	0.168069	22.525	2.2525	24.11	3.0695	23.525	C39
0.67	0.65373	0.433423	5.8	0.928	22.62	2.588	8.8	C36
0.69	0.761015	0.384513	5.8	0.928	22.84	2.6056	8.8	C36
0.61	0.92566	0.315946	7.23	1.0845	22.88	2.66925	10.23	C36
0.48	0.975222	0.296866	7.23	1.0845	22.85	2.6685	10.23	C36
0.39	1.23991	0.207236	7.68	1.0752	22.83	2.5242	10.18	C36
0.67	0.638685	0.440544	5.8	0.928	22.62	2.588	8.8	C36
0.69	0.852527	0.345422	5.8	0.928	22.84	2.6056	8.8	C36
0.61	0.964536	0.300919	7.23	1.0845	22.88	2.6655	10.23	C36
0.48	1.07241	0.261561	7.23	1.0845	22.85	2.64825	10.23	C36
0.39	1.34715	0.176724	7.68	1.0752	22.83	2.4899	10.18	C36
0.91	0.425032	0.548436	4.07	0.4477	23.26	1.3101	4.57	C39
0.83	0.586013	0.465976	4.69	0.6566	23.6	1.9278	6.19	C39
0.74	0.827084	0.356045	5.28	0.8448	23.68	2.4432	7.78	C39
0.91	0.974291	0.297218	4.875	0.8775	23.85	2.8008	7.875	C39
1.03	1.15727	0.233011	4.875	0.8775	24	2.8134	7.875	C39
0.84	1.37707	0.168791	4.75	0.7125	24.12	2.21025	6.75	C39
1.08	1.50778	0.137028	4.355	0.74035	24.27	2.50495	6.855	C39
0.72	0.483088	0.517889	1.75	0.2625	22.61	0.86025	2.25	C26
0.7	0.749885	0.389433	4.69	0.6566	22.75	1.9418	6.19	C38
0.61	0.822123	0.358139	4.75	0.7125	23.4	2.1555	6.75	C38
0.54	0.928465	0.314847	4.285	0.6856	23.56	2.2608	6.285	C38
0.54	1.09857	0.252533	5.28	0.8448	23.4	2.5544	7.78	C38
0.57	1.22884	0.210573	5.445	1.03455	23.35	3.31835	9.445	C38
0.7	0.64736	0.43643	4.69	0.6566	22.75	1.9418	6.19	C38
0.61	0.854872	0.344453	4.75	0.7125	23.4	2.19975	6.75	C38
0.54	0.95422	0.304863	4.285	0.6856	23.56	2.2608	6.285	C38
0.54	1.06209	0.265176	5.28	0.8448	23.4	2.4584	7.78	C38
0.57	1.31151	0.186501	5.445	1.03455	23.35	3.26135	9.445	C38
0.54	0.469524	0.524945	1.5	0.15	20.76	0.934	1.5	I65
0.54	0.632926	0.443287	1.5	0.15	21.04	0.934	1.5	I65
0.26	0.907374	0.323168	2.625	0.34125	22.2	1.4092	3.125	I65
0.54	1.37273	0.169926	5.28	0.8448	23.4	2.4744	7.78	C38
0.57	1.49711	0.139447	5.445	1.03455	23.35	3.22335	9.445	C38
0.26	0.909598	0.322285	2.625	0.34125	22.2	1.45145	3.125	I65
0.31	1.07971	0.259023	2.165	0.28145	21.88	1.37865	2.665	I65

0.4	1.22897	0.210535	2.165	0.28145	21.84	1.37865	2.665	I65
0.97	0.440464	0.540229	3.5	0.35	22.96	1.058	3.5	C39
0.91	0.645908	0.437117	4.07	0.4477	23.26	1.3101	4.57	C39
0.83	0.787148	0.373101	4.69	0.6566	23.6	1.9278	6.19	C39
0.74	0.735231	0.395966	5.28	0.8448	23.68	2.4432	7.78	C39
0.91	0.801139	0.367073	4.875	0.8775	23.85	2.7486	7.875	C39
1.03	0.913812	0.320614	4.875	0.8775	24	2.6253	7.875	C39
0.84	1.10291	0.251056	4.75	0.7125	24.12	2.037	6.75	C39
1.08	1.23951	0.207356	4.355	0.74035	24.27	2.3086	6.855	C39
0.19	0.750669	0.389085	3.2	0.448	22.63	1.8557	4.2	N77
0.15	1.01301	0.282806	3.3	0.528	22.77	2.252	4.8	N77
0.13	1.0888	0.255883	2.75	0.4125	22.85	1.92825	3.75	N77
0.13	1.29439	0.191325	2.75	0.4125	22.76	1.94325	3.75	N77
0.64	0.81247	0.362233	1	0.1	21.31	0.9795	1	C39
0.48	0.773214	0.379161	1	0.1	21.85	0.9795	1	C39
0.51	0.851782	0.34573	1.5	0.15	21.95	1.0295	1.5	C39
0.5	0.920142	0.318115	2.335	0.39695	22.36	1.9601	3.335	C39
0.43	1.11557	0.246776	2.165	0.28145	22.46	1.43	2.665	C39
0.49	1.26483	0.199852	2.165	0.28145	22.58	1.43	2.665	C39
0.5	0.920142	0.318115	2.335	0.39695	22.36	2.0026	3.335	C39
0.43	1.11557	0.246776	2.165	0.28145	22.46	1.4885	2.665	C39
0.49	1.26483	0.199852	2.165	0.28145	22.58	1.49565	2.665	C39
1.3	1.55409	0.126873	2.5	0.25	22.75	0.9775	2.5	C38
1.3	0.651875	0.434297	2.5	0.25	22.75	0.9775	2.5	C38
0.42	0.751892	0.388543	2.335	0.39695	20.76	1.84535	3.335	C34
0.41	0.803939	0.365873	2.165	0.28145	20.75	1.34615	2.665	C34
0.42	1.07435	0.260885	2.875	0.5175	21.7	2.1339	4.375	C34
0.43	1.23084	0.209968	3.125	0.71875	21.73	2.95665	5.625	C34
0.52	0.611613	0.453519	2.165	0.28145	20.74	1.35005	2.665	C34
0.42	0.960216	0.302567	2.875	0.5175	21.7	2.1393	4.375	C34
0.43	1.18692	0.223535	3.125	0.71875	21.73	2.9739	5.625	C34
0.74	0.277994	0.62974	1	0.1	22.32	0.9815	1	C36
0.7	0.501487	0.508397	1	0.1	22.56	0.9815	1	C36
0.64	0.574164	0.471804	1	0.1	22.49	0.9815	1	C36
0.54	0.780167	0.37613	1	0.1	22.5	1.0305	1	C36
0.43	0.88884	0.33059	4.785	1.2441	22.85	4.6787	10.285	C24
0.37	1.0543	0.267928	4.715	1.1316	22.62	4.2048	9.715	C24
0.42	1.24877	0.20459	4.645	1.06835	22.55	3.9146	9.145	C24
1.08	1.39777	0.163449	2	0.2	23.83	1.0775	2	C30
1.08	0.667816	0.426814	2	0.2	23.83	1.0775	2	C30
0.92	1.12273	0.244378	1	0.1	23.63	1.022	1	C30
0.99	1.19218	0.221882	1.5	0.15	23.71	1.0375	1.5	C30
0.49	1.27015	0.198299	2.625	0.34125	22.71	1.35785	3.125	C22
0.64	0.125983	0.719449	6.665	2.86595	23.21	12.0035	21.665	C38
0.59	0.147942	0.706149	7.5	3.75	23.45	16.7	27.5	C38
0.57	0.238989	0.652225	7.75	2.7125	23.56	10.29	22.75	C38
0.63	0.281032	0.628004	8.27	2.8945	23.78	10.99	24.77	C38
0.49	0.175751	0.689468	9.285	4.2711	23.94	18.5863	34.285	C38
0.68	0.43804	0.541513	9.355	4.39685	24.18	19.505	35.355	C38
0.58	0.579784	0.469035	10	4	23.97	16.164	34	C38
0.5	0.679958	0.421162	11.355	6.47235	23.79	32.5983	51.355	C38

0.59	0.401015	0.561334	7.5	3.75	23.45	16.725	27.5	C38
0.57	0.656203	0.432259	7.75	2.7125	23.56	10.3127	22.75	C38
0.63	0.616947	0.450946	8.27	2.8945	23.78	11.0128	24.77	C38
0.49	0.625881	0.446655	9.285	4.2711	23.94	18.6139	34.285	C38
0.68	0.732223	0.397315	9.355	4.39685	24.18	19.3875	35.355	C38
0.58	0.946643	0.30778	10	4	23.97	16.09	34	C38
0.5	0.988289	0.291957	11.355	6.47235	23.79	32.3332	51.355	C38
2.63	0.132368	0.71557	7.05	2.8905	23.66	11.6133	22.55	C13
0.63	0.529467	0.494138	8.27	2.8945	23.78	10.976	24.77	C38
0.49	0.567118	0.475288	9.285	4.2711	23.94	18.5702	34.285	C38
0.68	0.719103	0.403228	9.355	4.39685	24.18	19.505	35.355	C38
0.58	0.812977	0.362017	10	4	23.97	16.17	34	C38
0.5	0.96372	0.30123	11.355	6.47235	23.79	32.5983	51.355	C38
0.49	0.242095	0.650421	9.285	4.2711	23.94	18.5012	34.285	C38
0.68	0.270662	0.633937	9.355	4.39685	24.18	19.364	35.355	C38
0.58	0.52449	0.496659	10	4	23.97	16.084	34	C38
0.5	0.705941	0.409208	11.355	6.47235	23.79	32.4102	51.355	C38
0.5	1.04975	0.269543	11.355	6.47235	23.79	32.5983	51.355	C38
0.37	1.46125	0.147804	4.43	0.8417	21.79	2.7265	7.43	I64
0.43	-0.00424	0.800588	1.5	0.15	21.4	1.0105	1.5	I65
2.8	0.42459	0.548672	4.57	0.9597	22.76	2.856	8.57	C13
3.23	0.59624	0.460977	4.93	1.4297	22.83	5.07645	11.43	C13
2.94	0.778431	0.376886	5.215	1.7731	23.05	6.7728	13.715	C13
2.77	0.884109	0.3325	6.22	2.1148	23.3	7.837	17.22	C13
2.76	0.954001	0.304947	6.5	2.6	23.58	10.194	20	C13
2.63	1.21608	0.214467	7.05	2.8905	23.66	11.6522	22.55	C13
2.77	0.998693	0.288084	6.22	2.1148	23.3	8.007	17.22	C13
2.76	1.03016	0.276565	6.5	2.6	23.58	10.214	20	C13
2.63	1.22943	0.210397	7.05	2.8905	23.66	11.6747	22.55	C13
2.98	0.244754	0.648878	3.25	0.8125	22.54	2.975	6.25	C13
2.65	0.388565	0.56808	3.7	0.888	22.72	3.096	7.2	C13
2.8	0.587529	0.465234	4.57	0.9597	22.76	3.0345	8.57	C13
3.23	0.790617	0.371601	4.93	1.4297	22.83	5.00395	11.43	C13
2.94	0.918833	0.318631	5.215	1.7731	23.05	6.5467	13.715	C13
2.77	1.10182	0.251428	6.22	2.1148	23.3	7.837	17.22	C13
2.76	1.26464	0.199909	6.5	2.6	23.58	10.254	20	C13
2.63	1.40168	0.162454	7.05	2.8905	23.66	11.5353	22.55	C13
0.33	0.30596	0.613851	1.5	0.15	23.14	0.9535	1.5	E50
0.34	0.472897	0.523186	2	0.2	23.2	1.0035	2	E50
0.34	0.633942	0.442802	2	0.2	23.18	1.0035	2	E50
0.73	0.935229	0.312206	2.97	0.5643	21.89	2.2268	2.97	C38
0.73	0.896353	0.327569	2.97	0.5643	21.89	2.2268	2.97	C38
0.55	1.33588	0.179776	3.3	0.528	24.01	2.1568	4.8	C42
0.29	0.748474	0.39006	1.39	0.2502	22.01	1.6947	1.39	C26
0.33	0.820712	0.358736	1.94	0.3686	22.55	1.88385	1.94	C26
0.27	0.933384	0.312925	1.94	0.3686	22.57	1.8753	1.94	C26
0.39	1.09716	0.253015	2.495	0.499	22.98	2.01	2.495	C26
0.34	1.24009	0.207182	4.035	0.84735	21.18	2.6355	5.035	C26
0.39	1.07763	0.259744	2.495	0.499	22.98	2.286	2.495	C26
0.34	1.25944	0.201434	4.035	0.84735	21.18	2.9253	5.035	C26
0.34	1.32276	0.183376	4.035	0.84735	21.18	3.01665	5.035	C26

0.14	0.916634	0.319499	1	0.1	23.08	1.035	1	L72
0.65	1.27984	0.195491	1.5	0.15	22.39	1.0775	1.5	C41
2.54	0.857338	0.343435	2.165	0.28145	24.51	1.6484	2.665	G60
1.36	1.34796	0.176505	2	0.2	25.25	1.171	2	G60
0.75	0.072011	0.752613	1.375	0.2475	20.94	1.6659	1.375	C26
0.67	0.178792	0.687655	1.365	0.23205	21.04	1.55805	1.365	C26
0.47	0.524598	0.496603	1.37	0.2329	21.08	1.564	1.37	C26
0.71	0.61682	0.451007	1.465	0.27835	21.37	1.9266	1.465	C27
0.98	1.27918	0.195682	2	0.2	22.3	1.0505	2	C29
0.58	0.956005	0.304178	3.97	0.7543	23.51	2.413	3.97	I65
0.45	-0.97453	1.50477	1.415	0.2547	21.15	1.8243	1.915	C39
0.46	-0.81702	1.38063	1.42	0.2556	21.26	1.8243	1.92	C39
0.42	-0.7165	1.30309	1.43	0.2717	21.58	1.92565	1.93	C39
0.38	-0.56091	1.18593	1.415	0.2547	21.73	1.8243	1.915	C39
0.53	0.957187	0.303725	2.165	0.28145	22	1.3845	2.665	C32
0.49	1.10999	0.248658	2.165	0.28145	22.11	1.3845	2.665	C32
0.47	1.29813	0.190264	2.165	0.28145	22.03	1.3845	2.665	C32
0.36	1.05336	0.268259	5.565	1.16865	23.26	3.4671	6.915	C35
0.34	0.888031	0.330916	2.505	0.501	22.84	2.309	2.505	C39
0.25	0.931262	0.313753	3.53	0.7413	22.99	2.793	4.03	C39
0.17	1.18329	0.224682	4.08	0.8976	22.87	3.1691	4.68	C39
0.76	0.749664	0.389531	3.485	0.697	23.44	2.765	4.985	C39
0.82	0.898927	0.326538	3.5	0.7	23.82	2.665	4.5	C39
0.36	1.0729	0.26139	5.565	1.16865	23.26	3.4671	6.915	C35
0.36	1.11258	0.247782	5.565	1.16865	23.26	3.4671	6.915	C35
0.25	1.06493	0.264179	3.53	0.7413	22.99	2.793	4.03	C39
0.17	1.20786	0.216997	4.08	0.8976	22.87	3.1691	4.68	C39
0.73	0.882999	0.332949	1.5	0.15	22.12	0.8345	1.5	C30
1.01	1.04287	0.271998	1.5	0.15	22.35	0.978	1.5	C30
0.98	1.22585	0.211482	1.5	0.15	22.53	0.977	1.5	C30
0.61	1.57003	0.123506	1.5	0.15	22.75	0.976	1.5	C30
0.84	-0.08529	0.852963	2.5	0.25	22.06	0.902	2.5	C17
0.81	-0.00215	0.799253	2.625	0.34125	22.11	1.2376	3.125	C17
0.34	0.492564	0.512989	7.105	1.5631	23.33	5.0765	13.105	C27
0.89	1.49512	0.139903	2.44	0.4636	22.24	2.15745	2.89	C39
1.13	1.02754	0.277515	2.835	0.76545	21.83	3.4587	5.335	C18
1.13	0.580124	0.468868	2.835	0.76545	21.83	3.4587	5.335	C18
0.22	1.02906	0.276963	2.55	0.5355	22.94	2.6124	3	I65
0.25	1.17833	0.226256	2.56	0.5376	22.93	2.5179	2.56	I65
0.47	1.0713	0.261949	2.56	0.5376	22.28	2.76255	3.61	C26
0.59	0.639689	0.440067	3.965	0.75335	22.54	2.40065	3.965	I65
0.59	1.287	0.193433	3.965	0.75335	22.54	2.40065	3.965	I65
0.82	1.09789	0.252767	18.765	4.69125	25.05	7.98125	20.515	C39
0.82	0.187826	0.682282	18.765	4.69125	25.05	7.98125	20.515	C39
0.86	-0.10905	0.868581	14.645	3.36835	24.87	6.05015	15.995	C39
0.82	0.100632	0.734944	18.765	4.69125	25.05	7.98125	20.515	C39
0.88	0.126671	0.719031	9.37	1.5929	24.45	3.21045	11.07	C39
0.89	0.295536	0.619751	12.535	2.63235	24.67	5.07045	14.835	C39
0.86	0.462323	0.528711	14.645	3.36835	24.87	6.05015	15.995	C39
0.82	0.637573	0.441073	18.765	4.69125	25.05	7.98125	20.515	C39
0.88	0.696444	0.413555	9.37	1.5929	24.45	3.21045	11.07	C39

0.89	0.788573	0.372484	12.535	2.63235	24.67	5.07045	14.835	C39
0.86	0.893363	0.328769	14.645	3.36835	24.87	6.05015	15.995	C39
0.82	1.07761	0.25975	18.765	4.69125	25.05	7.98125	20.515	C39
0.88	0.807677	0.364276	9.37	1.5929	24.45	3.21045	11.07	C39
0.89	0.960679	0.30239	12.535	2.63235	24.67	5.07045	14.835	C39
0.86	1.16848	0.229399	14.645	3.36835	24.87	6.05015	15.995	C39
0.82	1.32518	0.182709	18.765	4.69125	25.05	7.98125	20.515	C39
0.88	0.768655	0.381156	9.37	1.5929	24.45	3.21045	11.07	C39
0.89	0.892596	0.329077	12.535	2.63235	24.67	5.07045	14.835	C39
0.86	1.03273	0.275637	14.645	3.36835	24.87	6.05015	15.995	C39
0.82	1.20822	0.216887	18.765	4.69125	25.05	7.98125	20.515	C39
0.41	0.424716	0.548605	1.36	0.2312	23.61	1.54955	1.36	C27
0.41	0.582677	0.467613	1.885	0.3393	23.78	1.7703	1.885	C27
0.35	0.639832	0.439999	1.885	0.3393	23.84	1.6848	1.885	C27
0.37	0.831587	0.354151	1.865	0.31705	23.88	1.5538	1.865	C27
0.44	1.00991	0.283944	1.375	0.23375	24.06	1.53935	1.375	C27
0.56	1.19872	0.219835	2.08	0.4576	24.1	2.7016	2.58	C27
0.57	1.36808	0.171149	2.085	0.4587	24.17	2.8314	3.085	C27
0.89	0.241224	0.650926	12.535	2.63235	24.67	5.07045	14.835	C39
0.86	0.450996	0.534663	14.645	3.36835	24.87	6.05015	15.995	C39
0.82	0.507478	0.505326	18.765	4.69125	25.05	7.98125	20.515	C39
0.82	0.880537	0.333947	18.765	4.69125	25.05	7.98125	20.515	C39
0.82	1.18887	0.222921	18.765	4.69125	25.05	7.98125	20.515	C39
0.82	1.1643	0.230742	18.765	4.69125	25.05	7.98125	20.515	C39
0.88	0.423684	0.549156	9.37	1.5929	24.45	3.21045	11.07	C39
0.89	0.43326	0.544052	12.535	2.63235	24.67	5.07045	14.835	C39
0.86	0.630114	0.44463	14.645	3.36835	24.87	6.05015	15.995	C39
0.82	0.906521	0.323508	18.765	4.69125	25.05	7.98125	20.515	C39
0.89	0.025284	0.781858	12.535	2.63235	24.67	5.07045	14.835	C39
0.86	0.17956	0.687198	14.645	3.36835	24.87	6.05015	15.995	C39
0.82	0.32396	0.603729	18.765	4.69125	25.05	7.98125	20.515	C39
0.89	0.478316	0.520366	12.535	2.63235	24.67	5.07045	14.835	C39
0.86	0.577558	0.470131	14.645	3.36835	24.87	6.05015	15.995	C39
0.82	0.720127	0.402764	18.765	4.69125	25.05	7.98125	20.515	C39
0.73	-0.43958	1.09718	1.93	0.3667	22.83	1.9608	1.93	C35
0.6	-0.3666	1.04498	1.93	0.3667	23.02	1.9608	1.93	C35
0.86	0.202342	0.67369	14.645	3.36835	24.87	6.05015	15.995	C39
0.82	0.389674	0.567478	18.765	4.69125	25.05	7.98125	20.515	C39
0.88	0.840219	0.350537	9.37	1.5929	24.45	3.21045	11.07	C39
0.89	0.920576	0.317944	12.535	2.63235	24.67	5.07045	14.835	C39
0.86	1.01579	0.28179	14.645	3.36835	24.87	6.05015	15.995	C39
0.82	1.27153	0.197897	18.765	4.69125	25.05	7.98125	20.515	C39
0.82	1.28488	0.19404	18.765	4.69125	25.05	7.98125	20.515	C39
0.73	-0.05437	0.832815	2.445	0.46455	22.17	2.16505	2.895	C39
0.75	0.08547	0.744281	2.45	0.4655	22.21	2.16505	2.9	C39
0.75	0.227663	0.658824	2.435	0.46265	22.28	2.16505	2.885	C39
0.58	0.737348	0.395018	1	0.1	21.76	0.958	1	I65
0.63	0.815917	0.360768	1.5	0.15	21.9	1.008	1.5	I65
0.6	0.928589	0.314798	2	0.2	22.18	1.024	2	I65
0.54	1.0987	0.252491	2.5	0.25	22.31	1.068	2.5	I65
0.57	0.894115	0.328467	2.665	0.61295	22.79	3.04405	4.665	C33

0.69	1.08954	0.255626	6.96	1.3224	22.95	3.9045	12.46	C33
0.65	1.23881	0.207568	6.96	1.3224	23.13	3.9045	12.46	C33
0.69	1.3637	0.172304	6.96	1.3224	22.95	3.9045	12.46	C33
0.65	1.50708	0.137187	6.96	1.3224	23.13	3.9045	12.46	C33
0.45	0.601787	0.458279	1.96	0.3724	21.64	2.07955	1.96	C36
0.43	0.497652	0.510368	1.965	0.37335	21.68	2.0197	1.965	C36
0.43	0.689761	0.416629	2.465	0.46835	21.73	2.21825	2.465	C36
0.45	0.800881	0.367183	2.475	0.495	22.06	2.635	3.975	C36
0.39	0.918332	0.318829	2.485	0.497	22.14	2.635	3.985	C36
0.35	1.11524	0.246887	2.495	0.499	22.15	2.688	3.995	C36
0.7	0.791454	0.37124	1.33	0.2261	22.29	1.4994	1.33	C36
0.76	1.06646	0.263642	1.345	0.22865	22.54	1.68895	1.345	C36
0.61	1.13591	0.239998	1.855	0.31535	22.5	1.62265	1.855	C36
0.58	1.33518	0.179968	1.875	0.3375	22.48	1.7595	1.875	C36
1.22	0.741929	0.392972	2.37	0.4029	23.5	1.73145	2.37	C39
1.35	0.755994	0.386728	2.95	0.5605	23.88	2.03015	2.95	C39
1.24	0.911588	0.321495	2.915	0.5247	24.15	2.0313	3.515	C39
1.25	1.05805	0.2666	2.94	0.5586	24.36	2.14415	3.54	C39
1.22	0.728901	0.398807	2.37	0.4029	23.5	1.73145	2.37	C39
1.35	0.794809	0.369793	2.95	0.5605	23.88	2.03015	2.95	C39
1.24	0.913812	0.320614	2.915	0.5247	24.15	2.0277	3.515	C39
1.25	1.07759	0.259758	2.94	0.5586	24.36	2.1318	3.54	C39
0.95	0.728901	0.398807	3.365	0.57205	23.17	1.8989	3.365	C39
0.84	0.801139	0.367073	3.88	0.6984	23.77	2.1006	3.88	C39
0.95	0.913812	0.320614	4.395	0.7911	24.01	2.1492	4.395	C39
1.13	1.07126	0.261963	4.48	0.896	24.32	2.501	4.83	C39
1.45	1.22052	0.213106	5.475	1.095	24.62	2.991	7.275	C39
0.95	0.741929	0.392972	3.365	0.57205	23.17	1.8989	3.365	C39
0.84	0.762324	0.383936	3.88	0.6984	23.77	2.1006	3.88	C39
0.95	0.911588	0.321495	4.395	0.7911	24.01	2.1906	4.395	C39
1.13	1.05172	0.268841	4.48	0.896	24.32	2.504	4.83	C39
1.45	1.23987	0.207249	5.475	1.095	24.62	2.994	7.275	C39
0.84	0.974291	0.297218	3.88	0.6984	23.77	2.1285	3.88	C39
0.95	1.15727	0.233011	4.395	0.7911	24.01	2.2392	4.395	C39
1.13	1.34542	0.17719	4.48	0.896	24.32	2.731	4.83	C39
1.45	1.48879	0.141356	5.475	1.095	24.62	3.221	7.275	C39
1.45	1.30319	0.188836	5.475	1.095	24.62	3.213	7.275	C39
1.45	0.912189	0.321257	5.475	1.095	24.62	3.213	7.275	C39
0.22	0.504781	0.506707	1.5	0.15	22.36	0.8455	1.5	C34
0.32	0.702332	0.410857	1.5	0.15	22.44	0.9305	1.5	C34
0.31	0.958141	0.30336	1.5	0.15	22.68	0.947	1.5	C34
0.44	1.06373	0.264599	4.355	0.74035	22.24	2.5381	6.855	C34
0.46	1.33214	0.180797	4.355	0.74035	22.4	2.54575	6.855	C34
0.46	1.13822	0.239238	4.355	0.74035	22.4	2.54575	6.855	C34
0.72	0.478322	0.520363	1.5	0.15	20.83	0.875	1.5	C18
0.77	0.690097	0.416475	1.5	0.15	20.93	0.8755	1.5	C18
0.6	0.831337	0.354256	2.165	0.28145	21.23	1.43455	2.665	C18
0.43	0.792081	0.370969	2.165	0.28145	21.2	1.43455	2.665	C18
0.45	0.88964	0.330267	2	0.2	21.21	1.0535	2	C18
0.57	1.00231	0.286744	2	0.2	21.11	1.059	2	C18
0.68	1.17242	0.228138	2	0.2	20.83	0.925	2	C18

0.91	1.32168	0.183673	2	0.2	20.82	0.925	2	C18
0.6	-0.69417	1.28606	2.165	0.28145	21.23	1.43455	2.665	C18
0.43	-0.46907	1.11853	2.165	0.28145	21.2	1.43715	2.665	C18
0.45	-0.26857	0.976357	2	0.2	21.21	1.057	2	C18
0.43	0.569645	0.474037	1.97	0.3743	21.98	2.0368	1.97	C36
0.42	0.634738	0.442423	1.97	0.3743	22.04	2.03015	1.97	C36
0.57	0.735694	0.395759	1.975	0.395	22.17	2.148	1.975	C36
0.52	0.900339	0.325974	1.43	0.2717	22.7	1.9817	1.43	C36
0.38	0.93724	0.311423	2.03	0.4263	22.71	2.37405	2.03	C36
0.6	-0.69417	1.28606	2.165	0.28145	21.23	1.43455	2.665	C18
0.43	-0.46907	1.11853	2.165	0.28145	21.2	1.43715	2.665	C18
0.57	-0.13227	0.883954	2	0.2	21.11	1.059	2	C18
0.68	0.011101	0.790831	2	0.2	20.83	1.059	2	C18
0.91	0.233446	0.655451	2	0.2	20.82	1.055	2	C18
0.5	0.556459	0.480585	2.5	0.5	21.07	2.084	4	C19
0.45	0.805414	0.365242	2.665	0.61295	21.48	2.5185	4.665	C19
0.25	0.506944	0.505599	2.665	0.61295	21.56	2.5185	4.665	C19
0.21	0.444123	0.538292	2.665	0.61295	21.65	2.5185	4.665	C19
0.24	0.522691	0.497571	2.665	0.61295	21.7	2.5185	4.665	C19
0.19	0.621599	0.448708	2.665	0.61295	21.66	2.5139	4.665	C19
0.18	0.842736	0.349488	2.835	0.76545	21.65	3.0429	5.335	C19
0.68	0.827558	0.355846	2.935	0.55765	23.74	2.7683	4.935	C39
0.71	0.920576	0.317944	3.555	0.74655	23.73	3.15105	5.555	C39
0.57	1.02845	0.277184	4.06	0.8526	23.66	3.18885	6.16	C39
0.5	1.29053	0.192426	4.59	1.0098	23.5	4.7124	11.94	C39
0.68	0.794809	0.369793	2.935	0.55765	23.74	2.8633	4.935	C39
0.71	0.894821	0.328184	3.555	0.74655	23.73	3.2592	5.555	C39
0.57	1.06493	0.264179	4.06	0.8526	23.66	3.38205	6.16	C39
0.5	1.20786	0.216997	4.59	1.0098	23.5	4.8081	11.94	C39
0.57	1.04539	0.271095	4.06	0.8526	23.66	3.29385	6.16	C39
0.5	1.22721	0.211069	4.59	1.0098	23.5	4.7157	11.94	C39
2.07	0.72035	0.402663	2.165	0.28145	21.03	1.37085	2.665	I64
0.73	1.06209	0.265176	2.81	0.4496	21.99	1.6152	2.81	C38
0.68	1.32417	0.182986	3.35	0.5695	22.12	1.87255	3.35	C38
2.07	0.733378	0.396796	2.165	0.28145	21.03	1.37085	2.665	I64
0.98	0.747443	0.390518	1.75	0.2625	22.12	1.50675	2.25	I64
0.61	0.896706	0.327428	1.75	0.2625	22.53	1.50675	2.25	I64
0.37	1.01785	0.281036	2	0.4	22.53	2.109	3	I64
0.41	1.206	0.217574	2	0.4	22.07	2.109	3	I64
0.37	1.10656	0.249816	5.61	1.2342	22.49	3.8632	11.11	C34
0.68	1.50977	0.136579	3.35	0.5695	22.12	1.87255	3.35	C38
0.54	1.22251	0.212498	2.48	0.496	22.57	2.261	2.48	C38
0.55	0.814897	0.361201	3.555	0.74655	22.13	2.84235	4.055	C39
0.68	0.914245	0.320443	4.08	0.8976	22.21	3.3627	5.58	C39
0.68	0.87537	0.336046	4.08	0.8976	22.21	3.3627	5.58	C39
0.22	0.930582	0.314019	1	0.1	22.29	0.971	1	I64
0.67	0.282586	0.627117	2.165	0.28145	21.56	1.25515	2.665	C32
0.7	0.47913	0.519943	2.165	0.28145	21.41	1.25775	2.665	C32
0.75	0.718668	0.403424	2.165	0.28145	21.41	1.26165	2.665	C32
0.83	0.727485	0.399444	2.165	0.28145	21.41	1.26425	2.665	C32
1.19	1.05614	0.267275	1.415	0.2547	21.98	1.7379	1.415	C38

1.23	1.1256	0.24342	1.42	0.2556	22.15	1.7235	1.42	C38
0.76	0.694694	0.414359	1	0.1	22.62	0.552	1	C26
0.67	0.794558	0.369901	3.33	0.5661	22.95	1.2784	3.33	C26
0.84	0.962812	0.301576	2.705	0.3787	23.06	0.9828	2.705	C26
0.67	0.780941	0.375794	2.295	0.3672	22.92	1.5088	2.295	C26
0.84	0.932925	0.313104	3.33	0.5661	22.95	1.8326	3.33	C26
0.79	1.00148	0.287053	2.705	0.3787	23.06	1.4252	2.705	C26
0.85	1.25984	0.201318	2.725	0.3815	23	1.4952	2.725	C26
0.84	1.18951	0.222721	3.33	0.5661	22.95	1.8734	3.33	C26
0.79	1.4093	0.160525	2.705	0.3787	23.06	1.5127	2.705	C26
0.85	1.55268	0.127174	2.725	0.3815	23	1.5127	2.725	C26
0.9	1.11125	0.24823	1.995	0.399	22.66	2.193	1.995	C38
0.73	0.755941	0.386751	1.805	0.2888	22.06	1.4464	1.805	I65
1.18	1.01195	0.283194	2.44	0.4636	22.57	2.0748	2.44	I65
0.93	1.08141	0.258433	2.47	0.4693	22.68	2.1242	2.47	I65
0.84	1.287	0.193433	2.47	0.4693	22.76	2.1299	2.47	I65
0.22	0.832544	0.35375	1.75	0.2625	23.1	1.52025	2.25	C33
0.18	1.00184	0.28692	1.75	0.2625	23.12	1.52025	2.25	C33
0.19	1.18718	0.223455	1.75	0.2625	23.15	1.5525	2.25	C33
0.22	1.44454	0.151816	1.75	0.2625	23.19	1.5645	2.25	C33
0.73	0.077581	0.74916	1.805	0.2888	22.06	1.4464	1.805	I65
1.18	0.229061	0.658008	2.44	0.4636	22.57	2.0748	2.44	I65
0.93	0.395998	0.564048	2.47	0.4693	22.68	2.1242	2.47	I65
0.84	0.557044	0.480294	2.47	0.4693	22.76	2.1299	2.47	I65
0.76	0.371179	0.577568	3	0.3	21.31	0.8145	3	C33
0.55	0.964183	0.301053	4.815	0.7704	22.84	2.504	7.315	C33
1.18	0.588105	0.464952	2.44	0.4636	22.57	2.0748	2.44	I65
0.93	0.752685	0.388192	2.47	0.4693	22.68	2.1242	2.47	I65
0.84	1.00446	0.285953	2.47	0.4693	22.76	2.1299	2.47	I65
0.58	1.07839	0.25948	4.75	0.7125	22.89	2.22525	6.75	C33
0.86	1.33413	0.180253	5.335	0.90695	22.81	2.7795	8.335	C33
0.55	0.458146	0.530902	4.815	0.7704	22.84	2.504	7.315	C33
0.58	0.704014	0.410088	4.75	0.7125	22.89	2.32275	6.75	C33
0.86	0.700171	0.411846	5.335	0.90695	22.81	2.9155	8.335	C33
0.86	1.27082	0.198106	5.335	0.90695	22.81	2.7795	8.335	C33
0.35	-0.03723	0.821738	1.5	0.15	21.18	0.879	1.5	B11
0.27	0.571838	0.472953	5.165	0.67145	22.24	1.97925	6.665	B11
0.32	0.634983	0.442306	5.7	0.798	22.27	2.254	7.7	B11
0.38	0.723717	0.401142	5.335	0.90695	22.08	2.68345	8.335	B11
0.57	0.837826	0.351537	4.69	0.6566	22.15	1.9411	6.19	B11
0.28	0.584275	0.466829	2.875	0.5175	21.95	2.0034	4.375	I64
1.15	0.701726	0.411135	3.915	0.7047	22.25	2.3634	6.415	I64
0.95	0.715791	0.404728	4	0.8	22.58	2.726	7	I64
0.79	0.852393	0.345478	3.6	0.792	22.94	2.8886	6.6	I64
0.87	1.03051	0.276438	3.4	0.612	22.85	2.1834	5.4	I64
1.42	1.16801	0.229548	4.785	1.2441	23.04	4.3238	10.285	I64
0.32	1.05281	0.268455	5.7	0.798	22.27	2.254	7.7	B11
0.38	1.25428	0.202956	5.335	0.90695	22.08	2.68345	8.335	B11
0.57	1.44263	0.152278	4.69	0.6566	22.15	1.9411	6.19	B11
0.76	0.6376	0.44106	1.5	0.15	22.18	0.9955	1.5	C13
0.8	0.997163	0.288652	1.5	0.15	22.27	0.965	1.5	C13

0.85	1.04672	0.27062	1.5	0.15	22.36	0.959	1.5	C13
0.91	1.30508	0.188302	1.5	0.15	22.41	1.0085	1.5	C13
0.49	0.786257	0.373487	2.75	0.4125	22.88	1.764	3.75	I64
0.27	0.89893	0.326537	2.75	0.4125	23.28	1.67925	3.75	I64
0.32	0.974081	0.297297	3	0.9	23.09	3.753	6	I64
0.25	1.17399	0.227637	3.6	0.792	23.02	2.9722	6.6	I64
0.54	1.28148	0.195017	1.75	0.2625	21.82	1.63875	2.25	C34
0.28	0.584275	0.466829	2.875	0.5175	21.95	2.0034	4.375	I64
1.15	0.701726	0.411135	3.915	0.7047	22.25	2.3634	6.415	I64
0.95	0.715791	0.404728	4	0.8	22.58	2.726	7	I64
1.15	0.688698	0.41712	3.915	0.7047	22.25	2.5344	6.415	I64
0.95	0.754605	0.387342	4	0.8	22.58	2.916	7	I64
0.79	0.854617	0.344558	3.6	0.792	22.94	3.0954	6.6	I64
0.87	1.05005	0.269438	3.4	0.612	22.85	2.3445	5.4	I64
1.42	1.14867	0.23581	4.785	1.2441	23.04	4.5565	10.285	I64
0.54	1.28148	0.195017	1.75	0.2625	21.82	1.63875	2.25	C34
0.32	0.986949	0.292458	3	0.3	23.98	1.121	3	E48
0.52	1.09085	0.255178	2.5	0.25	23.84	1.0515	2.5	E48
0.32	1.1603	0.232032	3	0.3	23.98	1.121	3	E48
0.37	1.24531	0.205622	2.5	0.25	24.18	1.102	2.5	E48
0.52	0.635458	0.44208	2.335	0.39695	21.84	1.97965	3.335	C22
0.43	-0.00883	0.803511	1	0.1	21.29	1.0145	1	C30
0.46	0.213521	0.667108	1	0.1	21.38	0.9705	1	C30
0.4	0.11019	0.729084	1	0.1	22.6	0.9375	1	N77
0.38	0.233831	0.655226	1	0.1	23.14	0.9725	1	N77
0.34	0.430375	0.545587	1	0.1	23.26	0.971	1	N77
0.35	0.669913	0.425835	1	0.1	23.1	1.0615	1	N77
0.32	0.67873	0.421732	1	0.1	23.02	1.0595	1	N77
0.28	0.626377	0.446417	2.875	0.48875	21.24	1.90995	3.375	C39
0.38	0.840219	0.350537	2.87	0.4879	20.99	1.9142	3.37	C39
0.63	0.764087	0.383161	1.44	0.2736	22	1.87815	1.44	C36
0.77	0.292616	0.621408	6.32	1.0112	24.93	2.8616	9.82	C36
0.64	0.951441	0.30593	3.665	0.47645	25.91	1.82325	4.665	C36
0.66	1.12155	0.244771	3.665	0.47645	25.99	1.8213	4.665	C36
0.71	0.098737	0.736107	1.355	0.23035	21.98	1.53935	1.355	C35
0.69	0.345035	0.591982	1.905	0.3429	22.09	1.809	1.905	C35
0.69	0.498947	0.509702	2.975	0.56525	22.25	2.09665	2.975	C35
0.72	0.61085	0.453887	2.47	0.4693	22.44	2.1261	2.47	C35
0.7	0.759749	0.385071	2.975	0.595	22.64	2.352	2.975	C35
0.77	0.116741	0.725081	6.32	1.0112	24.93	3.0216	9.82	C36
0.65	0.202399	0.673657	3.75	0.5625	24.97	2.091	5.25	C36
0.68	0.333549	0.59837	3.75	0.5625	25.08	2.0265	5.25	C36
0.65	0.422722	0.54967	3.75	0.5625	25.27	2.0265	5.25	C36
0.74	0.645015	0.43754	3.75	0.5625	25.47	2.14275	5.25	C36
0.77	0.846197	0.348048	2.335	0.39695	25.7	2.16155	3.335	C36
0.64	0.977197	0.296121	3.665	0.47645	25.91	1.79725	4.665	C36
0.66	1.08507	0.257167	3.665	0.47645	25.99	1.833	4.665	C36
0.63	1.32815	0.18189	2.335	0.39695	26	2.21255	3.335	C36
0.77	0.223124	0.661478	6.32	1.0112	24.93	2.8616	9.82	C36
0.77	-0.32035	1.01238	6.32	1.0112	24.93	3.1016	9.82	C36
0.65	-0.39785	1.06722	3.75	0.5625	24.97	2.091	5.25	C36

0.68	-0.21324	0.938411	3.75	0.5625	25.08	2.091	5.25	C36
0.65	0.070479	0.753564	3.75	0.5625	25.27	2.121	5.25	C36
0.74	0.109406	0.729564	3.75	0.5625	25.47	2.14275	5.25	C36
0.69	0.841859	0.349853	1.905	0.3429	22.09	1.809	1.905	C35
0.69	0.941207	0.309883	2.975	0.56525	22.25	2.04155	2.975	C35
0.69	0.902331	0.325178	2.975	0.56525	22.25	2.23155	2.975	C35
0.72	0.951893	0.305757	2.47	0.4693	22.44	2.1261	2.47	C35
0.7	1.20392	0.218217	2.975	0.595	22.64	2.452	2.975	C35
0.7	1.32451	0.182893	2.975	0.595	22.64	2.352	2.975	C35
0.63	-0.04312	0.825535	2.55	0.5355	22.56	2.5158	2.55	C27
0.57	0.115711	0.72571	2.41	0.4338	22.79	2.1564	2.41	C27
0.31	1.04739	0.270384	2.575	0.54075	23.37	2.78775	3.625	C38
0.4	1.23553	0.208553	2.56	0.5376	23.18	2.66175	3.01	C38
0.4	1.06692	0.26348	2.575	0.54075	23.37	2.78775	3.625	C38
0.45	0.674998	0.423466	1.75	0.2625	21.79	1.63875	2.25	C18
0.4	0.729144	0.398698	2.25	0.5625	21.75	2.98125	3.75	C18
0.38	0.660914	0.430045	3	1.2	22.21	5.37	6	C18
0.41	0.810177	0.363209	3	1.2	22.66	5.37	6	C18
0.37	0.931323	0.313729	3.25	1.4625	22.71	6.26625	6.75	C18
0.47	1.02451	0.278612	4	2.4	22.39	9.255	9	C18
0.4	0.729144	0.398698	2.25	0.5625	21.75	3.0975	3.75	C18
0.38	0.660914	0.430045	3	1.2	22.21	5.556	6	C18
0.41	0.810177	0.363209	3	1.2	22.66	5.556	6	C18
0.37	0.931323	0.313729	3.25	1.4625	22.71	6.4755	6.75	C18
0.47	1.02451	0.278612	4	2.4	22.39	9.534	9	C18
0.66	0.398143	0.562887	1.85	0.3145	21.71	1.61755	1.85	C38
0.78	0.737224	0.395074	2.805	0.4488	22.09	1.9288	4.305	C38
0.82	0.809462	0.363514	2.865	0.48705	22.2	2.13435	4.865	C38
0.92	0.903144	0.324853	6	1.2	22.3	3.804	11.5	C38
1	1.08591	0.256877	3.905	0.7029	22.51	2.6127	6.905	C38
0.86	1.21618	0.214434	6.03	1.2663	22.55	3.57315	9.53	C38
0.78	0.829077	0.355206	2.805	0.4488	22.09	1.8488	4.305	C38
0.82	0.982614	0.294083	2.865	0.48705	22.2	2.04935	4.865	C38
0.92	1.1466	0.236484	6	1.2	22.3	4.101	11.5	C38
1	1.36007	0.173267	3.905	0.7029	22.51	2.8926	6.905	C38
0.86	1.48445	0.142358	6.03	1.2663	22.55	3.8997	9.53	C38
0.92	0.796896	0.368895	6	1.2	22.3	3.711	11.5	C38
1	0.927007	0.315418	3.905	0.7029	22.51	2.5299	6.905	C38
0.86	1.10492	0.250372	6.03	1.2663	22.55	3.89865	9.53	C38
0.86	0.416988	0.552739	6.03	1.2663	22.55	3.95115	9.53	C38
0.56	0.76901	0.381	3.5	1.05	21.85	4.1625	7.5	C14
0.64	0.862692	0.341231	3.625	1.19625	21.92	4.88235	8.125	C14
0.72	0.969495	0.299034	4.125	1.77375	22.25	7.1982	10.625	C14
0.68	1.07445	0.260851	4.5	2.25	22.27	9.12	12.5	C14
0.61	0.369186	0.57866	1	0.1	22.38	0.9565	1	C34
0.53	0.458359	0.530791	1	0.1	22.69	0.9565	1	C34
0.45	0.649	0.435655	1.75	0.2625	22.94	1.7205	2.25	C34
0.34	0.862842	0.341169	1.75	0.2625	23.09	1.73475	2.25	C34
0.33	0.968521	0.299404	1.75	0.2625	23.38	1.7295	2.25	C34
0.25	1.07639	0.260172	1.75	0.2625	23.41	1.71825	2.25	C34
0.26	1.3448	0.177357	1.75	0.2625	23.28	1.7205	2.25	C34

0.45	0.919682	0.318296	1.225	0.18375	22.89	1.16325	1.225	C39
0.4	0.943923	0.308831	1.97	0.3743	22.9	2.0349	1.97	C39
0.4	1.20228	0.218727	1.975	0.37525	22.66	2.03965	1.975	C39
1.24	0.792388	0.370836	1.475	0.295	22.3	2.052	1.475	C26
1.23	1.07372	0.261103	1.46	0.2774	22.57	2.00925	1.46	C26
1.05	1.14318	0.237607	1.465	0.27835	22.67	1.83445	1.465	C26
0.96	1.287	0.193433	1.46	0.2774	22.62	1.881	1.46	I65
0.28	0.820761	0.358715	2.165	0.28145	21.93	1.30585	2.665	C34
0.27	1.09577	0.253492	2.165	0.28145	21.97	1.3468	2.665	C34
0.35	1.16522	0.230447	2.165	0.28145	22.09	1.33575	2.665	C34
0.38	1.37081	0.17043	2.625	0.34125	22.11	1.417	3.125	C34
0.84	0.285375	0.625527	2.81	0.4496	21.32	1.6152	2.81	I65
0.8	0.368218	0.579192	3.33	0.5661	21.47	1.88615	3.83	I65
0.77	0.590511	0.463774	4.37	0.7429	21.59	2.2525	5.42	I65
0.76	0.798023	0.36841	4.4	0.792	21.7	2.628	6.5	I65
0.81	0.910032	0.322113	4.365	0.74205	21.78	2.49985	6.815	I65
0.92	1.01158	0.283333	4.39	0.7902	21.93	2.3814	5.44	I65
0.84	0.578391	0.469721	2.81	0.4496	21.32	1.6952	2.81	I65
0.8	0.732292	0.397284	3.33	0.5661	21.47	2.006	3.83	I65
0.77	0.693036	0.415122	4.37	0.7429	21.59	2.2695	5.42	I65
0.76	0.765274	0.38264	4.4	0.792	21.7	2.592	6.5	I65
0.81	0.884277	0.332432	4.365	0.74205	21.78	2.4667	6.815	I65
0.92	1.04805	0.270146	4.39	0.7902	21.93	2.3544	5.44	I65
0.18	0.507792	0.505165	2	0.2	21.87	0.7055	2	P82
0.82	1.29461	0.191262	1.5	0.15	22.1	0.9375	1.5	C14
0.75	1.40067	0.162712	2	0.4	22.14	2.079	3	C14
0.34	0.772618	0.379422	1.86	0.3162	22.17	1.632	1.86	C27
0.33	0.905611	0.32387	2.025	0.42525	22.39	2.3646	2.025	C27
0.46	0.728772	0.398865	2.75	0.4125	22.22	1.719	3.75	C24
0.8	-0.04868	0.829128	2	0.2	23.01	0.979	2	G54
0.61	0.17367	0.69071	2	0.2	23.02	0.9845	2	G54
0.82	0.243409	0.649658	2	0.2	22.77	1.0045	2	B11
0.82	1.09666	0.253185	2	0.2	22.77	1.0045	2	B11
0.71	-0.10041	0.862889	3.2	0.448	21.63	1.2292	4.2	C34
0.5	0.70485	0.409707	5.7	0.798	21.59	1.7227	7.7	C34
0.35	0.812629	0.362165	5.6	0.672	21.47	1.4028	6.6	C34
0.5	1.23542	0.208587	5.7	0.798	21.59	1.6527	7.7	C34
0.35	1.41744	0.158485	5.6	0.672	21.47	1.3008	6.6	C34
0.5	1.08273	0.257977	5.7	0.798	21.59	1.6534	7.7	C34
0.35	1.36379	0.17228	5.6	0.672	21.47	1.3188	6.6	C34
0.22	0.923774	0.316686	2.875	0.5175	22.25	2.232	4.375	C34
1.19	1.33752	0.17933	1.345	0.22865	22.66	1.52575	1.345	C38
0.5	0.194451	0.678355	2	0.2	24.12	1.0305	2	A03
0.49	0.30721	0.613145	2.875	0.5175	24.69	2.2149	4.375	A03
0.49	0.822467	0.357993	2.875	0.5175	24.69	2.2014	4.375	A03
0.59	0.87678	0.335472	1.75	0.2625	23.11	1.6935	2.25	N77
0.65	1.04224	0.272222	2.165	0.28145	23.52	1.5327	2.665	N77
0.44	1.21772	0.213962	1.75	0.2625	23.7	1.6935	2.25	N77
0.72	0.919789	0.318254	1.9	0.342	20.84	1.8009	1.9	C36
0.83	1.0899	0.255504	1.9	0.342	20.91	1.8	1.9	C36
0.72	0.813542	0.361777	1.9	0.342	20.84	1.8009	1.9	C36

0.83	0.930992	0.313858	1.9	0.342	20.91	1.8009	1.9	C36
1.16	0.816416	0.360556	2.415	0.4347	21.53	1.9161	2.415	C38
0.9	0.963622	0.301267	3.475	0.695	21.89	2.329	3.475	C38
0.78	1.1466	0.236484	3.485	0.697	21.98	2.666	4.485	C38
0.76	1.34741	0.176653	3.485	0.697	22.16	2.673	4.485	C38
0.67	1.49078	0.140897	3.485	0.697	22.21	2.873	5.485	C38
0.44	0.636611	0.441531	4.625	0.60125	20.47	1.5392	5.625	C32
0.41	0.812085	0.362396	4.75	0.7125	20.7	1.80525	6.75	C32
0.31	1.33681	0.179522	5.445	1.03455	22.13	2.94405	9.445	C32
0.6	0.677643	0.422236	8.285	7.9536	24	40.0512	38.285	R86
0.49	0.920576	0.317944	2.04	0.4284	23.66	2.39715	2.04	C39
0.7	1.02212	0.279481	2.595	0.5709	24.19	2.6147	2.595	C39
0.56	1.29053	0.192426	2.59	0.5698	24.27	2.7269	2.59	C39
0.8	0.171548	0.691978	1.735	0.26025	20.43	1.2525	1.735	C40
0.51	0.440682	0.540113	1.715	0.2401	21.36	1.1795	1.715	C40
0.42	0.456589	0.53172	4.865	0.82705	21.9	2.52705	6.865	C40
0.42	0.666103	0.427614	4.875	0.82875	22.01	2.3953	6.075	C40
0.8	0.637045	0.441324	1.735	0.26025	20.43	1.2525	1.735	C40
0.51	0.857218	0.343484	1.715	0.2401	21.36	1.2145	1.715	C40
0.42	0.943905	0.308838	4.865	0.82705	21.9	2.49305	6.865	C40
0.42	1.05178	0.268822	4.875	0.82875	22.01	2.125	6.075	C40
0.38	1.32018	0.184088	4.345	0.73865	22.05	2.55425	8.195	C40
0.42	0.623967	0.447572	4.865	0.82705	21.9	2.26865	6.865	C40
0.42	0.721398	0.40219	4.875	0.82875	22.01	2.0298	6.075	C40
0.38	0.929188	0.314564	4.345	0.73865	22.05	2.71405	8.195	C40
0.6	0.630186	0.444595	1.75	0.2625	22.27	1.548	2.25	C33
1.88	1.25089	0.203962	2.625	0.34125	25.1	1.5119	3.125	E48
0.59	0.245243	0.648594	1.98	0.396	22.37	2.16	1.98	C27
0.58	0.445236	0.537704	1.985	0.397	22.49	2.167	1.985	C27
0.98	1.08885	0.255863	1.5	0.15	22.19	1.0005	1.5	E50
1.07	1.15831	0.232677	1.5	0.15	22.32	1.0065	1.5	E50
0.82	1.3639	0.172252	1.5	0.15	22.29	1.024	1.5	E50
0.96	1.49512	0.139903	1.955	0.37145	20.9	2.00355	1.955	C39
0.67	1.3618	0.172808	3.1	0.372	22.16	1.2156	3.6	C35
1.07	0.841744	0.349901	3	0.3	21.84	1.0185	3	C33
0.97	1.11675	0.24638	3.5	0.35	21.97	1.103	3.5	C33
1.02	1.1862	0.223762	3.5	0.35	22.12	1.124	3.5	C33
1.16	1.3918	0.164979	3.5	0.35	22.32	1.147	3.5	C33
0.54	0.769305	0.380871	3.48	0.696	21.87	2.563	3.98	C39
1.07	0.896487	0.327515	3	0.3	21.84	1.0185	3	C33
0.97	1.00217	0.286799	3.5	0.35	21.97	1.053	3.5	C33
1.02	1.11004	0.24864	3.5	0.35	22.12	1.024	3.5	C33
1.16	1.37845	0.168433	3.5	0.35	22.32	1.0685	3.5	C33
1.07	1.03689	0.274141	3	0.3	21.84	1.0185	3	C33
0.97	1.21987	0.213304	3.5	0.35	21.97	1.073	3.5	C33
1.02	1.42068	0.157676	3.5	0.35	22.12	1.124	3.5	C33
1.16	1.56405	0.124761	3.5	0.35	22.32	1.124	3.5	C33
0.97	0.96329	0.301394	3.5	0.35	21.97	1.053	3.5	C33
1.02	1.01285	0.282865	3.5	0.35	22.12	1.024	3.5	C33
1.16	1.27121	0.197991	3.5	0.35	22.32	1.147	3.5	C33
0.54	1.30319	0.188836	3.48	0.696	21.87	2.563	3.98	C39

0.85	1.3548	0.174671	3.3	0.528	21.74	1.5424	4.8	E50
0.81	1.48551	0.142113	3.4	0.612	21.95	1.8252	5.4	E50
0.69	1.07958	0.259066	1.955	0.37145	21.77	2.57355	4.955	C38
0.41	0.723434	0.40127	1	0.1	20.86	0.4705	1	B11
0.54	0.913562	0.320713	2	0.2	21.35	0.5705	2	B11
0.64	1.06475	0.264241	2	0.2	21.65	0.4595	2	B11
0.84	1.20615	0.217526	2.75	0.4125	22.92	1.686	3.75	C22
0.84	0.988446	0.291898	2.75	0.4125	22.92	1.686	3.75	C22
0.85	0.846549	0.347901	1.8	0.288	20.86	1.44	1.8	C39
0.86	0.945897	0.308068	1.845	0.31365	21	1.6065	1.845	C39
0.63	1.04744	0.270365	2.395	0.4311	20.88	1.8819	2.395	C39
0.56	1.32218	0.183536	1.845	0.31365	20.79	1.60735	1.845	C39
0.85	-0.15109	0.896495	1.8	0.288	20.86	1.44	1.8	C39
0.86	0.094348	0.738807	1.845	0.31365	21	1.6065	1.845	C39
0.63	0.269093	0.634838	2.395	0.4311	20.88	1.8819	2.395	C39
0.56	0.451786	0.534247	1.845	0.31365	20.79	1.60735	1.845	C39
0.99	1.31151	0.186501	3.435	0.65265	22.1	2.2553	3.435	C38
0.99	1.20427	0.218108	3.435	0.65265	22.1	2.2553	3.435	C38
0.83	0.930579	0.31402	2.225	0.33375	21.41	1.31325	2.225	C39
0.98	1.08971	0.25557	2.225	0.3115	21.65	1.2257	2.225	C39
1.05	1.27785	0.196065	1.68	0.2352	21.74	1.1557	1.68	C39
0.83	1.17626	0.226912	2.225	0.33375	21.41	1.31325	2.225	C39
0.98	1.3834	0.167145	2.225	0.3115	21.65	1.2285	2.225	C39
1.05	1.52677	0.132795	1.68	0.2352	21.74	1.1585	1.68	C39
0.83	0.932803	0.313151	2.225	0.33375	21.41	1.38825	2.225	C39
0.98	1.10924	0.24891	2.225	0.3115	21.65	1.2915	2.225	C39
1.43	1.1604	0.232001	2.25	0.5625	23.21	2.6925	3.75	L72
1.1	0.600755	0.45878	2.25	0.5625	21.25	2.7925	3.75	C13
0.5	0.951976	0.305725	1.755	0.26325	21.23	1.2855	1.755	C35
0.64	1.15521	0.233679	1.205	0.1687	21.53	1.1963	1.205	C35
1.12	0.92612	0.315766	1.855	0.31535	22.55	1.62605	1.855	C36
0.88	1.24549	0.205568	2.365	0.40205	22.57	1.90485	3.415	C36
0.42	0.77077	0.38023	2.86	0.4862	22.42	1.90655	3.46	C39
0.56	0.706261	0.409063	1.5	0.15	23.04	0.702	1.5	E48
0.56	0.801379	0.36697	1.5	0.15	23.12	0.626	1.5	E48
0.56	0.658051	0.43139	1.5	0.15	23.04	0.702	1.5	E48
0.56	0.829412	0.355066	1.5	0.15	23.12	0.626	1.5	E48
0.67	0.834991	0.352724	6.135	0.79755	23.19	1.99615	7.635	C38
0.42	1.01964	0.280382	7.115	0.8538	23.23	1.9836	8.615	C38
0.46	1.22745	0.210997	7.19	1.0066	23.32	2.5361	9.69	C38
0.47	1.3968	0.163696	7.19	1.0066	23.43	2.5459	9.69	C38
0.52	0.645463	0.437328	4.215	0.5901	22.43	1.9537	5.715	C38
0.48	0.741147	0.393321	5.335	0.90695	22.5	2.79735	8.335	C38
0.45	0.752438	0.388301	4.43	0.8417	22.67	2.93645	7.43	C38
0.29	0.898145	0.326851	4.5	0.9	22.66	3.191	8	C38
0.49	0.414364	0.554147	2.165	0.28145	22.15	1.4131	2.665	C38
0.49	0.588006	0.465	4.215	0.5901	22.15	1.9418	5.715	C38
0.48	0.819972	0.359049	5.335	0.90695	22.5	2.89765	8.335	C38
0.45	0.964404	0.300969	4.43	0.8417	22.67	3.04855	7.43	C38
0.29	1.1466	0.236484	4.5	0.9	22.66	3.309	8	C38
0.64	1.13712	0.2396	3.665	0.47645	21.98	1.51645	4.665	M74

0.71	1.35692	0.174106	3	0.3	22.12	1.088	3	M74
0.39	1.52221	0.133804	2	0.2	21.39	1.003	2	G59
0.93	0.849599	0.346635	6	2.4	23.2	8.74	18	Q83
0.57	0.784359	0.374309	6.79	1.0864	23.05	2.8096	10.29	I65
0.57	0.89548	0.327919	6.835	1.16195	23.2	3.0702	10.835	I65
0.54	1.08606	0.256827	6.875	1.2375	23.06	3.3507	11.375	I65
0.45	1.01412	0.2824	3	0.3	22.73	0.9695	3	C27
0.56	1.10933	0.248878	3.1	0.372	22.74	1.2612	3.6	C27
0.62	1.3904	0.165338	4	0.4	22.85	1.11	4	C27
0.28	0.329654	0.600544	8.465	1.60835	23.01	4.44315	15.465	R86
0.28	0.545617	0.486005	8.5	1.7	23.61	4.891	16	R86
0.18	0.721477	0.402154	8.8	2.288	23.71	7.579	20.8	R86
0.2	0.782843	0.374967	9.655	3.18615	23.73	11.9163	28.155	R86
0.2	0.890717	0.329833	10.145	3.34785	23.63	12.2958	29.645	R86
0.68	1.16133	0.2317	3.7	0.888	22.11	3.1776	7.2	I64
0.33	0.132422	0.715538	1.5	0.15	20.28	0.881	1.5	I65
0.42	0.288755	0.623603	1.5	0.15	20.4	0.881	1.5	I65
0.36	0.404598	0.5594	1.5	0.15	20.44	0.851	1.5	I65
0.4	0.616373	0.451222	1.5	0.15	20.52	0.8795	1.5	I65
0.43	0.776604	0.377681	1.5	0.15	20.58	0.881	1.5	I65
0.49	0.737348	0.395018	1.5	0.15	20.76	0.881	1.5	I65
0.51	0.815917	0.360768	1.5	0.15	21.51	0.881	1.5	I65
0.51	0.928589	0.314798	1.5	0.15	21.69	0.852	1.5	I65
0.55	1.0987	0.252491	1.5	0.15	21.76	0.851	1.5	I65
0.59	1.24796	0.204832	1.5	0.15	21.84	0.851	1.5	I65
0.53	0.627494	0.445882						C26
0.73	1.52735	0.132667	1.425	0.2565	21.71	1.7523	1.425	C26
0.44	1.24995	0.20424	2.5	0.25	22.47	1.128	2.5	I64
0.33	0.622258	0.448392	3.1	0.372	21.15	1.2888	3.6	I64
0.24	0.739708	0.393963	3.1	0.372	20.95	1.2888	3.6	I64
0.27	0.766434	0.38213	3.1	0.372	20.82	1.2888	3.6	I64
0.28	0.915697	0.319869	3.1	0.372	20.85	1.2888	3.6	I64
0.91	0.910737	0.321833	2.75	0.4125	23.54	1.87425	3.75	L72
1.37	1.01861	0.280759	2.75	0.4125	23.55	1.86975	3.75	L72
0.83	0.922568	0.31716	3.53	0.7413	23.11	2.78775	4.03	C38
0.66	1.03044	0.276464	3.55	0.7455	23.26	2.835	4.55	C38
0.68	1.30518	0.188275	3	0.6	23.3	2.699	4	C38
0.6	1.10982	0.248713	1.925	0.3465	23.96	1.8441	1.925	C26
1.41	0.782148	0.375269	1.525	0.32025	22.7	2.2617	1.525	C39
1.47	0.894821	0.328184	1.535	0.32235	22.74	2.28165	1.535	C39
1.14	1.07126	0.261963	1.525	0.305	22.96	2.146	1.525	C39
0.65	1.21419	0.215046	1.53	0.3213	22.92	2.26695	1.53	C39
0.34	0.754804	0.387254	1.86	0.3162	20.94	1.6337	1.86	C26
0.31	0.814381	0.36142	2.48	0.496	20.99	2.112	2.93	C26
0.86	0.722938	0.401494	2.015	0.403	22.35	2.229	2.015	C39
0.92	0.743333	0.392346	2.03	0.4263	22.83	2.34045	2.03	C39
0.92	0.898927	0.326538	2.015	0.403	23.03	2.229	2.015	C39
0.86	1.05172	0.268841	2.01	0.402	23.01	2.229	2.01	C39
0.88	1.23987	0.207249	2.01	0.402	22.99	2.229	2.01	C39
0.86	0.903144	0.324853	3.48	0.696	23.53	2.461	3.48	C38
0.55	1.18946	0.222734	3.49	0.698	23.66	2.383	3.49	C38

0.65	1.35882	0.173599	3.485	0.697	23.97	2.397	3.485	C38
0.52	0.64103	0.43943	1.745	0.26175	22.51	1.26825	1.745	C38
0.52	0.829551	0.355007	1.955	0.37145	22.8	2.0349	1.955	C38
0.56	0.935229	0.312206	1.97	0.3743	22.93	2.0064	1.97	C38
0.6	1.03677	0.274182	3.01	0.602	23.06	1.881	3.01	C38
0.58	1.29885	0.190061	3.525	0.74025	23.42	2.6691	4.025	C38
0.81	0.94324	0.309095	2.625	0.34125	22.09	1.47355	3.125	C39
0.87	0.624479	0.447327	2.365	0.40205	20.77	1.7272	2.365	C39
0.86	0.741929	0.392972	2.365	0.40205	21.39	1.7272	2.365	C39
0.8	0.905257	0.324011	2.925	0.55575	22.4	2.0254	2.925	C39
0.74	1.05805	0.2666	2.925	0.55575	22.7	2.0254	2.925	C39
0.66	1.23354	0.209153	3.535	0.74235	22.85	2.3436	3.535	C39
0.98	1.0996	0.252183	2.335	0.39695	21.93	1.4943	3.335	C13
0.83	1.34901	0.176222	3	0.6	22.23	2.489	5	C13
0.24	1.15118	0.23499	2.335	0.39695	23.12	2.19895	3.335	R87
0.37	1.27998	0.195451	1.425	0.2565	21.59	1.7523	1.425	I65
0.59	0.9696	0.298994	1.5	0.3	20.73	2.097	1.5	C35
0.48	0.831191	0.354318	4.715	1.1316	22.57	4.2504	9.715	B11
0.25	0.812276	0.362315	4.785	1.2441	22.43	4.7125	10.285	B11
0.21	0.995118	0.289411	4.57	0.9597	22.25	3.4398	8.57	B11
0.37	0.902085	0.325276	2.665	0.61295	21.45	2.72895	4.665	C29
0.43	1.07219	0.261636	2.665	0.61295	21.45	2.668	4.665	C29
0.46	1.22146	0.212821	2.665	0.61295	21.32	2.668	4.665	C29
0.62	0.591051	0.46351	1.19	0.1666	21.11	1.0374	1.19	I65
0.8	0.903268	0.324804	1.69	0.2366	21.87	1.0395	1.69	I65
0.82	1.07338	0.261224	1.695	0.2373	22.06	1.0143	1.695	I65
0.68	1.22264	0.21246	2.685	0.3759	22.75	1.1543	2.685	I65
0.29	0.322881	0.604333	1.84	0.3128	22.05	1.59885	1.84	C35
0.31	0.412053	0.555387	1.875	0.31875	22.15	1.59885	1.875	C35
0.38	0.621686	0.448667	1.935	0.36765	22.12	1.96935	1.935	C35
0.32	0.835528	0.352499	1.935	0.36765	22.16	1.9855	1.935	C35
0.4	0.941207	0.309883	1.93	0.3667	22.2	1.97885	1.93	C35
0.42	1.04908	0.269781	2.43	0.4617	22.27	2.0634	2.43	C35
0.46	1.32382	0.183084	2.42	0.4356	22.37	1.971	2.42	C35
0.38	-0.01001	0.804269	1.925	0.3465	21.44	1.8441	1.925	C39
0.44	0.253885	0.643594	3.455	0.65645	21.64	2.38735	3.955	C39
0.48	0.469988	0.524703	3.975	0.795	21.75	2.554	3.975	C39
0.54	0.54546	0.486083	3.965	0.75335	21.88	2.4263	3.965	C39
0.21	0.675918	0.423038	2	0.4	22.23	2.119	3	N77
0.31	0.928107	0.314987	2.165	0.28145	22.7	1.3871	2.665	N77
0.35	1.18647	0.223679	2.165	0.28145	22.82	1.48525	2.665	N77
0.39	0.416782	0.55285	1.76	0.264	21.07	1.599	1.76	C35
0.41	0.628557	0.445374	1.75	0.2625	21.17	1.629	1.75	C35
0.34	0.71788	0.403781	2.495	0.499	22.02	2.292	2.495	C35
0.31	0.796448	0.369087	3	0.6	22.01	2.492	3.5	C35
0.25	0.909121	0.322474	2.99	0.598	22.4	2.318	2.99	C35
0.23	1.07923	0.259188	2.995	0.599	22.5	2.332	2.995	C35
0.23	1.22216	0.212605	3.035	0.63735	22.38	2.4486	3.035	C35
0.31	0.343669	0.59274	3	0.6	22.01	2.495	3.5	C35
0.25	0.535993	0.490843	2.99	0.598	22.4	2.378	2.99	C35
0.23	0.650978	0.434721	2.995	0.599	22.5	2.393	2.995	C35

0.23	0.82824	0.355558	3.035	0.63735	22.38	2.59035	3.035	C35
0.47	0.85452	0.344598	2.825	0.452	21.11	1.6368	2.825	C35
0.58	0.953868	0.304998	3.825	0.65025	21.08	1.8972	3.825	C35
0.68	1.06807	0.263077	2.815	0.4504	21.09	1.624	2.815	C35
0.77	1.34281	0.177895	3.255	0.48825	21.13	1.6095	3.255	C35
1.14	0.835881	0.352351	1.895	0.3411	22.84	1.7883	1.895	C38
1.31	0.94156	0.309746	1.895	0.3411	23.29	1.782	1.895	C38
1.28	1.04943	0.269655	1.89	0.3402	23.65	1.7541	1.89	C38
1.19	1.30518	0.188275	1.975	0.395	23.88	1.987	1.975	C38
0.29	1.13193	0.241317	2.84	0.4828	22.35	1.768	2.84	C38
0.3	0.718357	0.403565	1.17	0.1521	20.73	0.9373	1.17	I65
0.29	1.05576	0.267409	2.84	0.4828	22.35	1.768	2.84	C38
0.49	0.809462	0.363514	1.37	0.2329	22.31	1.56315	1.37	C38
0.51	1.08591	0.256877	2.92	0.5256	22.79	1.8396	2.92	C38
0.34	0.982388	0.294168	1.5	0.15	22.07	0.8425	1.5	C30
0.48	0.787646	0.372885	1.22	0.1708	20.35	1.0808	1.22	C39
0.58	0.924248	0.3165	1.28	0.2048	20.78	1.2352	1.28	C39
0.77	0.03282	0.777109	4.815	0.7704	21.24	2.2432	7.315	C29
0.63	0.108614	0.730049	7.165	1.64795	22.13	4.87025	15.165	C29
0.74	0.29014	0.622815	7.25	1.8125	22.16	5.60625	16.25	C29
0.78	0.450596	0.534874	7.415	2.0762	22.25	6.8656	18.415	C29
0.71	0.625846	0.446672	7	2.1	22.25	7.38	18	C29
0.8	1.22685	0.211178	2.465	0.46835	22.74	2.11945	2.465	C39
0.63	0.842745	0.349484	7.165	1.64795	22.13	4.87025	15.165	C29
0.74	0.966805	0.300055	7.25	1.8125	22.16	5.60625	16.25	C29
0.78	0.912423	0.321164	7.415	2.0762	22.25	6.8642	18.415	C29
0.71	1.05172	0.268841	7	2.1	22.25	7.08	18	C29
1.3	1.41459	0.159197	2.835	0.76545	21.67	3.43575	5.335	G59
0.77	1.05725	0.266886	1.5	0.15	23.09	1.0965	1.5	Q83
0.62	0.835881	0.352351	2.885	0.5193	22.13	2.1123	2.885	C38
0.46	0.935229	0.312206	2.955	0.56145	22.49	2.19925	2.955	C38
0.47	1.03677	0.274182	3.49	0.698	22.35	2.552	4.24	C38
0.51	1.30518	0.188275	3.015	0.603	22.39	2.547	4.015	C38
0.62	0.744369	0.391885	2.885	0.5193	22.13	1.9566	2.885	C38
0.46	0.840867	0.350267	2.955	0.56145	22.49	2.19925	2.955	C38
0.47	0.892422	0.329148	3.49	0.698	22.35	2.632	4.24	C38
0.51	1.14801	0.236026	3.015	0.603	22.39	2.631	4.015	C38
0.33	0.774402	0.378642	4.875	0.8775	22.82	2.7108	7.875	C34
0.43	0.73578	0.39572	2.625	0.34125	20.74	1.1609	3.125	C34
0.23	0.084862	0.744656	4	0.4	22.21	1.2295	4	N77
0.22	0.265938	0.636649	4	0.4	22.4	1.2295	4	N77
0.17	0.442301	0.539256	4.5	0.45	22.5	1.2795	4.5	N77
0.86	0.842212	0.349706	1.37	0.2329	21.43	1.65155	1.37	C38
0.73	0.935229	0.312206	1.435	0.27265	21.47	1.83255	1.435	C38
0.73	1.0431	0.271913	1.445	0.27455	21.55	1.81925	1.445	C38
0.61	1.31151	0.186501	1.425	0.27075	21.65	1.84585	1.425	C38
0.53	0.118366	0.724089	1.465	0.27835	22.42	1.9285	1.465	C27
0.5	0.295168	0.61996	1.385	0.2493	22.47	1.827	1.385	C27
0.59	1.06005	0.265897	8.43	1.6017	23.64	3.33735	9.18	C38
0.63	1.24819	0.204763	9.45	1.7955	23.85	3.54635	10.3	C38
0.78	0.724563	0.400761	3.88	0.6984	22.64	2.1249	3.88	C38

0.65	0.848871	0.346937	4.885	0.8793	23.18	2.3148	4.885	C38
0.63	0.955619	0.304326	5.4	0.972	23.51	2.43	5.4	C38
0.65	0.774472	0.378611	4.885	0.8793	23.18	2.3148	4.885	C38
0.63	0.692395	0.415416	5.4	0.972	23.51	2.43	5.4	C38
0.59	0.82708	0.356047	8.43	1.6017	23.64	3.33735	9.18	C38
0.63	1.03273	0.275639	9.45	1.7955	23.85	3.57675	10.3	C38
0.83	0.584651	0.466644	1.32	0.2112	20.78	1.3952	1.32	C38
0.58	0.362594	0.582281	7.69	1.8456		5.49	16.69	M74
0.65	0.575975	0.470911	8.535	1.79235		4.78485	16.535	M74
0.6	0.729769	0.398417	7.855	1.33535		3.349	12.855	M74
0.46	0.86966	0.338376	7.82	1.2512		3.072	12.32	M74
1.01	1.01158	0.283333	1.41	0.2538	21.62	1.7271	1.41	I65
0.55	0.268013	0.635457	4.2	0.588	22.39	1.7675	6.3	C39
0.65	0.34836	0.590139	4.415	0.7947	22.83	2.2725	6.515	C39
0.62	0.51294	0.502534	4.92	0.8856	23.26	2.5605	8.12	C39
0.56	0.677081	0.422497	4.95	0.9405	23.4	2.70275	8.15	C39
0.6	0.891192	0.329642	4.955	0.94145	23.46	3.02765	7.355	C39
0.55	0.747892	0.390318	4.2	0.588	22.39	1.7675	6.3	C39
0.65	0.846878	0.347764	4.415	0.7947	22.83	2.6514	6.515	C39
0.62	0.9543	0.304832	4.92	0.8856	23.26	2.9529	8.12	C39
0.56	1.17356	0.227773	4.95	0.9405	23.4	3.1768	8.15	C39
0.6	1.32283	0.183357	4.955	0.94145	23.46	3.02765	7.355	C39
0.5	0.615356	0.451713	1.475	0.295	21.01	2.051	1.475	C35
0.56	0.829198	0.355155	2	0.4	21.11	2.203	2	C35
0.65	0.934876	0.312343	2	0.4	21.23	2.199	2	C35
0.56	1.31116	0.186599	1.995	0.399	21.91	2.503	3.495	C35
0.18	-0.14992	0.89571	1	0.1	21.07	0.8645	1	J67
0.08	-0.37793	1.05303	1.75	0.2625	21.93	1.515	2.25	J67
0.17	-0.24163	0.957807	2.75	0.4125	22.44	1.74	3.75	J67
0.26	0.376561	0.574623	2.25	0.5625	21.38	2.62125	3.75	I64
0.22	0.552623	0.482499	2.665	0.61295	22.34	2.64155	4.665	I64
0.17	0.644752	0.437665	2.835	0.76545	22.79	3.23595	5.335	I64
0.2	0.665148	0.428061	3.375	0.945	23.07	3.7758	6.875	I64
0.17	0.782759	0.375004	3.625	1.19625	23.01	4.78005	8.125	I64
0.25	0.954548	0.304737	3.5	1.05	22.49	4.1955	7.5	I64
0.27	1.14269	0.237766	3.5	1.05	22.51	4.1955	7.5	I64
0.38	0.959764	0.302739	1.105	0.1326	22.52	1.2234	1.105	C35
0.41	1.08556	0.256998	1.47	0.2793	22.57	1.94655	1.47	C35
0.44	1.22849	0.21068	1.5	0.3	22.79	2.049	1.5	C35
0.2	0.665148	0.428061	3.375	0.945	23.07	4.2714	6.875	I64
0.17	0.810502	0.363071	3.625	1.19625	23.01	5.36415	8.125	I64
0.25	0.98784	0.292125	3.5	1.05	22.49	4.7265	7.5	I64
0.27	1.19818	0.220004	3.5	1.05	22.51	4.7265	7.5	I64
0.64	0.735599	0.395801	1.89	0.3402	20.97	1.7784	1.89	C39
0.92	0.768655	0.381156	1.87	0.3179	21.61	1.6796	1.87	C39
0.89	1.08338	0.257752	1.765	0.26475	21.33	1.557	2.265	C39
0.96	1.27785	0.196065	2.215	0.3101	21.31	1.5162	2.665	C39
0.24	1.08971	0.25557	1.72	0.2408	23.28	1.1473	1.72	C39
0.26	0.765274	0.38264	1.89	0.3402	22.36	1.7775	1.89	I65
0.3	0.890607	0.329878	1.825	0.292	22.42	1.4784	1.825	I65
0.34	1.04805	0.270146	1.885	0.3393	22.57	1.6443	1.885	I65

0.38	1.19732	0.220273	1.915	0.3447	22.26	1.7343	2.415	I65
0.64	0.711107	0.406855	5.22	0.7308	21.2	1.9187	7.22	M73
0.59	0.744163	0.391977	5.65	0.7345	21.59	1.78165	7.15	M73
0.55	0.887096	0.331293	6.18	0.8652	22.04	2.0587	8.18	M73
0.55	1.0209	0.279924	7.345	1.24865	22.25	3.09485	11.845	M73
0.46	1.1584	0.232645	7.73	1.9325	22.75	5.80125	17.23	M73
0.55	0.673577	0.424127	7.345	1.24865	22.25	3.22065	11.845	M73
0.46	0.82795	0.355681	7.73	1.9325	22.75	5.99375	17.23	M73
0.32	1.02414	0.278746	2.875	0.5175	21.68	2.2491	4.375	R85
0.32	1.14175	0.238074	2.665	0.61295	21.31	2.87385	4.665	R85
0.44	0.408659	0.557213	1.33	0.2261	21.99	1.4943	1.33	I65
0.53	0.569704	0.474008	1.355	0.23035	22.07	1.4943	1.355	I65
0.71	0.738622	0.394449	2.805	0.4488	20.92	1.6072	2.805	I65
0.52	0.699366	0.412215	2.8	0.448	21.48	1.6072	2.8	I65
0.42	1.20365	0.218303	2.36	0.4012	22.13	1.6167	2.36	I65
0.51	0.917918	0.318992	1.84	0.3128	21.91	1.59715	1.84	C39
0.57	1.25886	0.201605	1.845	0.31365	21.98	1.59715	1.845	C39
0.67	0.803879	0.365899	1.71	0.2394	21.16	1.1347	1.71	I65
0.68	0.963747	0.301219	1.72	0.2408	21.3	1.2047	2.22	I65
0.64	1.13407	0.240607	1.775	0.284	21.41	1.4032	1.775	I65
0.6	1.32221	0.183526	1.895	0.3411	21.61	1.7946	1.895	I65
0.48	1.08502	0.257186	2.8	0.448	21.21	1.7584	3.8	C35
0.44	1.26683	0.199268	2.835	0.48195	21.38	1.8683	3.835	C35
0.71	0.964889	0.300784	1.205	0.1687	20.75	1.0598	1.205	C39
0.71	1.04744	0.270365	1.405	0.2529	20.85	1.3599	1.405	C39
0.52	1.21694	0.214203	1.37	0.2329	21.23	1.5606	1.37	C38
0.88	0.835528	0.352499	1.95	0.3705	21.31	2.109	2.45	C35
0.95	0.941207	0.309883	2.46	0.4674	21.58	2.2572	2.91	C35
0.8	1.04908	0.269781	2.96	0.5624	21.69	2.32275	3.56	C35
0.76	1.31749	0.184836	2.965	0.56335	21.78	2.5745	4.565	C35
0.67	0.688698	0.41712	4.875	0.8775	22.08	2.889	7.875	I64
0.47	0.767266	0.381765	4.875	0.8775	22.56	2.889	7.875	I64
0.37	0.873608	0.336764	4.94	0.9386	23.21	3.14355	8.44	I64
0.34	1.03105	0.276243	5.065	1.06365	22.9	3.6183	9.565	I64
0.39	1.16133	0.2317	5.19	1.2456	23.05	4.3752	10.69	I64
0.47	1.032	0.2759	2.165	0.28145	22.09	1.4196	2.665	N77
0.52	1.10146	0.25155	2.165	0.28145	22.6	1.46185	2.665	N77
0.41	1.32604	0.18247	2.5	0.25	22.94	1.1035	2.5	N77
0.41	1.31269	0.186171	2.5	0.25	22.94	1.1035	2.5	N77
0.44	1.12783	0.242678	2.43	0.4617	21.49	2.1375	2.88	C37
0.64	1.26295	0.200403	5.925	1.12575	21.64	3.192	8.425	C37
0.64	1.0673	0.263346	5.925	1.12575	21.64	3.18345	8.425	C37
1.8	0.956231	0.304091	2.42	0.4356	22.61	2.0106	2.87	C36
0.95	1.20826	0.216874	2.93	0.5567	22.65	2.14605	2.93	C36
0.46	1.10982	0.248713	1.39	0.2502	21.36	1.692	1.39	C26
0.79	1.1636	0.230966	1.87	0.3179	24.3	1.64815	1.87	C39
0.7	1.36441	0.172117	1.865	0.31705	24.49	1.67025	1.865	C39
0.66	1.50145	0.13846	1.88	0.3384	24.57	1.7685	1.88	C39
0.8	0.749885	0.389433	1.215	0.1701	21.04	1.0724	1.215	C38
0.77	0.828453	0.355469	1.215	0.1701	21.37	1.0724	1.215	C38
0.75	0.915804	0.319826	1.385	0.2493	22.02	1.3194	1.385	C38

0.59	1.08591	0.256877	1.395	0.2511	22.34	1.2537	1.395	C38
0.63	1.23517	0.20866	1.38	0.2484	22.41	1.2537	1.38	C38
0.55	0.818719	0.359579	2.45	0.4655	21.95	2.0919	2.45	C27
0.64	0.931392	0.313702	2.455	0.46645	22.18	2.0729	2.455	C27
0.79	1.1015	0.251536	2.465	0.46835	22.25	2.12135	2.465	C27
0.75	1.24443	0.205884	1.975	0.395	22.04	2.133	1.975	C27
0.76	1.29966	0.18983	2.86	0.4862	22.64	1.802	2.86	I65
0.23	1.07707	0.259938	6	1.2	23.28	3.81	11	I64
0.18	1.34597	0.177042	2	0.2	22.79	1.079	2	I64
0.36	1.08932	0.255704	3.2	0.448	21.79	1.5449	4.2	F52
1.11	1.16261	0.231285	3.8	0.988	21.95	3.6491	7.8	F52
0.43	1.17275	0.228034	1.385	0.2493	20.9	1.6821	1.385	I65
0.76	0.686705	0.418039	1.91	0.3438	20.9	1.9107	2.41	I65
0.54	0.765274	0.38264	2.425	0.4365	21.37	1.9917	2.875	I65
0.51	0.877946	0.334999	2.885	0.5193	21.7	2.1321	3.885	I65
0.56	1.04805	0.270146	3.88	0.6984	21.68	2.3949	5.38	I65
0.67	1.19732	0.220273	4.875	0.8775	21.76	2.5929	6.475	I65
0.6	0.320505	0.605666	1.895	0.3411	20.77	1.8765	2.395	C36
0.6	0.499952	0.509185	1.89	0.3402	20.88	1.8783	2.39	C36
0.57	0.687863	0.417505	1.895	0.3411	20.96	1.971	2.395	C36
0.35	1.18421	0.224391	1.5	0.15	21.41	0.9225	1.5	C34
0.43	1.3898	0.165491	1.5	0.15	21.37	0.9235	1.5	C34
0.79	0.784265	0.37435	1.265	0.18975	20.76	1.21875	1.265	I65
0.53	0.896937	0.327335	1.265	0.18975	20.96	1.21425	1.265	I65
0.58	1.06071	0.265662	1.305	0.2088	21.48	1.3712	1.305	I65
0.65	1.20998	0.216343	1.315	0.2104	21.72	1.3712	1.315	I65
0.6	1.10455	0.250499	1.315	0.2104	20.87	1.3864	1.315	C35
0.52	1.25381	0.203095	1.315	0.2104	21.1	1.3864	1.315	C35
0.45	1.33285	0.180604	1.24	0.186	19.73	1.18875	1.24	C40
0.71	1.06239	0.26507	1.9	0.342	21.69	1.7991	1.9	C40
0.56	1.24421	0.205951	2.465	0.46835	22.17	1.99405	2.465	C40
0.19	1.12248	0.244459	2.295	0.3672	22	1.5096	2.295	C26
0.13	1.27175	0.197835	2.315	0.3704	22.4	1.5096	2.315	C26
1.26	0.924382	0.316447	1.5	0.15	20.51	0.841	1.5	C17
1.28	1.03006	0.276602	1.5	0.15	20.71	0.8215	1.5	C17
1.7	1.13793	0.239331	1.5	0.15	21.07	0.8045	1.5	C17
1.59	1.30505	0.18831	3.8	0.988	21.4	3.6166	7.8	C17
0.84	1.13514	0.240252	2.18	0.3052	21.43	1.1592	2.18	C26
0.91	1.19099	0.222256	1.455	0.27645	20.56	1.91235	1.455	I65
0.35	1.09189	0.25482	1.385	0.2493	20.34	1.6866	1.385	C35
0.75	1.56206	0.125181	1.5	0.15	21.7	0.8695	1.5	C34
0.87	1.19099	0.222256	1.965	0.37335	20.62	2.0197	1.965	I65
0.81	0.503638	0.507293	2.365	0.40205	20.86	1.7272	2.365	C39
0.75	0.60921	0.45468	2.385	0.4293	21.05	1.8666	2.385	C39
0.76	0.77077	0.38023	1.835	0.31195	21.19	1.6014	1.835	C39
0.43	0.897536	0.327095	1.425	0.2565	20.97	1.7595	1.425	C39
0.41	1.09916	0.252334	1.42	0.2556	20.98	1.7505	1.42	C39
1.56	0.917068	0.319327	3.2	0.448	20.64	1.6695	4.2	L72
1.9	0.99962	0.287741	3.915	0.7047	21.04	2.4651	6.415	L72
2.33	1.2617	0.200772	4.94	0.9386	21.24	3.03145	8.44	L72
0.59	1.03485	0.274874	1.345	0.22865	21.06	1.5181	1.345	I65

0.64	1.223	0.212351	1.34	0.2278	21.12	1.5181	1.34	I65
0.56	1.09658	0.253214	1.31	0.2096	21.09	1.3728	1.31	C39
0.56	1.24584	0.205463	1.31	0.2096	21.11	1.3728	1.31	C39
0.31	1.22685	0.211178	1.94	0.3686	21.35	1.97315	1.94	C39
0.8	1.39125	0.16512	2.5	0.5	20.89	2.132	4	C13
0.79	1.427	0.156108	3.335	1.23395	21.04	4.86365	7.335	C13
0.8	0.471361	0.523986	2.5	0.5	20.89	2.132	4	C13
0.79	0.52479	0.496506	3.335	1.23395	21.04	4.8692	7.335	C13
0.45	0.849345	0.34674	4	0.8	22.35	2.645	7	M74
0.49	1.01945	0.280452	4	0.8	22.38	2.634	7	M74
0.5	1.19404	0.221299	5.8	0.928	22.5	2.4272	8.8	M74
0.5	1.16947	0.229083	5.8	0.928	22.5	2.5904	8.8	M74
0.89	1.54834	0.128103	2.75	0.4125	20.84	1.63575	3.75	C24
0.76	1.47484	0.144596	2.5	0.5	21.58	2.216	4	E48
1.56	1.54037	0.129823	1.75	0.2625	21.37	1.4205	2.25	C29
0.85	1.22714	0.211091	3.1	0.372	20.82	1.221	3.6	C21
0.83	1.41528	0.159023	3.2	0.448	20.88	1.5064	4.2	C21
0.73	1.55865	0.125902	3.2	0.448	21.13	1.5064	4.2	C21
0.65	1.29333	0.191626	1.39	0.2502	21.21	1.6965	1.39	I65
1.01	1.07909	0.259235	4.66	0.6058	19.99	1.43195	5.06	C39
0.82	1.3475	0.176629	4.665	0.60645	20.07	1.4352	5.065	C39
0.73	1.1525	0.234562	2.5	0.25	21.45	0.9375	2.5	C30
0.69	1.30176	0.189239	2.5	0.25	21.76	0.9375	2.5	C30
0.48	1.07325	0.261267	5	1	25.03	3.461	9	C38
0.52	1.21618	0.214434	5.06	1.0626	25.34	3.3978	6.66	C38
0.99	1.32218	0.183536	1.36	0.2312	21.37	1.5487	1.36	C39
0.69	1.34281	0.177895	8.27	1.2405	23.97	2.2815	8.27	C35
0.85	1.22405	0.21203	1	0.1	21.72	0.971	1	R85
1.17	0.022753	0.783456	1.5	0.15	25.29	1.1675	1.5	C31
0.94	0.164507	0.696191	1.5	0.15	25.32	1.18	1.5	C31
0.92	0.340945	0.594253	1.5	0.15	25.27	1.1845	1.5	C31
1.04	0.790428	0.371683	4.815	0.7704	25.29	2.6648	7.315	C31
0.61	0.757502	0.386062	4.75	0.7125	25.27	2.42775	6.75	C31
1.04	0.426354	0.547731	4.815	0.7704	25.29	2.6648	7.315	C31
0.61	0.377546	0.574084	4.75	0.7125	25.27	2.42775	6.75	C31
1.04	0.646749	0.436719	4.815	0.7704	25.29	2.6648	7.315	C31
0.61	0.493098	0.512713	4.75	0.7125	25.27	2.42775	6.75	C31
0.41	-0.28794	0.989777	4	2	26.76	9.3975	10	D44
0.41	-0.0656	0.840109	4	2	26.78	9.4125	10	D44
0.3	-0.23165	0.950972	4.165	0.95795	25.31	4.13195	8.165	G55
0.29	-0.25553	0.967358	5.855	2.75185	25.48	12.9015	18.855	G55
0.28	-0.07771	0.848005	6.065	2.48665	25.67	11.3099	18.565	G55
0.26	0.064484	0.757291	6.065	2.48665	25.7	11.3427	18.565	G55
0.25	0.251816	0.644789	6.125	2.63375	25.68	12.141	19.125	G55
0.97	0.177898	0.688188	4.07	0.4477	26.1	1.55485	4.57	C31
0.99	0.33423	0.59799	5.055	0.55605	26.17	1.65165	5.555	C31
0.99	0.450073	0.53515	6.045	0.66495	26.17	1.75065	6.545	C31
0.86	0.661848	0.429607	5.55	0.6105	26.15	1.7622	6.05	C31
0.82	0.82208	0.358157	4.565	0.50215	26.16	1.60875	5.065	C31
0.71	0.782824	0.374976	4.07	0.4477	26.18	1.56145	4.57	C31
0.74	0.861392	0.341765	4.565	0.50215	26.31	1.61205	5.065	C31

0.93	0.961404	0.302113	4.625	0.60125	26.58	2.04685	5.625	C31
0.89	1.13151	0.241454	4.625	0.60125	26.54	2.05335	5.625	C31
0.86	1.28077	0.195222	4.145	0.53885	26.55	1.9981	5.145	C31
0.3	0.667008	0.427191	4.165	0.95795	25.31	4.13195	8.165	G55
0.29	0.565904	0.47589	5.855	2.75185	25.48	12.9015	18.855	G55
0.28	0.688816	0.417065	6.065	2.48665	25.67	11.3099	18.565	G55
0.26	0.886667	0.331466	6.065	2.48665	25.7	11.3427	18.565	G55
0.25	1.05101	0.269094	6.125	2.63375	25.68	12.141	19.125	G55
0.99	0.154304	0.702317	5.055	0.55605	26.17	1.65165	5.555	C31
0.86	0.30904	0.612113	6.045	0.66495	26.09	1.7897	6.545	C31
0.86	0.443747	0.538491	5.55	0.6105	26.15	1.7622	6.05	C31
0.82	0.623719	0.447691	4.565	0.50215	26.16	1.60875	5.065	C31
0.74	1.03177	0.275985	4.565	0.50215	26.31	1.61205	5.065	C31
0.93	1.20487	0.217925	4.625	0.60125	26.58	2.04685	5.625	C31
0.89	1.40845	0.160741	4.625	0.60125	26.54	2.05335	5.625	C31
0.99	0.366237	0.580279	6.045	0.66495	26.17	1.75065	6.545	C31
0.86	0.573343	0.472209	5.55	0.6105	26.15	1.7622	6.05	C31
0.82	0.688512	0.417206	4.565	0.50215	26.16	1.60875	5.065	C31
0.71	0.748056	0.390245	4.07	0.4477	26.18	1.56145	4.57	C31
0.74	0.797081	0.368815	4.565	0.50215	26.31	1.61205	5.065	C31
0.93	0.945509	0.308218	4.625	0.60125	26.58	2.04685	5.625	C31
0.89	1.05056	0.269256	4.625	0.60125	26.54	2.05335	5.625	C31
0.86	1.30892	0.187226	4.145	0.53885	26.55	1.9981	5.145	C31
0.99	0.379875	0.572812	6.045	0.66495	26.17	1.75065	6.545	C31
0.86	0.514999	0.501484	5.55	0.6105	26.15	1.7622	6.05	C31
0.82	0.678401	0.421884	4.565	0.50215	26.16	1.60875	5.065	C31
0.71	0.795852	0.369344	4.07	0.4477	26.18	1.56145	4.57	C31
0.74	0.822577	0.357947	4.565	0.50215	26.31	1.61205	5.065	C31
0.93	0.95918	0.302963	4.625	0.60125	26.58	2.04685	5.625	C31
0.89	1.11198	0.247986	4.625	0.60125	26.54	2.05335	5.625	C31
0.86	1.30012	0.1897	4.145	0.53885	26.55	1.9981	5.145	C31
0.45	0.644268	0.437894	2.5	0.25	24.28	1.2595	2.5	D44
0.39	0.766518	0.382093	4.285	0.6856	24.58	2.5224	6.285	D44
0.34	0.676618	0.422712	4.715	1.1316	24.67	4.5276	9.715	D44
0.3	0.767847	0.38151	5.61	1.2342	24.74	4.4671	11.11	D44
0.27	0.842538	0.34957	5.89	1.6492	25.12	6.4036	13.89	D44
0.25	0.999984	0.287606	5.5	1.65	25.32	6.7665	13.5	D44
0.22	1.14292	0.237692	5.07	1.5717	25.43	6.71305	12.57	D44
0.08	0.672974	0.424408	2.5	0.25	25.85	1.213	2.5	D44
0.09	0.837554	0.351651	2.5	0.25	25.85	0.9875	2.5	D44
0.12	1.08932	0.255701	2.5	0.25	25.84	1.22	2.5	D44
0.39	0.366171	0.580315	4.285	0.6856	24.58	2.5224	6.285	D44
0.34	0.108597	0.73006	4.715	1.1316	24.67	4.5276	9.715	D44
0.3	0.384061	0.57053	5.61	1.2342	24.74	4.4671	11.11	D44
0.27	0.380977	0.572211	5.89	1.6492	25.12	6.4036	13.89	D44
0.25	0.577831	0.469997	5.5	1.65	25.32	6.7665	13.5	D44
0.22	0.860569	0.342104	5.07	1.5717	25.43	6.71305	12.57	D44
0.09	0.480867	0.519041	2.5	0.25	25.85	0.9875	2.5	D44
0.12	0.641913	0.439011	2.5	0.25	25.84	1.22	2.5	D44
1.53	0.910013	0.32212	3.5	0.35	28.1	1.4905	3.5	B07
1.81	1.02746	0.277541	3.5	0.35	28.1	1.4955	3.5	B07

0.08	0.31393	0.609359	2.5	0.25	25.85	1.213	2.5	D44
0.1	-0.12177	0.87699	6.43	3.7937	26.9	18.0392	23.43	J67
0.1	-0.00196	0.799131	6.5	3.9	27.15	18.684	24	J67
0.06	0.098644	0.736165	6.875	3.9875	27.12	19.1458	25.875	J67
0.07	0.214402	0.66659	6.43	3.7937	27.16	18.2693	23.43	J67
0.06	0.299418	0.61755	6.835	3.21245	27.21	14.7416	23.335	J67
0.06	0.49338	0.512568	6.89	3.3072	27.4	15.2928	23.89	J67
1.28	-0.01729	0.808925	6	0.6	24.17	1.3855	6	C35
1.22	0.150385	0.704677	5.55	0.6105	24.66	1.52405	6.05	C35
0.81	0.204391	0.672481	6.225	0.93375	24.89	2.412	8.725	C35
0.58	0.335542	0.597259	6.225	0.93375	24.88	2.35125	8.725	C35
0.48	0.437375	0.541866	6.665	0.86645	24.87	2.03775	8.665	C35
0.37	0.653338	0.433608	6.18	0.8652	24.84	2.2057	8.18	C35
0.38	0.886171	0.331666	5.055	0.55605	24.84	1.47125	5.555	C35
0.64	0.99185	0.290628	5.055	0.55605	24.79	1.5763	5.555	C35
0.84	1.09972	0.25214	5.055	0.55605	25.02	1.6225	5.555	C35
0.92	1.3618	0.172808	3.1	0.372	25.23	1.365	3.6	C35
0.06	0.469258	0.525084	6.875	3.9875	27.12	19.1458	25.875	J67
0.07	0.612191	0.453239	6.43	3.7937	27.16	18.2693	23.43	J67
0.06	0.840953	0.350231	6.835	3.21245	27.21	14.7416	23.335	J67
0.06	1.02277	0.279245	6.89	3.3072	27.4	15.2928	23.89	J67
0.06	-0.08482	0.852654	6.89	3.3072	27.4	15.2928	23.89	J67
0.3	-0.1315	0.883444	5.61	1.2342	24.74	4.4671	11.11	D44
0.27	0.019838	0.785298	5.89	1.6492	25.12	6.4036	13.89	D44
0.25	0.00209	0.796554	5.5	1.65	25.32	6.7665	13.5	D44
0.22	0.159162	0.699397	5.07	1.5717	25.43	6.71305	12.57	D44
0.57	0.963489	0.301317	2.625	0.34125	25.3	1.61005	3.125	E48
0.4	0.129019	0.717604	3.2	0.448	26.82	1.6149	4.2	E48
0.46	0.285351	0.625541	3.2	0.448	26.85	1.6149	4.2	E48
0.51	0.401194	0.561237	3.2	0.448	26.97	1.6184	4.2	E48
0.56	0.6193	0.449814	3.665	0.47645	27	1.56	4.665	E48
0.53	0.773201	0.379167	5.22	0.7308	27.03	2.2848	7.22	E48
0.48	0.733945	0.396542	5.22	0.7308	27.15	2.4514	7.22	E48
0.44	0.818843	0.359527	6.135	0.79755	27.15	2.32895	7.635	E48
0.46	0.937846	0.311187	7.605	0.9126	27.08	2.3196	9.105	E48
0.52	1.10162	0.251493	8.135	1.05755	27.02	2.6455	10.135	E48
0.52	1.25089	0.203962	8.135	1.05755	27.06	2.64745	10.135	E48
0.66	-0.14538	0.892683	1.5	0.15	22.4	0.995	1.5	F51
0.73	0.068684	0.75468	1.5	0.15	22.41	0.994	1.5	F51
0.5	0.225745	0.659945	1.5	0.15	22.59	0.9805	1.5	F51
0.39	0.387615	0.568597	1.5	0.15	22.77	0.972	1.5	F51
0.56	-0.09215	0.857462	3.665	0.47645	27	1.56	4.665	E48
0.53	0.016621	0.787333	5.22	0.7308	27.03	2.2848	7.22	E48
0.48	0.221231	0.662586	5.22	0.7308	27.15	2.4514	7.22	E48
0.44	0.463694	0.527993	6.135	0.79755	27.15	2.32895	7.635	E48
0.46	0.654335	0.433138	7.605	0.9126	27.08	2.3196	9.105	E48
0.52	0.812585	0.362184	8.135	1.05755	27.02	2.6455	10.135	E48
0.52	1.06436	0.26438	8.135	1.05755	27.06	2.64745	10.135	E48
0.44	0.707818	0.408353	6.135	0.79755	27.15	2.32895	7.635	E48
0.46	0.831599	0.354146	7.605	0.9126	27.08	2.3196	9.105	E48
0.52	0.942719	0.309297	8.135	1.05755	27.02	2.6455	10.135	E48

0.52	1.13963	0.238774	8.135	1.05755	27.06	2.64745	10.135	E48
0.46	0.924726	0.316312	7.605	0.9126	27.08	2.3196	9.105	E48
0.52	0.967958	0.299618	8.135	1.05755	27.02	2.6455	10.135	E48
0.52	1.22632	0.21134	8.135	1.05755	27.06	2.64745	10.135	E48
0.46	0.935622	0.312053	7.605	0.9126	27.08	2.3196	9.105	E48
0.52	1.08209	0.258197	8.135	1.05755	27.02	2.6455	10.135	E48
0.52	1.27023	0.198275	8.135	1.05755	27.06	2.64745	10.135	E48
0.52	0.455898	0.532083	8.135	1.05755	27.02	2.6455	10.135	E48
0.52	0.616944	0.450947	8.135	1.05755	27.06	2.64745	10.135	E48
4.52	1.12945	0.242139	4.6	1.932	24.91	8.5176	12.6	L72
0.52	1.37578	0.169127	8.135	1.05755	27.02	2.6455	10.135	E48
0.52	1.51916	0.134481	8.135	1.05755	27.06	2.64745	10.135	E48
1.29	1.09025	0.255383	3.835	0.65195	24.1	2.3902	5.835	C39
1.16	1.25217	0.203581	4.75	0.7125	24.1	2.259	6.75	C39
1.16	0.25113	0.645186	4.75	0.7125	24.1	2.24325	6.75	C39
1.09	0.746734	0.390832	3	0.3	24.28	1.149	3	C36
0.93	0.864185	0.340618	4	0.4	24.31	1.2685	4	C36
0.86	0.981635	0.29445	5	0.5	24.33	1.3565	5	C36
0.82	1.17854	0.226187	5	0.5	24.32	1.3615	5	C36
0.71	-0.32038	1.01241	1.5	0.15	25.74	1.186	1.5	E48
0.62	-0.18408	0.918646	2	0.2	25.95	1.228	2	E48
0.49	-0.04071	0.823978	1.5	0.15	26.11	1.234	1.5	E48
0.73	0.84979	0.346556	1	0.1	20.68	0.864	1	C40
0.83	0.962462	0.301709	1	0.1	20.65	0.857	1	C40
0.98	-0.35101	1.03395	1	0.1	21.49	1.0405	1	C14
1.78	-0.23584	0.953834	1.5	0.15	22.17	1.049	1.5	C14
1.64	-0.36841	1.04627	1.5	0.15	22.38	1.034	1.5	C14
1.91	-0.38577	1.05861	1.5	0.15	22.44	1.0335	1.5	C14
1.77	-0.22237	0.944631	1.5	0.15	22.57	1.037	1.5	C14
1.74	-0.12495	0.879096	1.5	0.15	22.76	1.039	1.5	C14
1.43	0.084467	0.744901	1.5	0.15	23.17	1.051	1.5	C14
0.55	-0.1746	0.912258	4.94	0.9386	24	3.2243	8.44	C27
0.5	0.068186	0.754989	9.89	1.7802	24.29	4.5891	16.89	C27
0.52	0.235122	0.654474	10.42	1.8756	24.29	4.8402	18.42	C27
0.61	0.392183	0.566115	9.915	1.7847	24.28	4.6665	17.415	C27
1.12	0.779191	0.376554	1	0.1	22.18	1.017	1	C36
1.15	0.85776	0.343261	1	0.1	22.28	1.0185	1	C36
1.19	0.970433	0.298678	1	0.1	22.42	1.0265	1	C36
1.18	1.14054	0.238473	1	0.1	22.49	1.032	1	C36
1.07	1.2898	0.192632	1	0.1	22.56	1.0355	1	C36
0.73	0.060302	0.759896	4	0.4	22.43	1.148	4	C27
0.94	0.226061	0.65976	3.5	0.35	22.72	1.069	3.5	C27
0.87	0.394176	0.565035	3.5	0.35	22.99	1.0815	3.5	C27
0.8	0.5293	0.494222	3	0.3	23.2	1.0295	3	C27
0.77	0.680041	0.421123	3.585	0.4302	23.28	1.3416	4.085	C27
0.78	0.7785	0.376855	3.75	0.5625	23.39	1.83	5.25	C27
0.76	0.805226	0.365323	3.75	0.5625	23.56	1.81875	5.25	C27
0.74	0.96715	0.299924	3.665	0.47645	23.65	1.51125	4.665	C27
0.72	1.11995	0.245307	3.665	0.47645	23.74	1.4885	4.665	C27
0.73	-0.27251	0.979076	4	0.4	22.43	1.148	4	C27
0.94	-0.2069	0.934097	3.5	0.35	22.72	1.069	3.5	C27

0.87	-0.10123	0.863427	3.5	0.35	22.99	1.0815	3.5	C27
0.8	0.000903	0.79731	3	0.3	23.2	1.0295	3	C27
0.77	0.156357	0.701082	3.585	0.4302	23.28	1.3416	4.085	C27
0.78	0.02388	0.782744	3.75	0.5625	23.39	1.83	5.25	C27
0.76	0.143687	0.708718	3.75	0.5625	23.56	1.81875	5.25	C27
0.74	0.32682	0.602128	3.665	0.47645	23.65	1.51125	4.665	C27
0.72	0.493756	0.512374	3.665	0.47645	23.74	1.4885	4.665	C27
0.64	0.635811	0.441912	3.3	0.528	23.76	1.8416	4.8	C27
0.64	1.26975	0.198415	3.3	0.528	23.76	1.8416	4.8	C27
0.73	-0.11444	0.872135	4	0.4	22.43	1.148	4	C27
0.94	0.125982	0.71945	3.5	0.35	22.72	1.069	3.5	C27
0.87	0.380538	0.572451	3.5	0.35	22.99	1.0815	3.5	C27
0.8	0.532158	0.492778	3	0.3	23.2	1.0295	3	C27
0.77	0.631891	0.443781	3.585	0.4302	23.28	1.3416	4.085	C27
0.78	0.677993	0.422074	3.75	0.5625	23.39	1.83	5.25	C27
0.76	0.785278	0.373911	3.75	0.5625	23.56	1.81875	5.25	C27
0.74	0.956254	0.304083	3.665	0.47645	23.65	1.51125	4.665	C27
0.72	1.00582	0.285451	3.665	0.47645	23.74	1.4885	4.665	C27
0.64	1.24518	0.205659	3.3	0.528	23.76	1.8416	4.8	C27
0.53	-0.14811	0.894501	1	0.1	24.46	1.096	1	C38
0.63	0.010582	0.79116	1.5	0.15	24.65	1.118	1.5	C38
0.65	0.189291	0.681413	1.5	0.15	24.81	1.1065	1.5	C38
0.58	0.282438	0.627202	1.5	0.15	24.98	1.1085	1.5	C38
0.55	0.442305	0.539254	1.5	0.15	25.04	1.1115	1.5	C38
0.48	0.578253	0.469788	1.5	0.15	25.15	1.116	1.5	C38
0.44	0.673991	0.423934	2	0.2	25.25	1.1495	2	C38
0.37	0.845352	0.348399	3	0.3	25.35	1.254	3	C38
0.73	-0.00442	0.800701	4	0.4	22.43	1.148	4	C27
0.94	0.030723	0.778429	3.5	0.35	22.72	1.069	3.5	C27
0.87	0.172477	0.691423	3.5	0.35	22.99	1.0815	3.5	C27
0.8	0.348915	0.589832	3	0.3	23.2	1.0295	3	C27
0.77	0.603022	0.457679	3.585	0.4302	23.28	1.3416	4.085	C27
0.78	0.633913	0.442816	3.75	0.5625	23.39	1.83	5.25	C27
0.76	0.822048	0.358171	3.75	0.5625	23.56	1.81875	5.25	C27
0.74	1.10971	0.24875	3.665	0.47645	23.65	1.51125	4.665	C27
0.72	1.17917	0.225989	3.665	0.47645	23.74	1.4885	4.665	C27
0.37	0.998421	0.288185	3	0.3	25.35	1.254	3	C38
1.2	0.457265	0.531365	2	0.2	24.91	1.109	2	C26
0.74	0.890969	0.329732	3	0.3	24.93	1.228	3	C26
0.77	1.03818	0.273678	3.1	0.372	24.94	1.5132	3.6	C26
0.85	1.22116	0.212911	3.1	0.372	24.88	1.5276	3.6	C26
0.8	1.42196	0.157356	3.1	0.372	24.94	1.5462	3.6	C26
0.84	1.56534	0.12449	3.1	0.372	24.82	1.5546	3.6	C26
0.74	0.286403	0.624942	3	0.3	24.93	1.228	3	C26
0.77	0.509875	0.5041	3.1	0.372	24.94	1.5132	3.6	C26
0.85	0.694186	0.414593	3.1	0.372	24.88	1.5276	3.6	C26
0.8	0.858766	0.342846	3.1	0.372	24.94	1.5462	3.6	C26
0.84	1.11054	0.248472	3.1	0.372	24.82	1.5546	3.6	C26
0.78	-0.53819	1.16914	1	0.1	23.94	1.1165	1	C39
0.76	-0.42028	1.08329	1	0.1	24.13	1.172	1	C39
0.69	-0.39057	1.06202	1	0.1	24.24	1.178	1	C39

0.59	-0.3176	1.01046	2	0.2	24.47	1.2255	2	C39
0.56	-0.11724	0.873988	2.5	0.25	24.63	1.274	2.5	C39
0.53	0.050877	0.76578	3	0.3	24.76	1.318	3	C39
0.47	0.158323	0.699901	3.1	0.372	24.78	1.6566	3.6	C39
0.43	0.352509	0.587843	3.1	0.372	24.88	1.6782	3.6	C39
0.39	0.531957	0.492879	3.1	0.372	24.88	1.6752	3.6	C39
0.36	0.719868	0.402881	3.1	0.372	24.85	1.6764	3.6	C39
0.78	0.168288	0.693927	1	0.1	23.94	1.1165	1	C39
0.76	0.32462	0.603359	1	0.1	24.13	1.172	1	C39
0.69	0.440464	0.540229	1	0.1	24.24	1.178	1	C39
0.59	0.652238	0.434126	2	0.2	24.47	1.2255	2	C39
0.56	0.81247	0.362233	2.5	0.25	24.63	1.274	2.5	C39
0.78	-0.12745	0.880753	1	0.1	23.94	1.1165	1	C39
0.76	0.086617	0.743573	1	0.1	24.13	1.172	1	C39
0.69	0.243678	0.649502	1	0.1	24.24	1.178	1	C39
0.59	0.405548	0.558888	2	0.2	24.47	1.2255	2	C39
0.56	0.412124	0.555349	2.5	0.25	24.63	1.274	2.5	C39
0.53	0.205193	0.672009	3	0.3	24.76	1.318	3	C39
0.47	0.455335	0.532379	3.1	0.372	24.78	1.6566	3.6	C39
0.43	0.490234	0.514192	3.1	0.372	24.88	1.6782	3.6	C39
0.39	0.699748	0.41204	3.1	0.372	24.88	1.6752	3.6	C39
0.36	0.988816	0.29176	3.1	0.372	24.85	1.6764	3.6	C39
0.43	0.129783	0.71714	3.1	0.372	24.88	1.6782	3.6	C39
0.39	0.271976	0.633184	3.1	0.372	24.88	1.6752	3.6	C39
0.36	0.471969	0.52367	3.1	0.372	24.85	1.6764	3.6	C39
1.63	0.463582	0.528052	2.875	0.5175	26.65	2.7333	4.375	C36
1.59	0.607628	0.455446	2.625	0.34125	26.75	1.85185	3.125	C36
1.44	0.679051	0.421583	3.1	0.372	26.96	1.7514	3.6	C36
1.36	0.780006	0.3762	4.145	0.53885	27.1	2.0748	5.145	C36
1.31	0.938321	0.311003	4.625	0.60125	27.31	2.1359	5.625	C36
1.19	0.981553	0.294482	4.69	0.6566	27.39	2.3737	6.19	C36
1.03	1.2589	0.201593	4.07	0.4477	27.47	1.70225	4.57	C36
0.75	0.689653	0.416679	1	0.1	21.94	1.079	1	C38
0.68	0.854233	0.344717	1.5	0.15	22.14	1.1035	1.5	C38
0.65	1.02353	0.27897	1.5	0.15	22.27	1.1035	1.5	C38
0.63	1.20887	0.216687	1.5	0.15	22.42	1.108	1.5	C38
0.43	0.977549	0.295988	3.1	0.372	24.88	1.6782	3.6	C39
0.39	1.08542	0.257045	3.1	0.372	24.88	1.6752	3.6	C39
0.36	1.35383	0.174931	3.1	0.372	24.85	1.6764	3.6	C39
0.56	0.611335	0.453653	2.5	0.25	24.63	1.274	2.5	C39
0.53	0.865067	0.340256	3	0.3	24.76	1.318	3	C39
0.47	1.01227	0.283077	3.1	0.372	24.78	1.6566	3.6	C39
0.43	1.19526	0.220918	3.1	0.372	24.88	1.6782	3.6	C39
0.39	1.39606	0.163886	3.1	0.372	24.88	1.6752	3.6	C39
0.36	1.53943	0.130026	3.1	0.372	24.85	1.6764	3.6	C39
1.44	0.634971	0.442312	3.1	0.372	26.96	1.7514	3.6	C36
1.36	0.816776	0.360403	4.145	0.53885	27.1	2.0748	5.145	C36
1.31	1.09178	0.254857	4.625	0.60125	27.31	2.1359	5.625	C36
1.19	1.1549	0.23378	4.69	0.6566	27.39	2.3737	6.19	C36
1.03	1.37949	0.168161	4.07	0.4477	27.47	1.70225	4.57	C36
1.36	0.799954	0.367581	4.145	0.53885	27.1	2.0748	5.145	C36

1.31	0.977197	0.296121	4.625	0.60125	27.31	2.1359	5.625	C36
1.19	1.07874	0.259358	4.69	0.6566	27.39	2.3737	6.19	C36
1.03	1.36614	0.171661	4.07	0.4477	27.47	1.70225	4.57	C36
0.62	0.852241	0.345541	1	0.1	23.11	0.8835	1	C39
0.71	1.02153	0.279694	1	0.1	23	0.923	1	C39
0.75	1.20687	0.217303	1	0.1	23.07	0.9395	1	C39
0.58	1.46424	0.147095	1	0.1	23.18	0.9485	1	C39
0.89	-0.23029	0.950038	2	0.4	23.01	2.359	3	J69
1	-0.12816	0.88122	2.5	0.5	23.18	2.538	4	J69
0.9	0.020969	0.784583	3.125	0.71875	23.14	3.1073	5.625	J69
0.68	-0.07353	0.845274	3.5	0.7	23.15	2.768	6	J69
1	0.294018	0.620612	2.5	0.5	23.18	2.538	4	J69
0.9	0.271118	0.633676	3.125	0.71875	23.14	3.1073	5.625	J69
0.68	0.332152	0.599149	3.5	0.7	23.15	2.768	6	J69
0.39	0.359809	0.583815	1.5	0.15	23.15	1.087	1.5	J69
0.64	0.596777	0.460716	1.75	0.2625	23.67	1.78275	2.25	J69
0.57	0.694208	0.414582	1.75	0.2625	23.63	1.77525	2.25	J69
0.58	0.901998	0.32531	1.75	0.2625	23.8	1.791	2.25	J69
0.9	-0.06825	0.841834	3.125	0.71875	23.14	3.1073	5.625	J69
0.68	0.155353	0.701686	3.5	0.7	23.15	2.768	6	J69
0.68	0.759919	0.384995	3.5	0.7	23.15	2.768	6	J69
0.81	-0.09252	0.857701	1	0.1	21.12	0.951	1	C13
0.99	0.1479	0.706175	1	0.1	21.05	0.916	1	C13
1.07	0.402456	0.560556	1	0.1	21.03	0.917	1	C13
1.08	0.554075	0.481774	1	0.1	21.07	0.922	1	C13
1.2	0.666469	0.427443	1	0.1	21.04	0.8965	1	C13
1.59	0.731563	0.397611	1	0.1	21.13	0.897	1	C13
1.33	0.838848	0.35111	1	0.1	21.32	0.9055	1	C13
1.43	0.997163	0.288652	1	0.1	21.44	0.9065	1	C13
1.42	0.983421	0.29378	1.5	0.3	21.51	1.915	2	C13
0.98	0.4787	0.520166	2	0.2	21.83	1.099	2	F51
1.32	0.631359	0.444035	3.75	0.5625	21.88	2.0265	5.25	F51
1.45	0.80227	0.366588	4.69	0.6566	21.83	2.0104	6.19	F51
1.24	1.05404	0.26802	4.215	0.5901	22.07	1.9488	5.715	F51
1.32	0.315586	0.608428	3.75	0.5625	21.88	2.0265	5.25	F51
1.45	0.501364	0.50846	4.69	0.6566	21.83	2.0104	6.19	F51
1.24	0.689274	0.416854	4.215	0.5901	22.07	1.9488	5.715	F51
1.32	0.261273	0.639333	3.75	0.5625	21.88	2.0265	5.25	F51
1.45	0.490037	0.514293	4.69	0.6566	21.83	2.0104	6.19	F51
1.24	0.55918	0.47923	4.215	0.5901	22.07	1.9488	5.715	F51
1.13	0.725393	0.400386	1.5	0.15	22.98	1.118	1.5	L72
1	0.803961	0.365864	1.5	0.15	23.12	1.127	1.5	L72
1.11	0.379149	0.573209	1.5	0.15	23.31	0.9445	1.5	C29
1.19	0.468321	0.525573	1.5	0.15	23.34	0.9345	1.5	C29
1.1	0.652632	0.43394	3.3	0.528	23.41	2	4.8	C29
1.02	0.904457	0.32433	4	0.4	23.73	1.263	4	C29
1.12	1.01014	0.283862	4.5	0.45	23.76	1.318	4.5	C29
1.17	1.11801	0.245957	4.5	0.45	23.84	1.3685	4.5	C29
1.13	1.38642	0.166365	4.5	0.45	24.1	1.3555	4.5	C29
1.02	0.812945	0.36203	4	0.4	23.73	1.263	4	C29
1.12	0.97126	0.298365	4.5	0.45	23.76	1.318	4.5	C29

1.17	1.02082	0.279953	4.5	0.45	23.84	1.3685	4.5	C29
1.13	1.27918	0.195682	4.5	0.45	24.1	1.4055	4.5	C29
0.44	0.430014	0.54578	3.3	0.528	23.35	1.9464	4.8	C39
0.49	0.464912	0.527356	3.3	0.528	23.3	1.9704	4.8	C39
0.54	0.693418	0.414946	6.625	0.86125	23.16	2.0943	8.125	C39
1.03	0.168288	0.693927	1	0.1	22.05	1.03	1	C39
1.26	0.32462	0.603359	1	0.1	21.9	1.0275	1	C39
0.97	0.440464	0.540229	1	0.1	21.76	1.0305	1	C39
0.89	0.652238	0.434126	1	0.1	21.58	1.0285	1	C39
0.6	0.81247	0.362233	1.5	0.15	21.29	1.039	1.5	C39
1.29	0.527754	0.495004	5.415	2.5992	25.32	11.5896	16.915	F51
1.27	0.503837	0.507191	6.285	3.5196	25.56	16.4332	22.285	F51
1.44	0.672091	0.424819	6.625	3.51125	25.89	16.3452	23.625	F51
0.54	0.981905	0.294349	6.625	0.86125	23.16	2.0943	8.125	C39
0.61	1.24026	0.207131	6.625	0.86125	23.12	2.10795	8.125	C39
0.54	1.07909	0.259235	6.625	0.86125	23.16	2.0943	8.125	C39
0.61	1.3475	0.176629	6.625	0.86125	23.12	2.10795	8.125	C39
0.54	0.956667	0.303924	6.625	0.86125	23.16	2.0943	8.125	C39
0.61	1.15357	0.234211	6.625	0.86125	23.12	2.10795	8.125	C39
0.54	0.525627	0.496082	6.625	0.86125	23.16	2.0943	8.125	C39
0.61	0.713537	0.405751	6.625	0.86125	23.12	2.10795	8.125	C39
0.61	0.982486	0.294131	6.625	0.86125	23.12	2.10795	8.125	C39
0.61	1.28418	0.194242	6.625	0.86125	23.12	2.10795	8.125	C39
2.52	0.346128	0.591376	2	0.2	23.93	1.1	2	C36
1.95	0.479934	0.519525	2.625	0.34125	24.06	1.57755	3.125	C36
1.33	0.542028	0.487806	4.145	0.53885	24.22	1.82325	5.145	C36
1.04	0.599388	0.459445	4.07	0.4477	24.2	1.49985	4.57	C36
1	0.65243	0.434036	4.07	0.4477	24.27	1.51305	4.57	C36
0.87	0.529304	0.49422	5.7	0.798	24.48	2.3282	7.7	C36
0.95	0.721413	0.402183	5.7	0.798	24.71	2.3324	7.7	C36
0.88	0.838863	0.351104	5.7	0.798	24.86	2.3464	7.7	C36
0.67	0.924662	0.316338	7.46	1.4174	24.8	4.2408	13.46	C36
0.83	1.13423	0.240554	6.365	1.08205	24.7	3.32775	10.365	C36
2.52	0.174265	0.690355	2	0.2	23.93	1.1	2	C36
1.95	0.311607	0.610667	2.625	0.34125	24.06	1.57755	3.125	C36
1.33	0.42745	0.547146	4.145	0.53885	24.22	1.82325	5.145	C36
1.04	0.651886	0.434292	4.07	0.4477	24.2	1.49985	4.57	C36
1	0.812117	0.362383	4.07	0.4477	24.27	1.51305	4.57	C36
0.87	0.75387	0.387667	5.7	0.798	24.48	2.3282	7.7	C36
0.95	0.832438	0.353794	5.7	0.798	24.71	2.3324	7.7	C36
0.88	0.945111	0.308372	5.7	0.798	24.86	2.3464	7.7	C36
0.67	1.08357	0.257686	7.46	1.4174	24.8	4.2408	13.46	C36
0.83	1.24549	0.205568	6.365	1.08205	24.7	3.32775	10.365	C36
0.87	0.66639	0.42748	5.7	0.798	24.48	2.3282	7.7	C36
0.95	0.773676	0.378959	5.7	0.798	24.71	2.3324	7.7	C36
0.88	0.93199	0.313468	5.7	0.798	24.86	2.3464	7.7	C36
0.67	0.949901	0.306524	7.46	1.4174	24.8	4.2408	13.46	C36
0.83	1.22092	0.212984	6.365	1.08205	24.7	3.32775	10.365	C36
0.61	0.156715	0.700867	1.5	0.15	23.65	0.762	1.5	C35
0.48	0.236043	0.653938	1.5	0.15	23.75	0.75	1.5	C35
0.24	0.67866	0.421764	2	0.2	24.07	0.863	2	C35

0.15	0.892502	0.329115	2	0.2	24.09	0.8855	2	C35
0.24	0.99818	0.288274	2	0.2	24.14	0.8855	2	C35
0.21	1.10605	0.249988	2	0.2	24.18	0.892	2	C35
0.21	1.37446	0.169473	2	0.2	24.24	1.022	2	C35
1.04	0.575531	0.47113	4	0.8	25.49	3.053	7	E48
0.82	0.911067	0.321702	5.9	1.062	26.1	3.3624	9.9	E48
1.95	0.135732	0.713531	2.625	0.34125	24.06	1.57755	3.125	C36
1.33	0.21506	0.666204	4.145	0.53885	24.22	1.82325	5.145	C36
1.04	0.358871	0.584332	4.07	0.4477	24.2	1.49985	4.57	C36
1	0.448043	0.536221	4.07	0.4477	24.27	1.51305	4.57	C36
0.87	0.651345	0.434547	5.7	0.798	24.48	2.3282	7.7	C36
0.95	0.865188	0.340207	5.7	0.798	24.71	2.3324	7.7	C36
0.88	0.970866	0.298514	5.7	0.798	24.86	2.3464	7.7	C36
0.67	1.04709	0.27049	7.46	1.4174	24.8	4.2408	13.46	C36
0.83	1.32815	0.18189	6.365	1.08205	24.7	3.32775	10.365	C36
1.33	0.135553	0.713639	4.145	0.53885	24.22	1.82325	5.145	C36
1.04	0.324652	0.603341	4.07	0.4477	24.2	1.49985	4.57	C36
1	0.591419	0.46333	4.07	0.4477	24.27	1.51305	4.57	C36
0.87	0.62231	0.448367	5.7	0.798	24.48	2.3282	7.7	C36
0.95	0.810445	0.363095	5.7	0.798	24.71	2.3324	7.7	C36
0.88	1.08545	0.257036	5.7	0.798	24.86	2.3464	7.7	C36
0.67	1.12325	0.244203	7.46	1.4174	24.8	4.2408	13.46	C36
0.83	1.34151	0.178248	6.365	1.08205	24.7	3.32775	10.365	C36
0.67	1.35773	0.173891	7.46	1.4174	24.8	4.2408	13.46	C36
0.83	1.51376	0.135685	6.365	1.08205	24.7	3.32775	10.365	C36
0.83	0.12482	0.720157	3.585	0.4302	25.88	1.5564	4.085	E48
0.76	0.295292	0.619889	3.585	0.4302	26	1.5474	4.085	E48
0.82	0.424246	0.548856	5.9	1.062	26.1	3.3624	9.9	E48
0.86	0.610613	0.454002	3.2	0.448	26.27	1.9341	4.2	E48
0.32	0.207422	0.670695	7.115	0.8538	23.49	1.911	8.615	C30
0.29	0.375537	0.575182	7.605	0.9126	23.64	1.962	9.105	C30
0.32	0.674064	0.4239	8.1	0.972	23.68	2.0028	9.6	C30
0.32	0.791514	0.371214	7.605	0.9126	23.9	1.9458	9.105	C30
0.31	0.811909	0.362471	7.645	0.99385	23.9	2.3452	9.645	C30
0.35	0.967503	0.29979	7.075	0.849	23.93	1.9998	8.075	C30
0.36	1.1203	0.245189	7.115	0.8538	24.14	2.061	8.615	C30
0.33	1.30845	0.187358	7.115	0.8538	24.24	2.0622	8.615	C30
0.29	-0.89709	1.44335	7.605	0.9126	23.64	1.962	9.105	C30
0.32	-0.67125	1.26865	8.1	0.972	23.68	2.0028	9.6	C30
0.32	-0.43304	1.09246	7.605	0.9126	23.9	1.9458	9.105	C30
0.31	-0.29019	0.991338	7.645	0.99385	23.9	2.3452	9.645	C30
0.35	-0.05406	0.832614	7.075	0.849	23.93	1.9998	8.075	C30
0.36	0.139241	0.711406	7.115	0.8538	24.14	2.061	8.615	C30
0.33	0.288054	0.624002	7.115	0.8538	24.24	2.0622	8.615	C30
0.99	-0.60615	1.21962	4.2	1.428	25.02	6.1608	10.2	C18
0.96	-0.39777	1.06716	4.415	1.2362	25.25	5.1338	9.915	C18
0.32	0.154017	0.70249	7.115	0.8538	23.49	1.911	8.615	C30
0.29	0.233345	0.655509	7.605	0.9126	23.64	1.962	9.105	C30
0.32	0.453668	0.533256	8.1	0.972	23.68	2.0028	9.6	C30
0.32	0.675961	0.423018	7.605	0.9126	23.9	1.9458	9.105	C30
0.31	0.883473	0.332757	7.645	0.99385	23.9	2.3452	9.645	C30

0.35	0.995482	0.289276	7.075	0.849	23.93	1.9998	8.075	C30
0.36	1.10336	0.250904	7.115	0.8538	24.14	2.061	8.615	C30
0.33	1.37176	0.17018	7.115	0.8538	24.24	2.0622	8.615	C30
0.99	-0.03302	0.819027	4.2	1.428	25.02	6.1608	10.2	C18
0.96	0.068605	0.754729	4.415	1.2362	25.25	5.0638	9.915	C18
1.49	0.262759	0.638477	2.335	0.39695	22.61	2.01195	3.335	F51
1.14	0.529527	0.494107	2.335	0.39695	22.8	2.09015	3.335	F51
0.96	0.579409	0.46922	2.335	0.39695	23.16	2.0927	3.335	F51
1.52	0.767544	0.381643	2.335	0.39695	23.52	2.08335	3.335	F51
2	1.05521	0.267606	2.75	0.4125	23.78	1.89675	3.75	F51
1.82	1.12466	0.243732	2.75	0.4125	23.75	1.944	3.75	F51
1.86	1.33026	0.181313	2.75	0.4125	23.84	2.03925	3.75	F51
0.32	0.329892	0.600411	7.115	0.8538	23.49	1.911	8.615	C30
0.29	0.445736	0.537439	7.605	0.9126	23.64	1.962	9.105	C30
0.32	0.817742	0.359993	8.1	0.972	23.68	2.0028	9.6	C30
0.32	0.778486	0.376862	7.605	0.9126	23.9	1.9458	9.105	C30
0.31	0.850724	0.346169	7.645	0.99385	23.9	2.3452	9.645	C30
0.35	0.969727	0.298946	7.075	0.849	23.93	1.9998	8.075	C30
0.36	1.13983	0.238705	7.115	0.8538	24.14	2.061	8.615	C30
0.33	1.2891	0.192834	7.115	0.8538	24.24	2.0622	8.615	C30
0.82	0.655618	0.432534	5.9	1.062	26.1	3.3624	9.9	E48
0.31	0.375185	0.575375	2.625	0.34125	23.96	1.72185	3.125	C27
0.36	0.484987	0.516905	2.335	0.39695	24.1	2.2882	3.335	C27
0.37	0.66738	0.427017	3.2	0.448	24.29	2.0013	4.2	C27
0.34	0.734188	0.396433	3.6	0.792	24.36	3.6025	6.6	C27
0.35	0.935498	0.312101	5.9	1.062	24.85	3.5406	9.9	C27
0.37	1.08197	0.25824	5.95	1.1305	24.98	3.84655	10.45	C27
0.36	-0.03679	0.821451	3	0.6	24.5	3.012	5	C27
0.39	0.451566	0.534363	5.445	1.03455	25.05	3.75915	9.445	C27
0.35	0.963477	0.301322	5.9	1.062	24.85	3.5406	9.9	C27
0.37	1.06502	0.264147	5.95	1.1305	24.98	3.80855	10.45	C27
0.39	1.33343	0.180445	5.445	1.03455	25.05	3.66415	9.445	C27
0.49	1.36848	0.171042	1	0.1	21.42	0.9125	1	C38
0.41	0.62395	0.44758	2.75	0.4125	24.27	1.79925	3.75	C32
0.34	0.812085	0.362396	2.75	0.4125	24.48	1.80525	3.75	C32
0.38	1.09342	0.254295	3.2	0.448	24.55	1.7416	4.2	C32
0.41	1.15654	0.233248	2.75	0.4125	24.68	1.8195	3.75	C32
0.4	1.36214	0.172719	2.75	0.4125	24.73	1.82925	3.75	C32
1.57	0.848055	0.347276	12.135	2.79105	23.37	7.6038	26.135	G60
1.61	0.994523	0.289633	13.7	3.288	23.72	9.1344	31.2	G60
1.47	1.17634	0.226888	12.275	3.06875	23.82	9.015	29.275	G60
1.61	0.693957	0.414698	13.7	3.288	23.72	9.0408	31.2	G60
1.47	0.869592	0.338404	12.275	3.06875	23.82	8.93375	29.275	G60
1.41	-0.67722	1.27318	1	0.1	22.13	1.0385	1	F51
1.35	-0.3077	1.00354	1	0.1	21.85	1.0455	1	F51
1.47	-0.10299	0.864583	1.5	0.15	21.81	1.056	1.5	F51
1.8	0.078818	0.748394	1.5	0.15	21.74	1.0935	1.5	F51
1.17	0.179269	0.687371	1.5	0.15	21.43	1.0915	1.5	F51
0.71	0.370769	0.577793	1.5	0.15	21.31	1.091	1.5	F51
0.48	0.525075	0.496361	1.5	0.15	21.26	1.0935	1.5	F51
0.45	0.594551	0.461801	1.5	0.15	21.32	1.101	1.5	F51

0.56	0.670624	0.425503	1.75	0.2625	21.29	1.72425	2.25	F51
0.43	0.702438	0.410809	2.25	0.5625	21.61	3.1125	3.75	F51
0.33	0.174757	0.690061	2	0.2	23.26	1.0815	2	B07
0.28	0.302084	0.616042	2	0.2	23.66	0.9025	2	B07
0.18	0.452611	0.533813	2.335	0.39695	24.09	1.99835	3.335	B07
0.2	0.616903	0.450967	2.335	0.39695	24.35	2.03915	3.335	B07
0.12	0.686719	0.418033	2.75	0.4125	24.43	1.88175	3.75	B07
0.1	0.853152	0.345164	2.875	0.5175	24.49	2.3553	4.375	B07
0.18	1.05047	0.269288	2.75	0.4125	24.53	1.8885	3.75	B07
0.27	1.27093	0.198073	2.75	0.4125	24.6	1.83675	3.75	B07
0.28	1.44662	0.151313	3.2	0.448	24.61	1.7983	4.2	B07
0.89	0.070379	0.753627	3.1	0.372	21.7	1.38	3.6	C38
0.81	0.127695	0.718409	2.625	0.34125	21.87	1.4404	3.125	C38
0.71	0.283213	0.62676	3.1	0.372	22.13	1.374	3.6	C38
0.75	0.420694	0.550754	3.1	0.372	22.2	1.3674	3.6	C38
0.89	-0.03593	0.820898	3.1	0.372	21.7	1.38	3.6	C38
0.71	0.217405	0.664828	3.1	0.372	22.13	1.374	3.6	C38
0.75	0.348555	0.590031	3.1	0.372	22.2	1.3674	3.6	C38
2.49	0.082862	0.745892	5.335	0.90695	22.53	2.62395	8.335	F51
0.85	1.13948	0.238821	3.665	0.47645	23.83	1.3013	4.665	C27
0.92	1.25709	0.202126	3.915	0.7047	24.04	2.4408	6.415	C27
0.5	0.818923	0.359493	1.5	0.15	23.87	1.1795	1.5	C26
0.6	0.977237	0.296106	1.5	0.15	24.1	1.1825	1.5	C26
3.78	-0.33559	1.02308	4.815	0.7704	22.38	2.436	7.315	F51
2.69	-0.48082	1.12708	4.875	0.8775	22.38	2.8494	7.875	F51
2.61	-0.49819	1.13975	4.875	0.8775	22.44	2.8458	7.875	F51
2.49	-0.32845	1.01807	5.335	0.90695	22.53	2.62395	8.335	F51
0.93	0.244014	0.649307	2	0.2	24.26	1.1135	2	C31
0.75	0.375164	0.575386	3	0.3	24.33	1.1885	3	C31
0.73	0.451675	0.534306	3.585	0.4302	24.4	1.491	4.085	C31
0.58	0.680299	0.421004	4.07	0.4477	24.32	1.40965	4.57	C31
0.68	0.856159	0.343921	4.355	0.74035	24.26	2.4939	6.855	C31
1.04	0.968168	0.299538	4.285	0.6856	24.35	2.2824	6.285	C31
1.1	1.06338	0.264723	3.915	0.7047	24.44	2.7567	6.415	C31
1.12	1.31913	0.184381	4	0.8	24.5	3.165	7	C31
0.73	0.595052	0.461557	3.585	0.4302	24.4	1.491	4.085	C31
0.68	0.801417	0.366954	4.355	0.74035	24.26	2.4939	6.855	C31
1.1	1.13954	0.238801	3.915	0.7047	24.44	2.5767	6.415	C31
1.12	1.33248	0.180705	4	0.8	24.5	3.165	7	C31
0.37	0.82841	0.355487	1	0.1	24.51	1.172	1	C31
0.34	0.789154	0.372233	1	0.1	24.52	1.175	1	C31
0.36	0.867722	0.339168	1	0.1	24.54	1.175	1	C31
0.48	0.980395	0.294917	1.5	0.15	24.55	1.191	1.5	C31
0.49	1.1505	0.235211	1.5	0.15	24.59	1.193	1.5	C31
0.46	1.29977	0.189801	1.5	0.15	24.6	1.1955	1.5	C31
0.79	-1.26031	1.73735	1.5	0.15	21.8	0.9385	1.5	C14
0.72	-1.04005	1.5573	1.5	0.15	22.12	0.9405	1.5	C14
0.57	-0.85852	1.41303	1.5	0.15	22.42	0.9385	1.5	C14
0.53	-0.71397	1.30116	1.5	0.15	22.57	0.9385	1.5	C14
0.58	-0.63268	1.23953	1.5	0.15	22.56	0.913	1.5	C14
0.67	-0.39447	1.06481	2	0.2	22.55	0.943	2	C14

0.74	-0.2453	0.960321	2	0.2	22.64	0.9495	2	C14
0.77	-0.01549	0.807774	2	0.2	22.81	0.9575	2	C14
0.78	0.177805	0.688244	2	0.2	22.92	0.956	2	C14
0.8	0.326618	0.602241	2	0.2	23.02	0.944	2	C14
0.67	0.592484	0.46281	2	0.2	22.55	0.943	2	C14
0.74	0.784593	0.374208	2	0.2	22.64	0.9495	2	C14
0.77	0.902043	0.325293	2	0.2	22.81	0.9575	2	C14
0.78	1.01949	0.280437	2	0.2	22.92	0.956	2	C14
0.8	1.2164	0.214367	2	0.2	23.02	0.944	2	C14
0.38	0.818945	0.359483	1	0.1	22.95	0.9545	1	C36
0.44	0.968208	0.299522	1.5	0.15	22.77	0.9475	1.5	C36
0.4	1.12101	0.244953	1	0.1	22.58	0.845	1	C36
0.78	1.10351	0.250851	7.77	1.9425	25.3	6.35375	17.77	C26
0.79	0.253976	0.643541	3	0.3	23.84	1.051	3	C26
0.75	0.385126	0.56995	3	0.3	24.16	1.0965	3	C26
0.6	0.448977	0.535728	4.215	0.5901	24.45	1.8452	5.715	C26
0.43	0.66494	0.428158	4.75	0.7125	24.59	2.172	6.75	C26
0.61	0.872452	0.337235	4.285	0.6856	24.65	2.3008	6.285	C26
0.9	0.978131	0.295769	4.285	0.6856	24.91	2.3304	6.285	C26
0.84	1.07967	0.259034	4.355	0.74035	25.07	2.5789	6.855	C26
0.78	1.29744	0.19046	7.77	1.9425	25.3	6.35375	17.77	C26
0.78	1.23412	0.208978	7.77	1.9425	25.3	6.35375	17.77	C26
0.83	0.1752	0.689797	2.625	0.34125	23.28	1.3273	3.125	C26
0.9	0.331532	0.599495	2.625	0.34125	23.58	1.34225	3.125	C26
0.79	0.466366	0.526595	3	0.3	23.84	1.051	3	C26
0.75	0.678141	0.422005	3	0.3	24.16	1.0965	3	C26
0.6	0.813051	0.361985	4.215	0.5901	24.45	1.8452	5.715	C26
0.43	0.767465	0.381678	4.75	0.7125	24.59	2.172	6.75	C26
0.61	0.839703	0.350753	4.285	0.6856	24.65	2.3008	6.285	C26
0.9	0.952376	0.305571	4.285	0.6856	24.91	2.3304	6.285	C26
0.84	1.11615	0.24658	4.355	0.74035	25.07	2.5789	6.855	C26
0.78	1.21477	0.214867	7.77	1.9425	25.3	6.35375	17.77	C26
0.78	0.533381	0.49216	7.77	1.9425	25.3	6.35375	17.77	C26
0.43	0.129331	0.717414	4.75	0.7125	24.59	2.172	6.75	C26
0.61	0.375629	0.575132	4.285	0.6856	24.65	2.3008	6.285	C26
0.9	0.535871	0.490904	4.285	0.6856	24.91	2.3304	6.285	C26
0.84	0.641443	0.439234	4.355	0.74035	25.07	2.5789	6.855	C26
0.78	0.74603	0.391146	7.77	1.9425	25.3	6.35375	17.77	C26
0.59	0.886524	0.331524	1	0.1	23.6	0.988	1	C38
0.48	0.992203	0.290496	1	0.1	23.68	0.9925	1	C38
0.51	1.10008	0.25202	1	0.1	23.84	0.9965	1	C38
0.49	1.36848	0.171042	1	0.1	23.85	1.0005	1	C38
0.75	0.86375	0.340797	6.5	1.95	22.99	7.0275	16.5	C26
0.72	1.0212	0.279817	6.6	2.112	23.04	7.8608	17.6	C26
0.71	1.18312	0.224736	7	2.1	23.13	7.5585	18	C26
0.4	0.512305	0.502858	8.365	1.42205	24.8	3.7808	13.865	C35
0.4	0.704414	0.409906	9.355	1.59035	24.83	4.0528	15.355	C35
0.34	0.809204	0.363624	10.925	2.07575	24.94	5.27915	19.425	C35
0.31	0.932985	0.31308	11.405	2.0529	24.98	5.103	19.905	C35
0.34	1.12989	0.241991	11.405	2.0529	25.03	5.1057	19.905	C35
0.47	0.664101	0.428551	7.115	0.8538	24.75	2.0448	8.615	C35

0.4	0.749899	0.389427	8.365	1.42205	24.8	3.7808	13.865	C35
0.4	0.776625	0.377672	9.355	1.59035	24.83	4.0528	15.355	C35
0.34	0.913227	0.320846	10.925	2.07575	24.94	5.27915	19.425	C35
0.31	1.07236	0.26158	11.405	2.0529	24.98	5.103	19.905	C35
0.34	1.2605	0.201123	11.405	2.0529	25.03	5.1057	19.905	C35
0.48	0.032419	0.777361	3	0.3	24.62	1.1575	3	C35
0.47	0.223575	0.661214	7.115	0.8538	24.75	2.0448	8.615	C35
0.4	0.318071	0.607032	8.365	1.42205	24.8	3.7808	13.865	C35
0.4	0.406011	0.558639	9.355	1.59035	24.83	4.0528	15.355	C35
0.34	0.515438	0.50126	10.925	2.07575	24.94	5.27915	19.425	C35
0.31	0.53082	0.493453	11.405	2.0529	24.98	5.103	19.905	C35
0.34	0.731112	0.397813	11.405	2.0529	25.03	5.1057	19.905	C35
0.48	0.413518	0.554601	3	0.3	24.62	1.1575	3	C35
0.47	0.407433	0.557873	7.115	0.8538	24.75	2.0448	8.615	C35
0.4	0.16885	0.693591	8.365	1.42205	24.8	3.7808	13.865	C35
0.4	0.431654	0.544907	9.355	1.59035	24.83	4.0528	15.355	C35
0.34	0.453891	0.533139	10.925	2.07575	24.94	5.27915	19.425	C35
0.31	0.669736	0.425917	11.405	2.0529	24.98	5.103	19.905	C35
0.34	0.958804	0.303106	11.405	2.0529	25.03	5.1057	19.905	C35
0.47	0.443705	0.538513	7.115	0.8538	24.75	2.0448	8.615	C35
0.4	0.634347	0.442609	8.365	1.42205	24.8	3.7808	13.865	C35
0.4	0.848189	0.34722	9.355	1.59035	24.83	4.0528	15.355	C35
0.34	0.941207	0.309883	10.925	2.07575	24.94	5.27915	19.425	C35
0.31	1.05541	0.267534	11.405	2.0529	24.98	5.103	19.905	C35
0.34	1.32382	0.183084	11.405	2.0529	25.03	5.1057	19.905	C35
1.66	1.20334	0.218398	4.285	0.6856	24.36	2.2488	6.285	F51
1.08	1.36284	0.172532	2.165	0.28145	22.62	1.45015	2.665	C38
0.76	0.320225	0.605823	1.5	0.15	22.03	0.916	1.5	F52
0.64	0.497071	0.510667	1.75	0.2625	22.3	1.37775	2.25	F52
0.69	0.68918	0.416898	1.75	0.2625	22.36	1.36875	2.25	F52
0.65	1.11466	0.247083	3.3	0.528	22.82	2.0096	4.8	F52
1.7	0.330258	0.600206	1	0.1	20.91	0.7515	1	B09
0.88	0.426822	0.547481	1.5	0.3	21.39	1.597	2	B09
0.89	0.56277	0.477445	1.5	0.3	21.9	1.6	2	B09
0.48	0.69016	0.416446	1.75	0.2625	22.29	1.218	2.25	B09
0.47	0.861521	0.341712	1.75	0.2625	22.15	1.21875	2.25	B09
1.66	1.31058	0.186762	4.285	0.6856	24.36	2.2488	6.285	F51
0.65	1.49419	0.140116	3.3	0.528	22.82	2.0096	4.8	F52
0.24	0.256555	0.642052	6.555	2.68755	26.24	11.4164	20.555	K70
0.17	0.428205	0.546743	9.665	4.15595	26.65	18.1482	34.665	K70
0.2	0.573794	0.471987	10.315	4.7449	26.74	21.4843	39.315	K70
0.22	0.806574	0.364747	10.94	5.3606	26.85	25.0905	43.94	K70
0.3	-0.30759	1.00346	2.5	0.5	21.82	1.996	4	C28
0.44	0.25692	0.641842	5.11	0.6132	23.57	1.7184	6.11	C26
0.77	0.41879	0.551774	5.11	0.6132	23.67	1.6926	6.11	C26
0.72	0.605014	0.456712	3.1	0.372	23.86	1.4286	3.6	C26
0.59	0.629575	0.444887	3.3	0.528	24.01	2.0048	4.8	C26
0.37	0.81771	0.360007	3.3	0.528	24.41	2.0288	4.8	C26
0.35	1.08005	0.258902	3.4	0.612	24.56	2.5173	5.4	C26
0.38	1.14951	0.235536	3.4	0.612	24.68	2.3481	5.4	C26
0.41	1.37409	0.16957	3.75	0.5625	24.67	2.0235	5.25	C26

0.59	0.000642	0.797476	1.5	0.15	23.32	1.0645	1.5	C26
0.53	0.174648	0.690126	1.5	0.15	23.57	1.065	1.5	C26
0.44	0.241315	0.650873	5.11	0.6132	23.57	1.7184	6.11	C26
0.77	0.372465	0.576863	5.11	0.6132	23.67	1.7526	6.11	C26
1.63	0.163244	0.696948	2.165	0.28145	24.73	1.53335	2.665	C32
1.9	0.319577	0.606186	2.165	0.28145	24.94	1.56455	2.665	C32
2.16	0.410099	0.556438	2.335	0.39695	25.08	2.1539	3.335	C32
2.1	0.634534	0.44252	2.75	0.4125	25.21	1.974	3.75	C32
2.15	0.801096	0.367091	3.2	0.448	25.28	1.9117	4.2	C32
2.28	0.815087	0.36112	3.915	0.7047	25.19	2.7927	6.415	C32
2.21	0.92776	0.315123	3.915	0.7047	25.3	2.7981	6.415	C32
2.14	1.09154	0.254941	4.43	0.8417	25.36	3.1426	7.43	C32
0.65	-0.41558	1.07991	1	0.1	21.32	1.0275	1	C39
0.62	-0.28834	0.990054	1	0.1	21.37	1.036	1	C39
0.57	-0.37218	1.04894	1	0.1	21.63	1.0475	1	C39
0.6	-0.18757	0.921004	1	0.1	21.75	1.07	1	C39
0.65	0.096153	0.737696	1	0.1	21.89	1.077	1	C39
2.15	0.40075	0.561477	3.2	0.448	25.28	1.9117	4.2	C32
2	0.187488	0.682483	2.75	0.4125	25.22	1.98	3.75	C32
2.28	0.431301	0.545094	3.915	0.7047	25.19	2.7927	6.415	C32
2.21	0.466199	0.526682	3.915	0.7047	25.3	2.7981	6.415	C32
2.14	0.669383	0.426082	4.43	0.8417	25.36	3.1426	7.43	C32
0.73	0.141908	0.709793	2.335	0.39695	21.85	1.92695	3.335	C30
1.06	0.323562	0.603952	2.165	0.28145	22.18	1.4599	2.665	C30
0.69	0.439405	0.54079	2.625	0.34125	22.12	1.51385	3.125	C30
0.59	0.156715	0.700867	1.5	0.15	22.51	0.791	1.5	C35
0.63	0.236043	0.653938	1.5	0.15	22.73	0.788	1.5	C35
0.68	0.367193	0.579754	1.5	0.15	22.87	0.822	1.5	C35
0.6	0.456366	0.531837	1.5	0.15	23.16	0.8295	1.5	C35
0.58	0.67866	0.421764	1.5	0.15	23.33	0.8845	1.5	C35
0.56	0.892502	0.329115	1.5	0.15	23.41	0.8365	1.5	C35
0.56	1.10605	0.249988	1.5	0.15	23.66	0.87	1.5	C35
0.54	1.35547	0.174493	2.165	0.28145	23.8	1.39295	2.665	C35
0.54	1.36882	0.170953	2.165	0.28145	23.8	1.39295	2.665	C35
0.57	1.00723	0.28493	1.75	0.2625	21.65	1.59225	2.25	C32
0.95	1.17755	0.226502	2.335	0.39695	22.85	1.921	3.335	C32
0.72	1.39102	0.165179	2.75	0.4125	22.9	1.74675	3.75	C32
0.76	1.53439	0.131124	2.75	0.4125	22.97	1.7745	3.75	C32
0.69	1.13444	0.240485	4.285	0.6856	24.11	2.3616	6.285	C18
0.75	1.29003	0.192567	4.75	0.7125	24.09	1.77375	6.75	C18
0.33	1.06643	0.263652	4.75	0.7125	21.67	1.92675	6.75	C39
0.48	1.33484	0.18006	4.75	0.7125	21.67	1.926	6.75	C39
0.75	1.04994	0.269476	4.625	0.60125	24.55	1.51775	5.625	I65
0.58	1.11306	0.247621	4.69	0.6566	24.68	1.792	6.19	I65
0.59	1.33131	0.181023	5.11	0.6132	24.77	1.6038	6.11	I65
0.75	0.935353	0.312157	4.625	0.60125	24.55	1.51775	5.625	I65
0.58	1.0369	0.274138	4.69	0.6566	24.68	1.792	6.19	I65
0.59	1.31796	0.184703	5.11	0.6132	24.77	1.6038	6.11	I65
1.27	-0.17421	0.911998	2.5	0.25	21.95	0.895	2.5	I65
1.32	0.066206	0.75622	2.5	0.25	22.16	0.8965	2.5	I65
1.19	0.320762	0.605521	2.5	0.25	22.24	0.983	2.5	I65

0.94	0.472382	0.523454	2.5	0.25	22.47	0.9575	2.5	I65
0.69	0.565784	0.475949	4.625	0.60125	22.61	1.68285	5.625	I65
0.71	0.643538	0.43824	4.565	0.50215	22.64	1.4388	5.065	I65
0.57	0.750824	0.389017	4.565	0.50215	23.04	1.47015	5.065	I65
0.45	1.19807	0.220039	4.215	0.5901	23.03	2.0447	5.715	I65
1.66	0.561019	0.478315	2.5	0.25	23.7	1.1285	2.5	C36
1.52	0.605718	0.456371	2.5	0.25	23.89	1.144	2.5	C36
1.33	0.65876	0.431056	3.5	0.35	24.01	1.245	3.5	C36
1.39	0.554626	0.481499	3.5	0.35	24.38	1.2545	3.5	C36
1.17	0.746734	0.390832	3.5	0.35	24.62	1.2635	3.5	C36
1.03	0.864185	0.340618	3.5	0.35	24.52	1.271	3.5	C36
1.08	0.981635	0.29445	3.5	0.35	24.58	1.299	3.5	C36
1.03	1.17854	0.226187	3	0.3	24.5	1.2665	3	C36
2.07	0.174265	0.690355	1.5	0.15	23.47	0.9395	1.5	C36
1.97	0.330598	0.600017	2	0.2	23.41	0.9745	2	C36
1.66	0.446441	0.537067	2.5	0.25	23.7	1.1285	2.5	C36
1.52	0.658216	0.431312	2.5	0.25	23.89	1.144	2.5	C36
1.33	0.818448	0.359694	3.5	0.35	24.01	1.245	3.5	C36
1.39	0.779191	0.376554	3.5	0.35	24.38	1.2545	3.5	C36
1.17	0.85776	0.343261	3.5	0.35	24.62	1.2635	3.5	C36
1.03	0.970433	0.298678	3.5	0.35	24.52	1.271	3.5	C36
1.08	1.14054	0.238473	3.5	0.35	24.58	1.299	3.5	C36
1.03	1.2898	0.192632	3	0.3	24.5	1.2665	3	C36
1.97	0.150671	0.704504	2	0.2	23.41	0.9745	2	C36
1.66	0.299859	0.617301	2.5	0.25	23.7	1.1285	2.5	C36
1.52	0.43734	0.541885	2.5	0.25	23.89	1.144	2.5	C36
1.33	0.617313	0.45077	3.5	0.35	24.01	1.245	3.5	C36
1.39	0.871044	0.33781	3.5	0.35	24.38	1.2545	3.5	C36
1.17	1.03091	0.276294	3.5	0.35	24.62	1.2635	3.5	C36
1.03	1.21389	0.215137	3.5	0.35	24.52	1.271	3.5	C36
1.08	1.4147	0.159169	3.5	0.35	24.58	1.299	3.5	C36
1.03	1.55807	0.126025	3	0.3	24.5	1.2665	3	C36
1.36	-0.90202	1.44723	1	0.1	21.07	0.796	1	C27
1.59	-0.61653	1.2274	1	0.1	21.21	0.796	1	C27
1.58	-0.34299	1.02829	1	0.1	21.49	0.796	1	C27
1.22	-0.12396	0.878435	4.07	0.4477	22.32	1.29195	4.57	C27
0.98	0.112498	0.727673	4.07	0.4477	22.41	1.27435	4.57	C27
0.99	0.279435	0.628916	4.07	0.4477	22.57	1.3211	4.57	C27
1.06	0.423835	0.549075	4.145	0.53885	22.65	1.64775	5.145	C27
0.35	0.103728	0.733043	4.07	0.4477	23.8	1.45915	4.57	G55
0.35	-0.057	0.834521	4.07	0.4477	23.8	1.45915	4.57	G55
0.35	0.514377	0.501801	4.07	0.4477	23.8	1.45915	4.57	G55
0.65	1.20245	0.218673	1	0.1	22.09	0.8815	1	C22
2.07	0.063571	0.757859	1.5	0.3	22.03	1.828	2	C13
3.03	0.127218	0.718699	1.5	0.3	22.1	1.843	2	C13
3.17	0.213102	0.667354	2	0.6	22.24	2.9805	3	C13
3.07	0.458199	0.530874	2.165	0.28145	22.4	1.2974	2.665	C13
2.68	0.61285	0.452921	2.335	0.39695	22.54	1.7476	3.335	C13
2.29	0.866582	0.339635	2.335	0.39695	22.53	1.8428	3.335	C13
1.52	1.07076	0.262136	1.5	0.15	23.79	0.9915	1.5	C13
1.11	1.25374	0.203115	1	0.1	23.96	0.991	1	C13

0.63	-0.2076	0.934576	5.11	0.6132	22.02	1.6308	6.11	C19
0.56	-0.10194	0.863891	5.11	0.6132	22.17	1.6308	6.11	C19
0.51	0.006528	0.793734	5.055	0.55605	22.12	1.4399	5.555	C19
0.43	0.652238	0.434126	3	0.3	22.14	1.105	3	C39
0.46	0.81247	0.362233	2.5	0.25	22.11	1.0535	2.5	C39
0.46	0.773214	0.379161	2.5	0.25	22.19	1.041	2.5	C39
0.5	0.851782	0.34573	2.5	0.25	22.35	1.044	2.5	C39
1.01	0.326613	0.602244	2.5	0.25	21.67	1.0085	2.5	C38
0.67	0.442456	0.539174	2.5	0.25	21.62	1.024	2.5	C38
0.52	0.654231	0.433187	2.5	0.25	21.37	1.0205	2.5	C38
0.38	0.814463	0.361385	2.5	0.25	21.68	1.078	2.5	C38
0.35	0.775206	0.378291	2	0.2	22.06	1.0345	2	C38
0.37	0.853775	0.344906	1	0.1	22.38	0.976	1	C38
0.26	0.947456	0.307466	2.165	0.28145	22.63	1.45925	2.665	C38
0.29	1.11756	0.246106	2.165	0.28145	22.57	1.4404	2.665	C38
0.32	1.26683	0.199269	2.165	0.28145	22.58	1.4794	2.665	C38
0.94	0.835767	0.352399	1.5	0.15	23.03	1.055	1.5	C36
1.08	1.11077	0.248393	1.5	0.15	23.2	1.057	1.5	C36
0.97	1.18022	0.225653	1.5	0.15	23.31	1.052	1.5	C36
0.85	1.38582	0.166519	1.5	0.15	23.38	1.054	1.5	C36
1.09	0.831782	0.35407	5.5	0.55	23.71	1.415	5.5	C38
1.08	1.10679	0.24974	6.5	0.65	24.06	1.518	6.5	C38
1.04	1.16991	0.228941	8.065	0.88715	24.32	1.94095	9.065	C38
0.82	1.34385	0.177613	7.785	1.2456	24.44	3.2264	11.785	C38
1.08	0.8602	0.342256	6.5	0.65	24.06	1.518	6.5	C38
1.04	0.97132	0.298342	8.065	0.88715	24.32	1.9448	9.065	C38
0.82	1.13658	0.239779	7.785	1.2456	24.44	3.2504	11.785	C38
1.08	-0.16495	0.905778	3.1	0.372	23.01	1.3308	3.6	E48
1.17	0.075463	0.750472	3.1	0.372	23.26	1.3404	3.6	E48
1.02	0.330019	0.60034	3.1	0.372	23.37	1.2684	3.6	E48
0.94	0.481639	0.518641	3.1	0.372	23.59	1.3572	3.6	E48
0.69	0.659126	0.430885	3.1	0.372	23.92	1.233	3.6	E48
0.56	0.766412	0.38214	3.1	0.372	24.03	1.4598	3.6	E48
0.55	0.924726	0.316312	3.1	0.372	24.34	1.4808	3.6	E48
0.45	0.974288	0.297219	3.585	0.4302	24.66	1.5336	4.085	E48
0.38	1.23265	0.209423	3.585	0.4302	24.81	1.5354	4.085	E48
0.93	0.979127	0.295394	3.6	0.792	22.73	2.9854	6.6	C33
0.94	0.518358	0.499773	3.6	0.792	22.85	2.9843	6.6	C33
0.75	0.605314	0.456567	4.43	0.8417	23	2.89465	7.43	C33
0.7	0.443421	0.538663	5.335	0.90695	23.07	2.73955	8.335	C33
0.57	0.666839	0.42727	4.875	0.8775	23.09	2.8278	7.875	C33
0.59	0.830905	0.354438	5.75	0.8625	23.17	2.48175	8.25	C33
0.7	0.653377	0.433589	5.335	0.90695	23.07	2.73955	8.335	C33
0.59	0.931638	0.313606	5.75	0.8625	23.17	2.48175	8.25	C33
0.69	0.974869	0.297	5.28	0.8448	23.31	2.58	7.78	C33
0.74	-0.53819	1.16914	1.5	0.15	22.81	1.03	1.5	C39
0.77	-0.42028	1.08329	1.5	0.15	23.03	1.037	1.5	C39
0.72	-0.39057	1.06202	3.5	0.35	23.25	1.18	3.5	C39
0.67	-0.3176	1.01046	5	0.5	23.39	1.322	5	C39
0.72	-0.11724	0.873988	6	0.6	23.46	1.4075	6	C39
0.78	0.050877	0.76578	6.5	0.65	23.67	1.461	6.5	C39

0.51	0.873511	0.336804	2.625	0.34125	22.85	1.34225	3.125	C35
0.55	0.99185	0.290628	4.07	0.4477	23.23	1.47235	4.57	C35
0.49	1.0744	0.260866	4.75	0.7125	23.22	2.30775	6.75	C35
0.51	1.35547	0.174493	6.665	0.86645	23.28	2.23535	8.665	C35
1.95	0.422054	0.550027	7.855	1.33535	24.13	3.72725	12.855	C26
1.58	0.646489	0.436842	6.275	0.94125	24.09	2.787	9.275	C26
1.3	0.806721	0.364684	6.225	0.93375	24.13	2.70525	8.725	C26
1.24	0.773795	0.378907	6.71	0.9394	24.42	2.5522	9.21	C26
0.9	0.846033	0.348116	6.75	1.0125	24.64	2.80575	9.75	C26
1.18	0.939715	0.310462	5.39	0.9702	24.74	3.1113	8.89	C26
1.13	1.09716	0.253015	4	0.8	24.64	2.982	7	C26
1.02	1.19578	0.220753	3.375	0.945	24.69	4.1622	6.875	C26
0.69	0.974869	0.297	5.28	0.8448	23.31	2.58	7.78	C33
0.75	1.25222	0.203567	6.135	0.79755	23.35	2.15215	7.635	C33
0.87	1.00759	0.284799	3.5	0.35	24.87	1.194	3.5	E48
0.55	1.19293	0.221647	3.5	0.35	25.05	1.2015	3.5	E48
0.57	1.45029	0.150427	3.5	0.35	25.23	1.208	3.5	E48
0.78	0.2605	0.639778	6.5	0.65	23.67	1.461	6.5	C39
0.72	0.356628	0.585569	3.5	0.35	23.25	1.18	3.5	C39
0.67	0.508247	0.504932	5	0.5	23.39	1.322	5	C39
0.72	0.620641	0.449169	6	0.6	23.46	1.4075	6	C39
0.78	0.685734	0.418488	6.5	0.65	23.67	1.461	6.5	C39
0.72	0.228073	0.658584	3.5	0.35	23.25	1.18	3.5	C39
0.67	0.359223	0.584137	5	0.5	23.39	1.322	5	C39
0.72	0.448396	0.536035	6	0.6	23.46	1.4075	6	C39
0.78	0.670689	0.425472	6.5	0.65	23.67	1.461	6.5	C39
0.8	0.884532	0.332329	1.5	0.15	23.91	1.063	1.5	C39
0.78	0.99021	0.291239	2.5	0.25	24.09	1.136	2.5	C39
0.83	1.09808	0.2527	2	0.2	24.1	1.12	2	C39
0.81	1.36649	0.171567	2.5	0.25	24.17	1.2065	2.5	C39
0.72	0.148567	0.705773	3.5	0.35	23.25	1.18	3.5	C39
0.67	0.325005	0.603144	5	0.5	23.39	1.322	5	C39
0.72	0.591772	0.463158	6	0.6	23.46	1.4075	6	C39
0.78	0.641654	0.439134	6.5	0.65	23.67	1.461	6.5	C39
1.75	-0.04367	0.825893	4.355	0.74035	23.93	2.6418	6.855	C26
2.18	0.117675	0.724511	5.445	1.03455	23.96	3.4371	9.445	C26
1.95	0.209663	0.669376	7.855	1.33535	24.13	3.72725	12.855	C26
1.58	0.319256	0.606366	6.275	0.94125	24.09	2.787	9.275	C26
1.3	0.586023	0.465971	6.225	0.93375	24.13	2.70525	8.725	C26
1.24	0.642236	0.438858	6.71	0.9394	24.42	2.5522	9.21	C26
0.9	0.82404	0.357329	6.75	1.0125	24.64	2.80575	9.75	C26
1.18	1.08005	0.258902	5.39	0.9702	24.74	3.1113	8.89	C26
1.13	1.13685	0.23969	4	0.8	24.64	2.982	7	C26
1.75	-0.15676	0.900283	4.355	0.74035	23.93	2.6418	6.855	C26
2.18	0.071001	0.75324	5.445	1.03455	23.96	3.4371	9.445	C26
1.95	0.338218	0.595769	7.855	1.33535	24.13	3.72725	12.855	C26
1.58	0.502498	0.507878	6.275	0.94125	24.09	2.787	9.275	C26
1.3	0.614892	0.451936	6.225	0.93375	24.13	2.70525	8.725	C26
1.24	0.686316	0.418219	6.71	0.9394	24.42	2.5522	9.21	C26
0.9	0.787271	0.373048	6.75	1.0125	24.64	2.80575	9.75	C26
1.18	0.926594	0.31558	5.39	0.9702	24.74	3.1113	8.89	C26

1.13	0.963495	0.301315	4	0.8	24.64	2.982	7	C26
0.55	0.504534	0.506833	4.07	0.4477	23.23	1.47235	4.57	C35
0.49	0.688727	0.417106	4.75	0.7125	23.22	2.31	6.75	C35
0.51	0.990456	0.291148	6.665	0.86645	23.28	2.30035	8.665	C35
0.66	0.988365	0.291928	2.5	0.25	22.84	1.112	2.5	C27
0.54	1.15847	0.232623	3	0.3	23.06	1.156	3	C27
0.51	1.30774	0.187557	3	0.3	23.1	1.1525	3	C27
1.16	0.742749	0.392606	3.5	0.35	23.74	1.1135	3.5	C38
1.14	0.8602	0.342256	5.5	0.55	24.01	1.3045	5.5	C38
1.15	0.97765	0.29595	6	0.6	24.19	1.4	6	C38
1.07	1.16823	0.22948	6.54	0.7194	24.42	1.65055	7.04	C38
0.66	0.986141	0.292761	2.5	0.25	22.84	1.112	2.5	C27
0.54	1.13894	0.239	3	0.3	23.06	1.162	3	C27
0.51	1.32708	0.182184	3	0.3	23.1	1.162	3	C27
0.53	0.603439	0.457476	3.125	0.71875	22.96	3.07165	5.625	C39
0.46	0.717055	0.404155	4.085	0.8987	23.47	3.4518	7.585	C39
0.41	0.900691	0.325833	4.875	0.8775	23.75	2.8917	7.875	C39
0.37	0.943923	0.308831	4.94	0.9386	23.62	3.17205	8.44	C39
0.43	1.20228	0.218727	4.94	0.9386	23.13	3.2262	8.44	C39
0.75	-0.01729	0.808925	1	0.1	21.43	0.854	1	C35
0.76	0.156715	0.700867	1	0.1	21.58	0.923	1	C35
0.75	0.236043	0.653938	1	0.1	21.48	0.924	1	C35
0.7	0.367193	0.579754	1	0.1	21.96	0.925	1	C35
0.68	0.456366	0.531837	1	0.1	22.28	0.922	1	C35
0.58	0.67866	0.421764	1	0.1	22.65	1.024	1	C35
0.49	0.892502	0.329115	1.5	0.15	22.8	1.038	1.5	C35
0.46	0.99818	0.288274	1.5	0.15	22.84	1.0615	1.5	C35
0.42	1.10605	0.249988	1.5	0.15	22.81	1.0255	1.5	C35
0.44	1.37446	0.169473	1.5	0.15	22.69	0.9485	1.5	C35
0.46	0.775818	0.378024	4.085	0.8987	23.47	3.4518	7.585	C39
0.41	0.913812	0.320614	4.875	0.8775	23.75	2.8917	7.875	C39
0.37	1.07759	0.259758	4.94	0.9386	23.62	3.07705	8.44	C39
0.43	1.22685	0.211178	4.94	0.9386	23.13	3.07705	8.44	C39
0.72	0.776604	0.377681	1.5	0.15	22.21	0.9725	1.5	I65
0.73	0.737348	0.395018	1.5	0.15	22.43	0.984	1.5	I65
0.78	0.815917	0.360768	1.5	0.15	22.51	0.9925	1.5	I65
0.87	0.928589	0.314798	1.5	0.15	22.39	1.046	1.5	I65
0.84	1.0987	0.252491	1.5	0.15	22.46	1.0545	1.5	I65
0.93	1.24796	0.204832	1.5	0.15	22.63	1.06	1.5	I65
0.71	0.966528	0.300161	8.265	1.23975	23.4	2.4795	12.265	C35
0.75	1.2732	0.197412	3	0.3	23.88	1.214	3	C32
0.94	1.14851	0.235861	2.5	0.25	23.84	1.166	2.5	C32
0.75	1.29777	0.190365	3	0.3	23.88	1.214	3	C32
0.48	0.342143	0.593587	1	0.1	23.51	1.1365	1	C38
0.32	0.49494	0.511764	1	0.1	23.57	1.1315	1	C38
0.2	0.557034	0.480298	1.5	0.15	23.5	1.1375	1.5	C38
0.33	0.601733	0.458305	1.5	0.15	23.02	1.1275	1.5	C38
0.42	0.654775	0.432931	1.5	0.15	22.87	1.119	1.5	C38
0.48	0.550641	0.483489	1.5	0.15	22.81	0.989	1.5	C38
0.46	0.742749	0.392606	1.5	0.15	22.99	0.993	1.5	C38
0.46	0.8602	0.342256	1.5	0.15	22.93	0.998	1.5	C38

0.39	0.97765	0.29595	1.5	0.15	22.71	0.983	1.5	C38
0.2	0.230066	0.657421	1.5	0.15	23.5	1.1375	1.5	C38
0.33	0.361216	0.58304	1.5	0.15	23.02	1.1275	1.5	C38
0.42	0.450388	0.534984	1.5	0.15	22.87	1.119	1.5	C38
0.48	0.672682	0.424544	1.5	0.15	22.81	0.989	1.5	C38
0.46	0.886524	0.331524	1.5	0.15	22.99	0.993	1.5	C38
0.46	0.992203	0.290496	1.5	0.15	22.93	0.998	1.5	C38
0.39	1.10008	0.25202	1.5	0.15	22.71	0.983	1.5	C38
0.5	1.36848	0.171042	1	0.1	22.63	0.912	1	C38
0.63	0.84537	0.348391	1.75	0.2625	21.7	1.52625	2.25	C33
0.63	1.00524	0.285665	1.75	0.2625	21.71	1.527	2.25	C33
0.76	1.18822	0.223126	1.75	0.2625	21.78	1.5255	2.25	C33
0.88	1.38903	0.165691	1.75	0.2625	21.79	1.5225	2.25	C33
0.93	1.5324	0.13156	1.75	0.2625	21.83	1.5315	2.25	C33
0.51	0.682126	0.420157	4	0.4	23.62	1.0455	4	C22
0.42	0.842358	0.349645	4	0.4	23.64	1.023	4	C22
0.53	0.803102	0.366232	3.5	0.35	23.64	0.981	3.5	C22
0.63	0.869009	0.338642	3.585	0.4302	23.72	1.416	4.085	C22
0.74	0.937369	0.311373	4.94	0.9386	24.02	3.1673	8.44	C22
0.77	1.09482	0.253817	8.535	1.79235	24.3	5.1513	16.535	C22
0.59	1.23775	0.207886	8.6	1.892	24.47	5.6166	17.6	C22
0.57	0.132422	0.715538	2	0.2	21.19	1.084	2	I65
0.6	0.288755	0.623603	2	0.2	21.28	1.0945	2	I65
0.54	0.360285	0.583552	2.335	0.39695	21.34	2.0366	3.335	I65
0.56	0.57206	0.472843	2.335	0.39695	21.57	2.03915	3.335	I65
0.52	0.732292	0.397284	2.335	0.39695	21.83	1.99665	3.335	I65
0.62	0.737348	0.395018	2	0.2	22.1	1.1075	2	I65
0.5	0.796925	0.368882	2.165	0.28145	22.24	1.5054	2.665	I65
0.5	0.833634	0.353293	2.25	0.5625	22.49	3.105	3.75	I65
0.53	1.03539	0.274679	3	0.6	22.55	2.732	5	I65
0.52	1.21631	0.214396	1.75	0.2625	22.85	1.716	2.25	I65
0.81	0.132422	0.715538	1	0.1	20.46	0.9765	1	I65
0.85	0.288755	0.623603	1	0.1	20.48	0.987	1	I65
0.84	0.616249	0.451282	3.3	0.528	22.91	2.0168	4.8	C38
0.74	0.782811	0.374981	3.75	0.5625	23	1.95975	5.25	C38
0.79	0.699242	0.412272	4.085	0.8987	23.37	3.3165	7.585	C38
0.59	0.796801	0.368936	4.94	0.9386	23.5	3.03145	8.44	C38
0.61	0.922135	0.317331	8.845	1.50365	23.54	3.68645	14.345	C38
0.62	1.07325	0.261267	6	1.2	23.64	3.703	11	C38
0.65	1.21618	0.214434	6.05	1.2705	23.7	4.1517	11.55	C38
0.4	0.006829	0.793542	3.1	0.372	23.61	1.3428	3.6	B11
0.39	0.041973	0.771357	3.1	0.372	23.66	1.2852	3.6	B11
0.62	0.183727	0.684718	3.1	0.372	23.74	1.3476	3.6	B11
0.82	0.360165	0.583619	3.1	0.372	24.06	1.3476	3.6	B11
0.39	0.87128	0.337714	4.565	0.50215	24.12	1.5598	5.065	B11
0.35	1.12729	0.242855	4.215	0.5901	24.07	2.0671	5.715	B11
0.37	1.20308	0.21848	4.625	0.60125	24.13	1.9136	5.625	B11
0.47	1.40867	0.160684	4.625	0.60125	24.18	1.9071	5.625	B11
0.59	0.76092	0.384554	5.22	0.7308	23.96	2.1147	7.22	C39
0.69	0.781316	0.375631	4.75	0.7125	24.12	2.20425	6.75	C39
0.79	0.924248	0.3165	5.28	0.8448	24.15	2.48	7.78	C39

0.73	1.07705	0.259946	4.815	0.7704	24.26	2.3992	7.315	C39
0.69	1.25886	0.201605	4.355	0.74035	24.24	2.61375	6.855	C39
0.52	0.676395	0.422816	4.645	1.06835	25.42	4.1377	9.145	C30
0.65	0.774854	0.378445	4.785	1.2441	25.53	4.914	10.285	C30
0.95	0.904965	0.324127	5.19	1.2456	25.73	4.656	10.69	C30
0.96	1.08921	0.25574	5.315	1.3819	25.91	5.3274	11.815	C30
0.83	1.04946	0.269646	1.5	0.3	23.24	2.164	2	C35
0.82	1.18222	0.225022	1.5	0.15	23.24	1.047	1.5	C35
0.73	1.38781	0.166005	2	0.2	23.17	1.1515	2	C35
0.39	0.83451	0.352925	4.565	0.50215	24.12	1.5598	5.065	B11
0.35	0.973834	0.297391	4.215	0.5901	24.07	2.0671	5.715	B11
0.37	1.02973	0.276723	4.625	0.60125	24.13	1.9136	5.625	B11
0.47	1.28808	0.193123	4.625	0.60125	24.18	1.9071	5.625	B11
0.49	-0.03907	0.822923	3.7	0.888	25.38	3.864	7.2	C30
0.52	0.087067	0.743295	4.645	1.06835	25.42	4.1377	9.145	C30
0.65	0.238547	0.652482	4.785	1.2441	25.53	4.914	10.285	C30
0.95	0.418144	0.552119	5.19	1.2456	25.73	4.656	10.69	C30
0.96	0.566529	0.47558	5.315	1.3819	25.91	5.3274	11.815	C30
0.52	0.787878	0.372785	4.645	1.06835	25.42	4.1377	9.145	C30
0.65	0.93818	0.311057	4.785	1.2441	25.53	4.914	10.285	C30
0.95	1.13618	0.239909	5.19	1.2456	25.73	4.656	10.69	C30
0.96	1.38088	0.167799	5.315	1.3819	25.91	5.3274	11.815	C30
0.62	-0.05279	0.831789	3	0.9	25.35	4.44	6	C30
0.49	0.189807	0.681107	3.7	0.888	25.38	3.864	7.2	C30
0.52	0.432271	0.544578	4.645	1.06835	25.42	4.1377	9.145	C30
0.65	0.59759	0.46032	4.785	1.2441	25.53	4.914	10.285	C30
0.95	0.774831	0.378455	5.19	1.2456	25.73	4.656	10.69	C30
0.96	1.01394	0.282466	5.315	1.3819	25.91	5.3274	11.815	C30
0.95	0.191162	0.680303	5.19	1.2456	25.73	4.656	10.69	C30
0.96	0.335563	0.597248	5.315	1.3819	25.91	5.3274	11.815	C30
0.67	-0.03856	0.822593	1.5	0.3	25.15	2.18	2	C30
0.53	0.103196	0.73337	1.5	0.3	25.2	2.347	2	C30
0.61	0.311286	0.610848	1.75	0.2625	25.26	1.79175	2.25	C30
0.62	0.483097	0.517884	3	0.9	25.35	4.44	6	C30
0.49	0.570962	0.473386	3.7	0.888	25.38	3.864	7.2	C30
0.52	0.765427	0.382572	4.645	1.06835	25.42	4.1377	9.145	C30
0.65	1.02144	0.279728	4.785	1.2441	25.53	4.914	10.285	C30
0.95	1.10355	0.250837	5.19	1.2456	25.73	4.656	10.69	C30
0.96	1.29649	0.19073	5.315	1.3819	25.91	5.3274	11.815	C30
1.26	0.509474	0.504305	2.165	0.28145	23.59	1.3676	2.665	C14
0.35	0.788955	0.372319	2.165	0.28145	27.32	1.67115	2.665	K70
0.36	0.888967	0.330538	3.75	0.5625	27.47	2.30325	5.25	K70
0.36	1.05907	0.26624	2.75	0.4125	27.67	2.1195	3.75	K70
0.38	1.20201	0.218812	3.3	0.528	27.77	2.4168	4.8	K70
0.79	0.561019	0.478315	1.5	0.15	22.71	0.998	1.5	C36
0.76	0.605718	0.456371	1.5	0.15	22.75	1.0155	1.5	C36
0.61	0.65876	0.431056	1.5	0.15	22.95	0.9965	1.5	C36
0.48	0.554626	0.481499	1.5	0.15	23.03	1.0265	1.5	C36
0.44	0.746734	0.390832	1.5	0.15	23.15	1.0375	1.5	C36
0.66	0.864185	0.340618	1.5	0.15	22.51	1.0295	1.5	C36
0.84	0.662201	0.429442	1.5	0.15	22.82	1.014	1.5	C34

0.81	0.822433	0.358008	1.5	0.15	22.88	1.017	1.5	C34
0.61	0.783176	0.374822	1.5	0.15	23.08	0.9985	1.5	C34
0.82	0.974418	0.29717	1.5	0.15	23.34	0.974	1.5	C34
0.83	1.14452	0.237165	2	0.2	23.39	1.004	2	C34
0.86	1.29379	0.191496	2.5	0.25	23.36	1.04	2.5	C34
0.84	0.369186	0.57866	1.5	0.15	22.82	1.014	1.5	C34
0.81	0.458359	0.530791	1.5	0.15	22.88	1.017	1.5	C34
0.61	0.680652	0.42084	1.5	0.15	23.08	0.9985	1.5	C34
0.82	1.00017	0.287536	1.5	0.15	23.34	0.974	1.5	C34
0.83	1.10805	0.249314	2	0.2	23.39	1.004	2	C34
0.86	1.37645	0.168952	2.5	0.25	23.36	1.04	2.5	C34
1.06	-0.1251	0.879196	3.1	0.372	22.86	1.3626	3.6	C26
1.25	0.115314	0.725952	3.1	0.372	22.91	1.3476	3.6	C26
1.23	0.357209	0.585248	3.2	0.448	23.1	1.6562	4.2	C26
1.29	0.521489	0.498181	3.1	0.372	23.15	1.356	3.6	C26
1.14	0.633883	0.44283	3.1	0.372	23.39	1.2636	3.6	C26
1.72	0.692646	0.415301	2.165	0.28145	24.21	1.57365	2.665	C26
1.19	0.806262	0.36488	3.1	0.372	24.3	1.545	3.6	C26
1.17	0.958246	0.30332	2.165	0.28145	24.38	1.58405	2.665	C26
1.04	0.976156	0.296514	3.4	0.612	24.55	2.6271	5.4	C26
0.79	1.24085	0.206956	3.835	0.65195	24.61	2.5619	5.835	C26
1.06	-0.01509	0.807515	3.1	0.372	22.86	1.3626	3.6	C26
1.25	0.020055	0.785161	3.1	0.372	22.91	1.3476	3.6	C26
1.23	0.149148	0.705422	3.2	0.448	23.1	1.6562	4.2	C26
1.29	0.338247	0.595753	3.1	0.372	23.15	1.356	3.6	C26
1.14	0.605014	0.456712	3.1	0.372	23.39	1.2636	3.6	C26
1.19	0.843031	0.349365	3.1	0.372	24.3	1.545	3.6	C26
1.06	-0.26326	0.972684	3.1	0.372	22.86	1.3626	3.6	C26
1.25	-0.06097	0.8371	3.1	0.372	22.91	1.3476	3.6	C26
1.23	-0.02765	0.815569	3.2	0.448	23.1	1.6562	4.2	C26
1.29	0.073661	0.75159	3.1	0.372	23.15	1.356	3.6	C26
1.14	0.156203	0.701175	3.1	0.372	23.39	1.2636	3.6	C26
1.19	0.34001	0.594772	3.1	0.372	24.3	1.545	3.6	C26
0.79	0.709338	0.40766	3.835	0.65195	24.61	2.5619	5.835	C26
1.37	0.278413	0.6295	7.5	2.25	23.11	8.0535	19.5	C17
1.48	0.43887	0.541074	7.625	2.51625	23.13	9.3687	21.125	C17
1.34	0.639441	0.440185	7.54	2.3374	23.09	8.5002	20.04	C17
1.37	0.235143	0.654462	7.5	2.25	23.11	8.0535	19.5	C17
1.48	0.383089	0.57106	7.625	2.51625	23.13	9.3687	21.125	C17
1.34	0.556795	0.480417	7.54	2.3374	23.09	8.5002	20.04	C17
1.37	0.224101	0.660906	7.5	2.25	23.11	8.0535	19.5	C17
1.48	0.427543	0.547096	7.625	2.51625	23.13	9.3687	21.125	C17
1.34	0.509346	0.50437	7.54	2.3374	23.09	8.5002	20.04	C17
1.47	0.587232	0.465379	2.75	0.4125	22.16	1.43175	3.75	M74
1	0.801074	0.367101	2.75	0.4125	22.3	1.5645	3.75	M74
0.86	0.906752	0.323416	2.75	0.4125	22.73	1.76325	3.75	M74
0.75	1.02729	0.277605	4.625	0.60125	22.86	1.7667	5.625	M74
1.37	0.476826	0.52114	7.5	2.25	23.11	8.0535	19.5	C17
1.48	0.628306	0.445494	7.625	2.51625	23.13	9.3687	21.125	C17
1.34	0.855078	0.344368	7.54	2.3374	23.09	8.5002	20.04	C17
0.75	0.930099	0.314207	4.625	0.60125	22.86	1.7667	5.625	M74

0.76	1.18846	0.223051	4.625	0.60125	23.05	1.79335	5.625	M74
0.75	1.06377	0.264588	4.625	0.60125	22.86	1.7667	5.625	M74
0.76	1.21303	0.215403	4.625	0.60125	23.05	1.79335	5.625	M74
0.76	1.29569	0.190955	4.625	0.60125	23.05	1.79335	5.625	M74
0.25	1.07135	0.261932	3	0.9	23.74	4.551	6	D45
0.25	1.20885	0.216692	3.5	1.4	23.78	6.718	8	D45
0.26	1.39653	0.163765	3.165	1.04445	23.83	5.22885	6.665	D45
0.13	-0.08134	0.850375	2.335	0.39695	22.64	1.8972	3.335	K70
0.15	0.060418	0.759823	2.335	0.39695	22.69	1.8972	3.335	K70
0.21	0.236856	0.653465	2.335	0.39695	22.81	1.8972	3.335	K70
0.35	-0.58801	1.20607	1	0.1	20.46	0.7805	1	M73
0.29	-0.4701	1.11928	1	0.1	20.56	0.7645	1	M73
0.27	-0.44038	1.09776	1	0.1	20.93	0.8675	1	M73
0.26	-0.36741	1.04555	1	0.1	21.12	0.86	1	M73
0.24	1.08498	0.257199	1.75	0.2625	24.16	1.71675	2.25	F51
0.26	1.23424	0.208942	1.75	0.2625	24.27	1.743	2.25	F51
1.26	0.097719	0.736733	2.875	0.5175	23.05	2.4687	4.375	F52
1.46	0.241391	0.650829	3	0.6	23.23	2.846	5	F52
1.72	0.369895	0.578271	2.875	0.5175	23.14	2.484	4.375	F52
1.79	0.58167	0.468108	2.875	0.5175	23.34	2.4831	4.375	F52
1.38	0.741902	0.392984	2.875	0.5175	23.35	2.4939	4.375	F52
1.12	0.689985	0.416526	3	0.6	23.57	2.874	5	F52
0.77	0.749562	0.389576	3.125	0.71875	23.87	3.4132	5.625	F52
0.71	0.817922	0.359917	3	0.9	23.91	4.509	6	F52
0.59	0.988029	0.292054	3.5	1.05	25.17	4.998	7.5	F52
0.44	1.19427	0.221227	6.05	1.2705	25.32	4.347	11.55	F52
0.85	0.447577	0.536467	4	0.8	23.23	2.841	7	C30
0.81	0.528662	0.494545	3.835	0.65195	23.3	2.26695	5.835	C30
0.83	0.57336	0.472201	3.835	0.65195	23.41	2.28565	5.835	C30
0.65	0.484286	0.517268	4.165	0.95795	23.94	3.4339	8.165	C30
0.61	0.676395	0.422816	4.165	0.95795	24.12	3.5236	8.165	C30
0.61	0.793845	0.370208	4.165	0.95795	24.18	3.5167	8.165	C30
0.61	0.879644	0.334309	4.415	1.2362	24.26	4.809	9.915	C30
0.58	1.10187	0.251409	5.19	1.2456	24.38	4.3236	10.69	C30
0.74	-0.18408	0.918646	2	0.2	23.55	1.0345	2	E48
0.9	-0.04071	0.823978	2	0.2	23.61	1.053	2	E48
0.89	0.18164	0.685959	2	0.2	23.72	1.066	2	E48
0.7	1.06881	0.262819	1.5	0.15	23.7	0.99	1.5	S90
0.64	1.21807	0.213855	1	0.1	24	0.9775	1	S90
0.72	0.172273	0.691545	1	0.1	20.98	0.899	1	C37
0.83	0.328605	0.60113	1	0.1	20.88	0.9805	1	C37
0.66	0.444449	0.53812	1	0.1	20.94	0.9815	1	C37
0.58	0.656224	0.432249	1	0.1	20.88	0.9855	1	C37
0.51	0.816455	0.360539	1	0.1	20.92	1.002	1	C37
0.32	0.777199	0.377422	1	0.1	21.24	0.9985	1	C37
0.36	0.855767	0.344083	1	0.1	21.29	0.9975	1	C37
0.78	0.96844	0.299434	1	0.1	21.36	1.001	1	C37
0.41	1.13855	0.239129	1.5	0.15	21.29	0.9625	1.5	C37
0.54	1.28781	0.193202	1	0.1	21.33	0.9305	1	C37
0.82	-0.06739	0.841278	4.5	1.8	23.92	7.654	12	F52
0.6	0.0748	0.750883	5	2	23.82	8.418	14	F52

0.41	0.369748	0.578352	4.25	1.0625	23.86	4.13625	8.75	F52
1.23	0.639769	0.440029	2.165	0.28145	23.64	1.37345	2.665	C36
1.22	0.535634	0.491023	2.165	0.28145	23.96	1.4118	2.665	C36
0.86	0.727743	0.399328	2.165	0.28145	23.95	1.41245	2.665	C36
0.7	0.845194	0.348465	2.165	0.28145	23.95	1.41115	2.665	C36
0.71	0.962644	0.30164	2.165	0.28145	23.97	1.4079	2.665	C36
0.69	1.15955	0.232274	2.625	0.34125	23.98	1.45535	3.125	C36
1.07	-0.1808	0.916434	6.09	0.7308	25.79	2.0334	7.09	C38
1.09	-0.05642	0.834144	6.225	0.93375	25.85	2.772	8.725	C38
1.11	0.178589	0.687776	2.625	0.34125	25.96	1.7784	3.125	C38
0.91	-0.02544	0.814151	4.165	0.95795	23.16	3.38905	8.165	C36
0.76	0.044023	0.770072	10.53	3.2643	24.34	10.957	29.03	C36
0.73	0.23821	0.652678	11.025	3.41775	24.29	11.4654	30.525	C36
1.18	0.392336	0.566033	16.735	5.85725	24.81	20.7795	53.735	C36
1.06	0.567586	0.475057	17.34	6.4158	24.76	24.2628	58.84	C36
0.34	0.856838	0.343641	2.835	0.76545	23.95	3.43845	5.335	C39
0.29	1.00795	0.284664	3	0.9	24.15	3.981	6	C39
0.24	1.20153	0.21896	3.125	0.71875	24.11	3.01645	5.625	C39
2.02	0.038387	0.773609	10.585	3.3872	24.43	12.0368	30.085	F51
2.57	0.352092	0.588074	6.5	1.95	24.33	7.191	16.5	F51
1.89	0.504751	0.506722	7.225	2.52875	24.44	9.80525	20.725	F51
1.95	0.688323	0.417293	8.115	2.5968	24.48	9.6928	22.615	F51
2.02	0.940093	0.310315	10.585	3.3872	24.43	12.0368	30.085	F51
2.57	0.707242	0.408615	6.5	1.95	24.33	7.191	16.5	F51
1.89	0.788262	0.372619	7.225	2.52875	24.44	9.80525	20.725	F51
1.95	0.977361	0.296059	8.115	2.5968	24.48	9.6928	22.615	F51
2.02	1.12662	0.243078	10.585	3.3872	24.43	12.0368	30.085	F51
0.7	0.130813	0.716515	1.5	0.15	21.88	1.079	1.5	F51
0.44	0.210141	0.669095	2	0.2	21.85	1.109	2	F51
0.39	0.341291	0.594061	2.5	0.25	21.75	1.1415	2.5	F51
0.43	0.430463	0.54554	2.5	0.25	21.68	1.125	2.5	F51
0.39	0.652757	0.433882	2.5	0.25	21.37	1.0815	2.5	F51
2.02	1.22264	0.212459	10.585	3.3872	24.43	12.0368	30.085	F51
2.02	1.39489	0.164185	10.585	3.3872	24.43	12.0368	30.085	F51
0.74	0.104523	0.732556	4.565	0.50215	22.65	1.4938	5.065	I65
0.77	0.206602	0.671178	5.6	0.672	22.79	1.8042	6.6	I65
0.83	0.278037	0.629715	6.135	0.79755	22.86	2.0397	7.635	I65
0.83	0.344308	0.592385	6.625	0.86125	22.96	2.04815	8.125	I65
0.82	0.340399	0.594556	6.625	0.86125	22.99	2.04815	8.125	I65
0.71	0.382442	0.571412	6.665	0.86645	23.22	2.11315	8.665	I65
0.64	0.359457	0.584009	4.565	0.50215	23.19	1.41845	5.065	I65
0.57	0.628076	0.445604	4.565	0.50215	23.28	1.48885	5.065	I65
0.54	0.725507	0.400335	5.055	0.55605	23.33	1.49985	5.555	I65
0.59	0.933297	0.312958	5.055	0.55605	23.4	1.57685	5.555	I65
0.77	-0.75689	1.33408	5.6	0.672	22.79	1.8042	6.6	I65
0.83	-0.59628	1.21225	6.135	0.79755	22.86	2.08455	7.635	I65
0.83	-0.46116	1.11279	6.625	0.86125	22.96	2.14955	8.125	I65
0.82	-0.30011	0.998244	6.625	0.86125	22.99	2.14955	8.125	I65
0.71	-0.18031	0.916102	6.665	0.86645	23.22	2.21455	8.665	I65
1.48	-0.0821	0.850874	3.25	0.8125	20.61	3.2625	6.25	C19
1.48	0.086017	0.743943	3.25	0.8125	20.63	3.2525	6.25	C19

1.44	-0.02747	0.815453	3.25	0.8125	20.63	3.25125	6.25	C19
1.35	0.11133	0.728387	3.6	0.792	20.74	2.9425	6.6	C19
1.27	0.294462	0.62036	3.5	0.7	20.82	2.577	6	C19
1.15	0.622444	0.448303	4	0.8	20.98	2.854	7	C19
1.2	1.10712	0.249626	3.5	0.7	20.87	2.679	6	C19
1.35	0.514225	0.501879	1	0.1	24.72	1.188	1	C36
1.26	0.626619	0.446301	1	0.1	24.87	1.1925	1	C36
1.28	0.691712	0.415731	1	0.1	25.09	1.1955	1	C36
1.33	0.798998	0.367992	1.5	0.15	25.4	1.222	1.5	C36
1.21	0.957312	0.303677	1.5	0.15	25.54	1.2275	1.5	C36
1.22	1.00687	0.285061	2	0.2	25.62	1.2625	2	C36
1.05	1.26523	0.199735	1.5	0.15	25.66	1.2335	1.5	C36
1.24	1.13456	0.240444	2	0.2	23.55	1.1395	2	C39
1.01	1.24584	0.205463	7.785	1.2456	24.9	2.9536	11.785	C39
2.62	0.814018	0.361574	5.75	2.0125	25.53	8.3195	15.75	F51
2.67	0.947213	0.30756	6.055	1.87705	25.58	7.34545	15.555	F51
2.55	1.16498	0.230525	5.94	2.3166	25.57	9.8436	17.44	F51
0.15	1.00615	0.285327	3.6	0.792	23.52	2.9667	6.6	B07
1.32	1.00887	0.284328	1.5	0.15	22.73	1.0225	1.5	C35
1.31	1.26723	0.199152	1.5	0.15	22.79	1.028	1.5	C35
0.46	0.062295	0.758654	1	0.1	20.81	0.999	1	C26
0.49	0.228053	0.658596	1	0.1	20.92	1.0185	1	C26
0.35	0.396168	0.563956	1	0.1	21.01	1.0125	1	C26
0.39	0.531292	0.493215	1	0.1	20.86	1.0085	1	C26
0.35	0.694694	0.414359	1	0.1	20.82	0.954	1	C26
0.43	0.812145	0.362371	1	0.1	20.79	0.9485	1	C26
0.42	0.838871	0.351101	1	0.1	20.75	0.942	1	C26
0.45	0.988134	0.292015	1	0.1	20.77	0.964	1	C26
0.44	1.14093	0.238344	1	0.1	20.67	0.829	1	C26
0.41	1.32908	0.181637	1	0.1	20.59	0.8205	1	C26
0.25	0.990358	0.291184	1	0.1	20.07	0.9925	1	C26
0.26	1.16047	0.231979	1	0.1	20.21	0.983	1	C26
0.89	0.790057	0.371843	5.95	1.1305	24.32	3.4713	10.45	C30
0.51	-0.01375	0.806658	2.5	0.5	23.96	2.449	4	C30
0.5	0.106058	0.731615	2.5	0.5	24.03	2.449	4	C30
0.71	0.308181	0.612598	2.75	0.4125	24.14	1.83675	3.75	C30
0.85	0.443466	0.538639	3.5	0.7	24.22	2.849	6	C30
0.89	0.610842	0.453891	5.95	1.1305	24.32	3.56155	10.45	C30
0.49	0.634919	0.442337	1.5	0.15	23.45	1.0585	1.5	I64
0.46	0.752369	0.388332	1.5	0.15	23.65	1.065	1.5	I64
0.45	0.779095	0.376597	2	0.2	23.74	1.095	2	I64
0.37	0.928358	0.314889	2	0.2	23.87	1.089	2	I64
0.3	0.859592	0.342506	4.25	1.9125	23.85	8.40375	11.25	I64
0.31	1.1237	0.244052	4.665	1.53945	23.48	6.4482	11.665	I64
0.55	-0.16382	0.905016	1.75	0.2625	23.87	1.64625	2.25	C30
0.64	-0.00513	0.801153	1.75	0.2625	23.97	1.64625	2.25	C30
0.51	0.235074	0.654503	2.5	0.5	23.96	2.549	4	C30
0.5	0.394941	0.56462	2.5	0.5	24.03	2.549	4	C30
0.71	0.562542	0.477558	2.75	0.4125	24.14	1.87275	3.75	C30
0.85	0.626628	0.446297	3.5	0.7	24.22	2.845	6	C30
0.89	0.804319	0.365711	5.95	1.1305	24.32	3.5663	10.45	C30

0.44	0.154569	0.702158	1.75	0.2625	22.59	1.0815	2.25	C30
0.37	0.310901	0.611064	1.75	0.2625	22.6	1.593	2.25	C30
0.35	0.458396	0.530771	1.5	0.15	22.59	1.005	1.5	C30
0.19	0.794011	0.370137	2.5	0.75	25.24	4.0125	4.5	K70
0.19	0.964119	0.301078	2.5	0.75	25.41	3.879	4.5	K70
0.17	1.11338	0.247512	2.5	0.75	25.57	3.903	4.5	K70
0.93	0.17825	0.687978	1.5	0.15	23.44	1.1155	1.5	C34
0.81	0.425105	0.548397	4.69	0.6566	23.97	2.128	6.19	C34
0.75	0.630549	0.444422	4.75	0.7125	24.02	2.3595	6.75	C34
0.73	0.78445	0.37427	4.285	0.6856	24.12	2.448	6.285	C34
0.64	0.751525	0.388706	4.75	0.7125	24.12	2.358	6.75	C34
0.09	0.415606	0.55348	8.69	3.8236	27.08	16.8938	30.69	J67
0.05	0.616787	0.451023	9.8	4.508	27.05	20.5114	36.8	J67
0.05	0.754117	0.387558	9.07	3.7187	27	16.0699	31.07	J67
0.04	0.855661	0.344127	9.105	3.8241	27.08	16.5375	31.605	J67
0.06	1.21269	0.215507	7.415	2.0762	27.18	7.5376	18.415	J67
0.05	0.584038	0.466945	9.8	4.508	27.05	20.5114	36.8	J67
0.05	0.728362	0.399049	9.07	3.7187	27	16.0699	31.07	J67
0.04	0.892139	0.329261	9.105	3.8241	27.08	16.5375	31.605	J67
0.06	1.13003	0.241946	7.415	2.0762	27.18	7.5376	18.415	J67
1.07	-1.11716	1.61975	4.5	0.45	24.67	1.3075	4.5	C39
0.98	-1.06104	1.57424	4.5	0.45	24.72	1.3185	4.5	C39
1.03	-0.90126	1.44663	4	0.4	24.8	1.2825	4	C39
1.11	-0.48794	1.13227	4	0.4	24.74	1.2945	4	C39
1.16	-0.30643	1.00265	4	0.4	24.82	1.332	4	C39
1.23	-0.20178	0.930624	8.235	1.23525	24.9	3.0795	11.735	C39
1.21	-0.05841	0.835437	8.265	1.23975	24.99	3.1485	12.265	C39
1.21	0.170266	0.692744	9.205	1.2887	25.03	3.003	12.705	C39
1.02	0.34015	0.594695	4	0.4	24.52	1.262	4	C39
1.07	0.492948	0.512791	4.5	0.45	24.67	1.3075	4.5	C39
0.98	0.555042	0.481292	4.5	0.45	24.72	1.3185	4.5	C39
1.03	0.59974	0.459273	4	0.4	24.8	1.2825	4	C39
0.99	0.652783	0.433869	3.5	0.35	24.82	1.244	3.5	C39
1.11	0.548648	0.484486	4	0.4	24.74	1.2945	4	C39
1.16	0.740757	0.393495	4	0.4	24.82	1.332	4	C39
1.23	0.826555	0.356268	8.235	1.23525	24.9	3.0795	11.735	C39
1.21	0.944006	0.308799	8.265	1.23975	24.99	3.1485	12.265	C39
1.21	1.14724	0.236275	9.205	1.2887	25.03	3.003	12.705	C39
1.23	0.930579	0.31402	8.235	1.23525	24.9	3.0795	11.735	C39
1.21	1.08338	0.257752	8.265	1.23975	24.99	3.1485	12.265	C39
1.21	1.27785	0.196065	9.205	1.2887	25.03	3.003	12.705	C39
1.02	-0.39485	1.06508	4	0.4	24.52	1.262	4	C39
1.07	-0.27967	0.984037	4.5	0.45	24.67	1.3075	4.5	C39
0.98	-0.41225	1.07752	4.5	0.45	24.72	1.3185	4.5	C39
1.03	-0.42961	1.08999	4	0.4	24.8	1.2825	4	C39
0.99	-0.26621	0.974722	3.5	0.35	24.82	1.244	3.5	C39
1.11	-0.16879	0.908351	4	0.4	24.74	1.2945	4	C39
1.16	0.040631	0.772199	4	0.4	24.82	1.332	4	C39
1.23	0.279206	0.629047	8.235	1.23525	24.9	3.0795	11.735	C39
1.21	0.50164	0.508318	8.265	1.23975	24.99	3.1485	12.265	C39
1.21	0.577112	0.47035	9.205	1.2887	25.03	3.003	12.705	C39

0.85	1.18423	0.224383	1.75	0.2625	22.03	1.6305	2.25	C35
0.81	1.38504	0.16672	1.75	0.2625	22.14	1.623	2.25	C35
0.74	1.54107	0.12967	6.625	0.86125	22.22	2.0007	8.125	C35
1.23	0.958558	0.3032	8.235	1.23525	24.9	3.0795	11.735	C39
1.21	1.06643	0.263652	8.265	1.23975	24.99	3.1485	12.265	C39
1.21	1.34117	0.178339	9.205	1.2887	25.03	3.003	12.705	C39
1.14	0.132422	0.715538	1	0.1	21.64	0.9045	1	I65
1.17	0.288755	0.623603	1	0.1	21.76	0.8735	1	I65
1.2	0.404598	0.5594	1	0.1	21.89	0.889	1	I65
0.98	0.616373	0.451222	1	0.1	22.13	0.9065	1	I65
0.88	0.776604	0.377681	1	0.1	22.37	0.8795	1	I65
0.68	0.737348	0.395018	1	0.1	22.58	0.947	1	I65
0.6	0.815917	0.360768	1	0.1	22.65	0.9455	1	I65
0.62	0.928589	0.314798	1	0.1	22.88	0.9465	1	I65
0.6	1.0987	0.252491	1	0.1	22.97	0.9555	1	I65
0.69	1.24796	0.204832	1	0.1	23.05	1.0055	1	I65
1.02	-0.02833	0.816007	4	0.4	24.52	1.262	4	C39
1.07	0.006813	0.793553	4.5	0.45	24.67	1.3075	4.5	C39
0.98	0.148567	0.705773	4.5	0.45	24.72	1.3185	4.5	C39
1.03	0.325005	0.603144	4	0.4	24.8	1.2825	4	C39
0.99	0.591772	0.463158	3.5	0.35	24.82	1.244	3.5	C39
1.11	0.641654	0.439134	4	0.4	24.74	1.2945	4	C39
1.16	0.829789	0.354907	4	0.4	24.82	1.332	4	C39
1.23	1.07314	0.261305	8.235	1.23525	24.9	3.0795	11.735	C39
1.21	1.14259	0.237798	8.265	1.23975	24.99	3.1485	12.265	C39
1.21	1.35452	0.174746	9.205	1.2887	25.03	3.003	12.705	C39
1.21	1.52677	0.132795	9.205	1.2887	25.03	3.003	12.705	C39
0.74	1.08414	0.257489	3	0.3	23.46	1.056	3	E48
0.63	1.33988	0.178688	3.1	0.372	23.71	1.4118	3.6	E48
1.13	0.479	0.52001	1	0.1	22.49	0.991	1	E48
1.05	0.541094	0.488275	1	0.1	22.53	0.991	1	E48
0.83	0.585793	0.466084	1.5	0.15	22.67	1.0065	1.5	E48
0.74	0.706261	0.409063	3	0.3	23.46	1.056	3	E48
0.63	0.788718	0.372421	3.1	0.372	23.71	1.4118	3.6	E48
0.2	0.173692	0.690697	2	0.6	22.06	3.54	3	J67
0.23	0.263186	0.638232	2.5	1	22.16	4.252	4	J67
0.21	0.325279	0.60299	2.5	1	22.37	4.346	4	J67
0.21	0.369978	0.578226	2.5	1	22.38	4.212	4	J67
0.19	0.486324	0.516213	2	0.6	22.74	2.9895	3	J67
0.48	-0.0153	0.807649	1	0.1	25.14	1.03	1	C34
0.52	0.158708	0.69967	1	0.1	25.14	1.047	1	C34
0.47	0.238036	0.652779	1	0.1	25.08	1.06	1	C34
0.54	0.369186	0.57866	1	0.1	25.08	1.075	1	C34
0.48	0.458359	0.530791	1	0.1	25.17	1.085	1	C34
0.42	0.680652	0.42084	2	0.2	25.18	1.1345	2	C34
0.39	0.894494	0.328315	2	0.2	25.16	1.1495	2	C34
0.37	1.00017	0.287536	2	0.2	25.09	1.148	2	C34
0.35	1.10805	0.249314	2.5	0.25	25.24	1.1875	2.5	C34
0.35	1.37645	0.168952	2.5	0.25	25.22	1.194	2.5	C34
0.42	0.651617	0.434419	2	0.2	25.18	1.1345	2	C34
0.39	0.839752	0.350732	2	0.2	25.16	1.1495	2	C34

0.37	1.11476	0.24705	2	0.2	25.09	1.148	2	C34
0.35	1.18421	0.224391	2.5	0.25	25.24	1.1875	2.5	C34
0.35	1.3898	0.165491	2.5	0.25	25.22	1.194	2.5	C34
0.35	1.21684	0.214234	2.5	0.25	25.24	1.1875	2.5	C34
0.35	1.4742	0.144747	2.5	0.25	25.22	1.194	2.5	C34
0.34	0.609016	0.454774	1	0.1	22.3	0.961	1	R85
0.26	0.726466	0.399903	1	0.1	22.23	0.9695	1	R85
0.19	1.04238	0.272171	3.665	1.57595	26.14	7.5035	8.665	D44
1.52	0.672829	0.424475	3.1	0.372	24.4	1.3806	3.6	C14
1.53	0.860964	0.341941	3.585	0.4302	24.39	1.5852	4.085	C14
1.53	1.13597	0.239979	3.585	0.4302	24.62	1.59	4.085	C14
1.63	1.20542	0.217752	3.585	0.4302	24.59	1.5948	4.085	C14
1.66	1.3667	0.171511	5.95	1.1305	24.83	3.61855	10.45	C14
0.6	0.964455	0.30095	1	0.1	23.04	1.0705	1	C39
0.47	1.13456	0.240444	1	0.1	23.07	1.085	1	C39
0.42	1.28383	0.194344	1	0.1	23.18	1.0905	1	C39
0.5	0.542148	0.487745	2.335	0.39695	21.39	1.5929	3.335	C15
0.61	0.730059	0.398286	2.335	0.39695	21.58	1.5929	3.335	C15
1.04	0.214126	0.666753	1	0.1	23.78	0.9755	1	E48
0.92	0.345276	0.591848	2.5	0.25	23.89	1.0285	2.5	E48
0.93	0.434448	0.543421	2.5	0.25	23.95	0.973	2.5	E48
1.99	0.63485	0.44237	1.5	0.15	22.32	0.91	1.5	F51
1.19	0.547829	0.484896	2.5	0.75	24.15	3.6885	4.5	F51
1.34	0.356957	0.585387	3.375	0.945	24.03	4.2812	6.875	F51
1.35	0.549655	0.483982	3.125	0.71875	23.95	3.28785	5.625	F51
0.05	0.840085	0.350593	4.165	0.95795	26.38	4.01925	8.165	J67
0.04	0.999213	0.287891	4.085	0.8987	26.44	3.7455	7.585	J67
0.05	1.18736	0.223397	4.085	0.8987	26.67	3.7521	7.585	J67
0.43	0.012481	0.789956	1	0.1	21.84	1.065	1	G55
0.43	0.17824	0.687984	1	0.1	22.03	1.0675	1	G55
0.38	0.346355	0.59125	1	0.1	22.26	1.078	1	G55
0.28	0.481479	0.518723	1	0.1	22.57	1.061	1	G55
0.24	0.644881	0.437604	1	0.1	22.59	1.062	1	G55
0.19	0.762332	0.383933	1	0.1	22.63	1.0625	1	G55
0.17	0.789057	0.372275	1	0.1	22.65	1.065	1	G55
0.18	0.93832	0.311003	1	0.1	22.72	1.0795	1	G55
0.16	1.09112	0.255085	1	0.1	22.93	1.0665	1	G55
0.15	1.27926	0.195658	1	0.1	22.97	1.067	1	G55
0.78	0.853775	0.344906	3	0.3	23.68	1.133	3	C38
0.62	0.934796	0.312375	4.75	0.7125	24.22	2.2485	6.75	C38
0.6	1.13022	0.241881	4.565	0.50215	24.4	1.50315	5.065	C38
0.66	1.27949	0.195593	4.565	0.50215	24.81	1.5246	5.065	C38
0.78	0.886524	0.331524	3	0.3	23.68	1.133	3	C38
0.62	0.960551	0.302439	4.75	0.7125	24.22	2.2485	6.75	C38
0.6	1.09375	0.254183	4.565	0.50215	24.4	1.44815	5.065	C38
0.66	1.36215	0.172715	4.565	0.50215	24.81	1.4663	5.065	C38
0.77	0.786567	0.373352	2	0.2	23.14	1.021	2	G56
0.59	0.747311	0.390576	2	0.2	23.5	1.0135	2	G56
0.47	0.825879	0.356553	2	0.2	23.7	0.8945	2	G56
0.54	0.938552	0.310913	2	0.2	23.75	0.9375	2	G56
0.56	1.10866	0.249106	2	0.2	24	0.9585	2	G56

0.61	0.672003	0.42486	1.75	0.2625	21.9	1.37325	2.25	F51
0.56	0.925006	0.316203	1.75	0.2625	21.86	1.356	2.25	F51
0.53	0.70034	0.411769	2	0.2	23.37	1.0335	2	C35
0.4	0.937158	0.311455	2.165	0.28145	23.3	1.40335	2.665	C35
0.46	1.15777	0.23285	2	0.2	23.18	1.025	2	C35
1.18	0.301146	0.616572	3.1	0.372	22.7	1.4628	3.6	C29
1.03	0.438627	0.541202	3.585	0.4302	22.84	1.5216	4.085	C29
0.9	1.40333	0.162036	4.69	0.6566	23.45	1.9726	6.19	C29
0.91	1.5467	0.128456	5.22	0.7308	23.61	2.1476	7.22	C29
1.18	0.363892	0.581568	3.1	0.372	22.7	1.4628	3.6	C29
1.03	0.515512	0.501223	3.585	0.4302	22.84	1.5228	4.085	C29
0.71	1.10108	0.251678	2.5	0.25	24.62	1.13	2.5	E48
0.59	1.28923	0.192797	2.5	0.25	24.75	1.154	2.5	E48
0.71	0.800516	0.36734	2.5	0.25	24.62	1.13	2.5	E48
0.59	0.98248	0.294133	2.5	0.25	24.75	1.154	2.5	E48
0.56	0.028959	0.779541	1.75	0.2625	26.72	1.82775	2.25	B06
0.58	0.183374	0.684928	2.165	0.28145	26.85	1.6471	2.665	B06
0.58	0.359812	0.583813	2.165	0.28145	26.96	1.65035	2.665	B06
0.47	0.626579	0.44632	2.165	0.28145	27.01	1.6393	2.665	B06
0.33	0.676462	0.422785	2.165	0.28145	27.05	1.6406	2.665	B06
0.32	0.864596	0.340449	2.165	0.28145	27.08	1.64125	2.665	B06
0.43	1.09529	0.253655	2.5	0.5	27.07	2.787	4	B06
0.45	1.18373	0.224542	2.335	0.39695	27.11	2.28905	3.335	B06
0.42	1.41465	0.159182	2.165	0.28145	27.06	1.77645	2.665	B06
0.08	0.211749	0.668149	1	0.1	22.26	0.941	1	J67
0.09	0.396435	0.563811	1	0.1	23.38	0.9085	1	J67
0.06	0.584506	0.466715	2	0.4	24.25	2.046	3	J67
0.03	0.749086	0.389788	2	0.4	24.42	2.135	3	J67
0.02	0.937371	0.311372	2.335	0.39695	24.57	2.01535	3.335	J67
0.01	1.42438	0.156756	1	0.1	24.15	1.046	1	J67
0.97	-0.04228	0.824993	1.5	0.15	24.26	1.083	1.5	E48
1.07	-0.00714	0.802432	1.5	0.15	24.54	1.0865	1.5	E48
1.01	0.134619	0.714205	1.5	0.15	24.8	1.096	1.5	E48
0.93	0.311057	0.610977	1.5	0.15	24.99	1.096	1.5	E48
0.89	0.577824	0.47	1.5	0.15	25.12	1.011	1.5	E48
0.77	0.627707	0.445781	1.5	0.15	25.13	1.0115	1.5	E48
0.64	0.815841	0.3608	2	0.2	25.15	1.048	2	E48
0.68	1.05919	0.266198	1.75	0.2625	25.19	1.65375	2.25	E48
0.88	1.12865	0.242405	2.75	0.4125	25.3	1.89825	3.75	E48
0.97	1.33424	0.180223	2.75	0.4125	25.48	1.935	3.75	E48
0.66	-0.07857	0.848567	1.5	0.15	23.25	0.896	1.5	A01
0.89	0.161848	0.697785	1.5	0.15	23.13	0.995	1.5	A01
1.05	0.416404	0.553052	2	0.2	23.13	1.043	2	A01
1.01	0.568023	0.47484	2	0.2	23.21	1.0515	2	A01
0.92	0.680417	0.420949	1.5	0.15	23.23	1.0025	1.5	A01
0.67	0.728445	0.399012	2.625	0.34125	23.28	1.48265	3.125	A01
0.67	0.893025	0.328905	2.625	0.34125	23.34	1.5665	3.125	A01
0.8	1.06232	0.265097	2.625	0.34125	23.32	1.5665	3.125	A01
0.48	1.235	0.208714	2.75	0.4125	23.43	1.881	3.75	A01
0.87	1.47337	0.144942	5.9	1.062	23.55	3.2661	9.9	A01
0.97	1.23823	0.207742	2.75	0.4125	25.48	1.935	3.75	E48

0.87	1.29296	0.191733	5.9	1.062	23.55	3.2661	9.9	A01
0.87	1.37562	0.169169	5.9	1.062	23.55	3.2661	9.9	A01
0.87	1.56123	0.125357	5.9	1.062	23.55	3.2661	9.9	A01
0.62	0.615708	0.451543	1.445	0.27455	22.44	1.8924	1.445	C38
0.56	0.829551	0.355007	1.465	0.27835	22.43	1.92755	1.465	C38
0.57	0.935229	0.312206	1.46	0.2774	22.43	1.91425	1.46	C38
0.63	1.0431	0.271913	1.935	0.36765	22.45	1.9627	1.935	C38
0.64	1.31784	0.184738	1.915	0.3447	22.49	1.8279	1.915	C38
0.97	0.778793	0.376728	2.45	0.4655	23.71	2.0862	2.45	C36
0.98	1.0538	0.268106	2.45	0.4655	23.89	2.1755	2.9	C36
0.78	1.12325	0.244203	2.95	0.5605	24	2.3769	3.95	C36
0.62	1.32884	0.1817	2.945	0.55955	24.12	2.3674	3.945	C36
0.53	-1.35912	1.81972	1.475	0.28025	24	1.94655	1.475	C38
0.4	-1.13887	1.63745	1.47	0.2793	24.1	1.93705	1.47	C38
0.43	-0.96367	1.49612	1.485	0.297	24.1	2.075	1.485	C38
0.42	-0.81912	1.38226	1.99	0.398	24.2	2.183	1.99	C38
0.44	-0.73783	1.31943	1.99	0.398	24.15	2.176	1.99	C38
0.42	-0.49962	1.1408	1.99	0.398	24.21	2.176	1.99	C38
0.42	-0.34411	1.02908	1.925	0.36575	24.27	1.9513	1.925	C38
0.42	-0.10798	0.867873	1.925	0.3465	24.24	1.8441	1.925	C38
0.42	0.263693	0.63794	1.99	0.398	24.2	2.183	1.99	C38
0.44	0.530461	0.493635	1.99	0.398	24.15	2.176	1.99	C38
0.42	0.580343	0.46876	1.99	0.398	24.21	2.176	1.99	C38
0.42	0.774808	0.378465	1.925	0.36575	24.27	1.9513	1.925	C38
0.42	1.05614	0.267275	1.925	0.3465	24.24	1.8441	1.925	C38
0.06	1.34132	0.178297	4.835	1.78895	27.05	7.73855	12.835	J67
0.19	1.32721	0.182151	2.92	0.5256	23.58	2.0124	2.92	C40
1.77	1.22052	0.213106	4	0.8	23.81	2.6	4	C39
1.55	0.389483	0.567581	1.305	0.2088	23.46	1.3664	1.305	C28
1.72	0.396059	0.564015	1.305	0.2088	23.34	1.3664	1.305	C28
1.51	0.189128	0.68151	1.305	0.2088	23.44	1.3664	1.305	C28
0.71	0.77556	0.378137	2.2	0.528	25.51	3.1164	2.2	C36
0.44	1.08992	0.255497	2.21	0.5304	25.65	3.3828	3.21	C36
0.03	0.498129	0.510122	3.1	0.372	30.51	1.4292	3.6	J66
0.02	0.807689	0.364271	3.1	0.372	30.68	1.4178	3.6	J66
0.02	0.925139	0.31615	3.1	0.372	30.75	1.59	3.6	J66
0.02	1.12205	0.244605	3.1	0.372	30.85	1.6206	3.6	J66
0.62	0.270428	0.634072	2.5	0.5	22.88	2.524	4	C17
0.48	0.407909	0.557616	2.5	0.5	22.91	2.524	4	C17
0.48	0.982489	0.29413	2.665	0.61295	23.17	3.036	4.665	C17
0.51	1.16547	0.230365	2.665	0.61295	23.18	3.1326	4.665	C17
0.47	1.36628	0.171623	2.665	0.61295	23.33	3.13605	4.665	C17
0.44	1.50965	0.136607	2.665	0.61295	23.38	3.14295	4.665	C17
0.29	-0.22377	0.945584	1.455	0.27645	23.4	1.9114	1.455	C38
0.25	-0.01435	0.807042	1.96	0.3724	23.41	2.01495	1.96	C38
0.26	0.255877	0.642443	1.96	0.3724	23.49	2.014	1.96	C38
0.29	0.478311	0.520369	1.955	0.37145	23.62	2.0083	1.955	C38
0.37	0.547453	0.485085	1.955	0.37145	23.75	2.0045	1.955	C38
0.25	0.829551	0.355007	1.96	0.3724	23.41	2.01495	1.96	C38
0.26	0.935229	0.312206	1.96	0.3724	23.49	2.014	1.96	C38
0.29	1.0431	0.271913	1.955	0.37145	23.62	2.0083	1.955	C38

0.37	1.31151	0.186501	1.955	0.37145	23.75	2.0045	1.955	C38
0.92	0.134619	0.714205	2.5	0.25	27.03	1.27	2.5	E48
0.03	0.283301	0.62671	8.165	1.06145	30.23	2.71115	10.665	J66
0.03	0.436099	0.542544	8.135	1.05755	30.37	2.6325	10.135	J66
0.03	0.510853	0.5036	7.57	0.8327	30.5	2.06745	8.57	J66
0.03	0.555552	0.481037	9.06	0.9966	30.57	2.2132	10.06	J66
0.03	0.608594	0.454978	8.565	0.94215	30.66	2.1626	9.565	J66
0.03	0.498129	0.510122	9.09	1.0908	30.73	2.4648	10.59	J66
0.02	0.690238	0.41641	9.585	1.1502	30.81	2.5344	11.085	J66
0.02	0.807689	0.364271	9.585	1.1502	30.89	2.5386	11.085	J66
0.02	0.925139	0.31615	9.585	1.1502	30.95	2.532	11.085	J66
0.02	1.12205	0.244605	10.08	1.2096	31.04	2.5956	11.58	J66
0.03	0.066102	0.756284	8.165	1.06145	30.23	2.71115	10.665	J66
0.03	0.193429	0.67896	8.135	1.05755	30.37	2.6325	10.135	J66
0.03	0.294084	0.620575	7.57	0.8327	30.5	2.06745	8.57	J66
0.03	0.40093	0.56138	9.06	0.9966	30.57	2.2132	10.06	J66
0.03	0.565221	0.476229	8.565	0.94215	30.66	2.1626	9.565	J66
0.03	0.616046	0.45138	9.09	1.0908	30.73	2.4648	10.59	J66
0.02	0.80147	0.366931	9.585	1.1502	30.81	2.5344	11.085	J66
0.02	0.979794	0.295143	9.585	1.1502	30.89	2.5386	11.085	J66
0.02	1.20026	0.219357	9.585	1.1502	30.95	2.532	11.085	J66
0.02	1.36961	0.170744	10.08	1.2096	31.04	2.5956	11.58	J66
0.03	-0.1952	0.92616	8.165	1.06145	30.23	2.71115	10.665	J66
0.03	0.045222	0.76932	8.135	1.05755	30.37	2.6325	10.135	J66
0.03	0.312439	0.610198	7.57	0.8327	30.5	2.06745	8.57	J66
0.03	0.464059	0.527802	9.06	0.9966	30.57	2.2132	10.06	J66
0.03	0.576453	0.470676	8.565	0.94215	30.66	2.1626	9.565	J66
0.03	0.635215	0.442195	9.09	1.0908	30.73	2.4648	10.59	J66
0.02	0.742501	0.392717	9.585	1.1502	30.81	2.5344	11.085	J66
0.02	0.900816	0.325783	9.585	1.1502	30.89	2.5386	11.085	J66
0.02	0.950378	0.30634	9.585	1.1502	30.95	2.532	11.085	J66
0.02	1.20874	0.216726	10.08	1.2096	31.04	2.5956	11.58	J66
0.03	0.154674	0.702095	9.09	1.0908	30.73	2.4648	10.59	J66
0.02	0.417477	0.552477	9.585	1.1502	30.81	2.5344	11.085	J66
0.02	0.452376	0.533937	9.585	1.1502	30.89	2.5386	11.085	J66
0.02	0.66189	0.429587	9.585	1.1502	30.95	2.532	11.085	J66
0.02	0.950958	0.306116	10.08	1.2096	31.04	2.5956	11.58	J66
0.03	-0.08518	0.852891	8.165	1.06145	30.23	2.71115	10.665	J66
0.03	-0.05004	0.830009	8.135	1.05755	30.37	2.6325	10.135	J66
0.03	0.104378	0.732645	7.57	0.8327	30.5	2.06745	8.57	J66
0.03	0.280816	0.628127	9.06	0.9966	30.57	2.2132	10.06	J66
0.03	0.547584	0.485019	8.565	0.94215	30.66	2.1626	9.565	J66
0.03	0.591136	0.463469	9.09	1.0908	30.73	2.4648	10.59	J66
0.02	0.77927	0.37652	9.585	1.1502	30.81	2.5344	11.085	J66
0.02	1.05428	0.267937	9.585	1.1502	30.89	2.5386	11.085	J66
0.02	1.12373	0.244043	9.585	1.1502	30.95	2.532	11.085	J66
0.02	1.32932	0.181569	10.08	1.2096	31.04	2.5956	11.58	J66
0.91	1.02082	0.279953	2	0.2	22.21	0.84	2	C29
0.58	1.27918	0.195682	2	0.2	22.66	1.0855	2	C29
0.27	0.726314	0.399971	4.355	0.74035	21.9	2.4803	6.855	J69
0.21	0.687058	0.417877	3.835	0.65195	21.79	2.06465	5.835	J69

0.5	0.759296	0.38527	3.915	0.7047	21.22	2.2311	6.415	J69
0.35	0.871969	0.337433	3.915	0.7047	21.42	2.6055	6.415	J69
0.51	0.764493	0.382983	1.995	0.399	23.18	2.194	1.995	C40
0.44	1.0395	0.273204	2.005	0.401	23.27	2.213	2.005	C40
0.43	1.10262	0.251155	2.055	0.43155	23.38	2.4234	2.055	C40
0.48	1.30821	0.187423	2.06	0.4326	23.33	2.44125	2.06	C40
1.25	1.08768	0.256269	4.145	0.53885	24.84	1.9318	5.145	G58
1.26	1.24327	0.206231	5.11	0.6132	24.85	1.9056	6.11	G58
1.25	1.06814	0.263052	4.145	0.53885	24.84	1.9318	5.145	G58
1.26	1.26262	0.200501	5.11	0.6132	24.85	1.9056	6.11	G58
1.26	0.155032	0.701879	5.11	0.6132	24.85	1.9056	6.11	G58
0.2	-0.01105	0.804933	3.405	0.6129	23.76	2.0799	3.405	C35
0.18	0.102426	0.733843	3.93	0.7467	23.71	2.3408	3.93	C35
0.23	0.272897	0.632657	3.93	0.7467	23.71	2.3408	3.93	C35
0.26	0.439834	0.540563	4.44	0.8436	23.7	2.4491	4.44	C35
0.26	0.600879	0.45872	4.475	0.85025	23.76	2.5156	4.475	C35
0.2	0.217827	0.664581	3.405	0.6129	23.76	2.0799	3.405	C35
0.18	0.447629	0.536439	3.93	0.7467	23.71	2.3408	3.93	C35
0.23	0.63194	0.443757	3.93	0.7467	23.71	2.3408	3.93	C35
0.26	0.796521	0.369056	4.44	0.8436	23.7	2.4491	4.44	C35
0.26	1.04829	0.270062	4.475	0.85025	23.76	2.5156	4.475	C35
0.18	0.835528	0.352499	3.93	0.7467	23.71	2.3408	3.93	C35
0.23	0.941207	0.309883	3.93	0.7467	23.71	2.3408	3.93	C35
0.26	1.04908	0.269781	4.44	0.8436	23.7	2.4491	4.44	C35
0.26	1.31749	0.184836	4.475	0.85025	23.76	2.5156	4.475	C35
0.58	0.168979	0.693513	5.11	0.6132	25.55	1.7688	6.11	E48
0.26	0.899175	0.326439	4.44	0.8436	23.7	2.4491	4.44	C35
0.26	1.1008	0.251775	4.475	0.85025	23.76	2.5156	4.475	C35
0.58	0.788718	0.372421	5.11	0.6132	25.55	1.7688	6.11	E48
0.58	1.07069	0.262163	5.11	0.6132	25.55	1.7688	6.11	E48
0.33	1.08273	0.257977	4.69	0.6566	23.83	2.0118	6.19	C34
0.37	1.36379	0.17228	3.585	0.4302	24.27	1.5042	4.085	C34
0.58	1.23265	0.209423	5.11	0.6132	25.55	1.7688	6.11	E48
0.58	1.35323	0.175091	5.11	0.6132	25.55	1.7688	6.11	E48
0.61	1.18764	0.223309	4.07	0.4477	26.75	1.59335	4.57	E48
0.67	1.38211	0.167479	3.1	0.372	26.81	1.6644	3.6	E48
0.75	1.52549	0.133079	3.1	0.372	26.85	1.692	3.6	E48
0.61	-0.19041	0.922922	4.07	0.4477	26.75	1.59335	4.57	E48
1.02	0.479934	0.519525	2.165	0.28145	24.22	1.36305	2.665	C36
1.1	0.561019	0.478315	2	0.2	24.47	0.9725	2	C36
1.15	0.605718	0.456371	2	0.2	24.69	0.9845	2	C36
1.06	0.595456	0.46136	3	0.6	24.84	2.462	5	C36
1.1	0.503982	0.507116	3.4	0.612	25	2.169	5.4	C36
0.82	1.13423	0.240554	5.85	0.9945	25.45	3.1076	9.35	C36
0.69	0.84135	0.350066	4.665	1.53945	28.25	7.1148	11.665	E48
0.72	1.04907	0.269786	5.57	2.2837	28.34	10.6784	16.57	E48
0.83	0.448282	0.536095	1.405	0.2529	23.06	1.7208	1.405	C36
0.87	0.497715	0.510335	1.495	0.299	23.29	2.088	1.495	C36
0.63	0.542414	0.487612	1.99	0.398	23.26	2.182	1.99	C36
0.49	0.595456	0.46136	1.99	0.398	23.22	2.183	1.99	C36
0.37	0.491322	0.51363	2.995	0.599	23.21	2.386	2.995	C36

0.3	0.683431	0.419553	2.995	0.599	23.19	2.388	2.995	C36
0.48	0.800881	0.367183	3	0.6	23.68	2.6	4	C36
1.1	0.918332	0.318829	8.995	1.799	24.04	4.069	11.395	C36
0.89	1.11524	0.246887	8.985	1.797	24.11	4.209	12.185	C36
0.67	0.083295	0.745625	3.25	0.8125	25.31	3.81375	6.25	C34
0.66	0.29027	0.622741	3.835	0.65195	25.39	2.5619	5.835	C34
0.68	0.399783	0.562	6.875	1.2375	25.5	3.6495	11.375	C34
0.64	0.63688	0.441403	3.2	0.448	25.59	1.9236	4.2	C34
0.66	0.114395	0.726513	3.835	0.65195	25.39	2.5619	5.835	C34
0.68	0.187393	0.68254	6.875	1.2375	25.5	3.6495	11.375	C34
0.42	0.968521	0.299404	2.75	0.4125	26.02	2.07825	3.75	C34
0.48	1.08906	0.255794	2.625	0.34125	26.11	1.7511	3.125	C34
0.66	0.110344	0.72899	3.835	0.65195	25.39	2.5619	5.835	C34
0.68	0.253201	0.643988	6.875	1.2375	25.5	3.6495	11.375	C34
0.85	0.409533	0.556743	2	0.2	25.52	1.186	2	C37
0.87	0.397118	0.563442	2.165	0.28145	25.74	1.59835	2.665	C37
0.89	0.310673	0.611193	2.5	0.25	26.61	1.259	2.5	E48
0.75	0.426516	0.547644	3.5	0.35	26.8	1.341	3.5	E48
0.7	0.638291	0.440732	3	0.3	26.97	1.319	3	E48
0.64	0.798522	0.368196	3	0.3	27.17	1.321	3	E48
0.6	0.733945	0.396542	3.2	0.448	27.32	2.0069	4.2	E48
0.56	0.818843	0.359527	2.625	0.34125	27.41	1.77385	3.125	E48
0.56	0.937846	0.311187	3.585	0.4302	27.47	1.7616	4.085	E48
0.54	1.10162	0.251493	5.65	0.7345	27.59	2.28475	7.15	E48
0.53	1.24456	0.205847	5.22	0.7308	27.74	2.4836	7.22	E48
0.89	-0.00714	0.802432	2.5	0.25	26.61	1.259	2.5	E48
0.75	0.134619	0.714205	3.5	0.35	26.8	1.341	3.5	E48
0.7	0.311057	0.610977	3	0.3	26.97	1.319	3	E48
0.64	0.577824	0.47	3	0.3	27.17	1.321	3	E48
0.6	0.602385	0.457988	3.2	0.448	27.32	2.0069	4.2	E48
0.56	1.07819	0.25955	3.585	0.4302	27.47	1.7616	4.085	E48
0.54	1.14131	0.238221	5.65	0.7345	27.59	2.28475	7.15	E48
0.53	1.34057	0.178501	5.22	0.7308	27.74	2.4836	7.22	E48
0.28	-0.03748	0.821899	4.94	0.9386	24.87	3.12645	8.44	B11
0.29	-0.00867	0.803413	5	1	24.9	3.395	9	B11
0.32	0.126753	0.718981	5.065	1.06365	25.04	3.68235	9.565	B11
0.36	0.347504	0.590613	5.22	0.7308	25.1	2.1826	7.22	B11
0.4	0.614271	0.452235	5.7	0.798	25.19	2.2491	7.7	B11
0.26	0.664154	0.428527	5.7	0.798	25.26	2.2428	7.7	B11
0.17	0.858619	0.342907	5.65	0.7345	25.11	2.00915	7.15	B11
0.23	1.1083	0.249227	7.345	1.24865	25.03	3.3677	11.845	B11
0.29	1.17142	0.228456	6.875	1.2375	25.04	3.4965	11.375	B11
0.41	1.39601	0.163899	6.75	1.0125	25.06	2.70825	9.75	B11
0.28	-0.03748	0.821899	4.94	0.9386	24.87	3.12645	8.44	B11
0.29	-0.00867	0.803413	5	1	24.9	3.395	9	B11
0.32	0.126753	0.718981	5.065	1.06365	25.04	3.68235	9.565	B11
1.02	1.10461	0.250478	3.06	0.6426	23.28	2.98305	4.66	C39
0.58	1.31021	0.186865	4.555	0.95655	23.35	3.39465	6.655	C39
1.02	0.931262	0.313753	3.06	0.6426	23.28	2.98305	4.66	C39
0.58	1.18962	0.222685	4.555	0.95655	23.35	3.49965	6.655	C39
1.14	0.431637	0.544916	2.995	0.599	22.85	2.387	2.995	C38

1.16	0.49373	0.512387	2.99	0.598	23.02	2.384	2.99	C38
1.14	0.538429	0.489616	3.005	0.601	23.12	2.411	3.005	C38
0.84	0.731709	0.397545	2.01	0.402	24.29	2.222	2.01	C38
0.58	1.18528	0.224052	1.59	0.3498	24.74	2.5058	1.59	C38
0.88	1.0272	0.277638	8	0.8	23.24	1.4945	8	E50
0.86	0.566427	0.47563	9.5	0.95	23.44	1.769	9.5	E50
0.9	0.558427	0.479605	10.085	2.2187	23.71	5.6199	20.585	E50
0.76	0.352222	0.588002	7.335	1.98045	23.83	6.00615	17.335	E50
1.58	0.432247	0.544591	7.715	1.0801	23.29	2.5543	10.715	L72
1.43	0.595649	0.461266	7.715	1.0801	23.41	2.5522	10.715	L72
1.38	0.7131	0.405949	8.2	1.148	23.48	2.6285	11.2	L72
1.34	0.739825	0.393911	8.72	1.2208	23.57	2.7685	12.22	L72
0.64	0.592285	0.462907	6.25	2.8125	23.96	11.5065	20.25	E50
0.62	0.841968	0.349808	6.44	3.1556	23.91	13.502	21.94	E50
0.67	1.0094	0.284133	6.785	4.4781	24.08	20.493	26.285	E50
1.58	-0.09615	0.860086	7.715	1.0801	23.29	2.5543	10.715	L72
1.43	0.071965	0.752642	7.715	1.0801	23.41	2.5522	10.715	L72
1.38	-0.04152	0.824504	8.2	1.148	23.48	2.6285	11.2	L72
1.34	0.078287	0.748723	8.72	1.2208	23.57	2.7685	12.22	L72
1.46	0.223437	0.661295	2.875	0.5175	23.76	2.2626	4.375	L72
1.59	0.095145	0.738316	5.7	0.798	22.59	2.1448	7.7	L72
1.92	0.360991	0.583164	6.75	1.0125	22.91	2.57775	9.75	L72
1.58	0.579096	0.469374	7.715	1.0801	23.29	2.5543	10.715	L72
1.43	0.739328	0.394133	7.715	1.0801	23.41	2.5522	10.715	L72
1.38	0.700072	0.411892	8.2	1.148	23.48	2.6285	11.2	L72
1.34	0.77864	0.376794	8.72	1.2208	23.57	2.7685	12.22	L72
1.38	0.13205	0.715763	8.2	1.148	23.48	2.6285	11.2	L72
1.34	0.394854	0.564667	8.72	1.2208	23.57	2.7685	12.22	L72
0.82	0.326659	0.602218	3.5	0.7	24.37	3.01	6	B09
0.87	0.479457	0.519773	4	0.8	24.68	3.21	7	B09
0.9	0.739927	0.393866	3.4	0.612	25.21	2.7162	5.4	B09
1.04	0.889029	0.330513	5.165	0.67145	25.22	2.1086	6.665	B09
1.03	1.00648	0.285207	7.155	0.93015	25.45	2.41215	9.155	B09
1.13	1.14641	0.236546	3.6	0.792	25.54	3.5255	6.6	B09
0.87	-0.06817	0.841783	4	0.8	24.68	3.21	7	B09
1.03	0.964879	0.300788	7.155	0.93015	25.45	2.41215	9.155	B09
0.82	-0.67278	1.26981	3.5	0.7	24.37	3.01	6	B09
0.87	-0.30326	1.00044	4	0.8	24.68	3.21	7	B09
1.03	0.813788	0.361672	7.155	0.93015	25.45	2.41215	9.155	B09
1.13	1.02269	0.279273	3.6	0.792	25.54	3.5255	6.6	B09
0.82	-0.49746	1.13922	3.5	0.7	24.37	3.01	6	B09
0.87	-0.33053	1.01953	4	0.8	24.68	3.21	7	B09
1.04	0.607083	0.45571	5.165	0.67145	25.22	2.1086	6.665	B09
1.03	0.702821	0.410634	7.155	0.93015	25.45	2.41215	9.155	B09
0.67	-1.40402	1.85744	9.095	2.0009	25.73	6.1193	18.595	G55
0.55	-1.19009	1.6794	9.125	2.09875	25.78	6.5182	19.125	G55
0.55	-1.00856	1.532	9.125	2.09875	25.83	6.5251	19.125	G55
0.43	-0.85135	1.40742	8.565	1.79865	25.81	5.51145	17.065	G55
0.43	-0.7574	1.33448	12.455	2.36645	25.73	6.08095	22.955	G55
0.39	-0.53185	1.16446	11.05	2.3205	25.72	6.5583	22.05	G55
0.52	-0.37002	1.0474	10.95	2.0805	25.51	5.5803	19.95	G55

0.72	-0.13388	0.885025	11.38	2.0484	25.62	5.2137	19.38	G55
0.67	0.059415	0.760448	10.42	1.8756	26.15	5.0526	18.42	G55
0.43	-0.79392	1.36269	12.455	2.36645	25.73	6.08095	22.955	G55
0.67	-0.06348	0.838734	9.095	2.0009	25.73	6.1193	18.595	G55
0.55	0.095945	0.737824	9.125	2.09875	25.78	6.5182	19.125	G55
0.55	0.26406	0.637729	9.125	2.09875	25.83	6.5251	19.125	G55
0.43	0.411845	0.555499	8.565	1.79865	25.81	5.51145	17.065	G55
0.43	0.587908	0.465048	12.455	2.36645	25.73	6.08095	22.955	G55
0.39	0.692697	0.415277	11.05	2.3205	25.72	6.5583	22.05	G55
0.52	0.732084	0.397377	10.95	2.0805	25.51	5.5803	19.95	G55
0.72	0.887677	0.331058	11.38	2.0484	25.62	5.2137	19.38	G55
0.67	1.04048	0.272854	10.42	1.8756	26.15	5.0526	18.42	G55
0.61	1.23495	0.208728	7.855	1.33535	26.29	3.86835	12.855	G55
0.43	-0.03753	0.821931	12.455	2.36645	25.73	6.08095	22.955	G55
0.67	0.068413	0.754848	9.095	2.0009	25.73	6.1193	18.595	G55
0.55	0.218415	0.664236	9.125	2.09875	25.78	6.5182	19.125	G55
0.55	0.334258	0.597975	9.125	2.09875	25.83	6.5251	19.125	G55
0.43	0.558694	0.479472	8.565	1.79865	25.81	5.51145	17.065	G55
0.43	0.731586	0.3976	12.455	2.36645	25.73	6.08095	22.955	G55
0.39	0.679669	0.421296	11.05	2.3205	25.72	6.5583	22.05	G55
0.52	0.770898	0.380174	10.95	2.0805	25.51	5.5803	19.95	G55
0.72	0.889901	0.330162	11.38	2.0484	25.62	5.2137	19.38	G55
0.67	1.06001	0.26591	10.42	1.8756	26.15	5.0526	18.42	G55
0.61	1.2156	0.214612	7.855	1.33535	26.29	3.86835	12.855	G55
0.43	0.558694	0.479472	8.565	1.79865	25.81	5.51145	17.065	G55
0.43	0.731586	0.3976	12.455	2.36645	25.73	6.08095	22.955	G55
0.39	0.679669	0.421296	11.05	2.3205	25.72	6.5583	22.05	G55
0.52	0.770898	0.380174	10.95	2.0805	25.51	5.5803	19.95	G55
0.72	0.889901	0.330162	11.38	2.0484	25.62	5.2137	19.38	G55
0.43	0.147381	0.706487	12.455	2.36645	25.73	6.08095	22.955	G55
0.72	0.489888	0.51437	11.38	2.0484	25.62	5.2137	19.38	G55
0.67	0.49894	0.509705	10.42	1.8756	26.15	5.0526	18.42	G55
0.43	0.530451	0.49364	12.455	2.36645	25.73	6.08095	22.955	G55
0.39	0.771522	0.379901	11.05	2.3205	25.72	6.5583	22.05	G55
0.52	0.94405	0.308782	10.95	2.0805	25.51	5.5803	19.95	G55
0.72	0.876781	0.335472	11.38	2.0484	25.62	5.2137	19.38	G55
0.67	0.926343	0.315678	10.42	1.8756	26.15	5.0526	18.42	G55
0.43	0.23146	0.656608	8.565	1.79865	25.81	5.51145	17.065	G55
0.43	0.510888	0.503582	12.455	2.36645	25.73	6.08095	22.955	G55
0.39	0.54811	0.484756	11.05	2.3205	25.72	6.5583	22.05	G55
0.52	0.748905	0.389868	10.95	2.0805	25.51	5.5803	19.95	G55
0.72	0.06789	0.755173	11.38	2.0484	25.62	5.2137	19.38	G55
0.67	0.210083	0.669129	10.42	1.8756	26.15	5.0526	18.42	G55
0.52	0.426622	0.547587	2.165	0.28145	23.4	1.42545	2.665	R85
0.61	0.571033	0.473351	5.8	0.928	23.55	2.616	8.8	R85
0.57	0.682153	0.420145	5.85	0.9945	23.61	2.8985	9.35	R85
0.56	0.702549	0.410758	5.9	1.062	23.68	3.2535	9.9	R85
0.55	0.83282	0.353634	6.05	1.2705	23.75	4.12335	11.55	R85
0.55	0.985618	0.292956	5.555	1.16655	23.8	3.9417	10.555	R85
0.55	1.20542	0.217754	4.815	0.7704	23.89	2.5352	7.315	R85
1.97	0.831857	0.354038	1.5	0.15	24.11	1.1355	1.5	F52

1.87	0.881226	0.333668	2	0.4	24.22	2.533	3	F52
1.94	1.05133	0.26898	2	0.4	24.4	2.577	3	F52
1.84	1.2006	0.219251	2	0.4	24.68	2.58	3	F52
0.03	0.51757	0.500175	4.355	0.74035	30.36	2.6979	6.855	J66
0.03	0.570612	0.473559	5.335	0.90695	30.45	2.94525	8.335	J66
0.03	0.485468	0.516656	4.69	0.6566	30.54	2.1875	6.19	J66
0.02	0.671247	0.425213	4.75	0.7125	30.67	2.44725	6.75	J66
0.02	0.795028	0.369699	5.22	0.7308	30.73	2.3436	7.22	J66
0.02	0.912478	0.321142	5.22	0.7308	30.78	2.3527	7.22	J66
0.02	1.10939	0.248861	5.22	0.7308	30.87	2.3772	7.22	J66
0.02	1.04161	0.272446	5.22	0.7308	30.73	2.3436	7.22	J66
0.02	1.11107	0.248293	5.22	0.7308	30.78	2.3527	7.22	J66
0.02	1.31666	0.185065	5.22	0.7308	30.87	2.3772	7.22	J66
0.78	1.20088	0.219162	2.165	0.28145	22.47	1.0465	2.665	C33
0.88	1.40169	0.162452	2.165	0.28145	22.44	1.06665	2.665	C33
0.94	1.54506	0.128809	2.165	0.28145	22.62	1.05495	2.665	C33
0.23	1.00488	0.285796	2	0.2	25.95	1.234	2	C37
0.2	1.26324	0.200318	2	0.2	25.93	1.239	2	C37
0.31	0.073077	0.751952	1.5	0.15	26.08	1.2135	1.5	D44
0.31	0.136724	0.71293	1.5	0.15	26.24	1.223	1.5	D44
0.3	0.285912	0.625222	1.5	0.15	26.34	1.242	1.5	D44
0.26	0.423392	0.549312	1.5	0.15	26.42	1.239	1.5	D44
0.23	0.603365	0.457512	2	0.2	26.45	1.259	2	D44
0.2	0.857096	0.343534	2	0.2	26.45	1.2605	2	D44
0.21	1.01696	0.281361	2	0.2	26.18	1.266	2	D44
0.28	1.19995	0.219453	2	0.2	26.19	1.267	2	D44
0.36	1.3058	0.188101	2.25	0.5625	26.39	3.47875	3.75	D44
0.33	1.51247	0.135974	1.75	0.2625	26.37	1.94025	2.25	D44
0.02	1.11796	0.245973	2.25	0.5625	29.43	3.29875	3.75	J66
0.02	1.31897	0.184424	2	0.4	29.54	2.533	3	J66
0.41	0.521169	0.498344	4	0.4	21.62	1.056	4	I64
0.39	0.565867	0.475908	4.5	0.45	21.75	1.152	4.5	I64
0.5	0.612579	0.453052	5.55	0.6105	21.98	1.41625	6.05	I64
0.45	0.508445	0.504831	5.55	0.6105	22.01	1.4982	6.05	I64
0.39	0.700553	0.411671	5.55	0.6105	22.03	1.5301	6.05	I64
0.37	0.811674	0.362572	5.6	0.672	22.02	1.7778	6.6	I64
0.43	0.929124	0.314589	7.075	0.849	22.14	1.9482	8.075	I64
0.48	1.1197	0.24539	6.625	0.86125	22.27	2.11445	8.125	I64
0.41	0.336392	0.596786	4	0.4	21.62	1.056	4	I64
0.39	0.471516	0.523906	4.5	0.45	21.75	1.152	4.5	I64
0.5	0.628588	0.445359	5.55	0.6105	21.98	1.41625	6.05	I64
0.45	0.746039	0.391142	5.55	0.6105	22.01	1.4982	6.05	I64
0.39	0.772764	0.379358	5.55	0.6105	22.03	1.5301	6.05	I64
0.37	0.915697	0.319869	5.6	0.672	22.02	1.7778	6.6	I64
0.43	1.06849	0.262929	7.075	0.849	22.14	1.9482	8.075	I64
0.48	1.25031	0.204134	6.625	0.86125	22.27	2.11445	8.125	I64
0.41	0.406591	0.558326	4	0.4	21.62	1.056	4	I64
0.39	0.618365	0.450263	4.5	0.45	21.75	1.152	4.5	I64
0.5	0.772267	0.379575	5.55	0.6105	21.98	1.41625	6.05	I64
0.45	0.73301	0.396961	5.55	0.6105	22.01	1.4982	6.05	I64
0.39	0.811579	0.362612	5.55	0.6105	22.03	1.5301	6.05	I64

0.37	0.917921	0.318991	5.6	0.672	22.02	1.7778	6.6	I64
0.43	1.08803	0.256147	7.075	0.849	22.14	1.9482	8.075	I64
0.48	1.23096	0.209932	6.625	0.86125	22.27	2.11445	8.125	I64
0.71	1.00113	0.287184	1.75	0.2625	22.51	1.39425	2.25	C21
1.35	0.924662	0.316338	4.935	0.93765	22.54	2.6125	5.335	C36
1.4	1.12157	0.244764	4.935	0.93765	22.64	2.60965	5.335	C36
1.35	0.482296	0.5183	4.935	0.93765	22.54	2.6125	5.335	C36
1.4	0.551438	0.483091	4.935	0.93765	22.64	2.60965	5.335	C36
0.4	0.64103	0.43943	1.26	0.189	20.9	1.21275	1.26	C38
0.41	0.854872	0.344453	1.755	0.26325	21.05	1.4325	2.755	C38
0.49	0.95422	0.304863	1.775	0.284	21.17	1.5632	2.775	C38
0.36	1.32417	0.182986	1.835	0.31195	21.28	1.76035	2.835	C38
1.35	1.04709	0.27049	4.935	0.93765	22.54	2.6125	5.335	C36
1.4	1.31549	0.18539	4.935	0.93765	22.64	2.60965	5.335	C36
0.78	1.52349	0.133519	3.1	0.372	22.99	1.2858	3.6	E50
0.67	0.414931	0.553842	1.5	0.15	21.08	0.8235	1.5	C41
0.36	0.111373	0.728361	1.455	0.27645	23.3	1.91235	1.455	C26
0.72	0.904679	0.324241	2.5	0.25	21.3	1.0255	2.5	R85
0.49	1.07479	0.260732	2.5	0.25	21.41	1.032	2.5	R85
0.45	1.20506	0.217865	2.625	0.34125	21.46	1.42545	3.125	R85
0.95	1.50778	0.137028	2.365	0.40205	22.83	1.72635	2.365	C39
0.63	0.199325	0.675472	1.75	0.2625	22.07	1.37025	2.25	M74
0.35	0.613229	0.452738	1.75	0.2625	21.99	1.485	2.25	G55
0.33	0.699028	0.41237	2	0.4	22.03	2.078	3	G55
0.32	0.757405	0.386105	1.75	0.2625	22.06	1.485	2.25	G55
0.37	0.875016	0.33619	2	0.4	22.2	2.083	3	G55
0.35	0.996162	0.289023	2.25	0.5625	22.33	2.74	3.75	G55
0.32	1.18431	0.22436	2.25	0.5625	22.4	2.74625	3.75	G55
0.59	1.04429	0.27149	1	0.1	21.03	0.89	1	N77
0.57	1.31269	0.186171	1.5	0.15	21.03	0.902	1.5	N77
0.57	1.24938	0.204411	1.5	0.15	21.03	0.902	1.5	N77
0.93	1.29114	0.192252	3	0.3	21.71	0.927	3	C21
1.02	0.326835	0.60212	2.335	0.39695	21.38	1.6728	2.335	C36
0.83	0.512613	0.502701	2.82	0.4512	21.86	1.8848	4.42	C36
0.96	0.694193	0.414589	2.835	0.48195	22.05	2.26865	5.835	C36
0.55	0.82466	0.357067	6.375	3.06	23.84	13.4928	21.375	M73
0.5	1.04446	0.271429	6.125	2.63375	24.1	11.2682	19.125	M73
0.45	1.16522	0.230447	2.625	0.34125	21.07	1.3728	3.125	C34
0.41	1.37714	0.168772	3.1	0.372	21.16	1.3182	3.6	C34
0.7	1.2439	0.206044	5.22	0.7308	21.68	1.8998	7.22	C34
1.43	0.961297	0.302154	2	0.2	22.36	0.9885	2	C34
1.56	1.01086	0.283596	2	0.2	22.45	1.042	2	C34
1.08	0.581788	0.46805	3.5	0.35	21.56	1.067	3.5	C21
1.07	0.814846	0.361222	3	0.3	21.94	1.056	3	C21
0.95	1.08072	0.25867	3.5	0.35	22.25	1.096	3.5	C21
0.95	1.29114	0.192252	3.5	0.35	22.25	1.096	3.5	C21
0.99	0.91933	0.318435	2.315	0.3704	20.6	1.7144	3.365	C36
0.98	0.359332	0.584077	1.34	0.2278	22.35	1.5113	1.34	C38
1.11	0.627952	0.445663	1.335	0.22695	22.37	1.5028	1.335	C38
1.21	1.37273	0.169926	1.32	0.2112	22.22	1.3888	1.32	C38
1.4	0.464744	0.527443	1.5	0.15	22.32	0.9525	1.5	C13

1.01	0.346936	0.590928	1.19	0.1666	22.24	1.0374	1.19	C38
0.67	0.450409	0.534973	1.46	0.2774	22.24	1.9228	1.46	C38
0.67	0.639132	0.440332	1.765	0.26475	22.23	1.29825	1.765	C38
0.55	0.756583	0.386468	2.265	0.33975	22.29	1.37175	2.265	C38
0.75	1.33976	0.178722	1.88	0.3384	22.43	1.9413	2.88	C27
0.88	1.22818	0.210773	1.435	0.27265	22.4	1.87435	1.435	C26
0.51	1.18665	0.223621	2	0.4	22.2	2.29	3	I64
1.64	1.07958	0.259066	4.435	0.84265	22.16	2.4434	4.435	C38
1.24	1.22884	0.210573	4.45	0.8455	22.19	2.5346	4.8	C38
1.23	1.32218	0.183536	2.85	0.4845	23.58	1.88785	3.45	C39
1.17	1.04111	0.272626	4.435	0.84265	22.72	2.50895	4.785	C39
1.11	1.31699	0.184973	3.1	0.372	22.42	1.5138	3.6	C13
1.43	0.928358	0.314889	1.5	0.15	21.02	0.9335	1.5	I64
1.29	1.08116	0.25852	1.5	0.15	21.19	0.9965	1.5	I64
1.07	1.2693	0.198547	1.5	0.15	21.37	1.026	1.5	I64
0.48	0.957147	0.303741	1.465	0.27835	21.14	1.92755	1.465	C27
0.44	1.06502	0.264147	1.43	0.2717	21.54	1.85725	1.43	C27
0.56	0.783484	0.374689	6.335	1.07695	22.39	2.7285	8.535	C40
0.51	1.05849	0.266447	6.335	1.07695	22.59	2.82115	9.085	C40
0.39	1.12794	0.24264	6.36	1.0812	22.58	3.0532	10.21	C40
0.44	1.33354	0.180416	7.36	1.2512	22.64	3.1195	10.61	C40
0.56	0.382002	0.571652	6.335	1.07695	22.39	2.7285	8.535	C40
0.51	0.588786	0.464618	6.335	1.07695	22.59	2.87045	9.085	C40
0.39	0.768158	0.381374	6.36	1.0812	22.58	3.0532	10.21	C40
0.44	0.950123	0.306438	7.36	1.2512	22.64	3.128	10.61	C40
0.51	1.0435	0.271772	1.5	0.15	23.16	1.084	1.5	N77
0.9	1.10295	0.251043	1.29	0.2064	21.77	1.4232	1.79	C26
1.06	1.28476	0.194075	1.325	0.22525	21.81	1.57335	1.825	C26
2.54	-0.59318	1.20993	1.5	0.15	22.02	1.0385	1.5	C13
2.5	-0.35557	1.03717	1.5	0.15	22.14	1.0465	1.5	C13
2.55	-0.06728	0.841205	1.5	0.15	22.37	1.066	1.5	C13
2.45	0.184489	0.684265	1.5	0.15	22.51	1.1025	1.5	C13
2.44	0.365565	0.580648	3	0.3	22.66	1.221	3	C13
2.21	0.522936	0.497447	2.625	0.34125	22.94	1.6289	3.125	C13
1.31	0.054554	0.763482	2.5	0.25	20.64	1.017	2.5	C34
0.77	0.855509	0.344189	3.585	0.4302	21.81	1.3644	4.085	C34
0.78	0.985621	0.292955	3	0.3	21.99	1.112	3	C34
0.71	1.18253	0.224923	3	0.3	22.34	1.1155	3	C34
0.65	0.841467	0.350017	4.07	0.4477	20.92	1.2188	4.57	C41
0.44	0.922488	0.317192	4.815	0.7704	21.58	2.1176	7.315	C41
0.38	1.06727	0.263357	5	1	21.73	2.976	9	C41
0.48	1.18488	0.224178	5.25	1.3125	21.62	4.30125	11.25	C41
0.65	0.782705	0.375027	4.07	0.4477	20.92	1.2188	4.57	C41
0.44	0.909367	0.322376	4.815	0.7704	21.58	2.1976	7.315	C41
0.38	0.933608	0.312838	5	1	21.73	3.038	9	C41
0.48	1.16031	0.232027	5.25	1.3125	21.62	4.42625	11.25	C41
0.4	0.557044	0.480294	1.475	0.28025	22.06	1.9456	1.475	I65
0.73	1.09155	0.254938	1.26	0.189	21.98	1.21875	1.26	C26
0.87	1.54599	0.128608	3.3	0.528	22.04	2.0664	4.8	C21
0.87	1.54599	0.128608	3.3	0.528	22.04	2.0664	4.8	C21
0.53	0.930891	0.313898	3.495	0.699	22.52	2.594	3.995	C37

0.47	1.03244	0.275744	4.05	0.8505	22.75	2.95575	4.65	C37
0.44	1.30084	0.189497	4.05	0.8505	22.95	3.02715	4.95	C37
0.45	0.919558	0.318345	1.425	0.2565	20.72	1.8414	1.925	C35
0.55	1.07236	0.26158	1.925	0.3465	21.1	1.8423	1.925	C35
0.45	0.921782	0.317469	1.425	0.2565	20.72	1.8414	1.925	C35
0.55	1.09189	0.25482	1.925	0.3465	21.1	1.836	1.925	C35
0.86	-0.41885	1.08226	5.165	0.67145	21.8	1.6172	6.665	C34
0.77	-0.11909	0.875216	5.165	0.67145	22.13	1.6107	6.665	C34
0.53	0.129196	0.717497	5.165	0.67145	22.1	1.61915	6.665	C34
0.51	0.641263	0.439319	5.335	0.90695	22.12	2.3341	8.335	C34
0.57	0.862968	0.341118	5.65	0.7345	22.16	1.6367	7.15	C34
0.61	1.54307	0.129239	5.165	0.67145	22.32	1.7173	6.665	C34
0.96	0.941126	0.309914	2.7	0.378	21.5	1.3986	3.7	C38
1.01	0.936556	0.311689	2.745	0.41175	22.06	1.50975	3.345	C36
0.95	1.08935	0.255691	4.255	0.63825	22.36	1.97325	6.355	C36
0.92	1.2775	0.196167	4.245	0.63675	22.39	1.95525	6.345	C36
0.92	1.25815	0.201814	4.245	0.63675	22.39	1.95525	6.345	C36
0.49	1.25217	0.203581	1.75	0.2625	20.89	1.3635	2.25	C39
0.75	1.59593	0.118174	1.5	0.15	20.97	0.7225	1.5	C14
0.92	-0.32946	1.01877	1.345	0.22865	23.01	1.51895	1.345	C37
0.99	-0.09966	0.862391	1.855	0.31535	23.16	1.6269	1.855	C37
0.95	0.093641	0.739242	1.855	0.31535	23.21	1.6269	1.855	C37
0.84	0.242455	0.650212	2.365	0.40205	23.3	1.7272	2.365	C37
0.84	1.51177	0.136132	2.365	0.40205	23.3	1.7272	2.365	C37
0.33	0.923852	0.316656	2	0.2	21.66	0.888	2	M74
0.29	1.08911	0.255776	2.75	0.4125	22.1	1.545	3.75	M74
0.33	1.04628	0.270779	2	0.2	21.66	0.888	2	M74
0.29	1.28303	0.194572	2.75	0.4125	22.1	1.62	3.75	M74
0.7	0.925535	0.315995	4.875	0.8775	23.53	2.8161	7.875	C32
0.8	1.10365	0.250803	5.22	0.7308	23.67	2.1644	7.22	C32
0.88	1.32415	0.182991	5.065	1.06365	23.87	3.77265	9.565	C32
0.76	0.812945	0.36203	1.5	0.15	22.43	0.875	1.5	C29
0.81	0.97126	0.298365	1.5	0.15	22.46	0.8825	1.5	C29
0.79	1.02082	0.279953	1.5	0.15	22.59	0.888	1.5	C29
0.97	1.48902	0.141303	4.25	1.0625	23.23	4.0625	8.75	C21
1.88	0.865054	0.340262	3	0.6	22.28	2.472	5	I64
1.95	1.03051	0.276438	3.4	0.612	22.55	2.3166	5.4	I64
1.71	1.23765	0.207916	4.75	0.7125	22.53	2.115	6.75	I64
1.83	0.787431	0.372978	2	0.2	21.6	0.921	2	G58
1.24	0.970531	0.298641	2.335	0.39695	22.86	1.9516	3.335	C32
1.68	1.57999	0.121435	1.5	0.15	22.75	0.9345	1.5	C24
0.62	1.06608	0.263775	2.285	0.3656	21.22	1.668	3.335	C36
0.78	1.33449	0.180157	2.32	0.3712	21.45	1.7232	3.37	C36
1.15	0.807469	0.364364	2.35	0.3995	21.24	1.69915	2.35	C39
0.96	0.932803	0.313151	2.26	0.339	21.67	1.36275	2.26	C39
0.82	1.09658	0.253214	2.31	0.3696	21.77	1.5336	2.31	C39
0.62	1.34103	0.178377	8.18	1.9632	25.48	6.1344	17.68	B09
0.83	0.021287	0.784382	1.71	0.2394	21.38	1.1333	1.71	C36
0.91	0.278853	0.629249	2.78	0.4448	21.51	1.568	2.78	C36
1.79	0.57676	0.470524	10.27	1.5405	23.59	4.2885	21.67	C36
1.79	1.34082	0.178435	10.27	1.5405	23.59	4.2885	21.67	C36

0.83	1.25886	0.201605	1.36	0.2312	22.49	1.7187	2.36	C39
1.8	0.138614	0.711785	2.94	0.5586	23.6	2.16125	2.94	C39
0.44	1.36577	0.171758	1.785	0.2856	21.07	1.4128	1.785	C27
0.5	1.30176	0.189239	1.5	0.15	19.77	0.844	1.5	C30
0.3	1.35875	0.173619	3.275	0.49125	22.54	1.98675	6.275	C27
0.48	0.937593	0.311286	3.975	0.795	21.75	2.554	3.975	C39
0.54	1.20228	0.218727	3.965	0.75335	21.88	2.49565	3.965	C39

diversity	interisit	jvs_dig
42	133.49	0
42	132.72	0
42	134.47	0
210	358.47	21
468	413.64	36
210	358.47	21
468	413.64	36
210	358.47	21
468	413.64	36
4	16.89	1
77	187.11	11
35	86.85	5
24	70.88	4
81	156.78	9
10	17.23	0
11	15.84	0
11	16.18	0
11	16.15	0
13	16.31	0
52	65.96	0
8	16.63	1
9	16.91	1
10	17.23	1
11	15.84	1
11	16.18	1
11	16.15	1
13	16.31	1
52	65.96	4
8	16.63	1
9	16.91	1
10	17.23	1
11	15.84	1
11	16.18	1
11	16.15	1
13	16.31	1
52	65.96	4
8	16.63	1
9	16.91	1
10	17.23	1
11	15.84	1
11	16.18	1
11	16.15	1
13	16.31	1
52	65.96	4
6	38	0
28	133.63	0
12	37.78	0
16	36.66	0
16	36.78	0
110	200.53	0

132	414.48	22
72	164.97	9
6	18.49	1
16	36.78	2
110	200.53	11
220	405.24	22
90	164.97	9
110	200.53	11
220	405.24	22
90	164.97	9
36	126.27	9
40	142.08	8
156	106.56	6
15	18.45	0
32	37.24	0
45	55.98	0
68	74.64	0
45	88.4	0
60	110.7	0
119	123.62	0
128	142.64	0
117	156.87	0
130	198.4	0
110	202.95	11
204	211.92	12
208	231.79	13
2	16.98	0
4	33.96	0
6	51	0
8	68.04	0
10	80.8	0
12	97.5	0
14	114.38	0
16	129.12	0
18	149.58	0
20	181.5	0
3	19.75	0
12	40.72	0
15	68.52	0
28	84.36	0
40	105.7	0
48	126.84	6
12	92.7	0
15	78.85	0
18	98.4	0
4	17.61	1
8	34.86	2
12	52.32	3
16	70.16	4
25	89.6	5
30	107.46	6

35	126.07	7
40	143.6	8
45	141.12	9
60	155.1	10
55	197.12	11
60	214.92	12
65	234.13	13
70	251.3	14
75	235.2	15
96	248.16	16
102	263.67	0
4	16.48	1
5	18.62	1
64	308	0
120	560.1	0
140	653.45	0
261	541.43	0
270	530.55	0
255	945.54	0
360	1111.2	0
304	646.76	0
400	987.5	0
774	889.24	0
64	308	16
261	515.91	29
270	480.33	27
774	846.24	43
1	18.59	0
1	18.19	0
1	19.25	0
4	17.21	0
9	16.25	0
10	16.1	1
10	16.23	1
4	17.21	0
3	17.13	0
4	14.91	0
7	16.33	0
9	16.25	0
10	16.1	0
10	16.23	0
4	17.21	1
3	17.13	1
4	14.91	1
7	16.33	1
9	16.25	1
10	16.1	1
10	16.23	1
7	16.33	0
9	16.25	0
10	16.1	0

10	16.23	0
9	16.25	1
10	16.1	1
10	16.23	1
10	16.1	0
10	16.23	0
30	17.53	1
58	35.06	2
87	48.09	3
112	64.08	4
155	76.8	5
78	90.36	6
91	111.23	7
112	127.12	8
126	143.01	9
5	19.95	1
10	38.02	2
24	55.68	3
36	68.2	4
45	84.8	5
54	108.12	6
63	126.14	7
56	144.16	8
72	158.04	0
90	179.5	0
99	197.45	0
108	215.4	0
117	242.58	0
140	261.24	0
3	18.6	1
8	37.2	2
12	56.88	0
12	73.52	0
15	91.8	0
18	111.06	0
21	137.06	0
16	159.04	0
36	161.64	9
30	183.3	10
33	201.96	11
36	222.12	12
39	241.54	13
24	161.52	0
96	661.44	0
24	165.04	0
96	661.44	0
24	173.04	0
96	663.68	0
9	202.68	0
156	845.91	0
126	870.24	0

126	871.5	0
11	19.93	1
12	20.29	1
11	19.93	1
12	20.29	1
10	39	2
10	38.96	2
12	38.74	2
24	79.6	4
16	40.28	2
18	61.44	3
12	20.29	1
5	19.29	0
12	39.96	0
3	18.07	1
5	17.56	1
6	17.85	1
6	18.74	1
14	37.22	2
70	187.4	10
300	578.1	30
480	765.2	40
5	17.56	1
6	17.85	1
6	18.74	1
14	37.22	2
70	187.4	10
300	578.1	30
480	765.2	40
300	578.1	30
480	765.2	40
300	578.1	30
480	765.2	40
300	578.1	30
480	765.2	40
12	74.36	4
20	85.9	5
24	96.12	6
35	115.22	7
40	131.28	8
45	163.62	9
50	183.9	10
55	224.4	11
35	115.22	7
40	131.28	8
45	163.62	9
50	183.9	10
55	224.4	11
2	34.84	0
3	52.14	0
11	189.2	0

6	98.82	0
9	163.08	0
28	245.28	0
32	282.08	0
12	215.28	0
20	176.1	0
28	245.28	14
32	282.08	16
20	176.1	10
7	16.92	1
20	34.2	2
33	52.56	3
40	70.52	4
50	87.5	5
66	103.8	6
77	122.5	7
104	138.64	8
117	157.5	9
140	175.6	10
121	192.72	0
120	211.56	0
130	227.5	0
154	242.2	0
165	262.5	0
208	277.28	0
221	297.5	0
252	316.08	0
247	329.27	19
260	350	20
294	368.76	21
308	386.32	22
10	205	10
57	374.11	19
9	15.49	1
9	15.49	0
9	16.25	1
11	16.1	1
9	16.51	1
9	16.25	1
11	16.1	1
9	16.51	1
84	577.08	0
324	2147.04	108
4	33.98	0
2	17.1	0
2	17.22	0
2	17.22	0
4	68.2	0
3	50.97	0
4	68.16	0
6	102.54	0

13	221.65	0
1	12.62	0
16	113.84	0
12	101.52	0
10	84.6	0
4	33.84	0
4	15	0
8	30.22	0
12	44.46	0
16	60	0
20	75.55	0
24	88.92	0
28	105	0
32	120.88	0
36	133.38	0
40	151.1	0
44	163.02	0
2	16.59	1
4	34.82	2
6	49.77	0
8	69.64	0
3	19.43	0
6	38.86	0
15	58.29	0
4	21.34	1
4	21.36	1
4	21.86	1
6	21.47	1
24	86.52	4
6	21.62	1
60	215.8	10
48	172.16	8
49	148.26	7
6	21.47	1
24	86.52	4
6	21.62	1
60	215.8	10
48	172.16	8
49	148.26	7
49	148.26	7
3	15.8	0
8	30.52	0
12	45.51	0
16	60.68	0
116	442.54	0
44	173.8	0
40	158.7	0
120	483.9	0
160	645.2	0
3	21.58	0
6	42.76	0

9	64.74	0
12	86.72	0
15	106.9	0
18	131.82	0
21	150.29	0
24	172.64	8
27	192.42	9
30	215.8	10
33	238.48	11
36	256.56	12
39	285.61	13
42	300.58	14
4	17.91	0
42	116.82	0
14	39.8	0
9	62.64	3
3	51.96	3
4	76.24	4
5	95.3	5
6	103.92	0
7	133.42	0
8	152.48	0
340	854.76	68
350	1171.8	70
290	787.06	58
435	1538.16	87
4	12.27	0
7	18.25	1
9	18.07	1
9	18.18	1
9	17.89	1
9	17.91	1
10	17.97	1
10	18.42	1
20	36.9	2
30	55.56	3
1	17.55	1
3	52.53	3
6	102.96	6
6	91.02	6
11	168.74	11
14	216.16	14
15	246	15
10	166.1	10
35	619.15	35
4	20.29	1
4	20.96	1
24	136.56	8
15	89.6	5
2	19.24	1

4	38.48	2
9	57.72	3
8	76.96	0
15	96.2	0
18	115.44	0
4	20.34	0
4	43.94	2
9	61.5	3
16	81.36	0
20	67.16	4
35	117.53	7
10	33.58	2
6	18.42	1
12	38.28	2
15	57.39	3
32	74.72	4
50	88.95	5
78	105.18	6
91	124.11	7
48	146.88	8
135	157.68	9
150	181.5	10
66	202.62	0
72	229.68	0
65	248.69	0
112	261.52	0
150	266.85	0
208	280.48	0
221	301.41	0
108	330.48	0
285	332.88	0
300	363	0
168	392.28	21
220	391.38	22
299	403.19	23
312	425.52	24
150	459	25
390	455.52	26
405	490.05	27
224	523.04	28
290	515.91	29
390	525.9	30
403	549.63	31
192	587.52	32
495	578.16	33
510	617.1	34
350	622.65	35
468	631.08	36
481	656.01	37
228	697.68	38
585	683.28	39

600	726	40
410	729.39	41
546	736.26	42
559	762.39	43
264	807.84	44
675	788.4	45
690	834.9	46
705	823.44	47
720	871.2	48
3	21.31	0
4	20.78	0
4	20.63	0
2	18.33	1
5	18.91	1
720	1133.6	80
504	1038.87	63
150	425.1	30
357	714.51	51
720	1133.6	80
504	829.71	63
1	19.75	1
2	39.74	2
3	59.94	3
4	80	4
50	86.45	5
72	104.82	6
84	118.16	7
96	135.44	8
108	153	9
100	172.9	10
132	192.17	11
144	202.56	12
156	220.09	13
168	238	14
2	16.34	1
4	32.24	2
6	48.36	3
8	65.36	4
10	85.9	5
12	103.08	6
14	124.81	7
16	150.64	8
2	17.1	1
2	17.3	1
2	17.3	1
2	18.17	1
40	367.8	20
99	603.24	33
96	578.88	32
30	183.9	10
99	596.64	33

96	578.56	32
8	39.34	2
12	59.25	3
16	79.28	4
3	19.49	1
6	39.44	2
7	20.75	1
16	41.46	2
15	62.25	3
28	83	0
40	104.15	0
9	15.85	1
34	31.7	2
57	47.55	3
80	61.84	4
30	80.35	0
54	96.42	0
119	112.49	0
152	128.56	0
180	148.14	0
5	16.44	0
35	115.08	0
15	49.32	0
15	49.32	0
24	65.76	0
10	32.88	0
2	19.04	0
1	18.1	0
1	18.1	0
1	18.2	0
1	17.41	0
1	17.63	0
1	17.71	0
2	35.64	0
3	54.39	0
4	77.52	0
4	18.84	1
6	38.68	2
12	57.99	3
16	78.12	4
15	99.5	5
18	120.66	6
6	17.56	1
6	17.76	1
7	17.67	1
5	17.39	1
6	17.56	1
6	17.76	1
7	17.67	1
5	17.39	1
6	17.56	1

6	17.76	1
7	17.67	1
4	16.1	0
8	32.2	0
18	54.96	0
20	73.48	0
20	80.5	5
24	96.6	6
42	128.24	7
40	146.96	8
1128	2336.37	141
1128	2336.37	141
1128	2336.37	141
219	1307.43	73
237	1401.46	79
255	1484.1	85
1128	2336.37	141
1128	2336.37	141
219	1307.43	73
237	1401.46	79
255	1484.1	85
1128	2336.37	141
11	15.91	1
24	31.82	0
12	16.74	1
225	1385.25	75
400	1436.8	80
4	36.94	2
2	18.22	1
4	37.62	2
18	60	3
24	80	4
10	40.44	2
30	100	0
5	20.22	0
2	21.05	0
2	32.84	2
3	49.05	3
4	65.28	4
5	81.6	5
6	98.1	6
7	114.94	7
16	131.36	8
18	162.99	9
30	181.2	10
33	199.32	11
9	22.44	1
14	45.68	2
15	65.91	0
20	89.56	4
64	601.28	32

52	488.54	26
58	544.91	29
2	16.21	0
2	16.32	0
4	32.52	0
8	32.68	2
7	17.58	0
14	35.16	0
7	17.58	0
8	17.58	0
27	52.74	0
40	70.32	0
6	17.72	1
7	17.58	1
14	35.42	2
7	17.71	1
8	17.71	1
27	54.75	3
40	73	4
3	18.53	1
3	16.8	1
8	33.6	2
4	16.01	1
5	15.74	1
5	15.74	1
1	18.27	1
1	19.29	1
6	19.18	1
8	16.42	0
12	47.49	0
16	62.8	0
20	77.85	0
24	92.76	0
28	116.2	0
32	132.48	0
36	147.24	0
8	16.42	2
12	47.49	3
16	62.8	4
20	77.85	5
24	92.76	6
28	116.2	7
32	132.48	8
36	147.24	9
7	15.65	1
9	15.65	1
8	36.8	0
12	55.2	0
4	8.21	0
8	31.66	0
12	47.1	0

16	62.28	0
20	77.3	0
24	99.6	0
28	115.92	0
32	130.88	0
9	18.18	0
3	17.73	0
33	664.29	0
64	623.36	0
120	602.4	0
42	281.82	0
63	422.73	0
147	986.37	0
159	1037.74	0
456	2220.72	0
160	779.2	0
1	17.33	1
6	38.62	2
12	57.93	3
20	77.24	4
26	221.52	13
16	136.72	8
16	68.36	4
12	52.62	3
12	54.06	3
14	35.44	2
22	34.74	2
32	36.04	2
3	14.72	1
5	13.72	1
5	13.69	1
6	13.69	1
18	43.26	3
6	14.82	1
21	43.56	3
14	28.64	2
7	14.82	1
2	35.08	2
3	52.62	3
4	70.8	4
5	88.75	5
6	106.5	6
14	125.16	7
16	145.84	8
27	164.07	9
24	201.24	12
20	167.7	10
36	201.24	12
72	301.86	18
3	10.41	1
9	31.23	3

3	13.08	1
3	13.68	1
9	41.04	3
2	17.48	1
6	17.95	1
6	17.95	0
72	418.32	0
136	610.3	0
66	400.62	0
210	358.47	0
468	413.64	0
2	19.99	1
6	16.13	1
16	65.08	4
28	65.8	4
10	32.6	2
6	16.13	0
16	65.08	0
28	65.8	0
8	33.78	0
8	16.63	1
9	16.91	1
11	16.18	1
11	16.15	1
13	16.31	1
52	65.96	4
10	17.23	0
11	15.84	0
11	16.18	0
11	16.15	0
13	16.31	0
52	65.96	0
8	16.63	1
9	16.91	1
10	17.23	1
11	15.84	1
11	16.18	1
11	16.15	1
13	16.31	1
52	65.96	4
2	18.96	1
2	18.87	1
10	31.34	2
369	642.47	41
216	371.28	24
48	122.48	8
54	136.35	9
40	122.32	8
8	32.88	2
16	66.56	4
4	17.64	1

8	35.28	2
8	35.28	2
8	32.96	2
48	197.76	12
40	164.8	10
16	154.48	8
1	18.43	0
1	18.53	0
1	18.63	0
1	18.67	0
1	19.6	0
1	18.42	0
1	19.3	0
1	20.36	0
1	20.58	0
1	19.81	1
1	19.84	1
1	19.83	1
1	19.83	1
4	19.98	1
4	36.26	2
6	54.39	3
8	72.52	4
10	89.2	5
12	106.5	6
21	124.25	7
12	33.74	2
24	67.44	4
28	67.04	4
7	16.77	1
7	17.06	1
42	119.42	7
30	82.65	5
72	198.36	12
28	67.04	0
7	16.76	0
7	16.76	0
42	112.42	0
30	77.65	0
72	186.36	0
1	16.22	1
3	15.86	1
3	15.37	1
3	15.37	1
3	15.37	1
3	15.37	1
3	15.37	1
3	15.37	1
4	37.7	2
4	18.63	1
4	18.63	1

2	19.39	1
3	18.4	1
3	18.3	1
2	18.3	1
4	36.8	2
2	19.39	1
2	19.39	1
12	58.17	3
6	39.28	2
6	39.52	2
2	18.26	0
1	19.59	0
2704	3856.58	338
261	409.19	29
264	354.72	24
730	1068.72	73
261	409.19	0
264	354.72	0
730	1083.32	0
3	17.16	0
3	17.16	0
28	73.56	0
28	65.56	4
42	416.22	21
46	426.42	23
27	525.15	27
25	491.25	25
66	1313.4	66
42	877.8	42
47	982.3	47
104	1086.8	52
78	811.2	39
24	70.88	4
81	156.78	9
24	70.88	4
81	156.78	9
2	18.02	1
77	187.11	11
35	86.85	5
81	156.78	9
2	18.68	1
4	34.36	2
6	51.39	3
8	68.52	4
10	88.5	5
12	112.08	6
14	130.76	7
16	151.84	8
18	171.9	9
20	191	10
2	14.79	0

4	29.58	0
6	44.58	0
8	60.04	0
10	77.75	0
90	174.2	10
5	15.36	1
9	15.32	1
20	30.36	2
9	15.96	1
9	15.77	1
39	45.87	3
77	187.11	0
35	86.85	0
2	14.61	0
2	14.4	0
2	14.4	0
2	14.4	0
77	187.11	11
35	86.85	5
9	15.32	1
20	30.36	2
9	15.96	1
9	15.77	1
39	45.87	3
9	15.32	1
20	30.36	2
9	15.96	1
108	510.57	0
80	373	0
80	360.6	0
88	352.66	22
26	210.21	13
48	385.68	24
52	444.86	26
106	976.26	53
134	1228.11	67
372	1587.51	93
295	1086.78	59
3	15.69	1
3	15.69	1
9	15.77	1
39	45.87	3
39	45.87	3
39	45.87	3
3	16.61	0
3	16.05	0
3	16.05	0
9	46.68	0
15	77.8	0
9	48.39	0
3	16.97	1

3	16.97	1
6	51.12	0
3	16.91	0
16	65.64	0
4	17.18	0
35	120.26	0
15	54.63	0
30	112.62	0
24	112.62	0
6	34.08	0
20	71.28	0
18	51.09	0
36	70.08	0
18	51.09	3
36	70.08	4
3	16.68	1
364	1572.48	0
352	1520.64	0
704	1587.52	0
92	1644.04	0
440	1578.72	0
364	1692.6	91
440	1615.68	88
704	1620.08	88
164	1184.08	82
182	1308.58	91
176	1261.04	88
352	1270.72	88
2	20.01	0
110	370.7	22
161	395.14	23
12	71.36	4
88	385.66	22
125	409.75	25
120	393.36	24
120	403.92	24
432	1336.32	72
1272	2890.62	159
1368	3112.2	171
11	17.74	1
26	36.24	2
60	56.64	3
96	77.72	4
110	360.36	22
125	409.75	25
120	393.36	24
120	403.92	24
432	1336.32	72
1272	2890.62	159
1272	2890.62	159
1368	3112.2	171

264	425.76	24
936	1304.64	72
3180	3160.92	159
6	42.93	3
6	43.83	3
9	45.33	3
9	45.33	3
9	48.39	3
12	50.94	3
15	50.88	3
24	16.43	1
425	1376.15	85
500	1613	100
462	1295.14	77
426	1195.64	71
10	31.92	0
12	47.94	3
2	18.26	1
44	68.6	4
36	52.26	3
33	52.47	3
39	53.55	3
376	605.83	47
2	18.51	1
7	10.79	1
14	35.52	0
18	54.42	0
28	75.16	0
30	95.35	0
42	115.32	0
49	135.38	0
48	158.4	0
5	15.82	1
10	158.9	10
14	230.3	14
120	1010.4	60
166	1397.72	83
1	11.29	1
1	11.29	1
1	12.8	1
8	119.44	8
7	106.05	7
2	33.24	2
22	182.82	11
14	38.36	2
12	38.76	2
13	19.63	1
2	17.56	1
4	32.46	2
9	47.07	3
16	61.72	4

25	75.05	5
30	88.56	6
42	111.79	7
3	19.94	1
3	19.84	1
4	19.65	1
5	19.65	1
5	19.84	1
5	19.94	1
12	39.88	2
2	18.66	1
4	37.3	2
4	38.74	2
6	15.97	1
3	13.82	1
20	193	10
26	256.36	13
13	265.33	13
3	60.99	0
2	40.66	0
4	81.32	0
11	223.63	0
20	203.3	0
26	264.29	0
75	271.35	15
40	144.32	8
75	231.15	15
1	18.24	1
1	18.57	1
1	18.99	1
2	11.92	1
2	11.13	1
2	14.96	1
2	14.89	1
2	14.05	1
3	15.62	1
3	16.33	1
3	17.34	1
1	16.39	1
3	14.62	1
3	15.33	1
3	15.33	1
35	112.84	7
35	113.68	7
35	112.84	7
35	113.68	7
320	3006.4	160
714	4398.24	238
801	4979.55	267
956	4414.33	239
2	14.49	1

4	28.98	2
6	43.47	3
4914	1790.1	117
5896	2040.82	134
1584	940.5	66
3920	1596	112
4914	1667.25	117
5896	1906.82	134
1584	940.5	66
3920	1610.56	112
4914	1790.1	117
5896	2174.82	134
195	248.1	15
104	132.32	8
42	51.18	3
468	475.92	36
1584	940.5	66
3920	1596	112
4914	1667.25	117
5896	2174.82	134
6	15.16	1
21	45.48	3
72	136.44	9
261	439.64	29
352	667.04	44
472	894.44	59
368	697.36	46
385	833.8	55
3920	1610.56	112
4914	1790.1	117
5896	2174.82	134
3920	1610.56	112
4914	1682.46	117
5896	1926.92	134
3920	1599.36	112
4914	1790.1	117
5896	2050.2	134
4914	1790.1	117
5896	2174.82	134
1584	940.5	66
3920	1588.16	112
4914	1778.4	117
5896	2173.48	134
4914	1790.1	117
5896	2174.82	134
4914	1790.1	117
5896	2174.82	134
16	33.48	2
16	34.66	2
7	17.33	1
36	69.32	4

45	87.45	5
3920	1610.56	112
4914	1790.1	117
5896	2174.82	134
4914	1907.1	117
5896	2174.82	134
1584	940.5	66
3920	1610.56	112
4914	1790.1	117
5896	2193.58	134
3920	1713.6	112
4914	1788.93	117
5896	2174.82	134
3920	1825.6	112
4914	1790.1	117
5896	2062.26	134
150	245.25	15
80	132.56	8
39	49.77	3
195	248.7	15
112	136.48	8
30	49.05	0
150	248.55	0
104	132.32	0
39	48.93	0
42	49.71	0
21	47.46	3
72	148.86	9
261	479.66	29
352	753.28	44
472	1018.34	59
368	803.62	46
385	960.85	55
2	8.47	1
16	33.48	2
16	33.48	2
7	17.26	1
36	71.72	4
45	89.65	5
16	33.48	2
16	34.66	2
7	17.26	1
36	66.92	4
45	86.65	5
72	600.48	36
66	550.44	33
148	617.16	37
36	67.72	4
45	84.65	5
148	641.21	37
129	740.03	43

174	998.18	58
6	15.16	1
21	47.46	3
72	148.86	9
261	479.66	29
352	727.76	44
472	895.03	59
368	697.36	46
385	833.8	55
20	78.04	4
15	60.45	3
4	19.71	1
4	19.91	1
10	185.9	0
11	204.49	0
22	204.49	0
39	234.78	0
18	108	0
27	162	0
39	241.28	13
18	113.4	6
27	171.09	9
4	15.55	1
4	15.55	1
21	116.97	7
15	83.55	5
16	66.84	4
8	33.42	2
3	16.77	1
16	67.08	4
8	33.72	2
19	353.97	0
17	316.71	0
21	391.23	0
42	823.62	0
49	125.93	7
63	162.36	9
63	162.36	9
30	185.5	10
30	185.5	10
10	194.4	10
20	187.5	10
72	286.02	18
9	16.83	1
10	16.8	1
12	16.8	1
130	168	10
14	16.81	1
28	34	2
16	16.82	1
17	17.38	1

10	16.9	0
12	16.93	0
130	169.3	0
14	16.93	0
28	33	0
16	16.45	0
17	16.45	0
20	31.3	2
130	167.2	10
14	16.74	1
28	34	2
16	16.85	1
17	17.38	1
14	16.44	1
28	32.8	2
16	16.42	1
17	16.72	1
17	17.38	1
7	15.7	1
2	18.21	0
14	24.4	2
21	45.03	3
28	63.36	4
45	75.5	5
54	89.82	6
70	110.88	7
45	80.5	5
54	90.42	6
70	111.65	7
4	13.8	1
10	27.6	2
14	27.8	2
21	43.53	3
28	58.04	4
45	75.5	5
54	91.62	6
70	106.89	7
4	34.14	0
3	17.07	0
3	17.07	0
44	213.84	11
44	213.84	11
5	18.96	0
4	71.32	4
10	89.15	5
12	106.44	6
21	119.7	7
48	136.8	8
21	139.02	7
48	158.88	8
48	165.84	8

1	19.7	1
2	19.55	0
156	1110.72	0
168	1143.52	0
1	17.51	0
2	34.66	0
3	52.2	0
1	19.28	1
3	18.01	1
6	19.4	1
2	36.54	2
3	54.81	3
4	73.08	4
5	91.35	5
12	69.2	0
6	34.6	0
9	51.9	0
36	85.28	4
300	2009	0
490	2018.8	0
408	1469.48	0
40	157.2	8
15	58.95	3
36	85.28	4
45	106.6	5
490	2018.8	98
408	1469.48	68
2	14.69	1
2	17.56	1
2	17.54	1
2	17.52	1
4	14.04	1
4	14.04	1
24	44.3	2
3	18.81	1
6	35.24	2
9	52.86	3
3	20.98	1
6	41.96	2
6	42.42	2
396	1271.82	66
396	1271.82	66
3710	2687.1	106
3710	2687.1	106
2511	2130.63	93
3710	2687.1	106
629	642.69	37
1794	1613.82	78
2511	2130.63	93
3710	2687.1	106
629	642.69	0

1794	1613.82	0
2511	2130.63	0
3710	2687.1	0
629	642.69	37
1794	1613.82	78
2511	2130.63	93
3710	2687.1	106
629	642.69	37
1794	1613.82	78
2511	2130.63	93
3710	2687.1	106
1	17.23	0
2	17.67	0
2	16.72	0
2	16.28	0
1	17.11	0
2	21.56	0
2	21.74	0
1794	1613.82	78
2511	2130.63	93
3710	2687.1	106
3710	2687.1	106
3710	2687.1	106
3710	2687.1	106
629	642.69	37
1794	1613.82	78
2511	2130.63	93
3710	2687.1	106
1794	1613.82	78
2511	2130.63	93
3710	2687.1	106
1794	1613.82	78
2511	2130.63	93
3710	2687.1	106
4	37.28	0
4	37.28	0
2511	2130.63	93
3710	2687.1	106
629	642.69	0
1794	1613.82	0
2511	2130.63	0
3710	2687.1	0
3710	2687.1	106
15	94.45	0
3	18.89	0
18	113.34	0
1	18.16	0
4	36.32	0
9	52.44	0
16	69.44	0
78	506.22	26

132	199.1	11
36	54.3	3
132	199.1	11
36	54.3	3
10	99.45	5
10	96.3	5
15	101.75	5
15	101.75	5
15	101.75	5
15	104.4	5
3	49.92	3
3	56.61	3
6	51.27	3
6	52.65	3
69	399.51	23
140	607.95	35
180	781.65	45
152	660.06	38
135	781.65	45
140	607.95	35
180	779.85	45
152	655.12	38
10	34.68	0
12	34.68	0
14	33.76	0
28	69.24	0
63	121.17	0
10	34.68	2
12	34.68	2
14	34.68	2
28	69.36	4
63	121.38	7
12	35.3	2
14	35.76	2
28	78.44	4
63	137.27	7
63	136.71	7
63	136.71	7
2	14.91	1
4	33.22	2
6	50.82	3
7	17.86	0
7	17.95	0
7	17.95	1
2	15.5	1
2	15.51	1
6	36.14	2
3	18.07	1
3	18.07	1
3	18.18	1
6	31	2

6	31	2
6	36.14	0
3	18.11	0
3	18.14	0
8	77.76	4
22	213.07	11
18	175.32	9
12	238.32	12
18	185.49	9
6	36.14	0
3	18.11	0
3	18.18	0
6	36.36	0
6	36.2	0
3	14.84	1
3	14.9	1
33	163.9	11
3	14.9	1
3	14.9	1
3	14.86	1
3	14.54	1
4	21.14	1
35	147.07	7
24	80.68	4
49	147.98	7
4	22.14	1
35	154.28	7
24	88.04	4
49	154.07	7
24	84.68	4
49	148.19	7
75	427.25	25
4	16.19	1
10	34.06	2
75	427.25	25
50	427.25	25
70	598.15	35
36	307.62	18
32	273.44	16
9	15.12	1
5	17.03	1
3	19.61	1
490	2064.86	98
960	3451.2	160
960	3451.2	160
10	184.2	0
3	15.31	1
6	30.7	2
9	46.23	3
12	61.8	4
5	91.55	0

6	108.9	0
38	381.52	38
165	331.32	33
60	150.6	15
99	523.38	33
75	248.4	15
72	294.48	18
36	156.24	9
75	255.6	15
72	316.98	18
36	158.49	9
2	19.93	1
60	482.4	30
99	621.72	33
60	387.2	20
63	407.82	21
2	17.27	1
2	17.27	1
10	88.5	5
10	89.3	5
60	482.4	0
99	621.72	0
60	387.2	0
63	407.82	0
20	45.16	4
56	128.1	7
99	621.72	0
60	387.2	0
63	407.82	0
56	123.69	7
45	88.5	5
144	329.4	18
56	132.79	7
45	96.5	5
45	88.5	5
2	15.58	1
27	55.35	3
30	54.6	3
36	66.28	4
8	16.73	1
4	15.26	0
18	45.78	0
12	30.52	0
15	45.78	0
15	45.78	0
21	45.78	0
30	54.6	3
36	66.28	4
8	16.73	1
18	161.19	0
8	69.2	4

14	120.26	7
12	109.02	6
152	665.76	38
408	1671.78	102
186	993.24	62
25	80.1	5
2	18.85	1
4	15.26	0
18	45.78	0
12	30.52	0
18	51.48	3
12	34.32	2
15	51.42	3
15	51.15	3
21	51.15	3
2	18.85	0
10	34.84	0
8	34.06	2
10	34.84	2
8	36.08	0
3	18.29	1
6	115.74	0
5	92.05	0
1	17.75	1
2	36.9	2
3	55.26	3
4	80.92	4
5	100.95	5
20	87.35	5
28	122.64	7
1	18.77	1
22	35.54	2
18	60.15	3
48	160.16	8
5	85.55	0
12	108.6	0
28	126.49	0
24	155.04	0
36	175.68	0
22	39.54	0
6	18.88	0
12	36.04	0
12	36.04	0
30	97.85	0
15	102.15	0
18	58.95	0
48	161.6	0
15	105.15	0
22	35.54	0
22	41.54	0
6	18.88	0

12	37.76	0
12	38.56	0
30	97.85	0
10	90.5	5
24	104.94	6
28	136.43	7
24	155.04	8
36	184.68	9
40	195.2	10
9	62.88	0
6	41.92	0
12	85.8	4
12	85.8	4
12	85.8	4
16	150.8	0
16	150.8	0
8	75.4	0
4	37.7	0
12	113.1	0
10	94.25	0
16	158.24	8
8	79.12	4
4	39.56	2
12	118.68	6
10	98.9	5
4	34.06	0
8	34.22	0
16	68.44	0
80	136.32	0
24	68.12	0
60	102.18	0
8	32.22	2
16	64.44	4
80	160.08	8
24	80.56	4
60	120.84	6
80	128.88	8
24	64.44	4
60	120.78	6
60	123.78	6
4	15.75	0
56	232.26	0
44	181.28	0
76	313.12	0
1	18.13	1
1	18.13	1
2	19.94	1
6	60.39	3
4	40.12	2
6	59.73	3
4	39.88	2

5	72.55	5
14	135.94	7
10	97.35	5
1	19.52	0
2	40.3	0
3	54.93	0
4	75.2	0
3	16.09	0
6	33.44	0
6	33.1	0
16	67.2	0
68	275.23	17
85	275.23	17
175	435	25
196	504	28
266	665.38	38
343	850.64	49
68	292.23	17
85	299.2	17
175	440	25
196	492.8	28
266	650.56	38
343	835.94	49
114	422.18	0
2	16.75	1
4	33.58	2
2	17.2	1
4	41.04	2
4	16.92	1
6	33.16	2
9	50.07	3
3	17.09	1
3	17.09	1
5	10.56	1
60	63.66	6
100	113.8	10
60	57.66	6
100	96.8	10
60	57.72	6
100	99.8	10
44	195.8	11
1	16.95	0
6	35.22	2
12	52.83	3
12	52.38	3
4	39.16	2
3	19.58	1
2	19.58	1
2	18.01	1
4	36	2
2	18.01	1

4	36.02	2
6	36.58	2
15	54.87	3
15	58.98	3
15	59.19	3
10	39.46	2
8	13.68	1
16	24.14	2
18	27.98	2
7	16.44	1
18	187.47	9
21	145.39	7
78	566.54	26
20	147	10
22	163.35	11
240	531.9	30
200	444.5	25
20	147	10
22	168.85	11
240	519.9	30
200	365	25
203	445.15	29
240	440.7	30
200	337	25
203	499.67	29
2	17.64	1
4	18.26	1
4	39.2	2
6	59.01	3
106	954.53	53
80	725.2	40
60	554.4	30
2	19.09	1
30	85.56	0
45	138.33	9
54	144.54	9
72	197.76	12
54	152.46	9
35	137.41	7
45	138.33	0
54	135.54	0
72	173.76	0
54	138.33	0
45	138.33	9
54	139.14	9
72	197.76	12
54	148.32	9
54	135.54	9
72	173.76	12
54	152.46	9
35	137.41	7

5	11.28	1
10	22.56	2
4	38.18	2
2	16.82	2
6	16.82	2
3	6.19	1
40	164.8	10
40	164.8	10
16	128	8
146	1233.7	73
129	770.13	43
78	659.49	39
16	128	0
146	1233.7	0
129	770.13	0
78	659.49	0
5	18.74	1
5	18.74	1
18	87.06	6
18	87.06	6
18	130.59	9
18	87.06	6
18	87.3	6
18	130.95	9
18	93.06	6
18	92.7	6
18	148.86	9
2	17.34	0
4	30.28	0
3	48.27	0
4	34.26	2
9	51.93	3
328	1412.86	0
2	12.04	1
4	21.04	2
6	36.12	0
8	42.08	0
33	50.13	3
52	68.24	4
65	91.15	5
78	110.22	6
455	1164.15	65
774	1540.26	86
1239	3170.07	177
2289	5856.57	327
162	957.96	0
455	1153.1	0
774	1641.74	0
1239	3378.93	0
2289	6242.43	0
6	15.33	1

10	33.52	2
48	272.96	16
8	11.7	1
1128	1515.28	94
864	1160.64	72
744	1006.26	62
10	28.78	2
25	75.1	5
49	106.4	7
255	302.09	17
135	170.19	9
165	212.3	11
192	230.64	12
255	277.8	15
45	130.32	9
54	421.74	0
52	406.12	0
24	180.24	0
40	311.8	0
36	281.16	0
32	249.92	0
12	93.72	0
26	195.52	0
54	405.54	0
104	781.04	0
6		0
3	55.41	3
4	18.56	1
10	30.96	2
15	46.44	3
20	61.92	4
25	77.4	5
360	1709.1	90
224	1060.08	56
65	267.15	13
55	220	11
32	167.92	8
2	18.49	0
4	82.16	4
2	41.46	2
18	368.28	18
26	535.34	26
2	17.22	0
3	17.22	0
14	142.03	7
10	101.45	5
2	20.29	1
28	284.06	14
52	527.54	26
40	156.88	8
90	338.94	18

55	208.67	11
46	342.93	23
28	271.88	14
54	516.24	27
136	503.54	34
135	524.34	27
4	17.67	1
3	17.32	1
12	69.28	4
48	207.84	12
8	34.64	2
15	51.96	3
51	213.86	17
64	270.24	16
3	20.87	1
1	18.47	1
4	79.88	4
35	92.1	0
7	18.25	0
49	124.32	0
12	66.92	4
18	97.2	6
15	81	5
10	138.2	10
14	89.95	7
34	212.33	17
64	199.84	16
2	16.81	1
4	33.62	2
4	37.46	2
2	18.9	1
18	169.47	9
18	112.32	6
3	18.9	1
12	110.94	6
10	38.26	2
12	39.08	2
18	58.62	3
8	68.76	4
3	17.34	1
12	75.4	4
8	77.28	0
6	59.16	0
3	19.92	0
8	39.84	0
4	19.18	0
4	19.32	0
8	38.64	0
24	119.7	6
16	79.12	4
56	279.02	14

52	268.71	13
84	345.66	21
96	261.12	16
108	440.1	27
5	16.46	1
4	35.74	2
2	17.8	1
4	34.98	2
4	35.74	2
40	168	0
1	13.42	0
20	84	5
3	52.17	3
20	82.2	5
12	89.1	6
8	115.52	0
8	115.52	0
16	30.08	0
24	28.7	0
12	14.85	0
12	15.04	0
11	16.2	0
6	38.62	0
24	28.7	2
24	29.7	2
36	45.09	3
11	14.2	1
15	87.25	5
22	219.23	0
44	214.17	11
32	153.2	8
25	95.1	5
16	77.88	4
16	70.96	0
16	76.6	0
20	79.28	0
20	101.55	0
32	64.48	4
4	12.86	1
7	17.59	1
14	35.18	2
24	52.77	3
2	36.86	2
5	91.45	5
3	54.45	3
3	55.29	3
3	57.9	3
3	57.9	3
15	18.63	1
68	74.52	4
42	123.27	7

8	17.72	1
45	90	5
8	17.72	1
27	54	3
60	74.52	4
34	37.9	2
1	16.44	1
13	14.75	1
15	14.57	1
42	46.2	3
42	46.2	3
2	36.38	2
7	14.05	0
14	28.1	0
16	28.1	0
32	56.2	0
104	247.91	0
105	210.75	15
42	109.56	6
40	92.05	5
48	114.24	6
48	114.42	6
8	156.08	8
8	80.12	4
18	179.91	9
2	20.03	1
1	16.29	0
12	103.2	6
8	34.4	2
2	15.97	1
3	15.97	1
3	15.97	1
4	15.97	1
12	47.91	3
16	63.88	4
12	47.91	3
4	77.56	4
2	38.98	2
2	38.98	2
8	39.02	0
4	19.51	0
20	97.55	0
36	175.59	0
8	71.04	4
10	88.8	5
2	17.76	1
3	17.76	1
4	28.78	0
16	142	0
12	98.88	0
6	48.81	0

2	16.27	0
9	14.41	1
20	28.82	2
11	14.41	1
26	28.82	2
39	43.23	3
91	111.23	0
13	15.95	0
16	71.96	4
27	161.91	9
7	116.06	7
7	116.06	7
32	128.72	8
40	160.9	10
18	96.12	6
20	167.9	10
18	151.11	9
8	56.84	4
10	71.05	5
6	46.62	3
6	53.82	3
12	47.94	0
20	79.9	0
5	70.7	5
1	14.11	1
2	34.72	2
2	19.2	1
6	39.72	2
4	19.25	1
16	79.6	4
16	36.2	2
32	72.4	4
16	36.18	2
16	34.92	2
56	122.22	7
12	71.36	4
6	36.98	2
20	90.35	5
8	36.14	2
15	93	0
30	55.8	0
40	74.04	4
6	36.88	2
4	18.59	1
2	35.6	2
8	69.56	4
4	35.3	2
2	17.65	1
4	57.28	0
3	42.96	0
8	109.28	0

3	38.79	0
10	129.3	0
21	133.14	0
33	207.02	0
9	57.99	0
4	38.66	0
8	34.4	2
10	18.1	1
6	37.16	2
5	15.07	1
10	30.14	2
2	35.38	2
6	54.69	3
9	54.69	3
12	53.07	3
30	88.05	5
32	70.44	4
16	142.8	0
60	536.1	0
62	585.9	0
12	98.7	6
12	98.82	6
3	45.75	3
5	75.95	5
6	96.84	6
6	96.84	6
3	48.99	3
1	16.33	1
1	14.85	1
2	17.99	0
3	17.99	0
3	15.87	1
3	15.87	1
2	14.82	1
4	28.86	2
12	84.54	6
20	59.28	4
3	13.56	1
2	38.26	2
2	35.48	2
8	61.56	4
8	77.04	4
18	103.92	6
36	212.88	12
18	151.56	9
8	148.4	8
7	129.15	7
35	117.95	7
18	49.17	3
16	33.82	2
2	33.72	0

5	84.3	0
7	113.12	7
1	16.16	1
6	56.31	3
3	15.32	1
9	45.87	3
15	76.6	0
45	229.8	0
72	173.4	12
6	14.34	1
90	129.06	9
20	32.76	0
12	47.43	3
9	48.48	3
12	95.64	6
45	129.15	9
5	14.52	1
60	174.24	12
1	17.85	1
32	52.92	4
64	106.24	8
4	14.75	1
4	14.75	1
8	18.61	1
8	21.16	1
17	292.74	17
240	246.72	16
8	147.36	0
6	64.05	3
2	21.6	1
4	43.38	2
8	20.31	1
48	122.22	6
8	20.31	1
136	346.29	17
80	203.1	10
112	285.18	14
21	158.13	0
30	226.5	0
18	65.79	3
70	219	10
32	88.68	4
8	22.33	1
32	89.88	4
7	20.27	1
36	80.12	4
55	99.15	5
110	231.44	11
24	60.75	3
21	61.17	3
8	20.31	1

152	408.31	19
88	237.49	11
35	108.7	5
60	219.3	10
98	306.6	14
96	266.04	12
16	44.66	2
16	44.94	2
162	360.54	0
132	246.48	0
190	399.76	0
80	202.5	0
104	264.03	0
8	21.49	0
16	43.18	0
22	39.66	0
30	63.12	0
8	20.25	0
14	40.78	0
136	345.27	0
176	472.78	0
136	367.03	0
126	391.32	0
165	297.45	0
10	21.04	0
8	20.25	0
7	20.39	0
40	101.55	0
24	64.47	0
8	21.59	0
14	43.48	0
8	42.38	2
7	20.53	1
21	62.19	3
45	103.05	5
45	103.7	5
8	21.11	1
63	191.79	9
4	20.26	1
8	31.5	2
8	40.8	2
21	61.59	0
7	20.73	0
18	41.22	0
18	41.48	0
8	21.11	0
133	404.89	0
40	157.5	10
24	122.4	6
12	47.62	0
78	310.83	0

24	121.56	6
56	161.2	0
77	223.08	0
32	80.08	0
7	20.93	0
81	186.57	0
63	145.04	0
55	83.55	5
40	66.84	4
44	64.64	4
22	30.7	2
24	30.7	2
22	33.02	2
18	33.5	2
27	55.98	3
36	78	4
50	167.5	10
96	240.24	0
63	188.37	0
90	207.3	0
45	103.6	0
198	455.84	0
117	267.93	13
63	145.18	7
32	84.44	4
112	340.96	16
84	415.17	21
105	337.47	0
140	449.96	0
115	370.76	0
144	384	0
216	471.36	0
63	154.14	0
77	152.81	0
28	43.32	0
60	86.8	0
75	108.65	0
16	143.2	0
14	125.16	0
8	70.44	0
12	104.64	0
24	64	0
36	78.56	0
81	198.18	0
176	349.28	0
238	368.22	0
210	303.8	0
150	217.3	0
88	174.64	8
112	173.28	8
90	130.2	6

30	43.46	2
854	1321.26	61
735	1063.3	49
570	825.74	38
28	43.32	2
15	21.7	1
60	86.92	4
240	347.2	16
165	239.03	11
90	352.08	0
165	238.7	11
195	282.49	13
78	235.56	13
96	217.44	12
128	286.56	0
135	485.46	27
140	367.4	20
9	18.13	1
72	145.84	8
18	195.48	0
30	215.6	0
12	136.08	0
15	244.2	15
5	80.7	5
7	138.67	0
10	94.9	0
20	186.8	0
50	466.75	0
38	356.06	0
8	75.12	0
40	380.4	0
144	340.92	18
36	37.98	2
76	75.12	4
54	56.55	3
13	251.42	13
14	271.18	14
5	97.65	5
1	19.64	1
3	59.13	3
21	47.88	0
12	30.76	0
24	62.52	0
5	15.59	0
6	15.36	0
72	184.8	0
12	30.5	0
18	45.75	0
6	14.9	0
35	79.8	0
30	76.9	0

18	46.89	0
5	15.59	0
24	61.44	0
30	77	0
60	152.5	0
48	122	0
30	74.5	0
5	15.02	0
60	180.24	0
91	207.48	0
42	107.66	0
24	62.52	0
10	31.18	0
6	15.36	0
6	15.4	0
90	228.75	0
84	213.5	0
102	253.3	0
110	330.44	0
26	543.92	0
30	305.4	0
28	281.82	0
18	181.53	0
10	101.15	0
12	121.92	0
18	119.94	0
50	200.8	0
21	47.88	0
204	522.92	0
204	531.42	0
180	561.24	0
192	491.52	0
36	92.4	0
30	76.25	0
6	15.25	0
18	44.7	0
20	80.32	4
12	76.72	4
5	19.56	1
10	38.44	2
65	252.98	13
15	59.31	3
15	59.73	3
10	39.12	2
25	96.1	5
5	19.46	1
25	98.85	5
15	59.73	3
3	63.99	0
5	112.2	0
6	135.36	0

3	21.51	0
8	42.96	0
10	42.72	0
90	388.98	0
50	219.7	0
35	153.44	0
105	460.74	0
15	319.95	15
12	269.28	12
3	67.68	3
63	451.71	21
60	322.2	15
17	362.61	0
5	112.2	0
2	45.12	0
3	21.51	0
12	64.44	0
5	21.36	0
5	21.61	0
5	21.97	0
5	21.92	0
25	109.7	0
20	87.88	4
20	87.68	4
75	329.1	15
36	210.33	9
28	164.43	7
40	185.52	8
49	160.44	7
16	45.72	2
128	366.56	16
63	206.55	9
19	391.02	0
10	100.35	0
8	80.28	0
8	80.64	0
40	175.76	8
45	197.28	9
60	263.28	12
32	171.84	8
35	149.52	7
25	108.05	5
30	131.82	6
65	284.96	13
55	241.34	11
20	92.76	0
7	22.92	0
32	91.44	0
24	68.73	0
14	45.9	0
63	206.28	0

32	91.44	4
16	45.82	2
21	68.85	3
4	66.68	0
4	69.84	0
7	124.53	0
6	107.82	0
18	176.31	0
9	58.14	0
24	108.12	0
5	17.68	0
18	116.28	0
56	252.28	0
35	123.76	0
28	276.36	0
2	20.77	0
10	103.35	0
14	146.16	0
8	36.04	2
10	35.36	2
10	35.36	2
2	36.04	0
2	34.64	0
2	34.68	0
4	69.76	0
1	16.93	0
3	50.82	0
1	17.11	0
2	34.26	0
4	68.6	0
36	227.76	12
78	234.26	13
40	88.6	5
35	89.2	5
6	18.02	1
24	53.16	3
147	374.64	21
162	486.54	27
32	70.88	4
105	267.6	15
26	264.68	0
14	143.78	0
8	67.56	0
4	33.38	0
10	34	0
112	292.16	0
16	36.72	0
8	19.37	0
32	76.44	0
21	54.78	3
32	73.44	4

112	271.18	14
64	160.88	8
160	522.56	32
65	216.19	13
108	154.98	9
39	764.4	0
42	821.1	0
42	823.62	0
11	215.27	0
270	2535.3	0
690	2218.35	115
63	177.21	9
8	19.68	1
228	327.18	19
204	296.31	17
252	361.62	21
588	854.07	49
552	792.12	46
336	488.04	28
1824	2617.44	152
48	69.72	4
24	34.86	2
48	69.72	4
24	152	8
40	192.7	10
42	114.3	6
49	134.89	7
77	214.61	11
100	192.6	10
100	193.2	10
10	19.52	1
52	78.56	4
11	20.15	1
27	171	9
116	558.83	29
336	914.4	48
700	1927	100
686	1911.98	98
680	1309.68	68
70	135.24	7
10	19.52	1
104	157.12	8
33	60.45	3
30	57.78	0
30	57.96	0
20	39.04	0
520	785.6	0
396	725.4	0
174	1151.88	87
202	1313	101
117	556.14	39

72	353.04	24
165	809.05	55
309	1528.52	103
534	3104.32	178
1164	3594.82	194
1880	3639.68	188
4	19.27	1
210	571.5	30
259	712.99	37
273	760.89	39
990	1906.74	99
660	1275.12	66
150	292.8	15
273	412.44	21
407	745.55	37
546	1485.9	78
651	1792.11	93
742	2068.06	106
10	19.26	1
10	19.32	1
50	97.6	5
26	39.28	2
33	60.45	3
26	39.28	2
44	80.6	4
114	359.86	19
96	300.64	16
160	309.76	16
80	330.08	16
455	554.75	0
406	455.3	0
795	815.14	0
42	46.29	0
70	90.4	0
13	18.33	0
78	110.1	0
104	146.96	0
14	15.7	1
30	30.76	2
14	15.43	1
84	108.48	6
104	146.64	8
39	55.05	3
299	422.51	23
175	673.4	35
270	885.15	45
494	602.3	0
56	62.8	0
60	61.52	0
70	77.15	0
14	18.08	0

13	18.33	0
26	36.7	0
26	36.74	0
20	76.96	0
42	134.19	0
9	56.01	3
6	39.18	2
12	78.48	4
33	214.61	11
36	173.61	9
48	239.04	12
36	190.71	9
117	142.65	9
14	15.7	1
15	15.38	1
98	108.01	7
56	72.32	4
13	18.33	1
169	238.55	13
169	238.81	13
190	367.84	19
20	107.45	0
9	65.76	0
30	129.54	0
85	369.75	0
120	256.08	0
80	171.92	0
20	110.6	0
144	361.12	0
150	320.1	15
10	21.09	1
108	258.84	12
26	448.5	0
392	1763.02	98
640	2891.2	160
420	1501.92	84
480	2191.2	120
16	73.56	4
726	531.96	33
375	241.8	15
396	290.16	18
225	138.06	0
264	185.64	0
15	296.55	15
8	159.28	8
18	172.08	9
104	1033.24	52
86	852.69	43
104	1030.64	52
74	735.19	37
46	460.46	23

60	599.7	30
66	656.7	33
60	372.6	0
63	316.05	0
3	18.51	0
15	94.95	0
32	152.72	0
20	95.85	0
28	134.26	0
48	221.88	0
30	112.14	0
20	68	4
24	102.96	6
10	33.8	2
30	100.74	6
55	187	0
105	354.9	0
115	386.17	0
9	15.87	1
12	24.04	2
12	32.24	2
4	43.18	2
2	21.65	1
8	17.45	0
8	17.66	0
8	17.62	0
18	31.74	0
3	19.27	1
5	18.77	1
6	17.85	1
91	229.19	13
77	190.74	11
77	192.83	11
6	19.63	1
18	58.95	3
18	53.55	0
14	34.68	0
18	52.89	0
24	78.6	0
4	89.76	4
4	90	4
4	90	4
6	65.46	3
8	87.44	4
4	43.82	2
26	218.01	13
16	134.48	8
20	167.7	10
4	33.54	2
8	65.04	4
24	126.88	8

12	63.96	4
18	96.9	6
45	241.8	15
39	206.44	13
15	79.3	5
21	111.93	7
15	80.75	5
357	1918.28	119
51	269.96	17
17	307.53	17
50	423.75	25
28	445.2	28
494	677.54	38
245	784.98	49
10	33.86	2
63	147.24	9
48	101.76	6
28	71.04	4
35	90.65	5
56	146.72	8
78	106.98	6
117	160.47	9
28	107.94	7
12	46.95	3
25	80.1	5
15	50.79	3
7	16.36	1
8	16.96	1
7	17.76	1
14	36.26	2
21	55.02	3
78	106.98	6
143	196.13	11
160	339.2	20
35	88.8	5
14	36.26	2
21	55.02	3
39	53.49	3
3	56.28	3
2	37.7	2
4	75.72	4
6	114.06	6
90	151.65	9
70	119.91	7
286	452.14	26
15	18.48	0
170	186.8	0
220	204.27	0
630	561	0
525	468.25	0
377	524.32	0

30	36.96	0
85	93.4	0
20	18.57	0
168	149.6	0
126	112.38	0
5	18.15	0
26	36.16	0
90	110.88	0
102	112.08	0
140	129.99	0
84	74.8	0
42	37.46	0
10	36.3	0
65	90.4	0
120	147.84	0
1241	1363.64	0
860	798.51	0
819	729.3	0
294	262.22	0
156	216.96	12
165	203.28	11
102	112.08	6
120	111.42	6
189	168.3	9
21	18.73	1
21	51.33	3
15	91.55	5
4	32.64	2
12	92.22	6
18	137.25	9
10	34.24	2
3	42.09	3
6	83.82	6
8	112	8
14	92.68	7
6	39.75	3
7	17.11	1
15	51.36	0
90	186.9	10
285	368.79	19
528	640.53	33
765	873.45	45
45	209.4	15
504	987.84	0
711	1359.59	0
410	1460.42	0
105	358.26	0
60	208.32	0
135	512.19	0
285	974.13	0
12	35.96	0

2	19.29	0
6	57.9	0
198	388.08	0
792	1602.48	0
441	2879.73	0
30	200.7	0
36	244.08	0
8	40.64	0
30	121.86	0
54	180.27	0
138	462.07	0
91	261.04	0
12	234.6	0
6	118.32	0
4	79.8	0
2	40.8	0
2	41.08	0
5	20.31	1
52	265.2	13
66	220.33	11
48	160.72	8
7	20.08	1
18	106.02	6
150	923	50
188	859.63	47
150	1036.5	75
148	1018.24	74
50	361	25
28	204.26	14
40	313.8	20
46	338.79	23
160	1232	80
381	2213.61	127
381	2213.61	0
332	3026.18	166
711	4171.2	237
132	570.57	33
168	741.72	42
322	851.92	46
392	570.85	49
312	533.91	0
32	54.72	0
16	26.7	0
144	262.8	0
234	408.98	0
1328	2216.1	166
1552	2832.4	194
9	15.73	1
8	27.8	0
4	13.93	0
4	15.66	0

8	30.3	0
8	15.89	0
8	17.16	0
136	301.41	0
245	672.35	0
52	241.41	13
112	528.64	28
126	396.9	21
72	229.08	12
72	231.24	12
54	174.78	9
120	399.6	20
80	325.28	16
34	285.43	17
3	16.49	1
24	111.42	6
20	94.4	5
42	132.3	7
30	95.45	5
6	19.27	1
84	271.88	14
156	519.48	26
5	20.33	1
21	115.43	0
12	55.71	0
32	151.04	0
108	340.2	0
66	209.99	0
48	154.16	0
36	116.52	0
30	99.9	0
25	101.65	0
23	343.16	0
14	208.88	0
27	402.84	0
238	526.66	0
189	409.59	0
14	32.04	0
28	65.4	0
7	18.53	0
7	18.53	0
14	37.06	0
2	33.26	2
5	81.4	5
1	16.43	1
4	67.48	4
36	191.52	12
36	186.72	12
6	33.36	2
6	53.49	3
2	37.64	2

18	32.36	2
54	97.08	6
81	145.62	9
45	153.9	0
28	119.49	0
96	403.68	0
60	253.2	0
40	161.7	10
24	98.88	6
20	82.05	5
36	158.04	9
27	159.21	9
7	129.64	7
39	239.85	13
27	163.44	9
81	506.52	27
12	114.6	6
6	57.42	3
2	19.04	1
2	19.08	1
10	18.3	0
120	183.6	0
1605	1957.03	0
3290	4307.55	0
2988	4571.64	249
90	110.16	6
294	391.23	21
215	695.74	43
235	767.98	47
325	984.1	65
360	1196.64	72
10	29.1	2
10	36.66	2
10	37.36	2
60	185.6	10
300	929.5	50
320	1032.96	64
290	935.54	58
7	17.47	1
18	34.46	2
16	34.84	2
20	36.18	2
27	51.69	0
160	289.44	0
135	273.75	0
122	1134.6	61
12	112.44	6
12	35.2	2
9	17.44	1
22	34.3	2
24	34.44	2

12	46.95	0
98	262.78	0
64	150.16	0
120	183.9	0
98	138.95	7
22	40.32	2
66	120.42	6
84	136.22	7
72	116.46	6
81	167.13	9
54	160.38	9
48	224.76	12
126	255.5	14
88	152.88	8
72	214.56	12
306	919.53	51
456	1380.16	76
1152	1653.12	96
36	105.6	6
27	52.32	3
44	68.6	4
36	51.66	3
6	17.6	1
99	191.84	11
121	188.65	11
84	120.54	7
158	1521.54	79
528	2471.04	132
3	19.4	1
8	40.26	2
96	281.6	16
9	17.44	1
22	34.3	2
288	413.28	24
196	534.24	28
117	249.34	13
84	119.1	6
242	443.52	22
165	301.05	15
12	19.46	1
240	388.2	20
495	1021.35	55
408	1211.76	68
35	95.4	0
36	76.72	0
98	138.95	0
33	60.48	0
253	461.61	0
24	38.92	0
12	19.41	0
252	519.96	0

198	588.06	0
154	412.94	22
112	263.2	14
384	620.48	32
84	383.04	21
5	18.12	1
20	72.2	4
66	178.97	11
60	96.54	6
66	102	6
72	102.06	6
8	36.48	0
20	72.96	0
5	18.24	0
8	35.42	0
36	110.28	0
64	145.04	0
56	128.73	0
24	56.88	0
1	16.08	1
18	314.28	18
19	332.12	19
36	630	36
5	87.2	5
12	233.76	12
22	206.36	11
20	192.3	10
2	18.51	1
6	50.91	3
42	128.66	7
32	72.52	4
248	539.09	31
296	643.43	37
86	750.35	0
36	318.24	0
70	624.75	0
148	1400.08	0
122	1164.49	0
162	1555.2	0
15	10.06	1
5	19.28	1
8	38.64	2
75	289.2	15
13	282.49	0
21	454.23	0
62	643.25	0
52	534.3	0
8	81.52	0
6	53.34	0
126	1125.18	0
82	736.36	0

52	459.16	0
2	20.75	0
58	595.95	0
68	692.92	0
72	640.08	0
64	571.52	0
2	17.96	0
4	35.32	0
19	327.56	0
22	190.85	0
38	329.84	0
34	294.78	0
4	34.6	0
6	52.26	0
14	27.82	2
42	80.76	6
30	68.1	5
6	16.6	1
24	55.02	3
150	180.6	10
90	108.36	6
9	56.04	0
21	132.23	0
57	360.24	0
57	360.81	0
123	758.09	0
306	1953.3	0
276	1762.72	0
4	39.68	0
12	57.96	0
96	954.24	0
84	1556.52	84
65	1218.1	65
430	1480.06	86
774	2209.77	129
702	2006.55	117
1040	2198.3	130
144	147.33	9
120	204.36	12
70	129.78	7
320	1048.32	64
375	1156.5	75
5	16.46	1
10	32.92	2
16	38.72	2
7	19.53	1
8	19.44	1
488	1179.74	61
432	826.08	48
816	1773.78	102
549	1037	61

72	152.91	9
42	112.5	6
112	319.68	0
196	554.4	0
256	633.6	0
72	179.82	0
31	608.84	0
152	1439.44	0
285	1902.85	0
16	38.72	2
28	78.12	4
32	77.76	4
8	19.34	1
15	60.6	3
189	539.46	27
224	633.6	32
176	435.6	22
352	879.12	44
14	39.96	2
56	158.4	8
48	118.8	6
48	119.88	6
12	82.4	0
70	282.8	0
210	599.4	0
217	613.8	0
296	732.6	0
320	799.2	0
248	613.8	31
8	19.98	1
19	376.2	19
105	2254.35	105
34	355.13	17
54	370.8	18
10	40.4	2
7	19.98	1
77	217.8	11
24	59.4	3
8	19.98	1
3	17.04	1
3	21.71	0
78	282.23	0
20	111.3	0
25	111.05	0
10	89.8	5
6	54.93	3
30	268.95	15
6	55.59	3
4	37.5	2
2	18.59	1
8	73.12	0

6	55.02	0
4	35.94	0
2	17.48	0
3	17.08	0
24	100.8	0
2	18.28	0
2	18.34	0
2	17.97	0
4	34.96	0
15	85.4	0
32	134.4	0
35	116.97	0
150	493.8	0
140	466.48	0
15	49.8	0
30	90.36	0
57	383.99	0
110	434.5	0
60	407.4	0
150	605.7	0
48	161.12	0
30	100.26	0
30	98.76	0
40	133.28	0
40	132.8	0
115	346.38	0
10	39.5	0
10	33.42	0
5	16.46	0
5	16.66	0
35	116.2	0
55	165.66	0
15	59.25	0
12	40.28	0
948	1397.51	79
1296	1920.24	108
24	35.68	2
12	17.69	1
24	35.56	2
60	89.2	5
24	35.38	2
1440	2133.6	120
1332	1980.24	111
68	222.53	17
68	252.62	17
36	157.59	9
256	549.76	32
36	53.07	3
240	355.6	20
144	214.08	12
88	188.98	11

48	105.54	6
56	120.26	7
32	70.36	4
16	35.18	2
3	21.34	1
6	43.18	2
90	650.7	30
54	311.76	0
150	866	0
198	1143.12	0
66	964.26	0
4	57.16	0
4	65.4	0
6	97.2	0
18	179.01	9
18	182.16	9
12	61.29	0
4	20.46	0
20	103	0
4	20.59	0
4	20.71	0
16	82.96	4
4	20.68	1
261	1832.22	87
420	2238.6	105
1140	2325.6	114
894	2445.09	149
600	1667	100
564	1587.66	94
648	1712.88	108
630	1747.2	105
6	16.58	1
6	17.35	1
200	425.75	25
3	17.69	1
18	108.36	6
18	109.92	6
12	106.8	0
6	111.3	0
10	169.8	0
26	483.86	0
39	726.57	0
22	411.62	0
35	666.4	0
2	37.94	0
8	151.6	0
14	266.28	0
26	224.25	0
4	70.44	0
70	255.78	14
114	343.71	19

156	470.34	26
54	308.34	18
3	17.72	1
3	17.73	1
6	35.42	2
3	17.66	1
4	17.39	1
594	1128.06	0
517	985.12	0
24	134.16	0
30	77.35	5
432	376.56	24
19	15.97	1
960	471.68	32
217	120.05	7
78	454.22	26
141	824.38	47
72	310.14	18
486	492.21	27
320	574.08	32
99	162.27	9
143	204.38	11
36	36.46	2
30	53.82	3
847	1388.31	77
1235	1765.1	95
1476	1494.86	82
2	19.58	1
3	19.18	1
20	94.15	5
48	222	12
312	1375.14	78
1098	1112.03	61
36	36.46	2
16	36.32	2
210	379.47	21
231	364.98	21
336	462.28	28
276	379.73	23
696	957.58	58
528	1108.14	66
24	54.21	3
54	103.62	6
63	130.69	7
40	72.28	0
44	72.28	0
48	72.28	0
144	216.84	0
180	271.05	0
48	193.2	12
76	304.38	19

16	64.04	4
10	31.5	2
45	141.93	9
264	727.76	44
25	83.95	5
5	113.8	5
1	22.85	1
2	45.82	2
6	67.32	3
4	45.1	2
9	66.75	3
2	22.67	1
48	316.64	16
42	44.76	3
32	78.16	0
18	38.78	0
80	194.8	0
20	63.88	4
2	18.45	0
4	37.12	0
9	170.82	0
11	213.07	0
3	57.75	0
1	19.17	0
1	18.08	0
2	35.94	0
4	71.36	0
2	36.56	0
3	46.74	0
3	46.23	0
11	207.35	0
8	149.28	0
70	122.78	7
42	258.86	14
48	295.84	16
4	18.49	1
30	110.94	6
60	110.94	6
20	191.7	10
12	115.8	6
6	37.8	2
12	75.12	4
8	38.7	2
42	133.56	7
8	75.8	0
2	18.95	0
3	19.49	0
9	58.47	0
20	94.85	0
100	369	0
190	352.26	0

46	262.66	0
62	565.44	0
48	434.4	0
26	269.75	0
92	913.56	0
114	1141.14	0
2	20.31	0
8	19.4	1
16	38.92	2
84	235.2	12
80	194.4	10
143	217.69	11
225	302.7	15
294	428.19	21
70	98.75	5
60	99.2	5
285	383.42	19
168	244.68	12
14	19.75	1
48	79.36	4
32	72.6	0
24	55.11	0
56	149.2	0
49	132.23	0
84	235.68	0
135	171.54	0
435	550.42	0
408	453.6	0
7	18.24	1
48	108.9	6
64	146.96	8
70	186.5	10
30	94.4	5
42	113.34	6
28	78.56	4
60	76.24	4
30	37.96	2
17	18.9	1
15	19.06	1
120	151.84	8
34	37.8	2
14	36.48	2
40	90.75	5
16	36.74	2
91	242.45	13
240	755.2	40
63	170.01	9
49	137.48	7
75	95.3	5
165	208.78	11
204	226.8	12

68	637.16	34
4	37.28	2
24	31.56	2
45	57.18	3
30	37.96	2
51	56.7	3
14	239.26	14
5	82.35	5
4	67.12	4
1	17.13	1
7	116.13	7
13	233.22	13
15	268.65	15
15	268.95	15
210	3803.1	210
153	2923.83	153
42	109.44	6
56	127.05	7
24	55.11	3
42	111.9	6
6	18.88	1
42	113.34	6
231	648.12	33
405	514.62	27
375	474.5	25
17	18.9	1
17	18.9	1
5	16.12	1
5	17.53	1
6	112.92	0
7	131.74	0
8	72.52	0
20	64.48	4
45	157.77	9
14	288.4	14
8	138.08	8
4	70.92	4
2	34.12	2
18	304.74	18
18	352.8	18
12	239.28	12
8	161.6	8
19	389.5	19
24	496.8	24
90	590.7	30
126	839.58	42
6	39.92	2
16	79	4
8	39.76	2
9	59.07	3
6	39.98	2

3	19.96	1
44	217.25	11
12	59.64	3
48	237	12
16	79.52	4
3	54.66	3
3	55.17	3
36	262.8	12
20	68.04	4
12	38.84	2
12	39	2
60	195.8	10
110	209.99	11
53	1081.73	53
5	103.5	5
4	83.24	4
18	82.44	6
18	82.44	6
7	129.57	7
32	132.56	8
60	231.9	15
20	162	0
18	167.31	9
68	332.86	17
4	19.59	1
18	62.85	3
6	21.05	1
6	21.11	1
2	40.6	0
2	40.7	0
1	20.56	0
5	101.1	0
6	121.44	0
12	243	0
9	182.7	0
1	20.59	0
4	81.32	0
8	162.72	0
55	194.26	11
96	215.76	12
160	366.6	20
448	1048.32	56
175	618.1	0
168	377.58	0
136	294.61	0
144	317.88	0
81	470.34	0
6	34.54	0
30	148.9	0
18	94.5	0
66	355.74	0

32	244.96	0
102	769.08	0
99	583.11	33
111	650.83	37
111	647.5	37
150	551.4	30
132	403.92	22
160	343.6	20
126	247.52	14
70	257.32	0
138	422.74	0
148	688.2	37
16	76.32	4
144	669.6	36
192	915.84	48
2	21.37	0
21	149.38	0
6	42.78	0
3	21.22	0
15	106.2	0
9	63.75	0
9	65.61	0
3	21.93	0
69	536.59	0
27	481.14	0
1	17.17	0
8	65.84	0
12	104.1	0
9	56.13	0
1	19.92	0
22	216.26	0
30	295.95	0
40	398.4	0
2	19.92	0
2	18.22	0
6	54.69	0
9	53.88	0
6	57.15	0
12	57.93	0
16	79.2	0
18	143.28	0
18	161.1	0
18	107.16	0
9	54.09	0
18	162.45	0
68	302.77	17
128	611.2	32
4	19.1	1
4	19.08	1
10	18.29	1
24	118.8	6

200	365.8	20
10	18.29	1
50	91.45	5
26	491.92	26
2	38.58	2
1	19.15	1
118	1100.94	59
6	54.93	3
312	1971.84	104
6	38	2
188	893.94	47
36	170.28	9
124	2416.76	0
52	1008.28	0
1	19.75	0
16	158.64	0
64	632.32	0
26	256.88	0
18	166.86	0
78	721.11	0
84	832.86	0
84	829.92	0
0	0	0
0	0	0
0	0	0
0	0	0
0	0	0
0	0	0
11	176.88	11
135	2170.8	135
115	1849.2	115
0	0	0
0	0	0
45	160.38	9
0	0	0
0	0	0
0	0	0
0	0	0
0	0	0
0	0	0
0	0	0
0	0	0
3	20.33	1
0	0	0
0	0	0
0	0	0
0	0	0
38	362.9	0
34	326.57	17
42	403.2	21
98	937.86	49

92	878.6	46
112	599.2	28
2280	3299.92	152
60	86	4
28	43.18	2
68	84.96	4
128	170.56	8
170	210.8	10
108	127.44	6
126	149.17	7
198	233.2	11
190	212.6	10
150	217.1	10
0	0	0
0	0	0
0	0	0
16	21.32	1
68	84.32	4
18	21.24	1
162	191.79	9
522	614.8	29
912	1020.48	48
1500	2171	100
1470	2107	98
952	1468.12	68
119	148.68	7
16	21.32	1
136	168.64	8
54	63.72	3
54	63.93	3
54	63.6	3
0	0	0
0	0	0
0	0	0
0	0	0
36	42.4	0
0	0	0
0	0	0
600	860	40
504	777.24	36
1479	1847.88	87
1616	2153.32	101
663	822.12	39
432	509.76	24
990	1172.05	55
1854	2183.6	103
3382	3784.28	178
582	2677.2	194
564	3517.48	188
7	17.18	1
0	0	0

0	0	0
0	0	0
0	0	0
0	0	0
0	0	0
0	0	0
210	621.6	30
333	768.12	37
273	808.08	39
891	2055.24	99
594	1370.16	66
75	271.65	15
126	391.44	21
222	689.68	37
546	1464.84	78
651	1811.64	93
530	1919.66	106
0	0	0
0	0	0
0	0	0
0	0	0
0	0	0
6	18.64	0
7	18.78	0
35	97.4	0
0	0	0
0	0	0
0	0	0
0	0	0
0	0	0
0	0	0
12	36.14	0
27	55.44	3
18	36.96	0
28	83.88	4
95	413.06	19
80	355.2	16
112	335.52	0
48	271.52	16
105	575.75	35
87	484.01	29
212	880.86	53
15	45.3	3
50	97.8	5
6	23.12	1
42	138.54	6
8	144.96	8
0	0	0
0	0	0
0	0	0
4	19.86	1

0	0	0
0	0	0
0	0	0
0	0	0
0	0	0
0	0	0
0	0	0
0	0	0
0	0	0
0	0	0
0	0	0
8	43.88	2
0	0	0
12	19.55	1
18	124.32	0
24	164.72	0
12	63.54	0
0	0	0
0	0	0
0	0	0
0	0	0
92	512.67	0
210	782.6	0
450	996.75	0
342	854.24	0
16	84.72	4
24	83.28	4
25	106.9	5
5	21.42	1
5	21.67	1
0	0	0
20	44.3	2
18	44.96	2
32	71.64	4
56	125.65	7
0	0	0
27	54.54	3
20	36.26	2
40	72.16	4
110	197.01	11
117	158.58	9
144	214.2	12
108	162.99	9
0	0	0
0	0	0
72	162.63	0
4	21.21	0
0	0	0
4	21.21	1
49	154.91	7
16	79.48	4

4	19.84	1
0	0	0
0	0	0
0	0	0
195	193.57	13
234	225.94	13
342	229.71	19
60	62.45	5
0	0	0
0	0	0
45	49.65	3
96	99.3	6
136	257.38	17
96	193.32	12
56	128.8	8
70	82.45	0
224	263.36	0
225	248.25	0
0	0	0
4	18.14	0
120	199.68	12
312	425.62	26
1372	1616.02	98
2240	2633.6	160
1260	1390.2	84
1920	1986	120
0	0	0
64	66.2	4
0	0	0
198	663.3	33
75	317.7	15
162	367.92	18
117	180.99	9
60	252.6	12
90	301.5	0
104	160.88	0
45	180.9	9
312	1045.2	52
559	864.73	43
260	1094.6	52
185	743.7	0
138	462.3	0
270	613.2	0
429	663.63	0
320	412.6	0
336	434.28	0
0	0	0
0	0	0
0	0	0
20	20.46	0
0	0	0

0	0	0
0	0	0
115	100.05	0
128	165.04	8
80	103.4	5
112	145.18	7
180	245.88	12
138	120.06	6
80	81.84	4
0	0	0
0	0	0
0	0	0
84	129.06	6
46	40.02	0
96	123.78	6
176	227.48	11
336	435.54	21
345	471.27	23
23	20.01	1
40	40.92	2
40	41.48	2
42	41.86	2
19	21.14	1
14	21.51	1
15	20.49	0
23	20.01	0
40	40.92	0
20	20.74	0
21	20.93	0
23	20.01	0
0	0	0
247	274.82	0
253	220.11	0
220	225.06	0
0	0	0
0	0	0
0	0	0
0	0	0
0	0	0
0	0	0
0	0	0
0	0	0
19	21.14	0
0	0	0
0	0	0
0	0	0
0	0	0
30	54.81	3
27	55.62	3
16	37.38	2
6	62.13	3

8	85.32	4
8	87.08	4
8	87.2	4
0	0	0
36	78.6	4
24	60.75	3
32	82.52	4
18	40.96	2
117	267.93	13
0	0	0
0	0	0
0	0	0
0	0	0
0	0	0
24	99.28	8
30	122.3	10
6	43.36	2
12	87.12	4
16	178.16	0
8	89.84	0
12	137.04	0
30	341.7	0
39	288.34	0
15	111.05	0
21	156.24	0
15	111.7	0
0	0	0
0	0	0
0	0	0
0	0	0
833	1680.28	119
0	0	0
0	0	0
0	0	0
0	0	0
0	0	0
0	0	0
0	0	0
119	240.04	17
136	255.68	17
250	368.75	25
280	454.72	28
380	639.16	38
490	863.87	49
26	34.94	2
108	157.77	9
42	84.72	6
32	60.16	4
50	73.75	5
80	129.92	8
60	100.92	6

90	158.67	9
91	122.29	7
36	52.59	3
10	77.95	5
24	56.1	3
8	18.67	1
8	18.7	1
8	18.67	1
0	0	0
0	0	0
0	0	0
0	0	0
0	0	0
0	0	0
0	0	0
0	0	0
0	0	0
0	0	0
0	0	0
0	0	0
0	0	0
0	0	0
0	0	0
4	33.56	0
0	0	0
6	50.49	0
12	101.52	0
22	186.67	0
20	336	20
0	0	0
0	0	0
0	0	0
0	0	0
20	81.8	5
8	33.38	2
24	54.66	3
24	55.23	3
12	48.36	3
10	31.94	2
36	56.56	4
18	100.62	0
27	160.56	0
42	107.38	0
130	419.12	0
6	15.92	0
60	159.2	10
33	179.63	0
30	503.4	30
25	417	25
29	474.44	29
0	0	0

0	0	0
0	0	0
0	0	0
6	30.58	2
10	87.85	5
0	0	0
2	18.9	1
0	0	0
0	0	0
0	0	0
56	149.68	8
30	115.38	6
2	16.67	1
4	35.86	2
12	111.12	6
6	115.74	6
7	129.85	7
0	0	0
0	0	0
0	0	0
0	0	0
0	0	0
0	0	0
0	0	0
0	0	0
0	0	0
4	63.16	4
2	33.02	2
4	37.54	0
0	0	0
0	0	0
10	100.25	0
40	155.36	0
292	1464.38	0
172	702.62	43
234	613.86	39
70	241.36	14
60	207.72	12
77	155.76	11
48	80.82	6
48	82.56	6
72	129.69	9
7	14.16	0
24	43.41	0
40	71.9	0
16	30.82	0
6	116.88	0
9	137.25	9
10	35.66	2
15	53.49	0
30	119.64	0

48	167.6	0
0	0	0
0	0	0
14	129.29	7
3	55.38	0
2	18.4	0
27	38.64	0
90	127.8	0
171	245.29	0
297	411.18	0
450	548.1	0
0	0	0
60	209.7	15
224	836.08	56
553	1193.69	79
574	1219.34	82
147	312.27	21
24	182.16	12
54	336.15	27
57	961.59	0
4	34.28	0
2	17.14	0
9	51.96	0
66	381.04	22
264	1298.88	88
588	2146.2	147
30	147.6	0
48	187.2	0
16	34.58	2
0	0	0
0	0	0
0	0	0
12	93.9	6
18	141.84	9
138	402.5	23
52	217.36	13
60	200.88	12
48	97.2	6
12	61.68	4
6	35.92	0
4	33.38	2
3	15.75	0
39	213.72	0
33	186.89	11
24	121.36	8
0	0	0
14	18.12	0
0	0	0
24	93.6	6
950	769	50
893	722.86	0

75	1291.5	75
296	1387.5	0
50	391.5	0
28	208.32	14
100	309.8	0
18	58.62	0
48	162.16	0
